# Supplementary figures and images for: Large, three-generation human families reveal post-zygotic mosaicism and variability in germline mutation accumulation (part 1 of 7)
Source: eLife. 2019 Sep 24;8:e46922. doi: 10.7554/eLife.46922 (PMC6759356; doi:10.7554/eLife.46922)

9

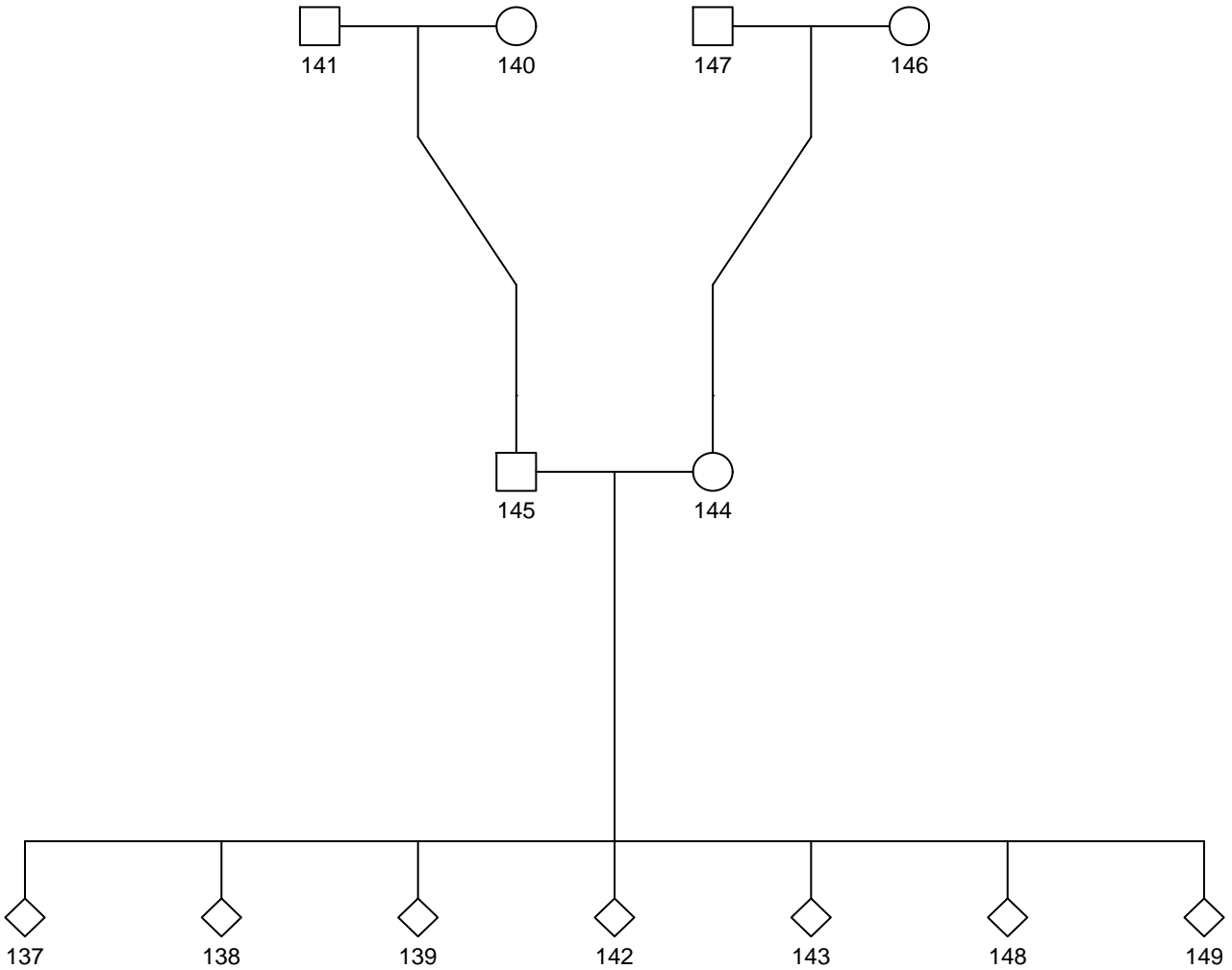

Supplement: Supplementary file 1. — All family and sample IDs have been anonymized, and the sexes of third-generation individuals have been hidden. [file elife-46922-supp1.zip › supp_file_1/9.pdf]

8

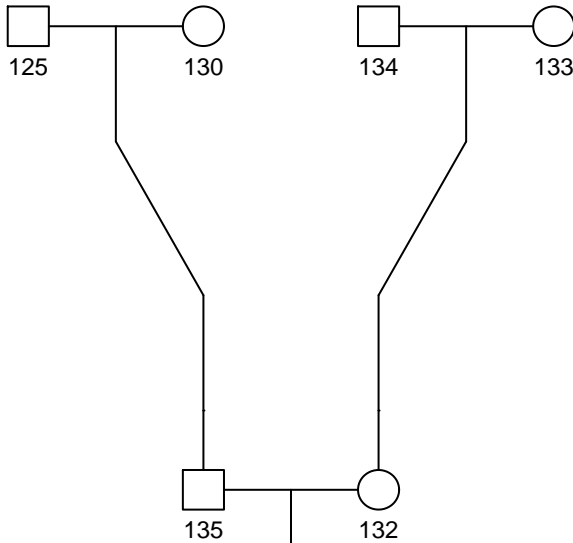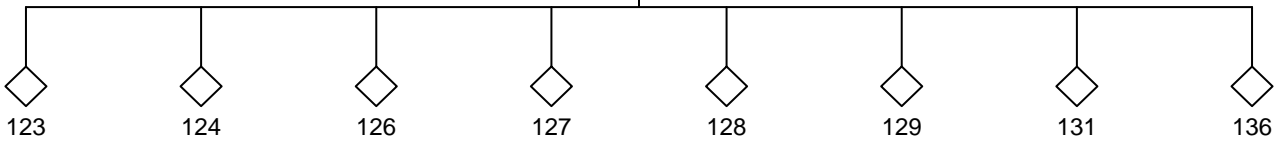

Supplement: Supplementary file 1. — All family and sample IDs have been anonymized, and the sexes of third-generation individuals have been hidden. [file elife-46922-supp1.zip › supp_file_1/8.pdf]

16

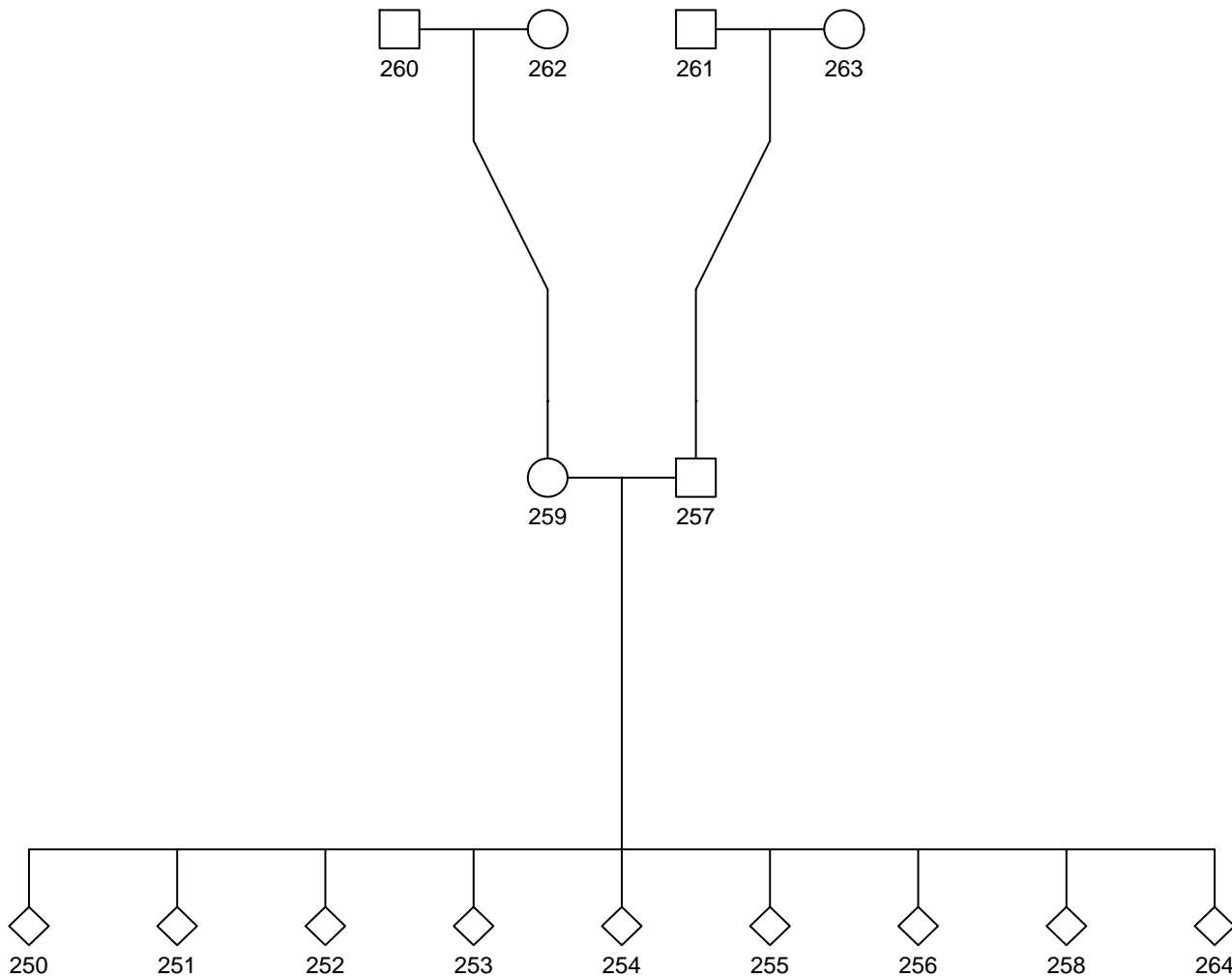

Supplement: Supplementary file 1. — All family and sample IDs have been anonymized, and the sexes of third-generation individuals have been hidden. [file elife-46922-supp1.zip › supp_file_1/16.pdf]

17

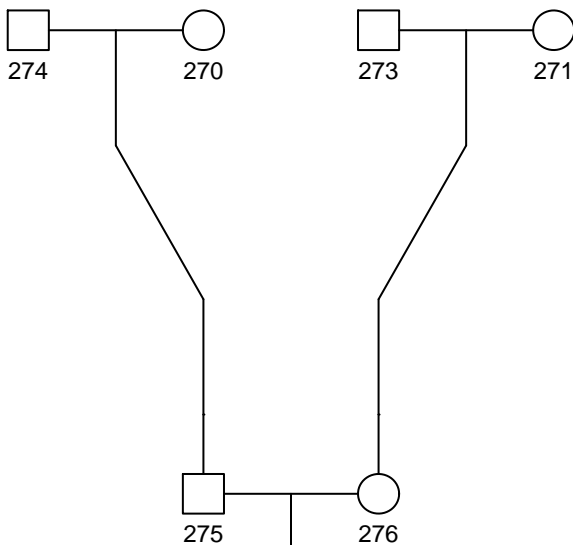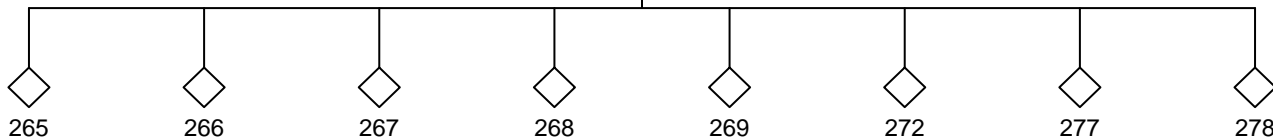

Supplement: Supplementary file 1. — All family and sample IDs have been anonymized, and the sexes of third-generation individuals have been hidden. [file elife-46922-supp1.zip › supp_file_1/17.pdf]

29

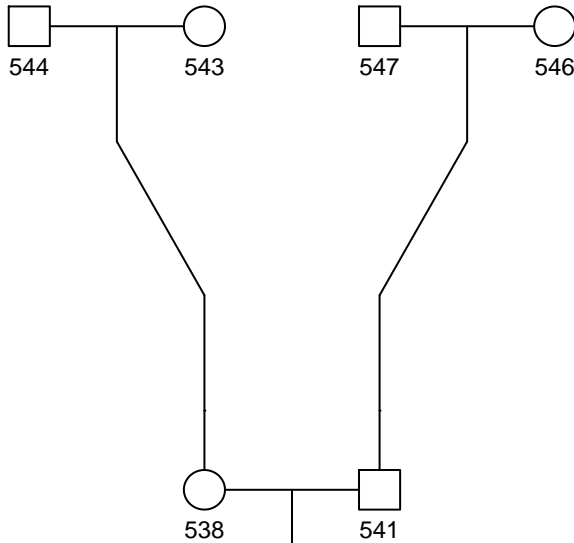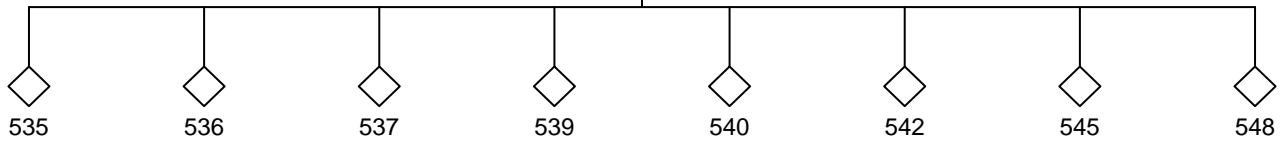

Supplement: Supplementary file 1. — All family and sample IDs have been anonymized, and the sexes of third-generation individuals have been hidden. [file elife-46922-supp1.zip › supp_file_1/29.pdf]

15

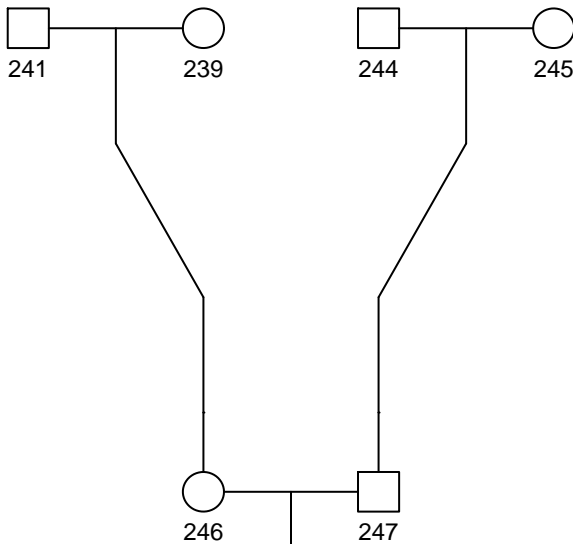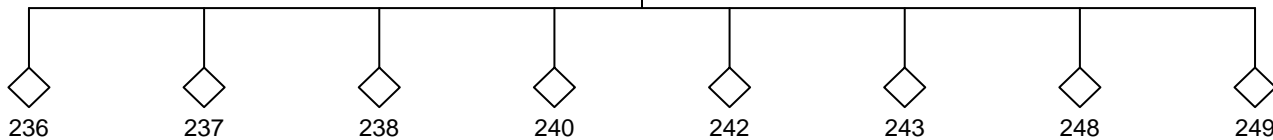

Supplement: Supplementary file 1. — All family and sample IDs have been anonymized, and the sexes of third-generation individuals have been hidden. [file elife-46922-supp1.zip › supp_file_1/15.pdf]

14

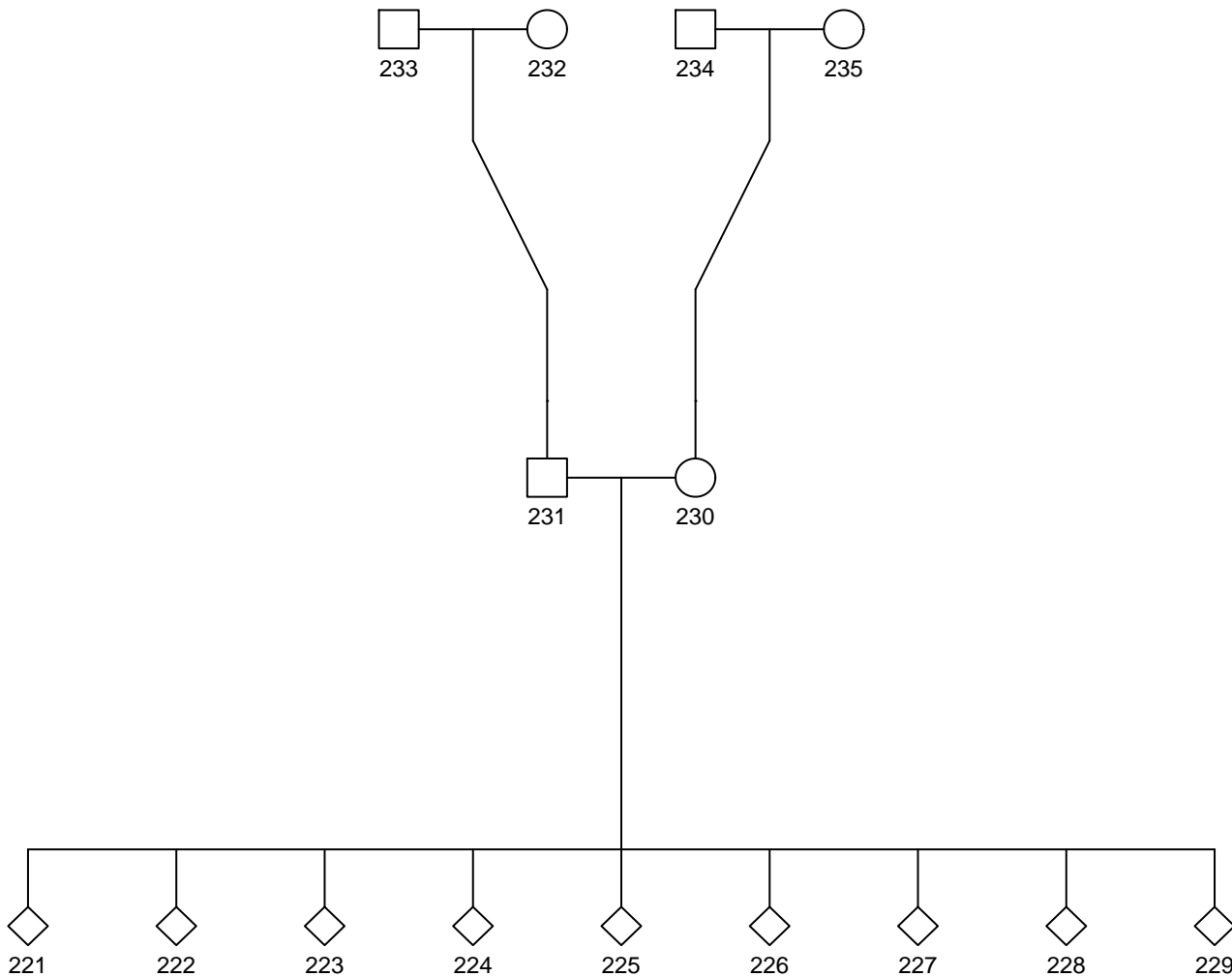

Supplement: Supplementary file 1. — All family and sample IDs have been anonymized, and the sexes of third-generation individuals have been hidden. [file elife-46922-supp1.zip › supp_file_1/14.pdf]

**28**

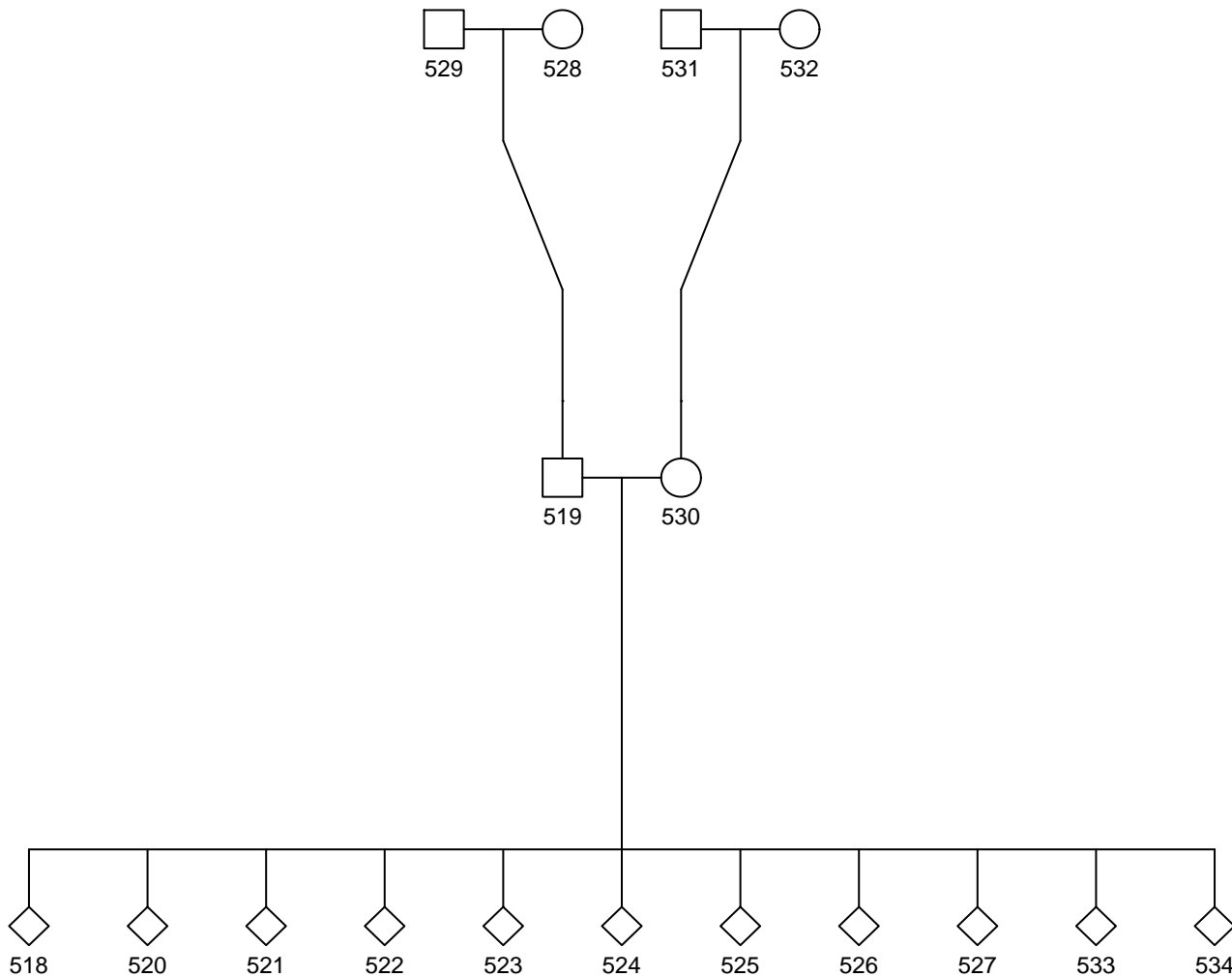

Supplement: Supplementary file 1. — All family and sample IDs have been anonymized, and the sexes of third-generation individuals have been hidden. [file elife-46922-supp1.zip › supp_file_1/28.pdf]

**10**

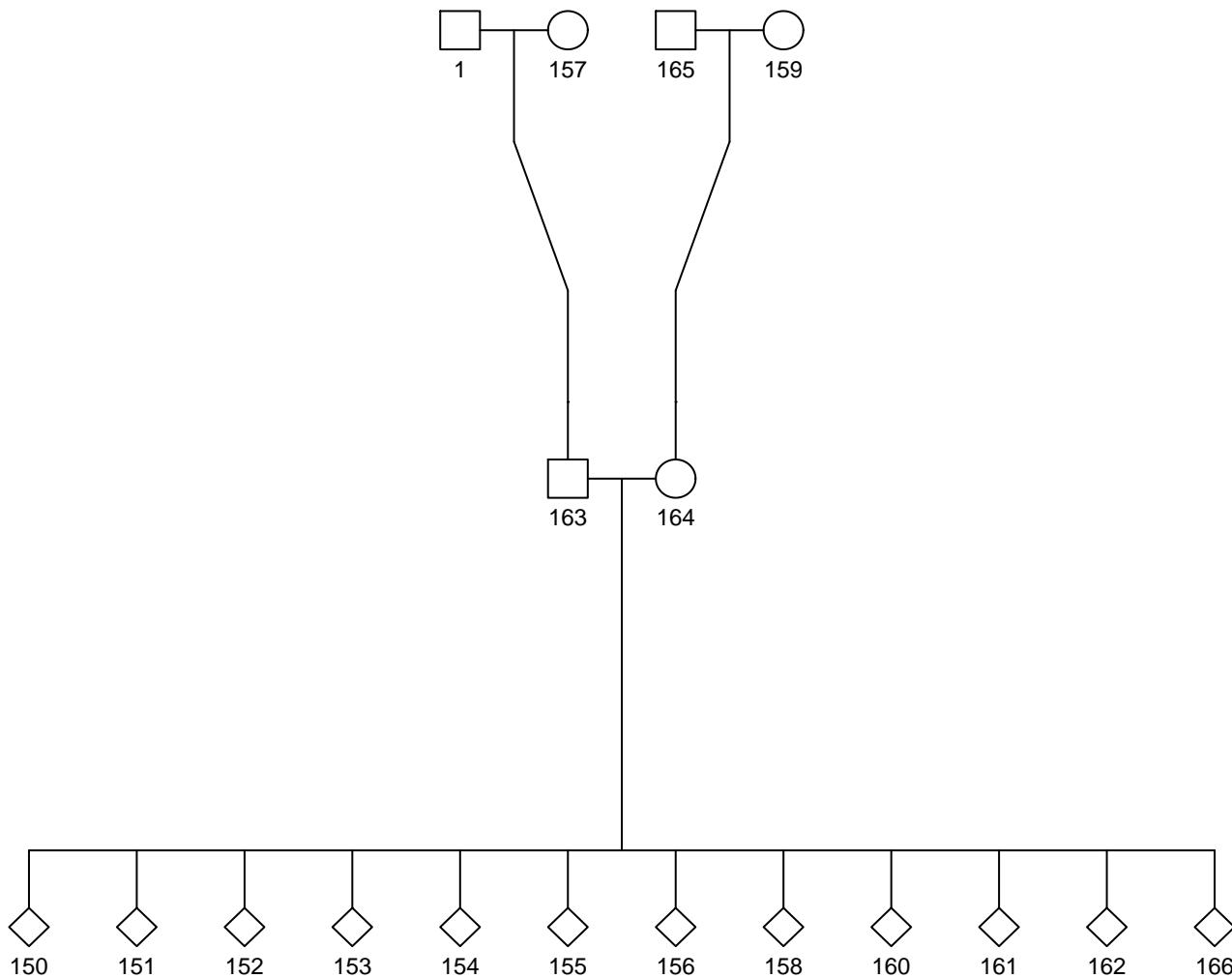

Supplement: Supplementary file 1. — All family and sample IDs have been anonymized, and the sexes of third-generation individuals have been hidden. [file elife-46922-supp1.zip › supp_file_1/10.pdf]

11

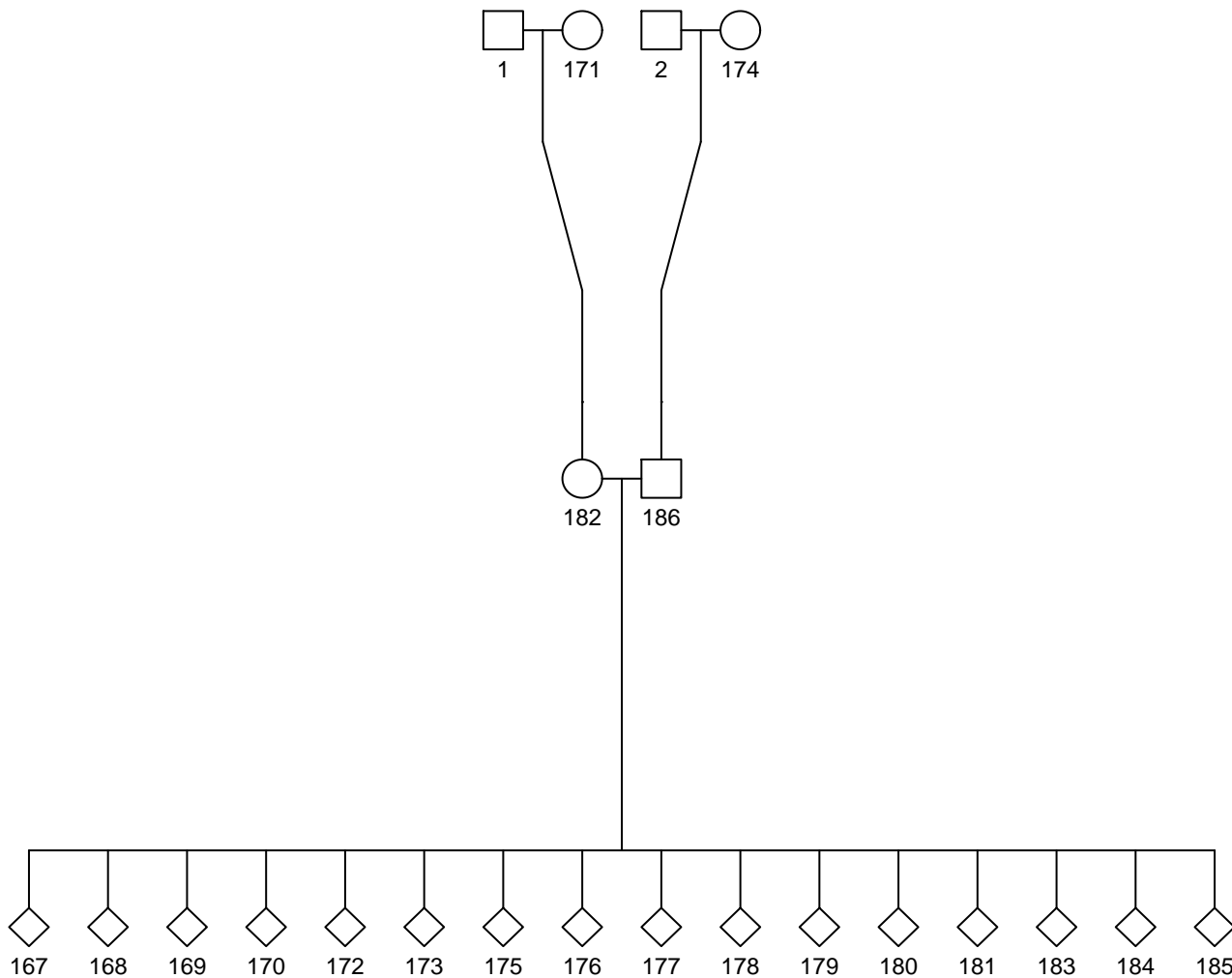

Supplement: Supplementary file 1. — All family and sample IDs have been anonymized, and the sexes of third-generation individuals have been hidden. [file elife-46922-supp1.zip › supp_file_1/11.pdf]

13

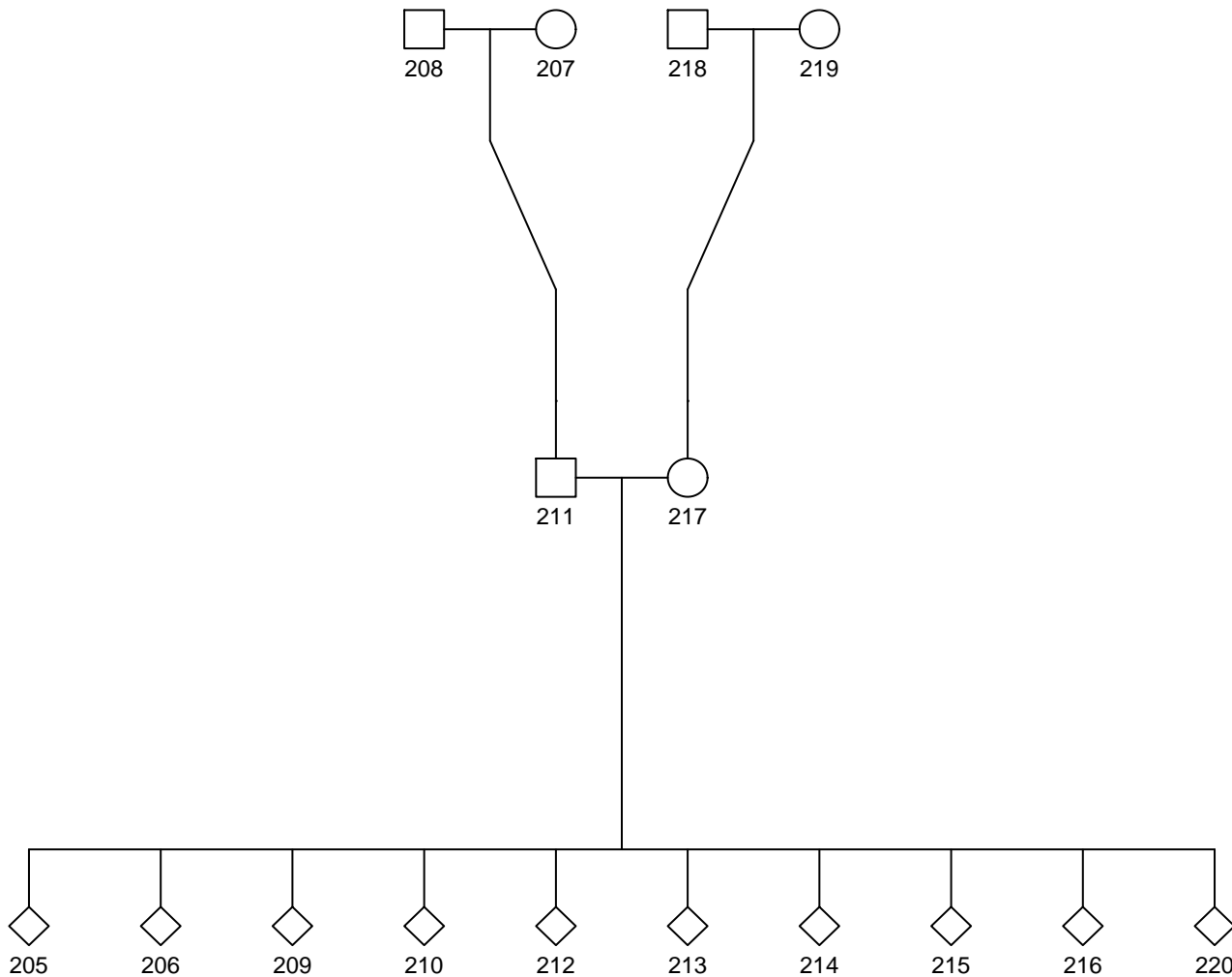

Supplement: Supplementary file 1. — All family and sample IDs have been anonymized, and the sexes of third-generation individuals have been hidden. [file elife-46922-supp1.zip › supp_file_1/13.pdf]

12

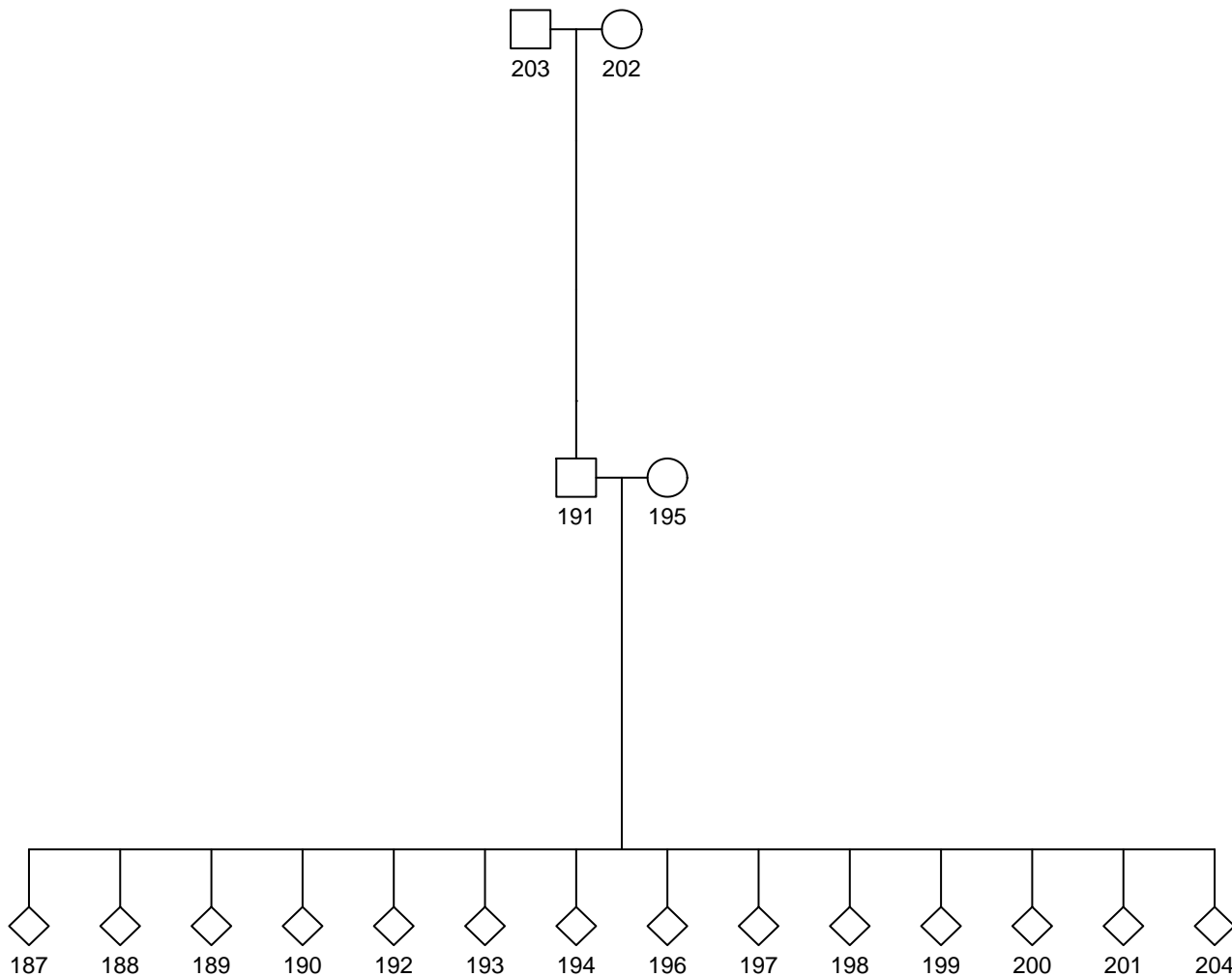

Supplement: Supplementary file 1. — All family and sample IDs have been anonymized, and the sexes of third-generation individuals have been hidden. [file elife-46922-supp1.zip › supp_file_1/12.pdf]

23

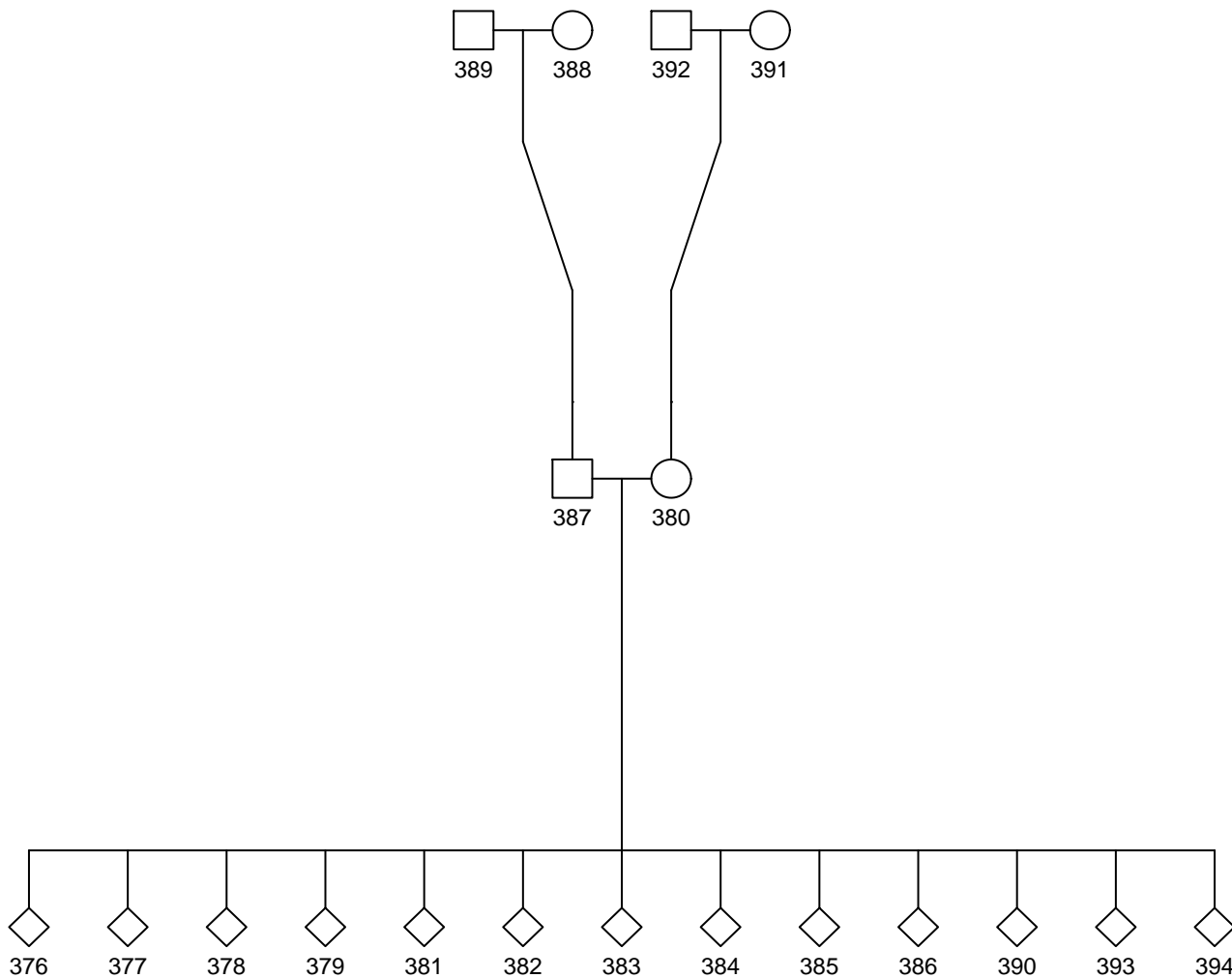

Supplement: Supplementary file 1. — All family and sample IDs have been anonymized, and the sexes of third-generation individuals have been hidden. [file elife-46922-supp1.zip › supp_file_1/23.pdf]

22

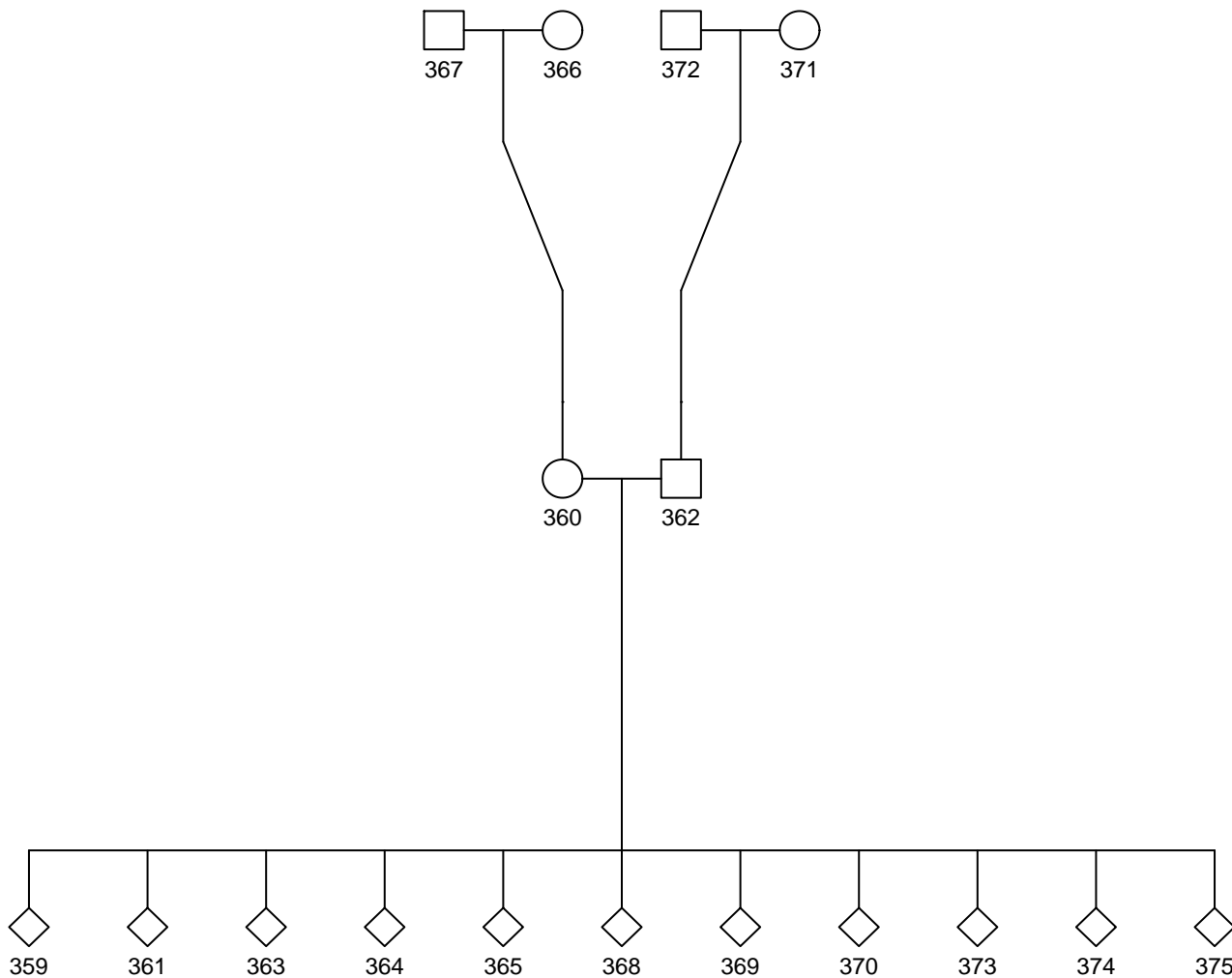

Supplement: Supplementary file 1. — All family and sample IDs have been anonymized, and the sexes of third-generation individuals have been hidden. [file elife-46922-supp1.zip › supp_file_1/22.pdf]

**20**

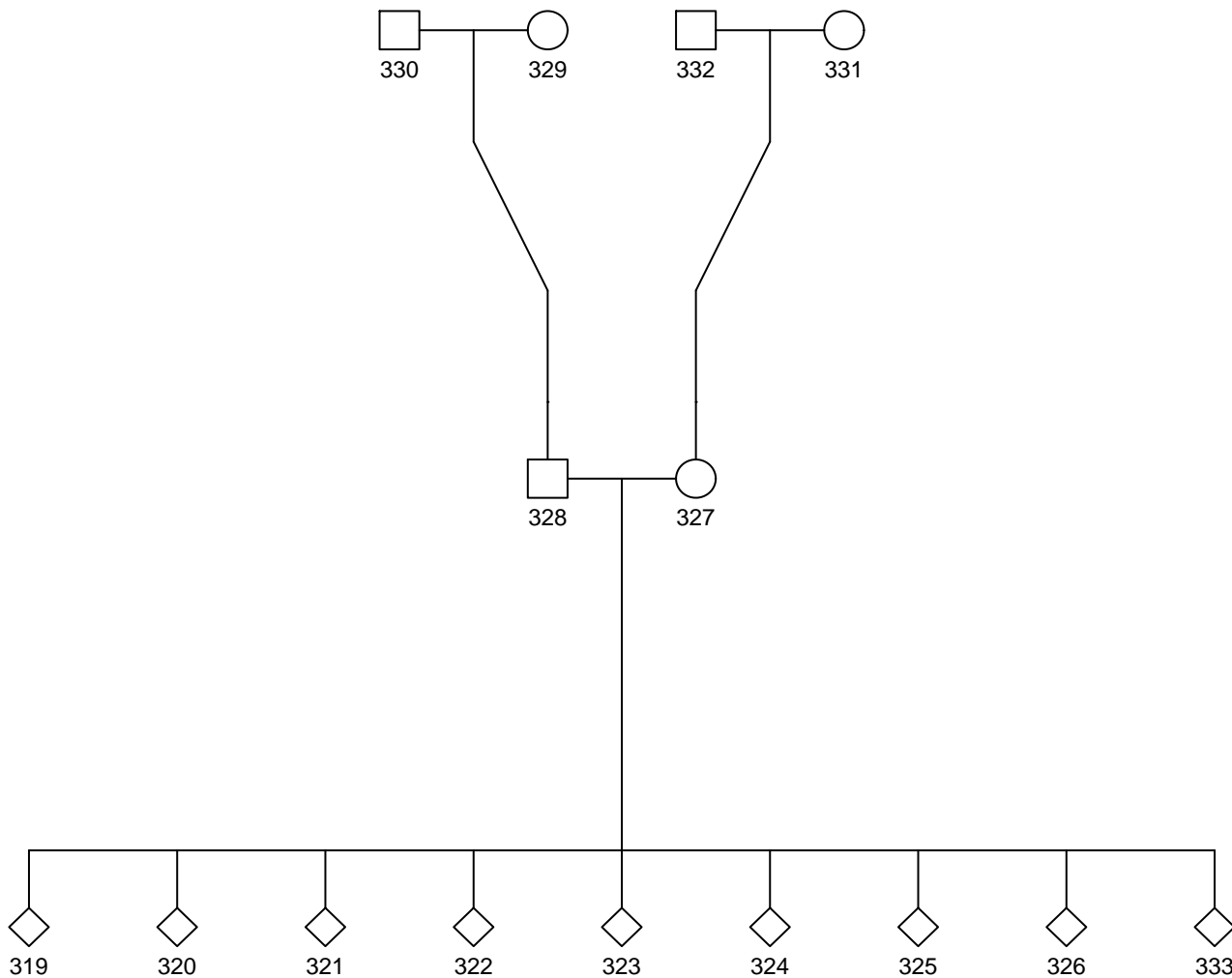

Supplement: Supplementary file 1. — All family and sample IDs have been anonymized, and the sexes of third-generation individuals have been hidden. [file elife-46922-supp1.zip › supp_file_1/20.pdf]

21

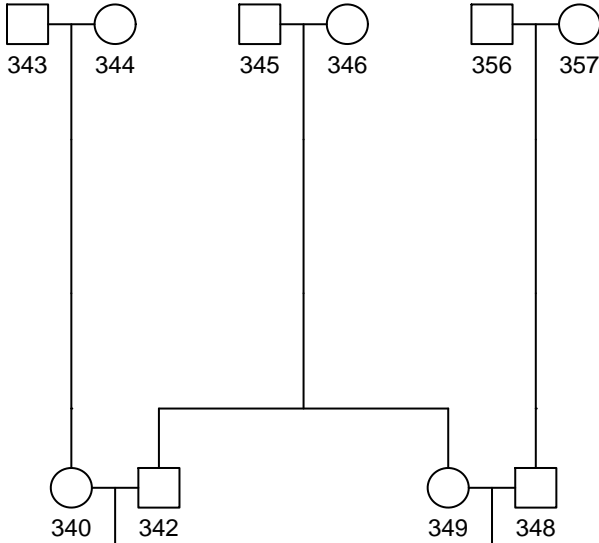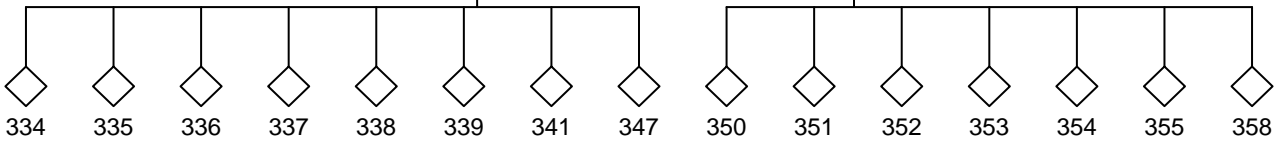

Supplement: Supplementary file 1. — All family and sample IDs have been anonymized, and the sexes of third-generation individuals have been hidden. [file elife-46922-supp1.zip › supp_file_1/21.pdf]

31

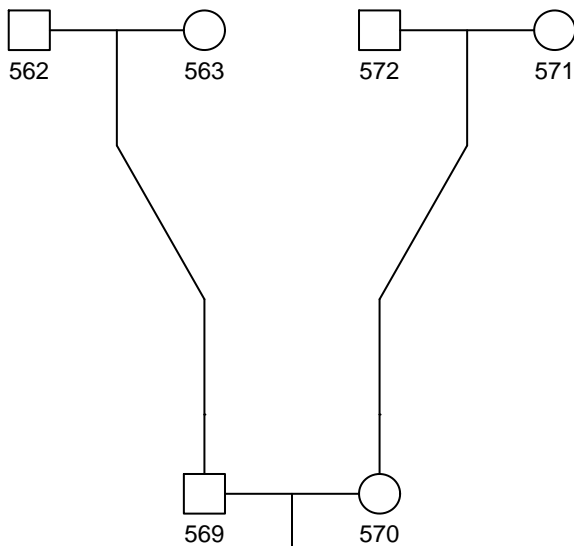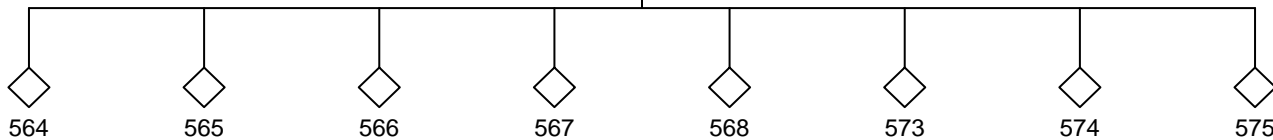

Supplement: Supplementary file 1. — All family and sample IDs have been anonymized, and the sexes of third-generation individuals have been hidden. [file elife-46922-supp1.zip › supp_file_1/31.pdf]

25

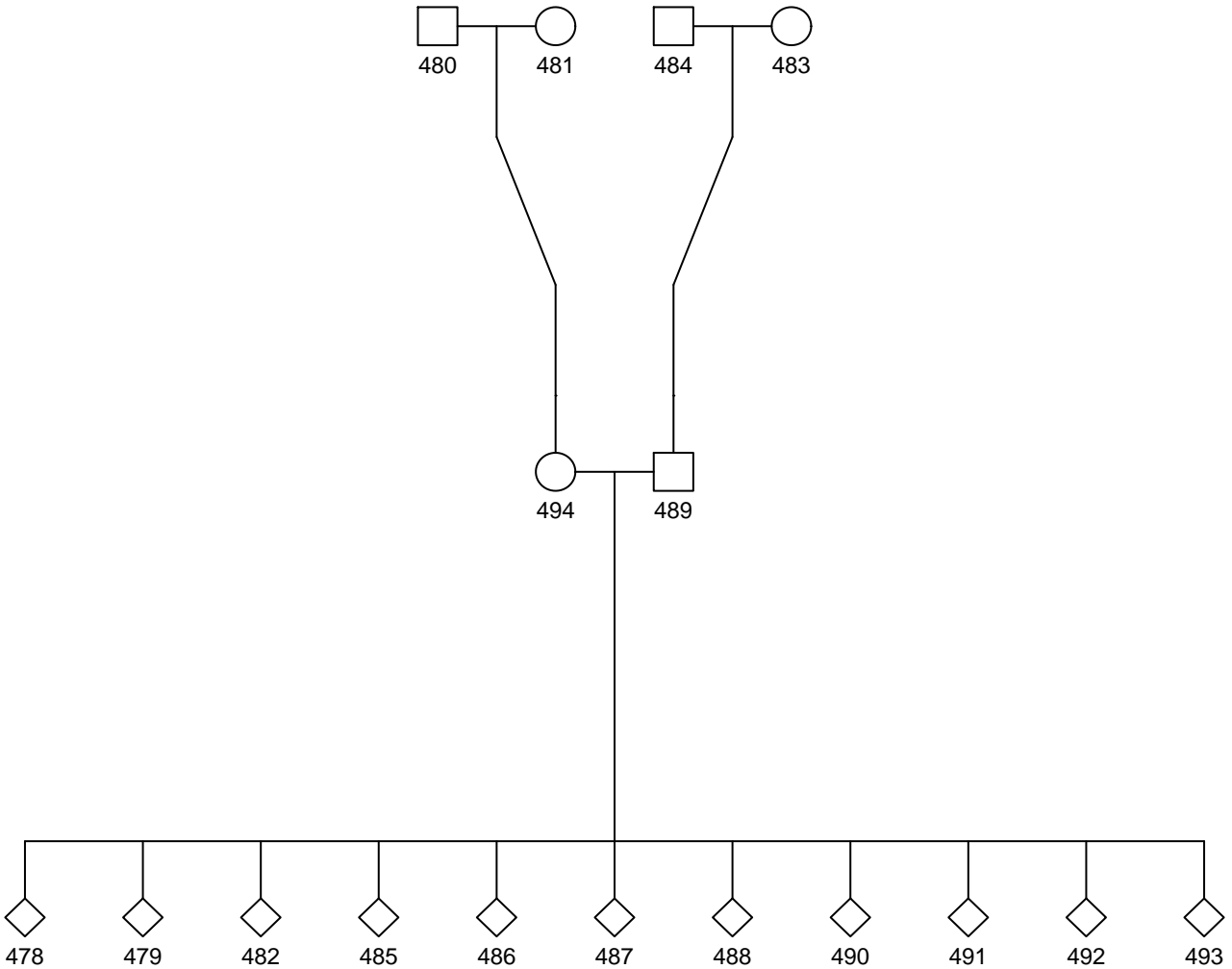

Supplement: Supplementary file 1. — All family and sample IDs have been anonymized, and the sexes of third-generation individuals have been hidden. [file elife-46922-supp1.zip › supp_file_1/25.pdf]

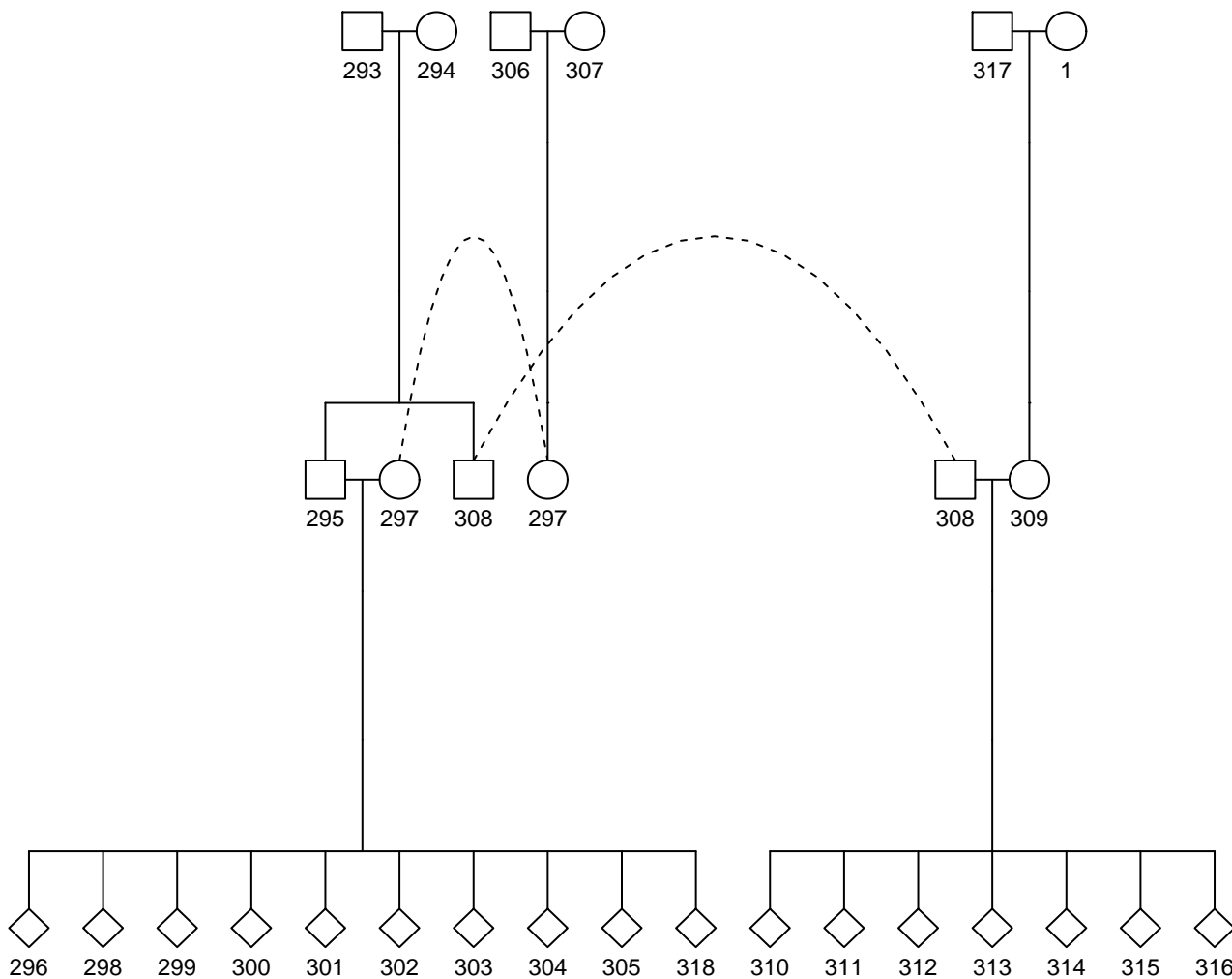

Supplement: Supplementary file 1. — All family and sample IDs have been anonymized, and the sexes of third-generation individuals have been hidden. [file elife-46922-supp1.zip › supp_file_1/19.pdf]

18

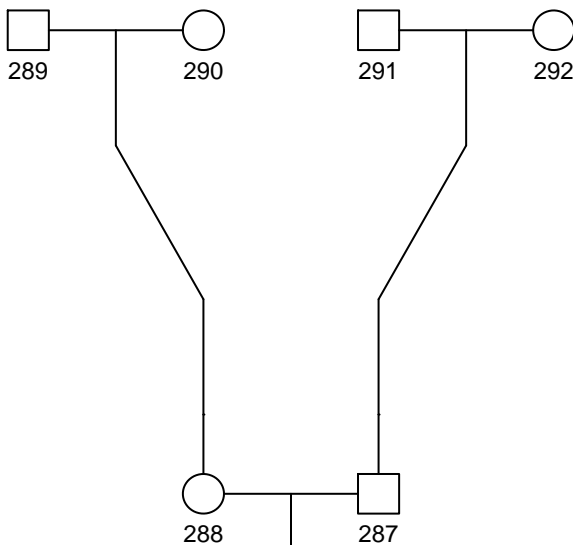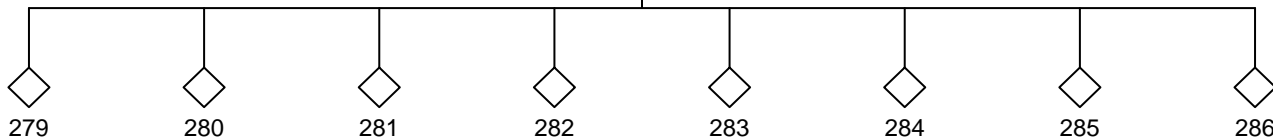

Supplement: Supplementary file 1. — All family and sample IDs have been anonymized, and the sexes of third-generation individuals have been hidden. [file elife-46922-supp1.zip › supp_file_1/18.pdf]

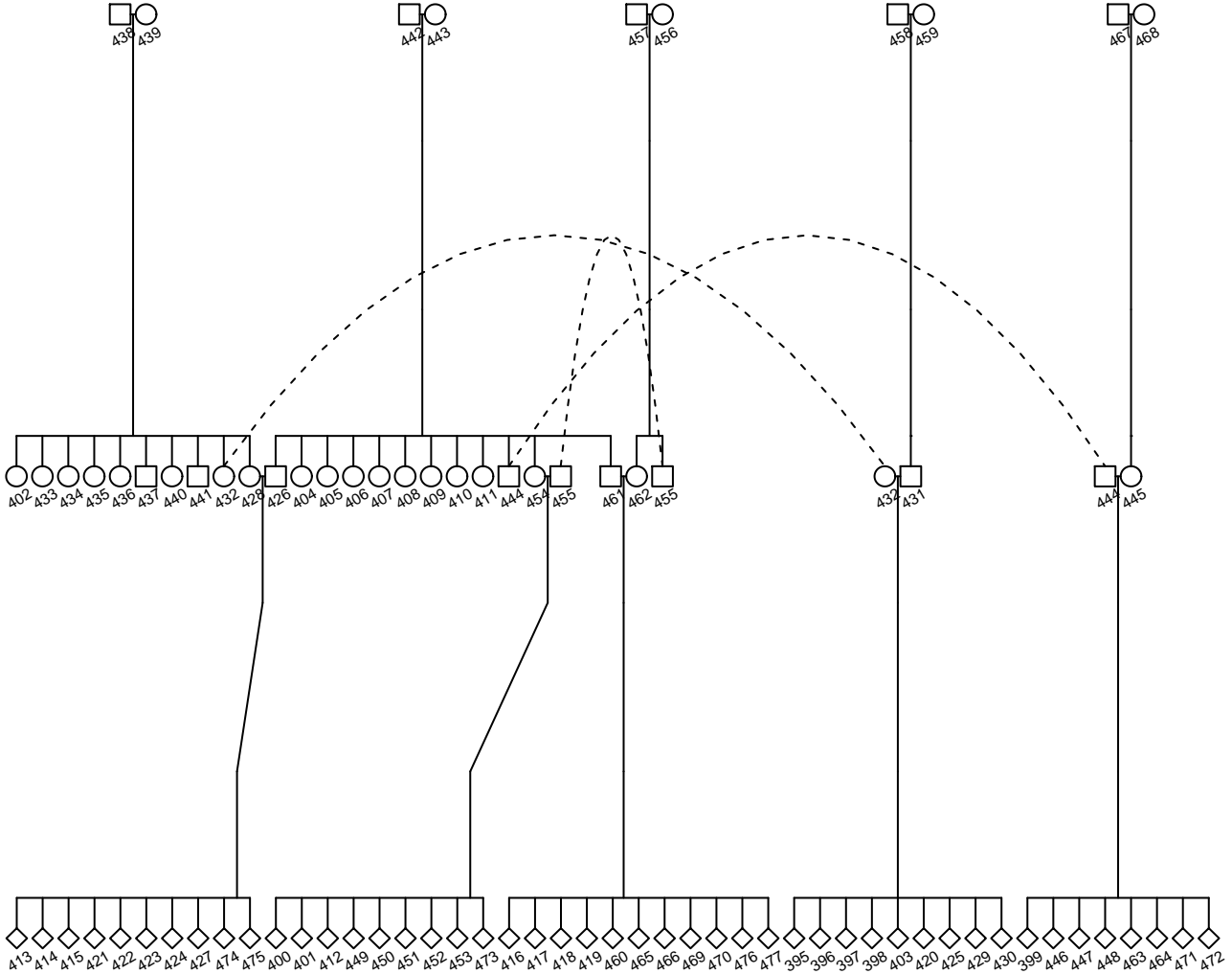

Supplement: Supplementary file 1. — All family and sample IDs have been anonymized, and the sexes of third-generation individuals have been hidden. [file elife-46922-supp1.zip › supp_file_1/24.pdf]

**30**

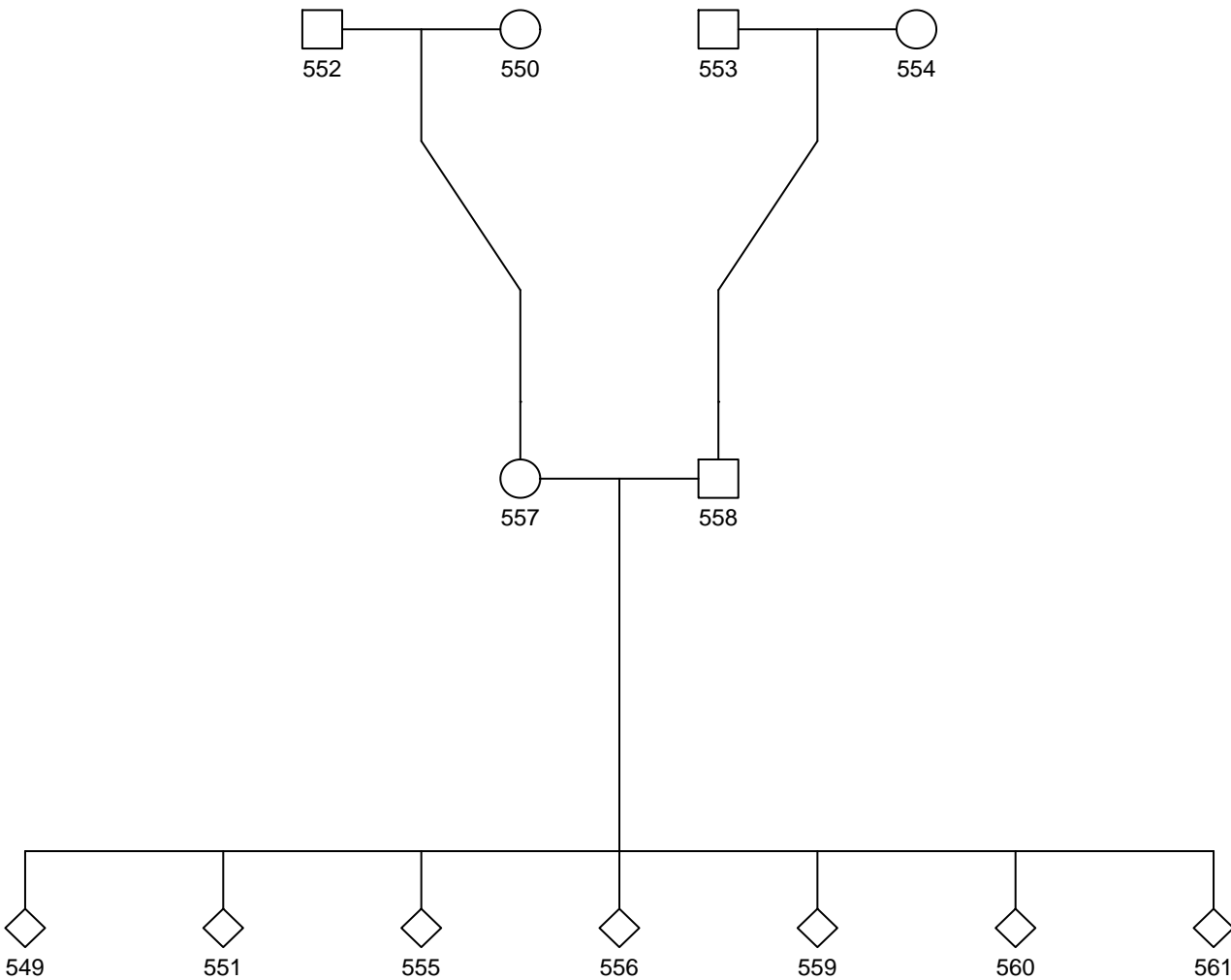

Supplement: Supplementary file 1. — All family and sample IDs have been anonymized, and the sexes of third-generation individuals have been hidden. [file elife-46922-supp1.zip › supp_file_1/30.pdf]

26

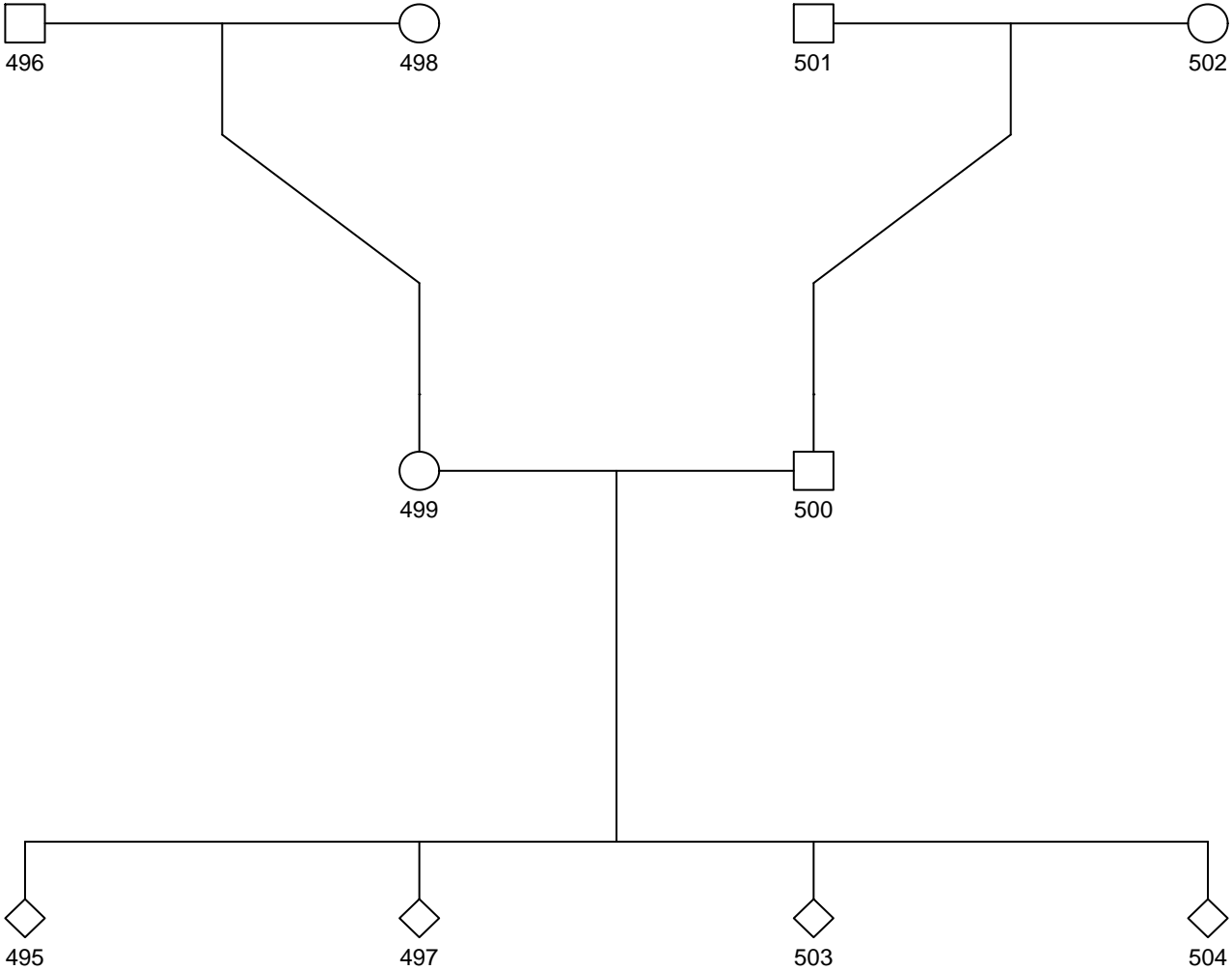

Supplement: Supplementary file 1. — All family and sample IDs have been anonymized, and the sexes of third-generation individuals have been hidden. [file elife-46922-supp1.zip › supp_file_1/26.pdf]

32

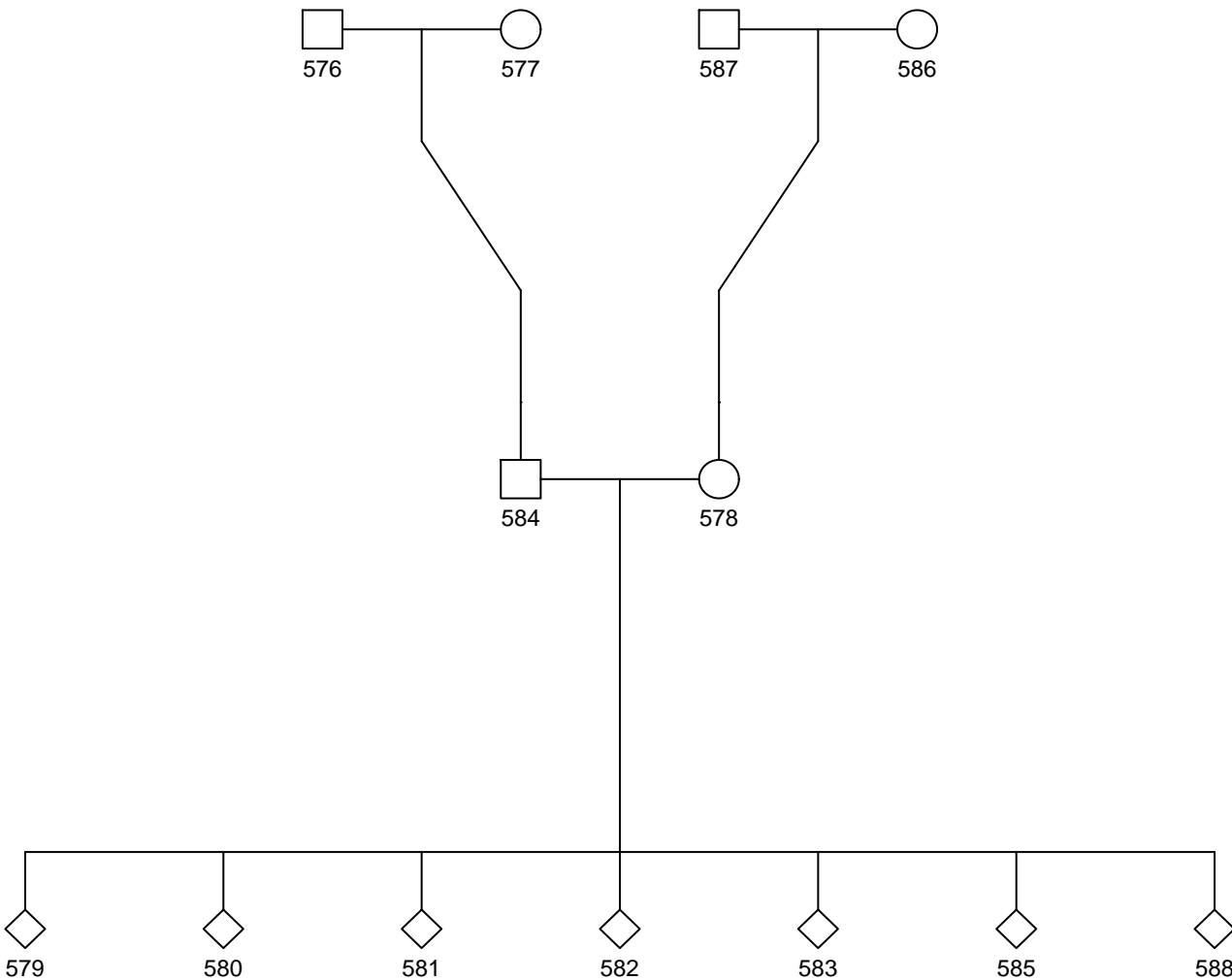

Supplement: Supplementary file 1. — All family and sample IDs have been anonymized, and the sexes of third-generation individuals have been hidden. [file elife-46922-supp1.zip › supp_file_1/32.pdf]

**33**

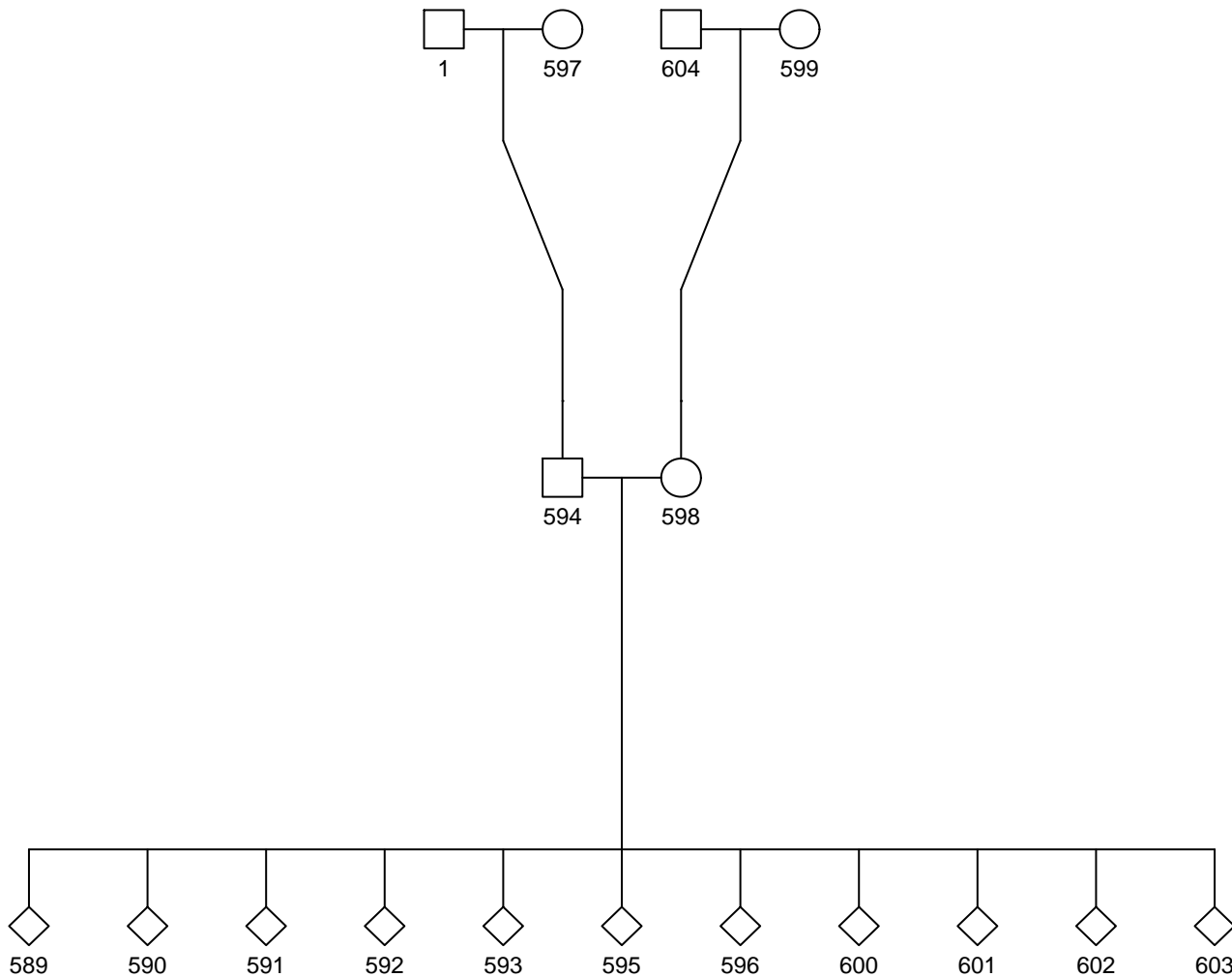

Supplement: Supplementary file 1. — All family and sample IDs have been anonymized, and the sexes of third-generation individuals have been hidden. [file elife-46922-supp1.zip › supp_file_1/33.pdf]

**27**

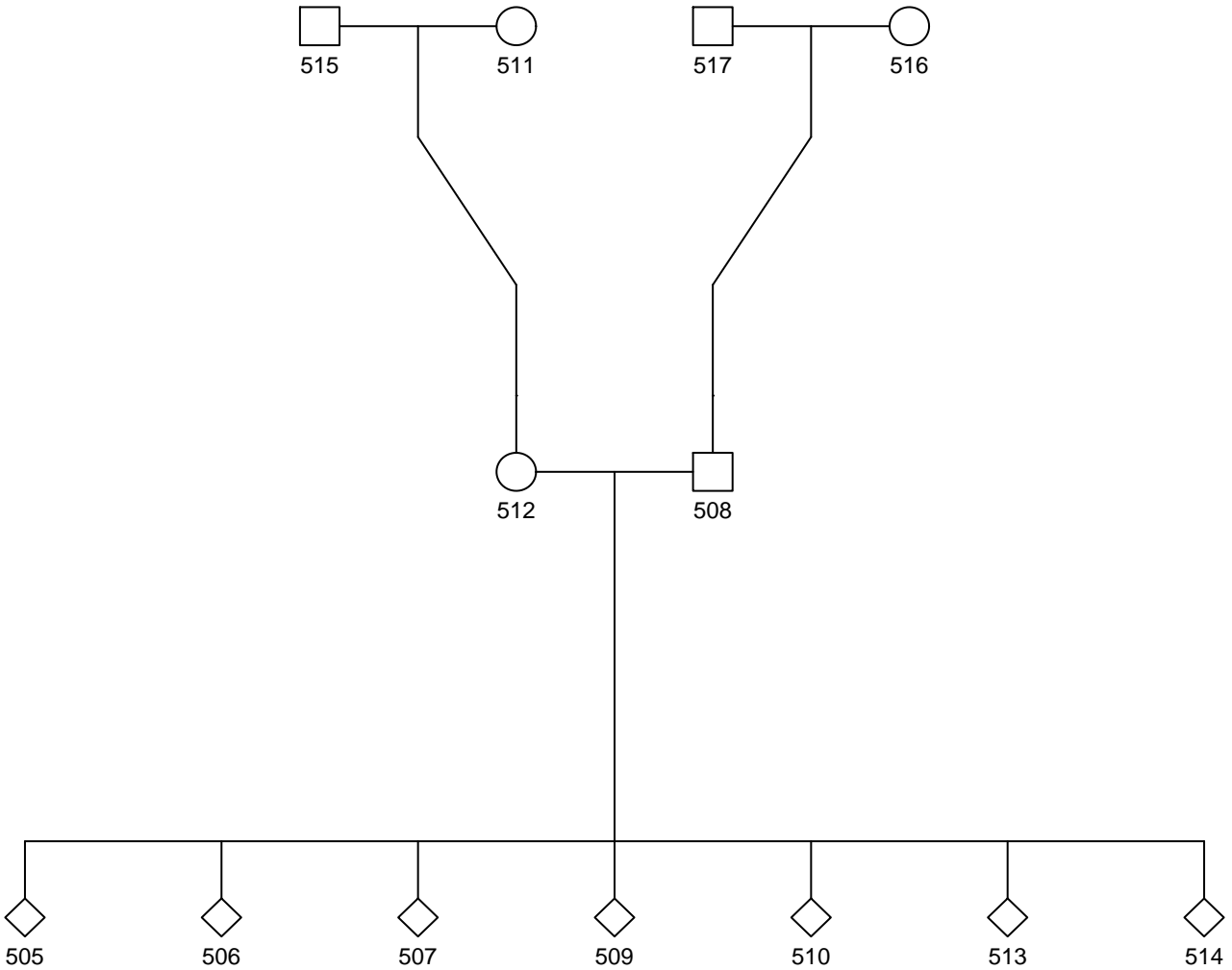

Supplement: Supplementary file 1. — All family and sample IDs have been anonymized, and the sexes of third-generation individuals have been hidden. [file elife-46922-supp1.zip › supp_file_1/27.pdf]

6

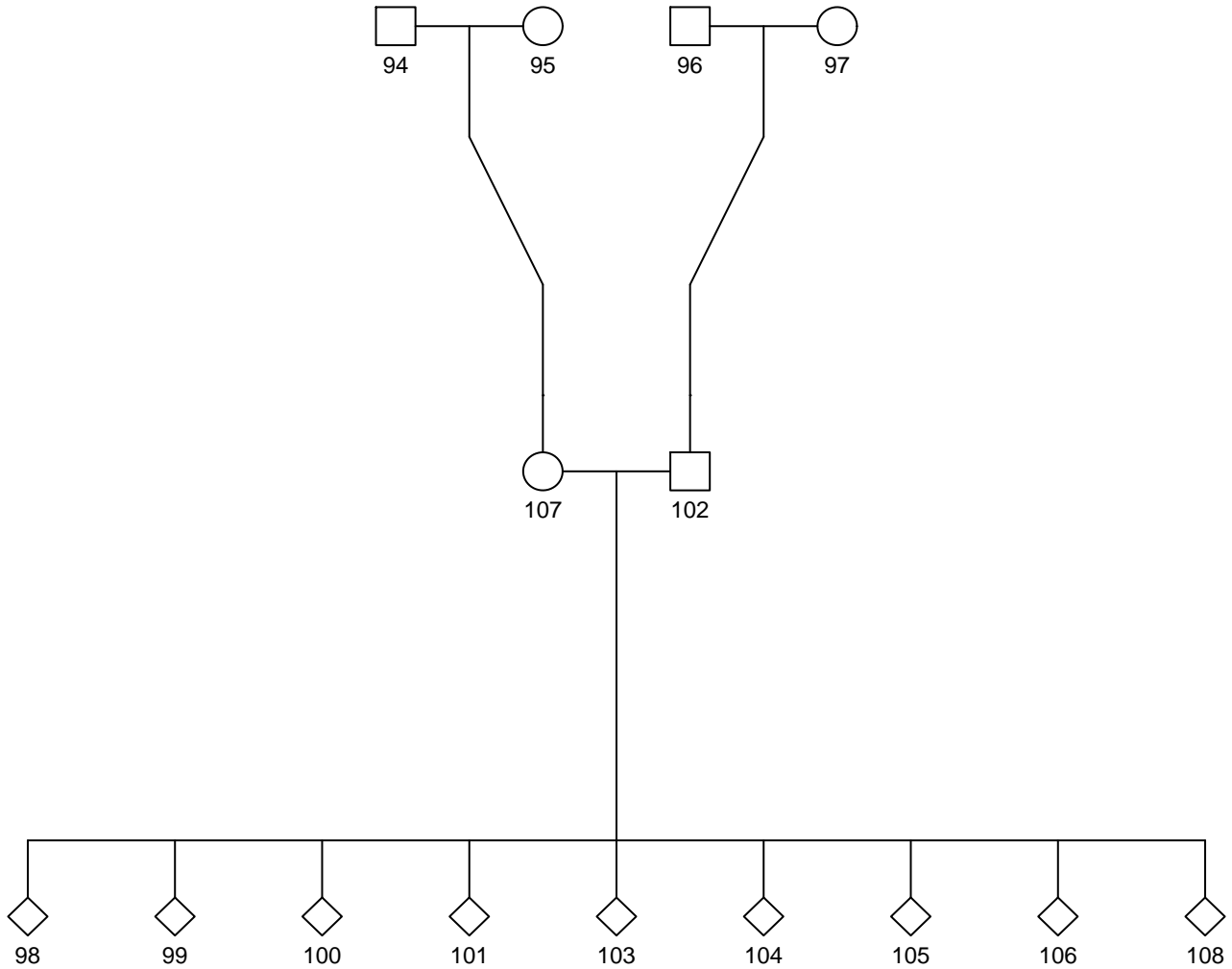

Supplement: Supplementary file 1. — All family and sample IDs have been anonymized, and the sexes of third-generation individuals have been hidden. [file elife-46922-supp1.zip › supp_file_1/6.pdf]

**7**

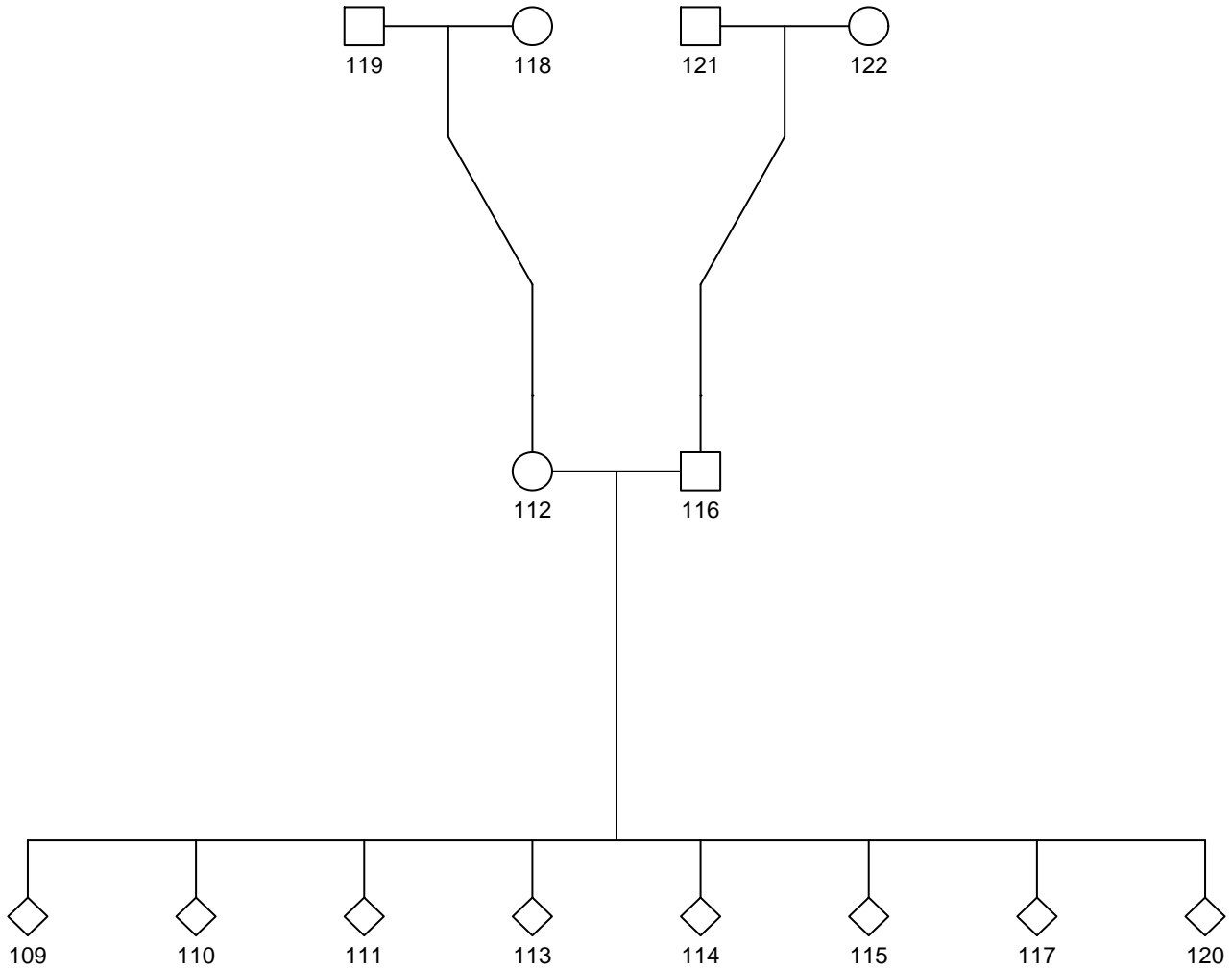

Supplement: Supplementary file 1. — All family and sample IDs have been anonymized, and the sexes of third-generation individuals have been hidden. [file elife-46922-supp1.zip › supp_file_1/7.pdf]

**5**

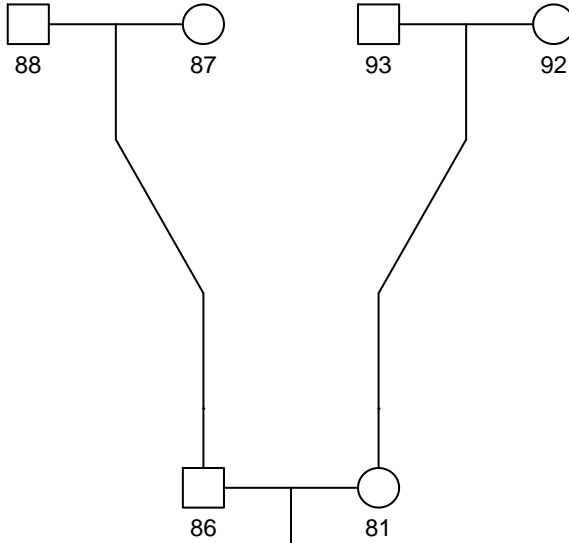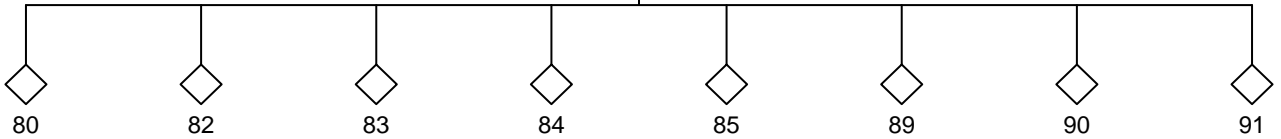

Supplement: Supplementary file 1. — All family and sample IDs have been anonymized, and the sexes of third-generation individuals have been hidden. [file elife-46922-supp1.zip › supp_file_1/5.pdf]

4

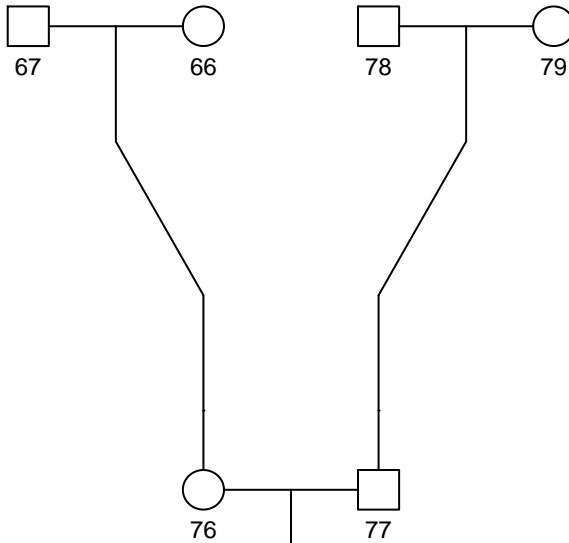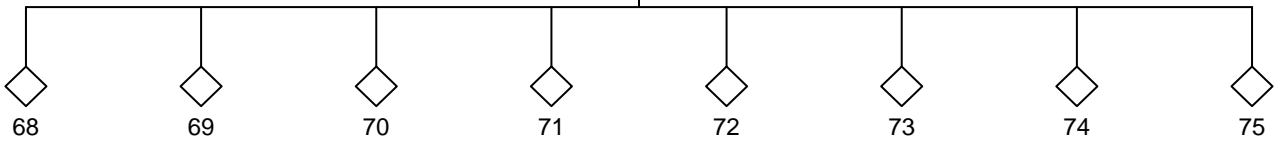

Supplement: Supplementary file 1. — All family and sample IDs have been anonymized, and the sexes of third-generation individuals have been hidden. [file elife-46922-supp1.zip › supp_file_1/4.pdf]

1

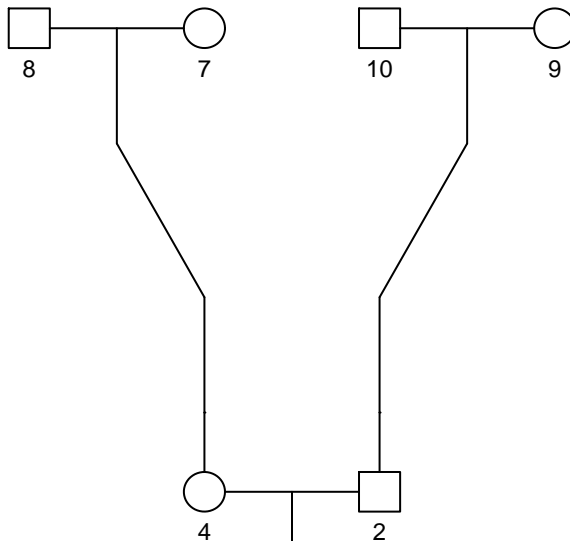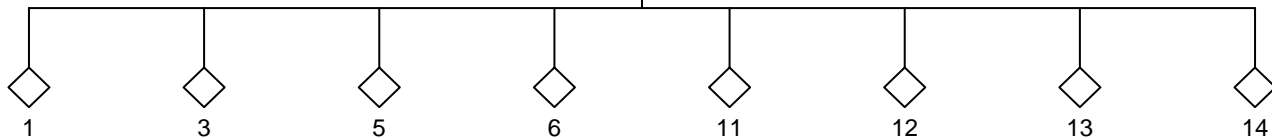

Supplement: Supplementary file 1. — All family and sample IDs have been anonymized, and the sexes of third-generation individuals have been hidden. [file elife-46922-supp1.zip › supp_file_1/1.pdf]

**3**

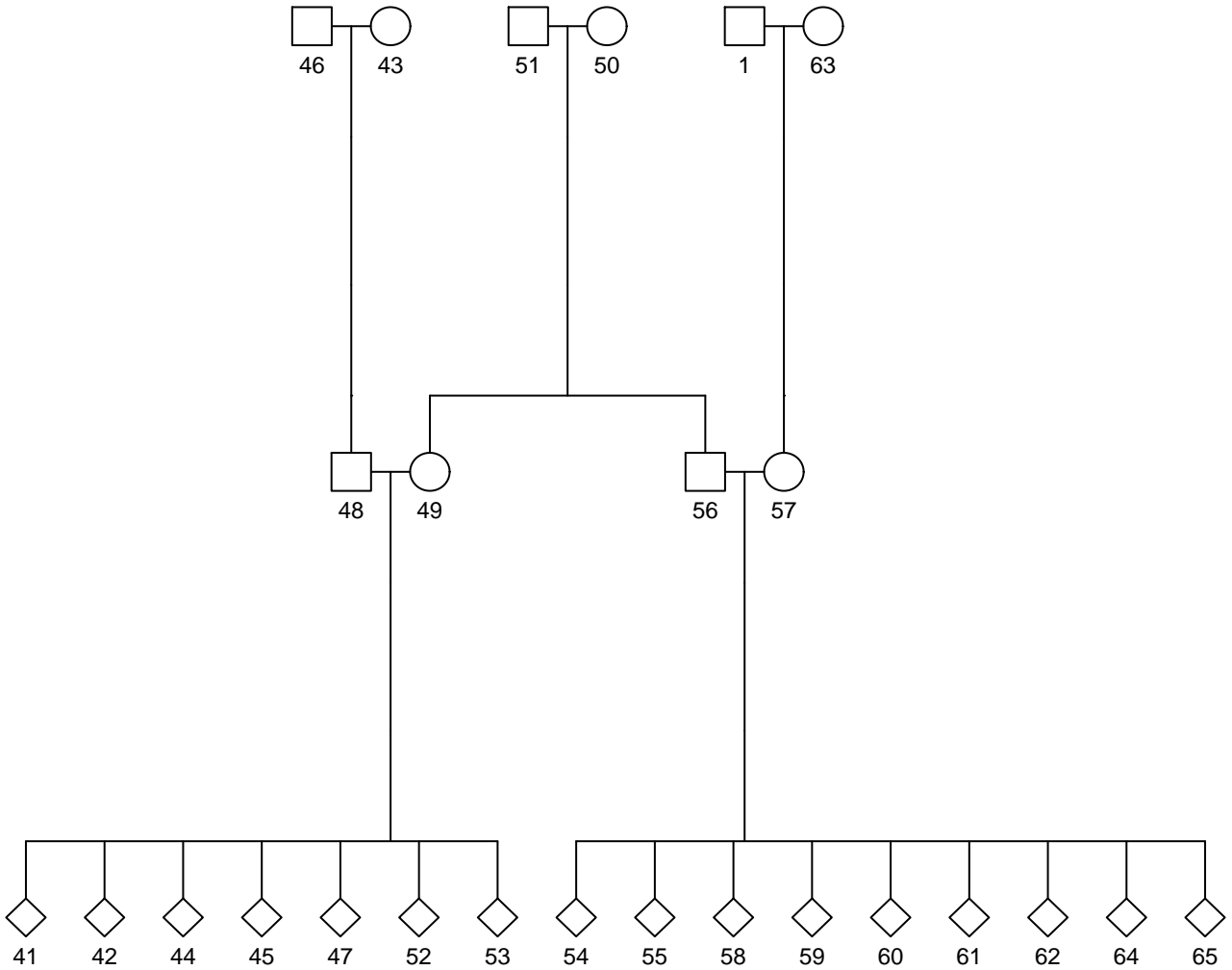

Supplement: Supplementary file 1. — All family and sample IDs have been anonymized, and the sexes of third-generation individuals have been hidden. [file elife-46922-supp1.zip › supp_file_1/3.pdf]

2

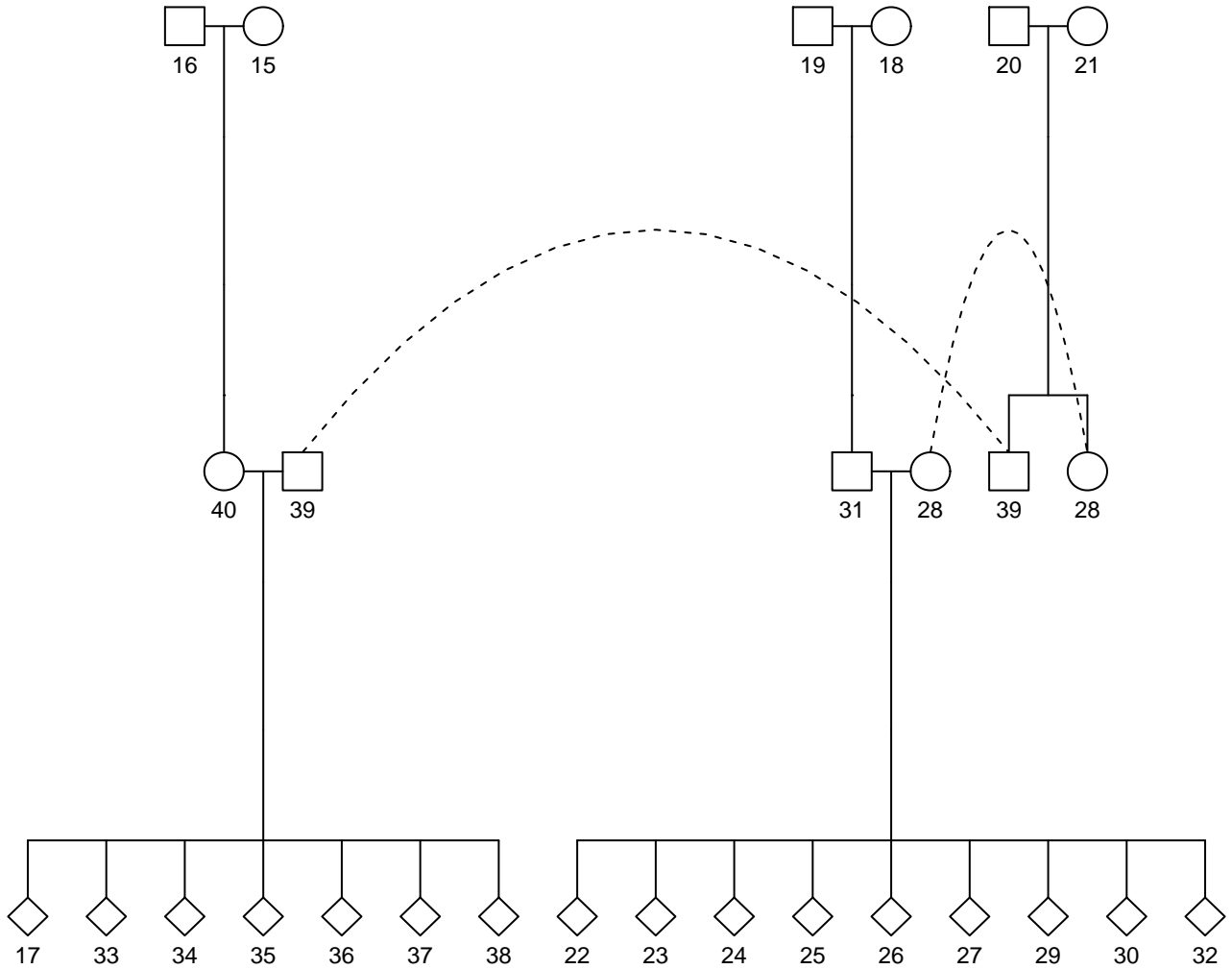

Supplement: Supplementary file 1. — All family and sample IDs have been anonymized, and the sexes of third-generation individuals have been hidden. [file elife-46922-supp1.zip › supp_file_1/2.pdf]

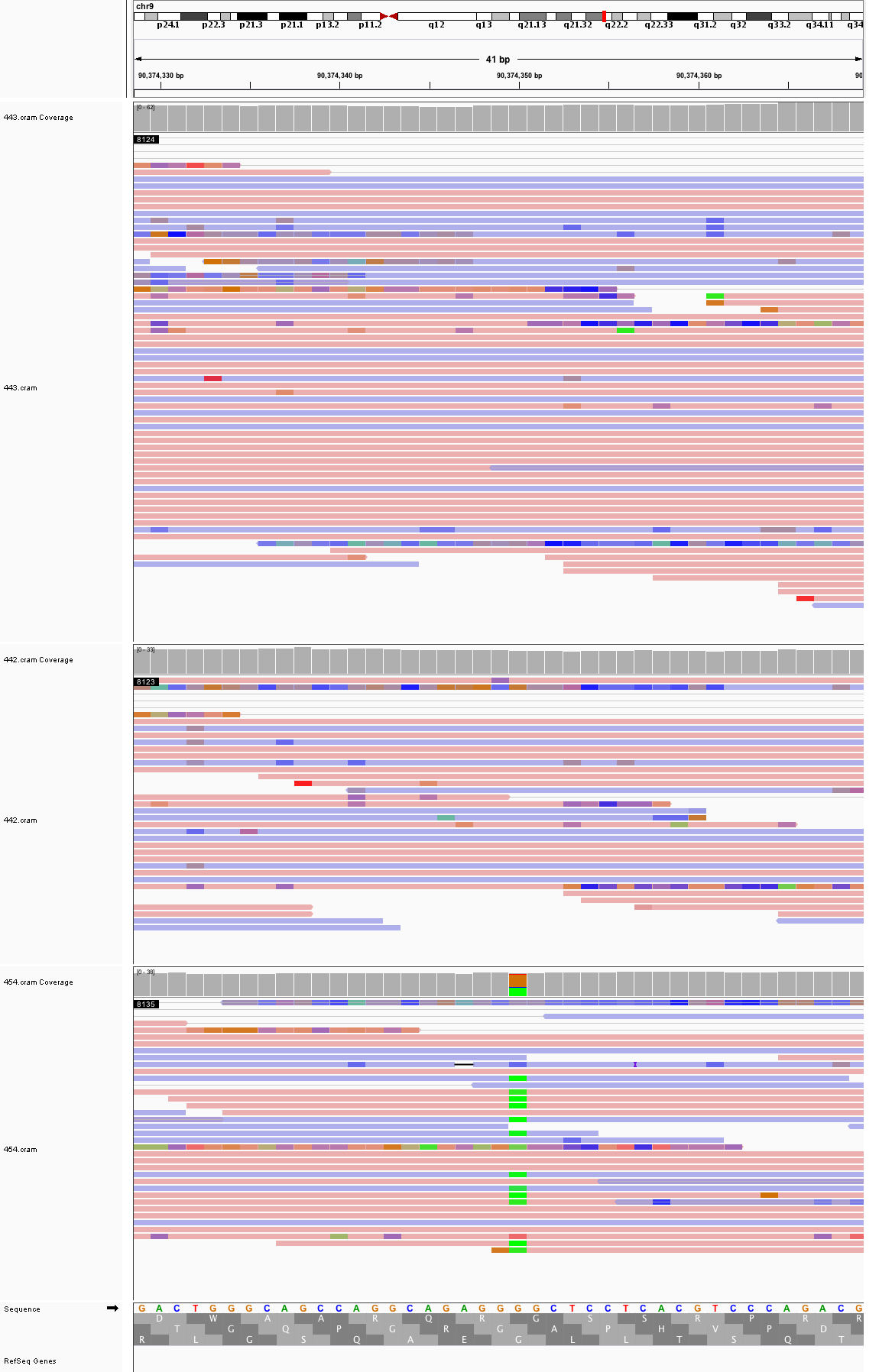

Supplement: Supplementary file 2. — In each image, the first two tracks contain alignments from the first-generation parents, and the third track contains the alignments for the second-generation child. Reads with mapping quality <20 are not included, as they were not considered by our variant calling pipeline, and mismatched bases are shaded by quality score (more transparent = lower base quality). [file elife-46922-supp2.zip › supp_file_2/chr9_90,374,329_90,374,369.png]

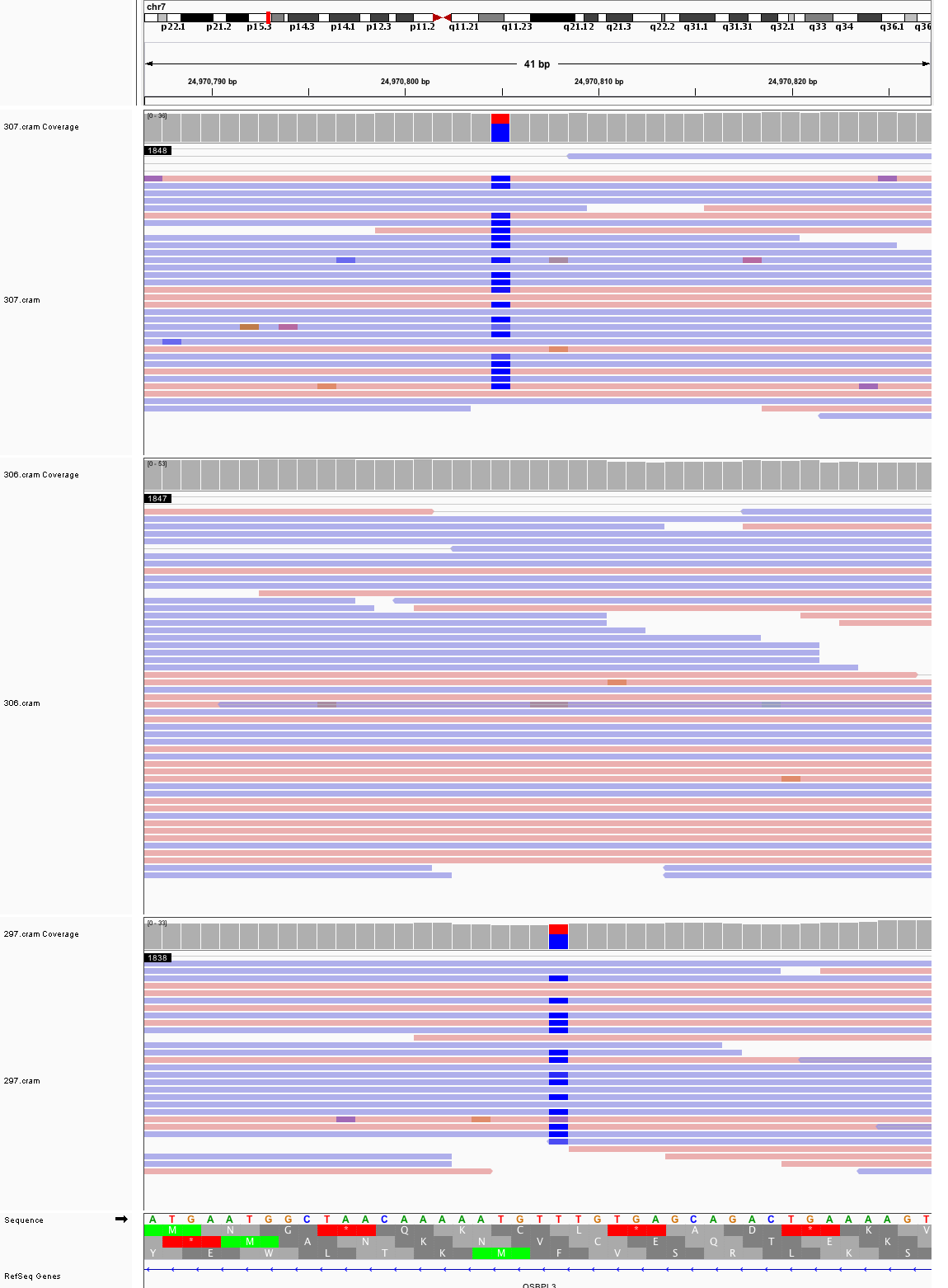

Supplement: Supplementary file 2. — In each image, the first two tracks contain alignments from the first-generation parents, and the third track contains the alignments for the second-generation child. Reads with mapping quality <20 are not included, as they were not considered by our variant calling pipeline, and mismatched bases are shaded by quality score (more transparent = lower base quality). [file elife-46922-supp2.zip › supp_file_2/chr7_24,970,787_24,970,827.png]

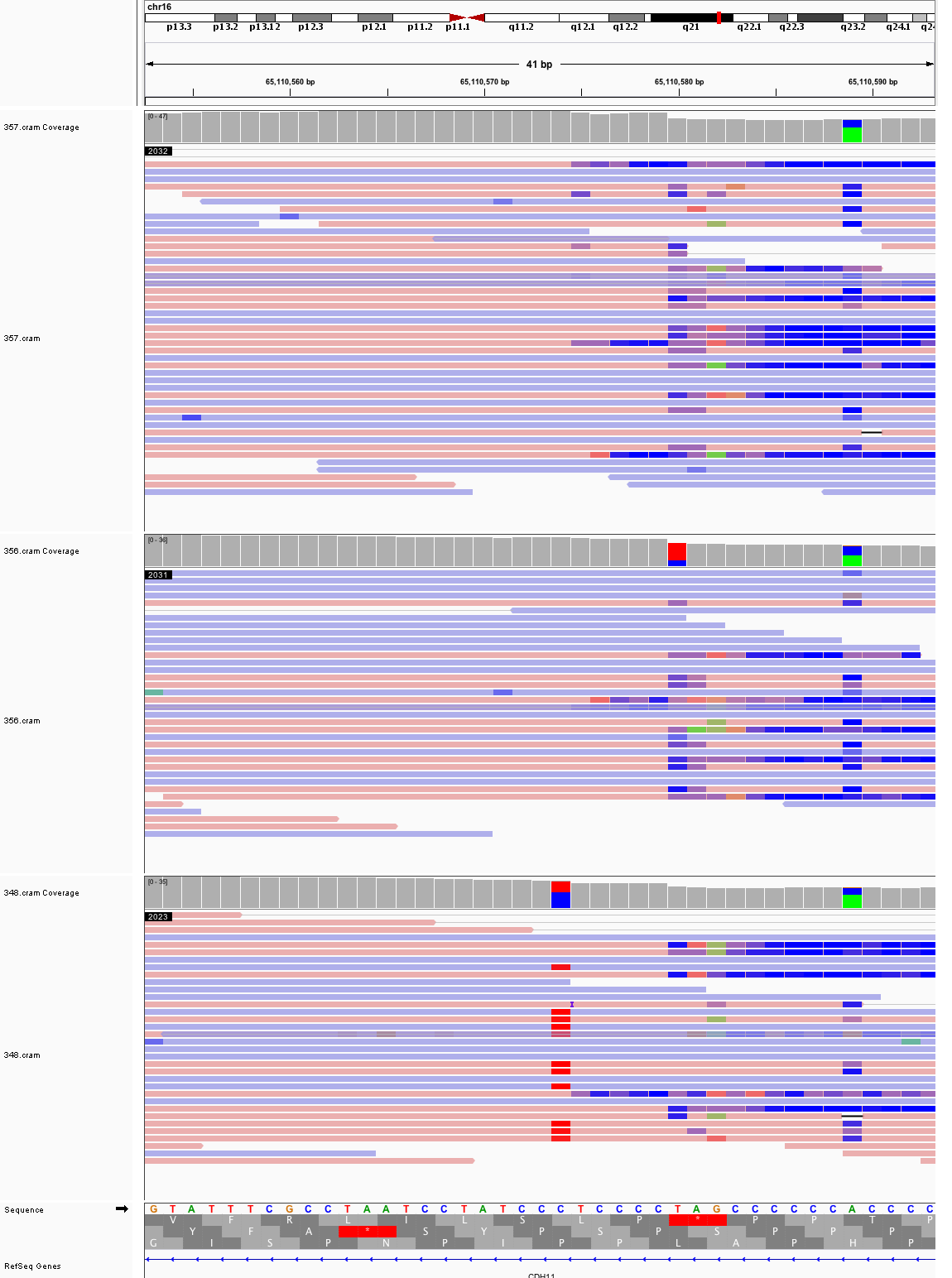

Supplement: Supplementary file 2. — In each image, the first two tracks contain alignments from the first-generation parents, and the third track contains the alignments for the second-generation child. Reads with mapping quality <20 are not included, as they were not considered by our variant calling pipeline, and mismatched bases are shaded by quality score (more transparent = lower base quality). [file elife-46922-supp2.zip › supp_file_2/chr16_65,110,553_65,110,593.png]

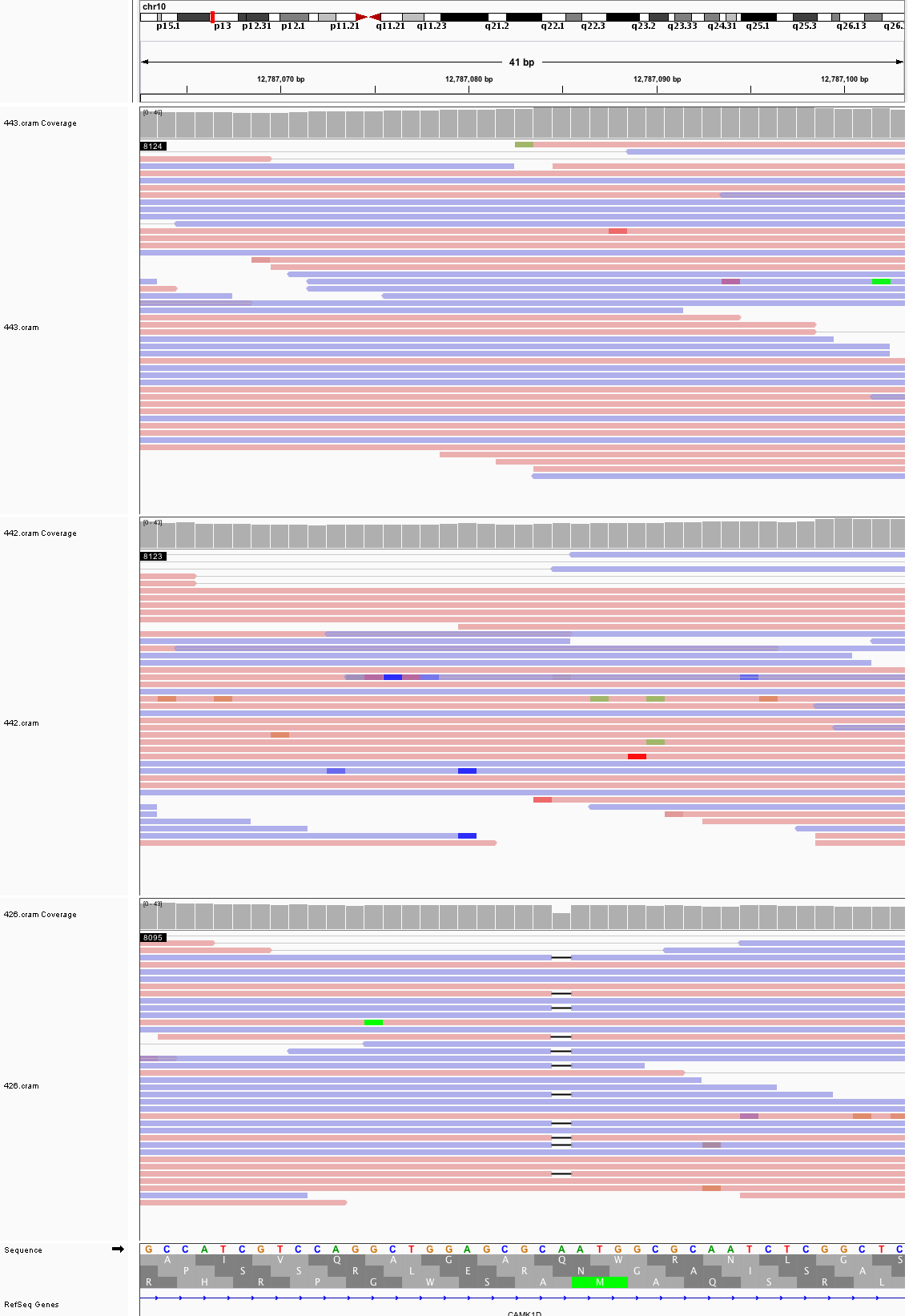

Supplement: Supplementary file 2. — In each image, the first two tracks contain alignments from the first-generation parents, and the third track contains the alignments for the second-generation child. Reads with mapping quality <20 are not included, as they were not considered by our variant calling pipeline, and mismatched bases are shaded by quality score (more transparent = lower base quality). [file elife-46922-supp2.zip › supp_file_2/chr10_12,787,063_12,787,103.png]

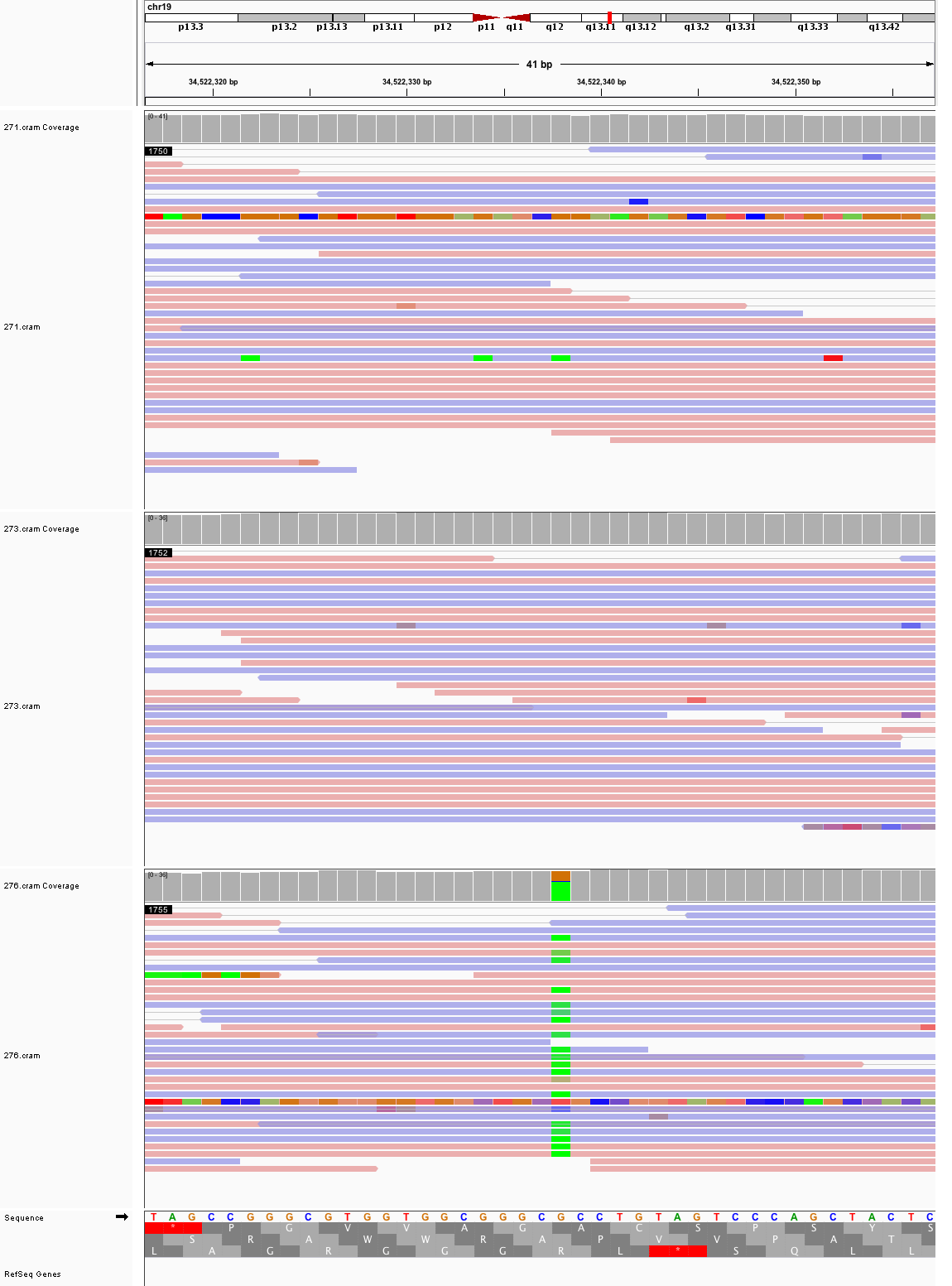

Supplement: Supplementary file 2. — In each image, the first two tracks contain alignments from the first-generation parents, and the third track contains the alignments for the second-generation child. Reads with mapping quality <20 are not included, as they were not considered by our variant calling pipeline, and mismatched bases are shaded by quality score (more transparent = lower base quality). [file elife-46922-supp2.zip › supp_file_2/chr19_34,522,317_34,522,357.png]

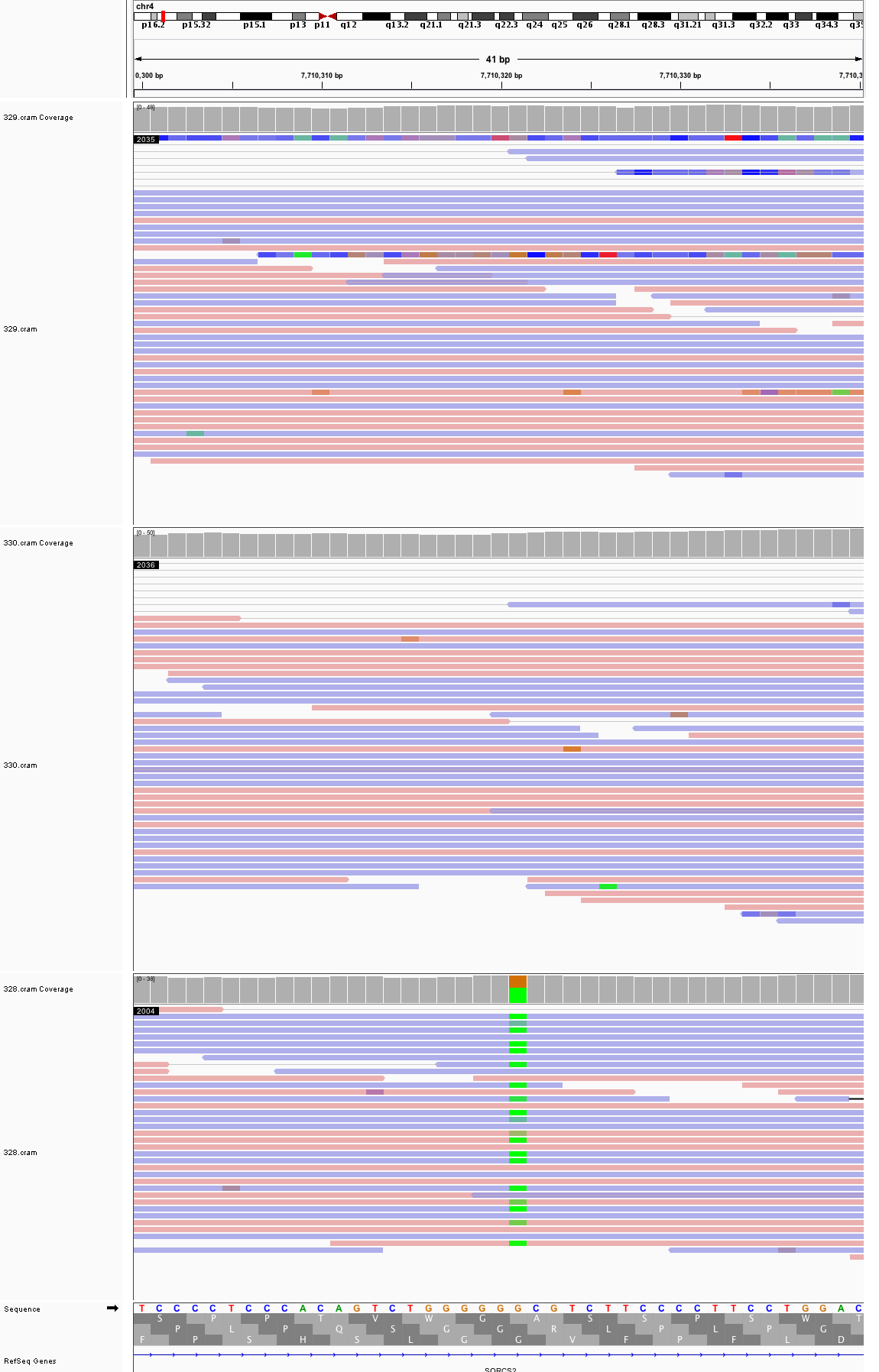

Supplement: Supplementary file 2. — In each image, the first two tracks contain alignments from the first-generation parents, and the third track contains the alignments for the second-generation child. Reads with mapping quality <20 are not included, as they were not considered by our variant calling pipeline, and mismatched bases are shaded by quality score (more transparent = lower base quality). [file elife-46922-supp2.zip › supp_file_2/chr4_7,710,300_7,710,340.png]

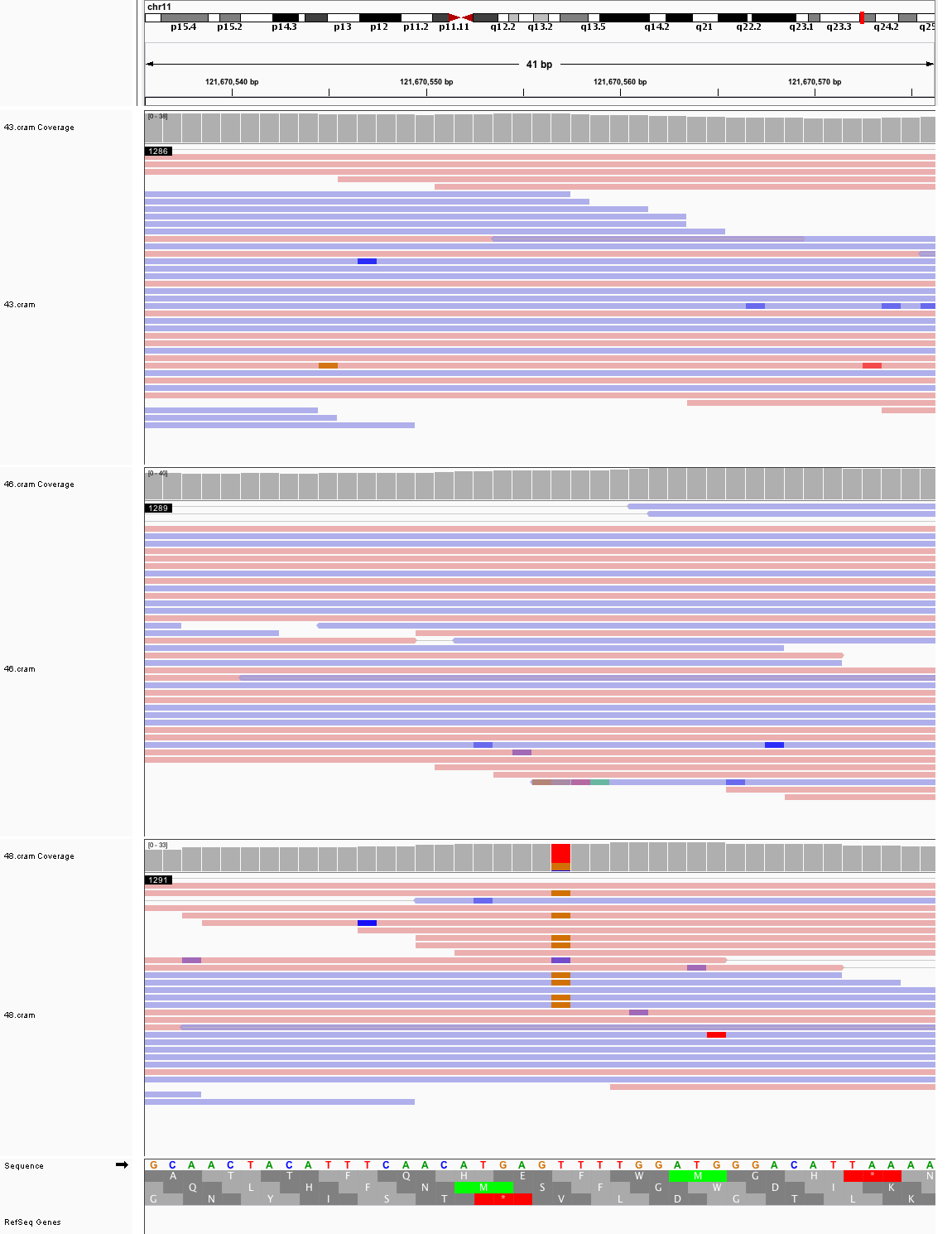

Supplement: Supplementary file 2. — In each image, the first two tracks contain alignments from the first-generation parents, and the third track contains the alignments for the second-generation child. Reads with mapping quality <20 are not included, as they were not considered by our variant calling pipeline, and mismatched bases are shaded by quality score (more transparent = lower base quality). [file elife-46922-supp2.zip › supp_file_2/chr11_121,670,536_121,670,576.png]

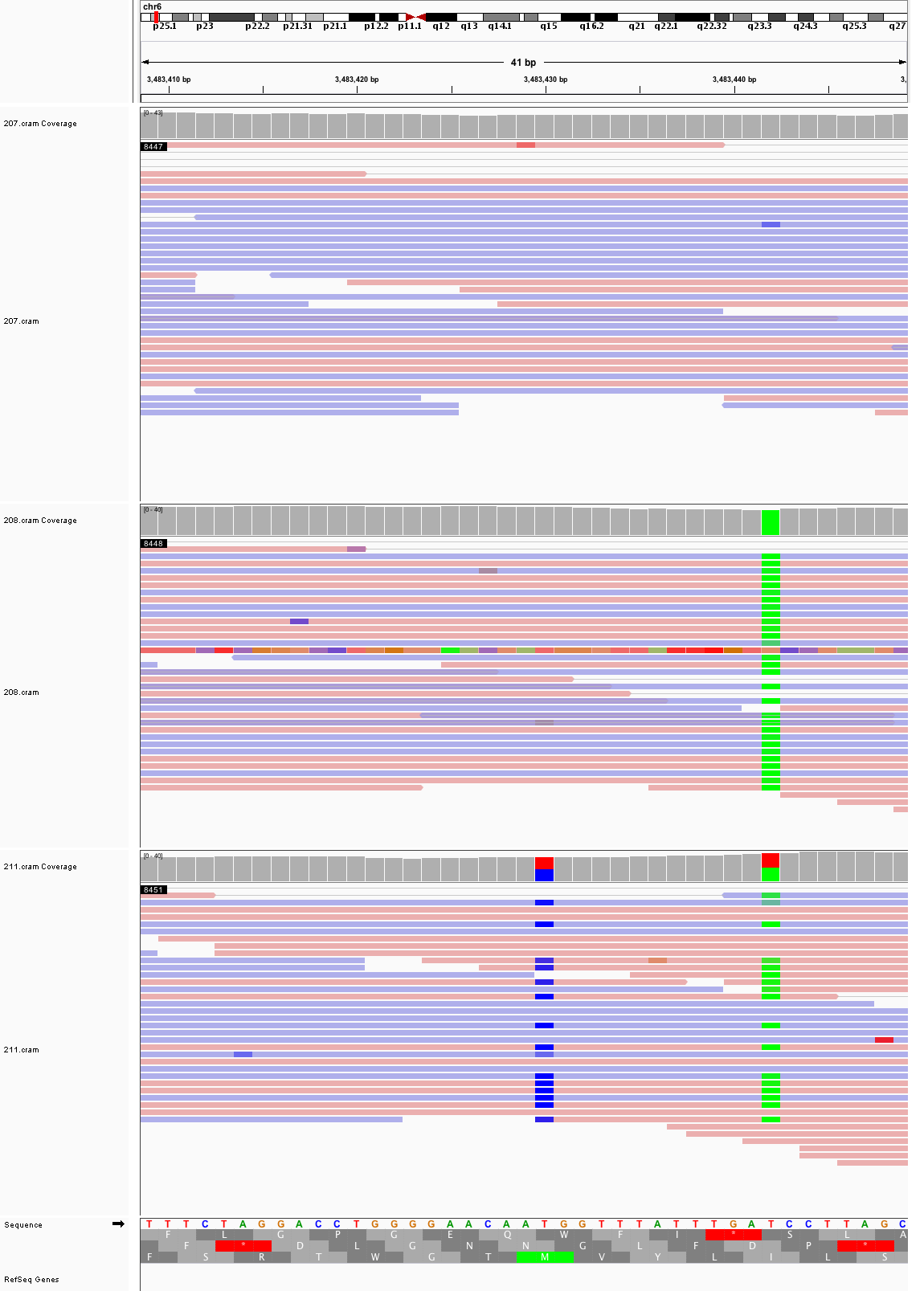

Supplement: Supplementary file 2. — In each image, the first two tracks contain alignments from the first-generation parents, and the third track contains the alignments for the second-generation child. Reads with mapping quality <20 are not included, as they were not considered by our variant calling pipeline, and mismatched bases are shaded by quality score (more transparent = lower base quality). [file elife-46922-supp2.zip › supp_file_2/chr6_3,483,409_3,483,449.png]

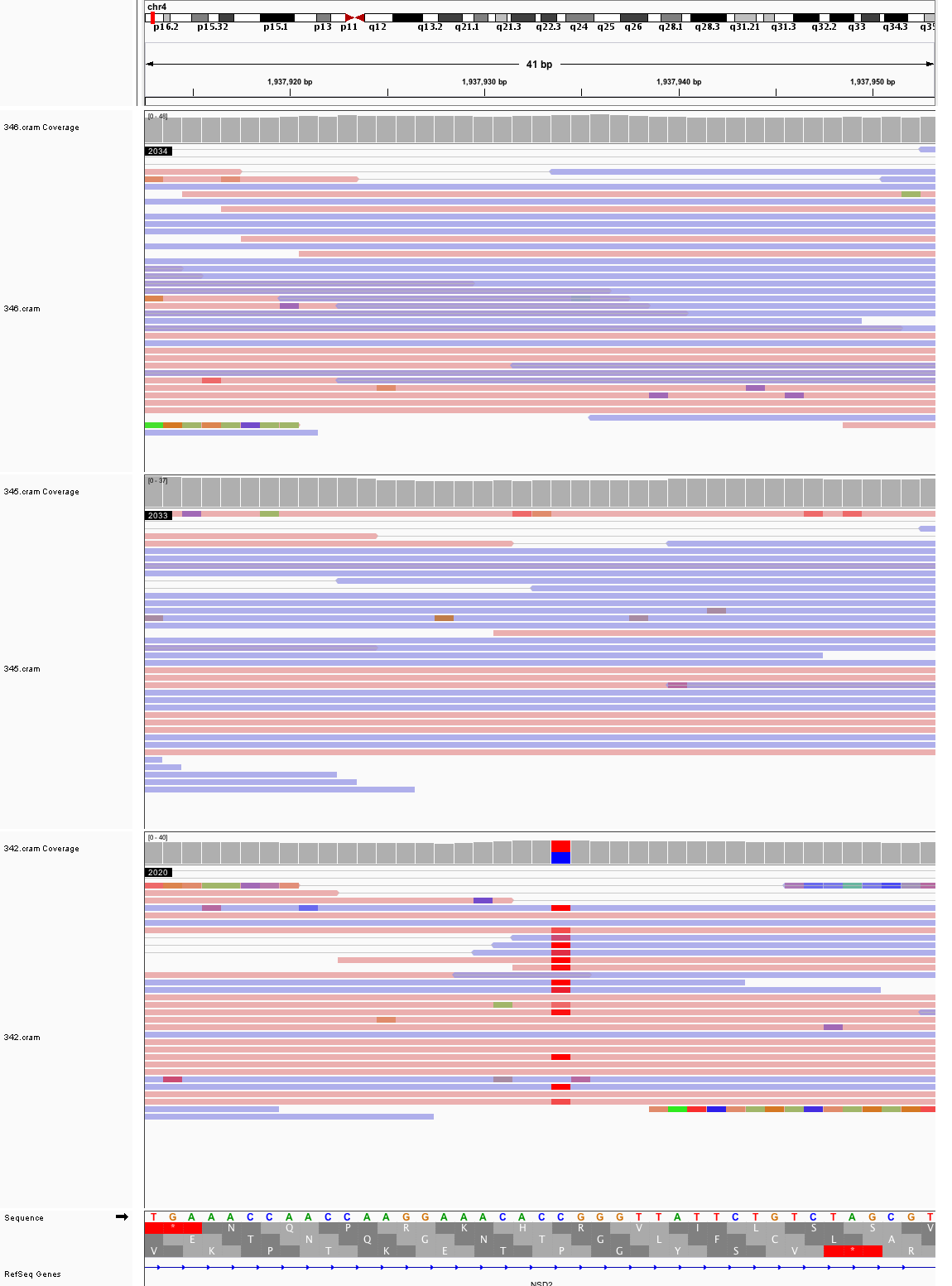

Supplement: Supplementary file 2. — In each image, the first two tracks contain alignments from the first-generation parents, and the third track contains the alignments for the second-generation child. Reads with mapping quality <20 are not included, as they were not considered by our variant calling pipeline, and mismatched bases are shaded by quality score (more transparent = lower base quality). [file elife-46922-supp2.zip › supp_file_2/chr4_1,937,913_1,937,953.png]

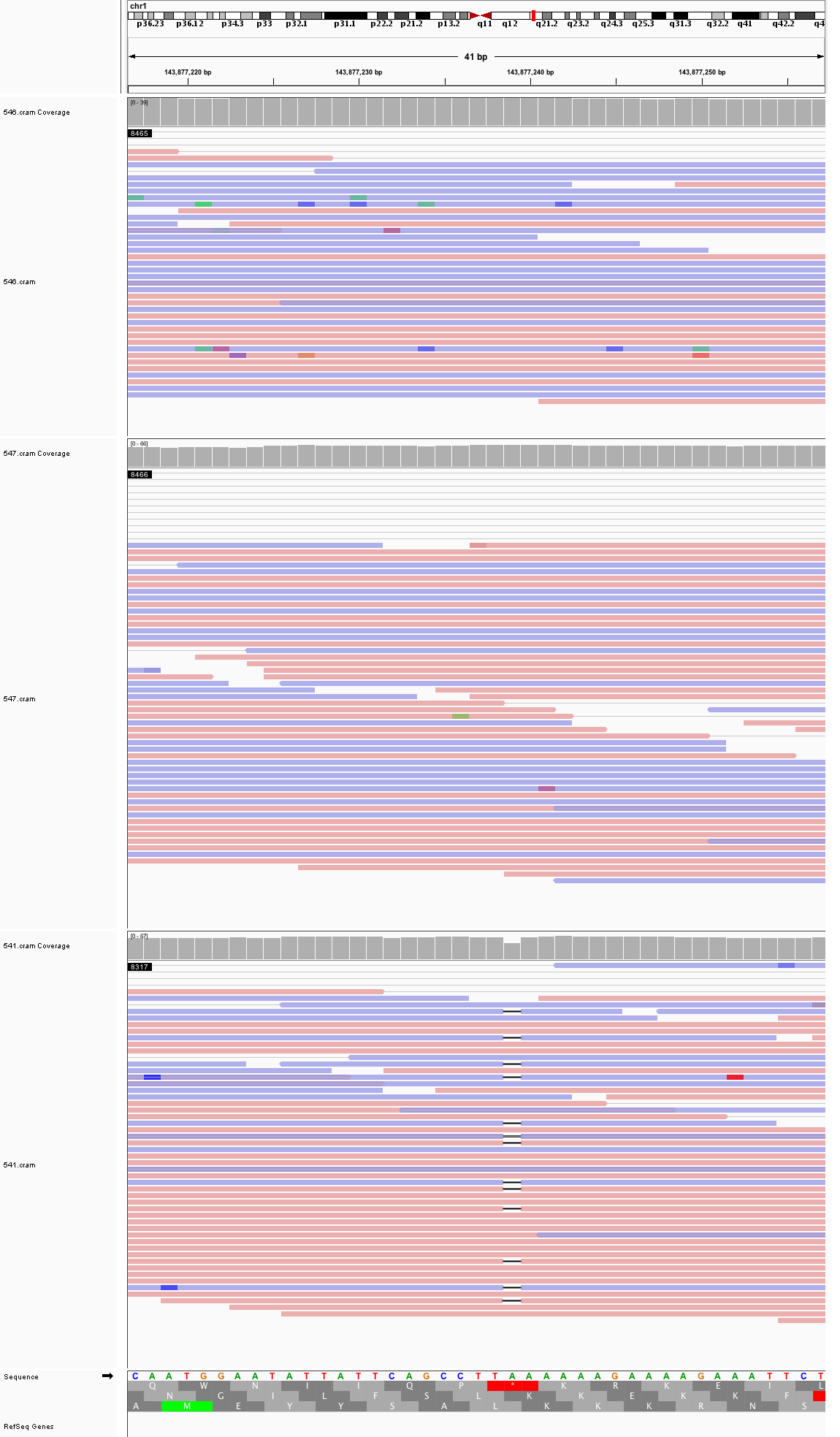

Supplement: Supplementary file 2. — In each image, the first two tracks contain alignments from the first-generation parents, and the third track contains the alignments for the second-generation child. Reads with mapping quality <20 are not included, as they were not considered by our variant calling pipeline, and mismatched bases are shaded by quality score (more transparent = lower base quality). [file elife-46922-supp2.zip › supp_file_2/chr1_143,877,217_143,877,257.png]

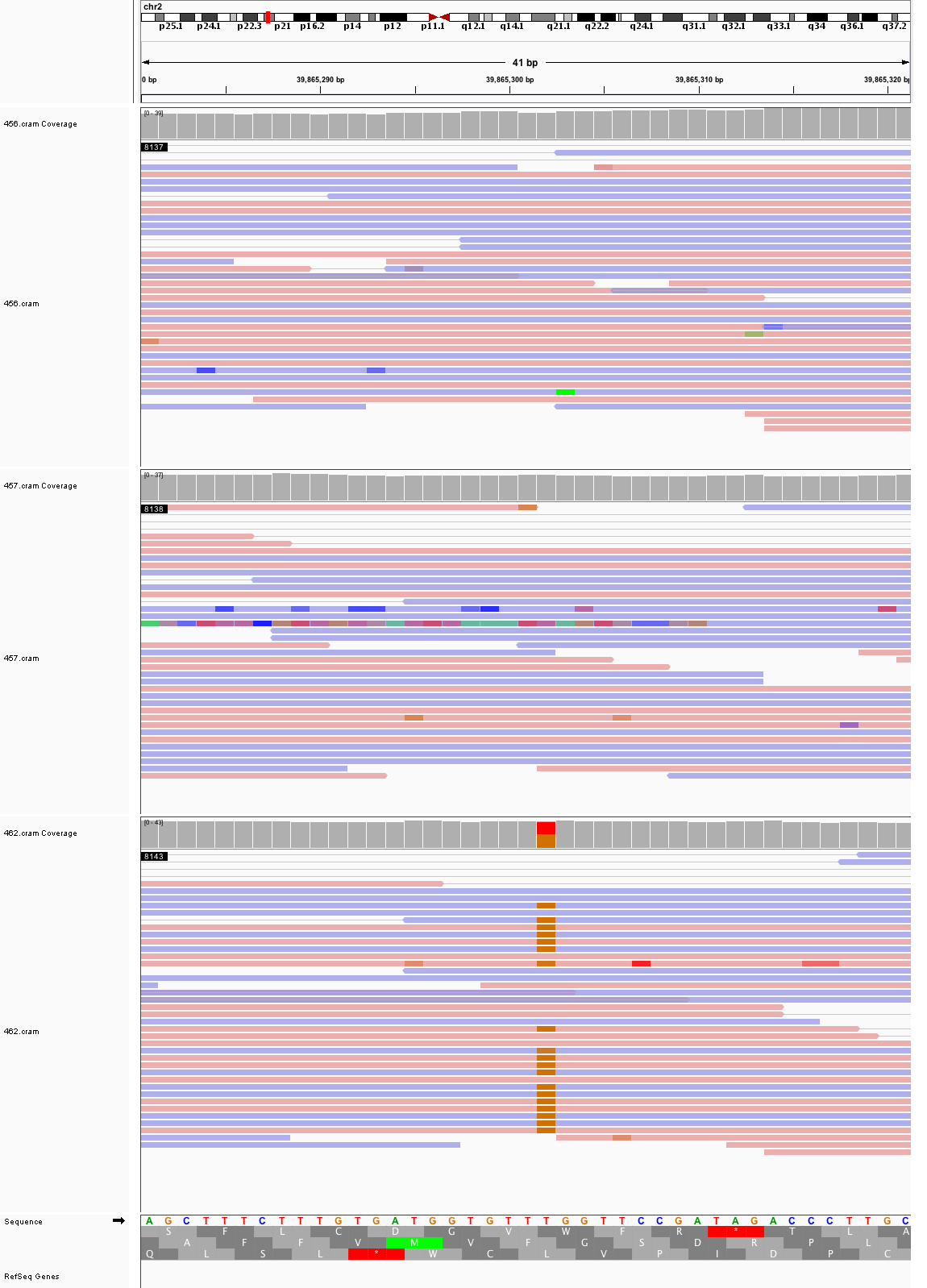

Supplement: Supplementary file 2. — In each image, the first two tracks contain alignments from the first-generation parents, and the third track contains the alignments for the second-generation child. Reads with mapping quality <20 are not included, as they were not considered by our variant calling pipeline, and mismatched bases are shaded by quality score (more transparent = lower base quality). [file elife-46922-supp2.zip › supp_file_2/chr2_39,865,281_39,865,321.png]

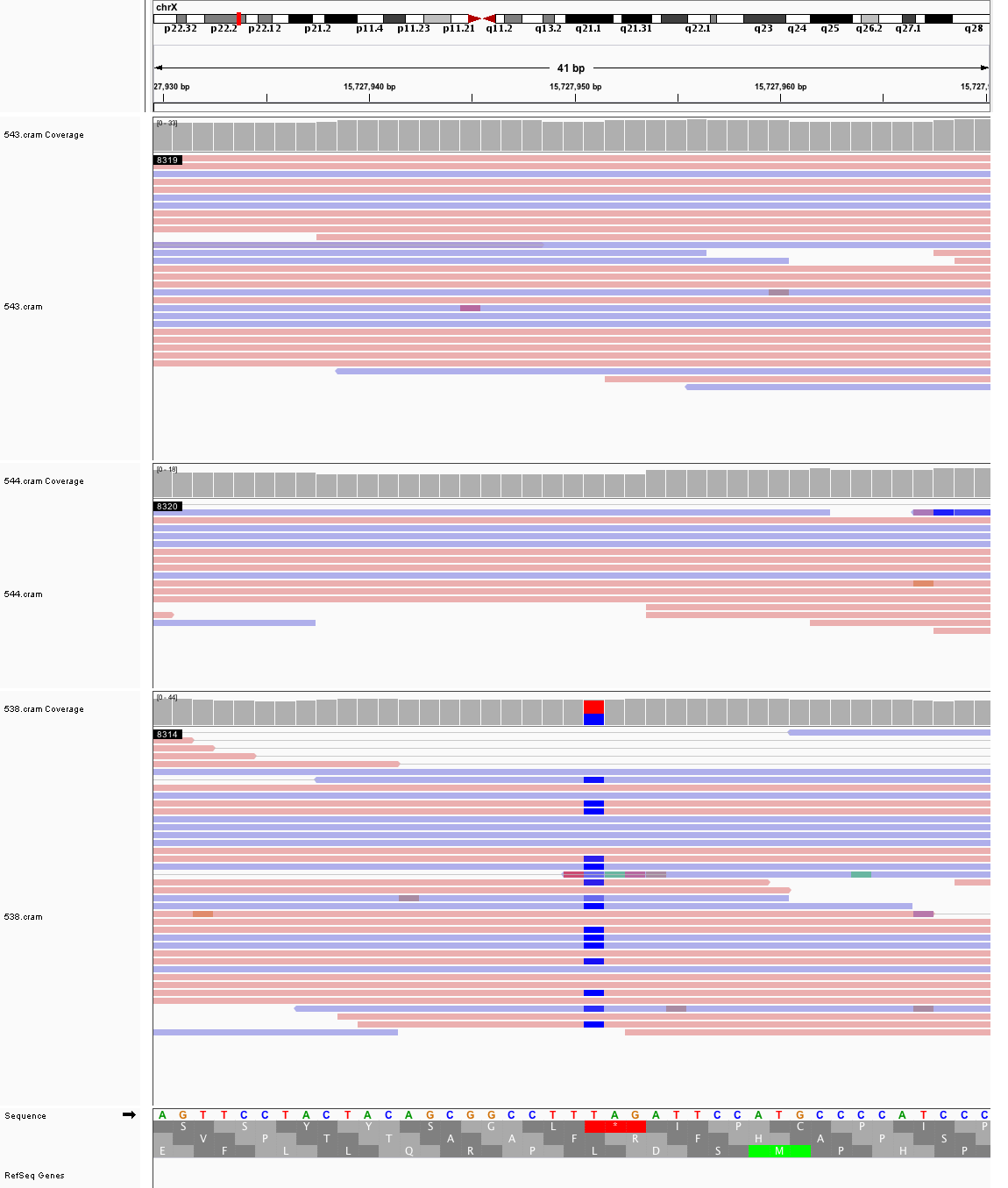

Supplement: Supplementary file 2. — In each image, the first two tracks contain alignments from the first-generation parents, and the third track contains the alignments for the second-generation child. Reads with mapping quality <20 are not included, as they were not considered by our variant calling pipeline, and mismatched bases are shaded by quality score (more transparent = lower base quality). [file elife-46922-supp2.zip › supp_file_2/chrX_15,727,930_15,727,970.png]

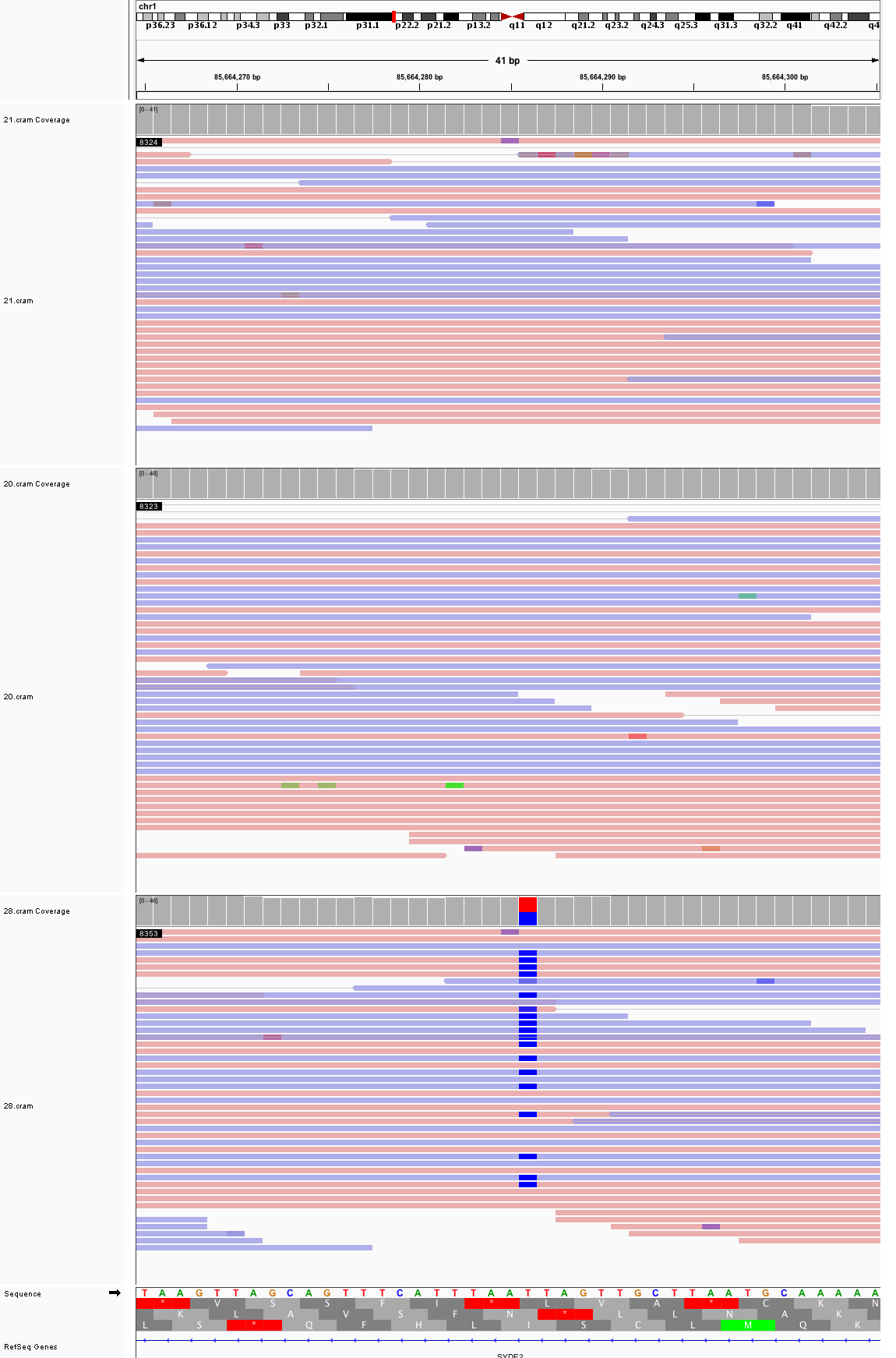

Supplement: Supplementary file 2. — In each image, the first two tracks contain alignments from the first-generation parents, and the third track contains the alignments for the second-generation child. Reads with mapping quality <20 are not included, as they were not considered by our variant calling pipeline, and mismatched bases are shaded by quality score (more transparent = lower base quality). [file elife-46922-supp2.zip › supp_file_2/chr1_85,664,265_85,664,305.png]

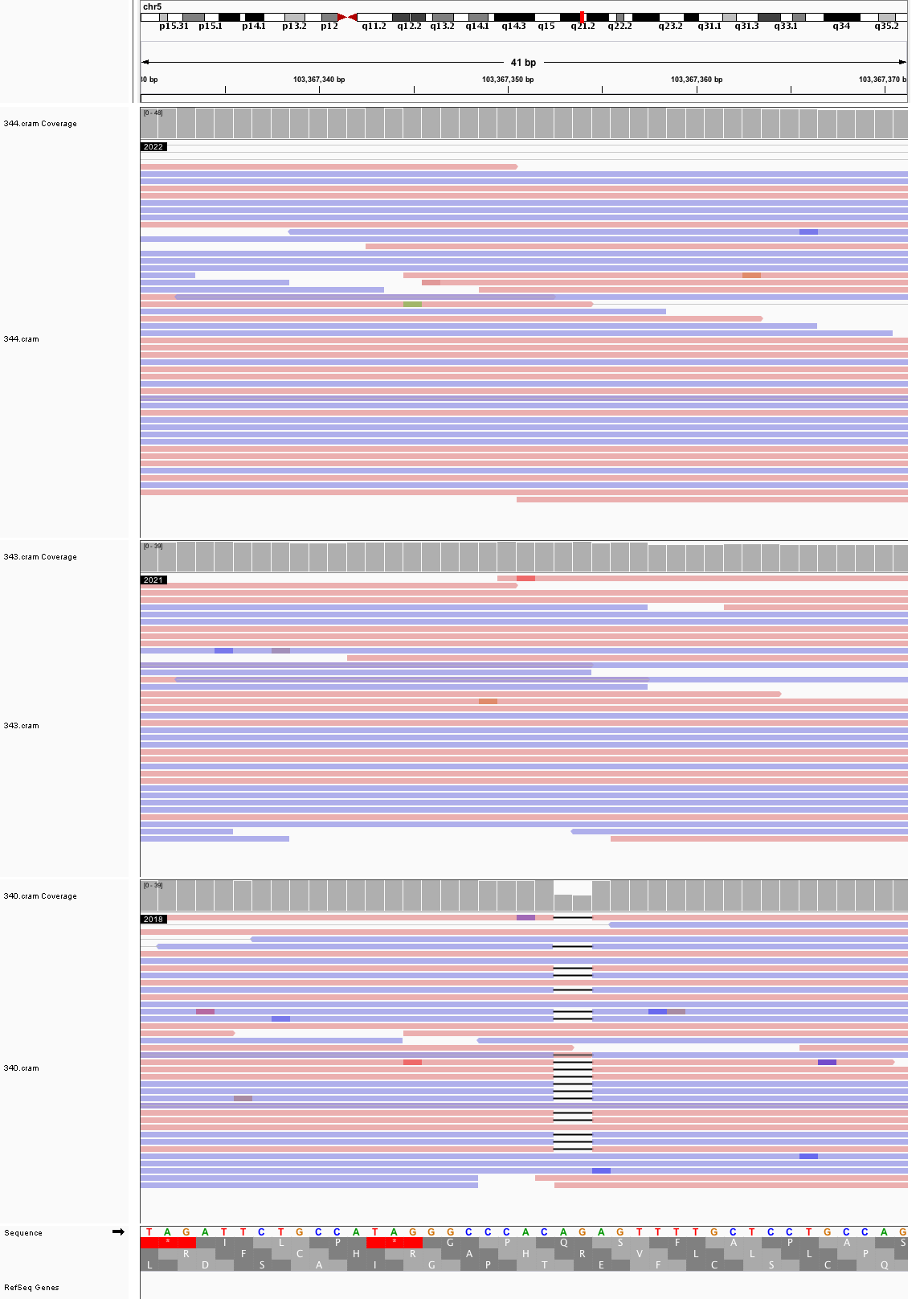

Supplement: Supplementary file 2. — In each image, the first two tracks contain alignments from the first-generation parents, and the third track contains the alignments for the second-generation child. Reads with mapping quality <20 are not included, as they were not considered by our variant calling pipeline, and mismatched bases are shaded by quality score (more transparent = lower base quality). [file elife-46922-supp2.zip › supp_file_2/chr5_103,367,331_103,367,371.png]

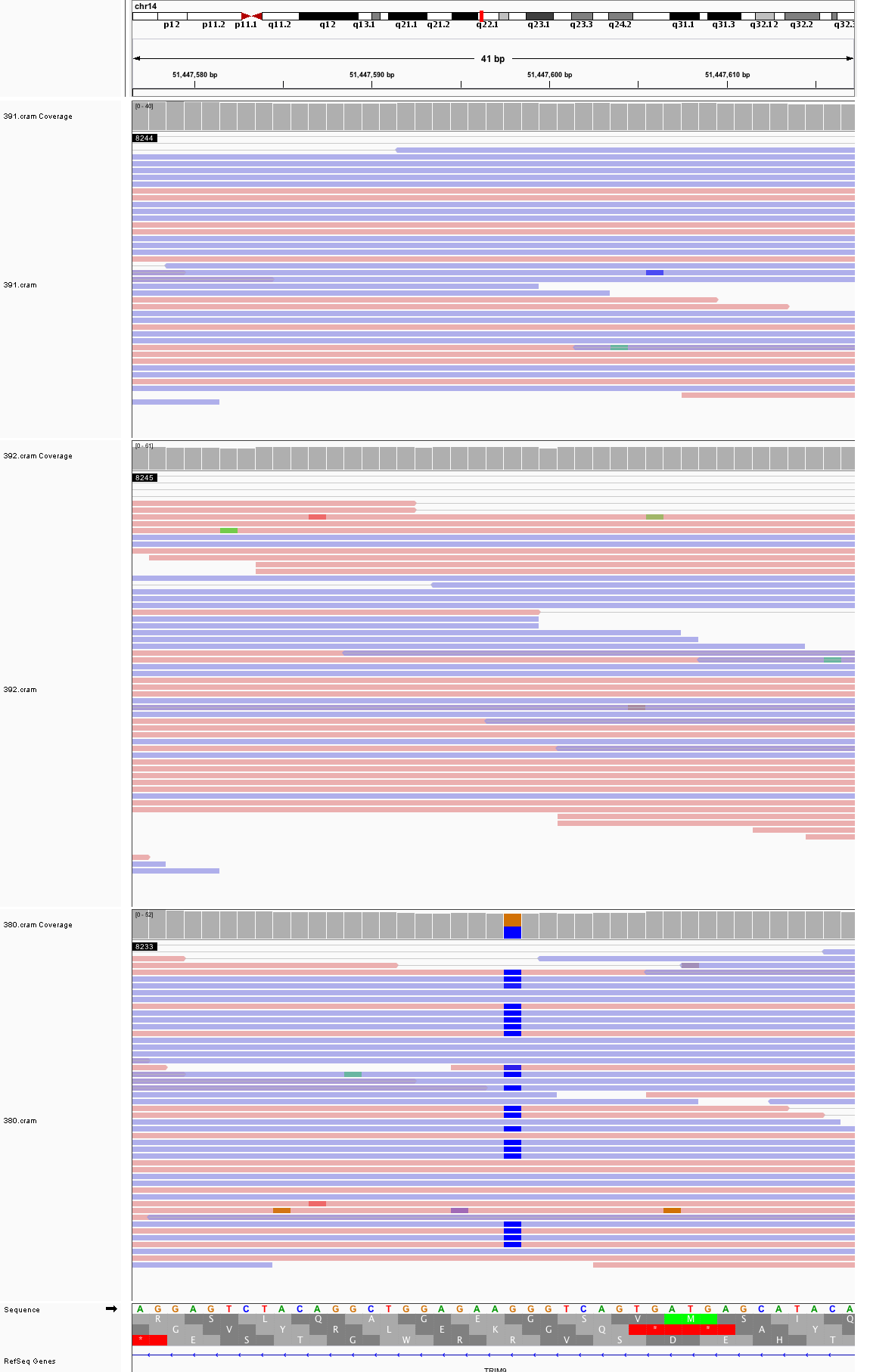

Supplement: Supplementary file 2. — In each image, the first two tracks contain alignments from the first-generation parents, and the third track contains the alignments for the second-generation child. Reads with mapping quality <20 are not included, as they were not considered by our variant calling pipeline, and mismatched bases are shaded by quality score (more transparent = lower base quality). [file elife-46922-supp2.zip › supp_file_2/chr14_51,447,577_51,447,617.png]

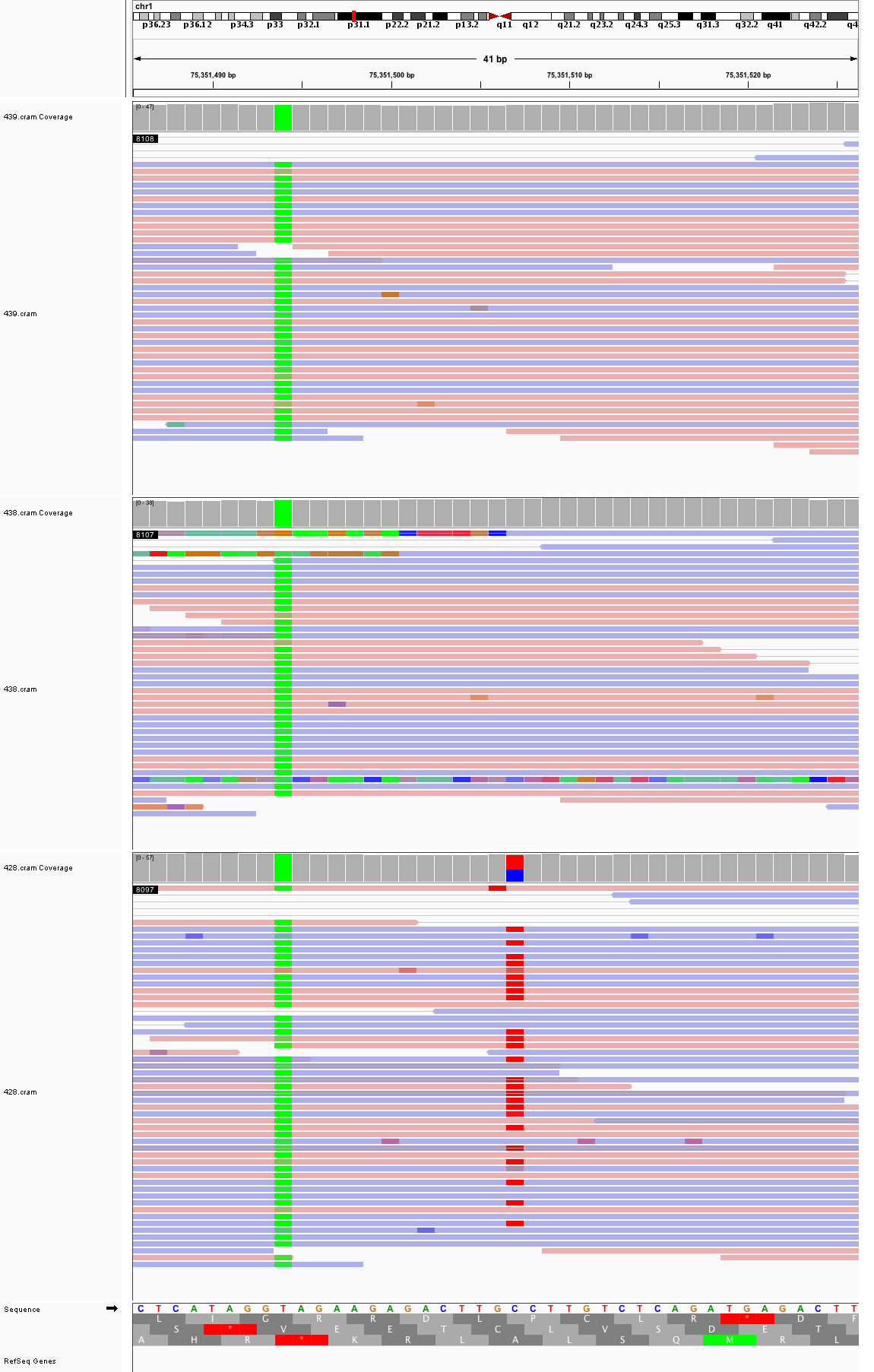

Supplement: Supplementary file 2. — In each image, the first two tracks contain alignments from the first-generation parents, and the third track contains the alignments for the second-generation child. Reads with mapping quality <20 are not included, as they were not considered by our variant calling pipeline, and mismatched bases are shaded by quality score (more transparent = lower base quality). [file elife-46922-supp2.zip › supp_file_2/chr1_75,351,486_75,351,526.png]

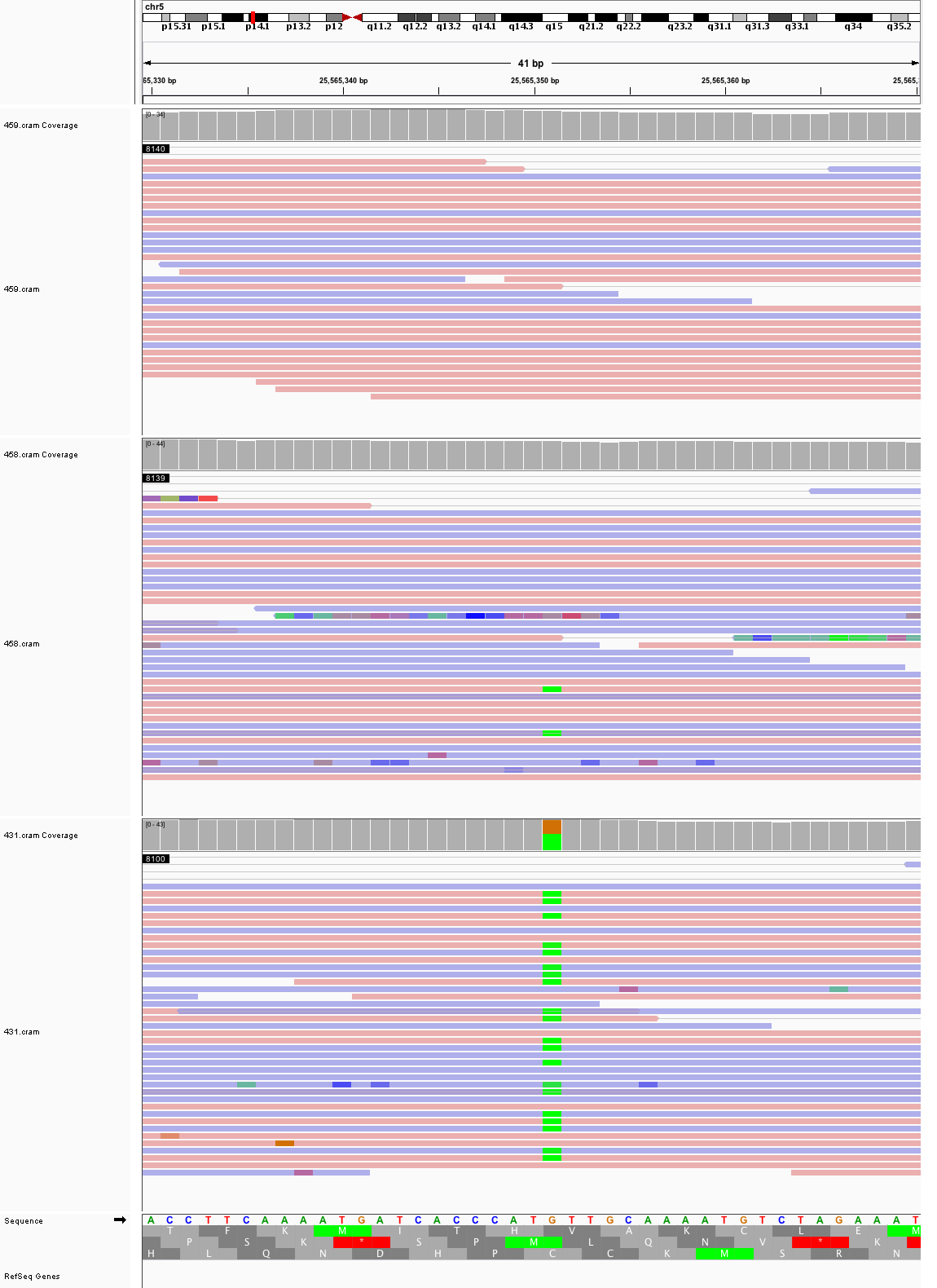

Supplement: Supplementary file 2. — In each image, the first two tracks contain alignments from the first-generation parents, and the third track contains the alignments for the second-generation child. Reads with mapping quality <20 are not included, as they were not considered by our variant calling pipeline, and mismatched bases are shaded by quality score (more transparent = lower base quality). [file elife-46922-supp2.zip › supp_file_2/chr5_25,565,330_25,565,370.png]

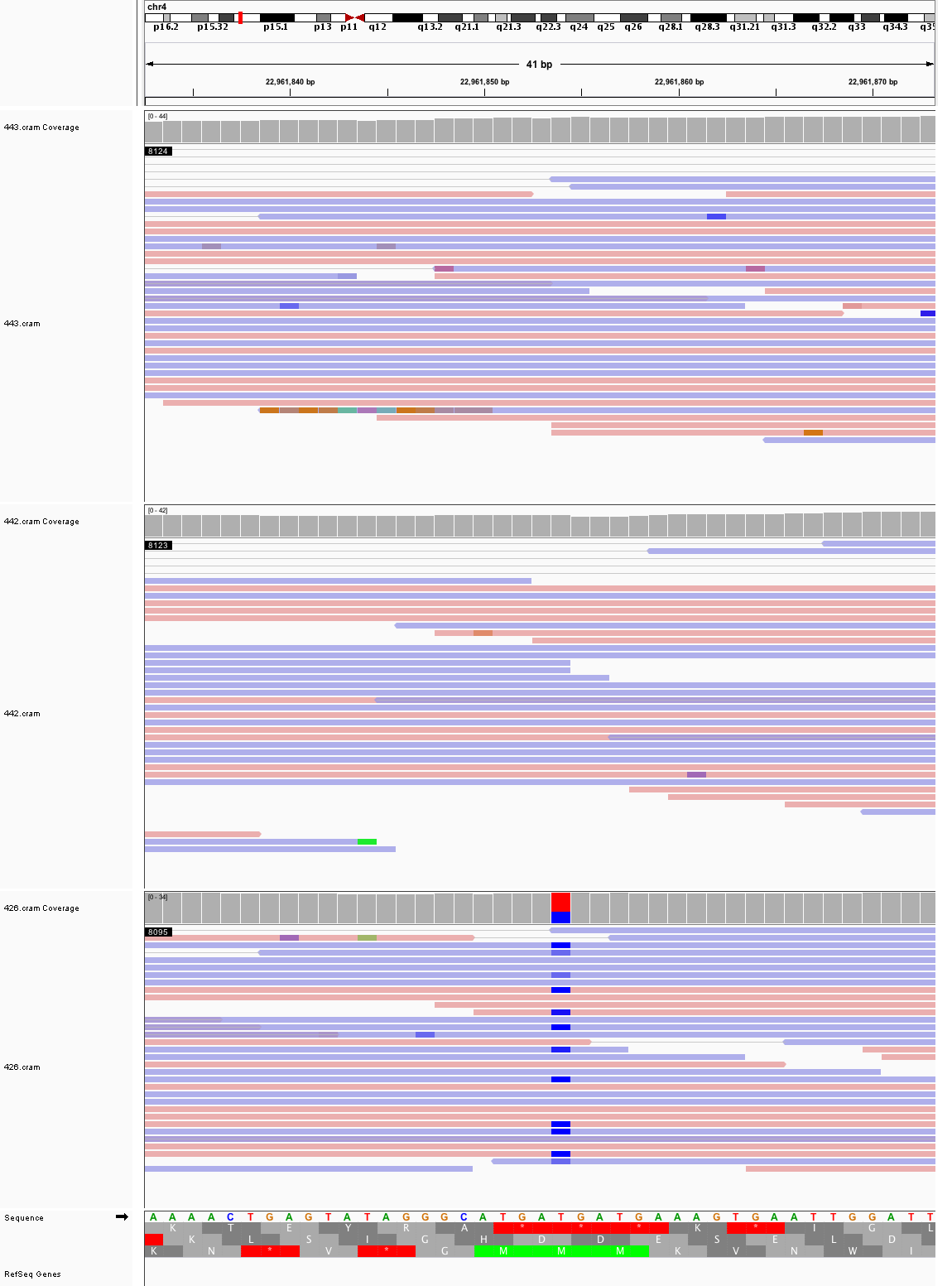

Supplement: Supplementary file 2. — In each image, the first two tracks contain alignments from the first-generation parents, and the third track contains the alignments for the second-generation child. Reads with mapping quality <20 are not included, as they were not considered by our variant calling pipeline, and mismatched bases are shaded by quality score (more transparent = lower base quality). [file elife-46922-supp2.zip › supp_file_2/chr4_22,961,833_22,961,873.png]

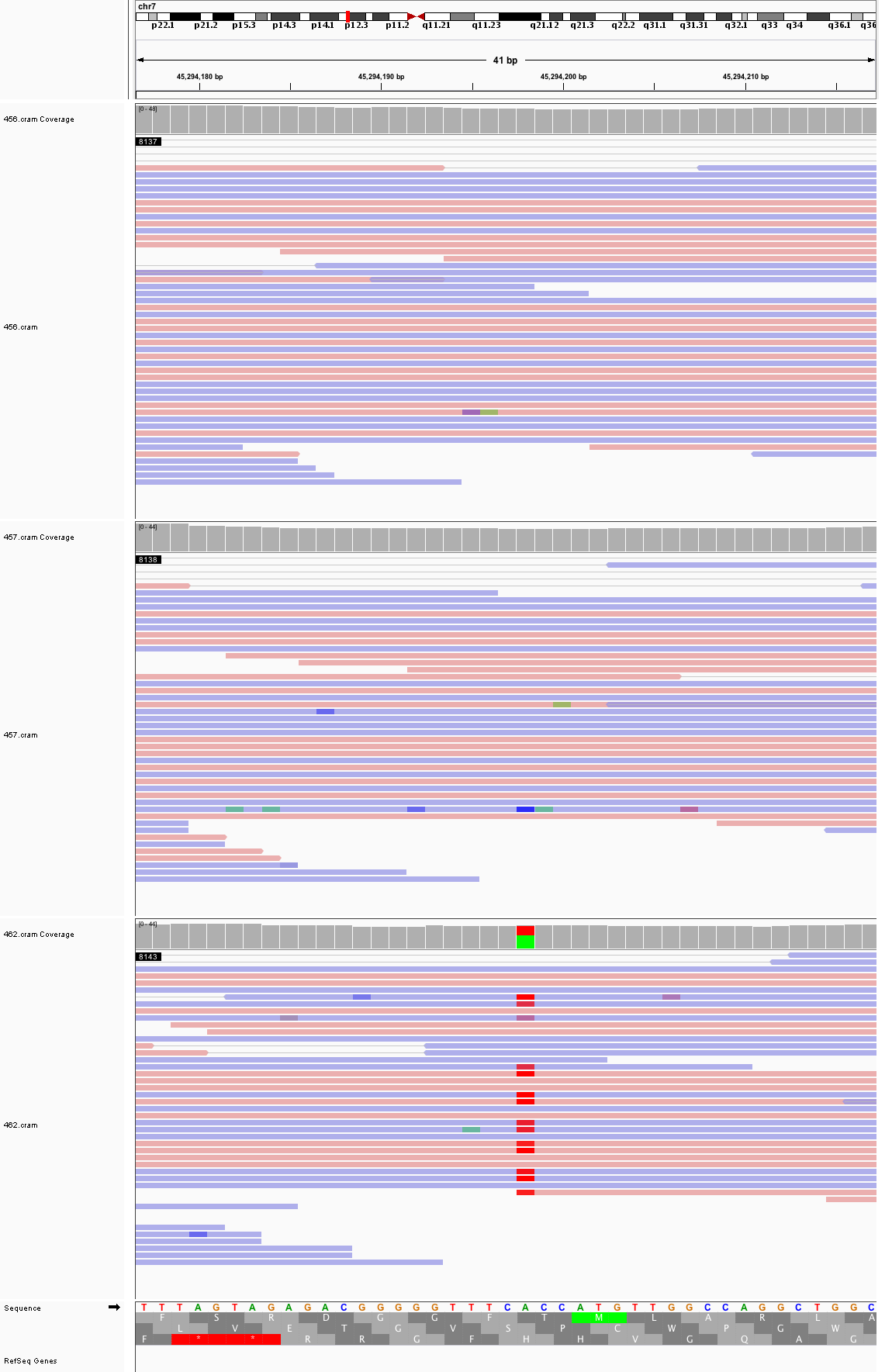

Supplement: Supplementary file 2. — In each image, the first two tracks contain alignments from the first-generation parents, and the third track contains the alignments for the second-generation child. Reads with mapping quality <20 are not included, as they were not considered by our variant calling pipeline, and mismatched bases are shaded by quality score (more transparent = lower base quality). [file elife-46922-supp2.zip › supp_file_2/chr7_45,294,177_45,294,217.png]

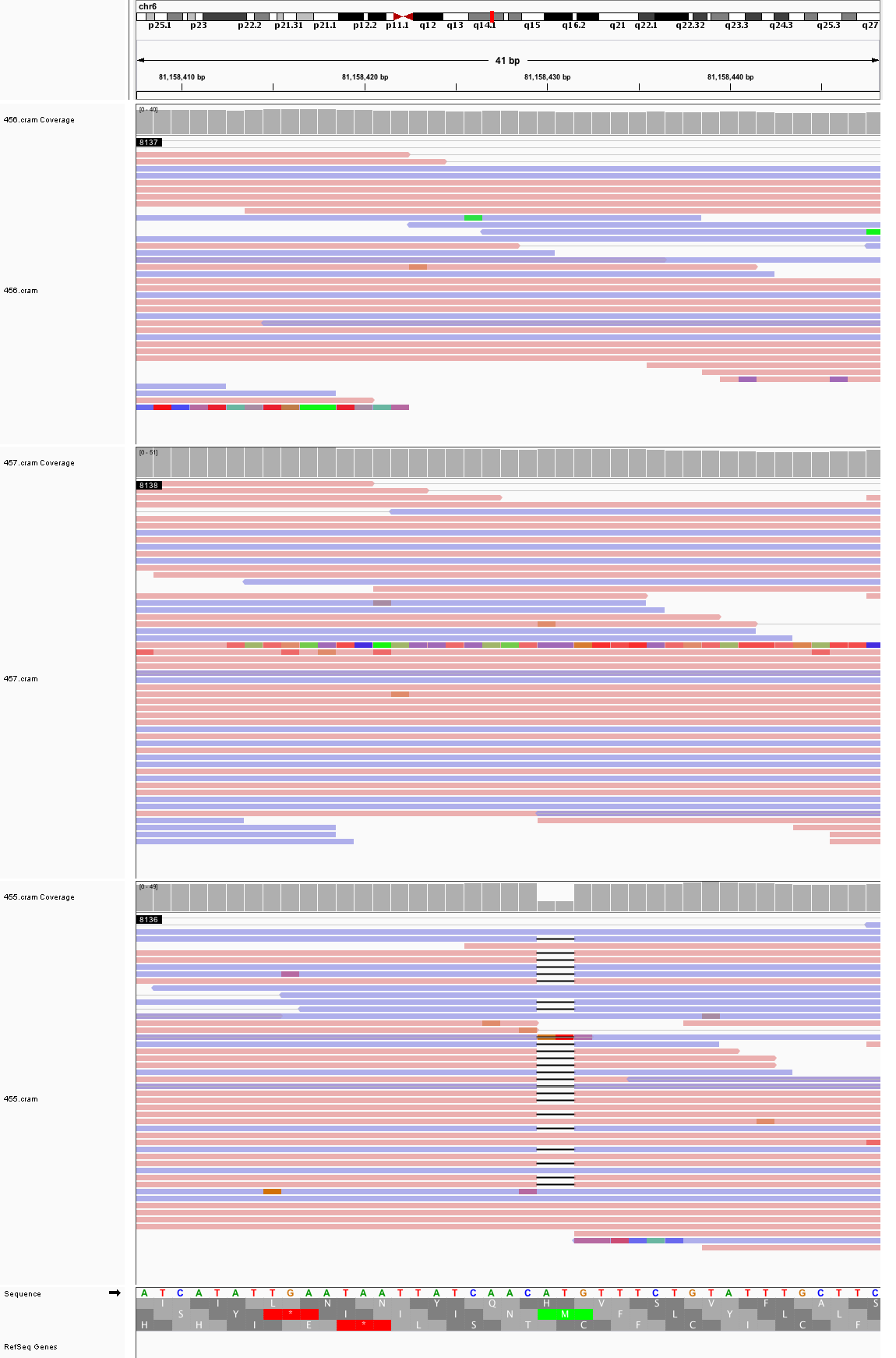

Supplement: Supplementary file 2. — In each image, the first two tracks contain alignments from the first-generation parents, and the third track contains the alignments for the second-generation child. Reads with mapping quality <20 are not included, as they were not considered by our variant calling pipeline, and mismatched bases are shaded by quality score (more transparent = lower base quality). [file elife-46922-supp2.zip › supp_file_2/chr6_81,158,408_81,158,448.png]

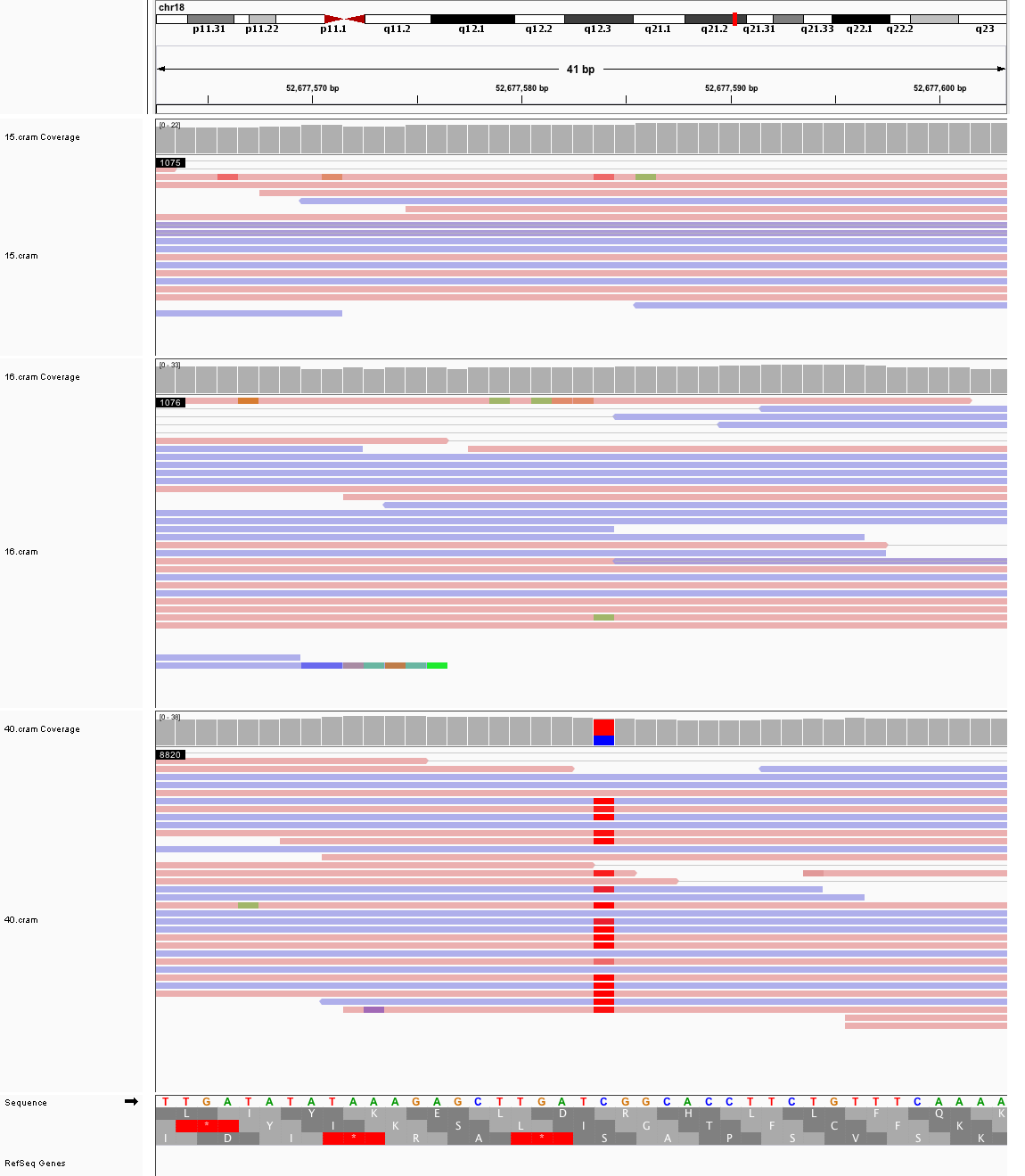

Supplement: Supplementary file 2. — In each image, the first two tracks contain alignments from the first-generation parents, and the third track contains the alignments for the second-generation child. Reads with mapping quality <20 are not included, as they were not considered by our variant calling pipeline, and mismatched bases are shaded by quality score (more transparent = lower base quality). [file elife-46922-supp2.zip › supp_file_2/chr18_52,677,563_52,677,603.png]

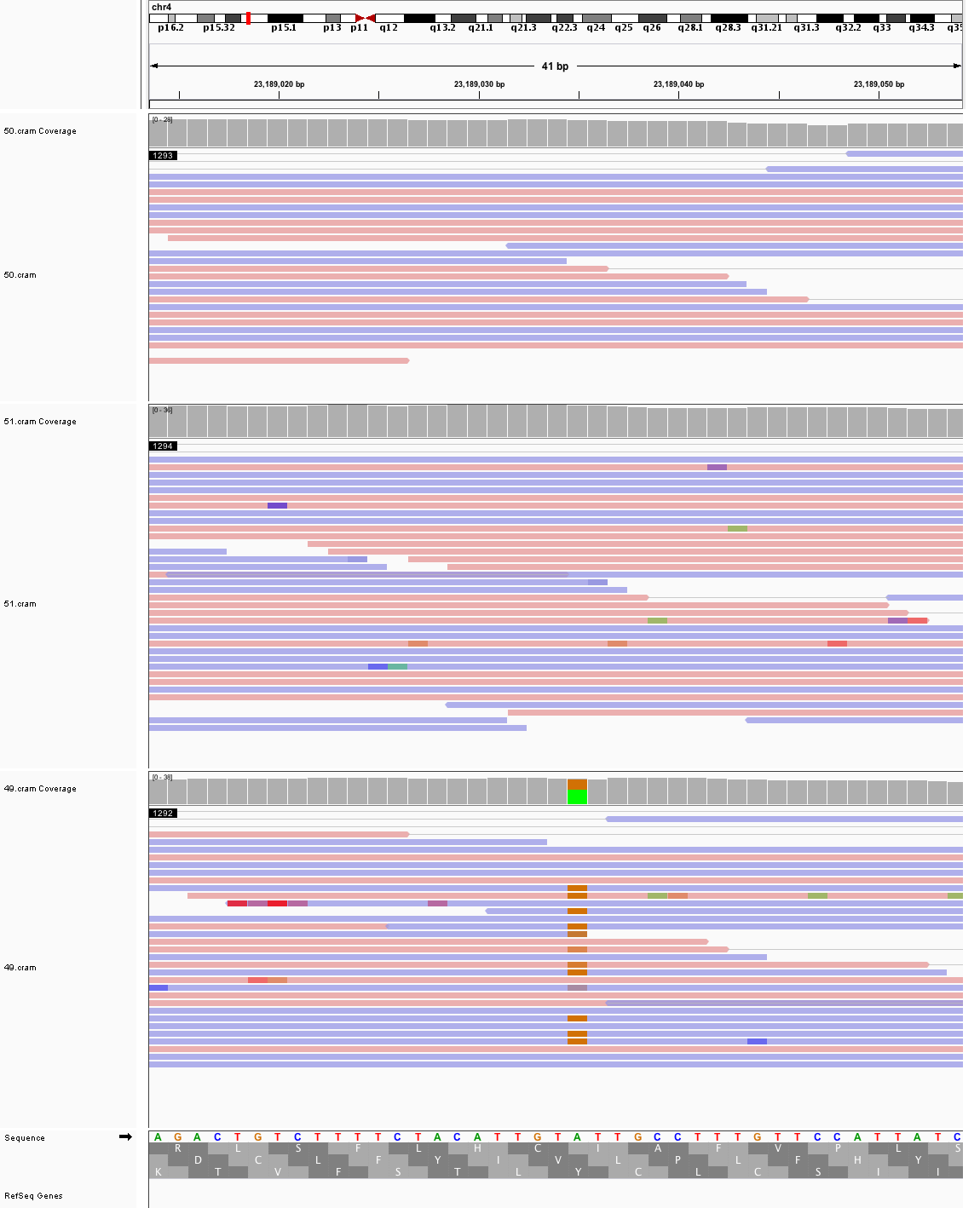

Supplement: Supplementary file 2. — In each image, the first two tracks contain alignments from the first-generation parents, and the third track contains the alignments for the second-generation child. Reads with mapping quality <20 are not included, as they were not considered by our variant calling pipeline, and mismatched bases are shaded by quality score (more transparent = lower base quality). [file elife-46922-supp2.zip › supp_file_2/chr4_23,189,014_23,189,054.png]

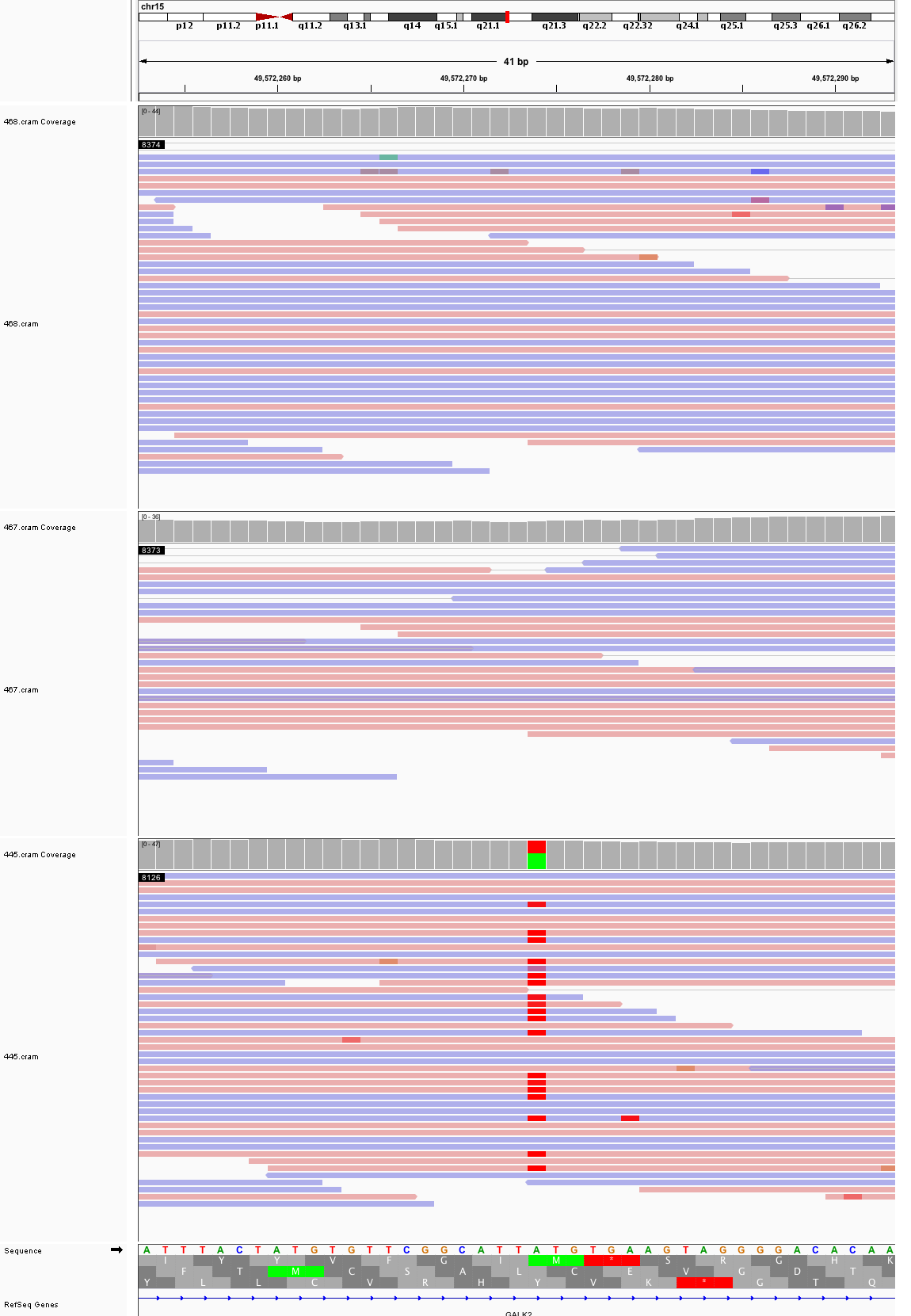

Supplement: Supplementary file 2. — In each image, the first two tracks contain alignments from the first-generation parents, and the third track contains the alignments for the second-generation child. Reads with mapping quality <20 are not included, as they were not considered by our variant calling pipeline, and mismatched bases are shaded by quality score (more transparent = lower base quality). [file elife-46922-supp2.zip › supp_file_2/chr15_49,572,253_49,572,293.png]

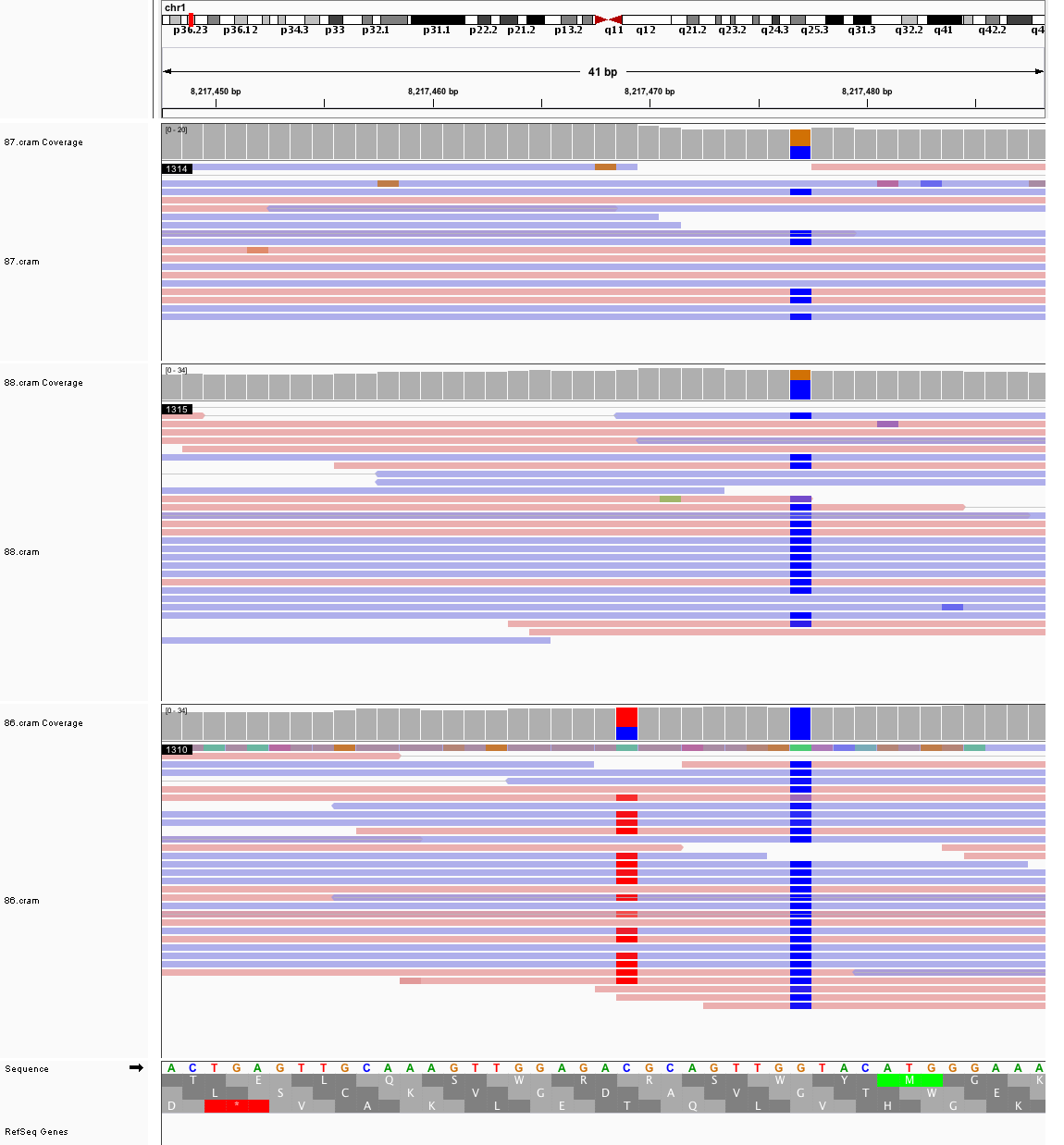

Supplement: Supplementary file 2. — In each image, the first two tracks contain alignments from the first-generation parents, and the third track contains the alignments for the second-generation child. Reads with mapping quality <20 are not included, as they were not considered by our variant calling pipeline, and mismatched bases are shaded by quality score (more transparent = lower base quality). [file elife-46922-supp2.zip › supp_file_2/chr1_8,217,448_8,217,488.png]

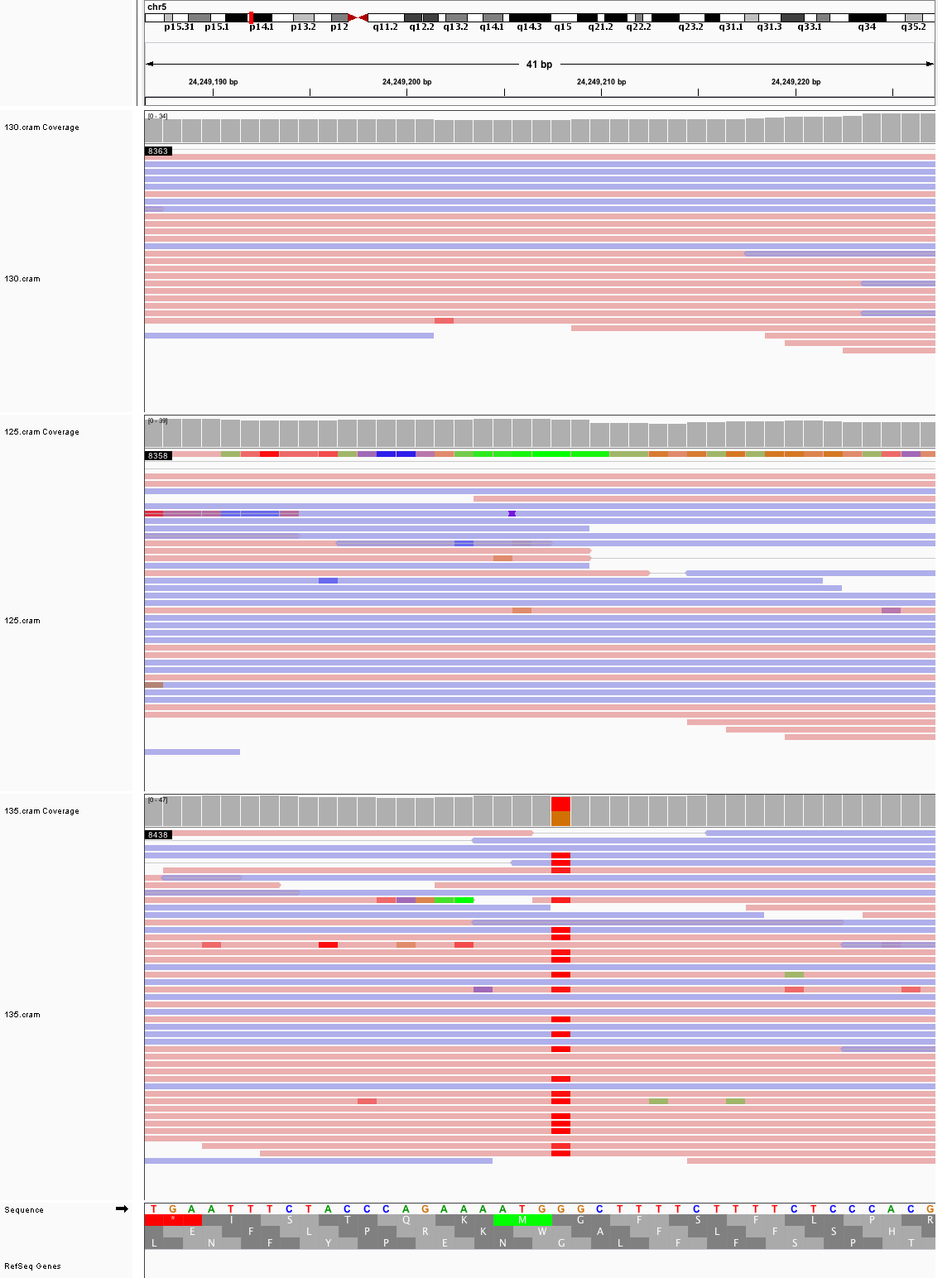

Supplement: Supplementary file 2. — In each image, the first two tracks contain alignments from the first-generation parents, and the third track contains the alignments for the second-generation child. Reads with mapping quality <20 are not included, as they were not considered by our variant calling pipeline, and mismatched bases are shaded by quality score (more transparent = lower base quality). [file elife-46922-supp2.zip › supp_file_2/chr5_24,249,187_24,249,227.png]

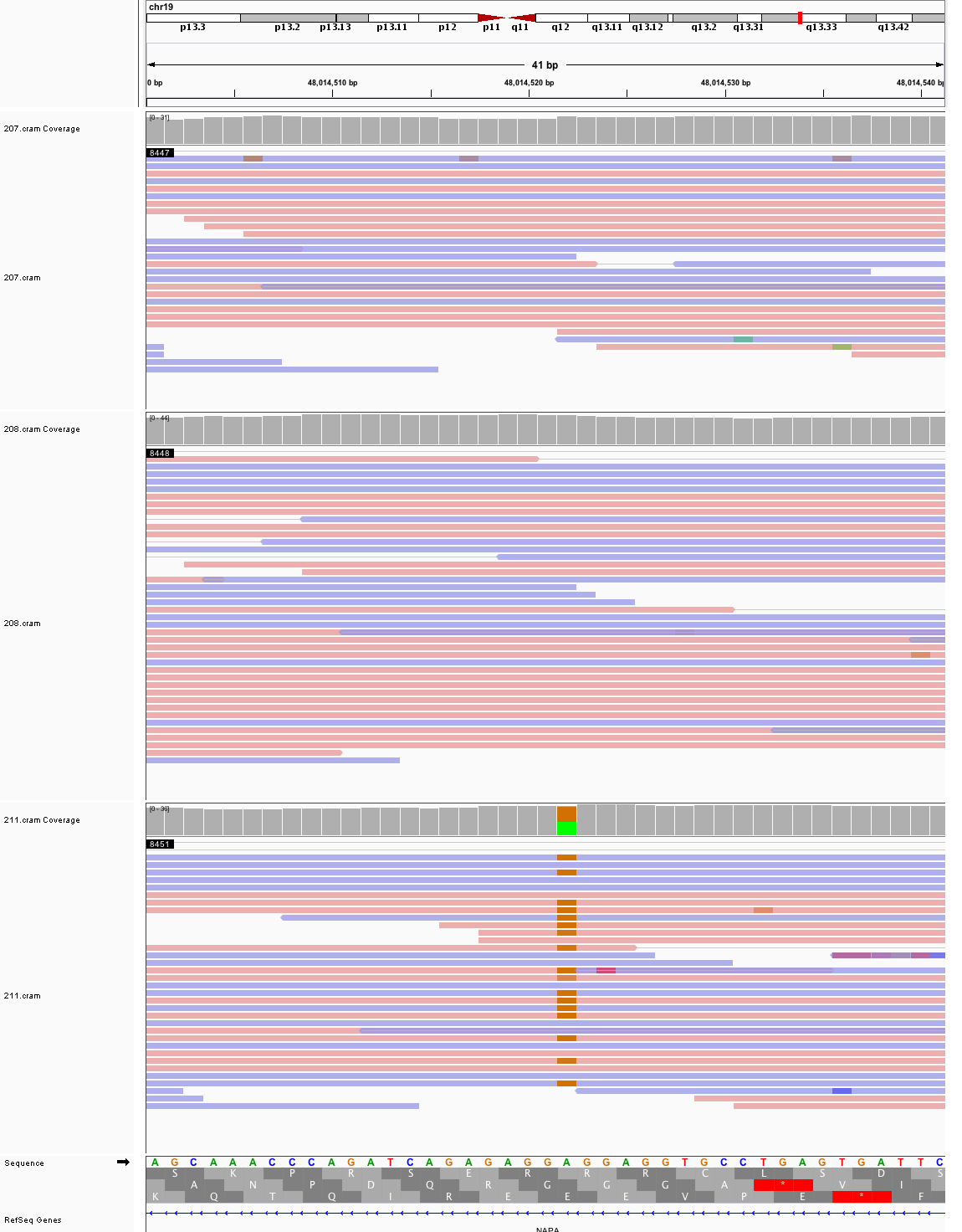

Supplement: Supplementary file 2. — In each image, the first two tracks contain alignments from the first-generation parents, and the third track contains the alignments for the second-generation child. Reads with mapping quality <20 are not included, as they were not considered by our variant calling pipeline, and mismatched bases are shaded by quality score (more transparent = lower base quality). [file elife-46922-supp2.zip › supp_file_2/chr19_48,014,501_48,014,541.png]

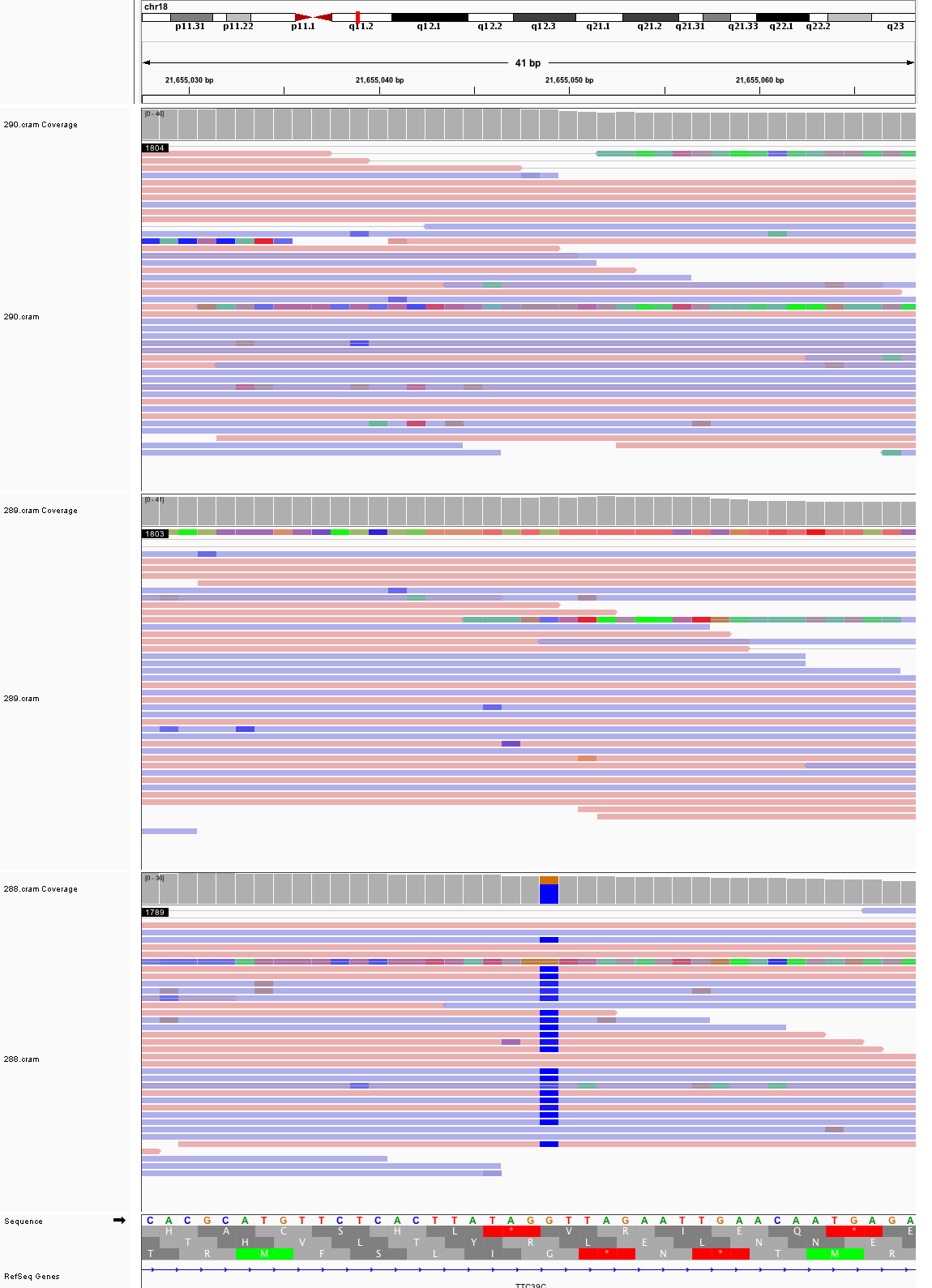

Supplement: Supplementary file 2. — In each image, the first two tracks contain alignments from the first-generation parents, and the third track contains the alignments for the second-generation child. Reads with mapping quality <20 are not included, as they were not considered by our variant calling pipeline, and mismatched bases are shaded by quality score (more transparent = lower base quality). [file elife-46922-supp2.zip › supp_file_2/chr18_21,655,028_21,655,068.png]

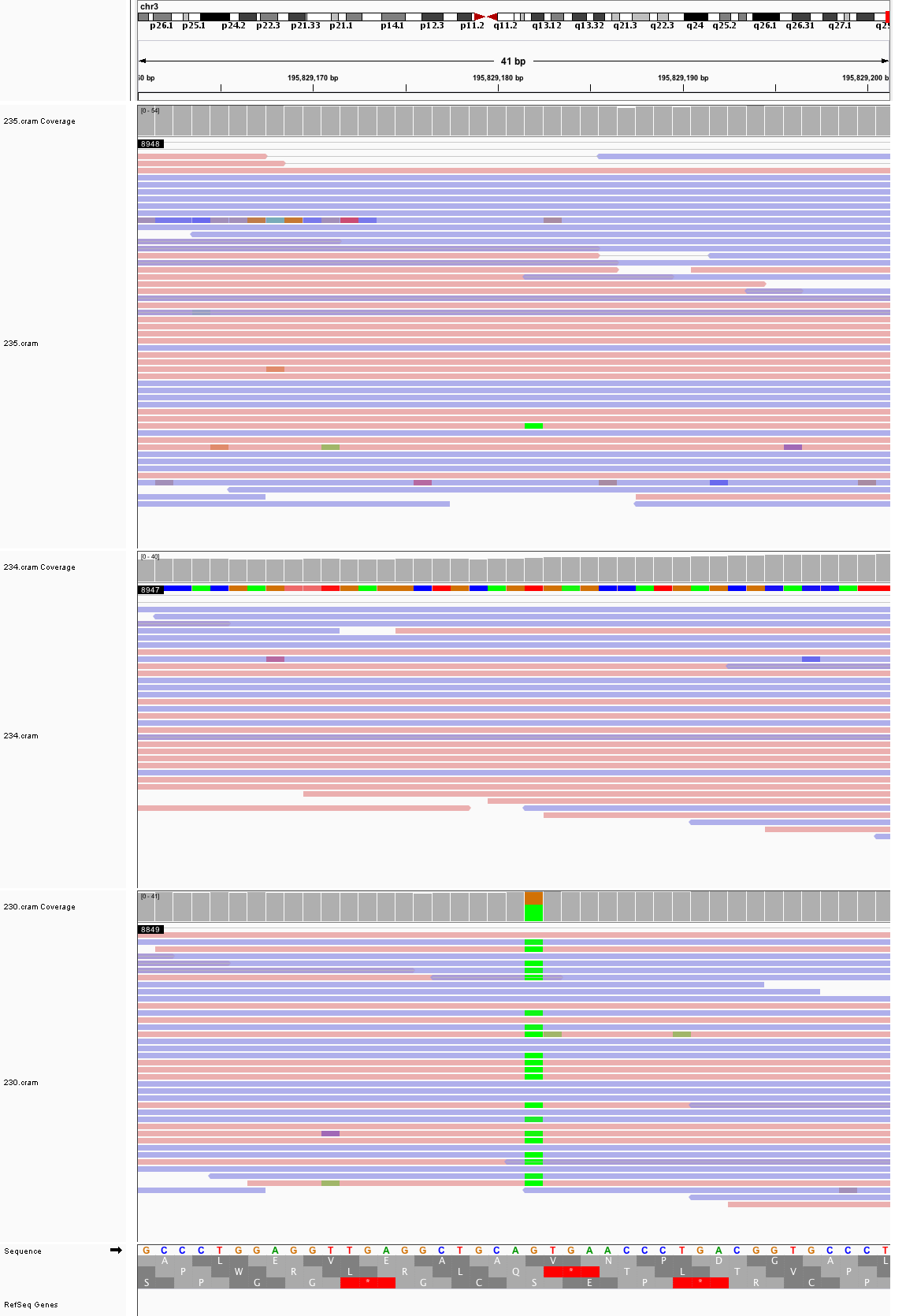

Supplement: Supplementary file 2. — In each image, the first two tracks contain alignments from the first-generation parents, and the third track contains the alignments for the second-generation child. Reads with mapping quality <20 are not included, as they were not considered by our variant calling pipeline, and mismatched bases are shaded by quality score (more transparent = lower base quality). [file elife-46922-supp2.zip › supp_file_2/chr3_195,829,161_195,829,201.png]

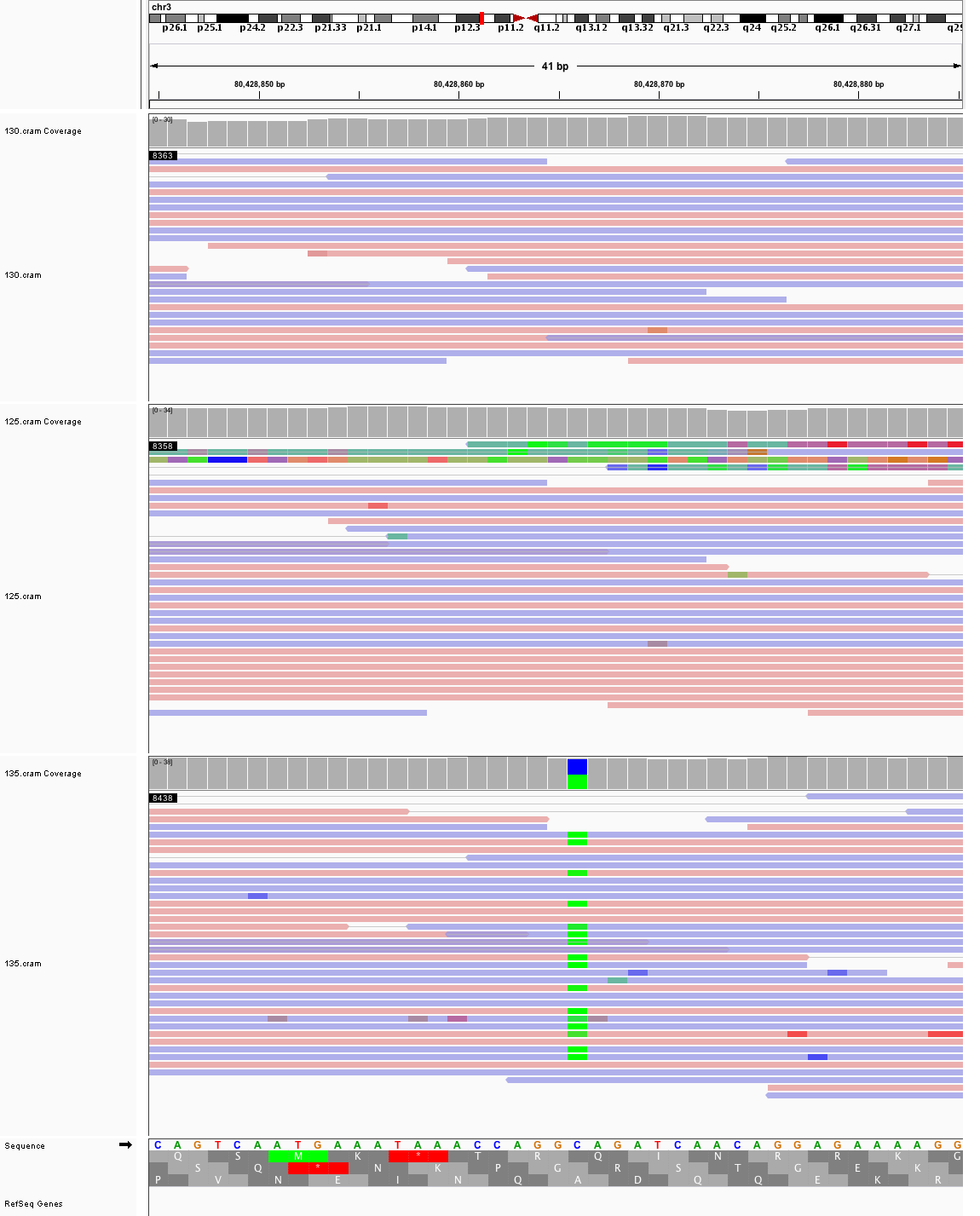

Supplement: Supplementary file 2. — In each image, the first two tracks contain alignments from the first-generation parents, and the third track contains the alignments for the second-generation child. Reads with mapping quality <20 are not included, as they were not considered by our variant calling pipeline, and mismatched bases are shaded by quality score (more transparent = lower base quality). [file elife-46922-supp2.zip › supp_file_2/chr3_80,428,845_80,428,885.png]

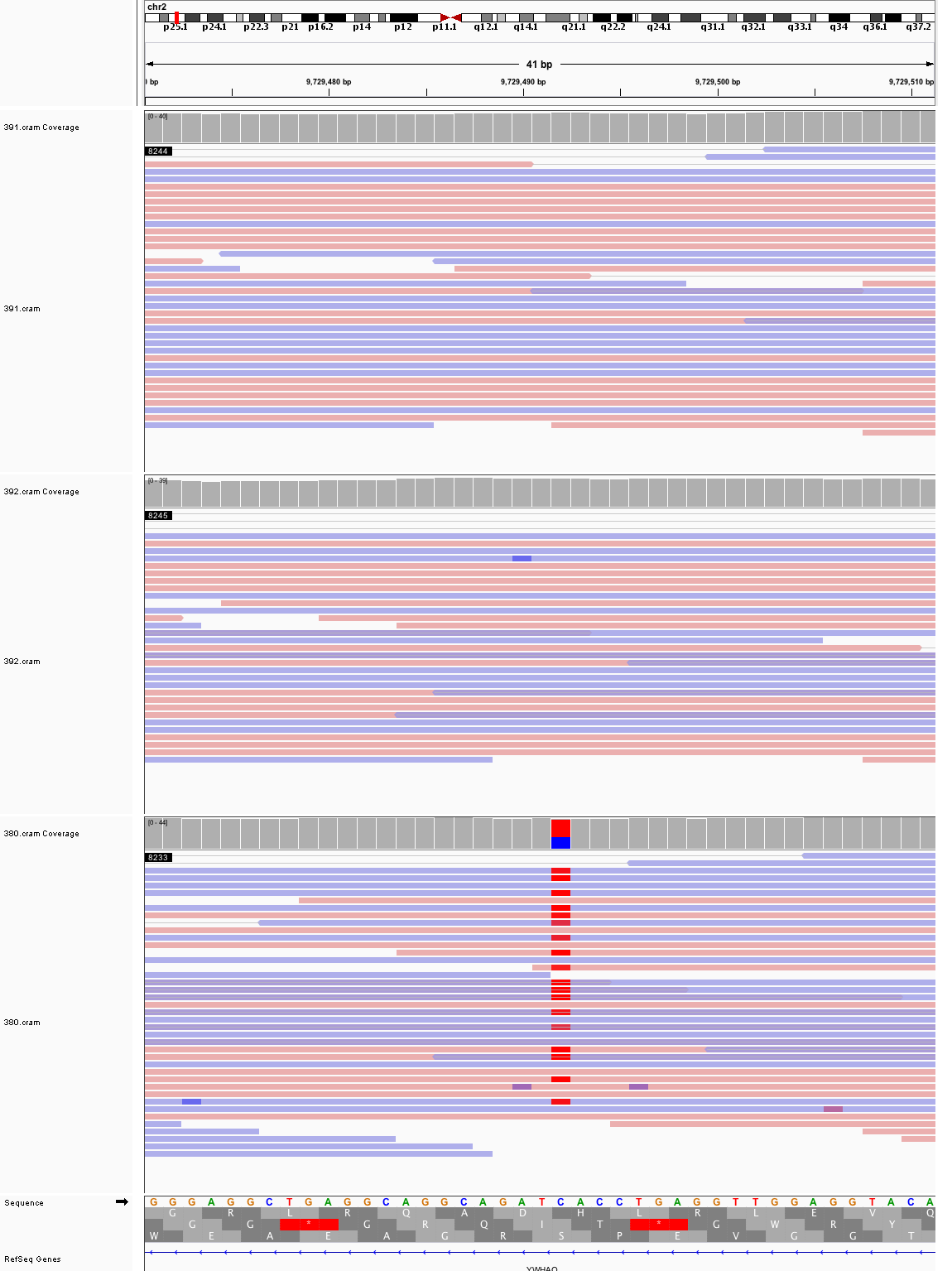

Supplement: Supplementary file 2. — In each image, the first two tracks contain alignments from the first-generation parents, and the third track contains the alignments for the second-generation child. Reads with mapping quality <20 are not included, as they were not considered by our variant calling pipeline, and mismatched bases are shaded by quality score (more transparent = lower base quality). [file elife-46922-supp2.zip › supp_file_2/chr2_9,729,471_9,729,511.png]

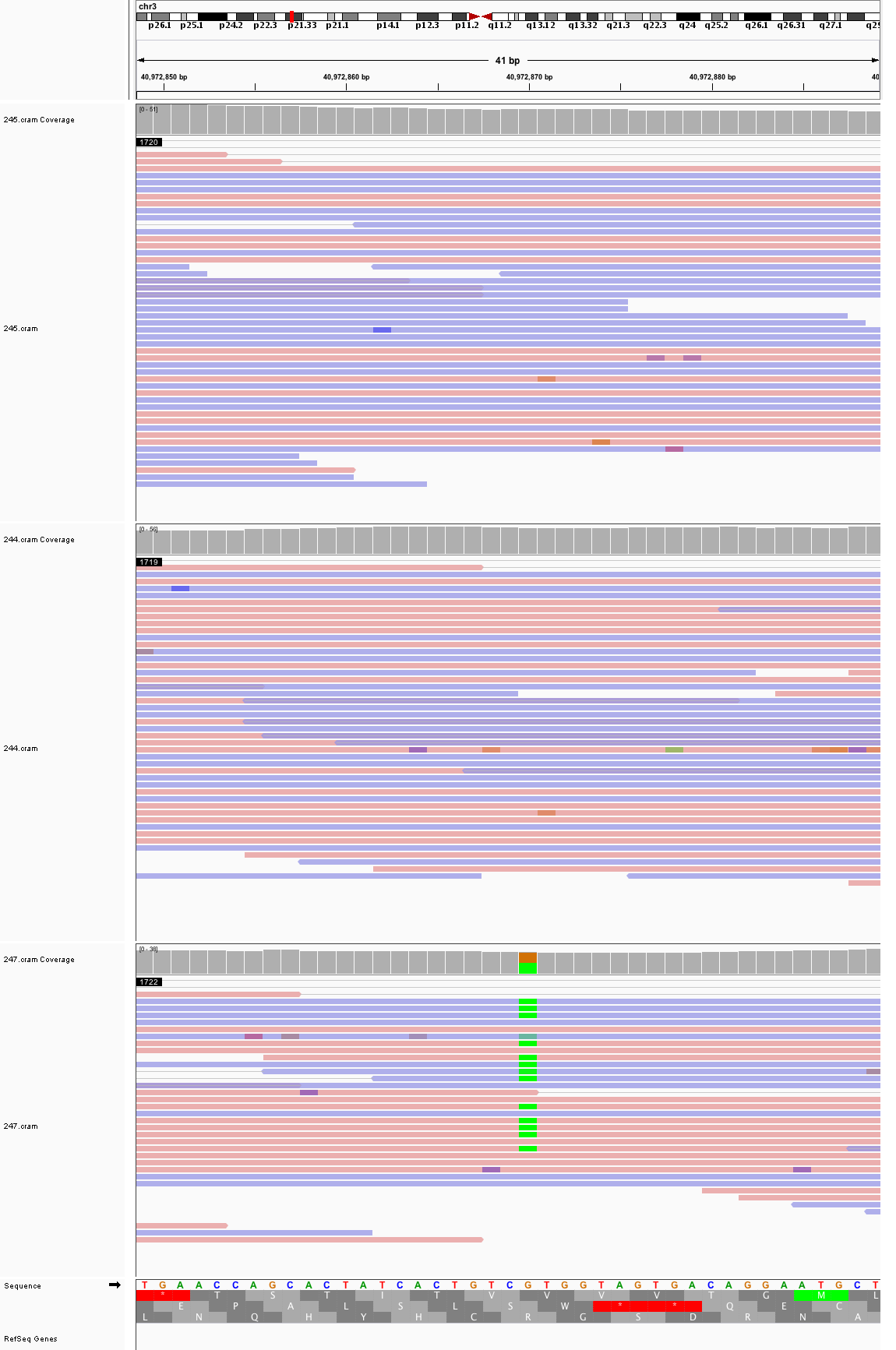

Supplement: Supplementary file 2. — In each image, the first two tracks contain alignments from the first-generation parents, and the third track contains the alignments for the second-generation child. Reads with mapping quality <20 are not included, as they were not considered by our variant calling pipeline, and mismatched bases are shaded by quality score (more transparent = lower base quality). [file elife-46922-supp2.zip › supp_file_2/chr3_40,972,849_40,972,889.png]

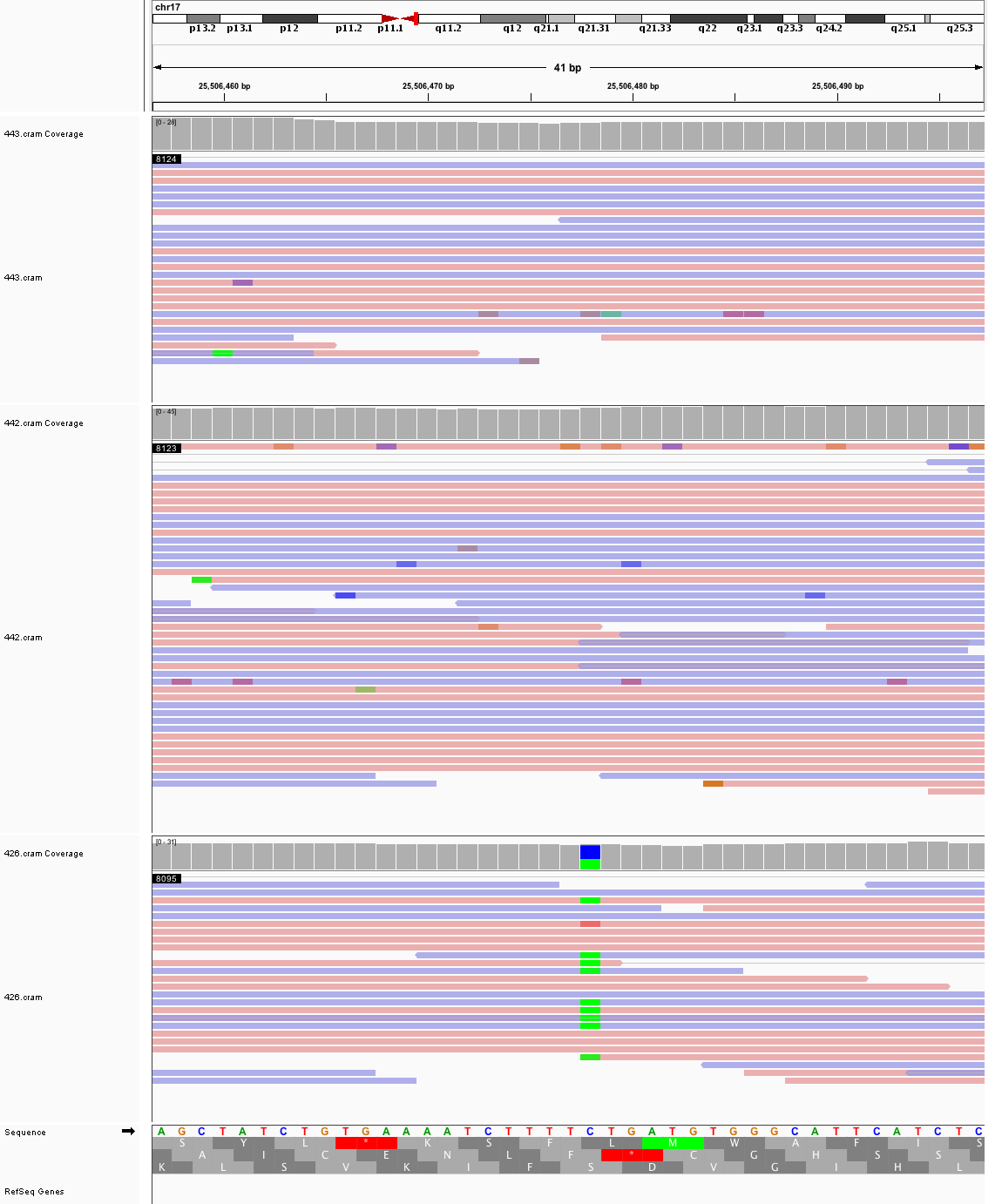

Supplement: Supplementary file 2. — In each image, the first two tracks contain alignments from the first-generation parents, and the third track contains the alignments for the second-generation child. Reads with mapping quality <20 are not included, as they were not considered by our variant calling pipeline, and mismatched bases are shaded by quality score (more transparent = lower base quality). [file elife-46922-supp2.zip › supp_file_2/chr17_25,506,457_25,506,497.png]

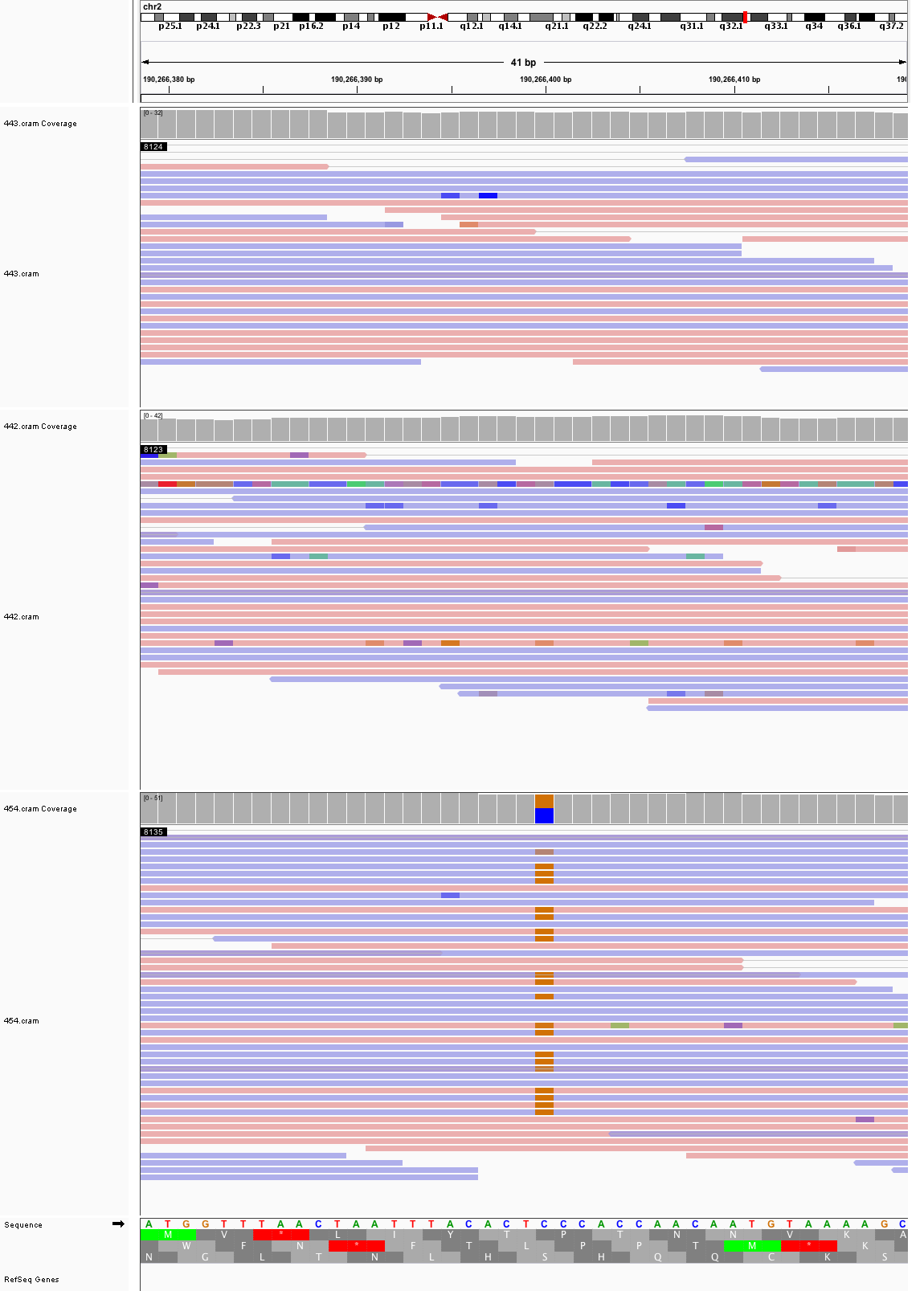

Supplement: Supplementary file 2. — In each image, the first two tracks contain alignments from the first-generation parents, and the third track contains the alignments for the second-generation child. Reads with mapping quality <20 are not included, as they were not considered by our variant calling pipeline, and mismatched bases are shaded by quality score (more transparent = lower base quality). [file elife-46922-supp2.zip › supp_file_2/chr2_190,266,379_190,266,419.png]

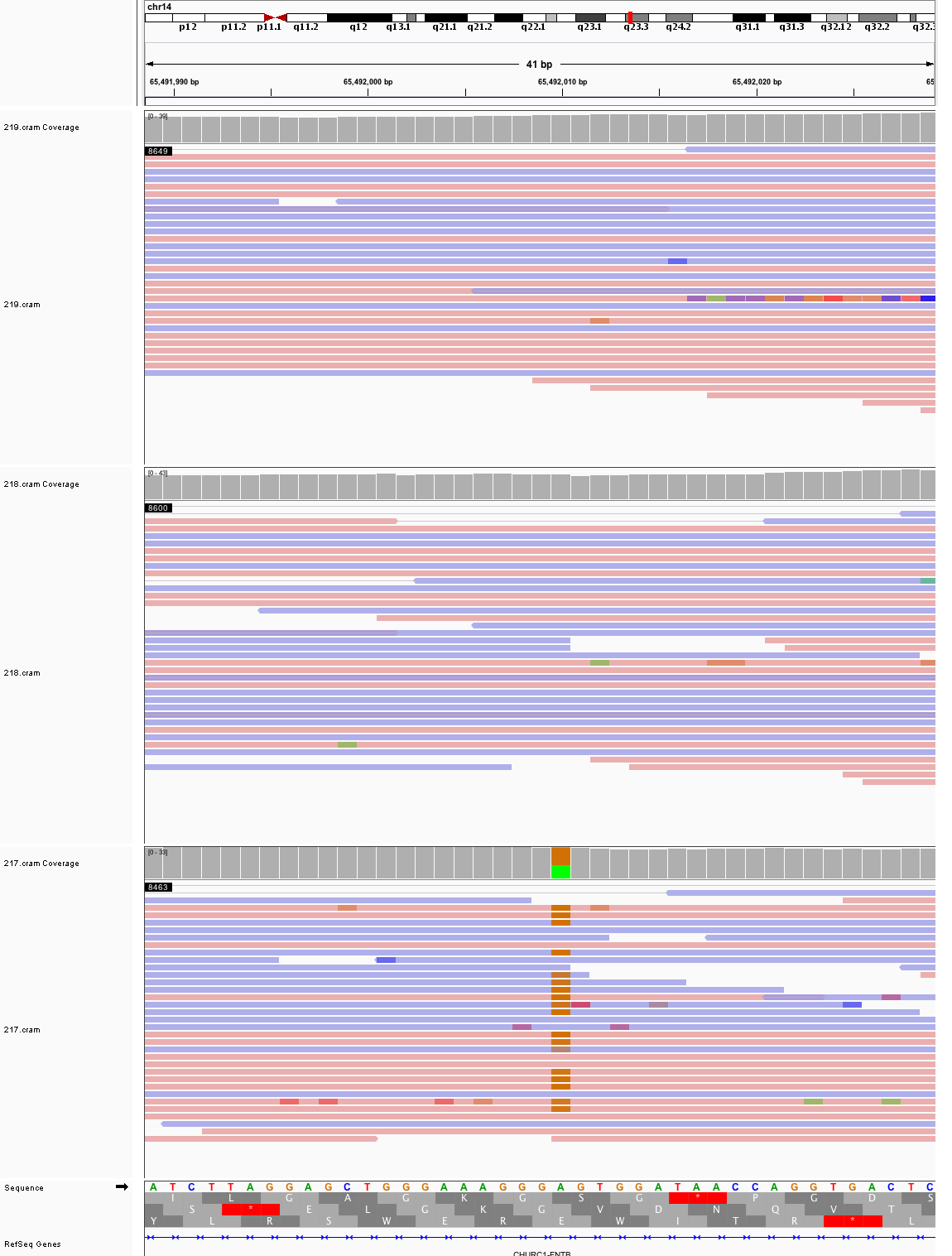

Supplement: Supplementary file 2. — In each image, the first two tracks contain alignments from the first-generation parents, and the third track contains the alignments for the second-generation child. Reads with mapping quality <20 are not included, as they were not considered by our variant calling pipeline, and mismatched bases are shaded by quality score (more transparent = lower base quality). [file elife-46922-supp2.zip › supp_file_2/chr14_65,491,989_65,492,029.png]

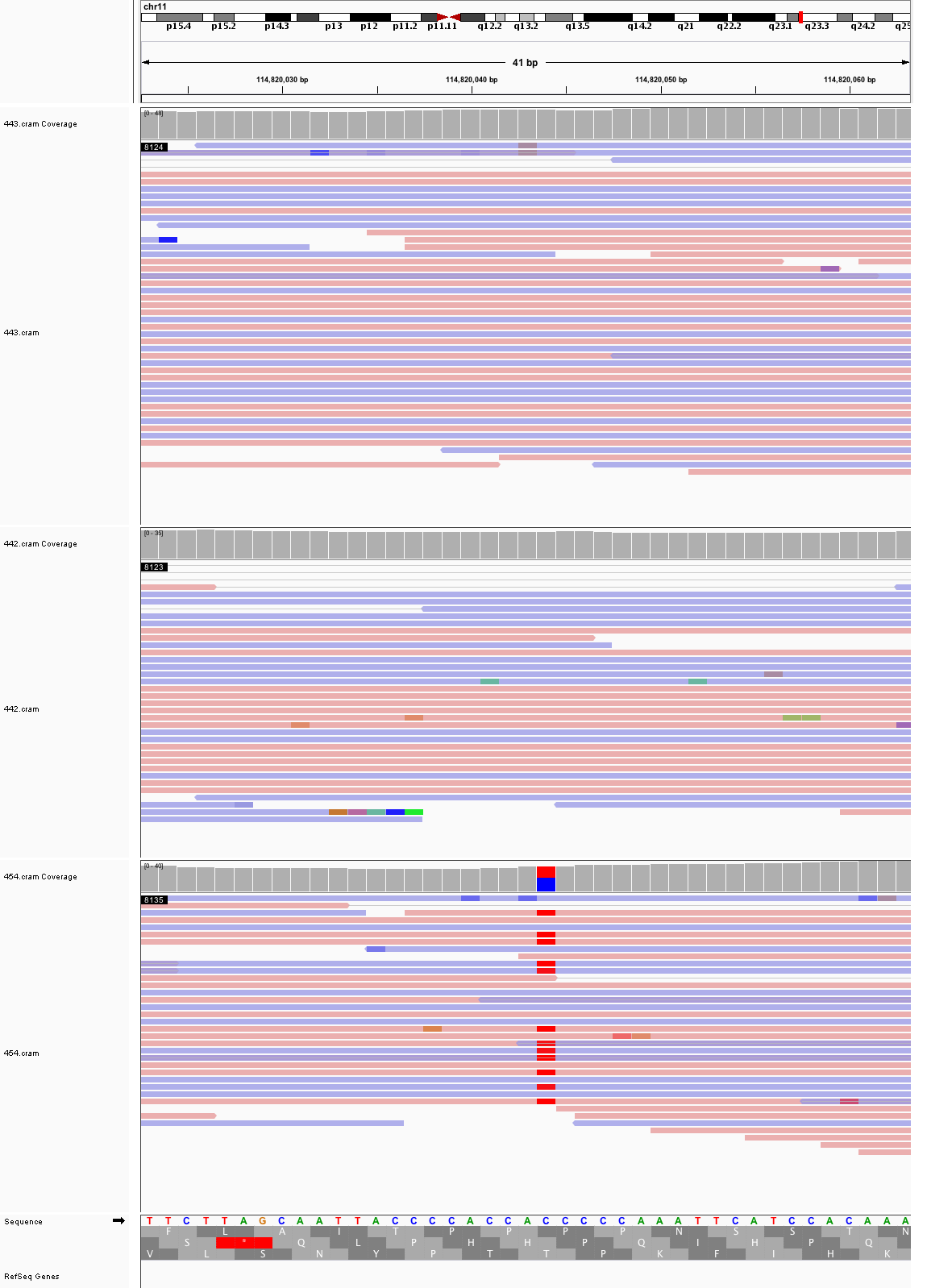

Supplement: Supplementary file 2. — In each image, the first two tracks contain alignments from the first-generation parents, and the third track contains the alignments for the second-generation child. Reads with mapping quality <20 are not included, as they were not considered by our variant calling pipeline, and mismatched bases are shaded by quality score (more transparent = lower base quality). [file elife-46922-supp2.zip › supp_file_2/chr11_114,820,023_114,820,063.png]

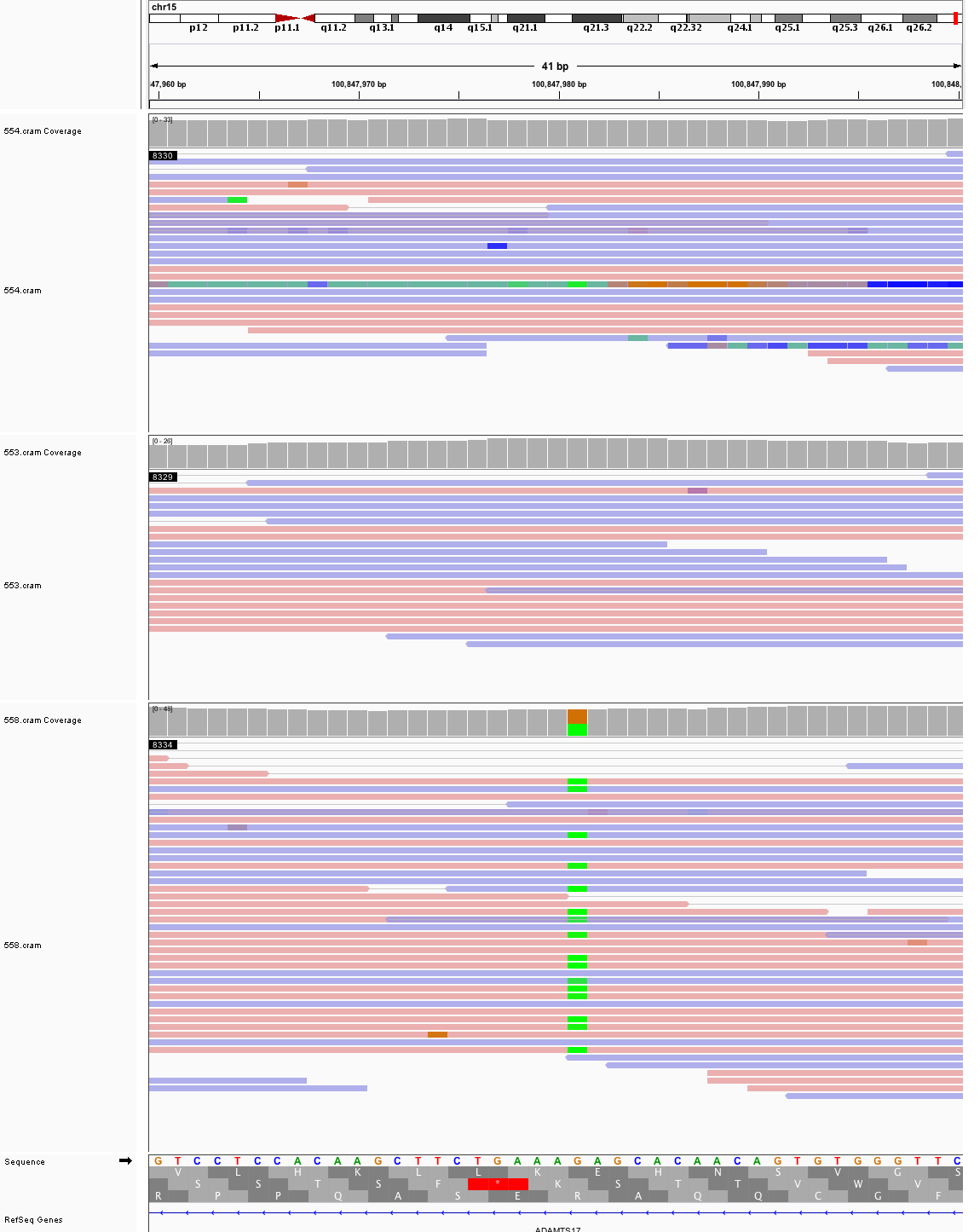

Supplement: Supplementary file 2. — In each image, the first two tracks contain alignments from the first-generation parents, and the third track contains the alignments for the second-generation child. Reads with mapping quality <20 are not included, as they were not considered by our variant calling pipeline, and mismatched bases are shaded by quality score (more transparent = lower base quality). [file elife-46922-supp2.zip › supp_file_2/chr15_100,847,960_100,848,000.png]

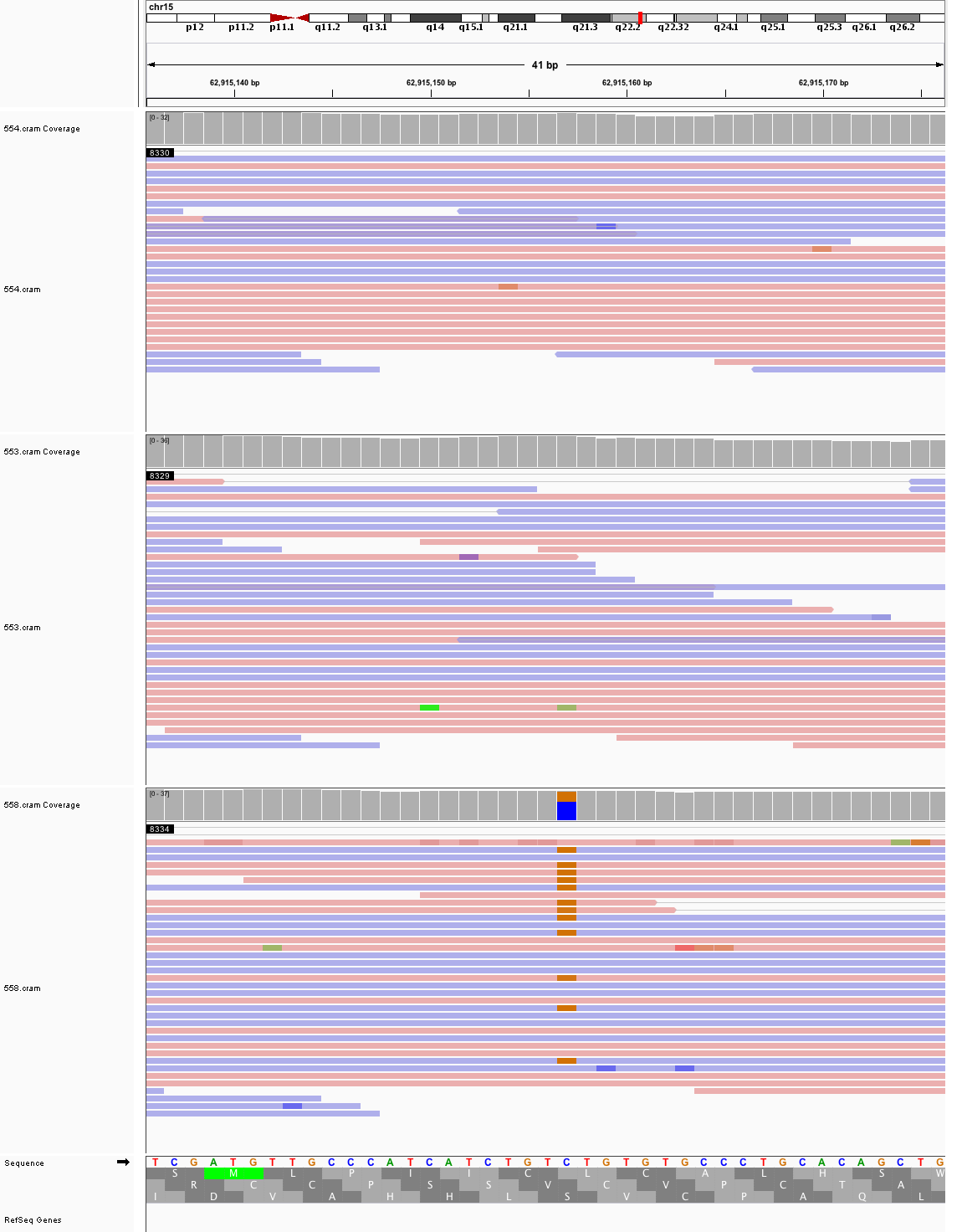

Supplement: Supplementary file 2. — In each image, the first two tracks contain alignments from the first-generation parents, and the third track contains the alignments for the second-generation child. Reads with mapping quality <20 are not included, as they were not considered by our variant calling pipeline, and mismatched bases are shaded by quality score (more transparent = lower base quality). [file elife-46922-supp2.zip › supp_file_2/chr15_62,915,136_62,915,176.png]

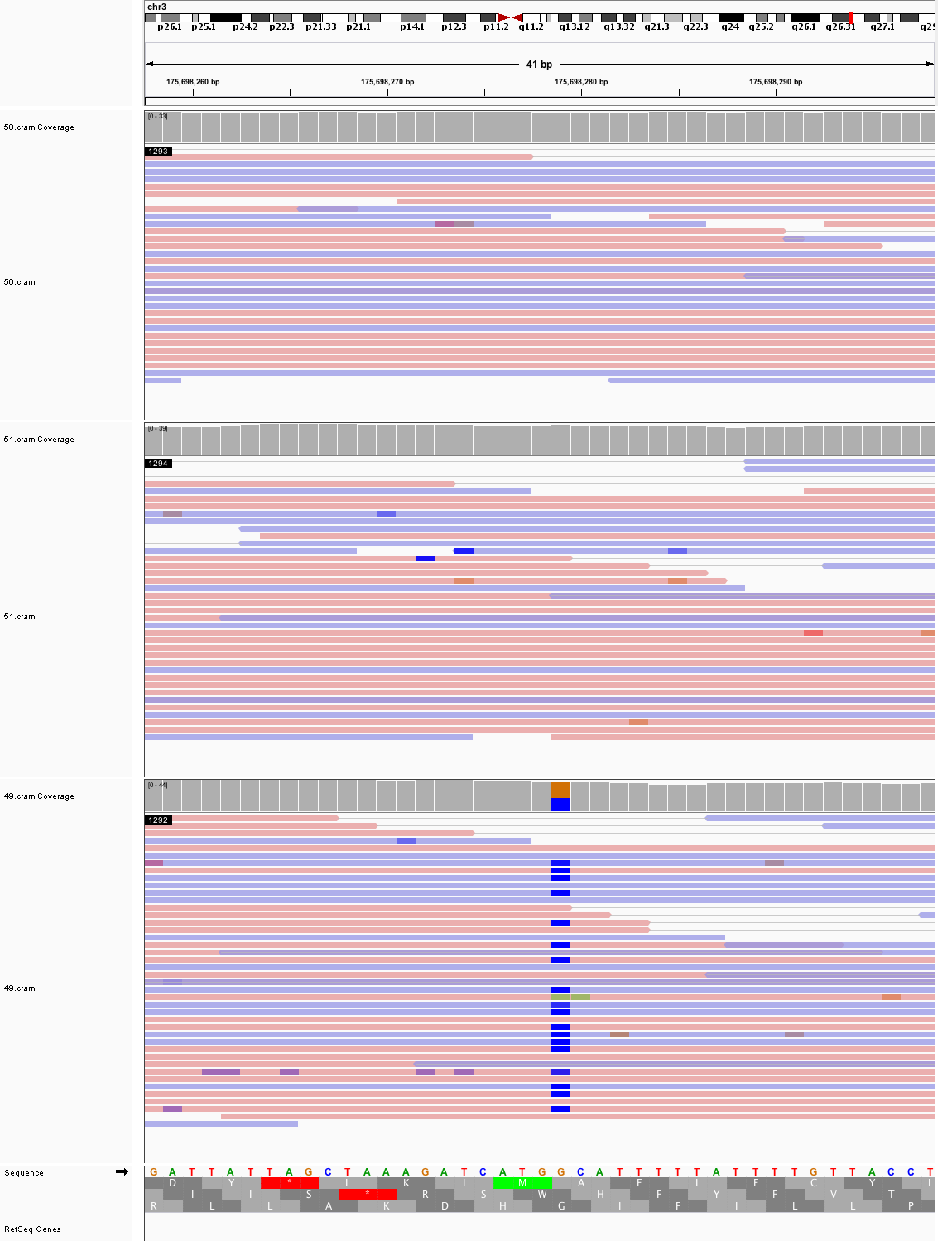

Supplement: Supplementary file 2. — In each image, the first two tracks contain alignments from the first-generation parents, and the third track contains the alignments for the second-generation child. Reads with mapping quality <20 are not included, as they were not considered by our variant calling pipeline, and mismatched bases are shaded by quality score (more transparent = lower base quality). [file elife-46922-supp2.zip › supp_file_2/chr3_175,698,258_175,698,298.png]

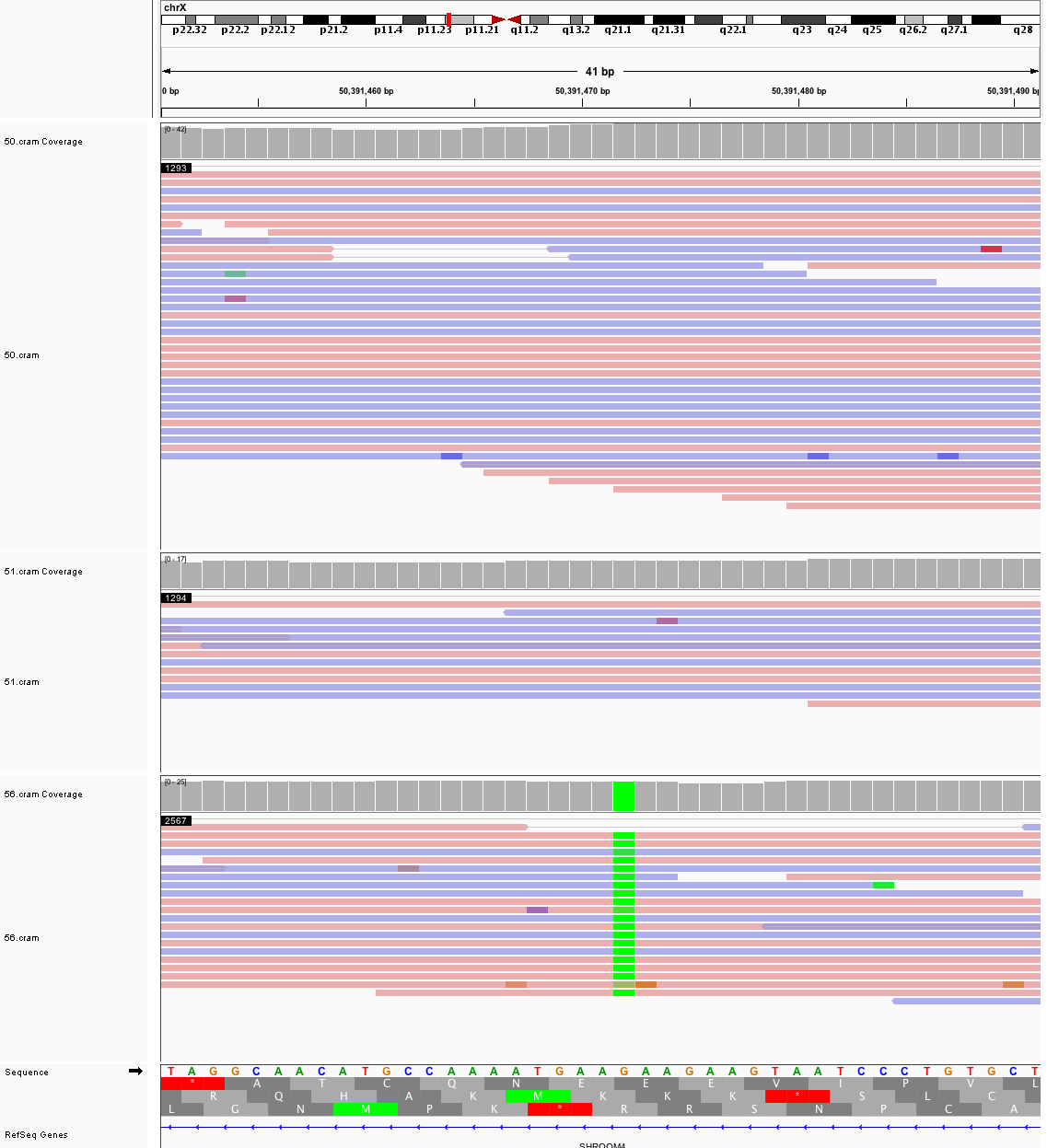

Supplement: Supplementary file 2. — In each image, the first two tracks contain alignments from the first-generation parents, and the third track contains the alignments for the second-generation child. Reads with mapping quality <20 are not included, as they were not considered by our variant calling pipeline, and mismatched bases are shaded by quality score (more transparent = lower base quality). [file elife-46922-supp2.zip › supp_file_2/chrX_50,391,451_50,391,491.png]

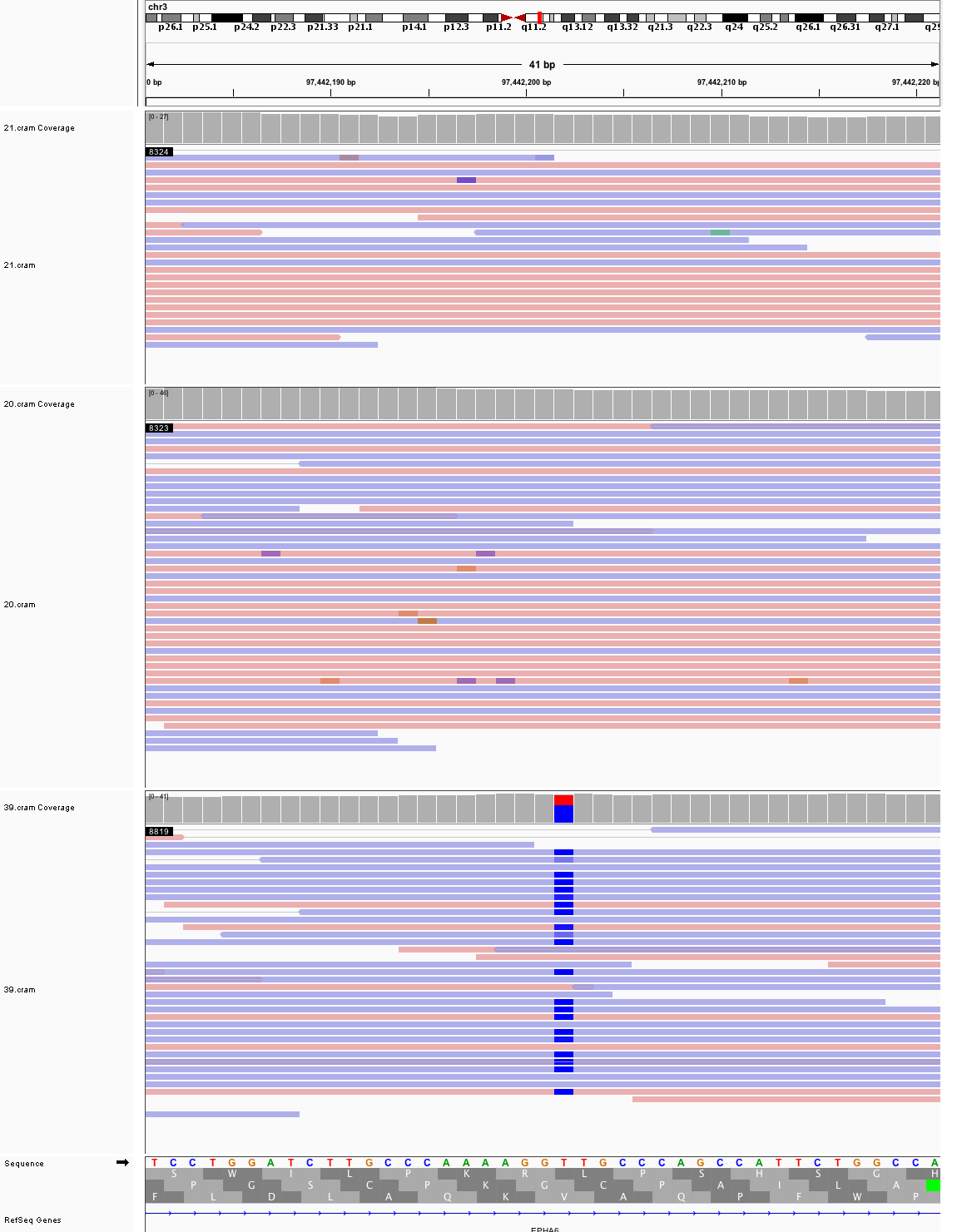

Supplement: Supplementary file 2. — In each image, the first two tracks contain alignments from the first-generation parents, and the third track contains the alignments for the second-generation child. Reads with mapping quality <20 are not included, as they were not considered by our variant calling pipeline, and mismatched bases are shaded by quality score (more transparent = lower base quality). [file elife-46922-supp2.zip › supp_file_2/chr3_97,442,181_97,442,221.png]

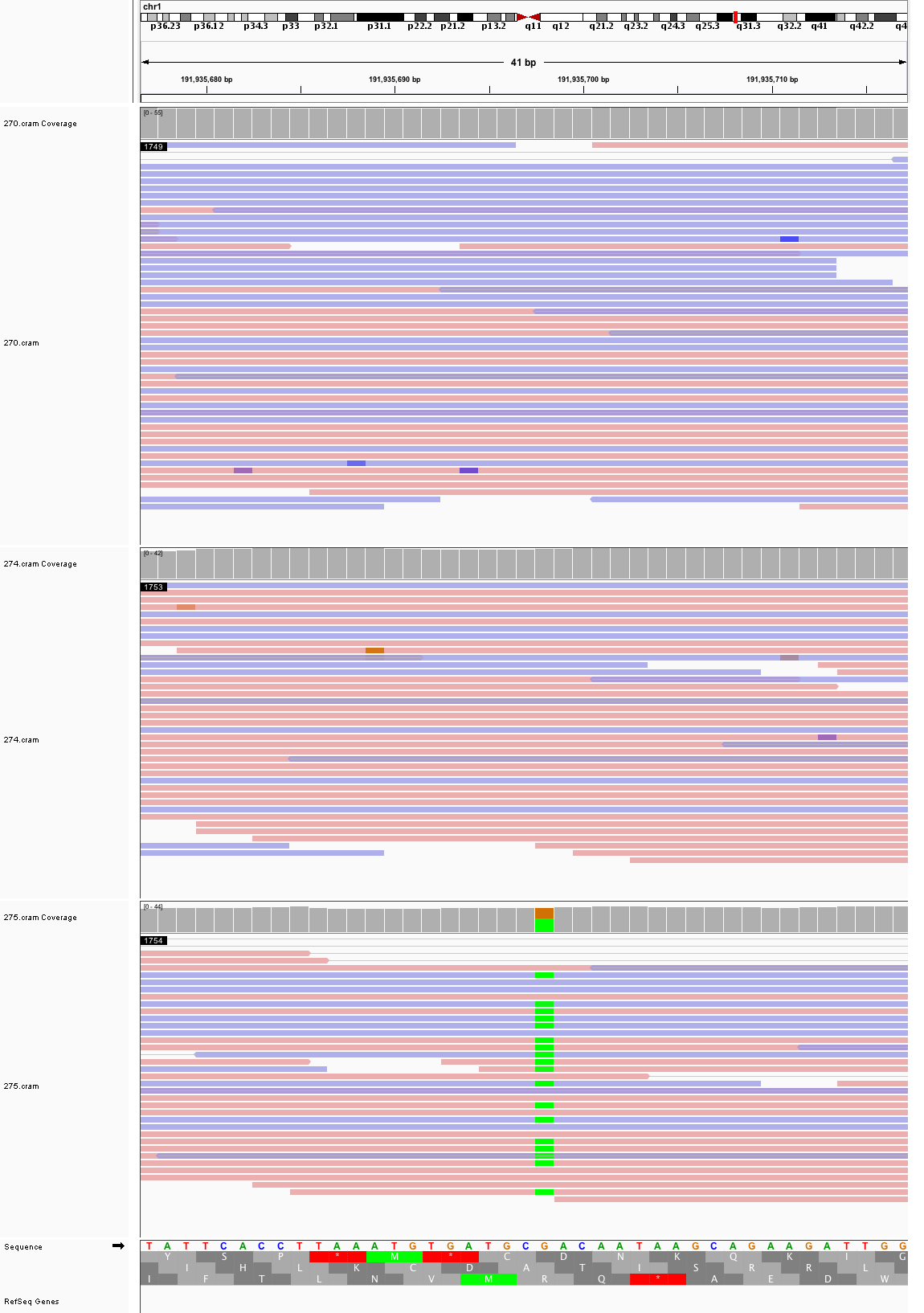

Supplement: Supplementary file 2. — In each image, the first two tracks contain alignments from the first-generation parents, and the third track contains the alignments for the second-generation child. Reads with mapping quality <20 are not included, as they were not considered by our variant calling pipeline, and mismatched bases are shaded by quality score (more transparent = lower base quality). [file elife-46922-supp2.zip › supp_file_2/chr1_191,935,677_191,935,717.png]

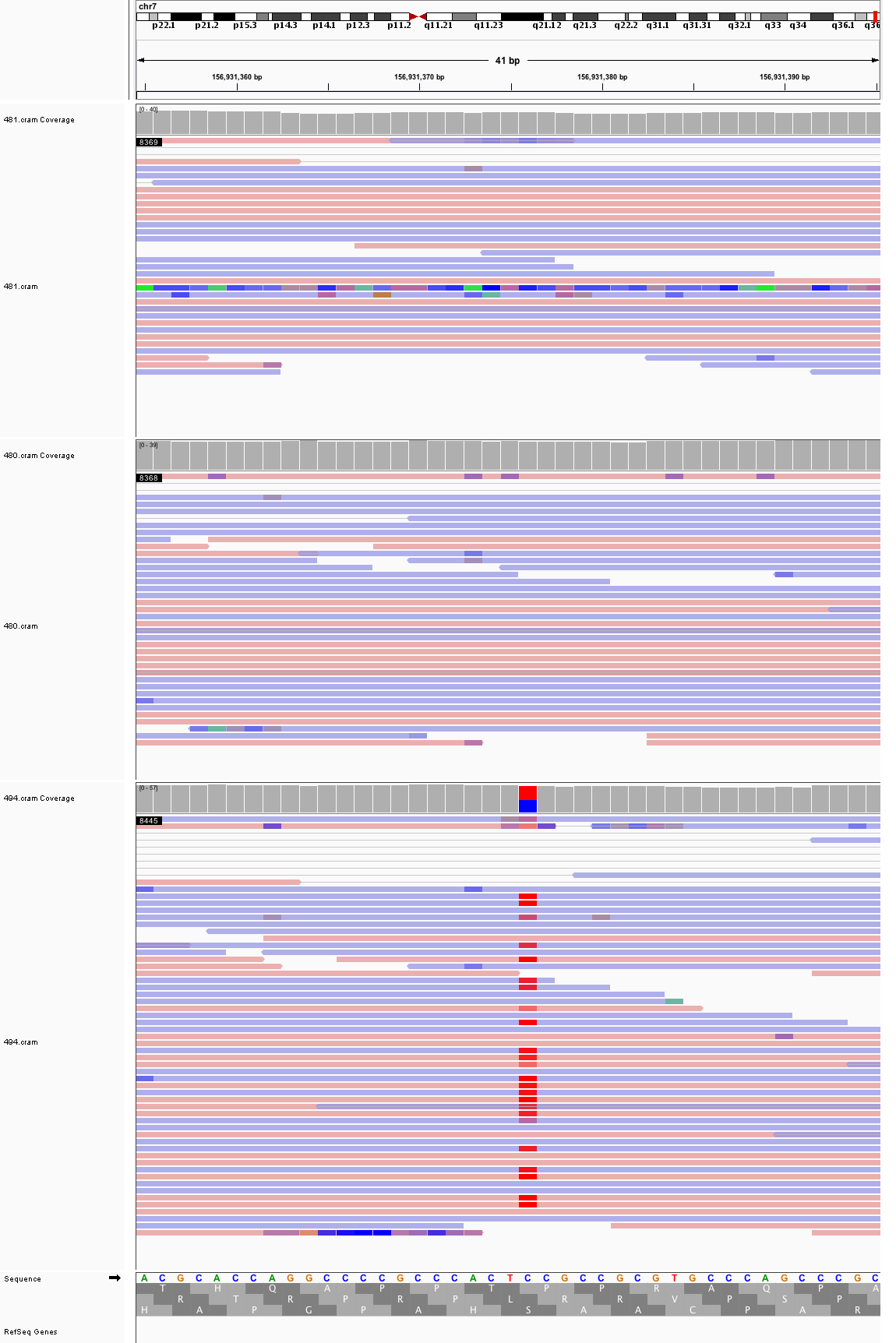

Supplement: Supplementary file 2. — In each image, the first two tracks contain alignments from the first-generation parents, and the third track contains the alignments for the second-generation child. Reads with mapping quality <20 are not included, as they were not considered by our variant calling pipeline, and mismatched bases are shaded by quality score (more transparent = lower base quality). [file elife-46922-supp2.zip › supp_file_2/chr7_156,931,355_156,931,395.png]

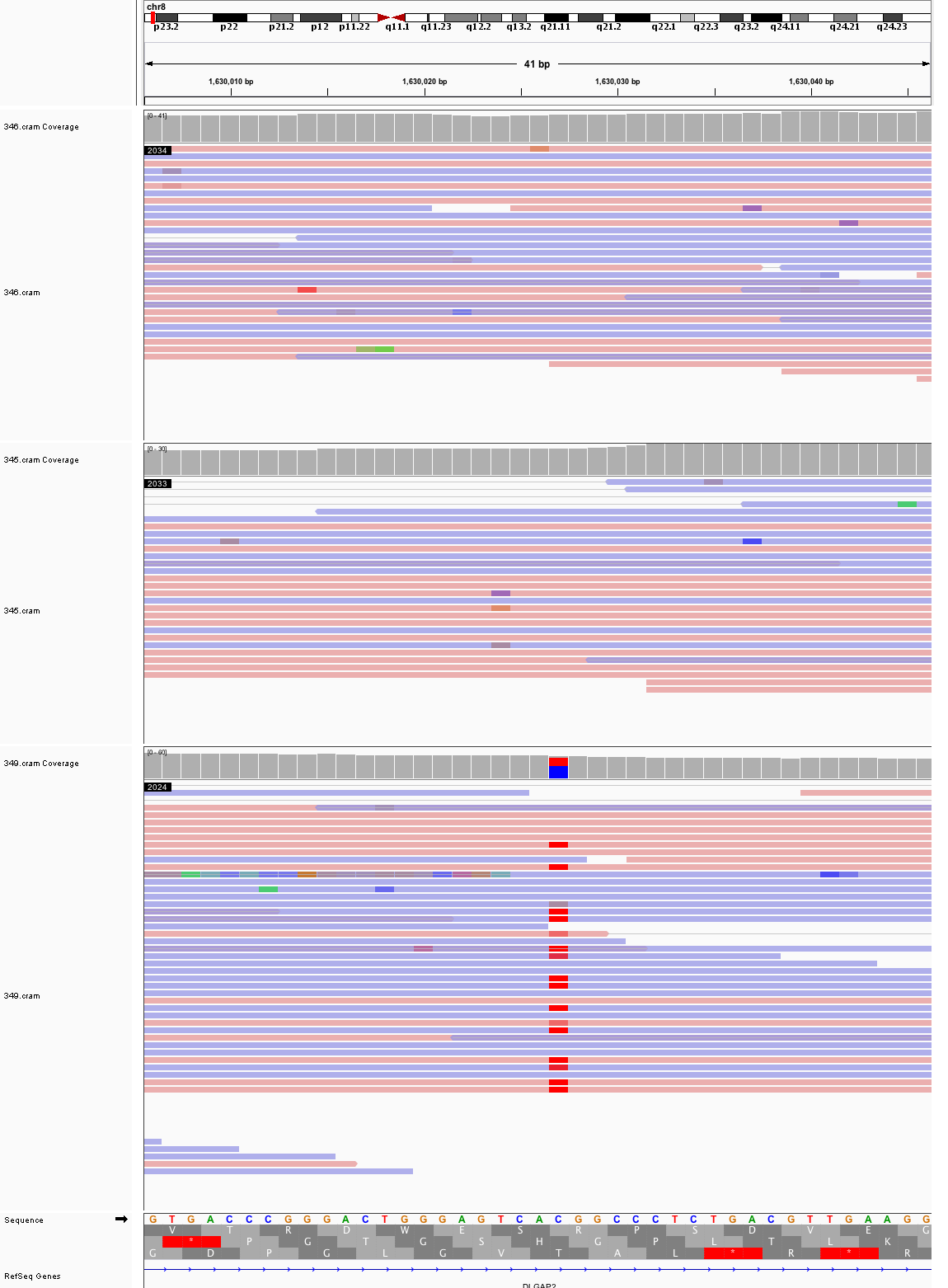

Supplement: Supplementary file 2. — In each image, the first two tracks contain alignments from the first-generation parents, and the third track contains the alignments for the second-generation child. Reads with mapping quality <20 are not included, as they were not considered by our variant calling pipeline, and mismatched bases are shaded by quality score (more transparent = lower base quality). [file elife-46922-supp2.zip › supp_file_2/chr8_1,630,006_1,630,046.png]

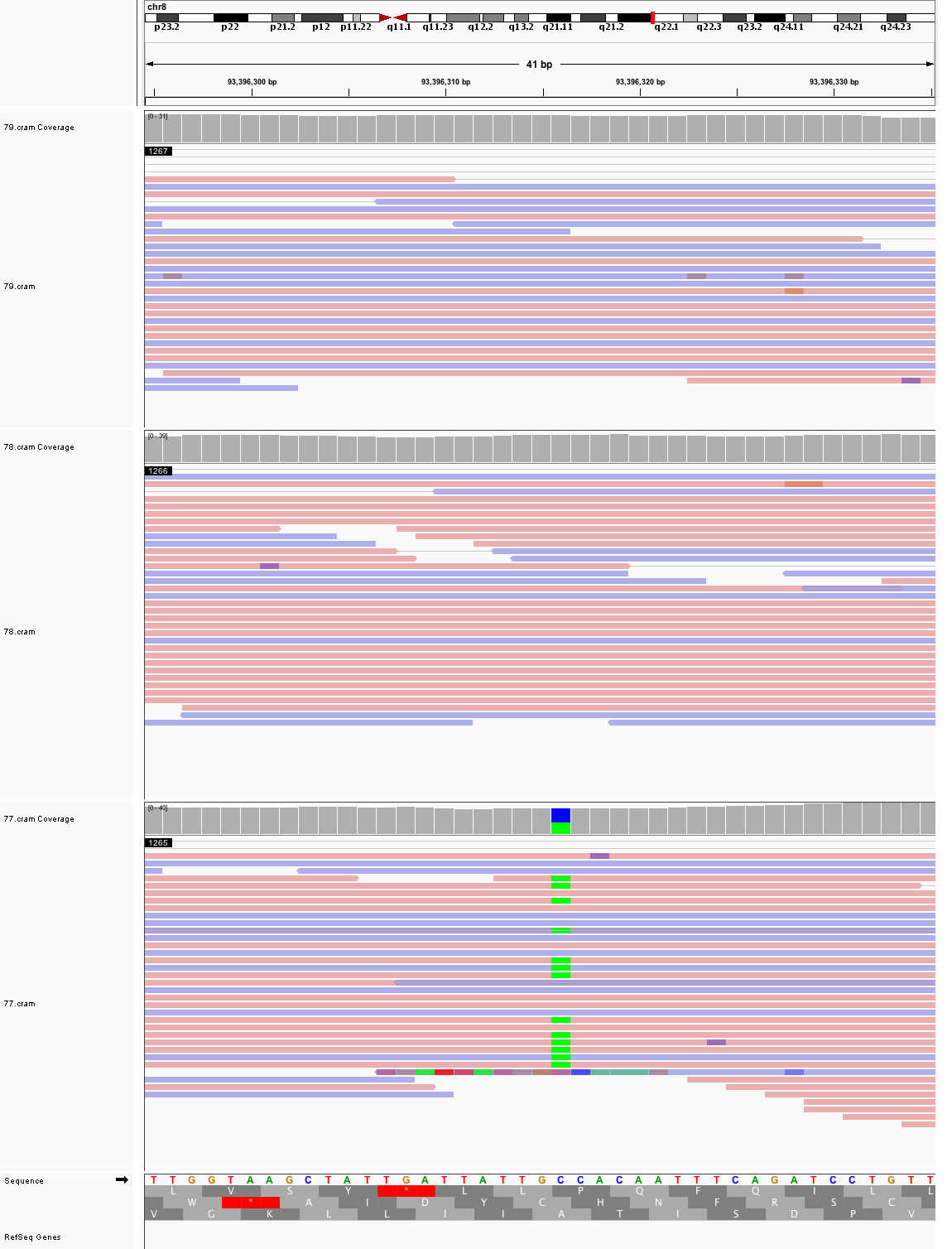

Supplement: Supplementary file 2. — In each image, the first two tracks contain alignments from the first-generation parents, and the third track contains the alignments for the second-generation child. Reads with mapping quality <20 are not included, as they were not considered by our variant calling pipeline, and mismatched bases are shaded by quality score (more transparent = lower base quality). [file elife-46922-supp2.zip › supp_file_2/chr8_93,396,295_93,396,335.png]

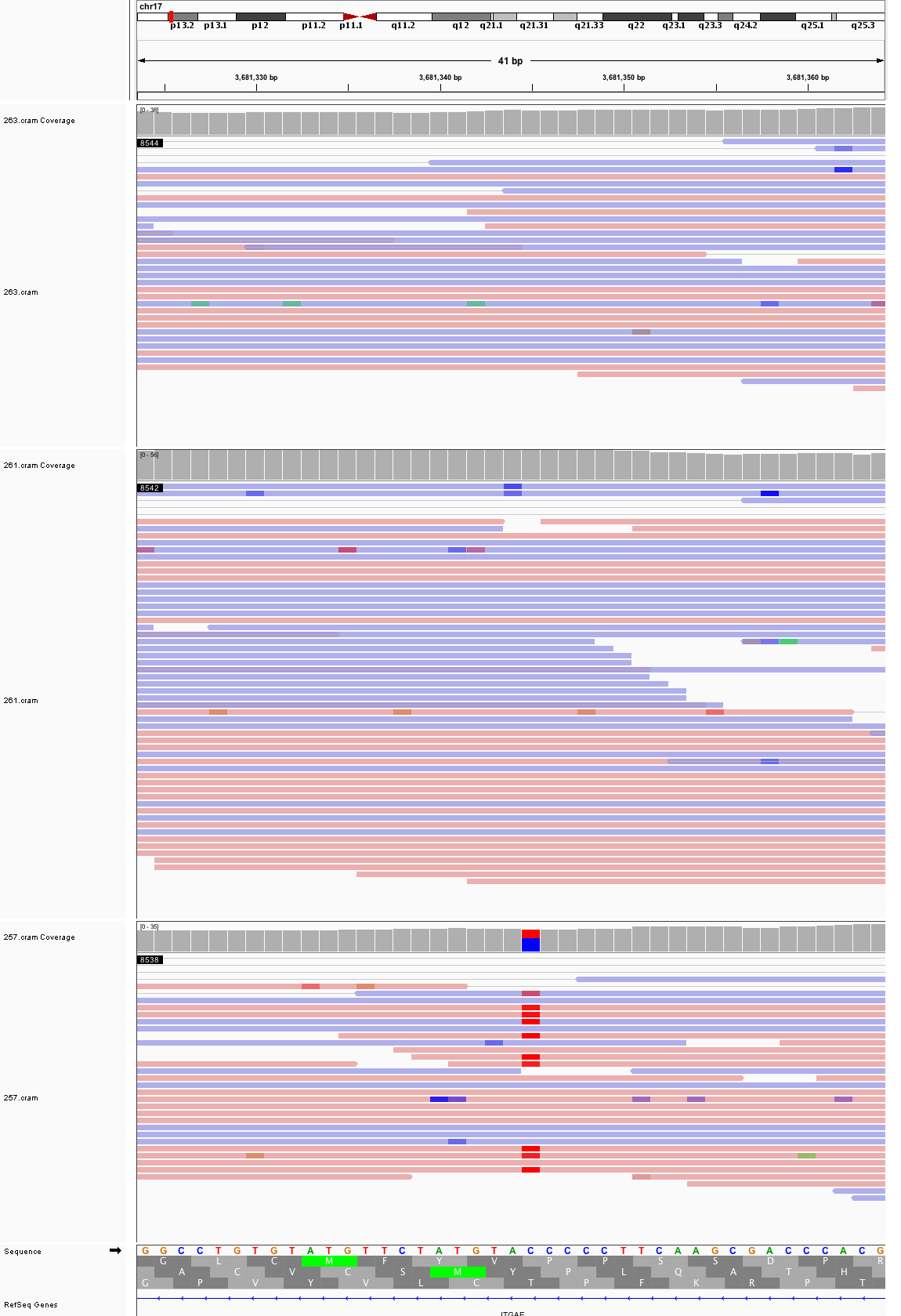

Supplement: Supplementary file 2. — In each image, the first two tracks contain alignments from the first-generation parents, and the third track contains the alignments for the second-generation child. Reads with mapping quality <20 are not included, as they were not considered by our variant calling pipeline, and mismatched bases are shaded by quality score (more transparent = lower base quality). [file elife-46922-supp2.zip › supp_file_2/chr17_3,681,324_3,681,364.png]

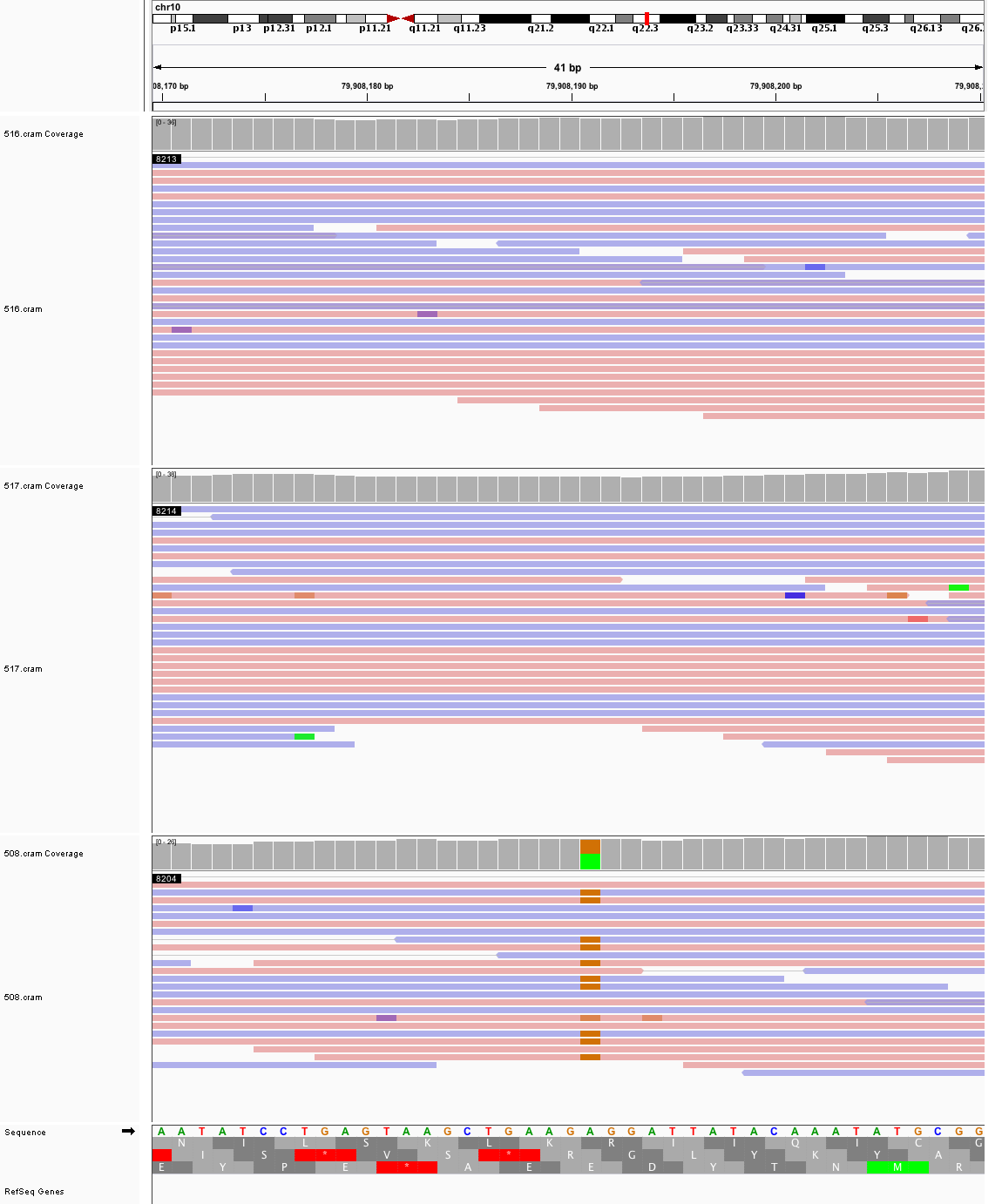

Supplement: Supplementary file 2. — In each image, the first two tracks contain alignments from the first-generation parents, and the third track contains the alignments for the second-generation child. Reads with mapping quality <20 are not included, as they were not considered by our variant calling pipeline, and mismatched bases are shaded by quality score (more transparent = lower base quality). [file elife-46922-supp2.zip › supp_file_2/chr10_79,908,170_79,908,210.png]

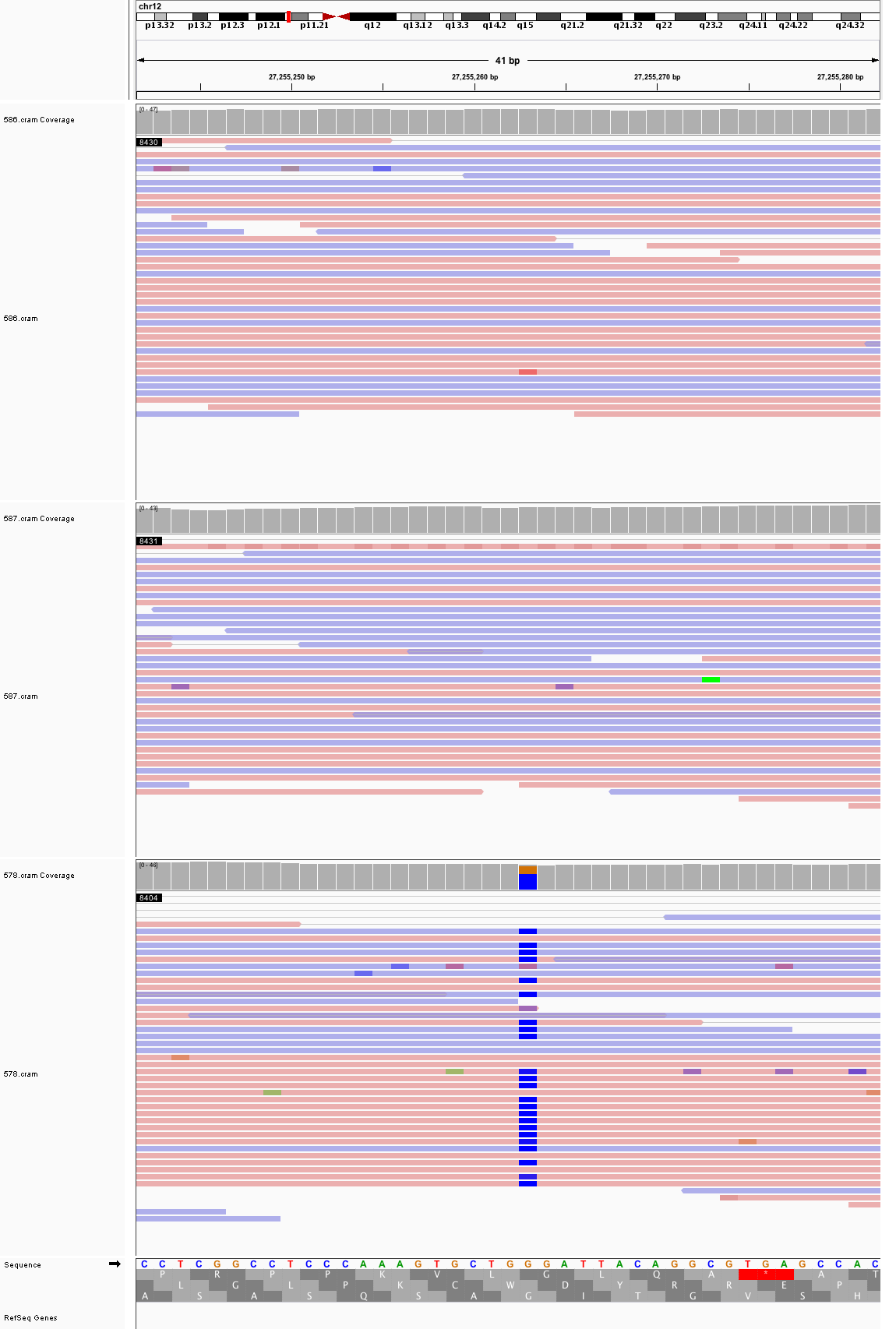

Supplement: Supplementary file 2. — In each image, the first two tracks contain alignments from the first-generation parents, and the third track contains the alignments for the second-generation child. Reads with mapping quality <20 are not included, as they were not considered by our variant calling pipeline, and mismatched bases are shaded by quality score (more transparent = lower base quality). [file elife-46922-supp2.zip › supp_file_2/chr12_27,255,242_27,255,282.png]

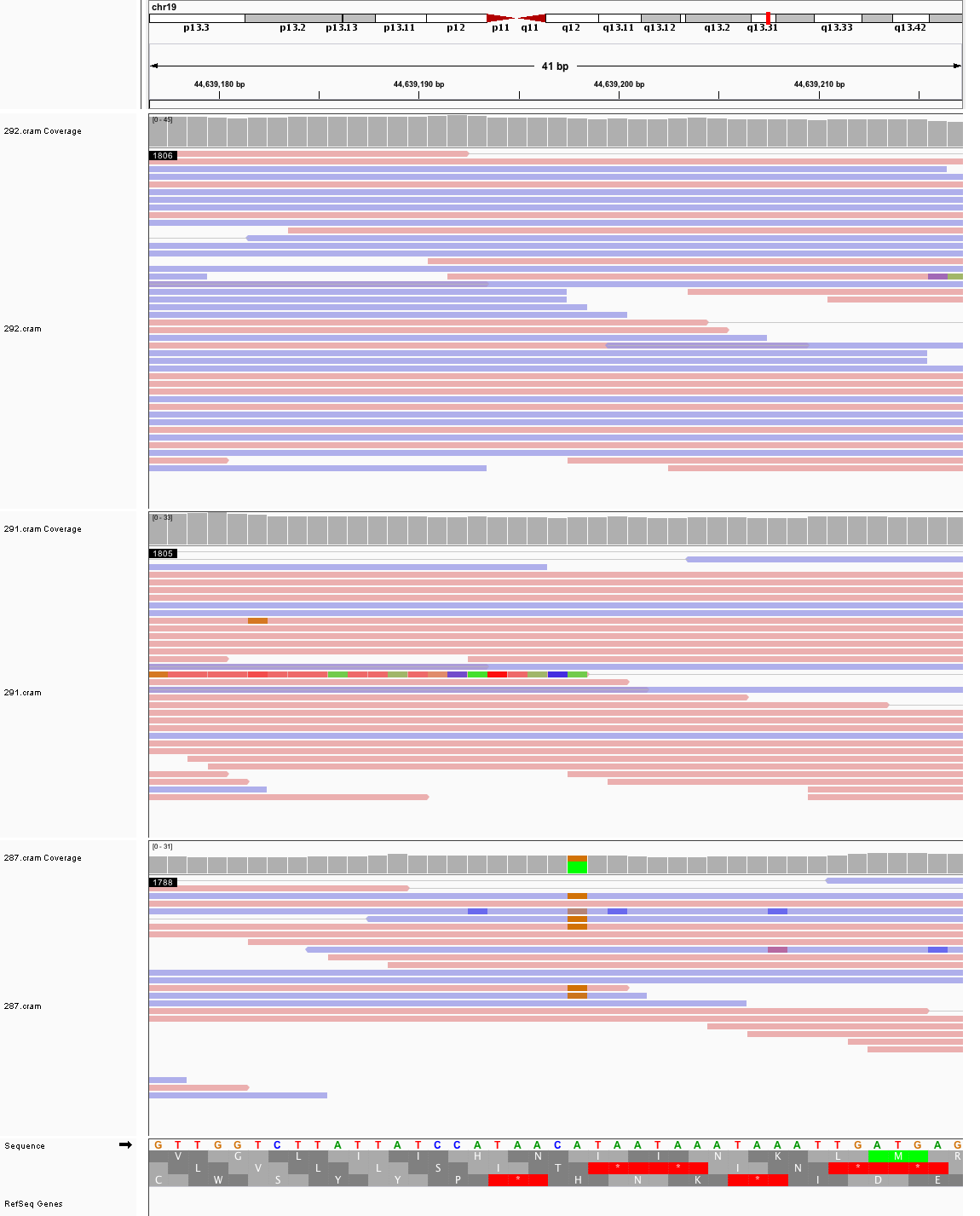

Supplement: Supplementary file 2. — In each image, the first two tracks contain alignments from the first-generation parents, and the third track contains the alignments for the second-generation child. Reads with mapping quality <20 are not included, as they were not considered by our variant calling pipeline, and mismatched bases are shaded by quality score (more transparent = lower base quality). [file elife-46922-supp2.zip › supp_file_2/chr19_44,639,177_44,639,217.png]

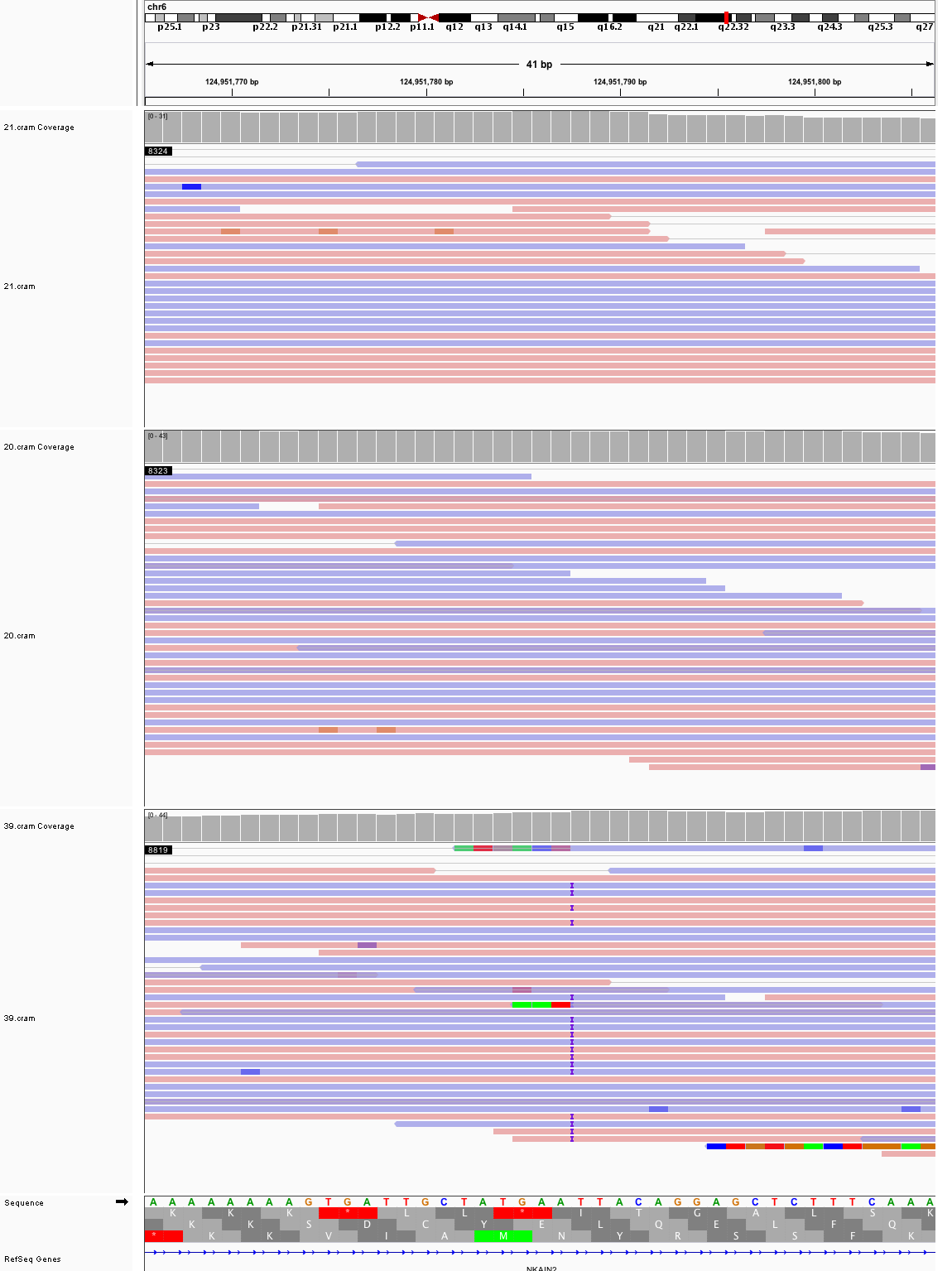

Supplement: Supplementary file 2. — In each image, the first two tracks contain alignments from the first-generation parents, and the third track contains the alignments for the second-generation child. Reads with mapping quality <20 are not included, as they were not considered by our variant calling pipeline, and mismatched bases are shaded by quality score (more transparent = lower base quality). [file elife-46922-supp2.zip › supp_file_2/chr6_124,951,766_124,951,806.png]

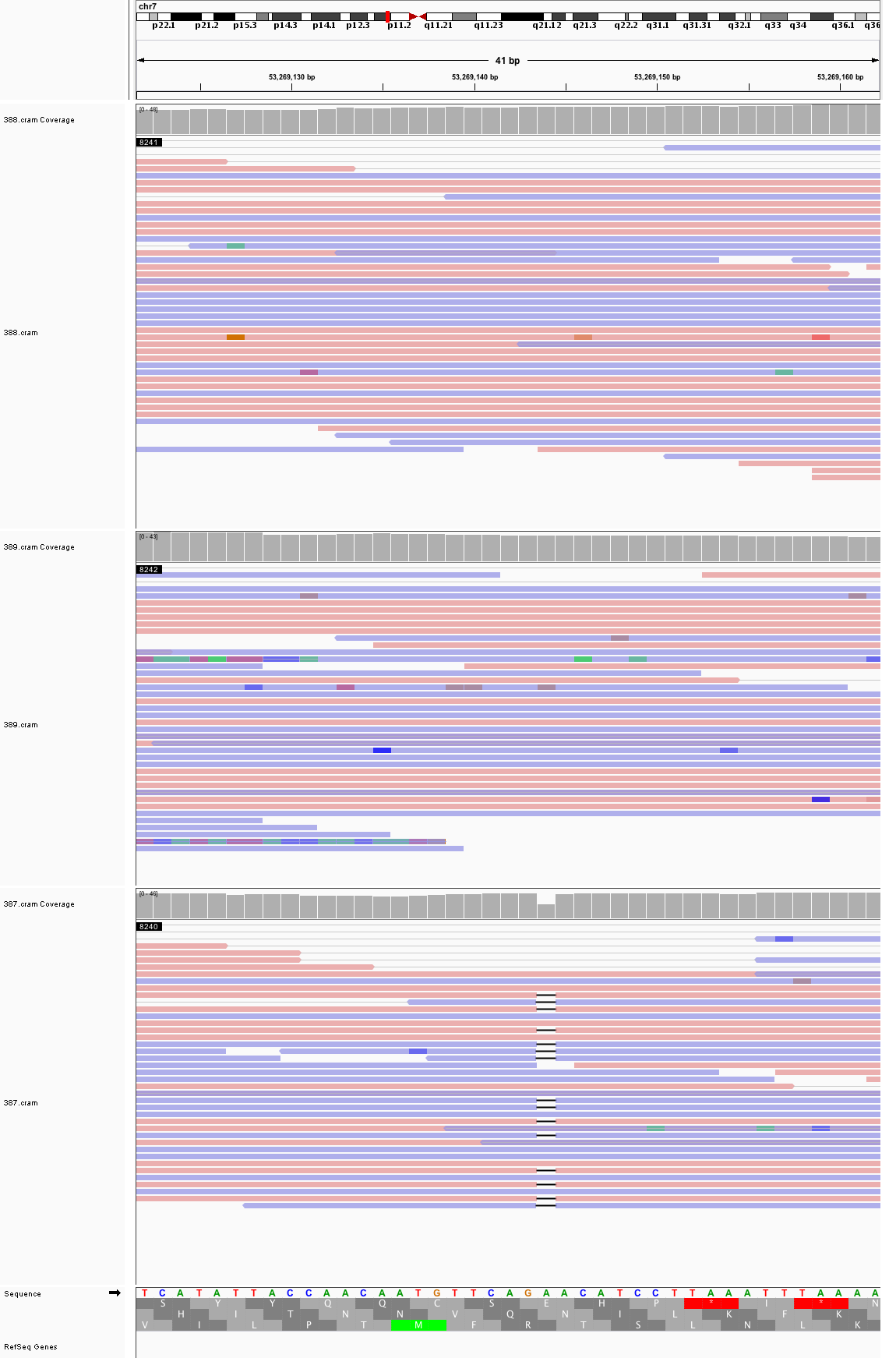

Supplement: Supplementary file 2. — In each image, the first two tracks contain alignments from the first-generation parents, and the third track contains the alignments for the second-generation child. Reads with mapping quality <20 are not included, as they were not considered by our variant calling pipeline, and mismatched bases are shaded by quality score (more transparent = lower base quality). [file elife-46922-supp2.zip › supp_file_2/chr7_53,269,122_53,269,162.png]

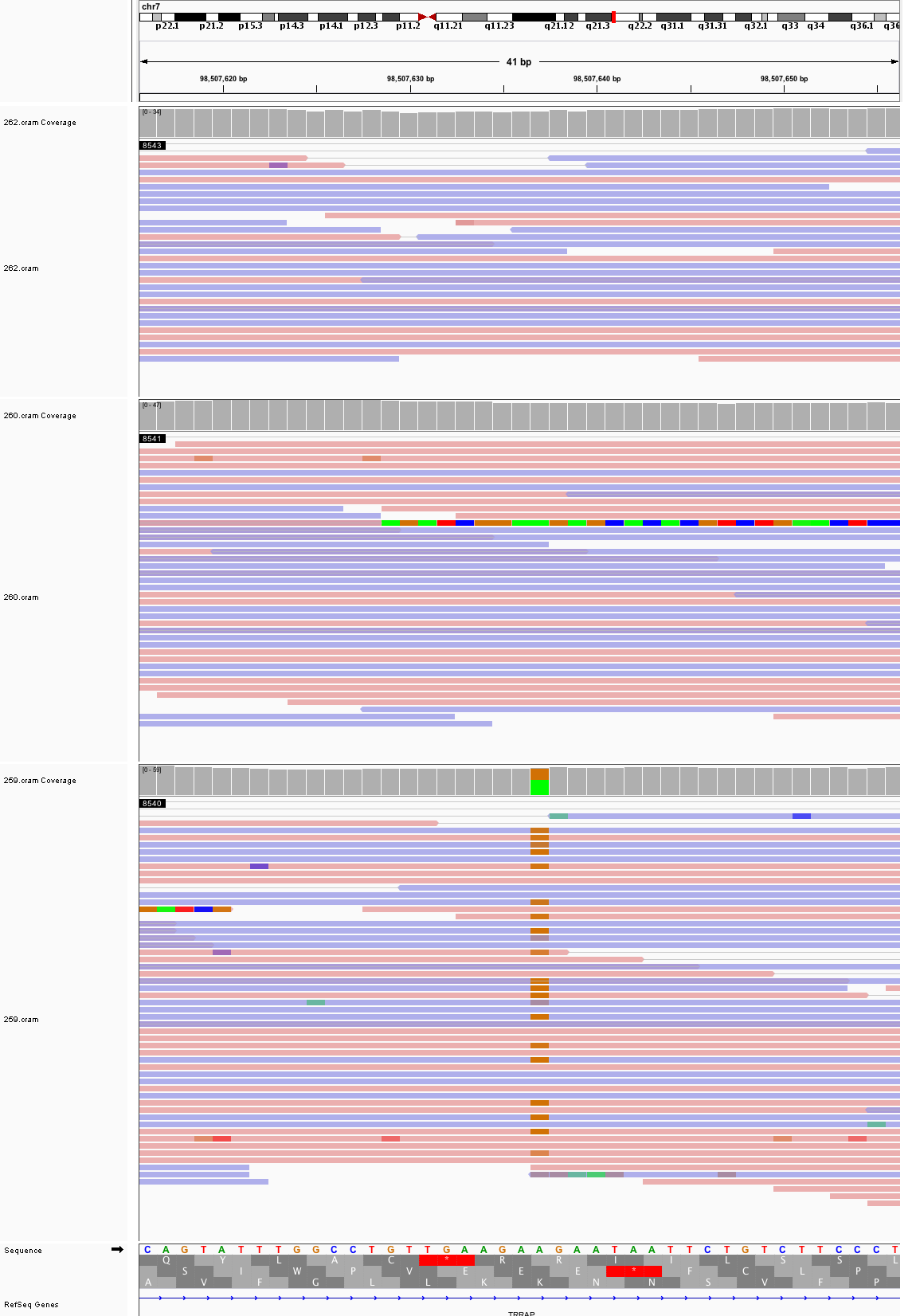

Supplement: Supplementary file 2. — In each image, the first two tracks contain alignments from the first-generation parents, and the third track contains the alignments for the second-generation child. Reads with mapping quality <20 are not included, as they were not considered by our variant calling pipeline, and mismatched bases are shaded by quality score (more transparent = lower base quality). [file elife-46922-supp2.zip › supp_file_2/chr7_98,507,616_98,507,656.png]

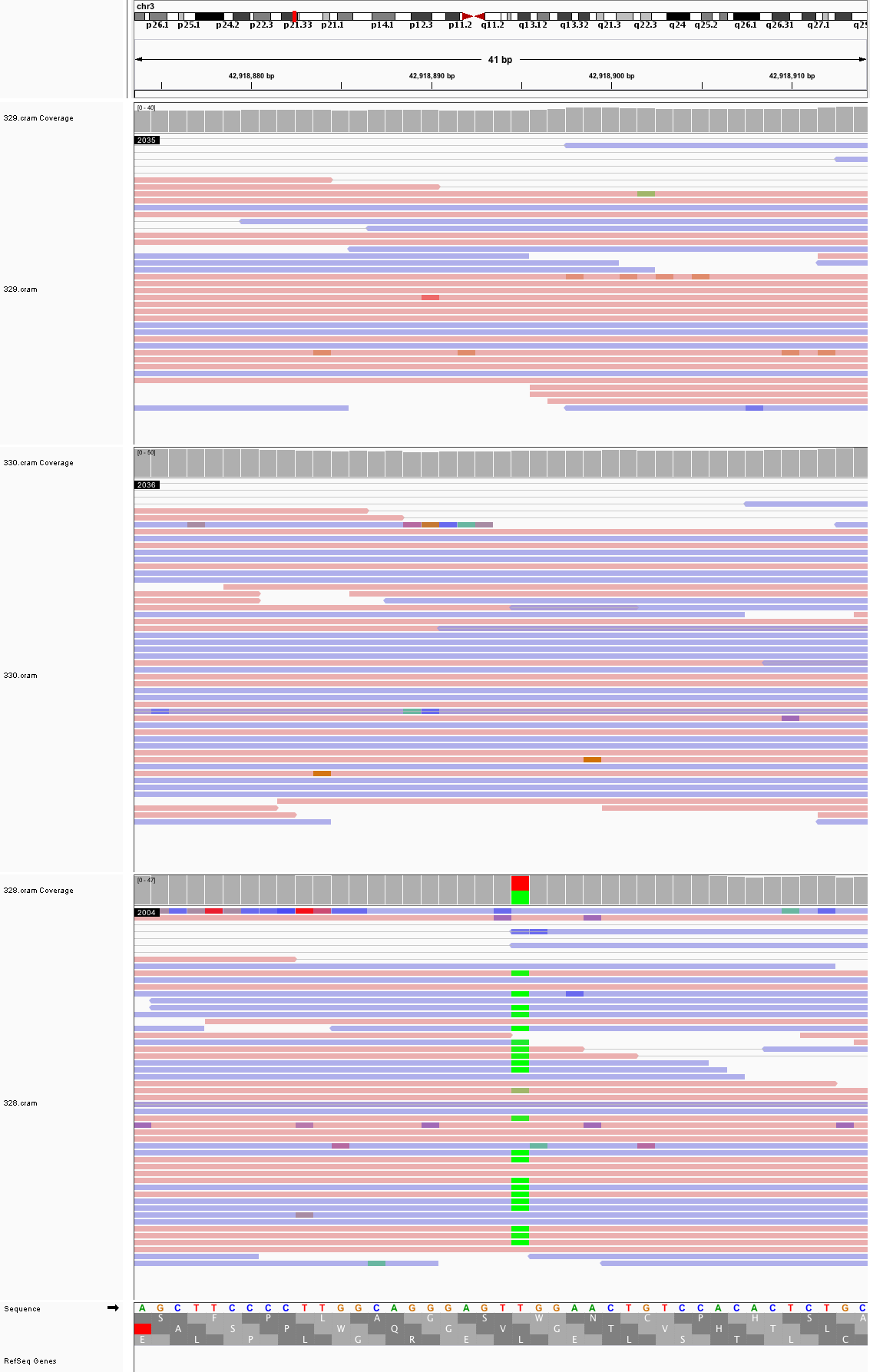

Supplement: Supplementary file 2. — In each image, the first two tracks contain alignments from the first-generation parents, and the third track contains the alignments for the second-generation child. Reads with mapping quality <20 are not included, as they were not considered by our variant calling pipeline, and mismatched bases are shaded by quality score (more transparent = lower base quality). [file elife-46922-supp2.zip › supp_file_2/chr3_42,918,874_42,918,914.png]

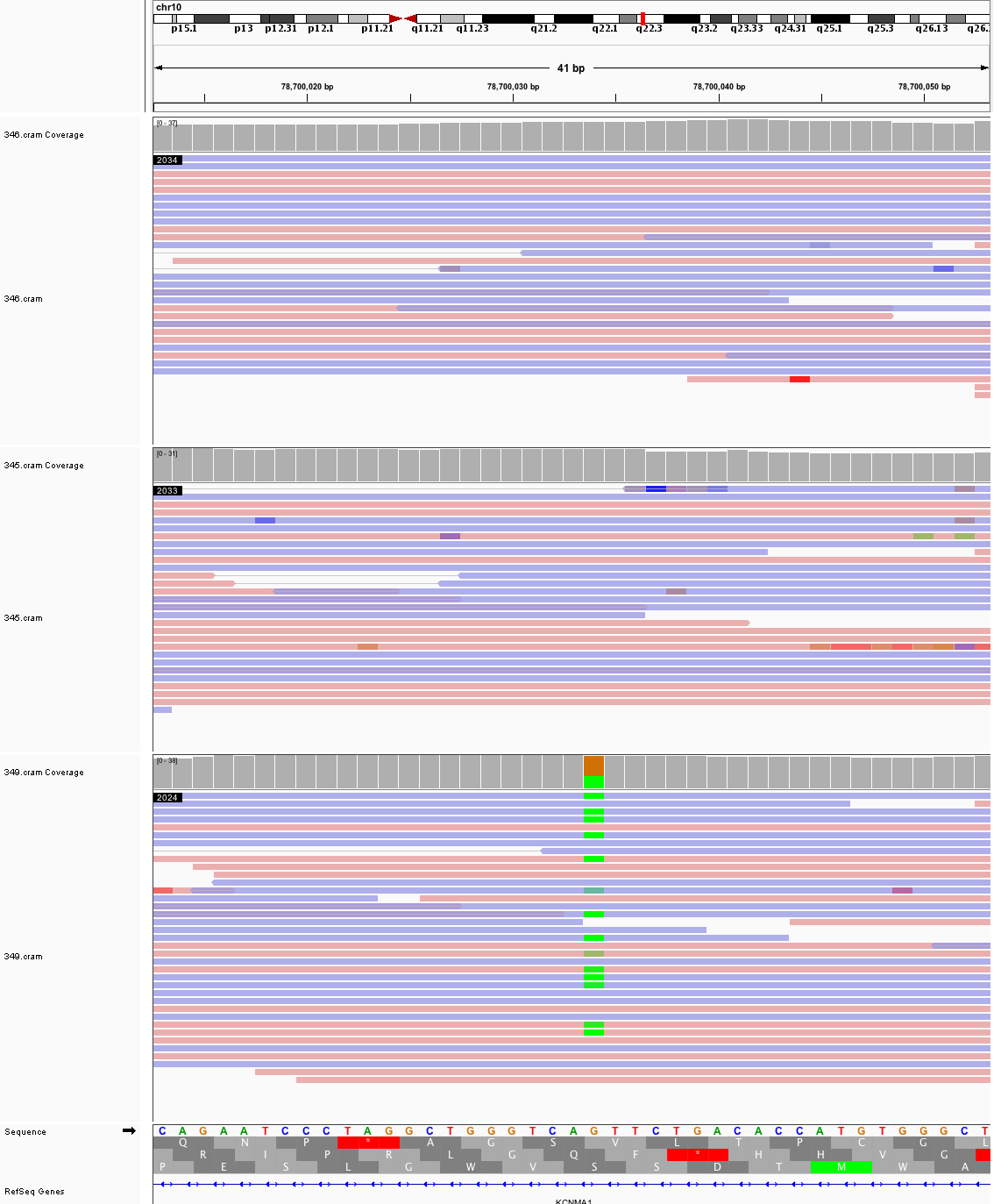

Supplement: Supplementary file 2. — In each image, the first two tracks contain alignments from the first-generation parents, and the third track contains the alignments for the second-generation child. Reads with mapping quality <20 are not included, as they were not considered by our variant calling pipeline, and mismatched bases are shaded by quality score (more transparent = lower base quality). [file elife-46922-supp2.zip › supp_file_2/chr10_78,700,013_78,700,053.png]

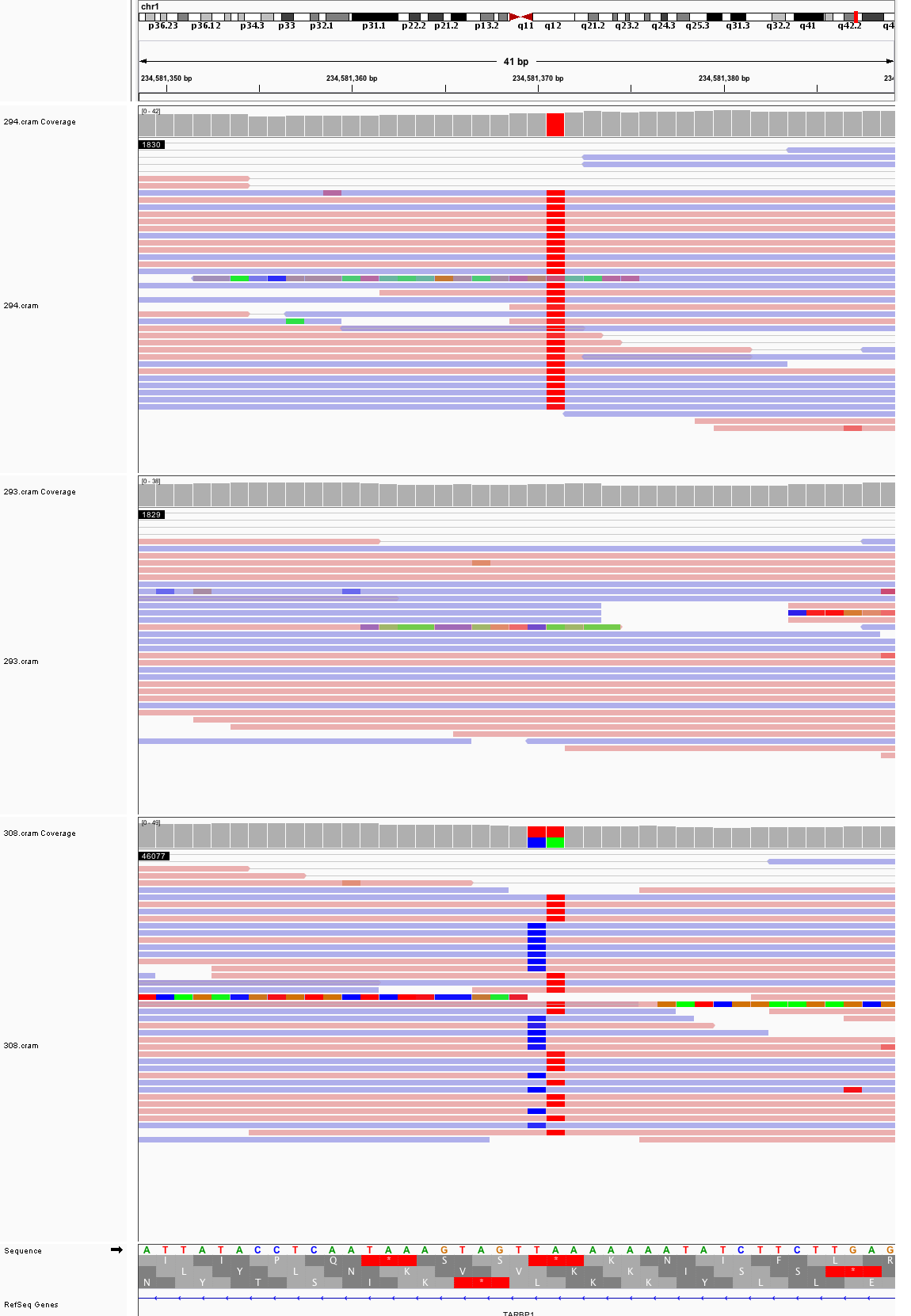

Supplement: Supplementary file 2. — In each image, the first two tracks contain alignments from the first-generation parents, and the third track contains the alignments for the second-generation child. Reads with mapping quality <20 are not included, as they were not considered by our variant calling pipeline, and mismatched bases are shaded by quality score (more transparent = lower base quality). [file elife-46922-supp2.zip › supp_file_2/chr1_234,581,349_234,581,389.png]

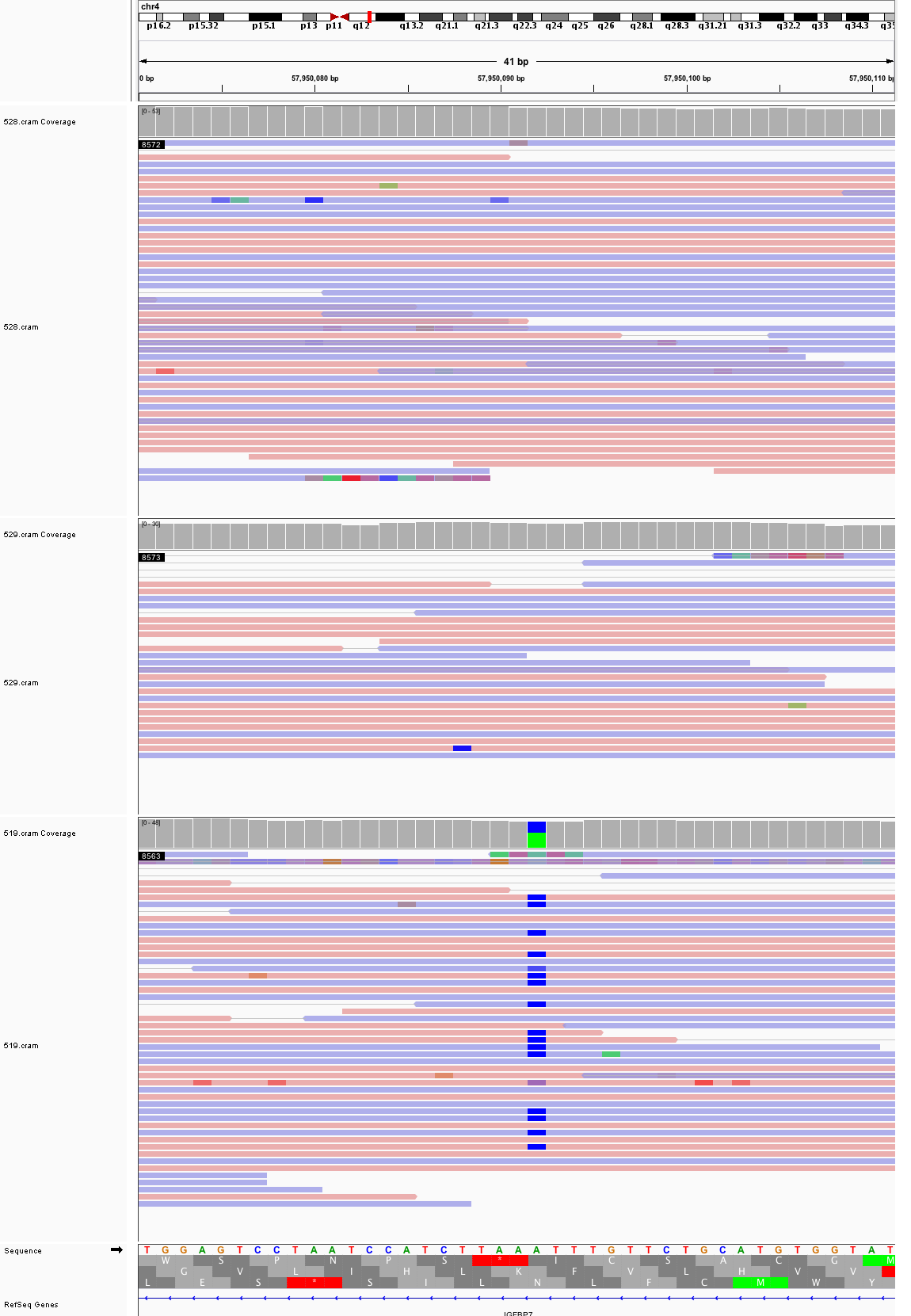

Supplement: Supplementary file 2. — In each image, the first two tracks contain alignments from the first-generation parents, and the third track contains the alignments for the second-generation child. Reads with mapping quality <20 are not included, as they were not considered by our variant calling pipeline, and mismatched bases are shaded by quality score (more transparent = lower base quality). [file elife-46922-supp2.zip › supp_file_2/chr4_57,950,071_57,950,111.png]

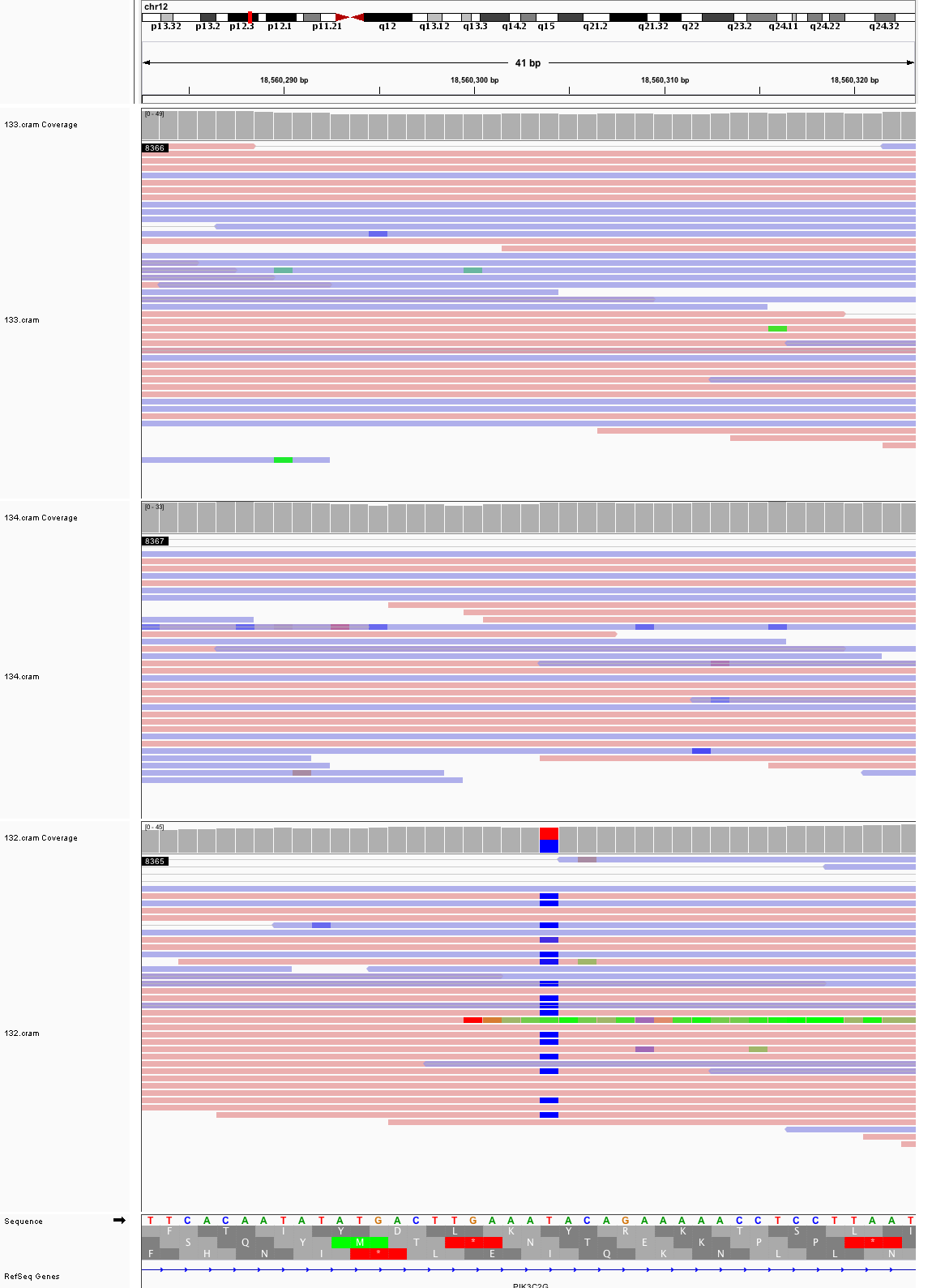

Supplement: Supplementary file 2. — In each image, the first two tracks contain alignments from the first-generation parents, and the third track contains the alignments for the second-generation child. Reads with mapping quality <20 are not included, as they were not considered by our variant calling pipeline, and mismatched bases are shaded by quality score (more transparent = lower base quality). [file elife-46922-supp2.zip › supp_file_2/chr12_18,560,283_18,560,323.png]

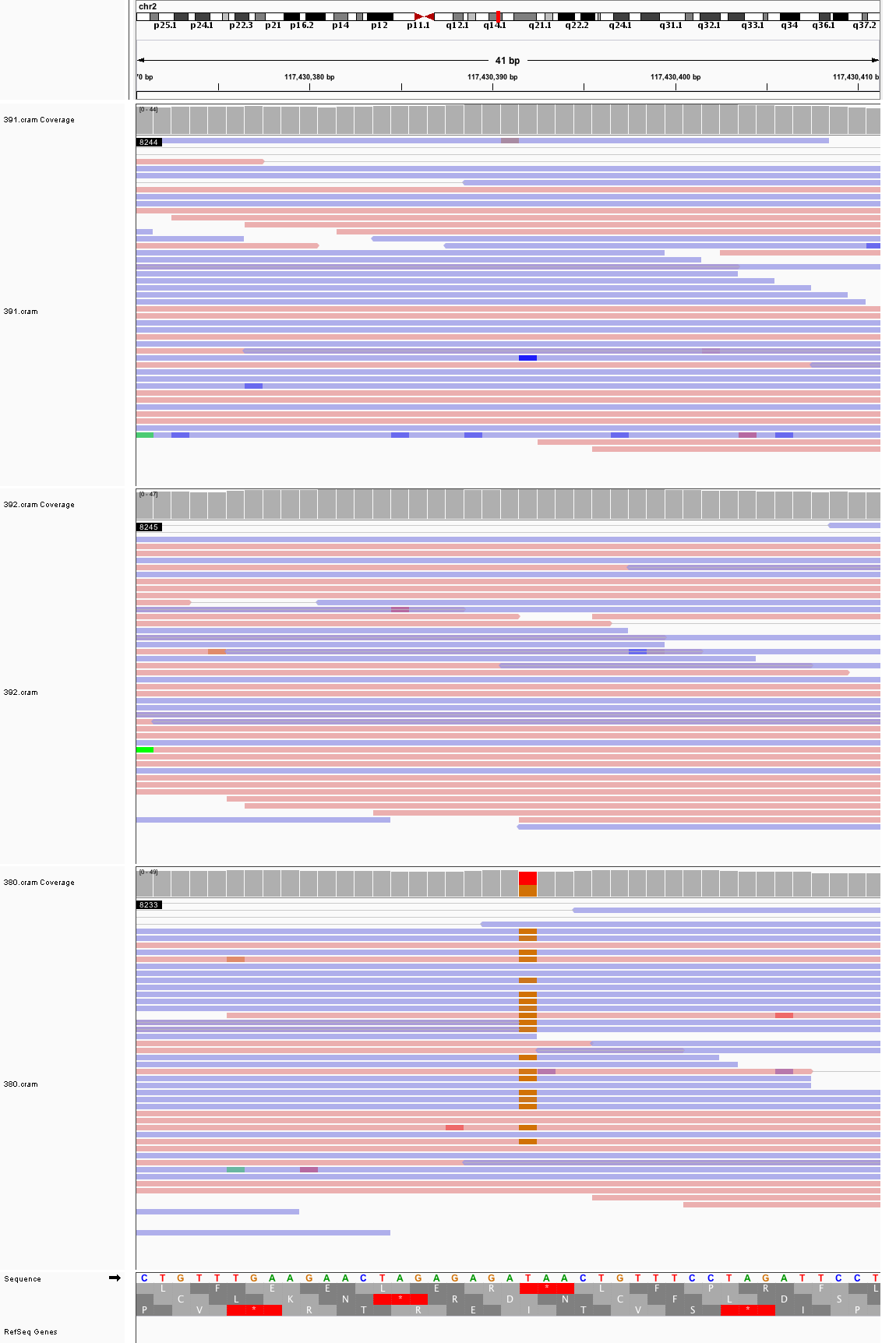

Supplement: Supplementary file 2. — In each image, the first two tracks contain alignments from the first-generation parents, and the third track contains the alignments for the second-generation child. Reads with mapping quality <20 are not included, as they were not considered by our variant calling pipeline, and mismatched bases are shaded by quality score (more transparent = lower base quality). [file elife-46922-supp2.zip › supp_file_2/chr2_117,430,371_117,430,411.png]

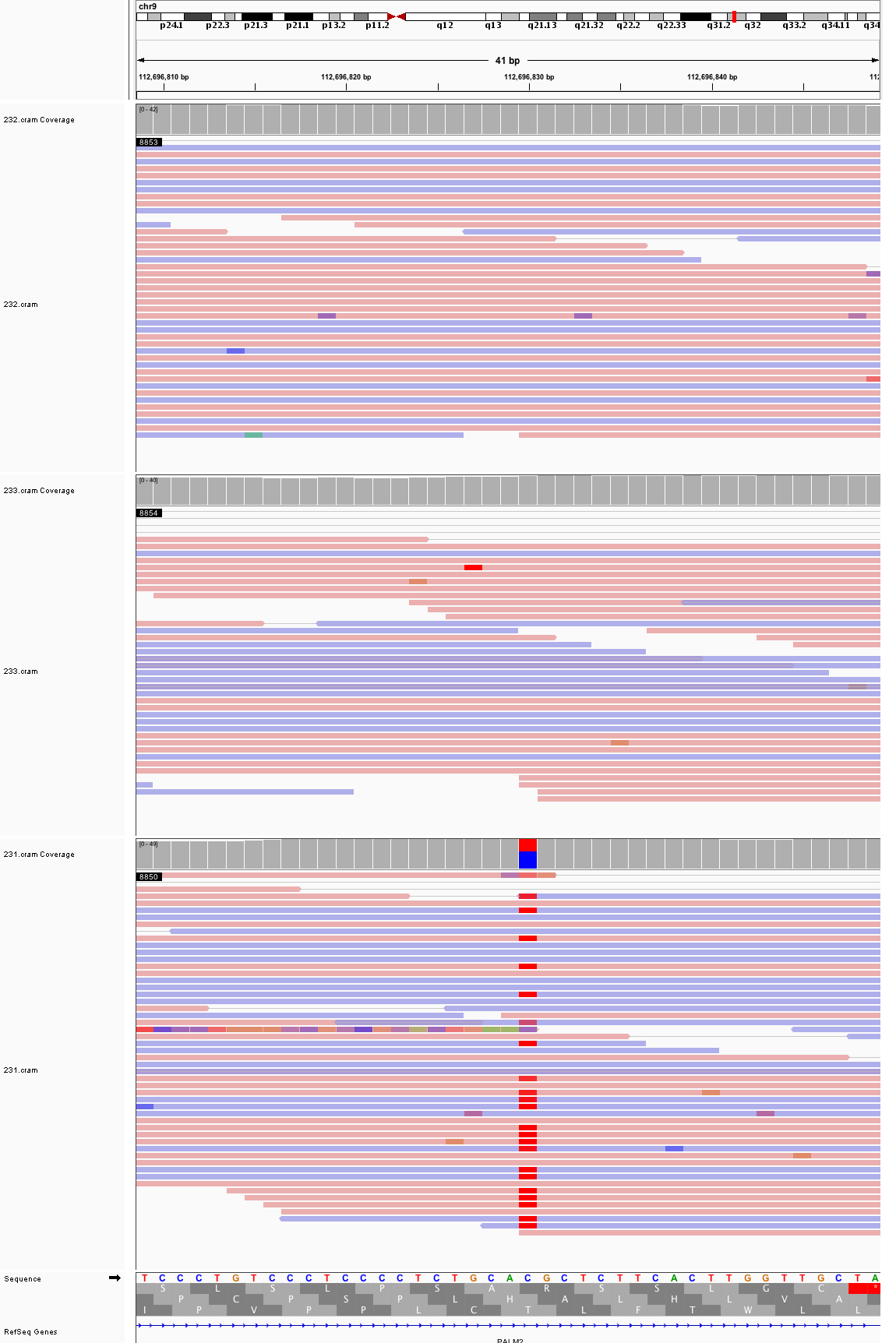

Supplement: Supplementary file 2. — In each image, the first two tracks contain alignments from the first-generation parents, and the third track contains the alignments for the second-generation child. Reads with mapping quality <20 are not included, as they were not considered by our variant calling pipeline, and mismatched bases are shaded by quality score (more transparent = lower base quality). [file elife-46922-supp2.zip › supp_file_2/chr9_112,696,809_112,696,849.png]

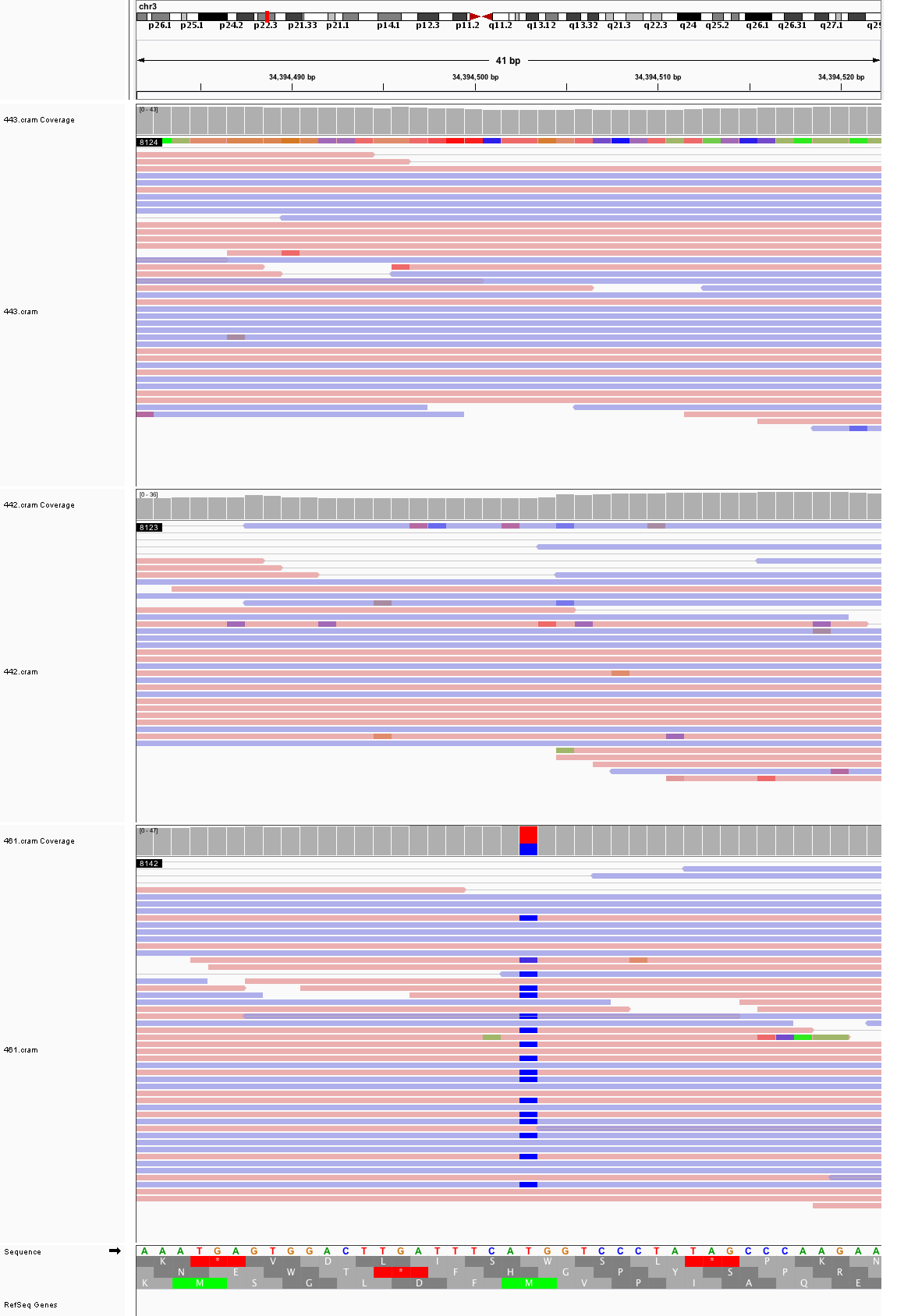

Supplement: Supplementary file 2. — In each image, the first two tracks contain alignments from the first-generation parents, and the third track contains the alignments for the second-generation child. Reads with mapping quality <20 are not included, as they were not considered by our variant calling pipeline, and mismatched bases are shaded by quality score (more transparent = lower base quality). [file elife-46922-supp2.zip › supp_file_2/chr3_34,394,482_34,394,522.png]

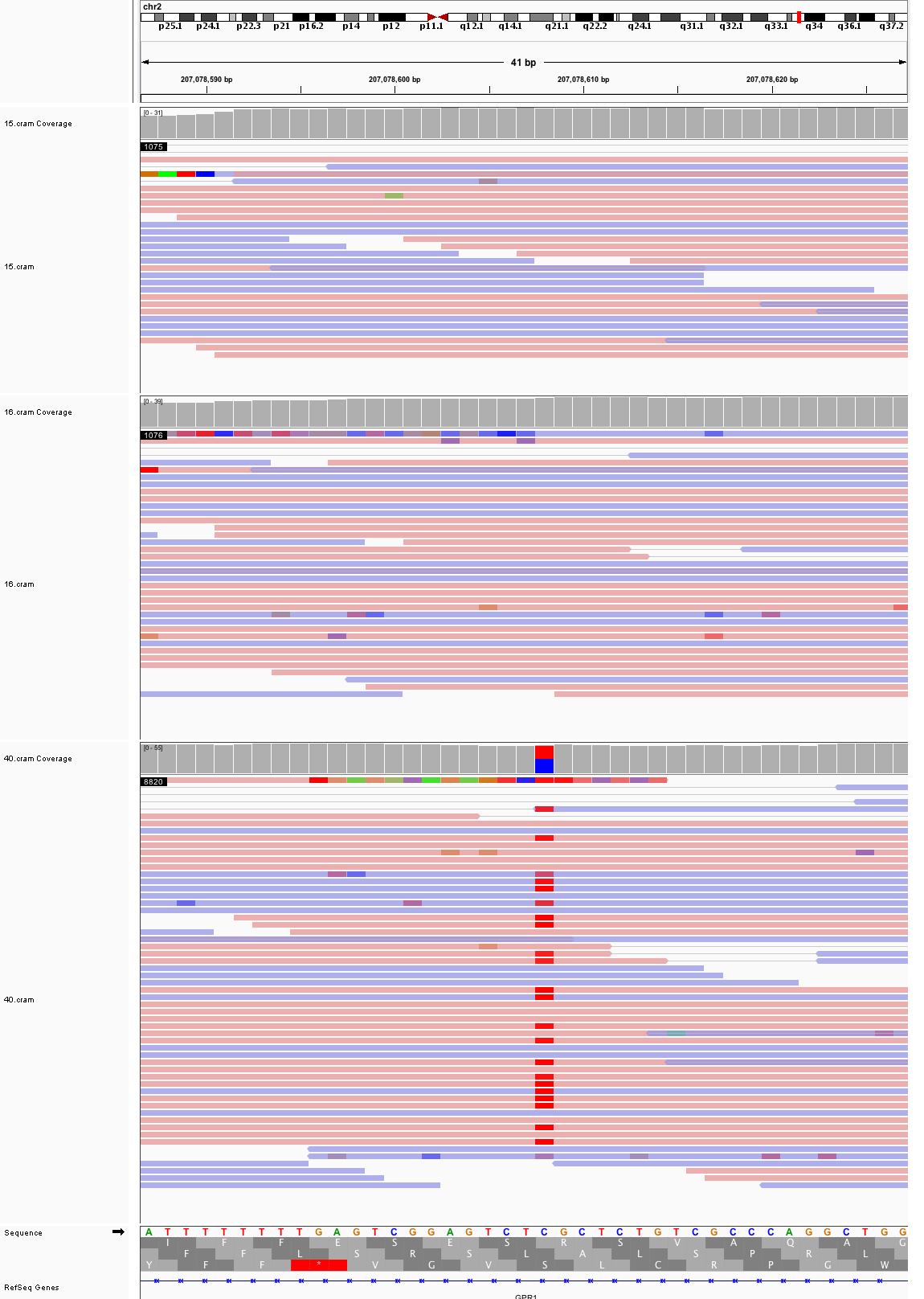

Supplement: Supplementary file 2. — In each image, the first two tracks contain alignments from the first-generation parents, and the third track contains the alignments for the second-generation child. Reads with mapping quality <20 are not included, as they were not considered by our variant calling pipeline, and mismatched bases are shaded by quality score (more transparent = lower base quality). [file elife-46922-supp2.zip › supp_file_2/chr2_207,078,587_207,078,627.png]

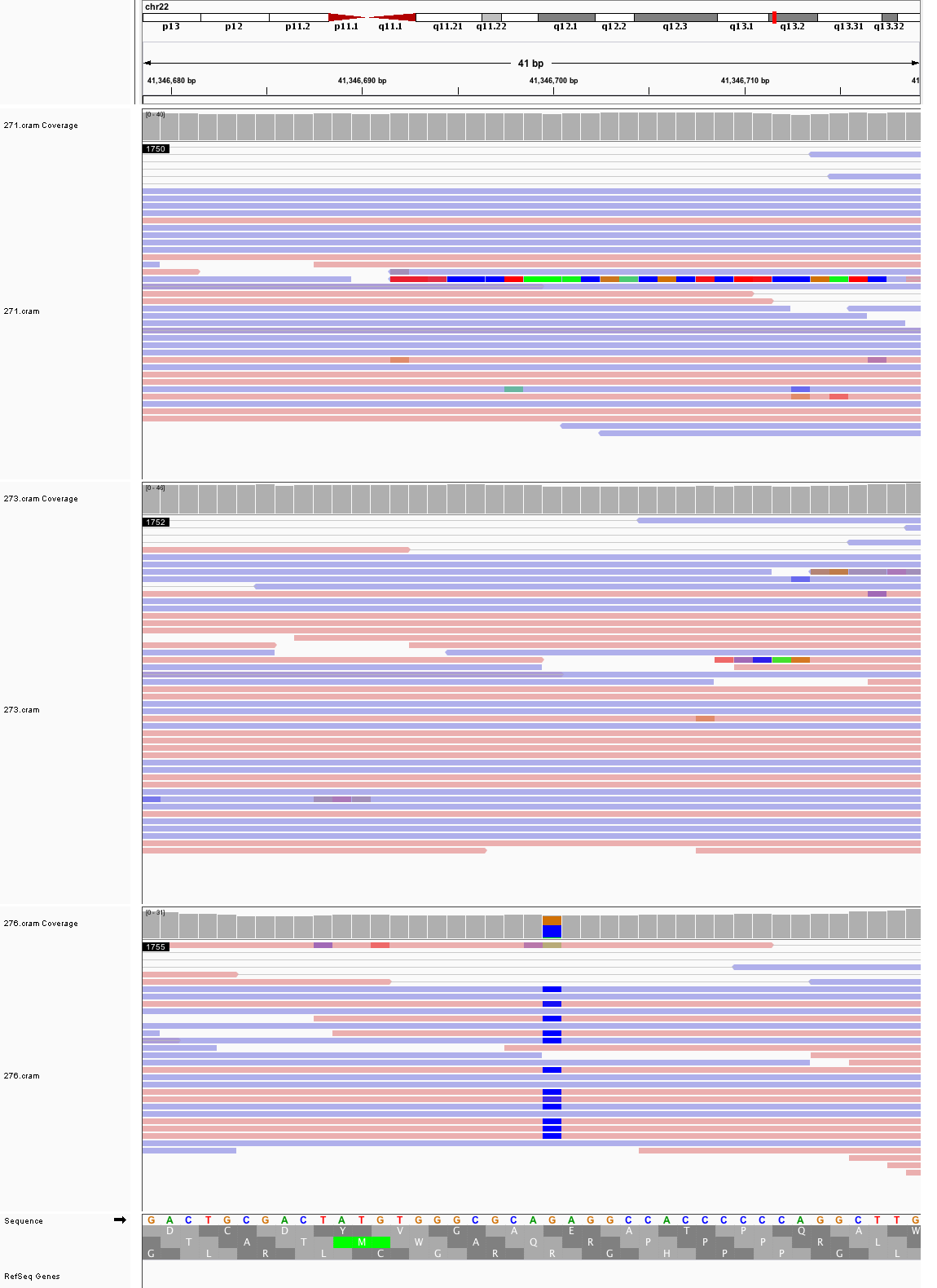

Supplement: Supplementary file 2. — In each image, the first two tracks contain alignments from the first-generation parents, and the third track contains the alignments for the second-generation child. Reads with mapping quality <20 are not included, as they were not considered by our variant calling pipeline, and mismatched bases are shaded by quality score (more transparent = lower base quality). [file elife-46922-supp2.zip › supp_file_2/chr22_41,346,679_41,346,719.png]

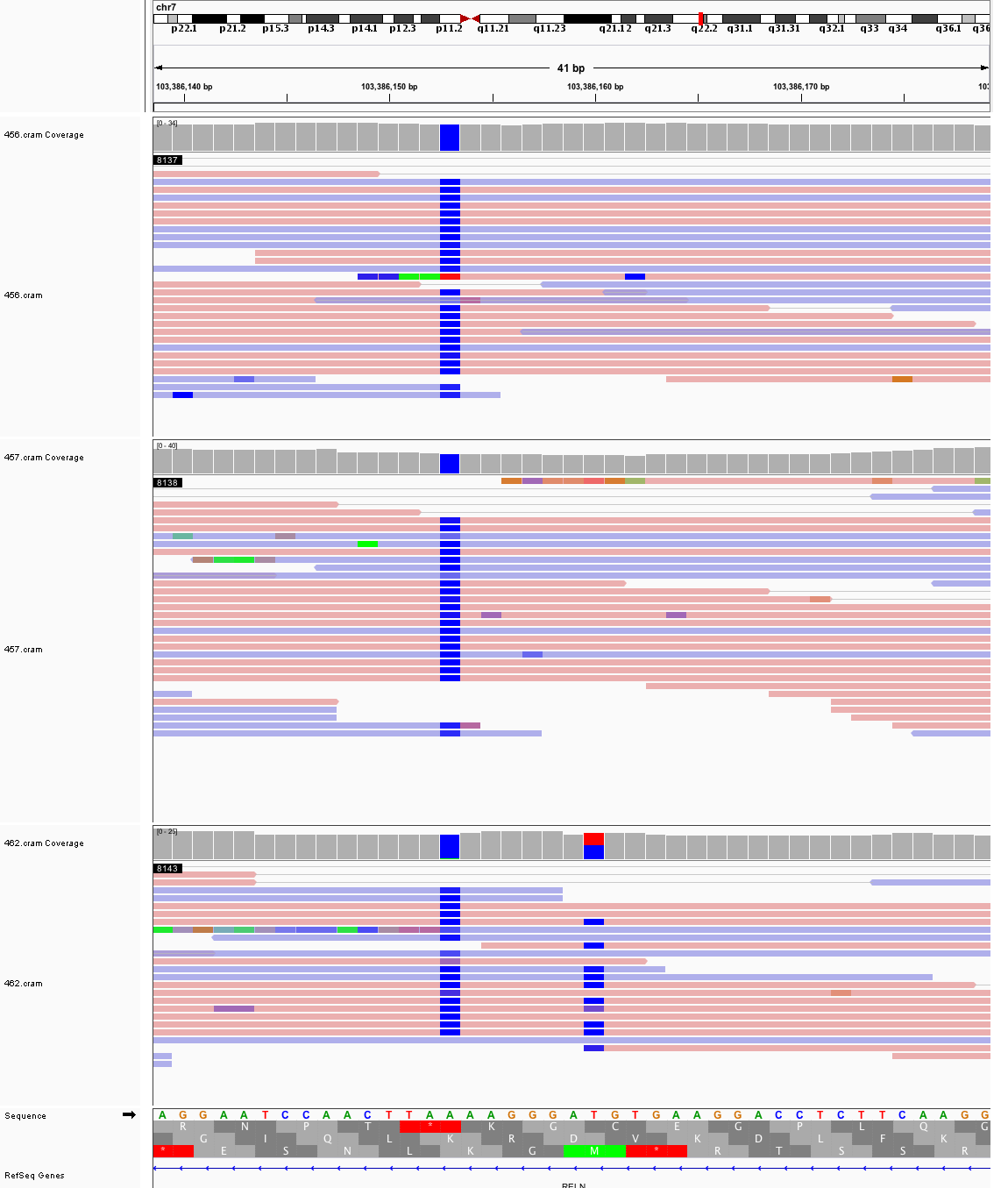

Supplement: Supplementary file 2. — In each image, the first two tracks contain alignments from the first-generation parents, and the third track contains the alignments for the second-generation child. Reads with mapping quality <20 are not included, as they were not considered by our variant calling pipeline, and mismatched bases are shaded by quality score (more transparent = lower base quality). [file elife-46922-supp2.zip › supp_file_2/chr7_103,386,139_103,386,179.png]

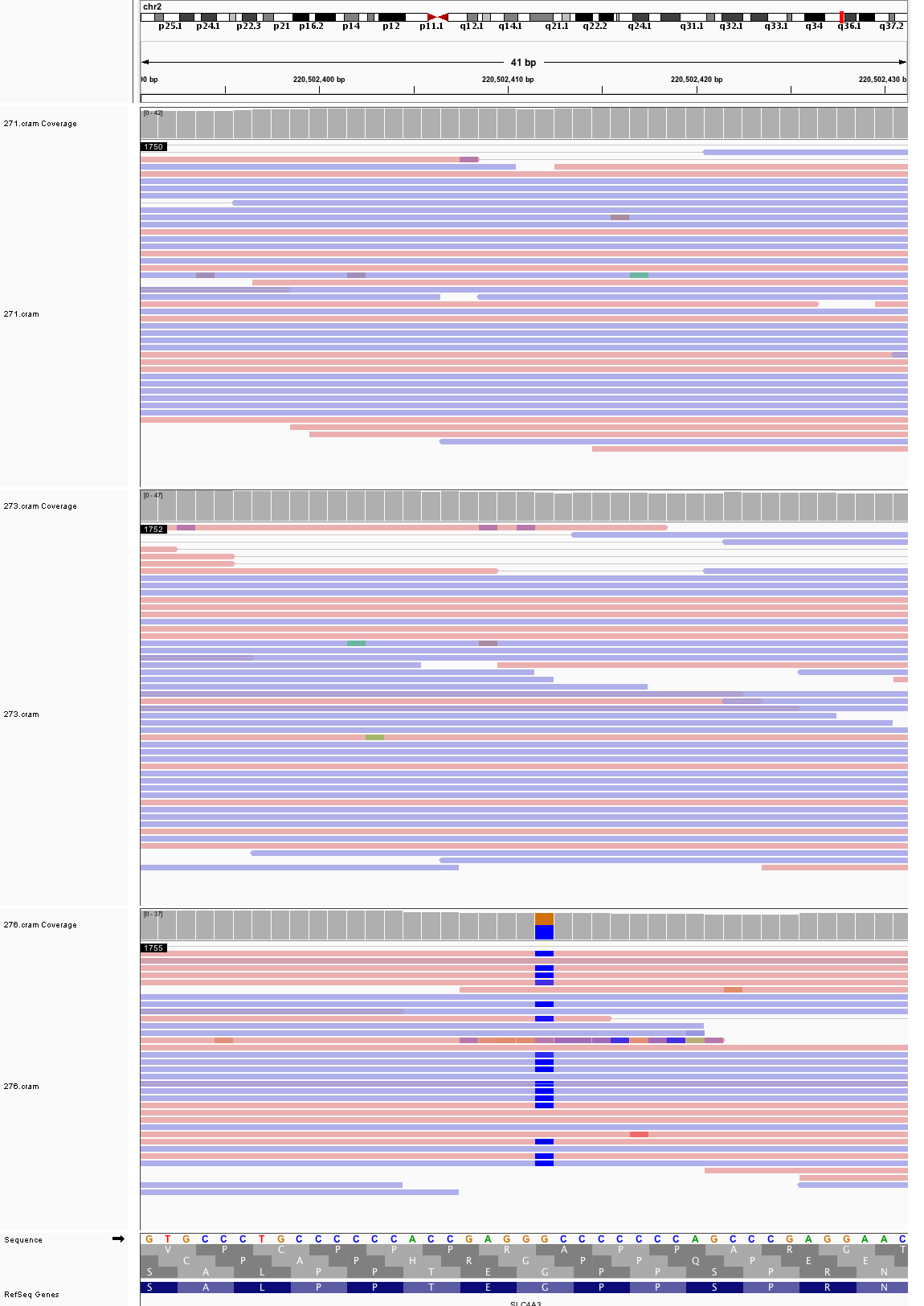

Supplement: Supplementary file 2. — In each image, the first two tracks contain alignments from the first-generation parents, and the third track contains the alignments for the second-generation child. Reads with mapping quality <20 are not included, as they were not considered by our variant calling pipeline, and mismatched bases are shaded by quality score (more transparent = lower base quality). [file elife-46922-supp2.zip › supp_file_2/chr2_220,502,391_220,502,431.png]

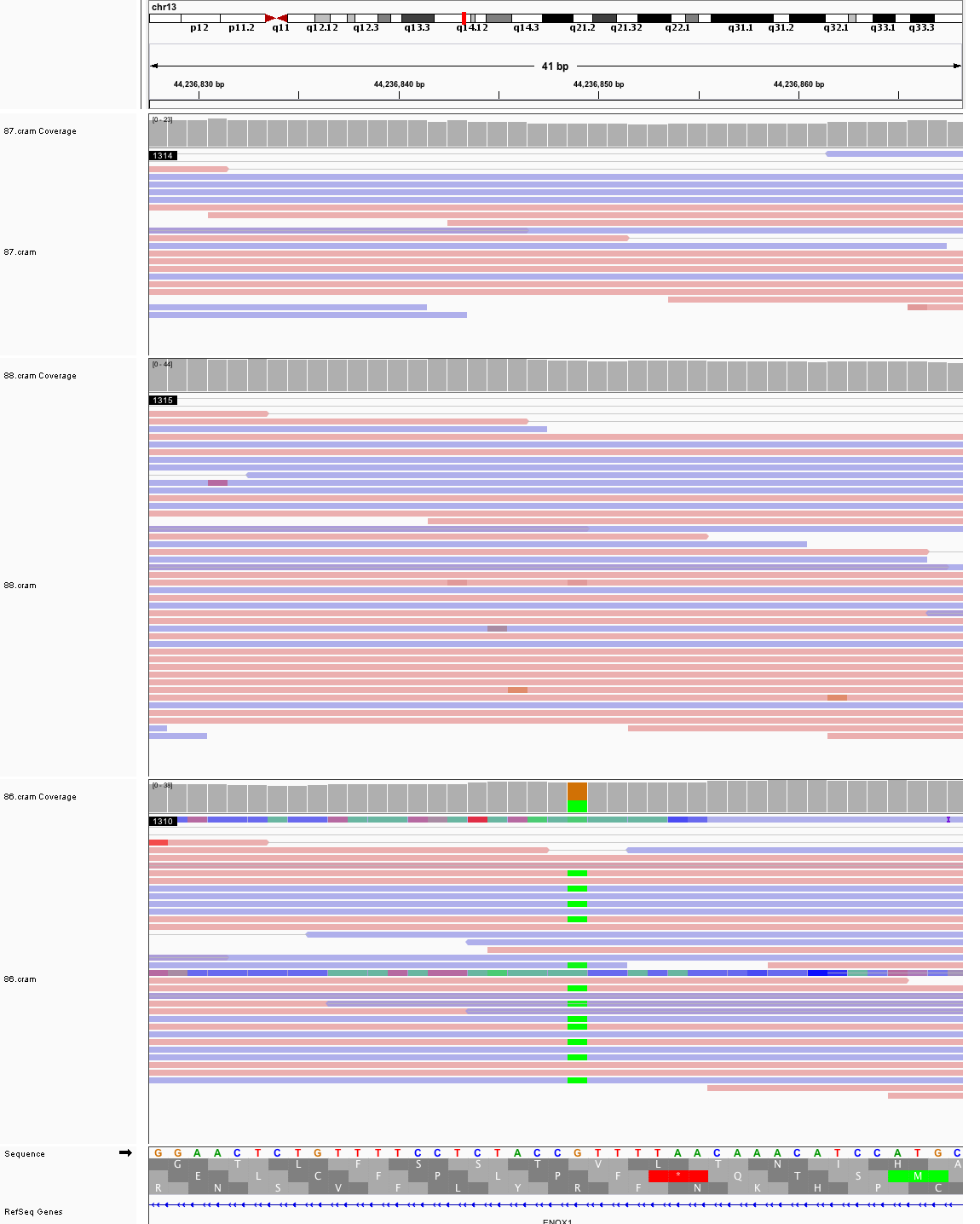

Supplement: Supplementary file 2. — In each image, the first two tracks contain alignments from the first-generation parents, and the third track contains the alignments for the second-generation child. Reads with mapping quality <20 are not included, as they were not considered by our variant calling pipeline, and mismatched bases are shaded by quality score (more transparent = lower base quality). [file elife-46922-supp2.zip › supp_file_2/chr13_44,236,828_44,236,868.png]

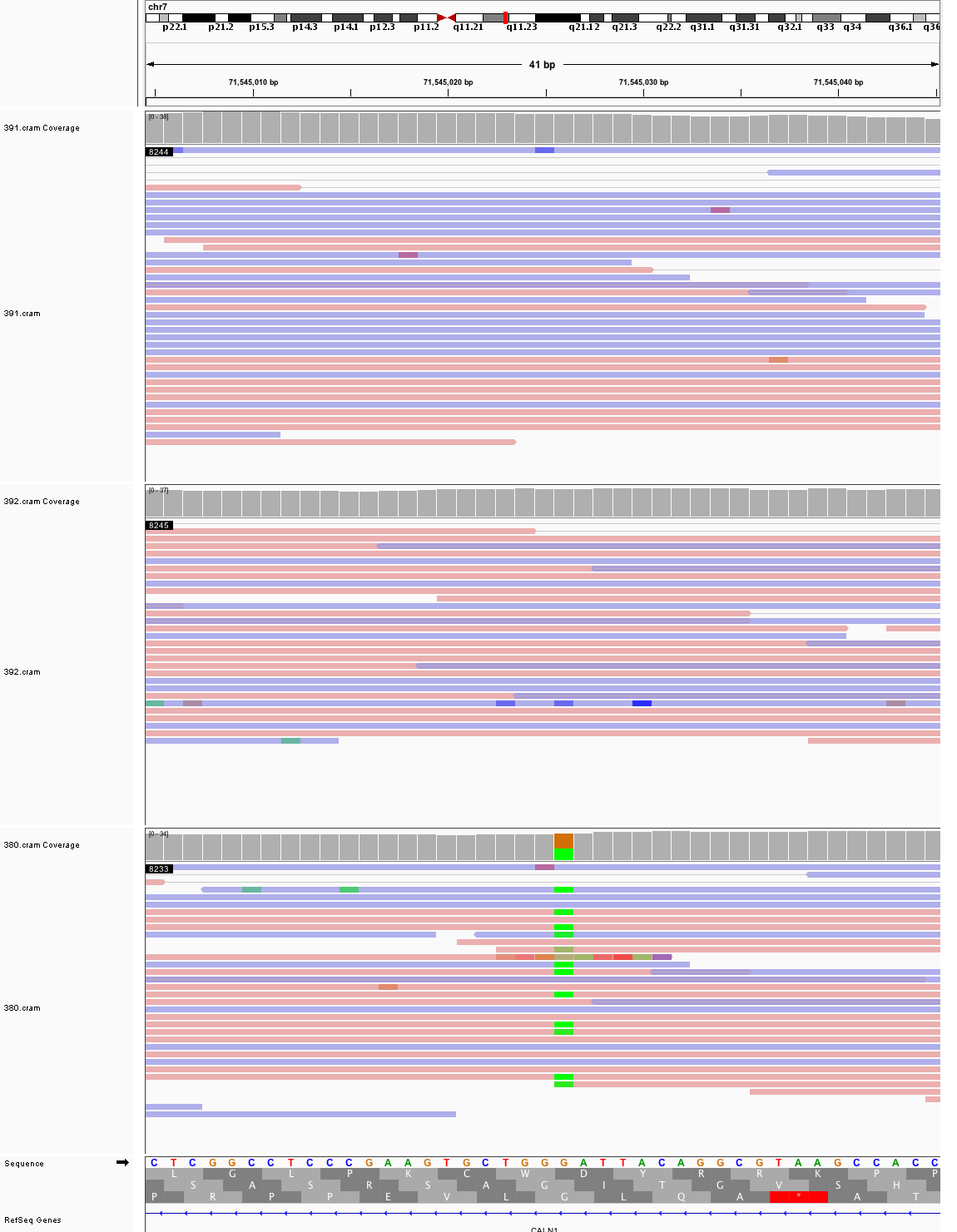

Supplement: Supplementary file 2. — In each image, the first two tracks contain alignments from the first-generation parents, and the third track contains the alignments for the second-generation child. Reads with mapping quality <20 are not included, as they were not considered by our variant calling pipeline, and mismatched bases are shaded by quality score (more transparent = lower base quality). [file elife-46922-supp2.zip › supp_file_2/chr7_71,545,005_71,545,045.png]

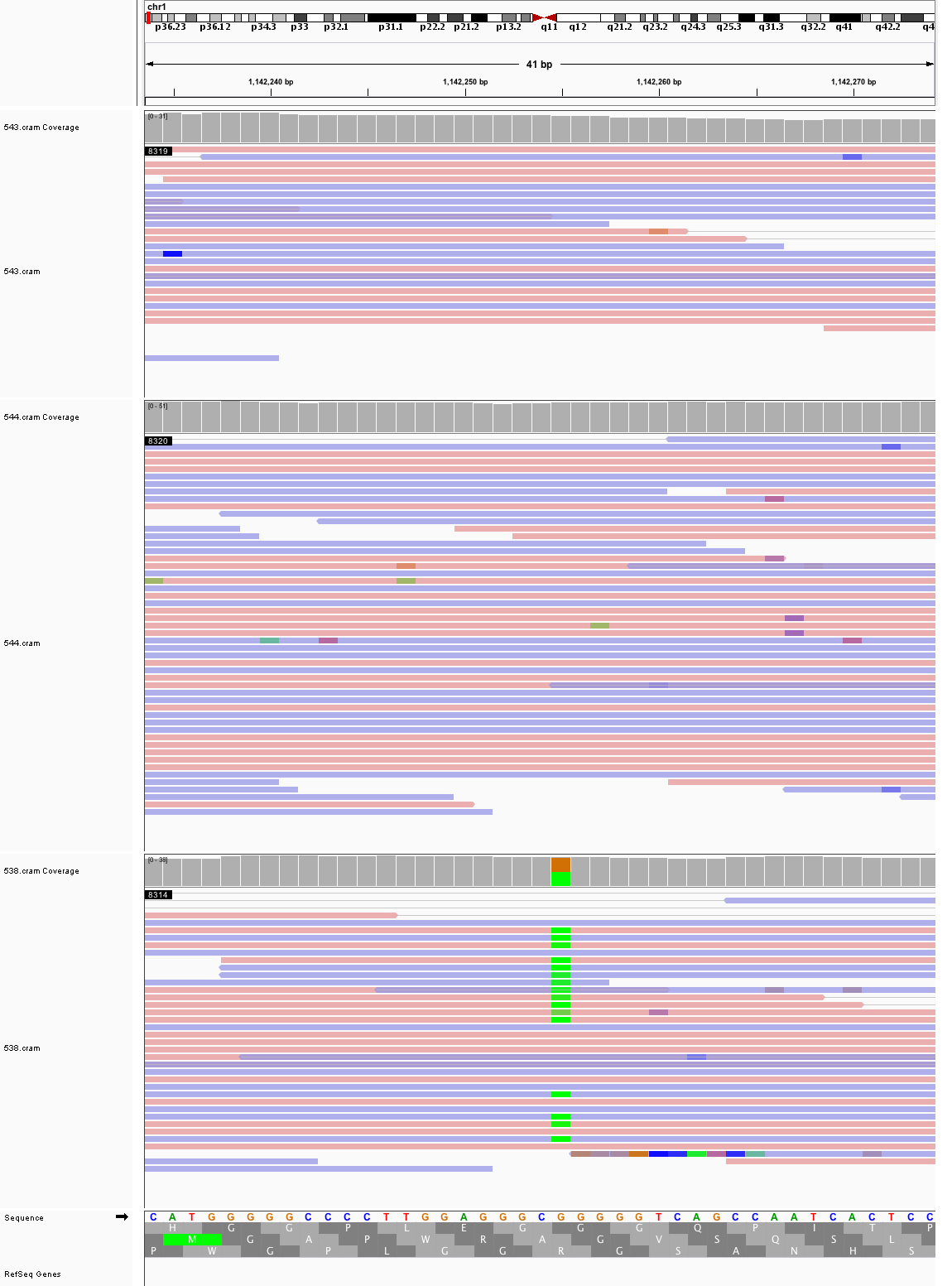

Supplement: Supplementary file 2. — In each image, the first two tracks contain alignments from the first-generation parents, and the third track contains the alignments for the second-generation child. Reads with mapping quality <20 are not included, as they were not considered by our variant calling pipeline, and mismatched bases are shaded by quality score (more transparent = lower base quality). [file elife-46922-supp2.zip › supp_file_2/chr1_1,142,234_1,142,274.png]

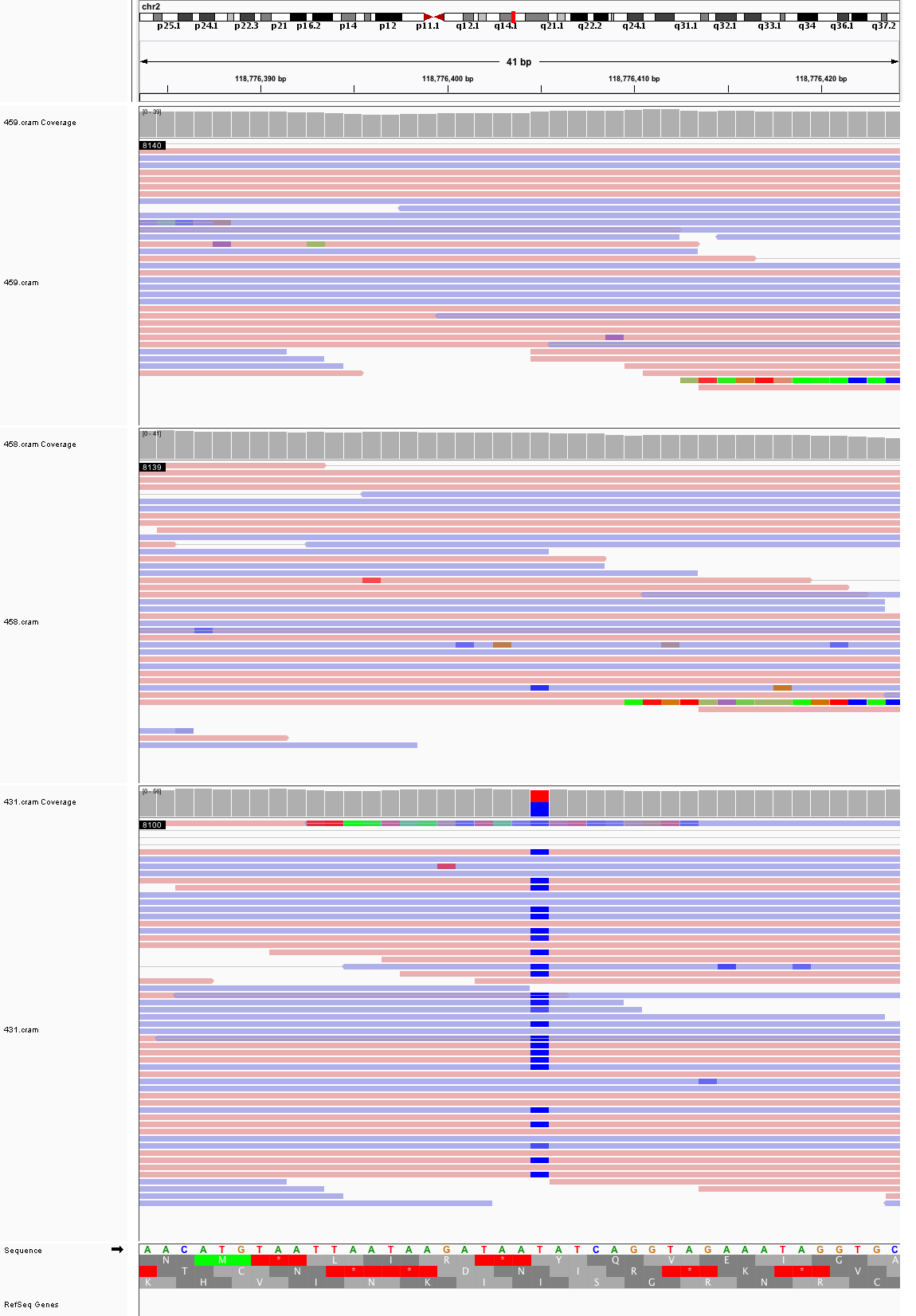

Supplement: Supplementary file 2. — In each image, the first two tracks contain alignments from the first-generation parents, and the third track contains the alignments for the second-generation child. Reads with mapping quality <20 are not included, as they were not considered by our variant calling pipeline, and mismatched bases are shaded by quality score (more transparent = lower base quality). [file elife-46922-supp2.zip › supp_file_2/chr2_118,776,384_118,776,424.png]
