# Supplementary figures and images for: Large, three-generation human families reveal post-zygotic mosaicism and variability in germline mutation accumulation (part 2 of 7)
Source: eLife. 2019 Sep 24;8:e46922. doi: 10.7554/eLife.46922 (PMC6759356; doi:10.7554/eLife.46922)

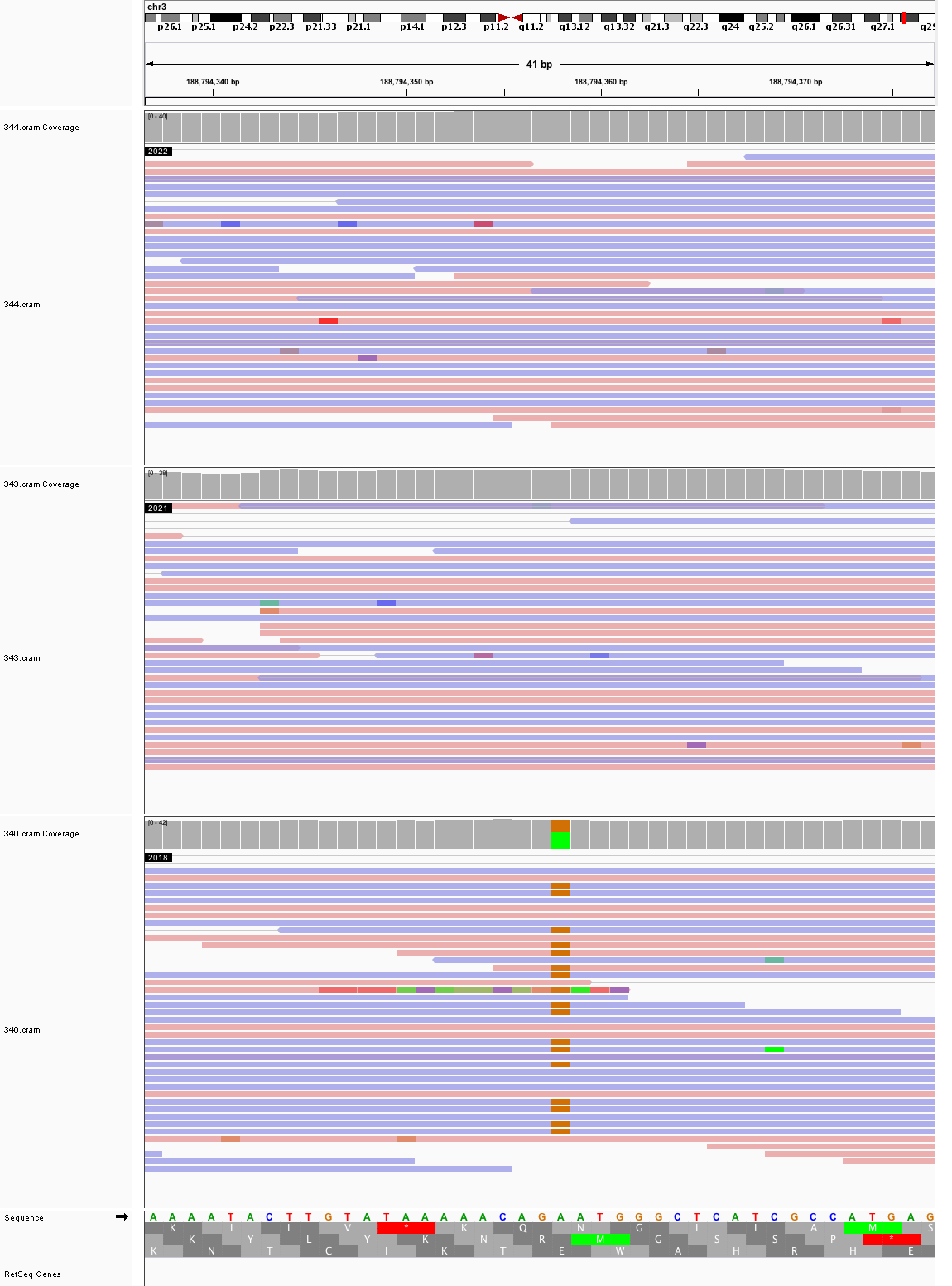

Supplement: Supplementary file 2. — In each image, the first two tracks contain alignments from the first-generation parents, and the third track contains the alignments for the second-generation child. Reads with mapping quality <20 are not included, as they were not considered by our variant calling pipeline, and mismatched bases are shaded by quality score (more transparent = lower base quality). [file elife-46922-supp2.zip › supp_file_2/chr3_188,794,337_188,794,377.png]

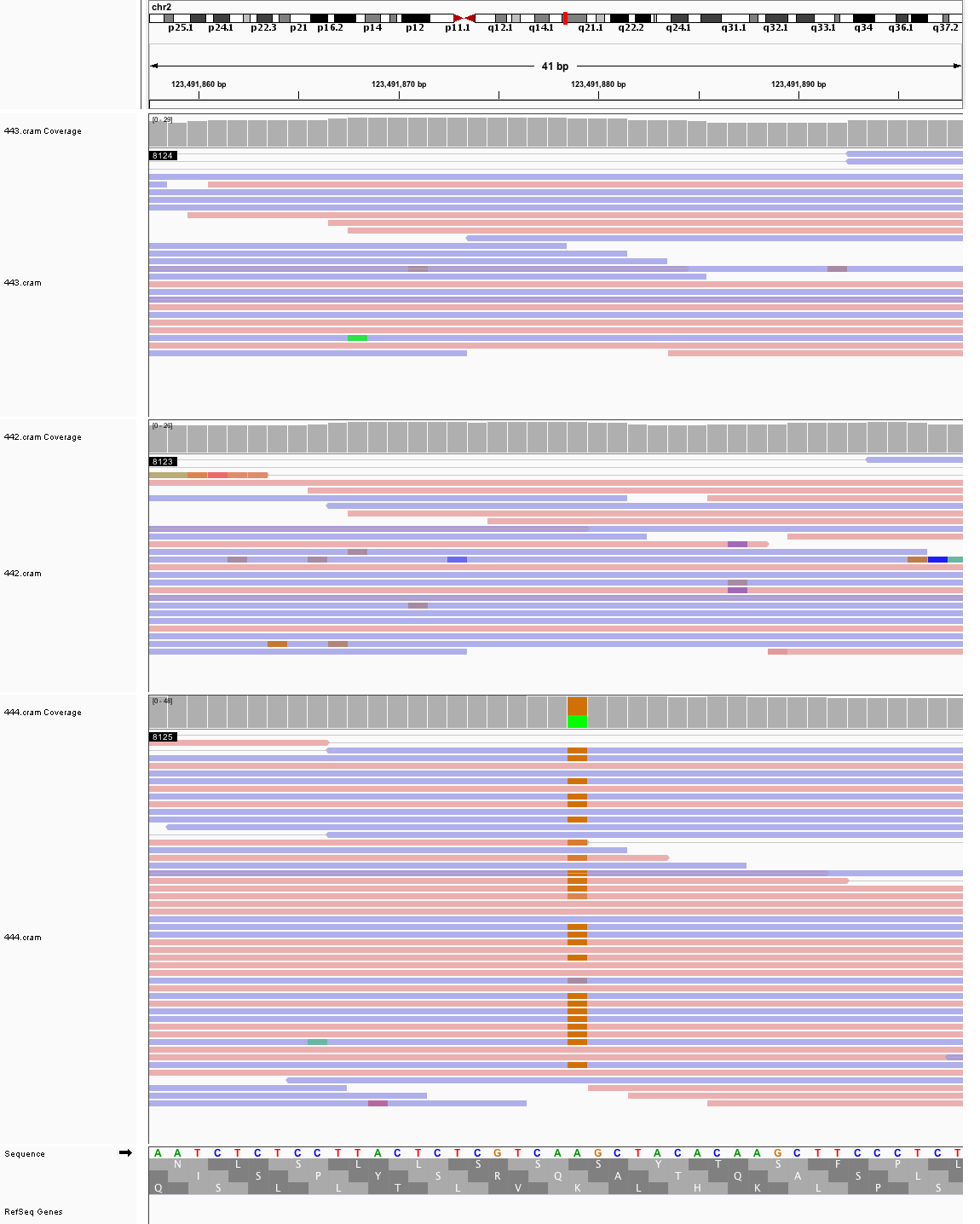

Supplement: Supplementary file 2. — In each image, the first two tracks contain alignments from the first-generation parents, and the third track contains the alignments for the second-generation child. Reads with mapping quality <20 are not included, as they were not considered by our variant calling pipeline, and mismatched bases are shaded by quality score (more transparent = lower base quality). [file elife-46922-supp2.zip › supp_file_2/chr2_123,491,858_123,491,898.png]

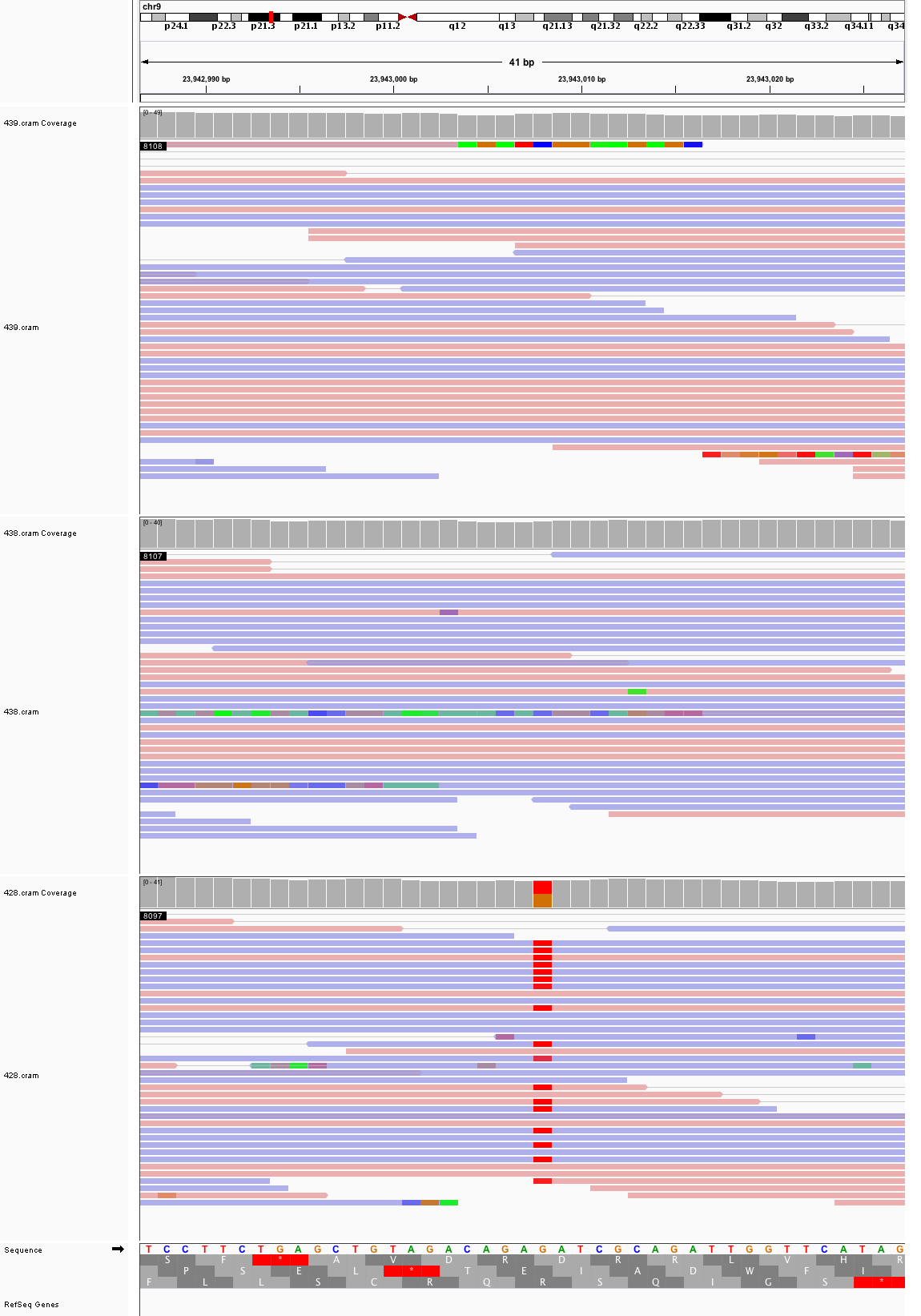

Supplement: Supplementary file 2. — In each image, the first two tracks contain alignments from the first-generation parents, and the third track contains the alignments for the second-generation child. Reads with mapping quality <20 are not included, as they were not considered by our variant calling pipeline, and mismatched bases are shaded by quality score (more transparent = lower base quality). [file elife-46922-supp2.zip › supp_file_2/chr9_23,942,987_23,943,027.png]

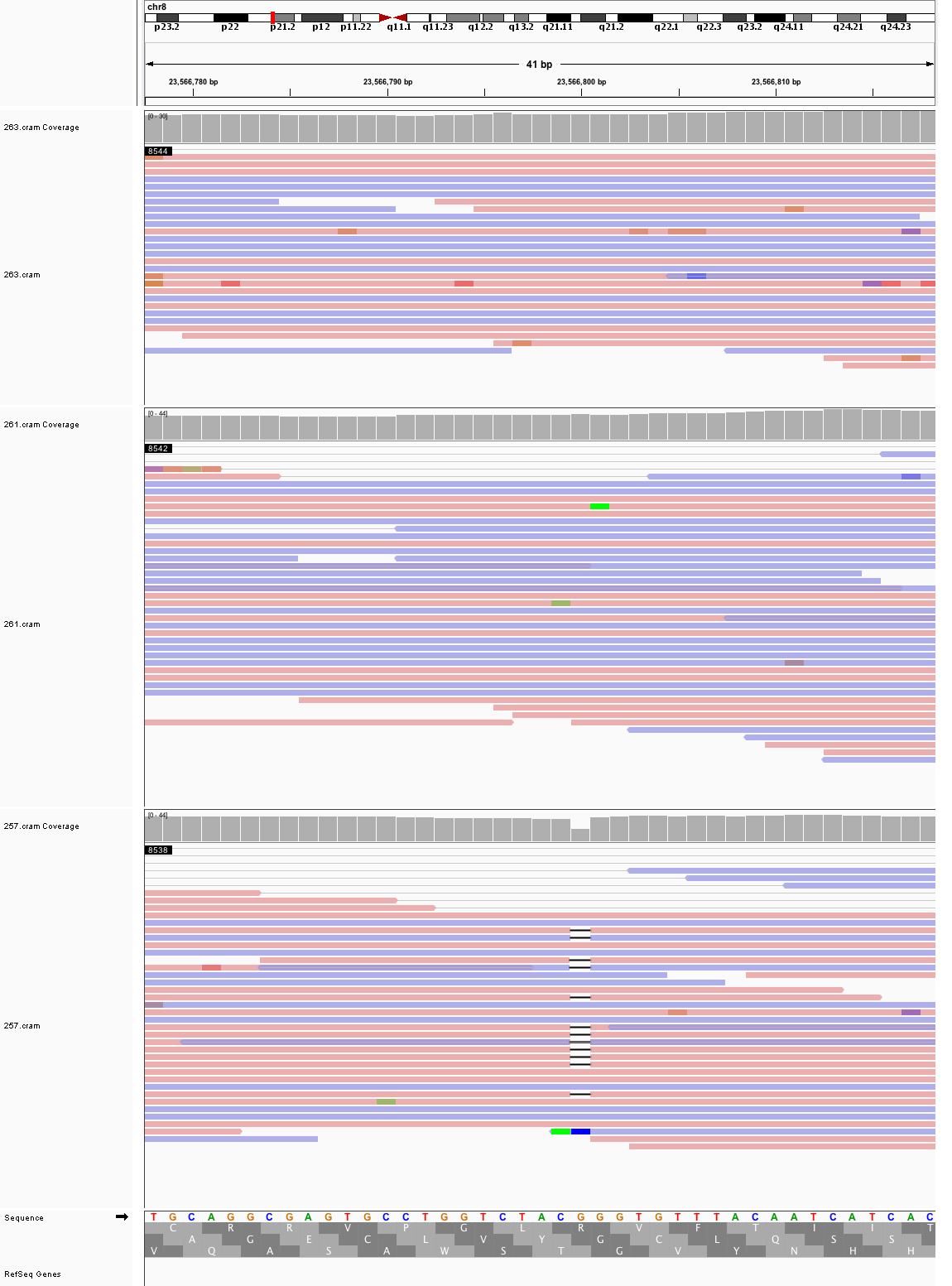

Supplement: Supplementary file 2. — In each image, the first two tracks contain alignments from the first-generation parents, and the third track contains the alignments for the second-generation child. Reads with mapping quality <20 are not included, as they were not considered by our variant calling pipeline, and mismatched bases are shaded by quality score (more transparent = lower base quality). [file elife-46922-supp2.zip › supp_file_2/chr8_23,566,778_23,566,818.png]

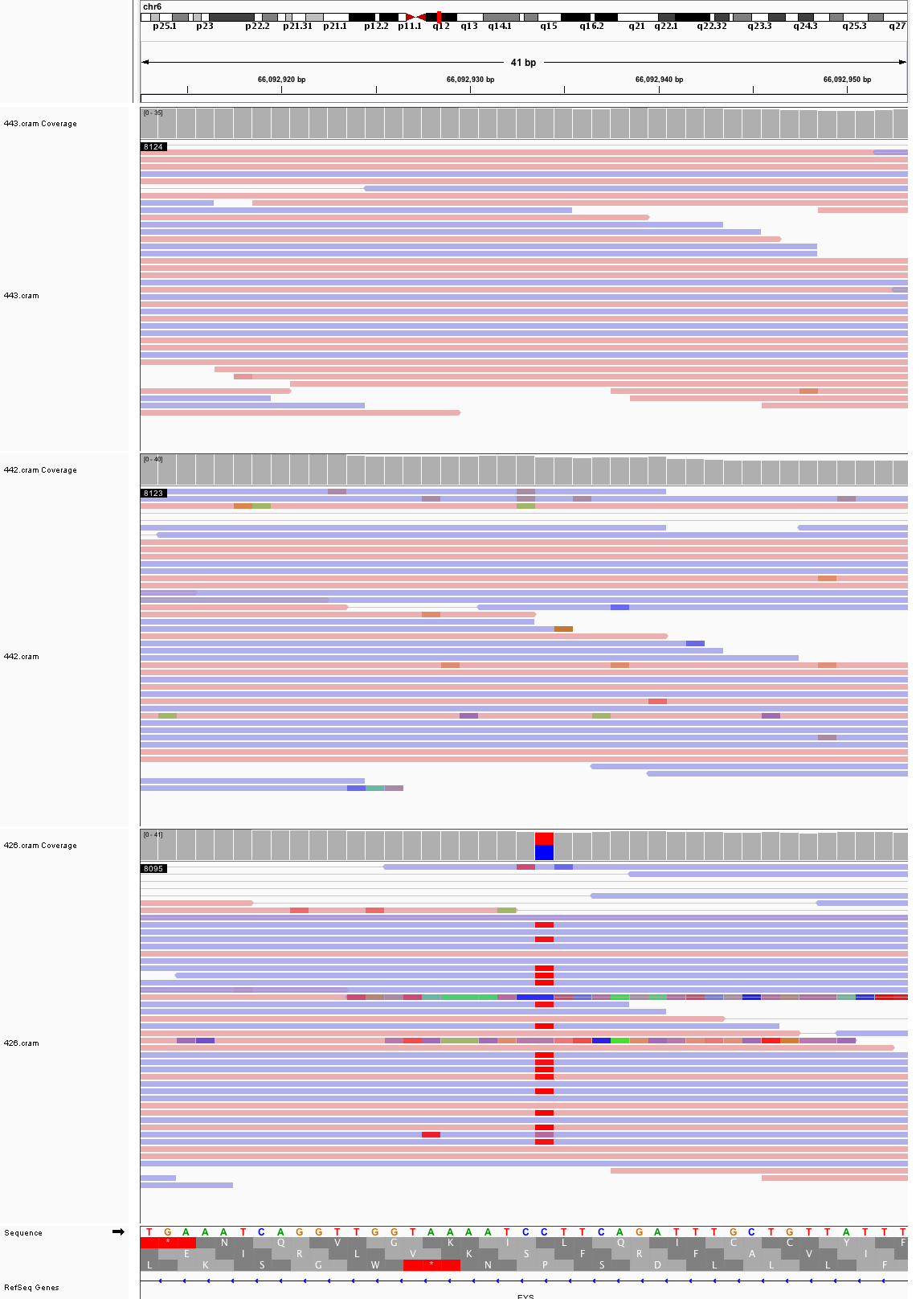

Supplement: Supplementary file 2. — In each image, the first two tracks contain alignments from the first-generation parents, and the third track contains the alignments for the second-generation child. Reads with mapping quality <20 are not included, as they were not considered by our variant calling pipeline, and mismatched bases are shaded by quality score (more transparent = lower base quality). [file elife-46922-supp2.zip › supp_file_2/chr6_66,092,913_66,092,953.png]

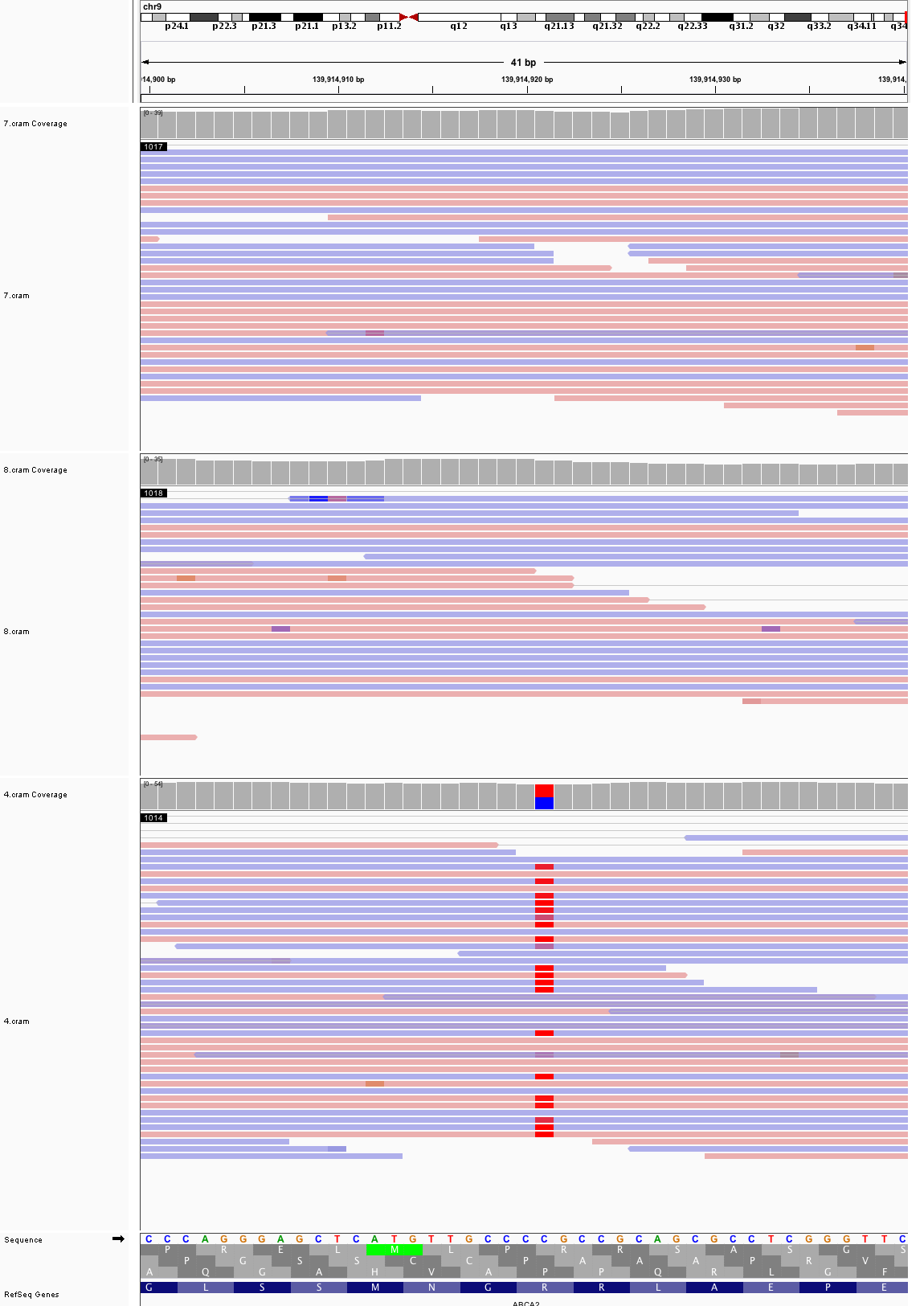

Supplement: Supplementary file 2. — In each image, the first two tracks contain alignments from the first-generation parents, and the third track contains the alignments for the second-generation child. Reads with mapping quality <20 are not included, as they were not considered by our variant calling pipeline, and mismatched bases are shaded by quality score (more transparent = lower base quality). [file elife-46922-supp2.zip › supp_file_2/chr9_139,914,900_139,914,940.png]

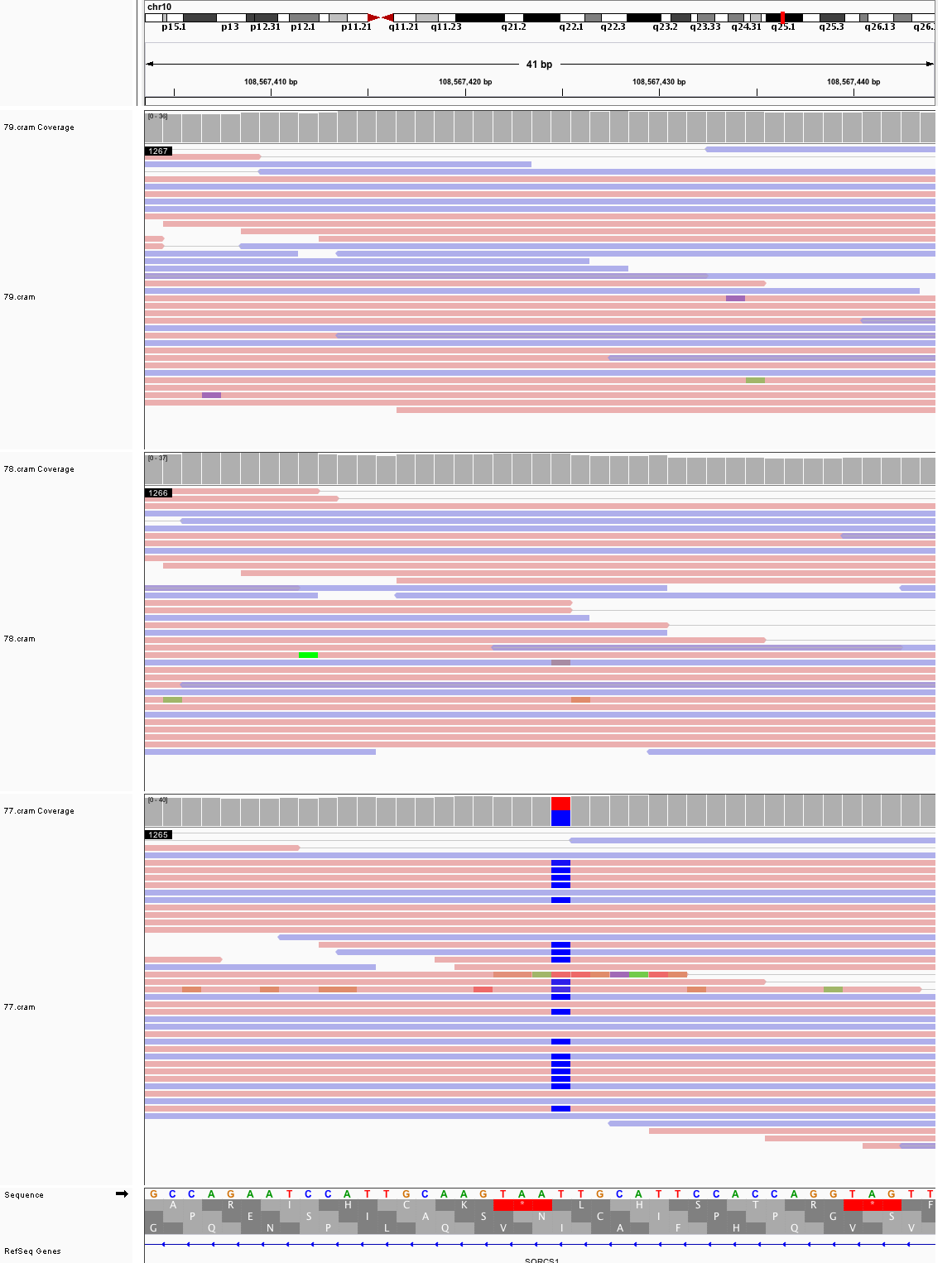

Supplement: Supplementary file 2. — In each image, the first two tracks contain alignments from the first-generation parents, and the third track contains the alignments for the second-generation child. Reads with mapping quality <20 are not included, as they were not considered by our variant calling pipeline, and mismatched bases are shaded by quality score (more transparent = lower base quality). [file elife-46922-supp2.zip › supp_file_2/chr10_108,567,404_108,567,444.png]

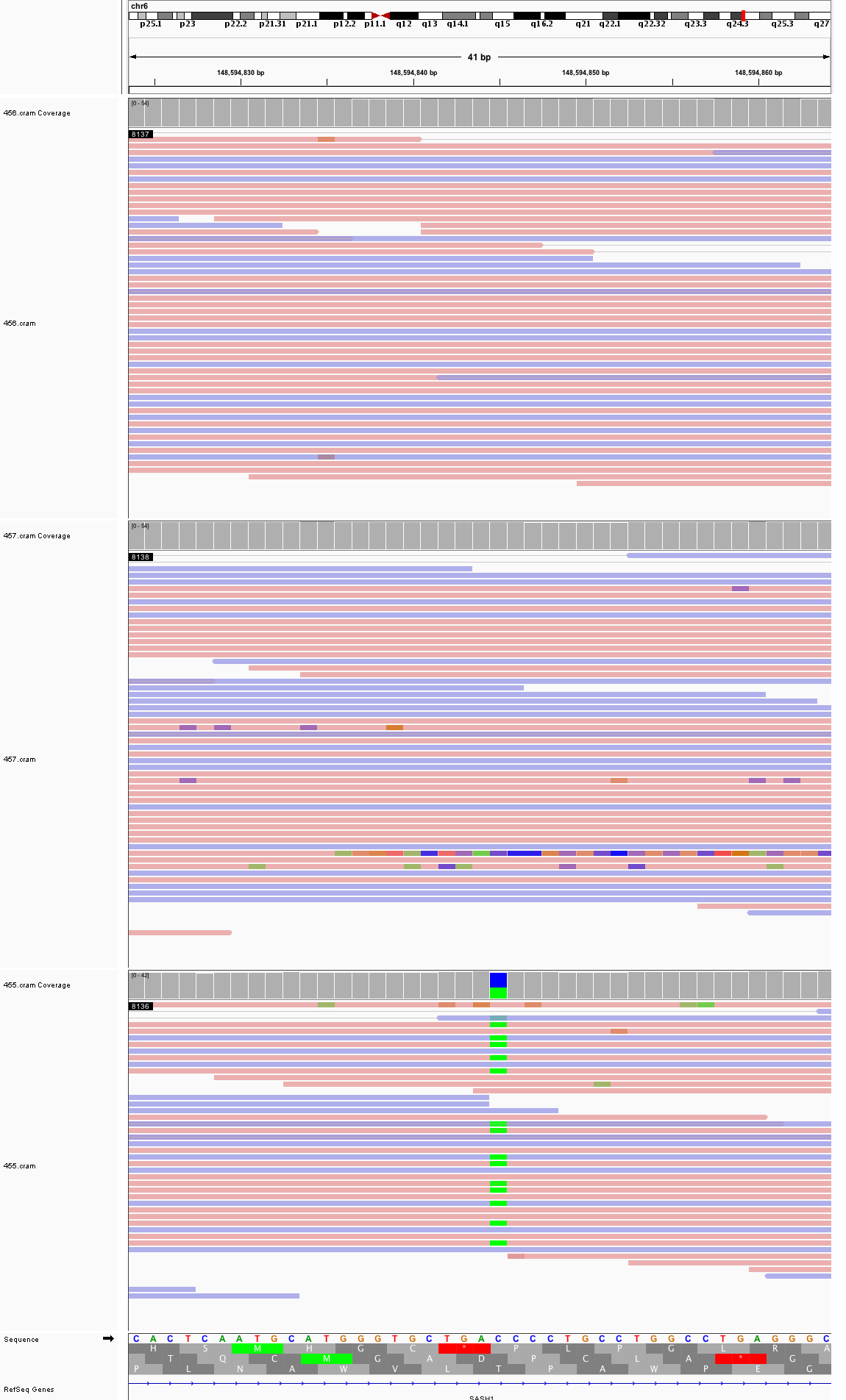

Supplement: Supplementary file 2. — In each image, the first two tracks contain alignments from the first-generation parents, and the third track contains the alignments for the second-generation child. Reads with mapping quality <20 are not included, as they were not considered by our variant calling pipeline, and mismatched bases are shaded by quality score (more transparent = lower base quality). [file elife-46922-supp2.zip › supp_file_2/chr6_148,594,824_148,594,864.png]

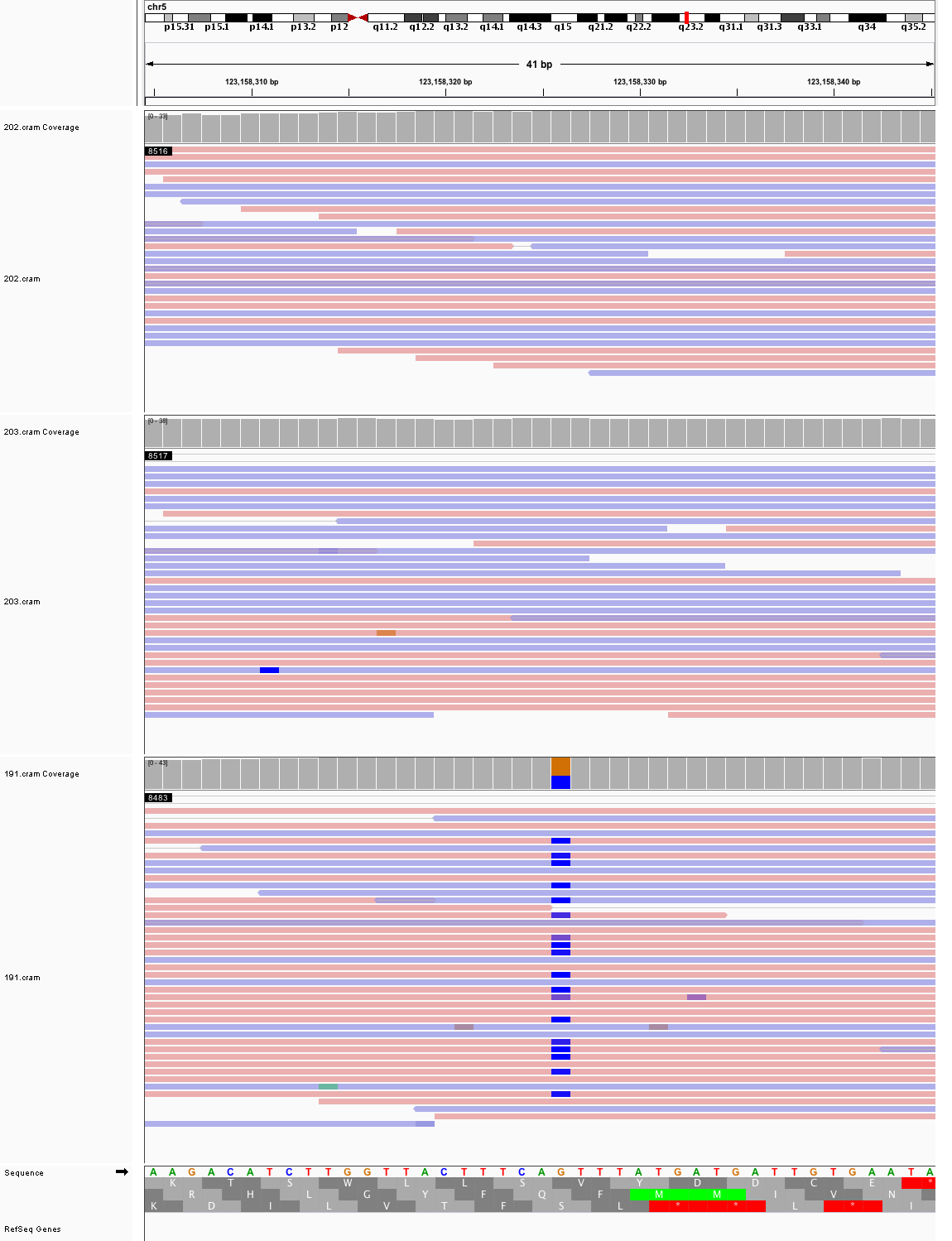

Supplement: Supplementary file 2. — In each image, the first two tracks contain alignments from the first-generation parents, and the third track contains the alignments for the second-generation child. Reads with mapping quality <20 are not included, as they were not considered by our variant calling pipeline, and mismatched bases are shaded by quality score (more transparent = lower base quality). [file elife-46922-supp2.zip › supp_file_2/chr5_123,158,305_123,158,345.png]

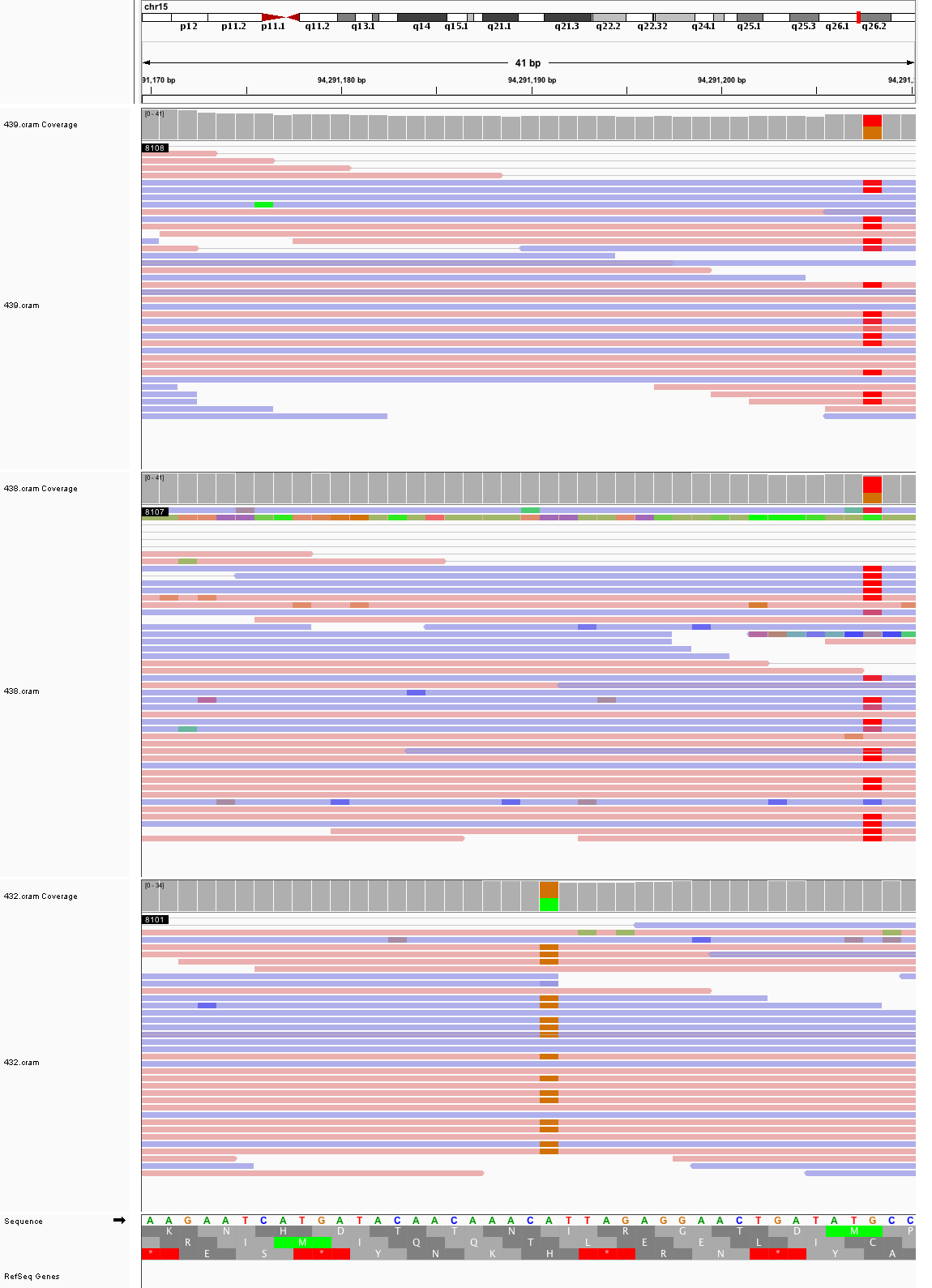

Supplement: Supplementary file 2. — In each image, the first two tracks contain alignments from the first-generation parents, and the third track contains the alignments for the second-generation child. Reads with mapping quality <20 are not included, as they were not considered by our variant calling pipeline, and mismatched bases are shaded by quality score (more transparent = lower base quality). [file elife-46922-supp2.zip › supp_file_2/chr15_94,291,170_94,291,210.png]

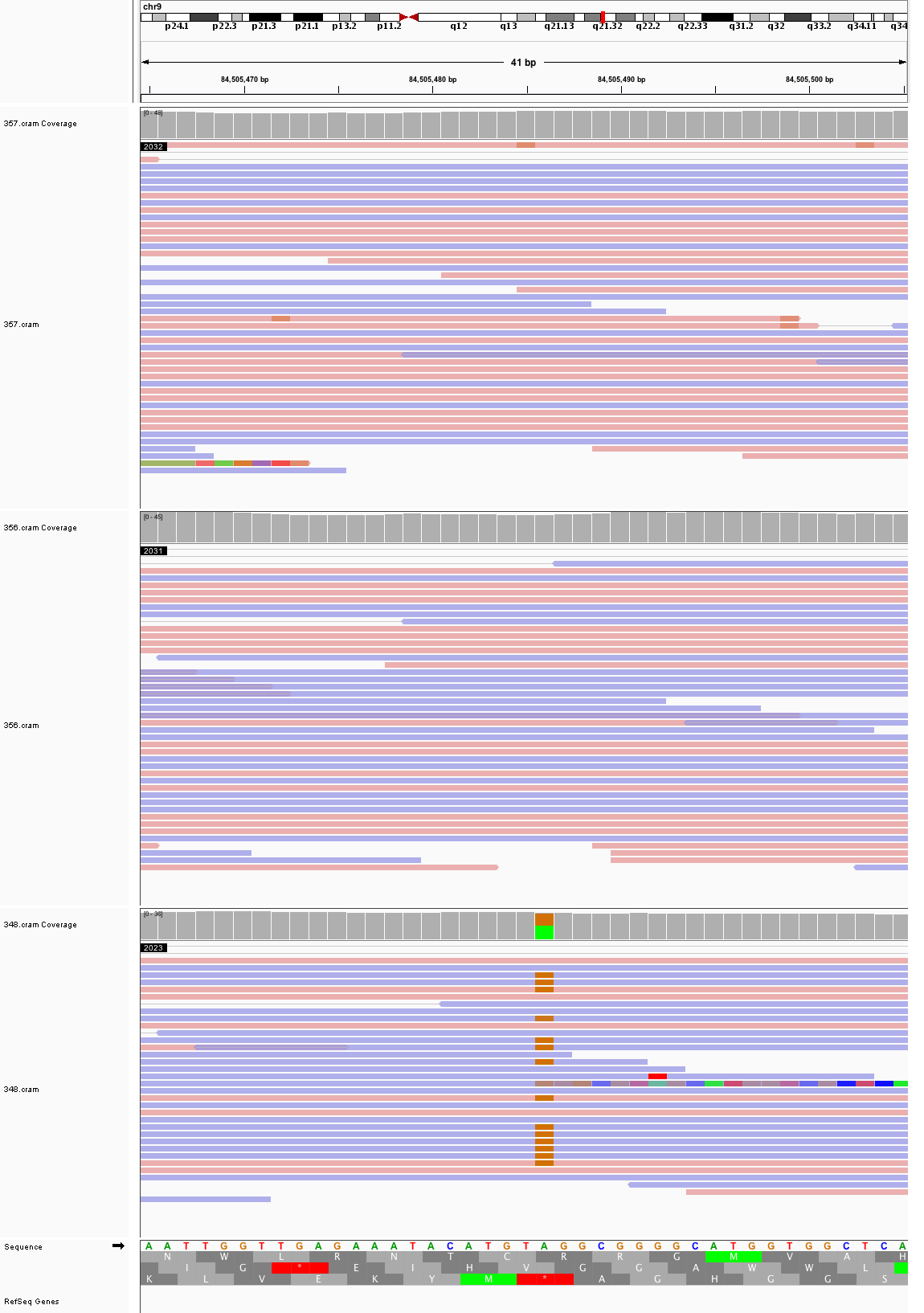

Supplement: Supplementary file 2. — In each image, the first two tracks contain alignments from the first-generation parents, and the third track contains the alignments for the second-generation child. Reads with mapping quality <20 are not included, as they were not considered by our variant calling pipeline, and mismatched bases are shaded by quality score (more transparent = lower base quality). [file elife-46922-supp2.zip › supp_file_2/chr9_84,505,465_84,505,505.png]

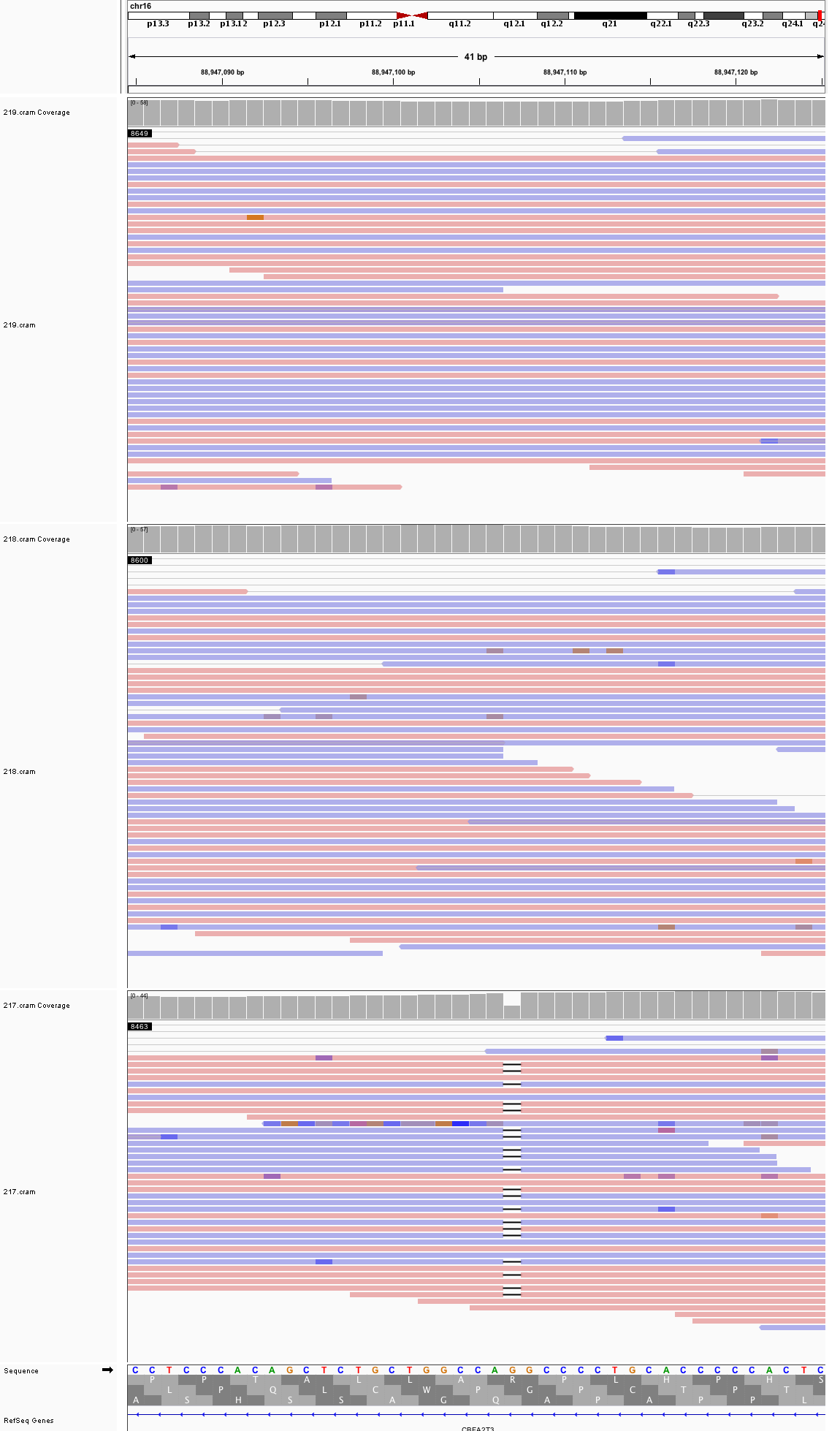

Supplement: Supplementary file 2. — In each image, the first two tracks contain alignments from the first-generation parents, and the third track contains the alignments for the second-generation child. Reads with mapping quality <20 are not included, as they were not considered by our variant calling pipeline, and mismatched bases are shaded by quality score (more transparent = lower base quality). [file elife-46922-supp2.zip › supp_file_2/chr16_88,947,085_88,947,125.png]

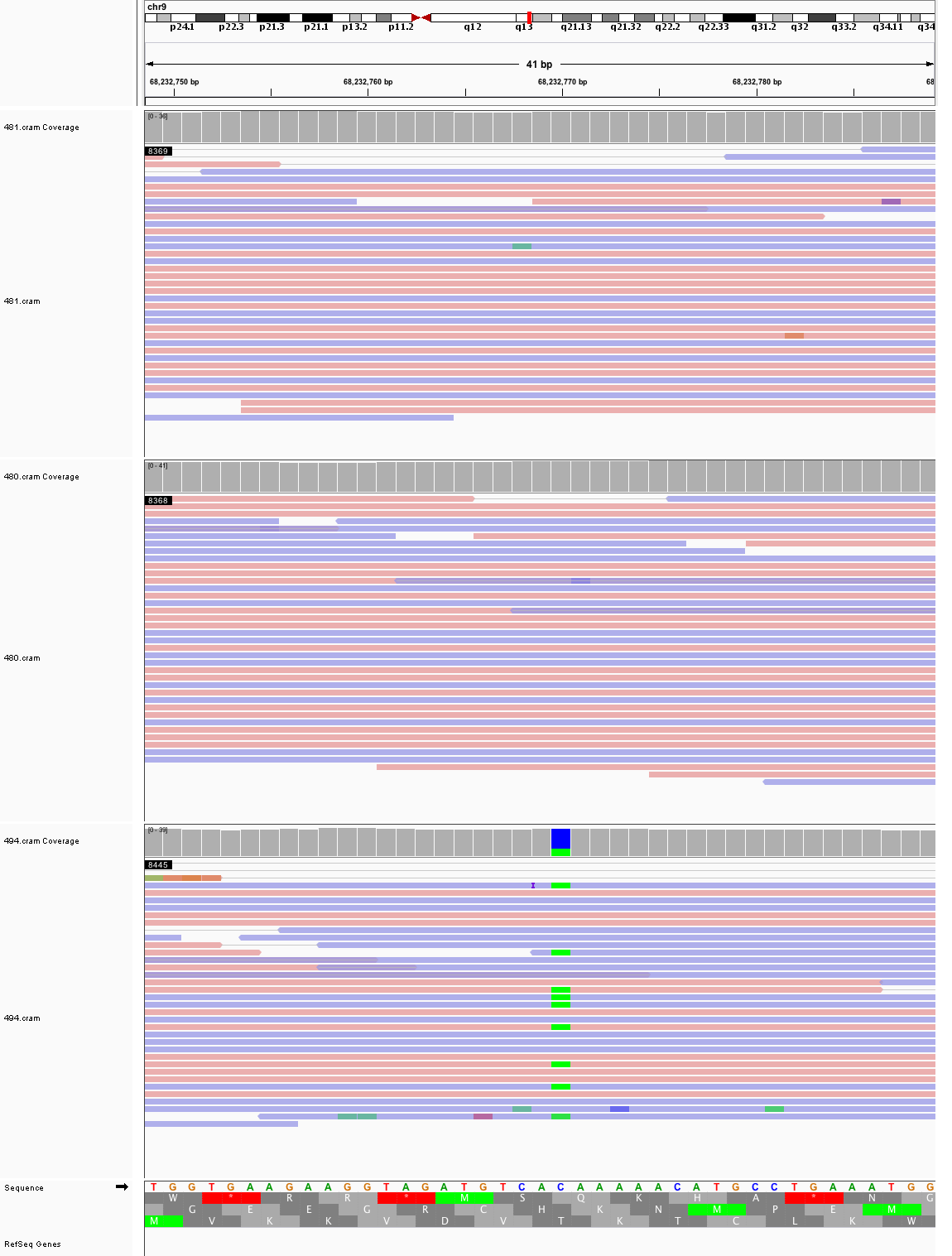

Supplement: Supplementary file 2. — In each image, the first two tracks contain alignments from the first-generation parents, and the third track contains the alignments for the second-generation child. Reads with mapping quality <20 are not included, as they were not considered by our variant calling pipeline, and mismatched bases are shaded by quality score (more transparent = lower base quality). [file elife-46922-supp2.zip › supp_file_2/chr9_68,232,749_68,232,789.png]

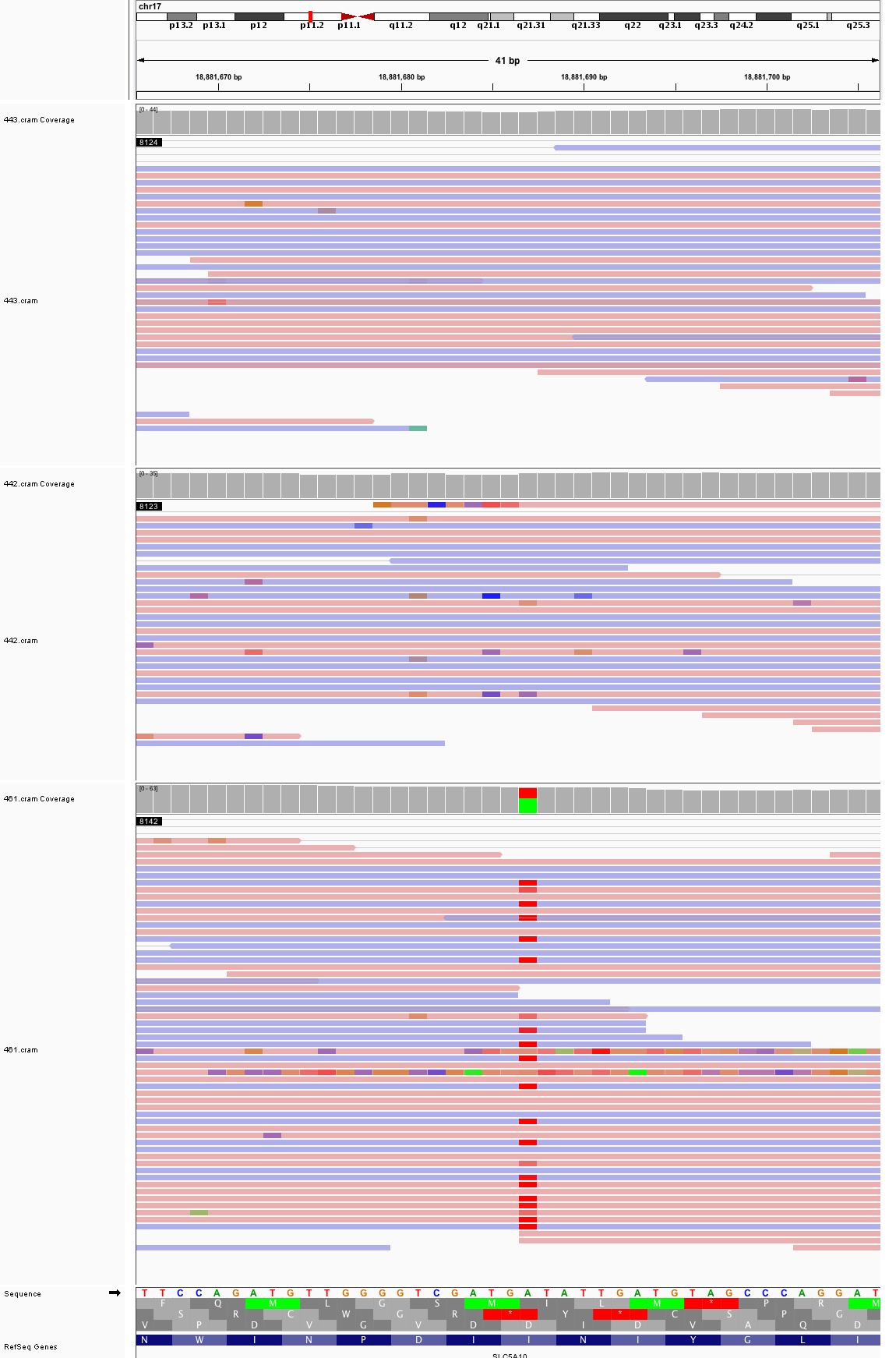

Supplement: Supplementary file 2. — In each image, the first two tracks contain alignments from the first-generation parents, and the third track contains the alignments for the second-generation child. Reads with mapping quality <20 are not included, as they were not considered by our variant calling pipeline, and mismatched bases are shaded by quality score (more transparent = lower base quality). [file elife-46922-supp2.zip › supp_file_2/chr17_18,881,666_18,881,706.png]

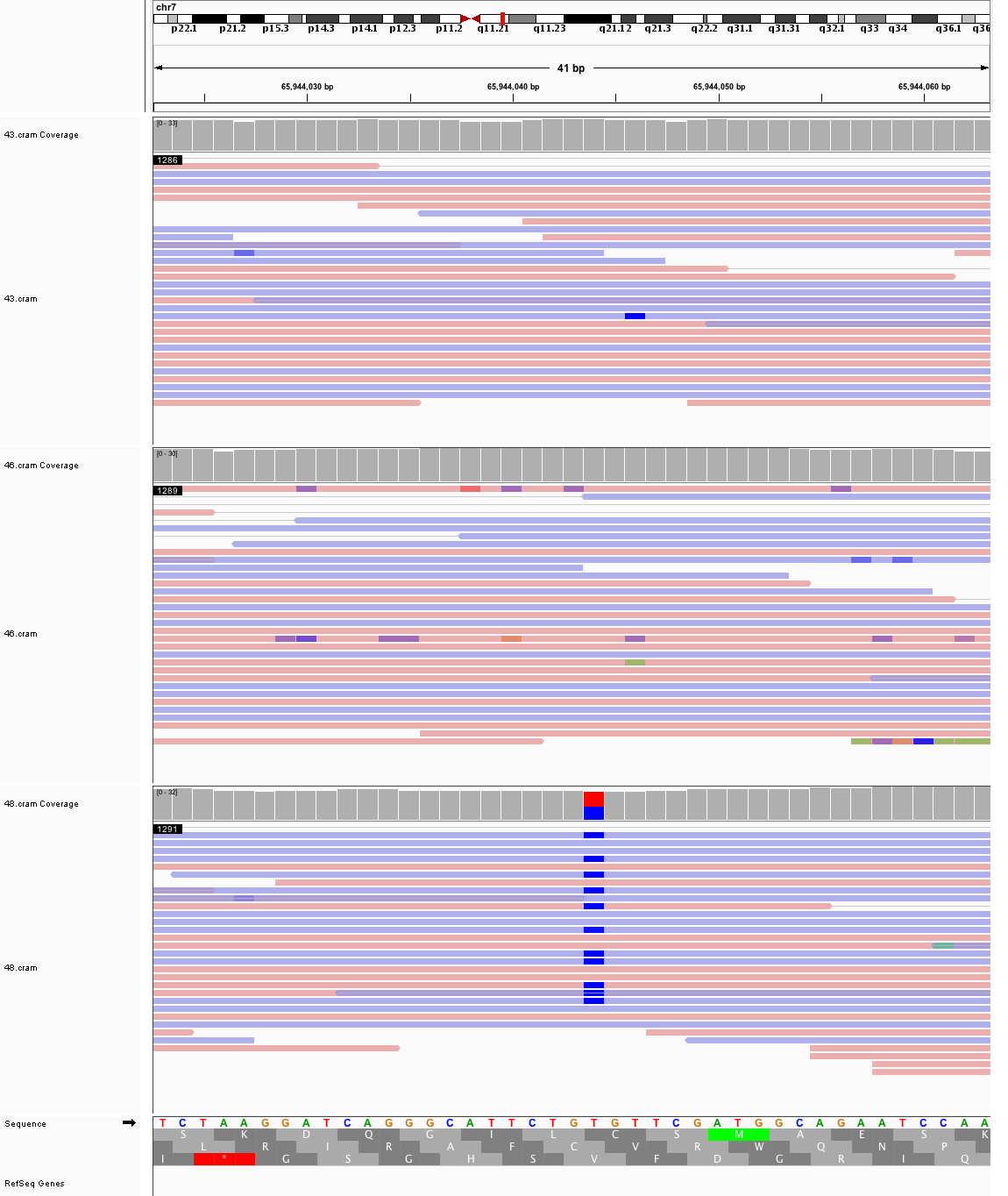

Supplement: Supplementary file 2. — In each image, the first two tracks contain alignments from the first-generation parents, and the third track contains the alignments for the second-generation child. Reads with mapping quality <20 are not included, as they were not considered by our variant calling pipeline, and mismatched bases are shaded by quality score (more transparent = lower base quality). [file elife-46922-supp2.zip › supp_file_2/chr7_65,944,023_65,944,063.png]

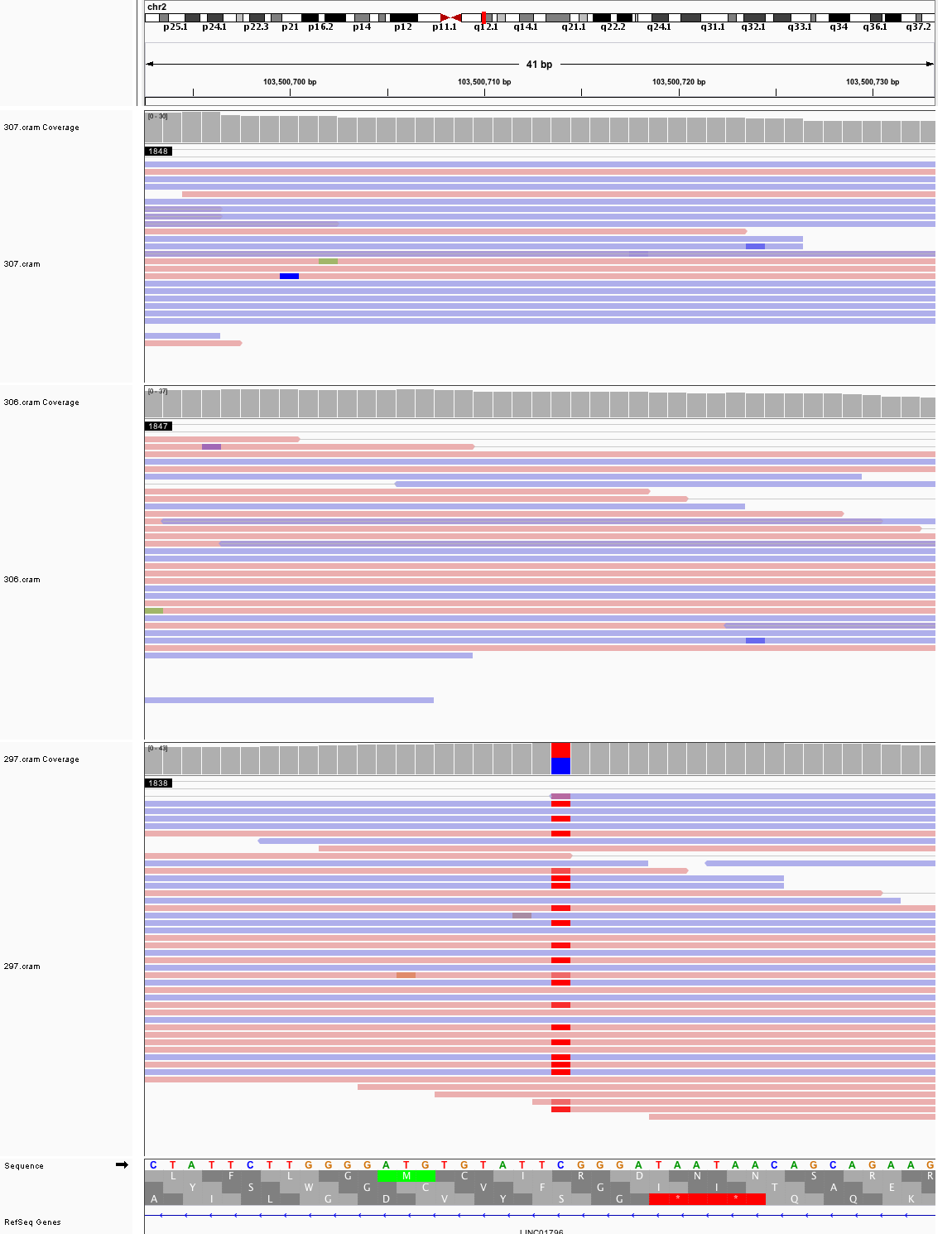

Supplement: Supplementary file 2. — In each image, the first two tracks contain alignments from the first-generation parents, and the third track contains the alignments for the second-generation child. Reads with mapping quality <20 are not included, as they were not considered by our variant calling pipeline, and mismatched bases are shaded by quality score (more transparent = lower base quality). [file elife-46922-supp2.zip › supp_file_2/chr2_103,500,693_103,500,733.png]

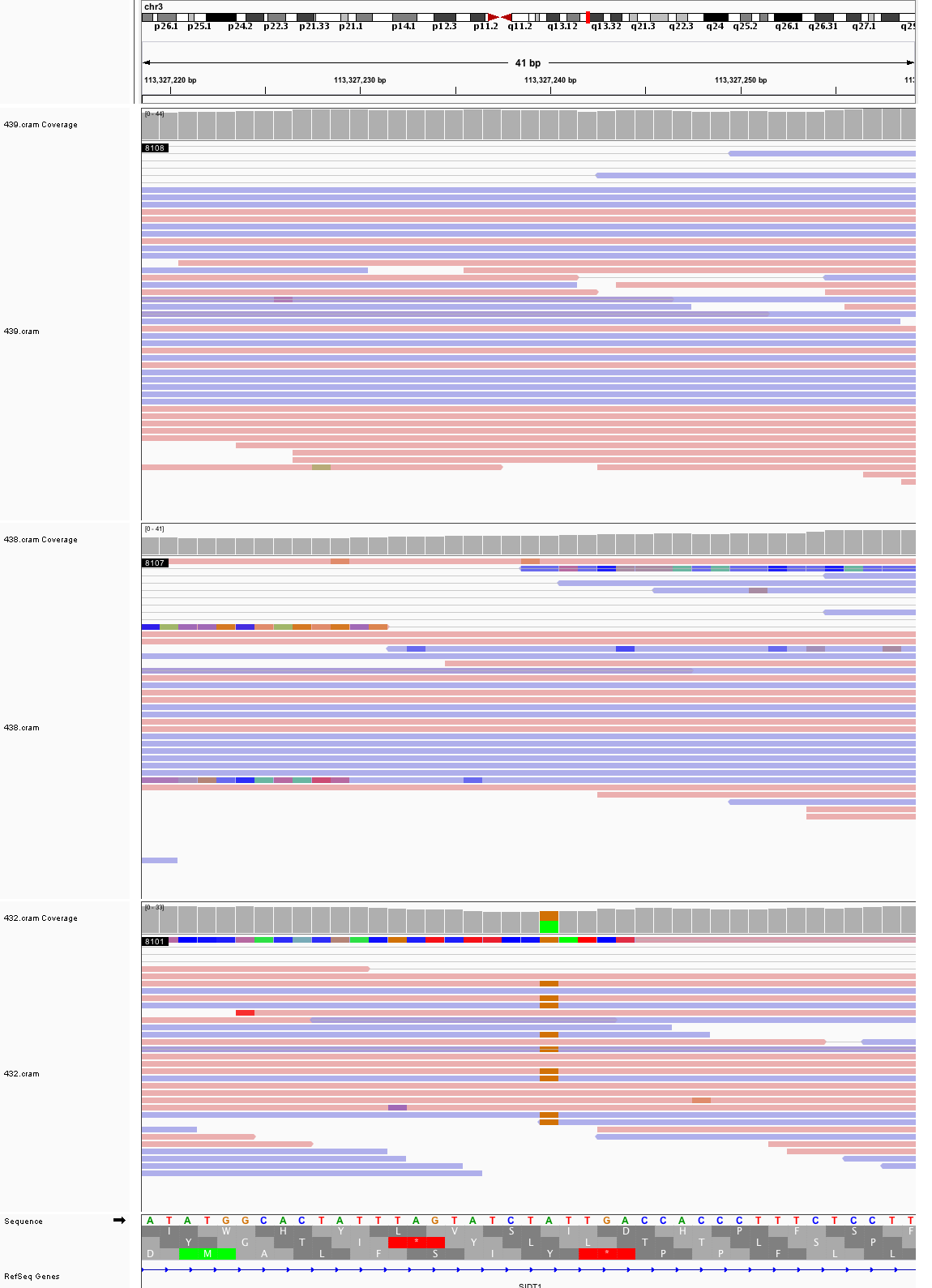

Supplement: Supplementary file 2. — In each image, the first two tracks contain alignments from the first-generation parents, and the third track contains the alignments for the second-generation child. Reads with mapping quality <20 are not included, as they were not considered by our variant calling pipeline, and mismatched bases are shaded by quality score (more transparent = lower base quality). [file elife-46922-supp2.zip › supp_file_2/chr3_113,327,219_113,327,259.png]

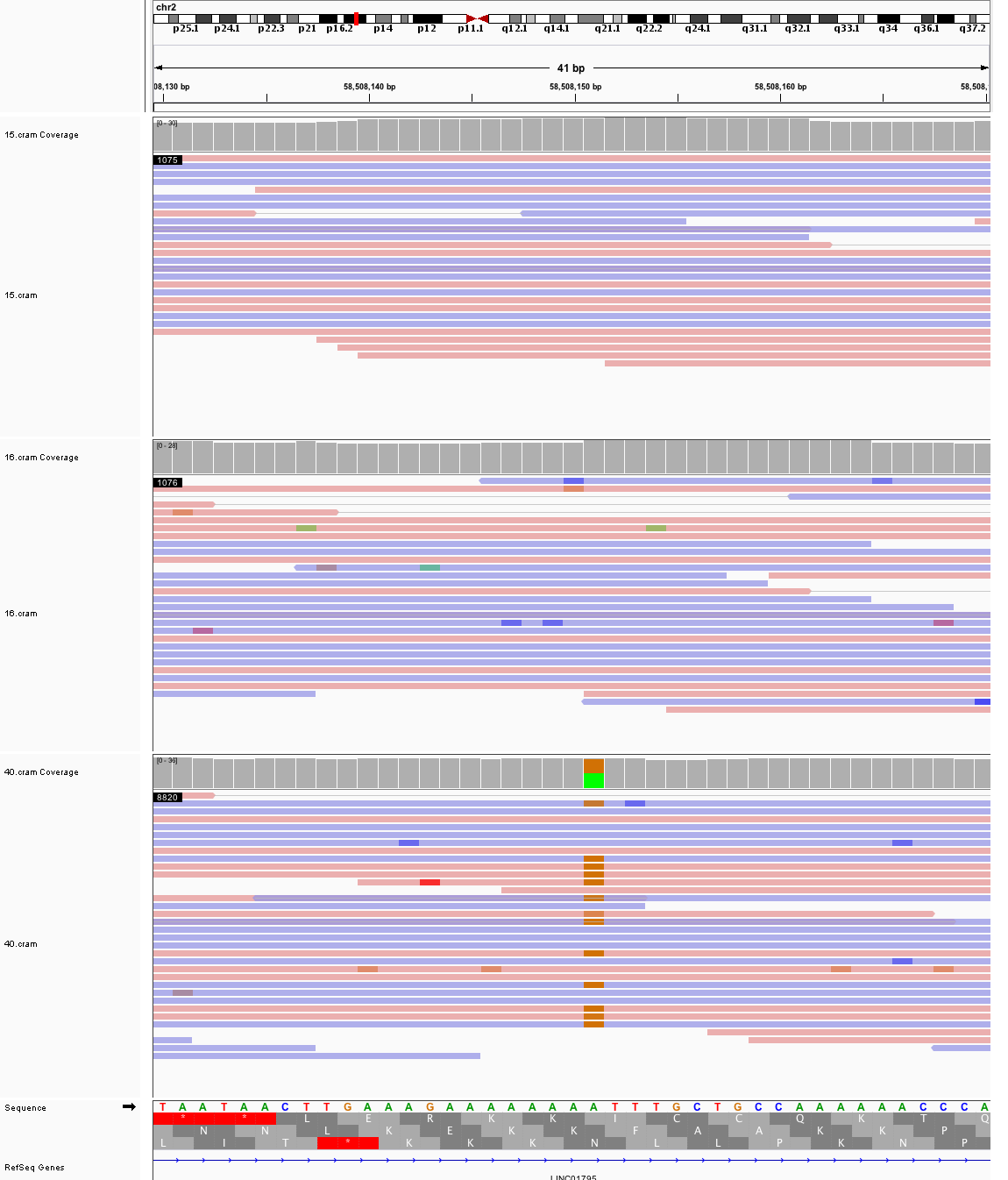

Supplement: Supplementary file 2. — In each image, the first two tracks contain alignments from the first-generation parents, and the third track contains the alignments for the second-generation child. Reads with mapping quality <20 are not included, as they were not considered by our variant calling pipeline, and mismatched bases are shaded by quality score (more transparent = lower base quality). [file elife-46922-supp2.zip › supp_file_2/chr2_58,508,130_58,508,170.png]

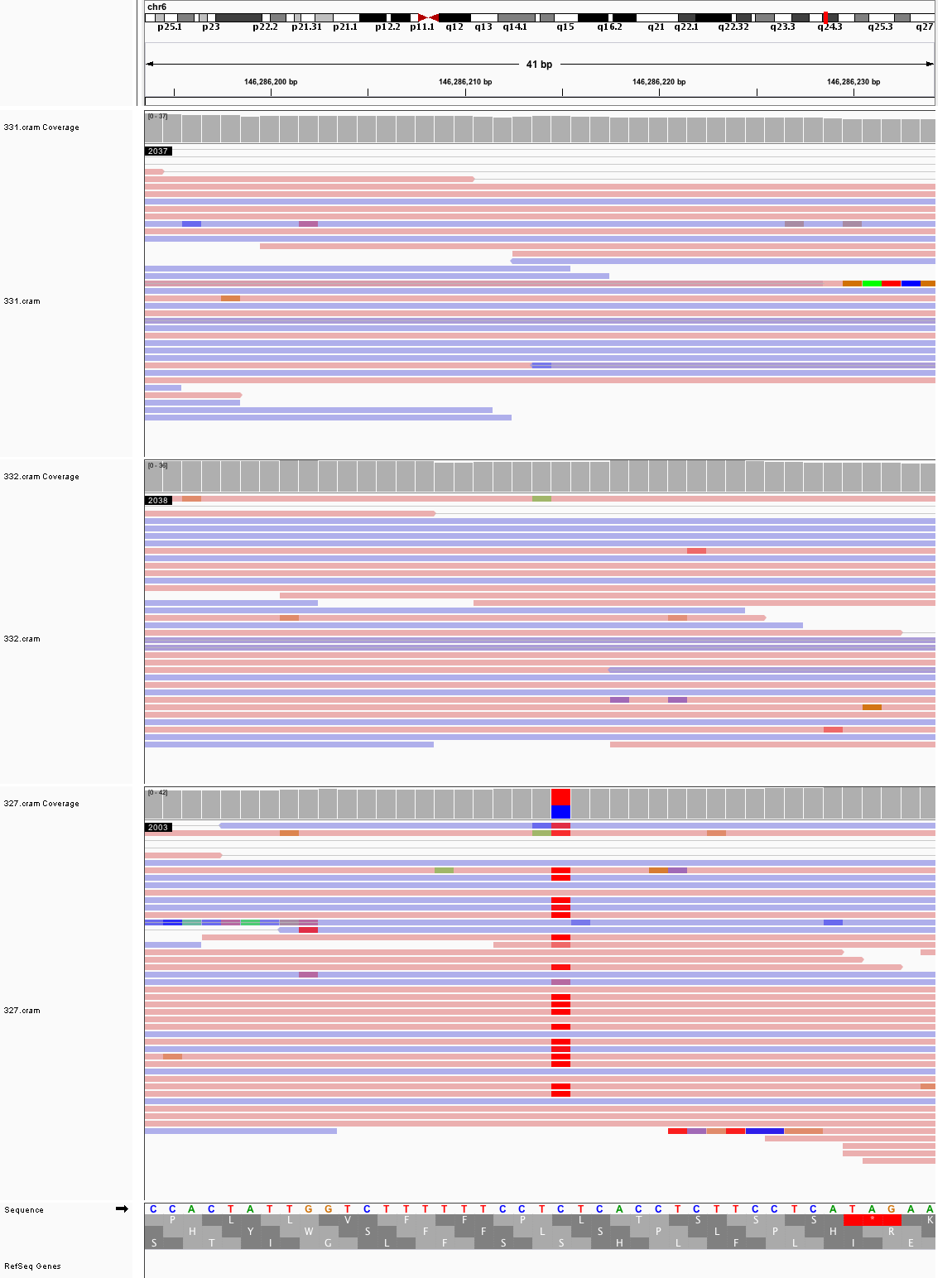

Supplement: Supplementary file 2. — In each image, the first two tracks contain alignments from the first-generation parents, and the third track contains the alignments for the second-generation child. Reads with mapping quality <20 are not included, as they were not considered by our variant calling pipeline, and mismatched bases are shaded by quality score (more transparent = lower base quality). [file elife-46922-supp2.zip › supp_file_2/chr6_146,286,194_146,286,234.png]

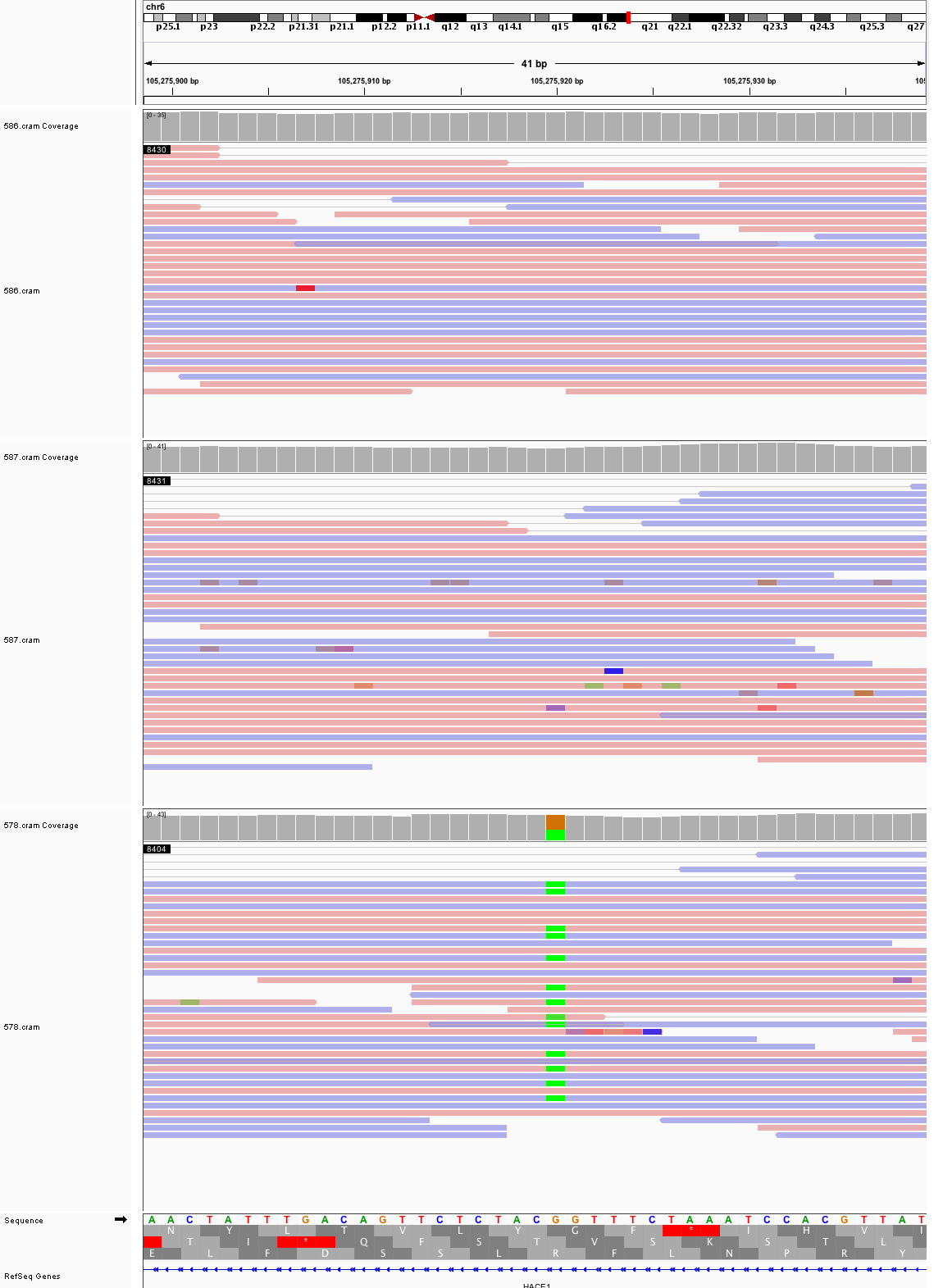

Supplement: Supplementary file 2. — In each image, the first two tracks contain alignments from the first-generation parents, and the third track contains the alignments for the second-generation child. Reads with mapping quality <20 are not included, as they were not considered by our variant calling pipeline, and mismatched bases are shaded by quality score (more transparent = lower base quality). [file elife-46922-supp2.zip › supp_file_2/chr6_105,275,899_105,275,939.png]

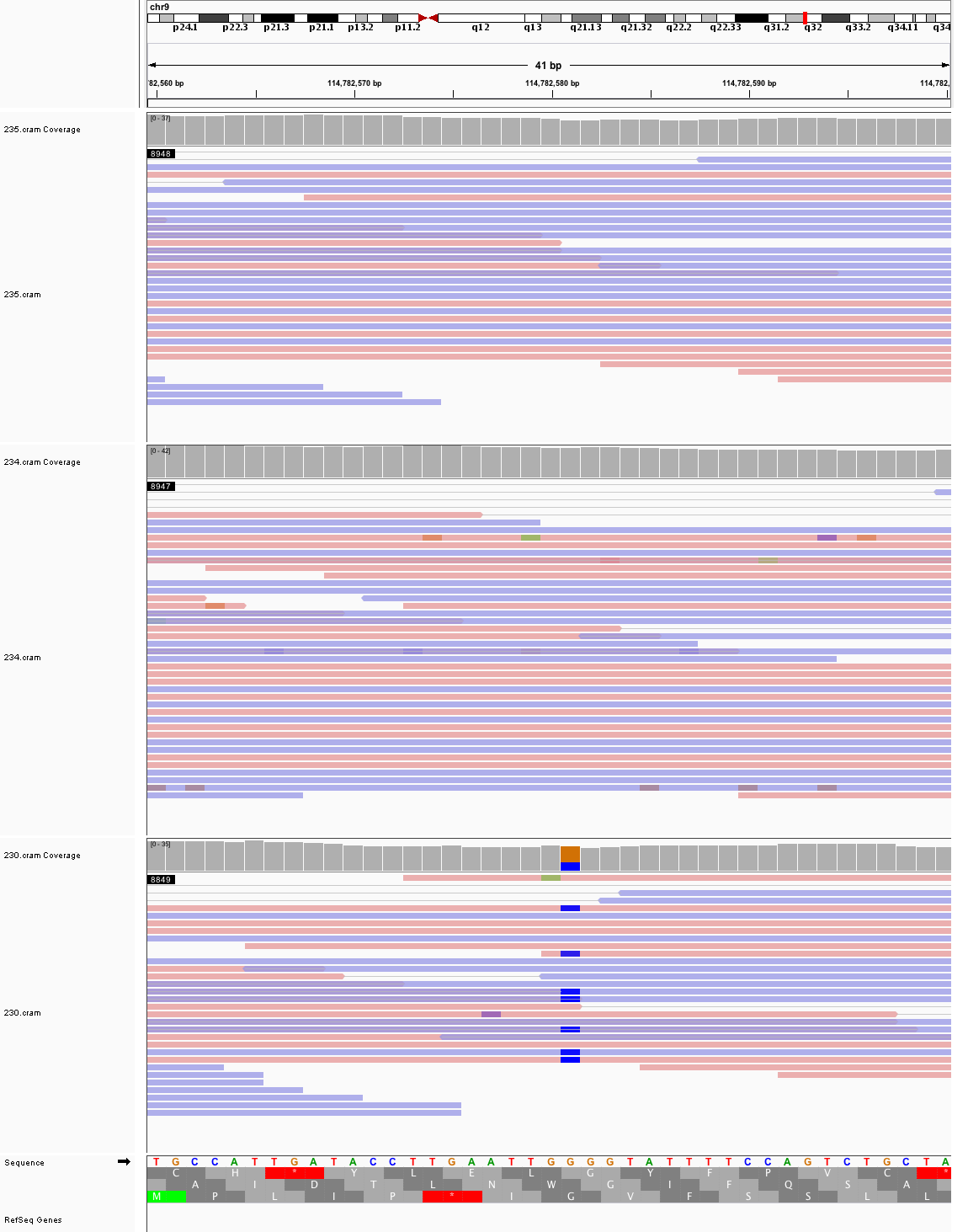

Supplement: Supplementary file 2. — In each image, the first two tracks contain alignments from the first-generation parents, and the third track contains the alignments for the second-generation child. Reads with mapping quality <20 are not included, as they were not considered by our variant calling pipeline, and mismatched bases are shaded by quality score (more transparent = lower base quality). [file elife-46922-supp2.zip › supp_file_2/chr9_114,782,560_114,782,600.png]

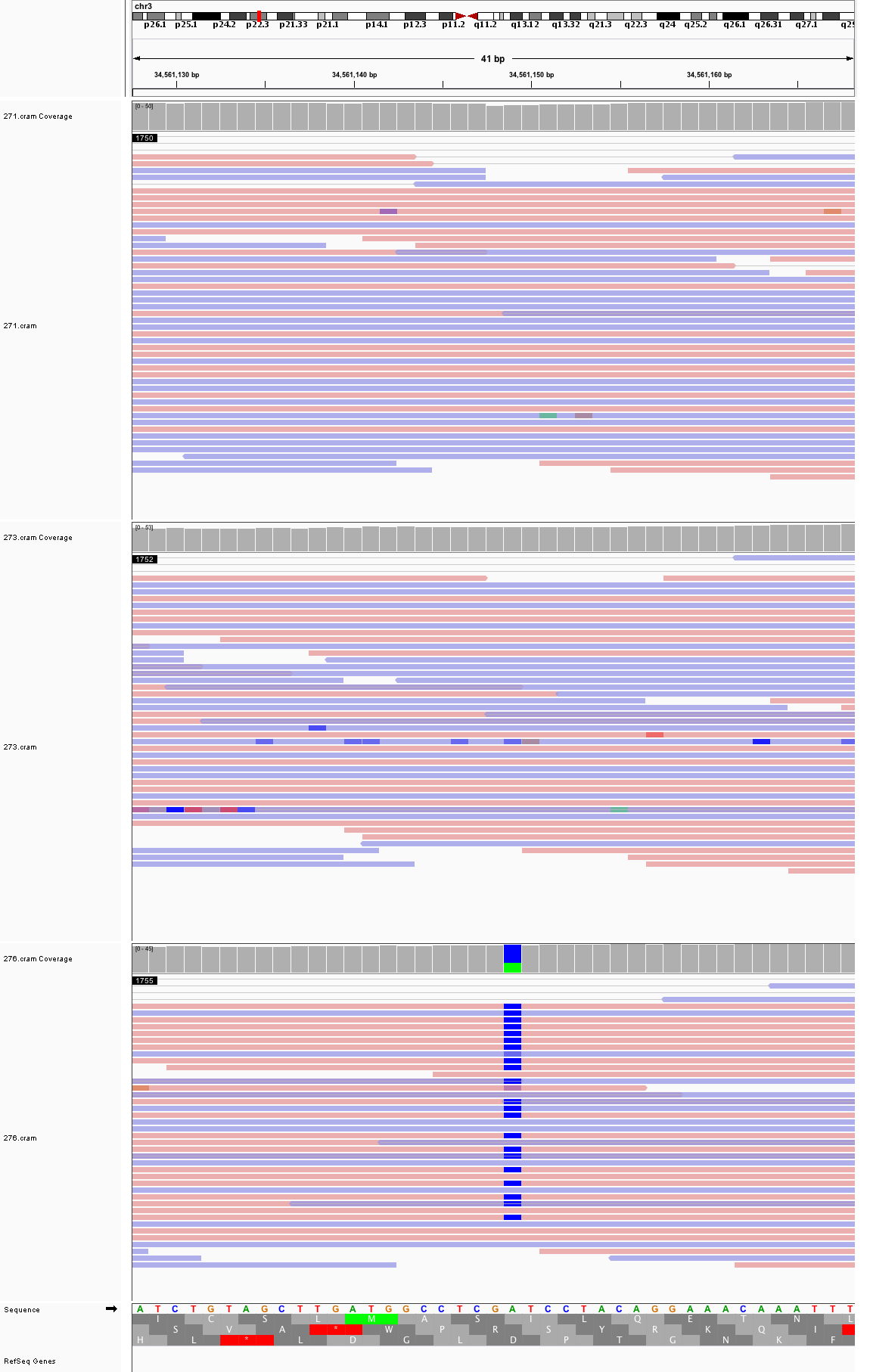

Supplement: Supplementary file 2. — In each image, the first two tracks contain alignments from the first-generation parents, and the third track contains the alignments for the second-generation child. Reads with mapping quality <20 are not included, as they were not considered by our variant calling pipeline, and mismatched bases are shaded by quality score (more transparent = lower base quality). [file elife-46922-supp2.zip › supp_file_2/chr3_34,561,128_34,561,168.png]

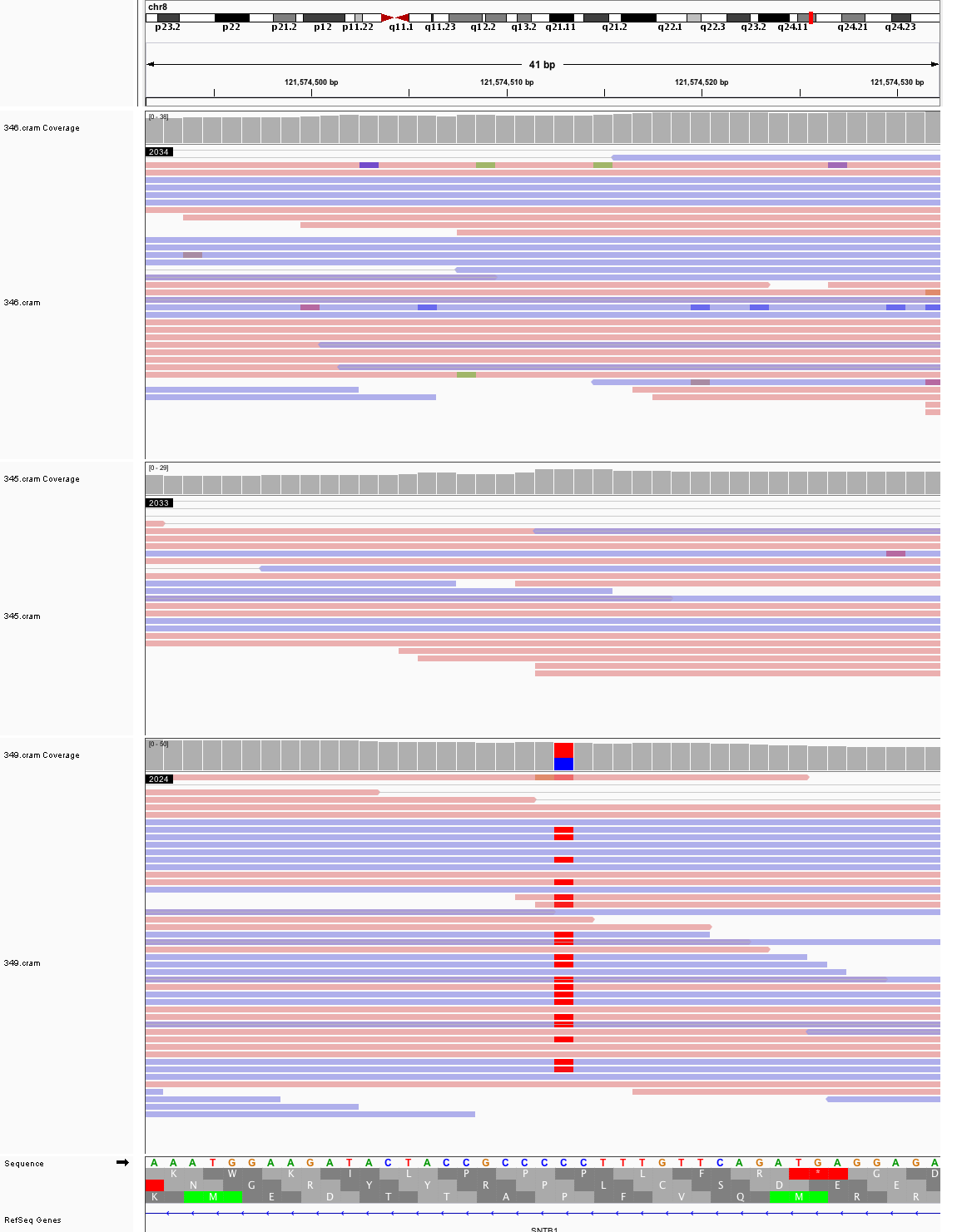

Supplement: Supplementary file 2. — In each image, the first two tracks contain alignments from the first-generation parents, and the third track contains the alignments for the second-generation child. Reads with mapping quality <20 are not included, as they were not considered by our variant calling pipeline, and mismatched bases are shaded by quality score (more transparent = lower base quality). [file elife-46922-supp2.zip › supp_file_2/chr8_121,574,492_121,574,532.png]

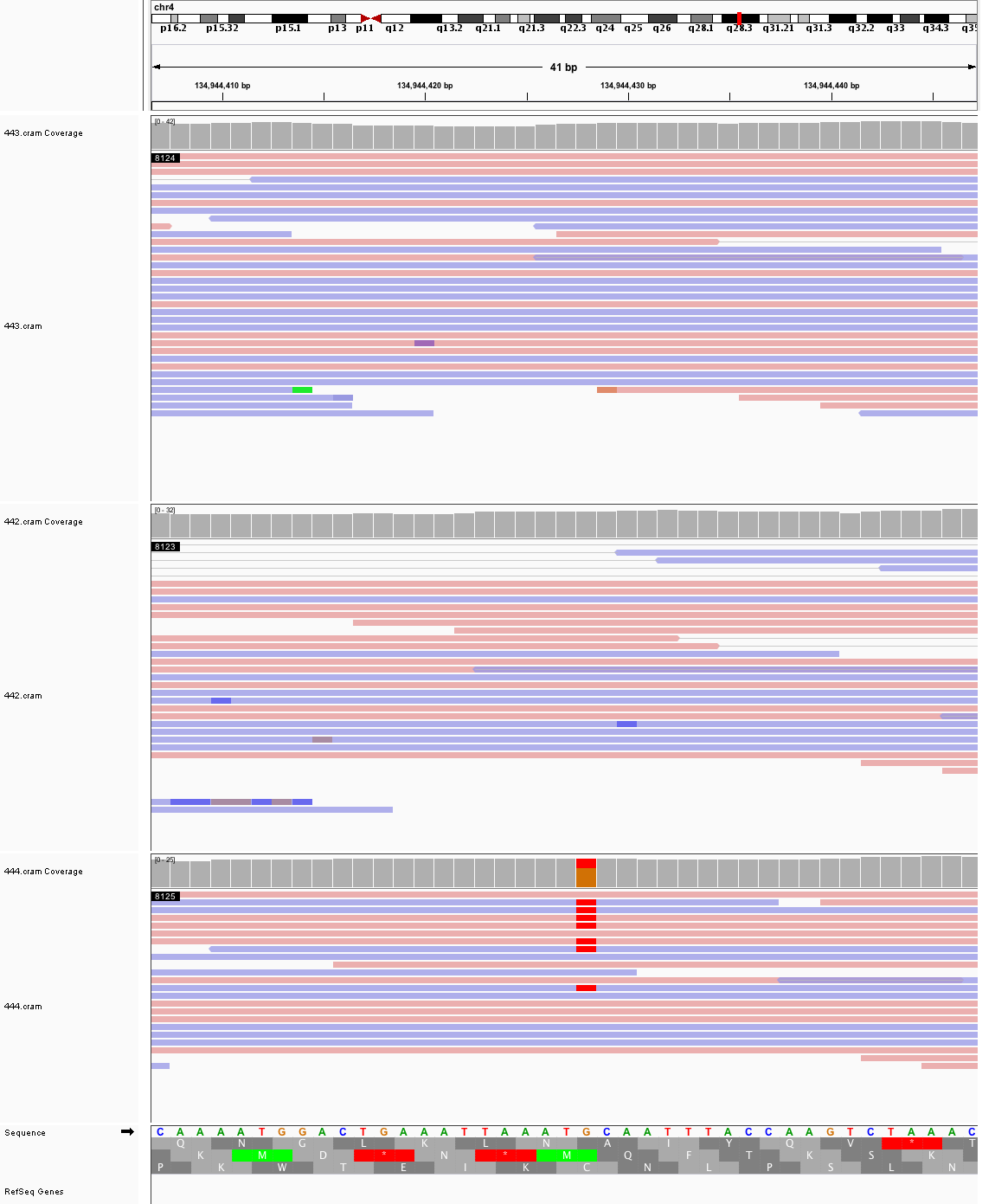

Supplement: Supplementary file 2. — In each image, the first two tracks contain alignments from the first-generation parents, and the third track contains the alignments for the second-generation child. Reads with mapping quality <20 are not included, as they were not considered by our variant calling pipeline, and mismatched bases are shaded by quality score (more transparent = lower base quality). [file elife-46922-supp2.zip › supp_file_2/chr4_134,944,407_134,944,447.png]

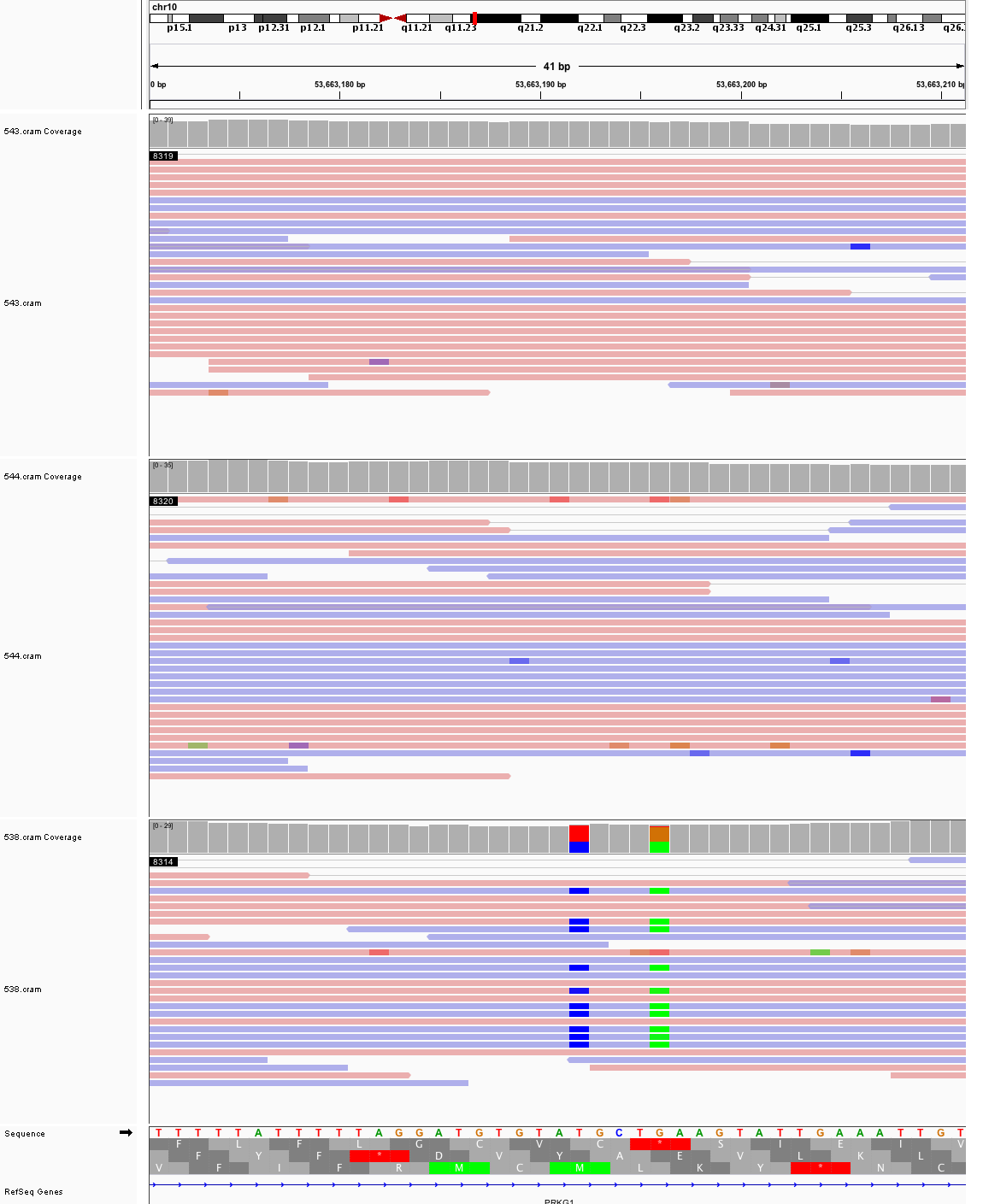

Supplement: Supplementary file 2. — In each image, the first two tracks contain alignments from the first-generation parents, and the third track contains the alignments for the second-generation child. Reads with mapping quality <20 are not included, as they were not considered by our variant calling pipeline, and mismatched bases are shaded by quality score (more transparent = lower base quality). [file elife-46922-supp2.zip › supp_file_2/chr10_53,663,171_53,663,211.png]

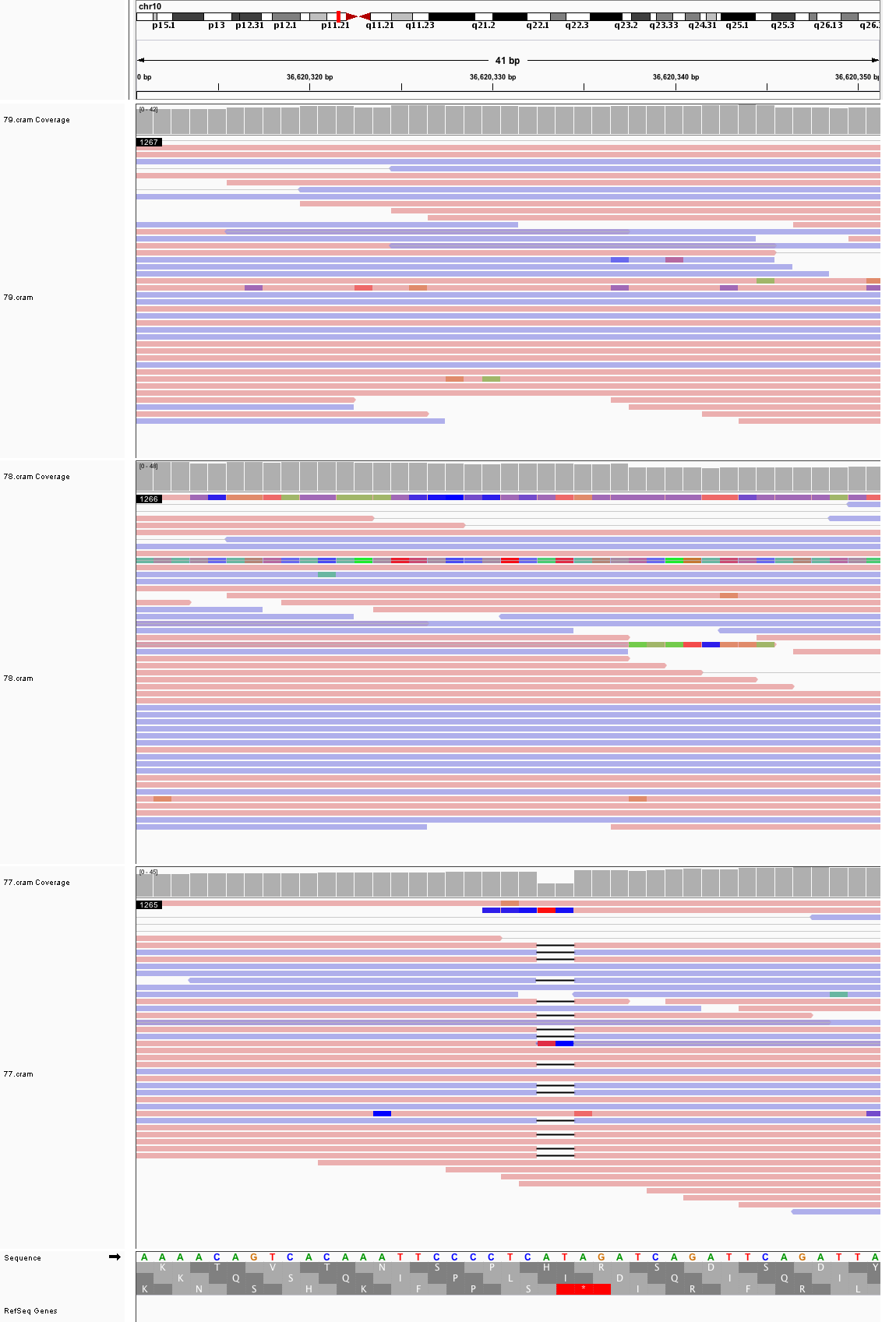

Supplement: Supplementary file 2. — In each image, the first two tracks contain alignments from the first-generation parents, and the third track contains the alignments for the second-generation child. Reads with mapping quality <20 are not included, as they were not considered by our variant calling pipeline, and mismatched bases are shaded by quality score (more transparent = lower base quality). [file elife-46922-supp2.zip › supp_file_2/chr10_36,620,311_36,620,351.png]

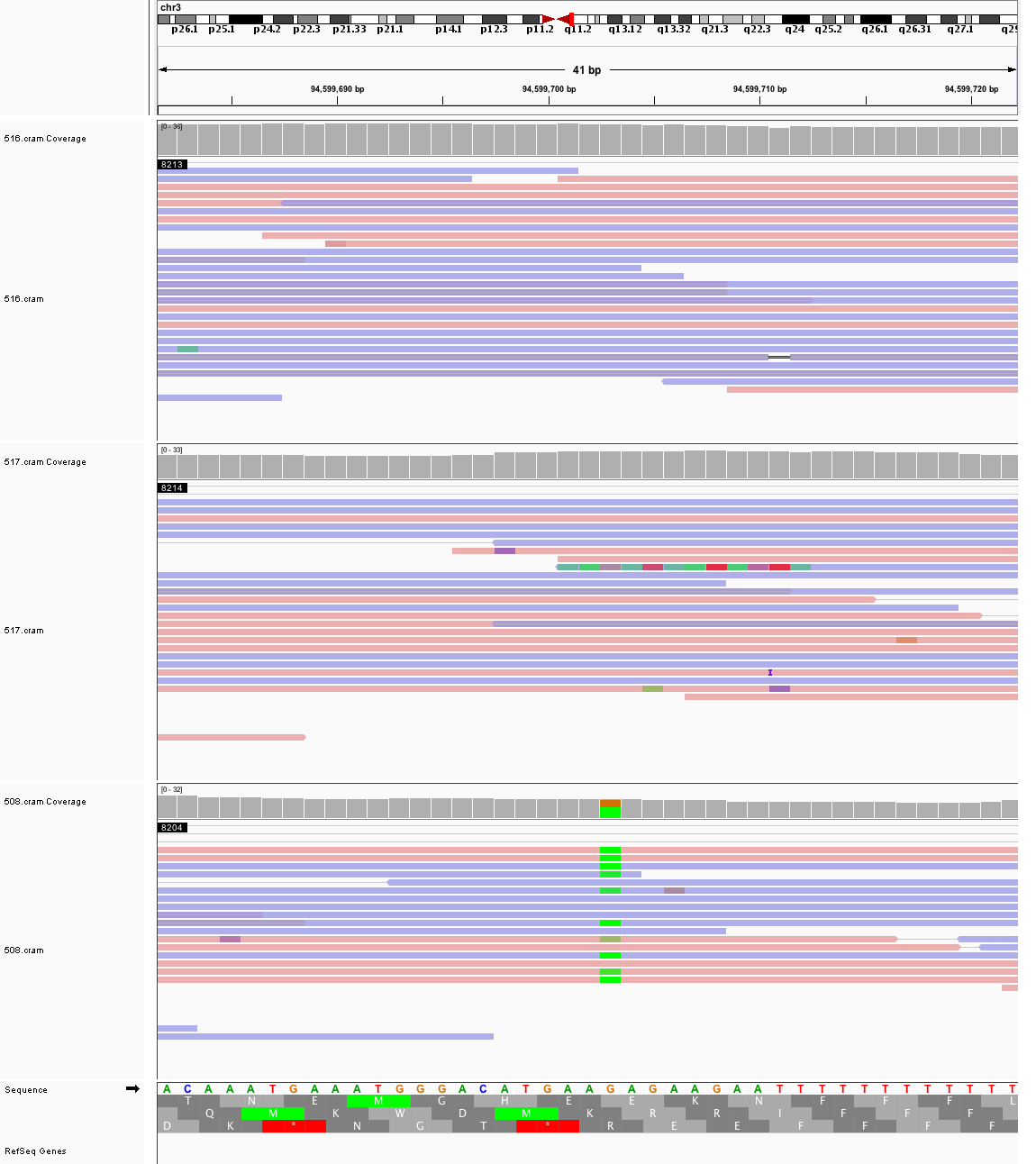

Supplement: Supplementary file 2. — In each image, the first two tracks contain alignments from the first-generation parents, and the third track contains the alignments for the second-generation child. Reads with mapping quality <20 are not included, as they were not considered by our variant calling pipeline, and mismatched bases are shaded by quality score (more transparent = lower base quality). [file elife-46922-supp2.zip › supp_file_2/chr3_94,599,682_94,599,722.png]

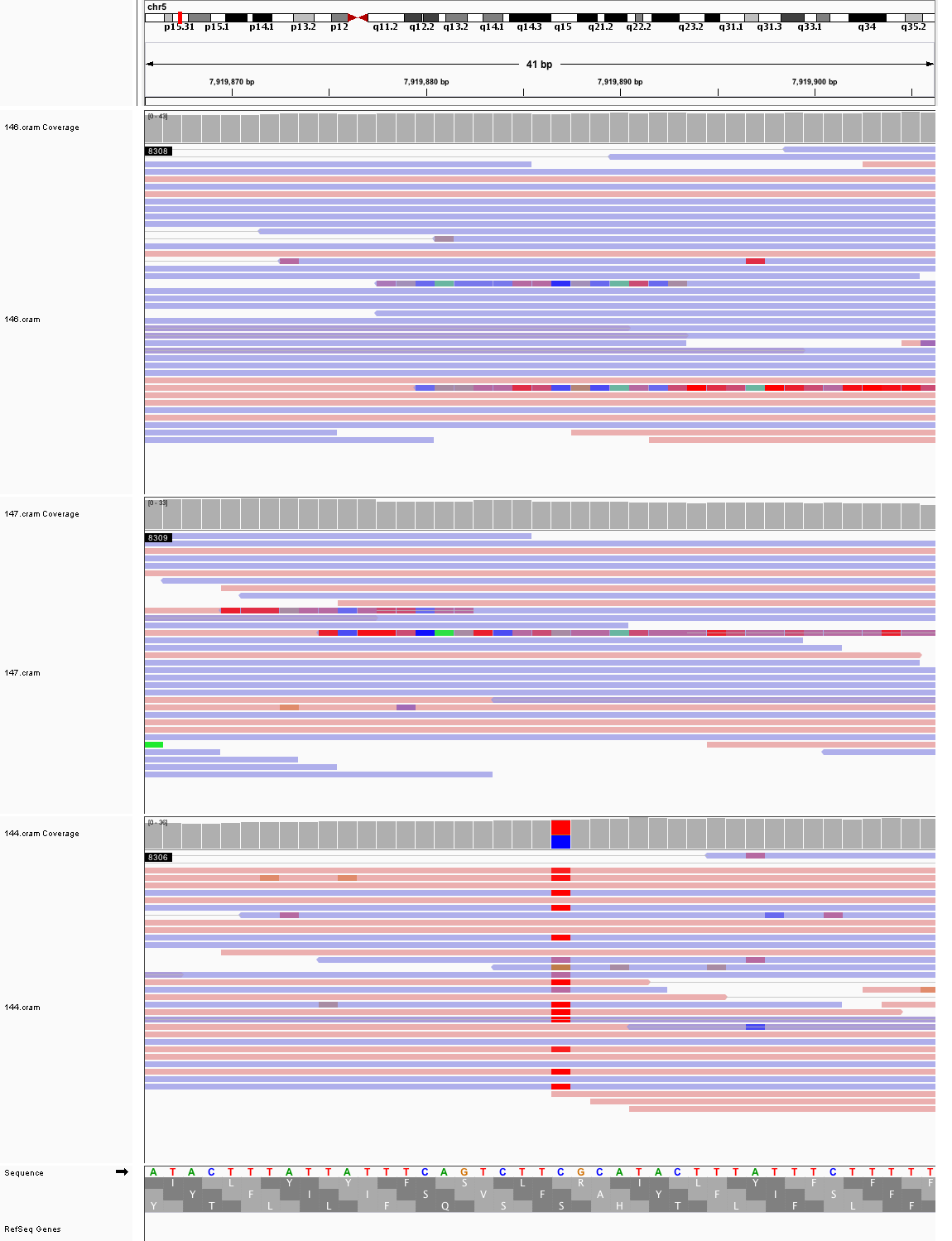

Supplement: Supplementary file 2. — In each image, the first two tracks contain alignments from the first-generation parents, and the third track contains the alignments for the second-generation child. Reads with mapping quality <20 are not included, as they were not considered by our variant calling pipeline, and mismatched bases are shaded by quality score (more transparent = lower base quality). [file elife-46922-supp2.zip › supp_file_2/chr5_7,919,866_7,919,906.png]

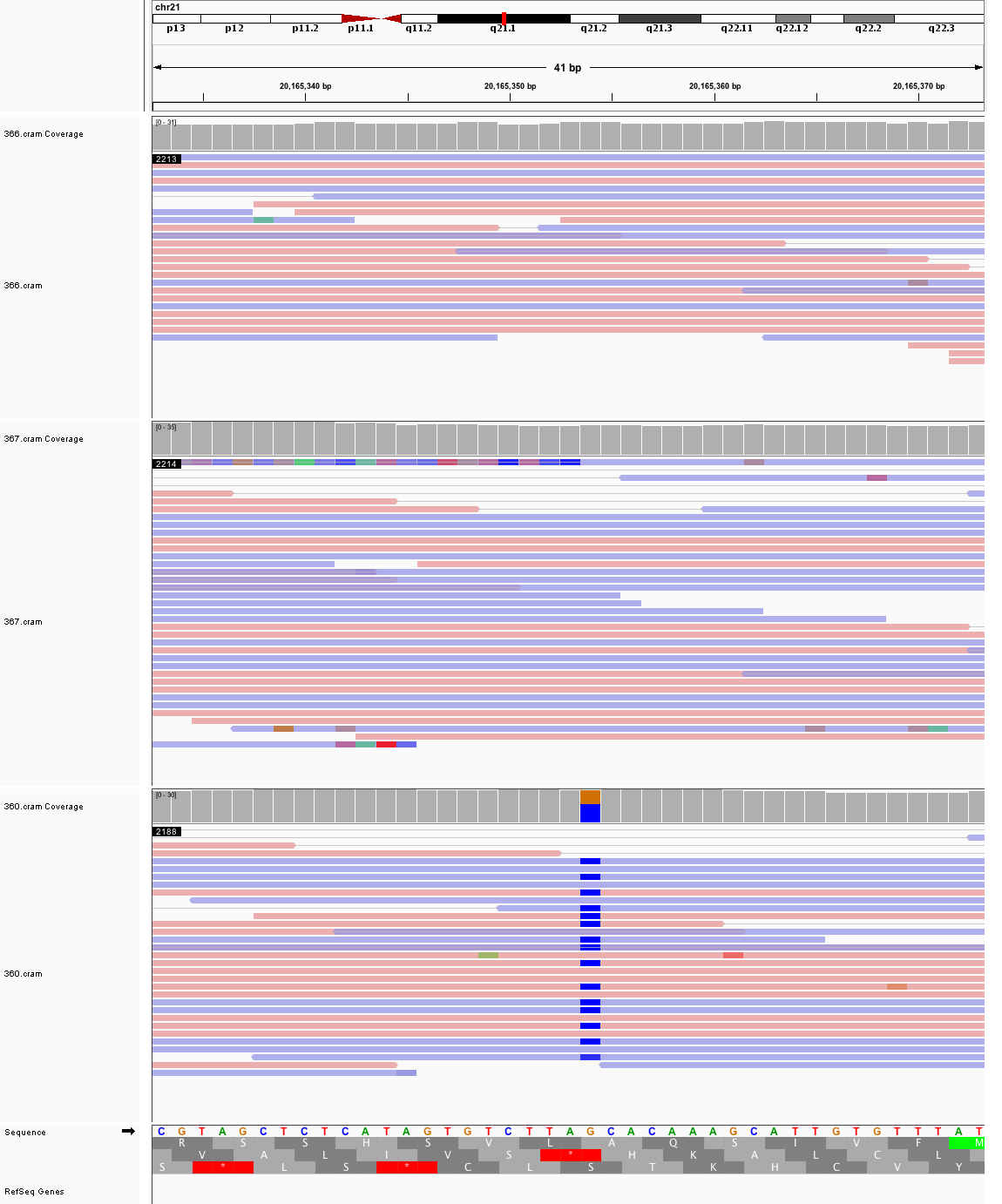

Supplement: Supplementary file 2. — In each image, the first two tracks contain alignments from the first-generation parents, and the third track contains the alignments for the second-generation child. Reads with mapping quality <20 are not included, as they were not considered by our variant calling pipeline, and mismatched bases are shaded by quality score (more transparent = lower base quality). [file elife-46922-supp2.zip › supp_file_2/chr21_20,165,333_20,165,373.png]

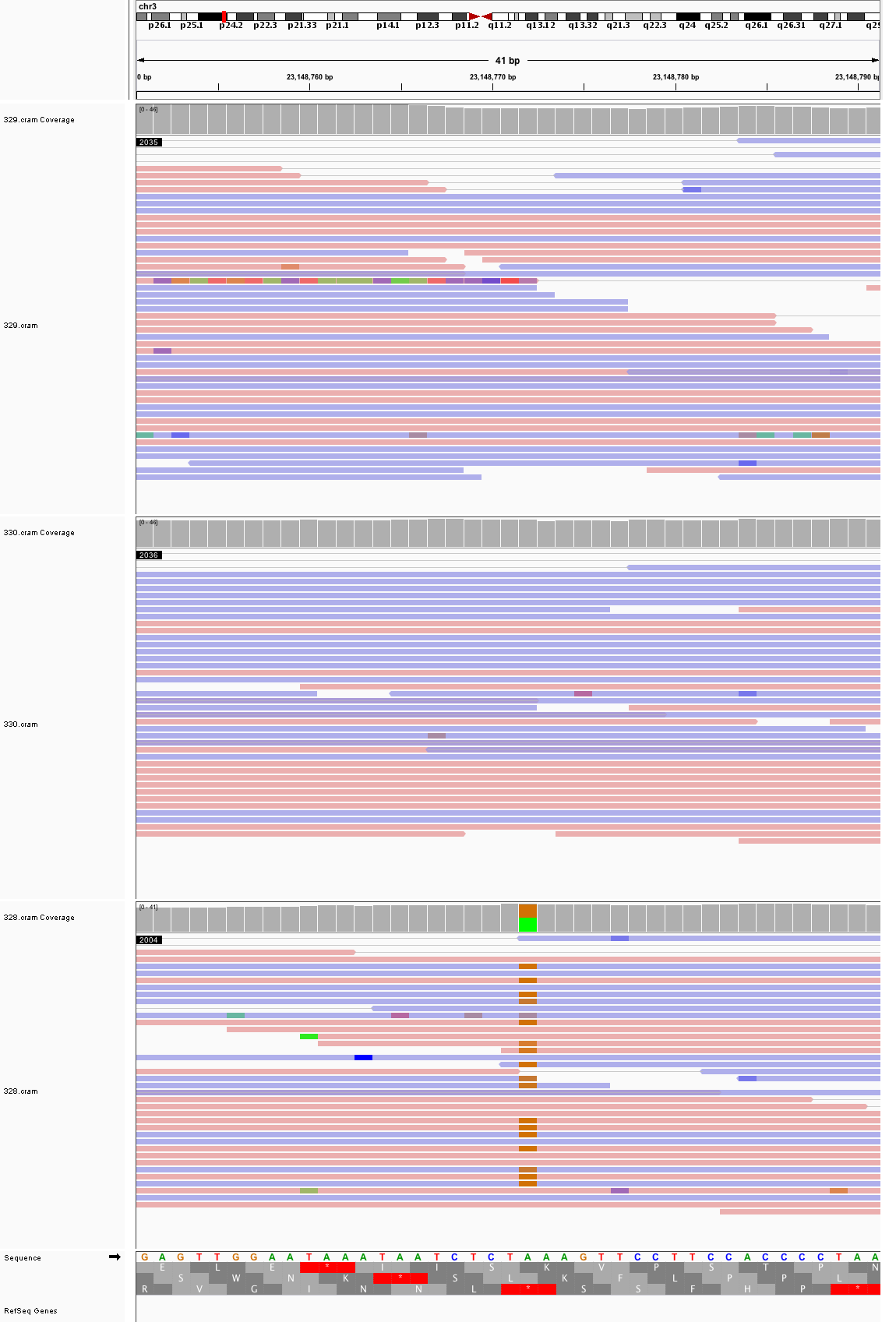

Supplement: Supplementary file 2. — In each image, the first two tracks contain alignments from the first-generation parents, and the third track contains the alignments for the second-generation child. Reads with mapping quality <20 are not included, as they were not considered by our variant calling pipeline, and mismatched bases are shaded by quality score (more transparent = lower base quality). [file elife-46922-supp2.zip › supp_file_2/chr3_23,148,751_23,148,791.png]

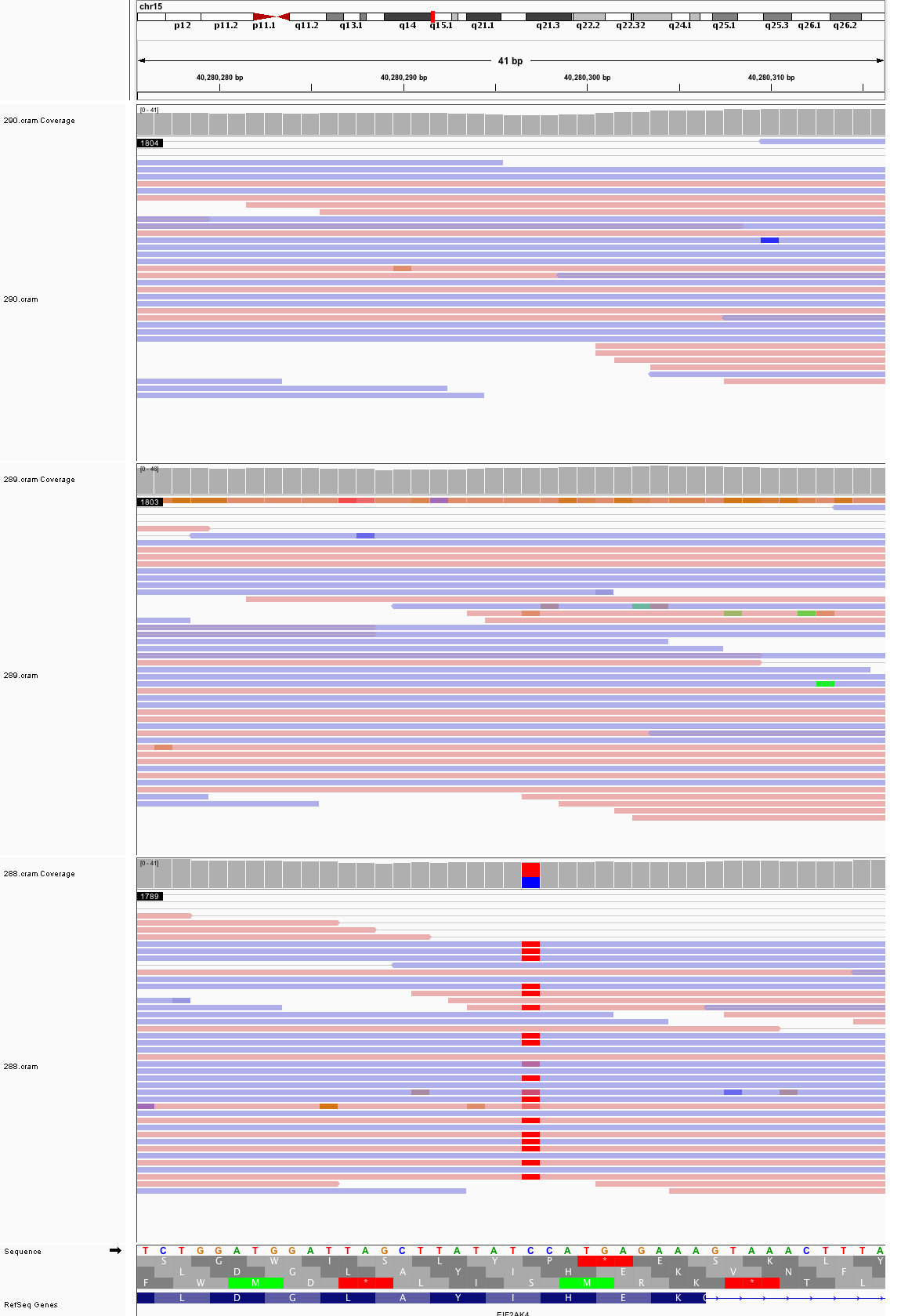

Supplement: Supplementary file 2. — In each image, the first two tracks contain alignments from the first-generation parents, and the third track contains the alignments for the second-generation child. Reads with mapping quality <20 are not included, as they were not considered by our variant calling pipeline, and mismatched bases are shaded by quality score (more transparent = lower base quality). [file elife-46922-supp2.zip › supp_file_2/chr15_40,280,276_40,280,316.png]

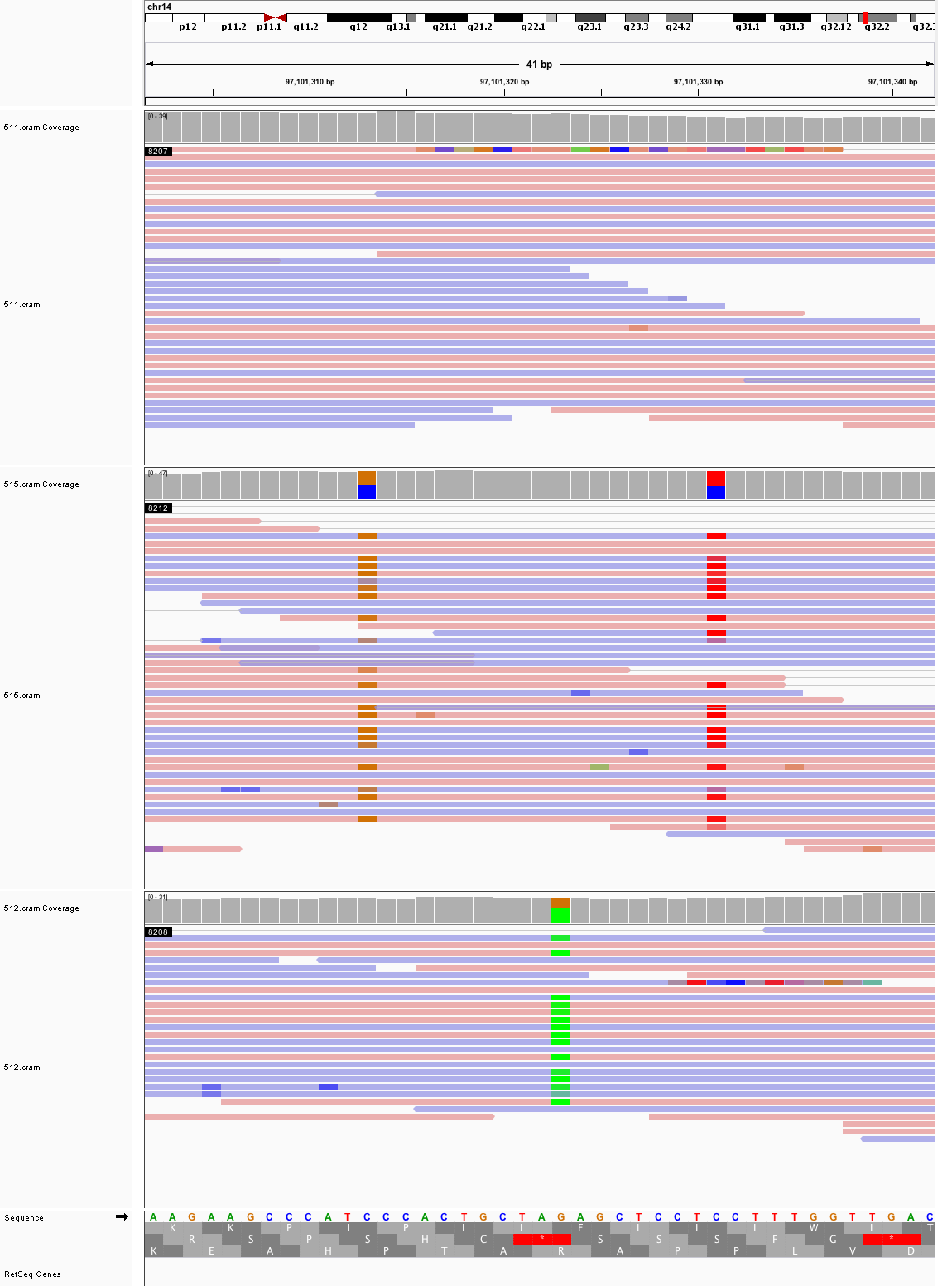

Supplement: Supplementary file 2. — In each image, the first two tracks contain alignments from the first-generation parents, and the third track contains the alignments for the second-generation child. Reads with mapping quality <20 are not included, as they were not considered by our variant calling pipeline, and mismatched bases are shaded by quality score (more transparent = lower base quality). [file elife-46922-supp2.zip › supp_file_2/chr14_97,101,302_97,101,342.png]

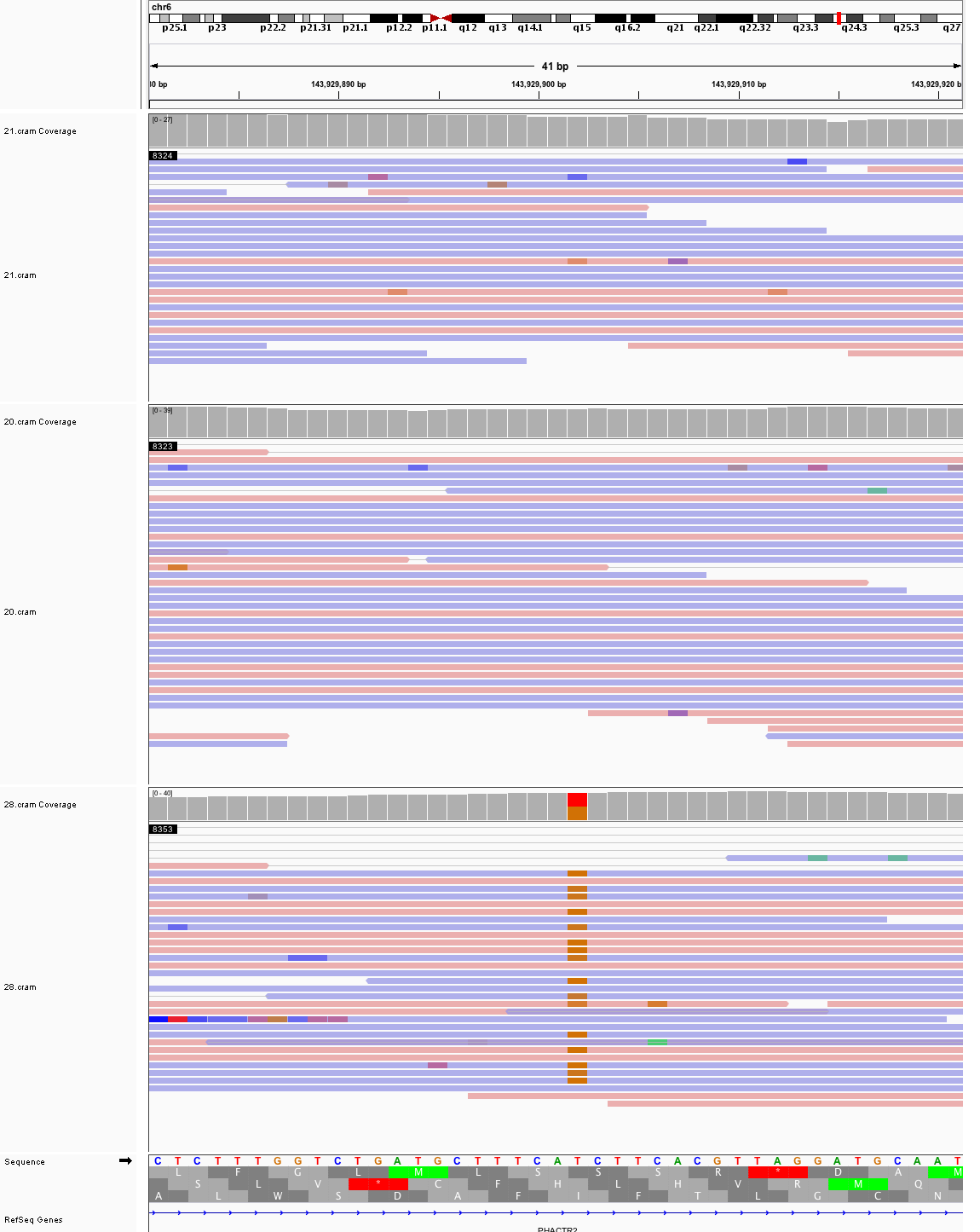

Supplement: Supplementary file 2. — In each image, the first two tracks contain alignments from the first-generation parents, and the third track contains the alignments for the second-generation child. Reads with mapping quality <20 are not included, as they were not considered by our variant calling pipeline, and mismatched bases are shaded by quality score (more transparent = lower base quality). [file elife-46922-supp2.zip › supp_file_2/chr6_143,929,881_143,929,921.png]

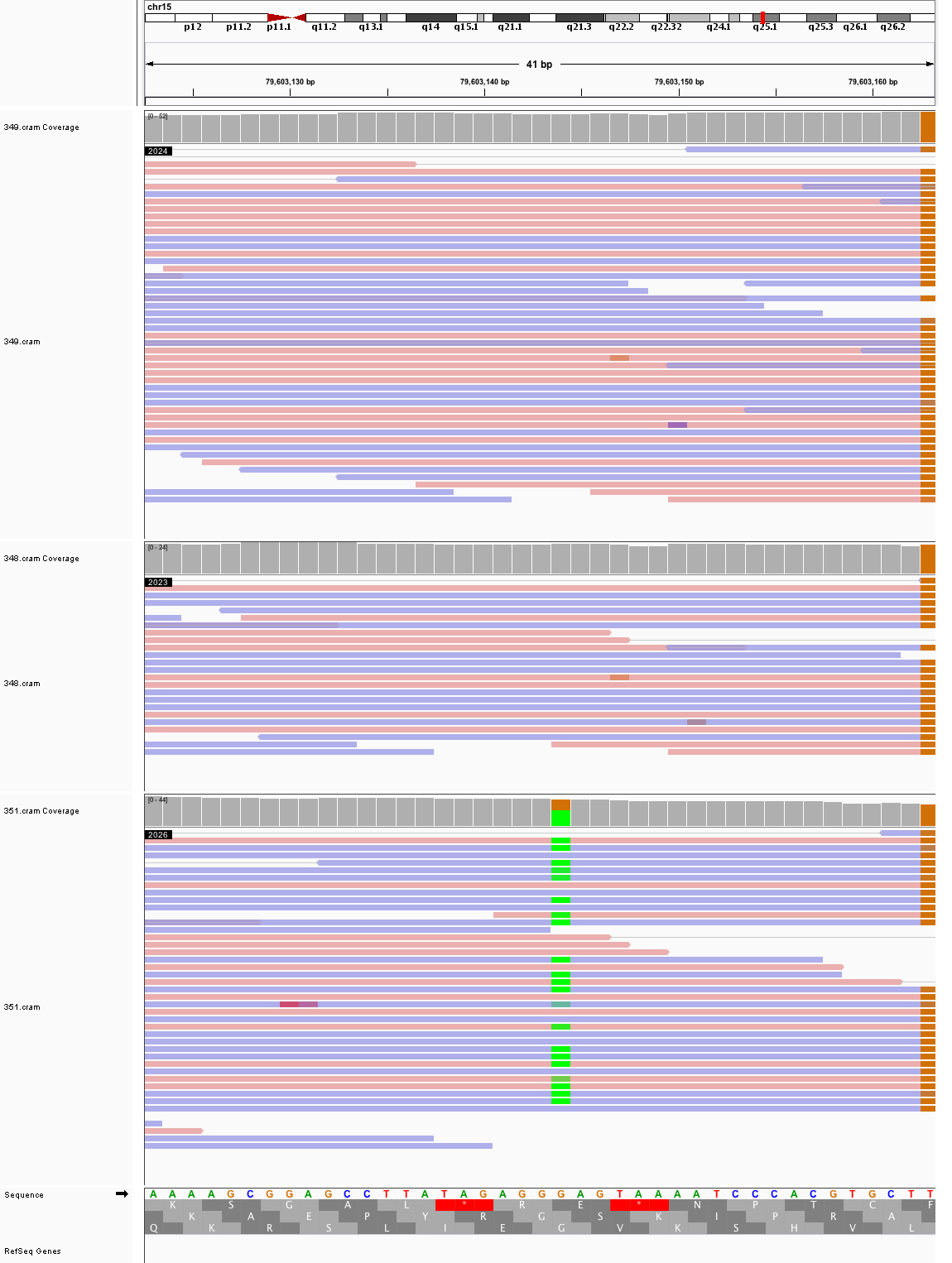

Supplement: Supplementary file 3. — DNMs identified in the third generation In each image, the first two tracks contain alignments from the second-generation parents, and the third track contains the alignments for the third-generation child. Reads with mapping quality <20 are filtered out, as they were not considered by our variant calling pipeline, and mismatched bases are shaded by quality score (more transparent = lower base quality). [file elife-46922-supp3.zip › supp_file_3/chr15_79,603,123_79,603,163.png]

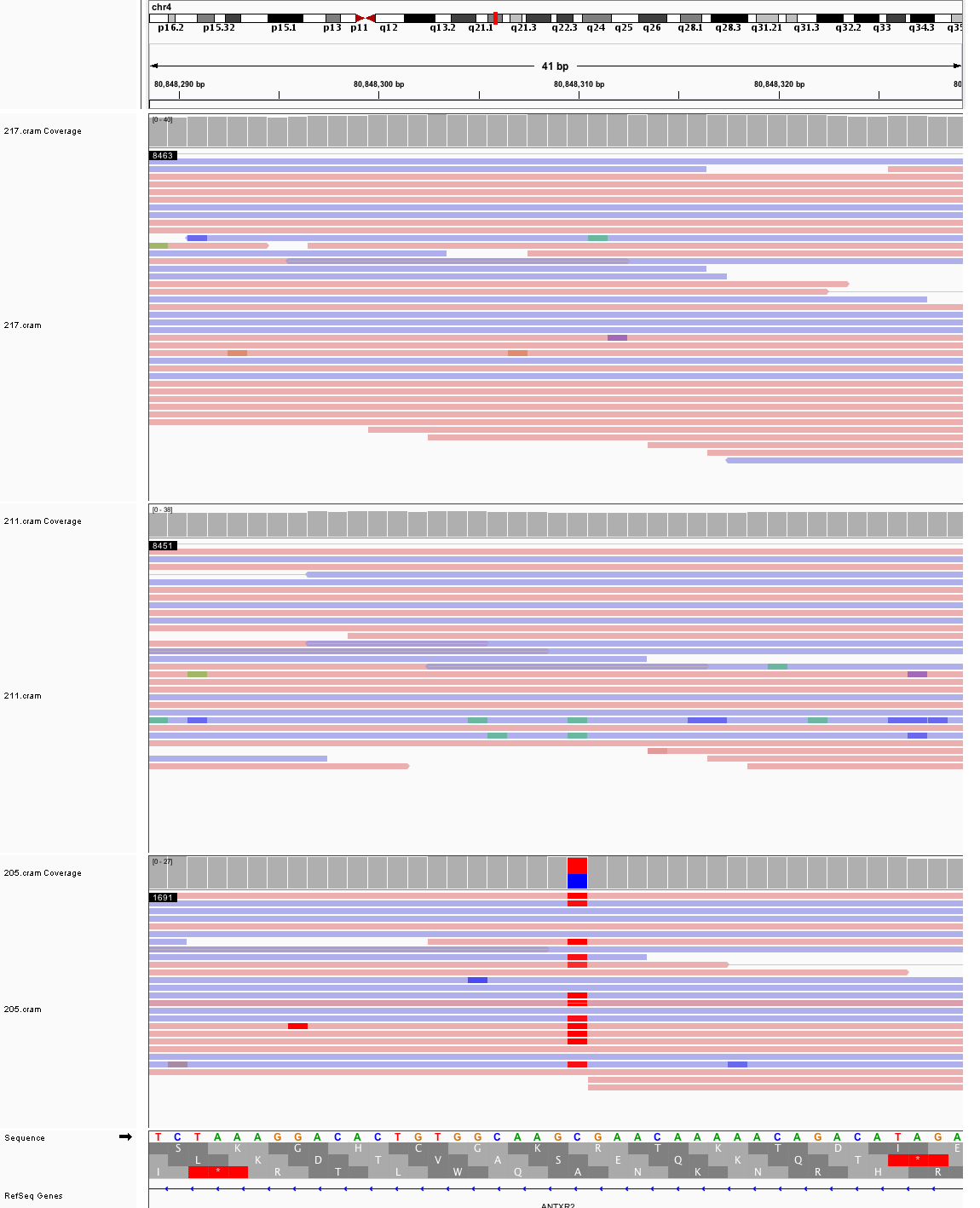

Supplement: Supplementary file 3. — DNMs identified in the third generation In each image, the first two tracks contain alignments from the second-generation parents, and the third track contains the alignments for the third-generation child. Reads with mapping quality <20 are filtered out, as they were not considered by our variant calling pipeline, and mismatched bases are shaded by quality score (more transparent = lower base quality). [file elife-46922-supp3.zip › supp_file_3/chr4_80,848,289_80,848,329.png]

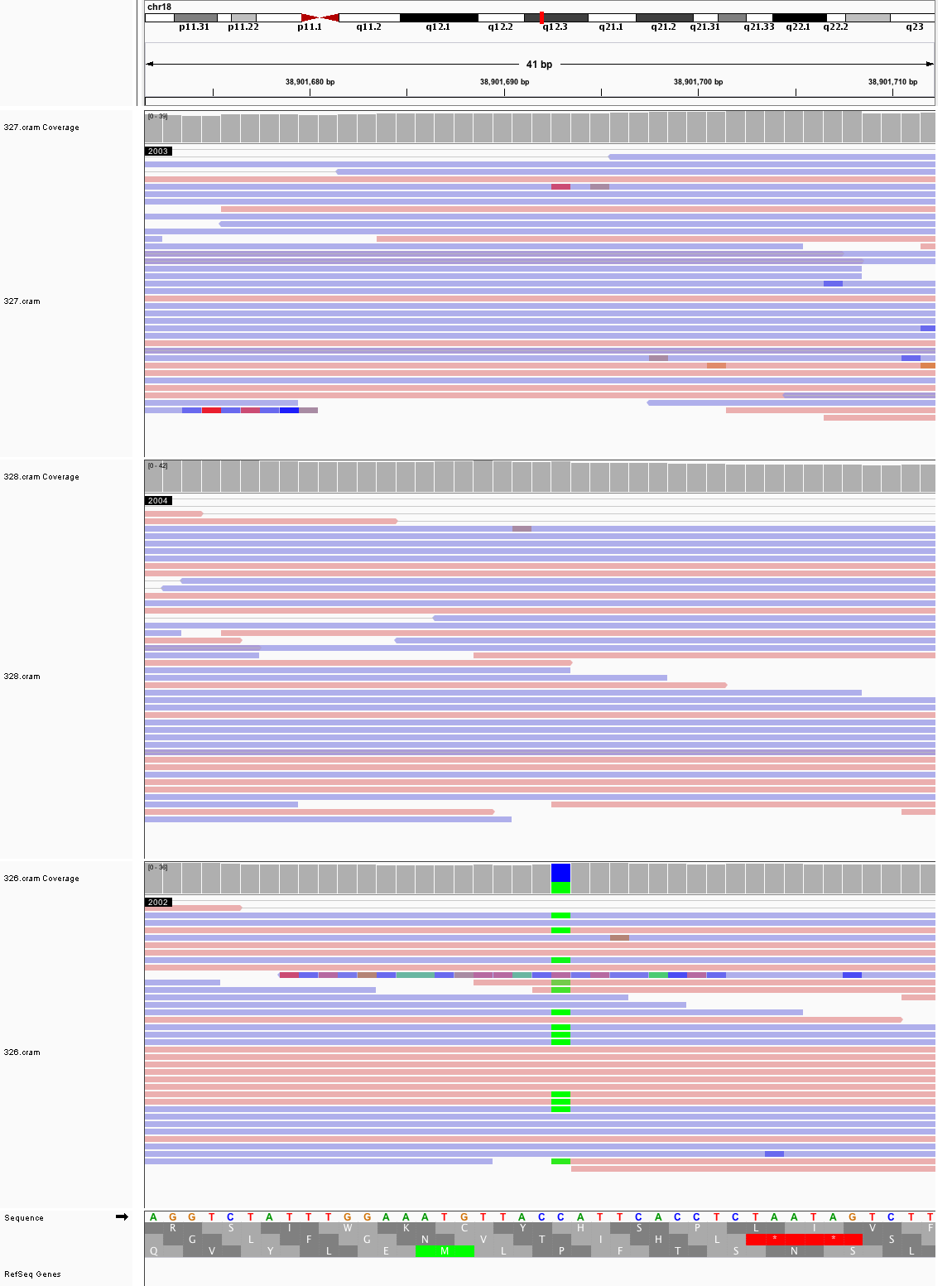

Supplement: Supplementary file 3. — DNMs identified in the third generation In each image, the first two tracks contain alignments from the second-generation parents, and the third track contains the alignments for the third-generation child. Reads with mapping quality <20 are filtered out, as they were not considered by our variant calling pipeline, and mismatched bases are shaded by quality score (more transparent = lower base quality). [file elife-46922-supp3.zip › supp_file_3/chr18_38,901,672_38,901,712.png]

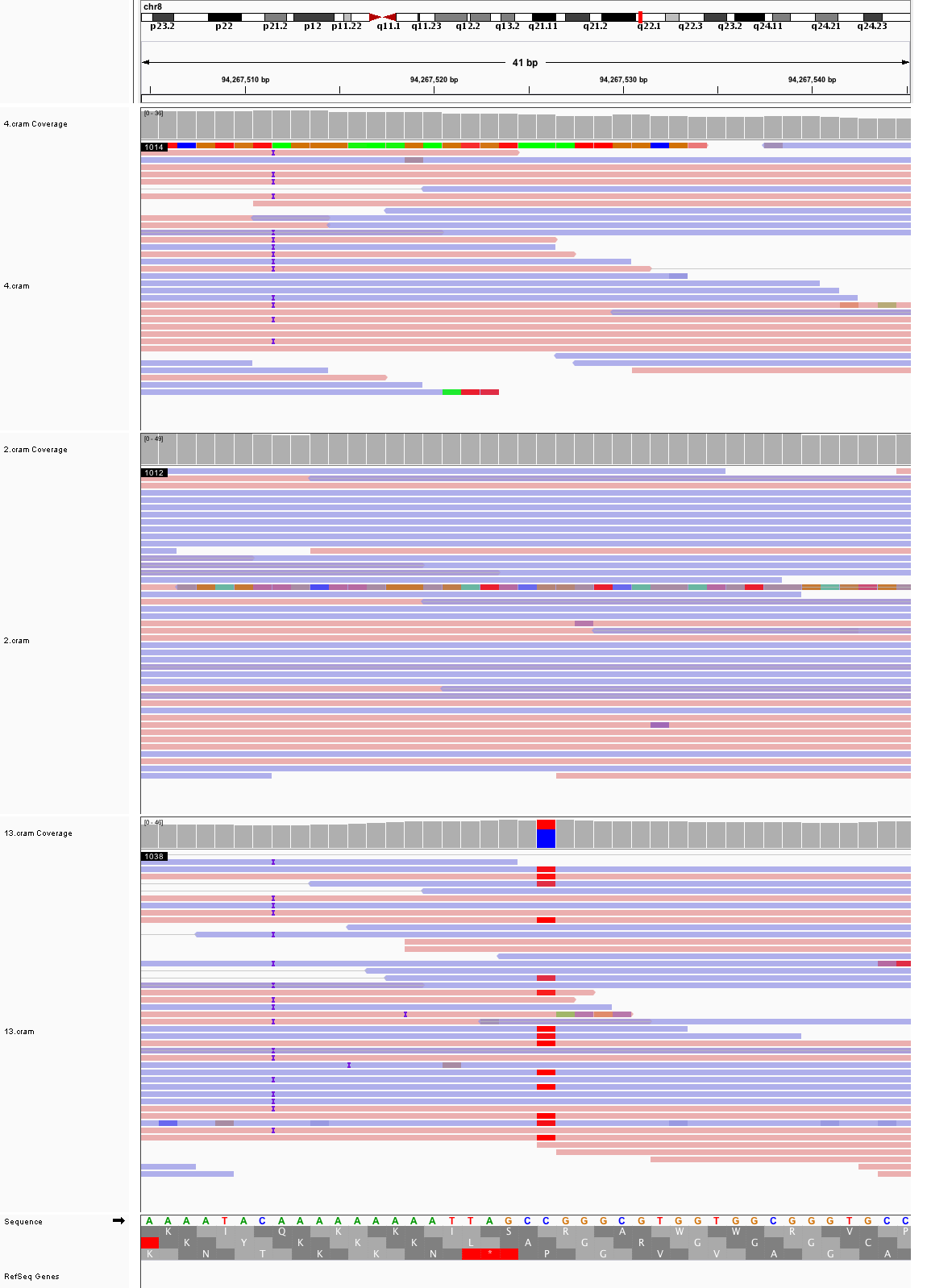

Supplement: Supplementary file 3. — DNMs identified in the third generation In each image, the first two tracks contain alignments from the second-generation parents, and the third track contains the alignments for the third-generation child. Reads with mapping quality <20 are filtered out, as they were not considered by our variant calling pipeline, and mismatched bases are shaded by quality score (more transparent = lower base quality). [file elife-46922-supp3.zip › supp_file_3/chr8_94,267,505_94,267,545.png]

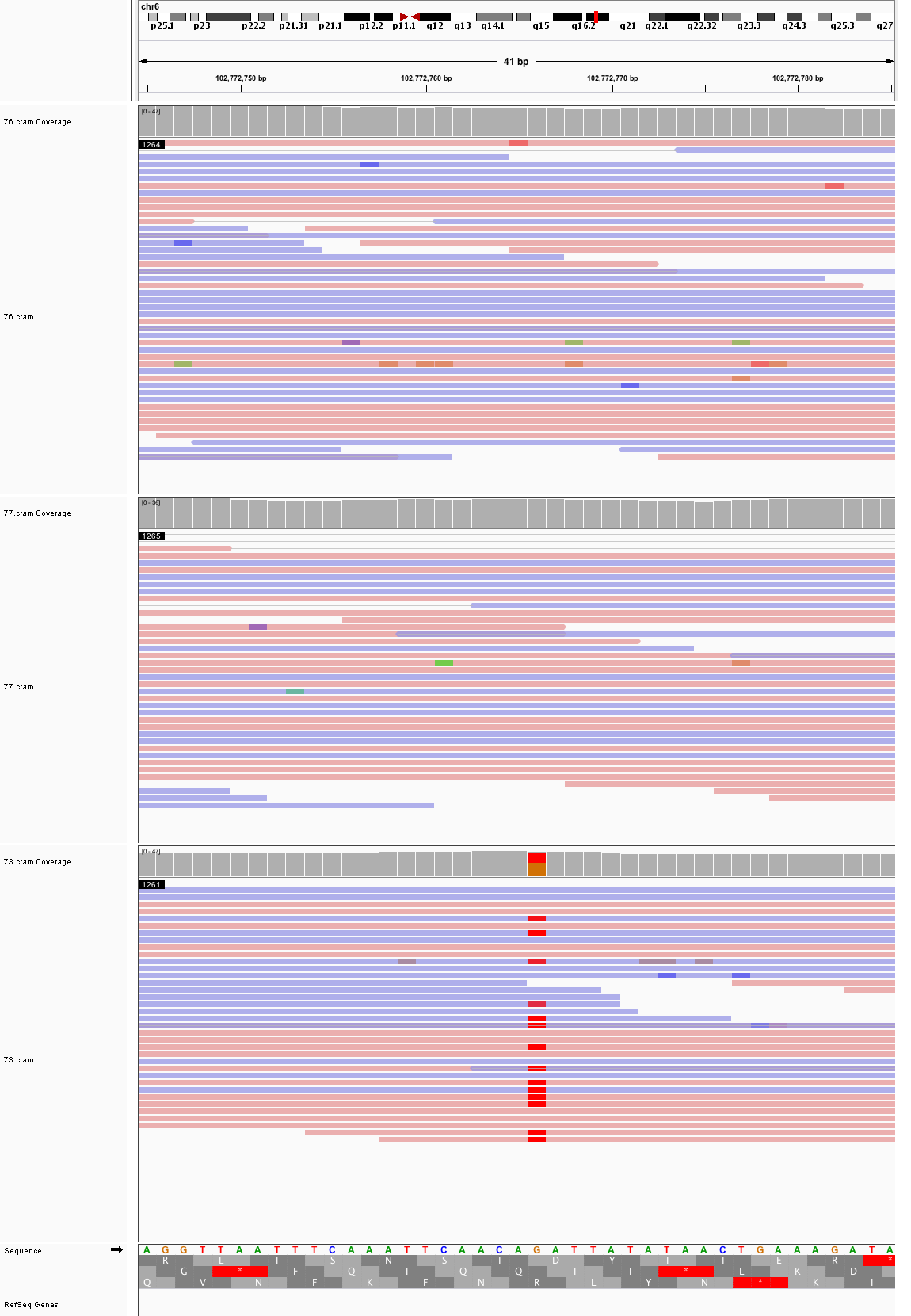

Supplement: Supplementary file 3. — DNMs identified in the third generation In each image, the first two tracks contain alignments from the second-generation parents, and the third track contains the alignments for the third-generation child. Reads with mapping quality <20 are filtered out, as they were not considered by our variant calling pipeline, and mismatched bases are shaded by quality score (more transparent = lower base quality). [file elife-46922-supp3.zip › supp_file_3/chr6_102,772,745_102,772,785.png]

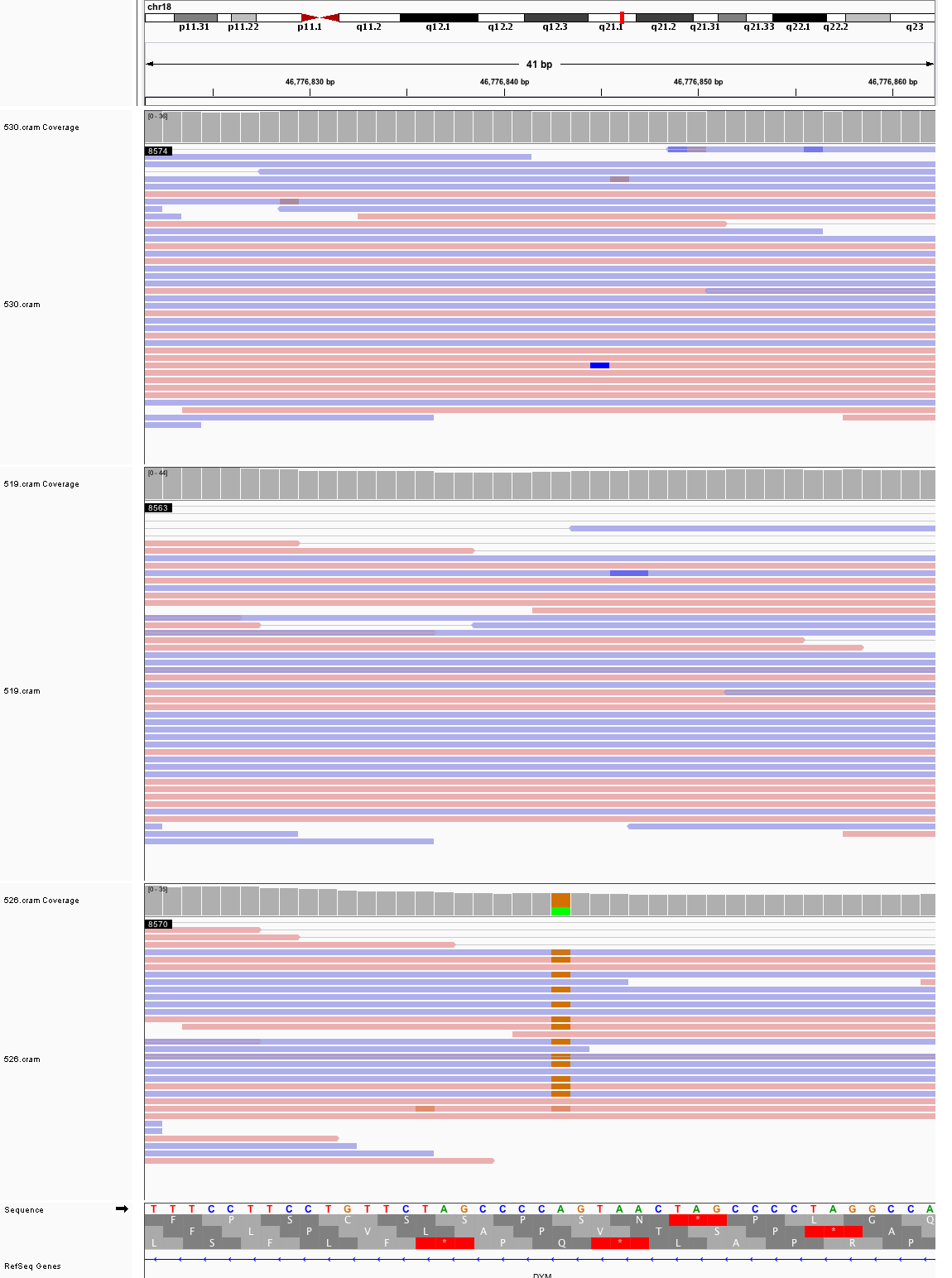

Supplement: Supplementary file 3. — DNMs identified in the third generation In each image, the first two tracks contain alignments from the second-generation parents, and the third track contains the alignments for the third-generation child. Reads with mapping quality <20 are filtered out, as they were not considered by our variant calling pipeline, and mismatched bases are shaded by quality score (more transparent = lower base quality). [file elife-46922-supp3.zip › supp_file_3/chr18_46,776,822_46,776,862.png]

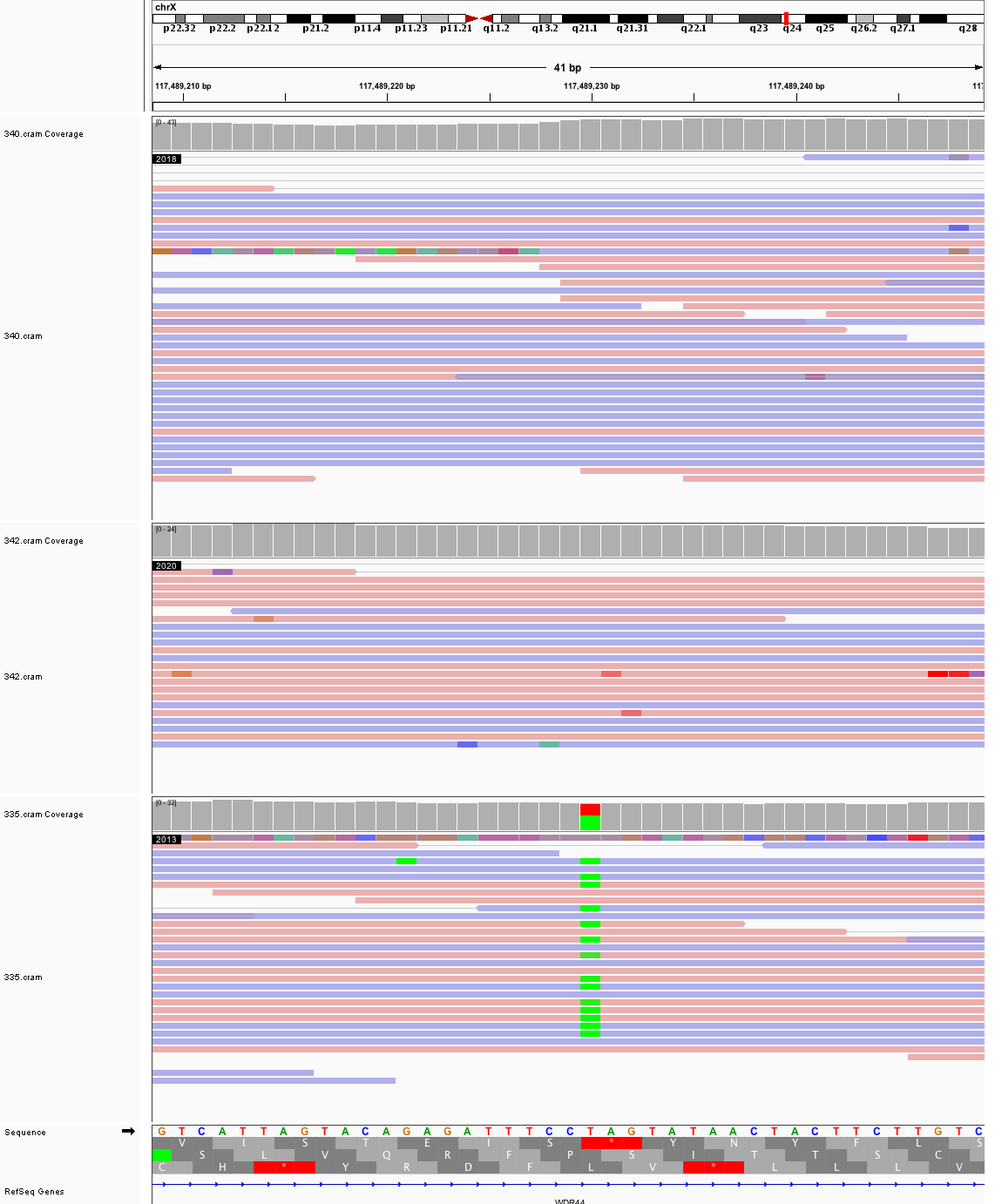

Supplement: Supplementary file 3. — DNMs identified in the third generation In each image, the first two tracks contain alignments from the second-generation parents, and the third track contains the alignments for the third-generation child. Reads with mapping quality <20 are filtered out, as they were not considered by our variant calling pipeline, and mismatched bases are shaded by quality score (more transparent = lower base quality). [file elife-46922-supp3.zip › supp_file_3/chrX_117,489,209_117,489,249.png]

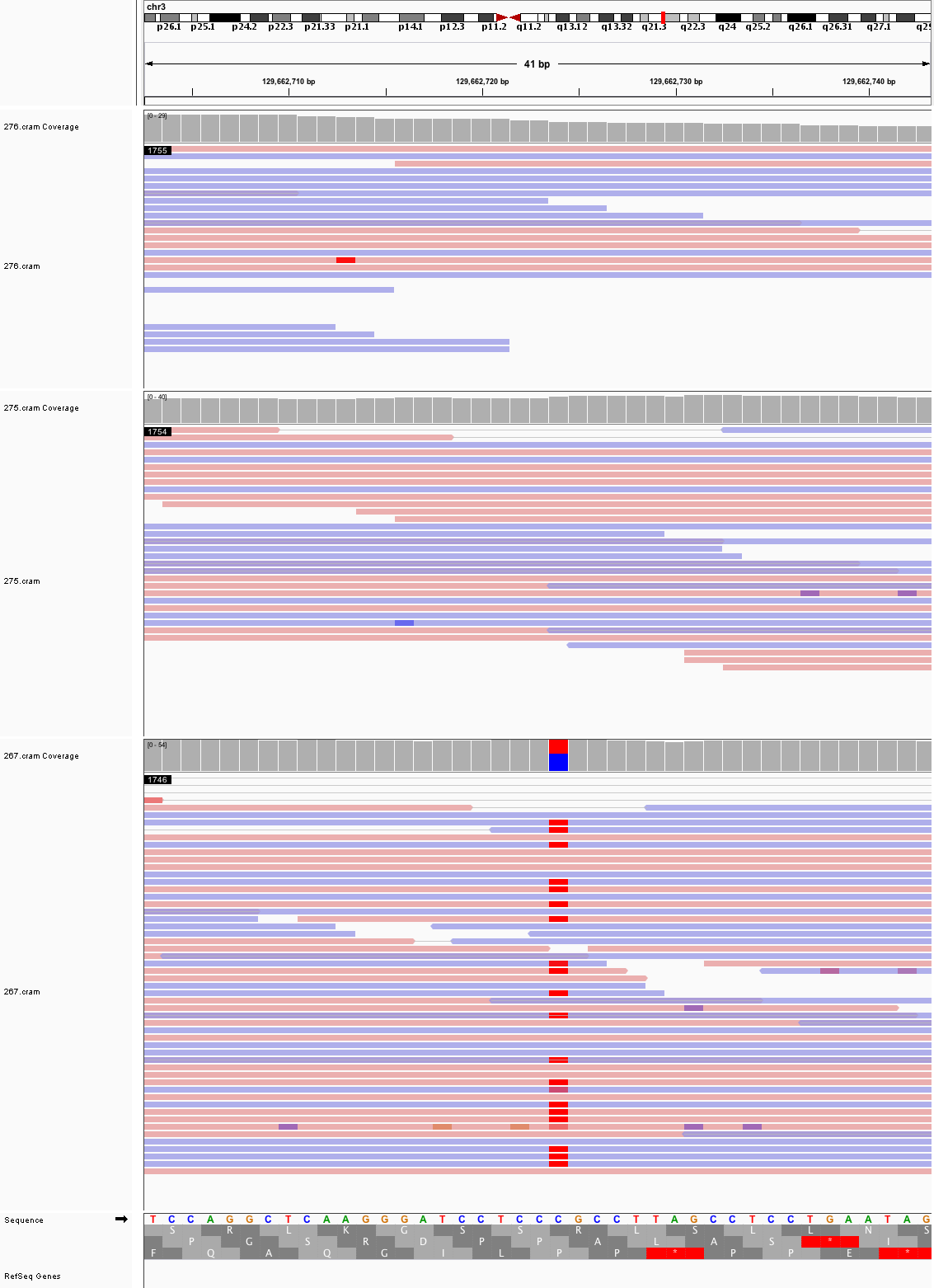

Supplement: Supplementary file 3. — DNMs identified in the third generation In each image, the first two tracks contain alignments from the second-generation parents, and the third track contains the alignments for the third-generation child. Reads with mapping quality <20 are filtered out, as they were not considered by our variant calling pipeline, and mismatched bases are shaded by quality score (more transparent = lower base quality). [file elife-46922-supp3.zip › supp_file_3/chr3_129,662,703_129,662,743.png]

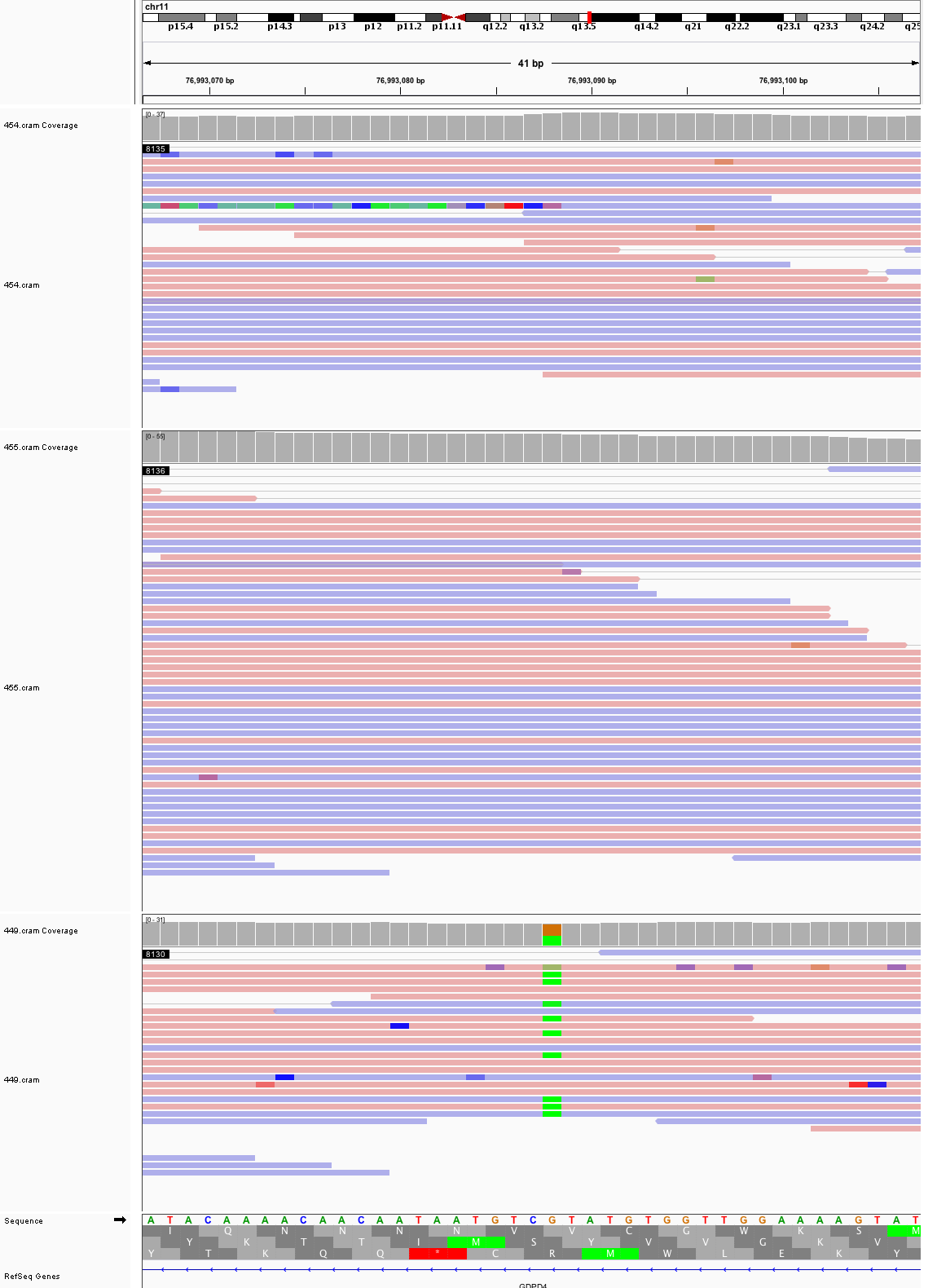

Supplement: Supplementary file 3. — DNMs identified in the third generation In each image, the first two tracks contain alignments from the second-generation parents, and the third track contains the alignments for the third-generation child. Reads with mapping quality <20 are filtered out, as they were not considered by our variant calling pipeline, and mismatched bases are shaded by quality score (more transparent = lower base quality). [file elife-46922-supp3.zip › supp_file_3/chr11_76,993,067_76,993,107.png]

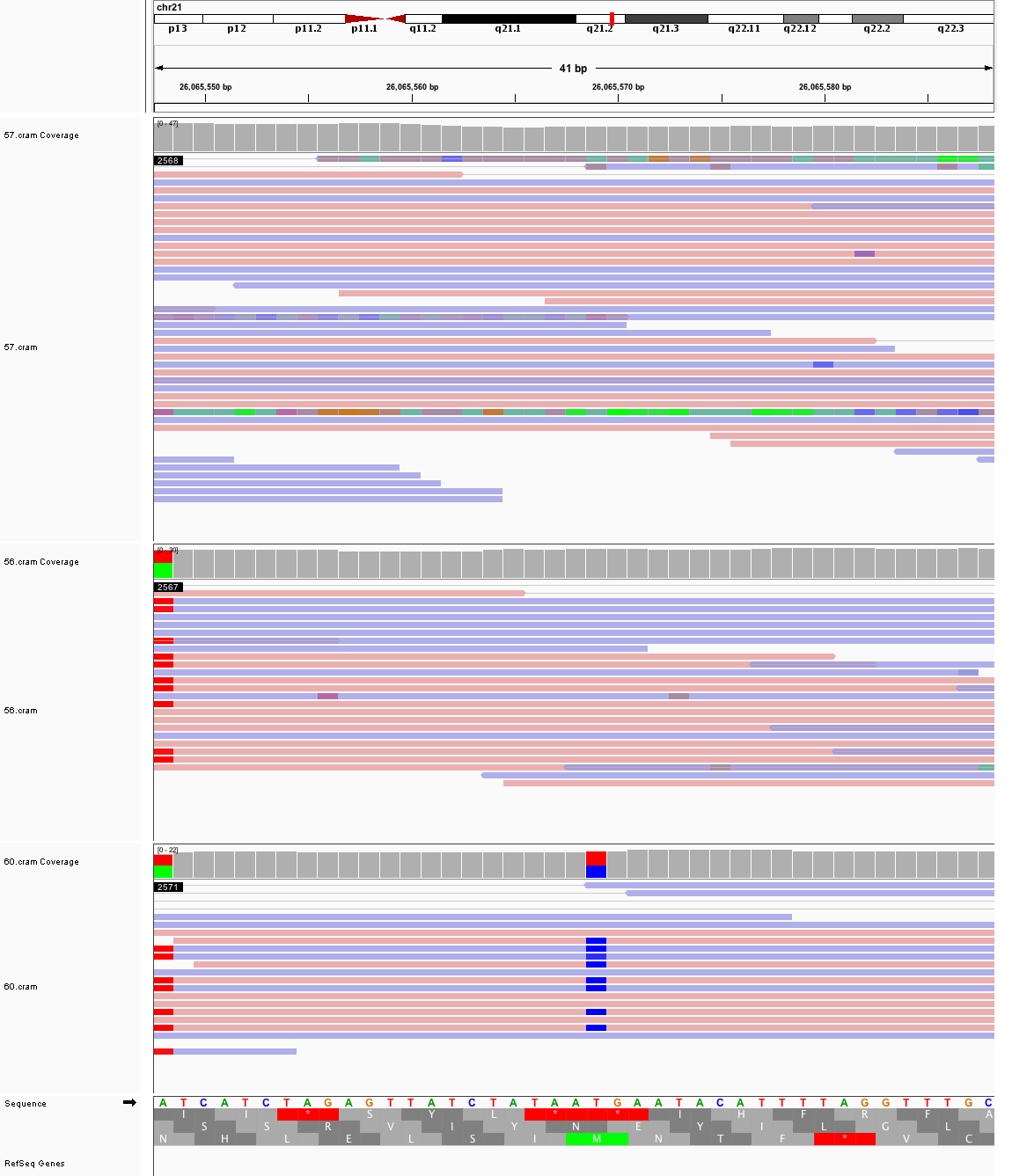

Supplement: Supplementary file 3. — DNMs identified in the third generation In each image, the first two tracks contain alignments from the second-generation parents, and the third track contains the alignments for the third-generation child. Reads with mapping quality <20 are filtered out, as they were not considered by our variant calling pipeline, and mismatched bases are shaded by quality score (more transparent = lower base quality). [file elife-46922-supp3.zip › supp_file_3/chr21_26,065,548_26,065,588.png]

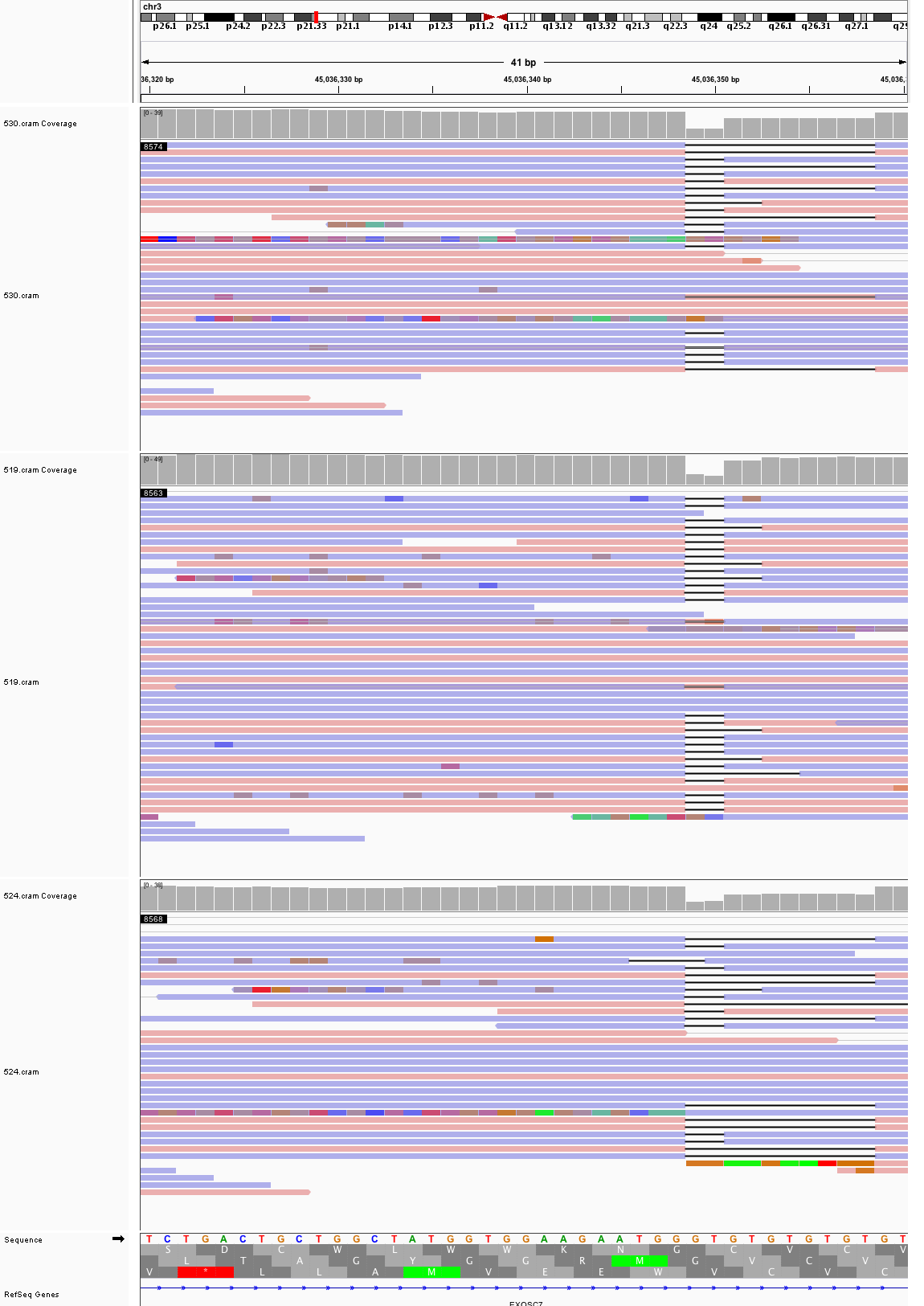

Supplement: Supplementary file 3. — DNMs identified in the third generation In each image, the first two tracks contain alignments from the second-generation parents, and the third track contains the alignments for the third-generation child. Reads with mapping quality <20 are filtered out, as they were not considered by our variant calling pipeline, and mismatched bases are shaded by quality score (more transparent = lower base quality). [file elife-46922-supp3.zip › supp_file_3/chr3_45,036,320_45,036,360.png]

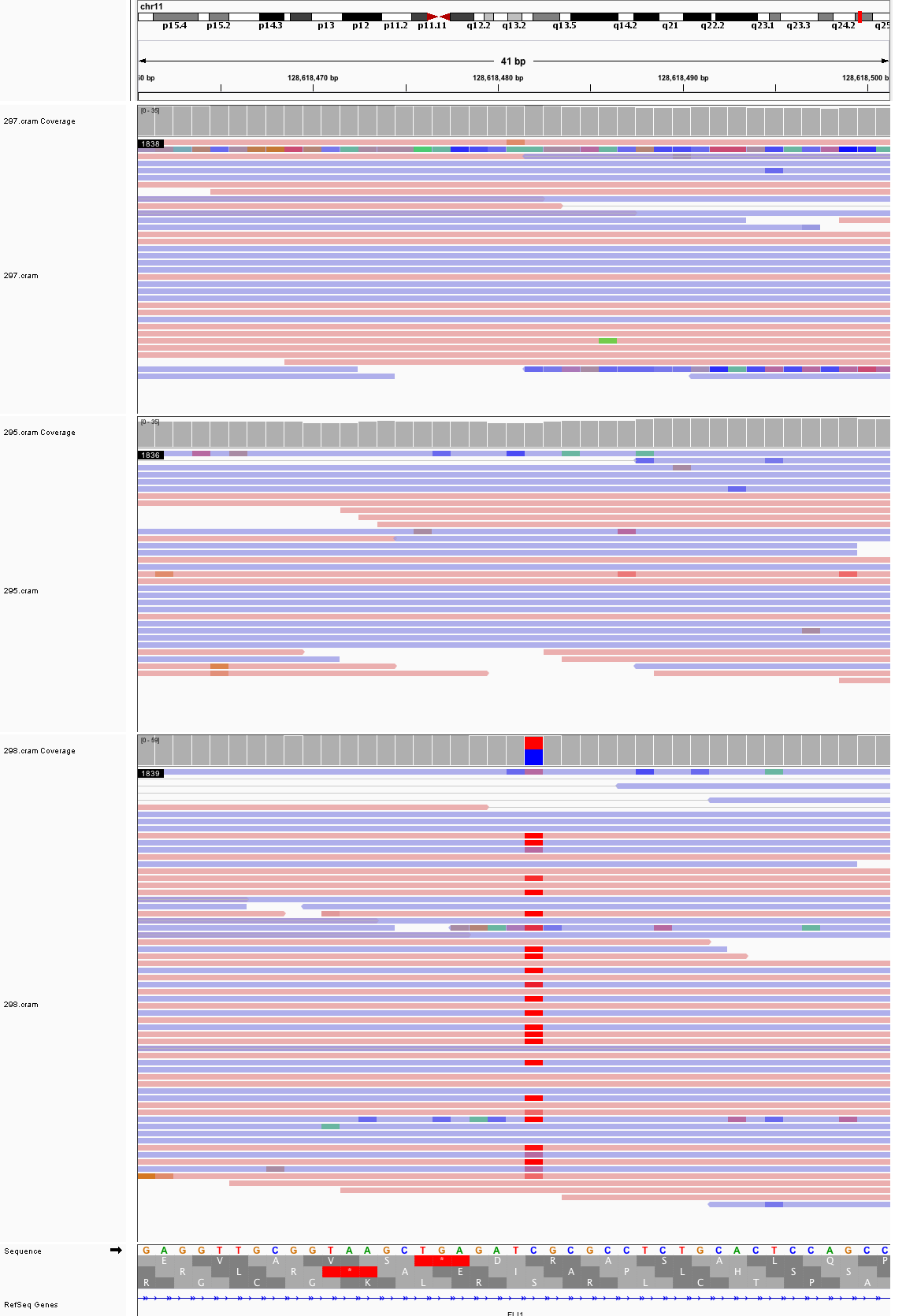

Supplement: Supplementary file 3. — DNMs identified in the third generation In each image, the first two tracks contain alignments from the second-generation parents, and the third track contains the alignments for the third-generation child. Reads with mapping quality <20 are filtered out, as they were not considered by our variant calling pipeline, and mismatched bases are shaded by quality score (more transparent = lower base quality). [file elife-46922-supp3.zip › supp_file_3/chr11_128,618,461_128,618,501.png]

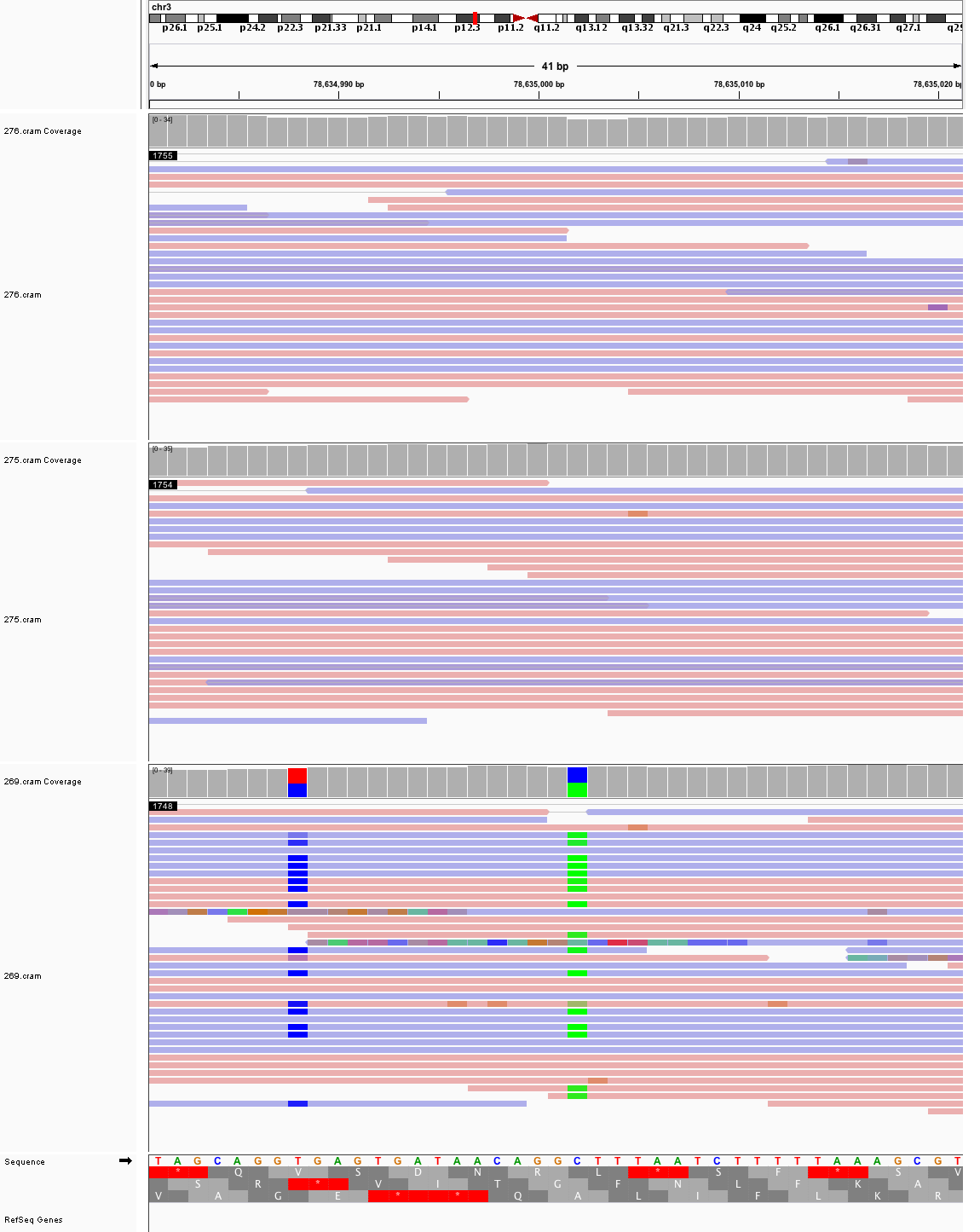

Supplement: Supplementary file 3. — DNMs identified in the third generation In each image, the first two tracks contain alignments from the second-generation parents, and the third track contains the alignments for the third-generation child. Reads with mapping quality <20 are filtered out, as they were not considered by our variant calling pipeline, and mismatched bases are shaded by quality score (more transparent = lower base quality). [file elife-46922-supp3.zip › supp_file_3/chr3_78,634,981_78,635,021.png]

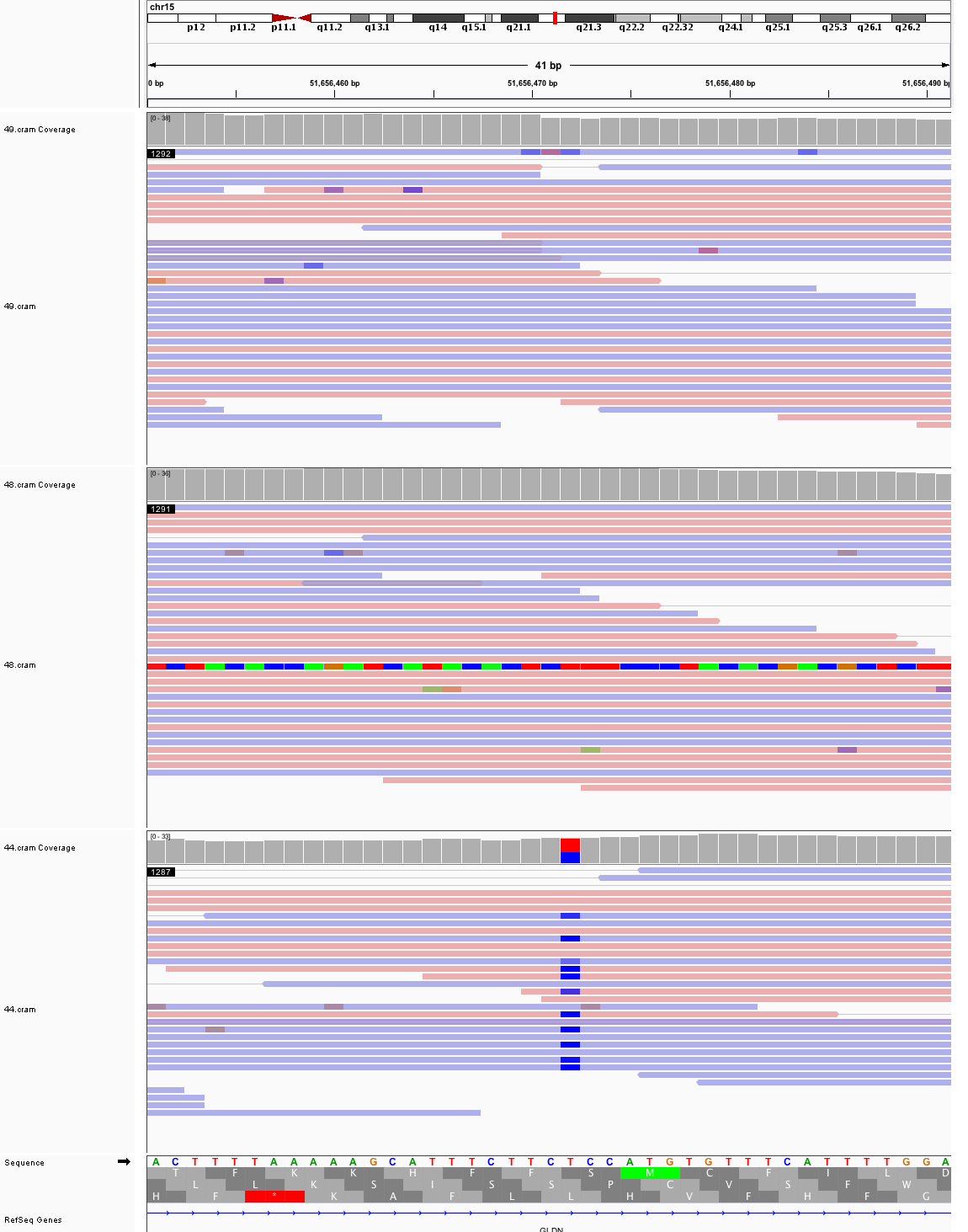

Supplement: Supplementary file 3. — DNMs identified in the third generation In each image, the first two tracks contain alignments from the second-generation parents, and the third track contains the alignments for the third-generation child. Reads with mapping quality <20 are filtered out, as they were not considered by our variant calling pipeline, and mismatched bases are shaded by quality score (more transparent = lower base quality). [file elife-46922-supp3.zip › supp_file_3/chr15_51,656,451_51,656,491.png]

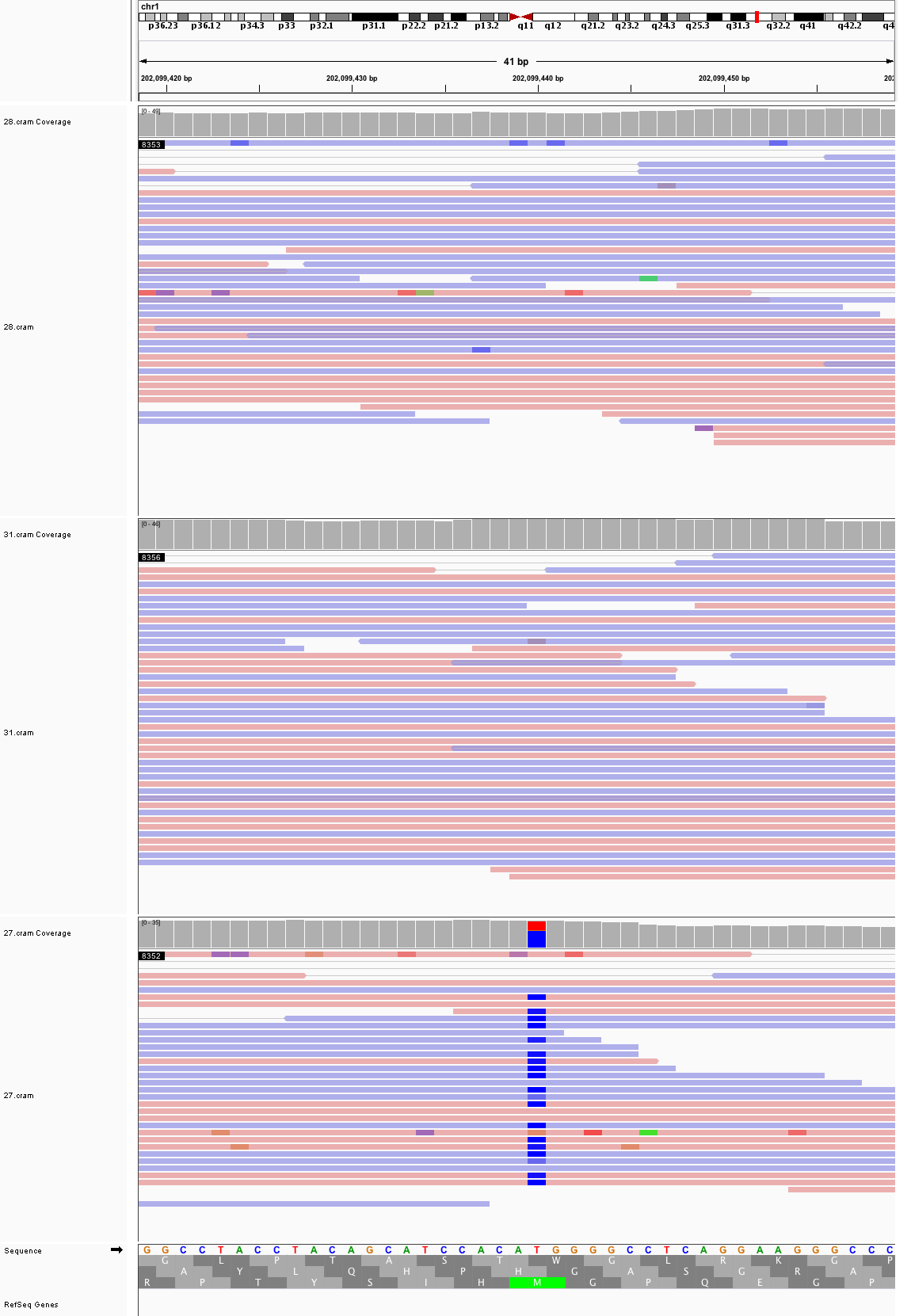

Supplement: Supplementary file 3. — DNMs identified in the third generation In each image, the first two tracks contain alignments from the second-generation parents, and the third track contains the alignments for the third-generation child. Reads with mapping quality <20 are filtered out, as they were not considered by our variant calling pipeline, and mismatched bases are shaded by quality score (more transparent = lower base quality). [file elife-46922-supp3.zip › supp_file_3/chr1_202,099,419_202,099,459.png]

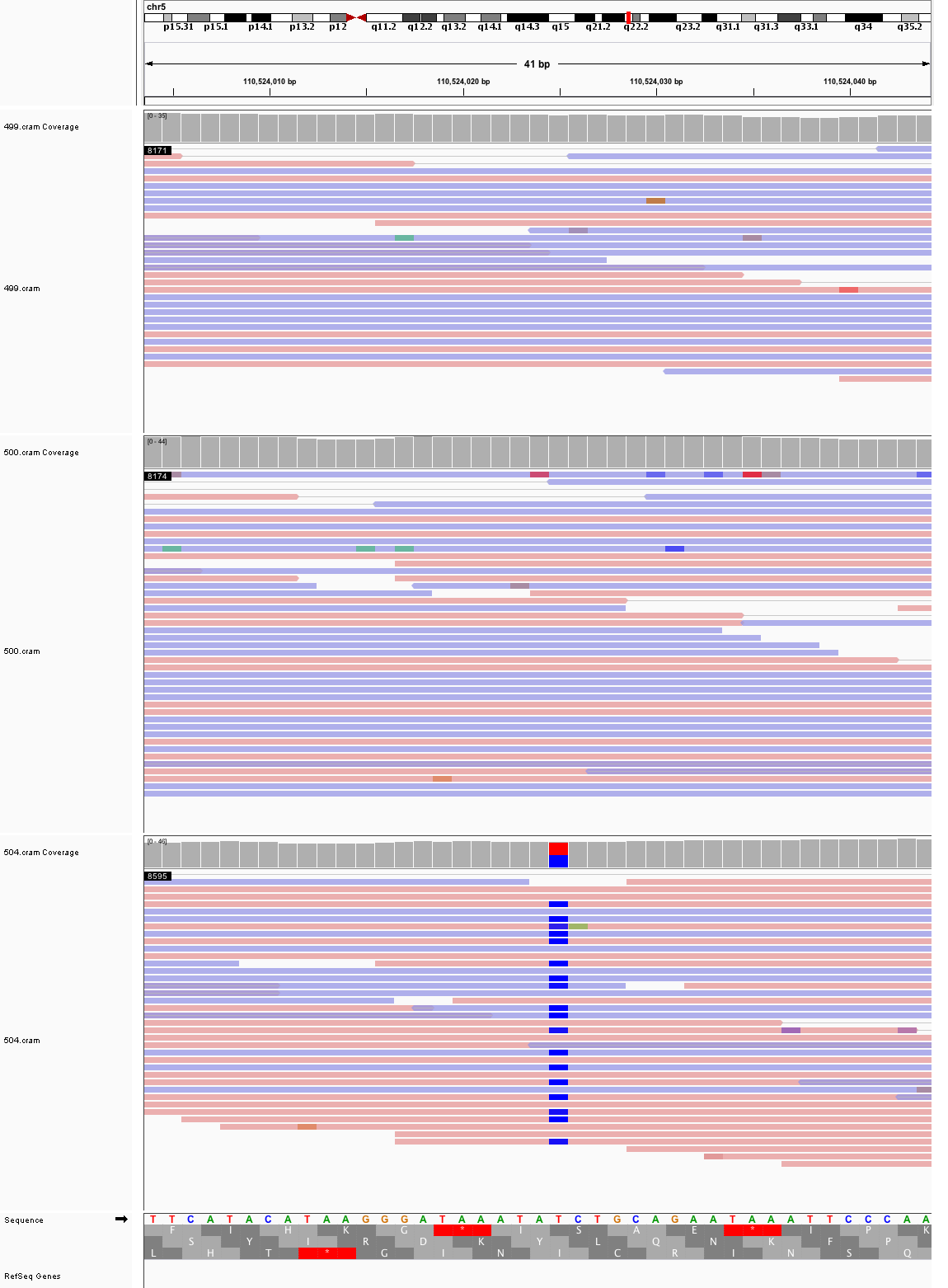

Supplement: Supplementary file 3. — DNMs identified in the third generation In each image, the first two tracks contain alignments from the second-generation parents, and the third track contains the alignments for the third-generation child. Reads with mapping quality <20 are filtered out, as they were not considered by our variant calling pipeline, and mismatched bases are shaded by quality score (more transparent = lower base quality). [file elife-46922-supp3.zip › supp_file_3/chr5_110,524,004_110,524,044.png]

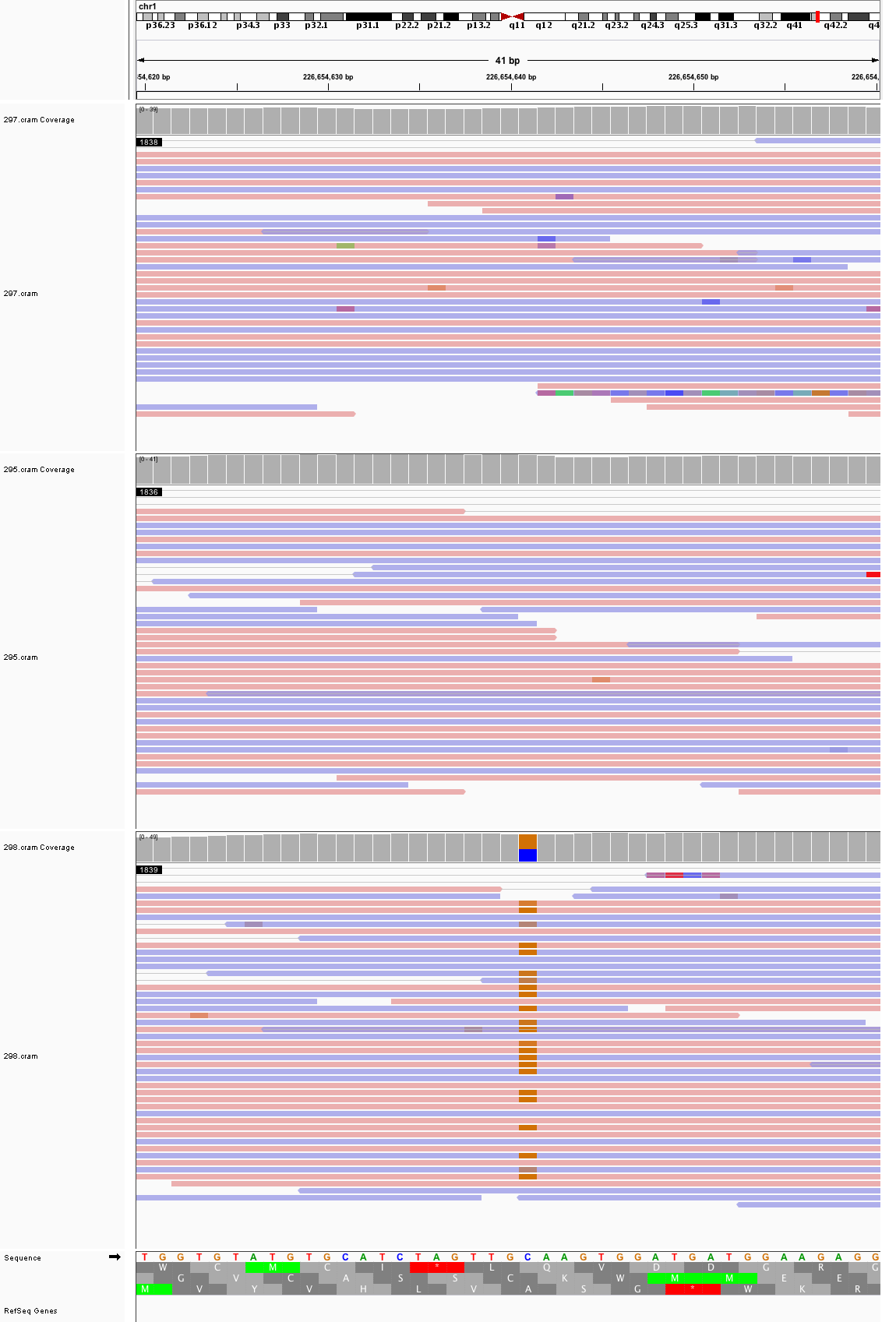

Supplement: Supplementary file 3. — DNMs identified in the third generation In each image, the first two tracks contain alignments from the second-generation parents, and the third track contains the alignments for the third-generation child. Reads with mapping quality <20 are filtered out, as they were not considered by our variant calling pipeline, and mismatched bases are shaded by quality score (more transparent = lower base quality). [file elife-46922-supp3.zip › supp_file_3/chr1_226,654,620_226,654,660.png]

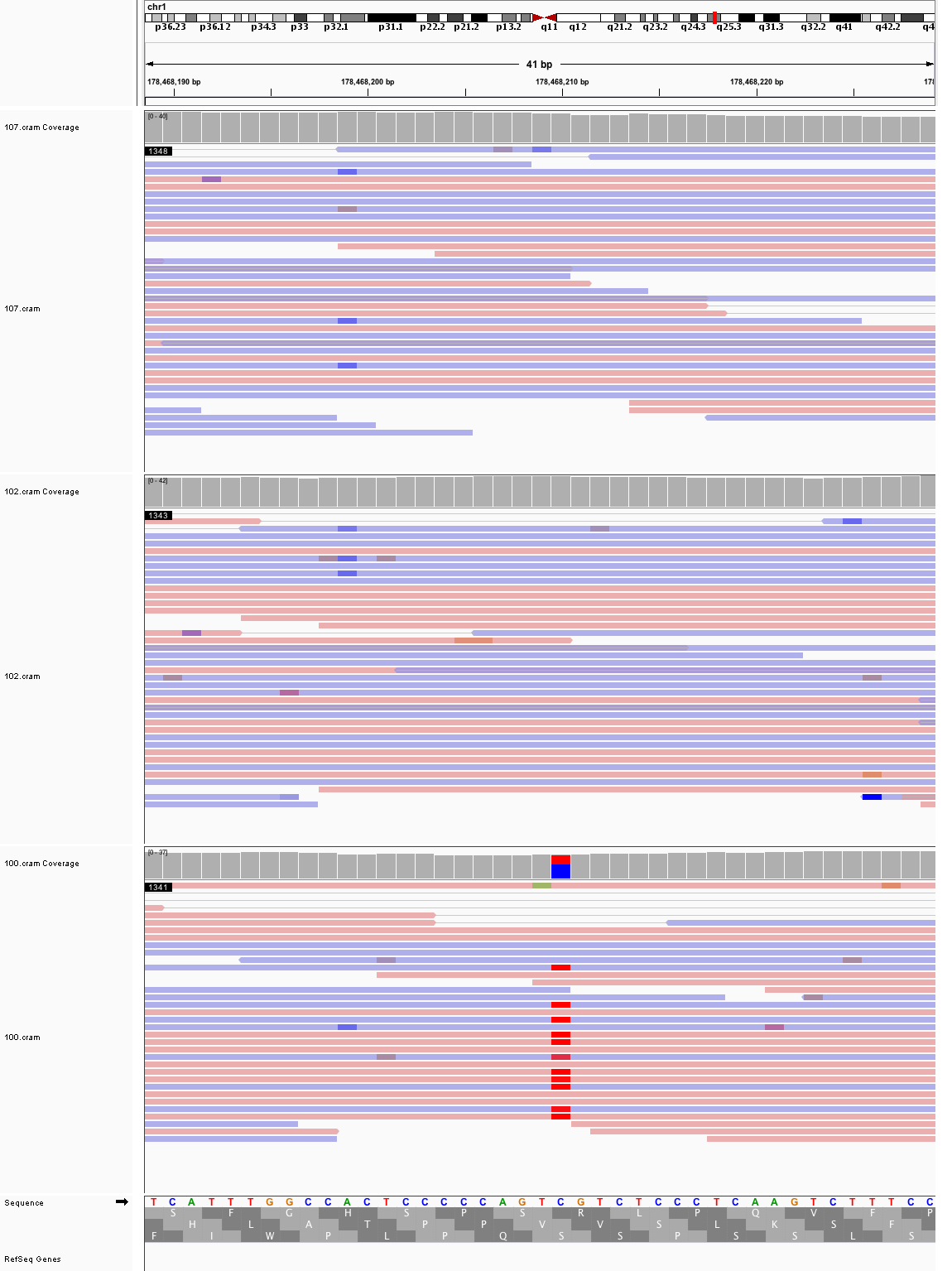

Supplement: Supplementary file 3. — DNMs identified in the third generation In each image, the first two tracks contain alignments from the second-generation parents, and the third track contains the alignments for the third-generation child. Reads with mapping quality <20 are filtered out, as they were not considered by our variant calling pipeline, and mismatched bases are shaded by quality score (more transparent = lower base quality). [file elife-46922-supp3.zip › supp_file_3/chr1_178,468,189_178,468,229.png]

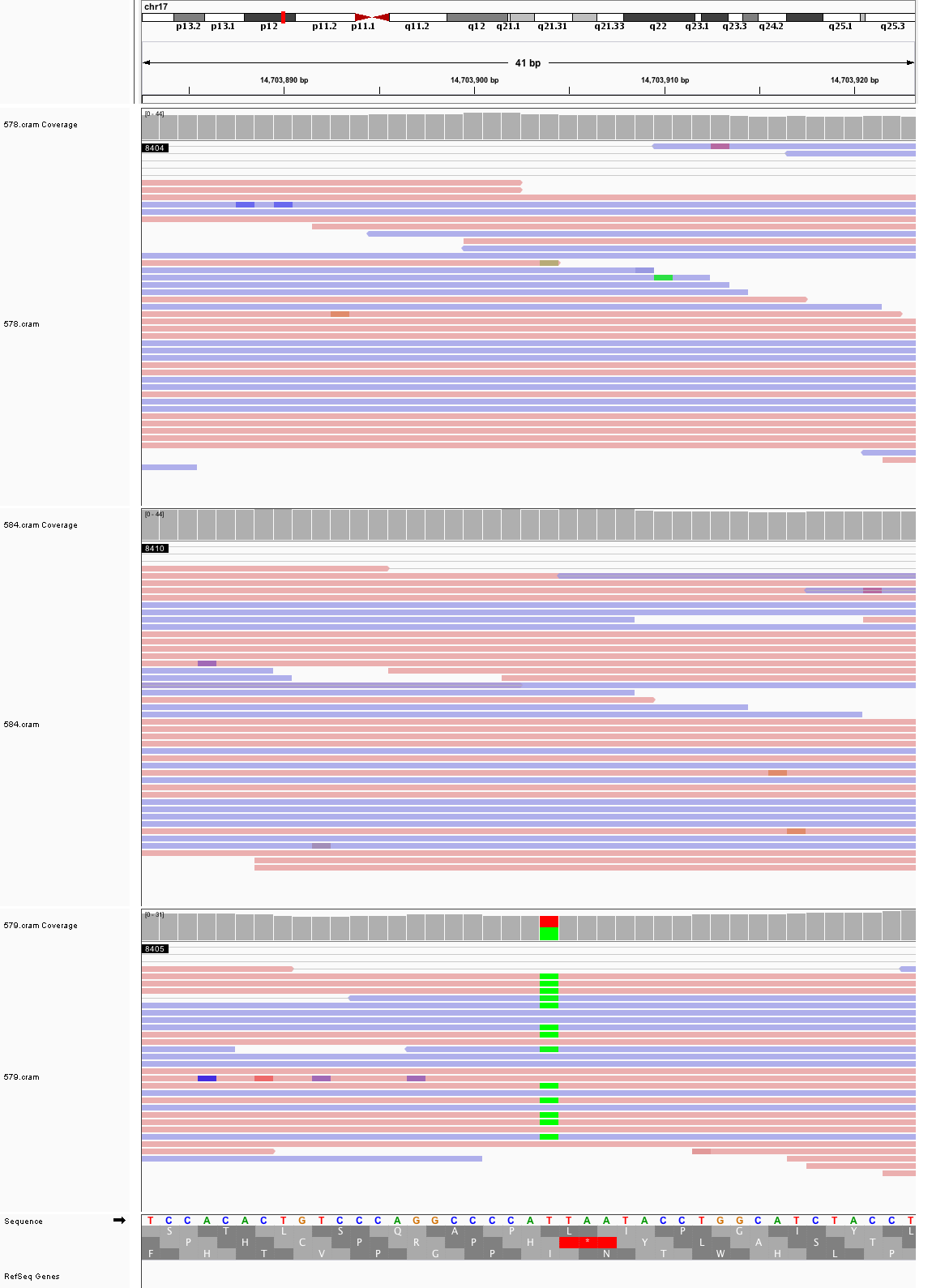

Supplement: Supplementary file 3. — DNMs identified in the third generation In each image, the first two tracks contain alignments from the second-generation parents, and the third track contains the alignments for the third-generation child. Reads with mapping quality <20 are filtered out, as they were not considered by our variant calling pipeline, and mismatched bases are shaded by quality score (more transparent = lower base quality). [file elife-46922-supp3.zip › supp_file_3/chr17_14,703,883_14,703,923.png]

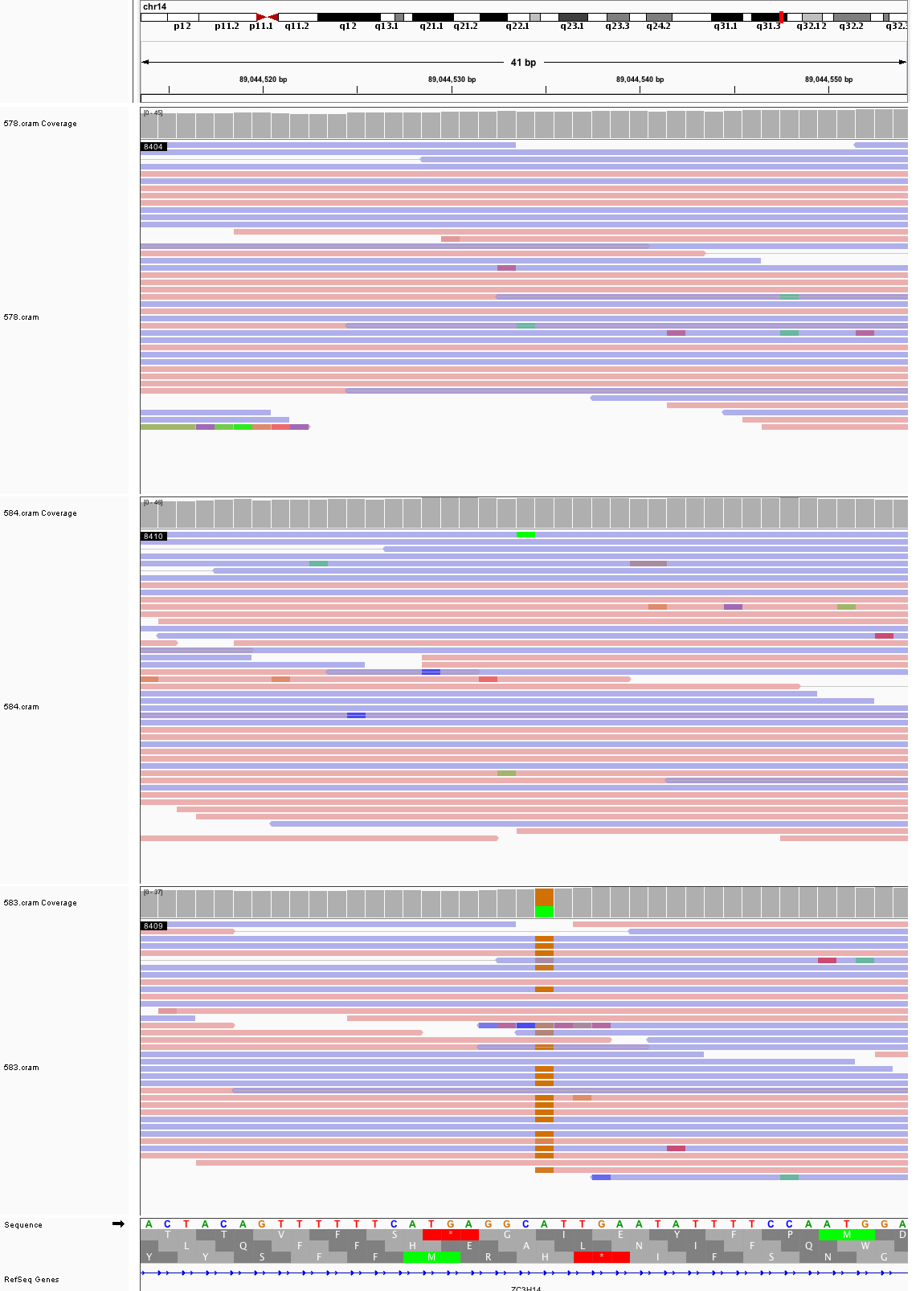

Supplement: Supplementary file 3. — DNMs identified in the third generation In each image, the first two tracks contain alignments from the second-generation parents, and the third track contains the alignments for the third-generation child. Reads with mapping quality <20 are filtered out, as they were not considered by our variant calling pipeline, and mismatched bases are shaded by quality score (more transparent = lower base quality). [file elife-46922-supp3.zip › supp_file_3/chr14_89,044,514_89,044,554.png]

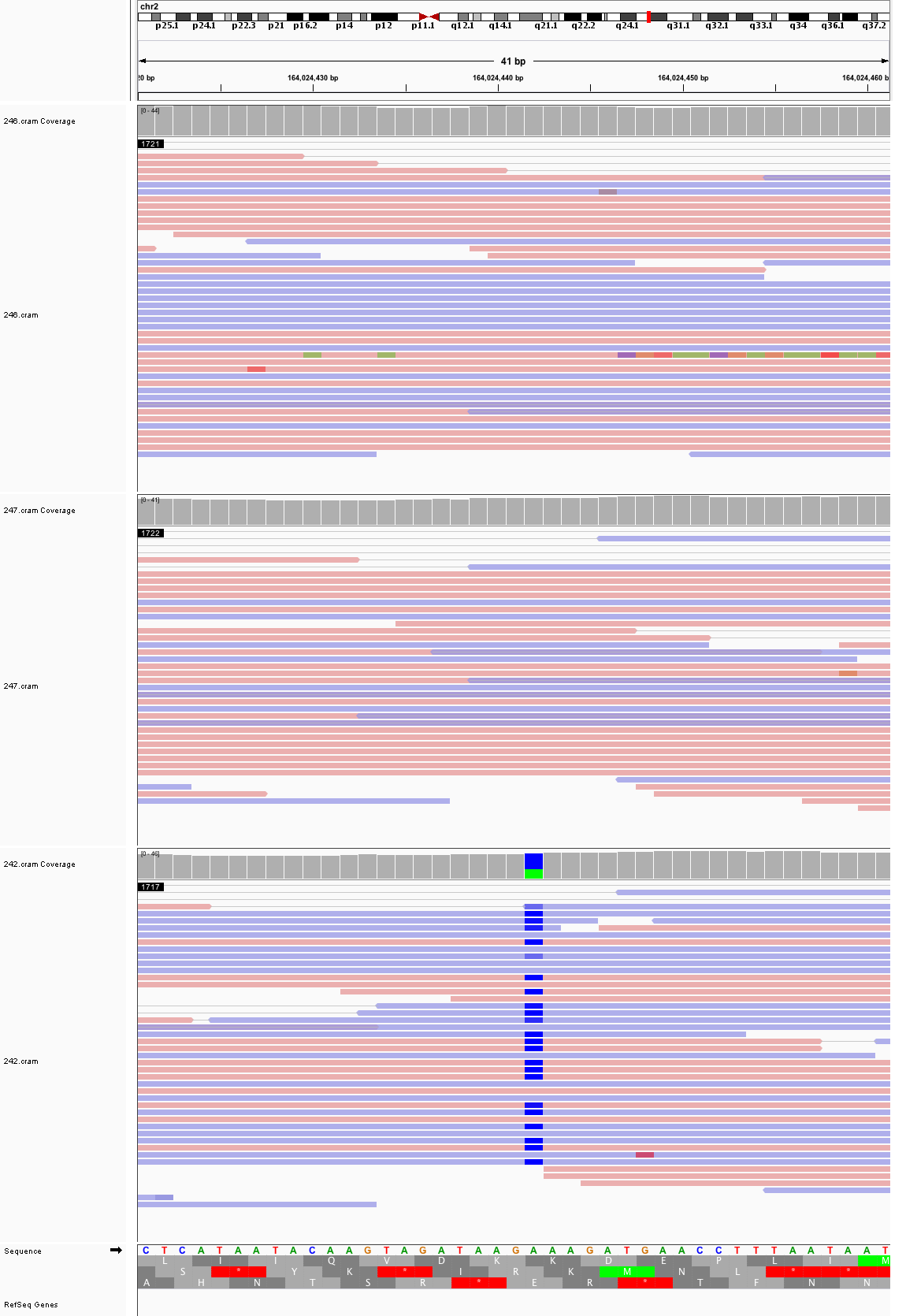

Supplement: Supplementary file 3. — DNMs identified in the third generation In each image, the first two tracks contain alignments from the second-generation parents, and the third track contains the alignments for the third-generation child. Reads with mapping quality <20 are filtered out, as they were not considered by our variant calling pipeline, and mismatched bases are shaded by quality score (more transparent = lower base quality). [file elife-46922-supp3.zip › supp_file_3/chr2_164,024,421_164,024,461.png]

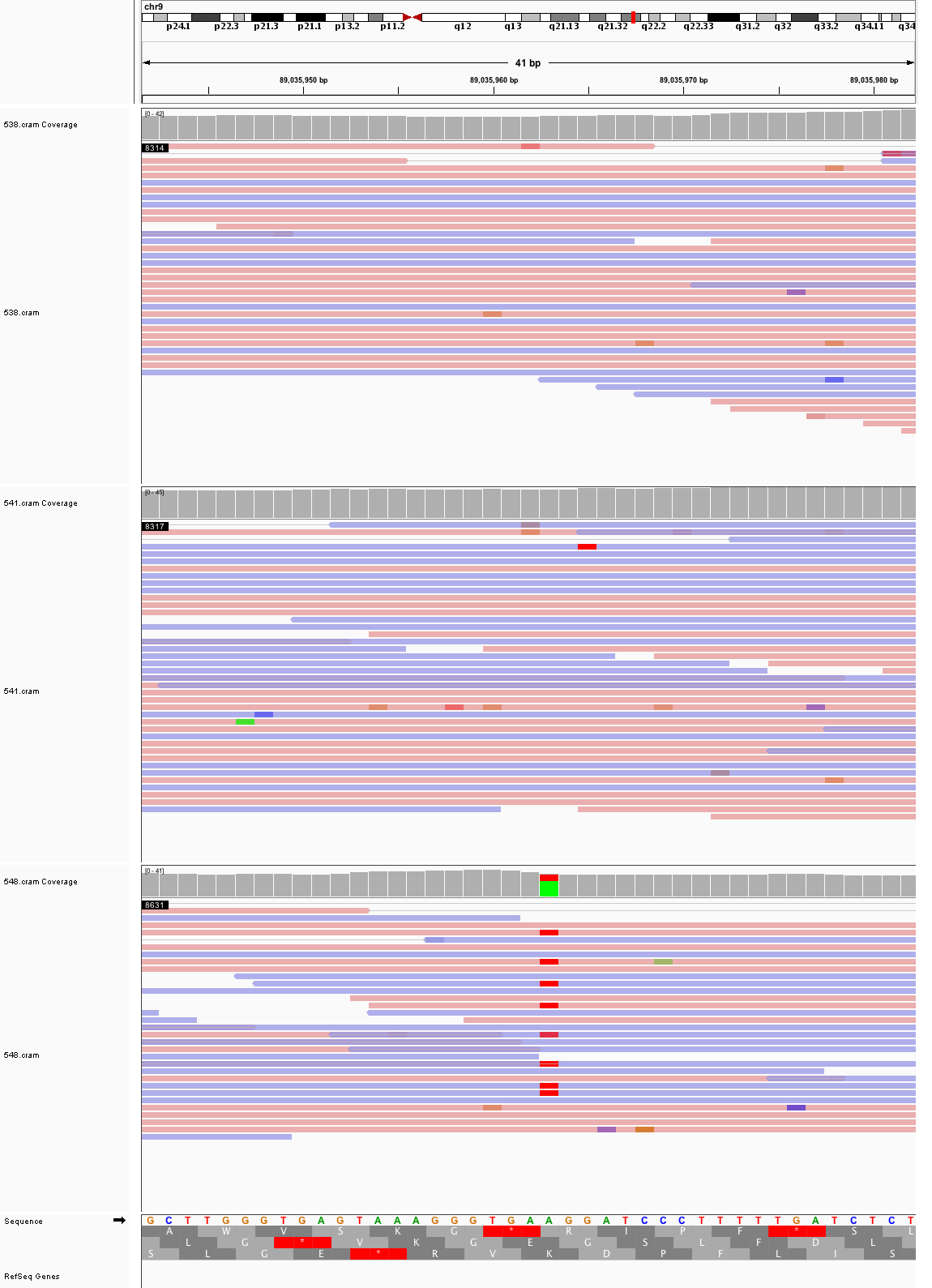

Supplement: Supplementary file 3. — DNMs identified in the third generation In each image, the first two tracks contain alignments from the second-generation parents, and the third track contains the alignments for the third-generation child. Reads with mapping quality <20 are filtered out, as they were not considered by our variant calling pipeline, and mismatched bases are shaded by quality score (more transparent = lower base quality). [file elife-46922-supp3.zip › supp_file_3/chr9_89,035,942_89,035,982.png]

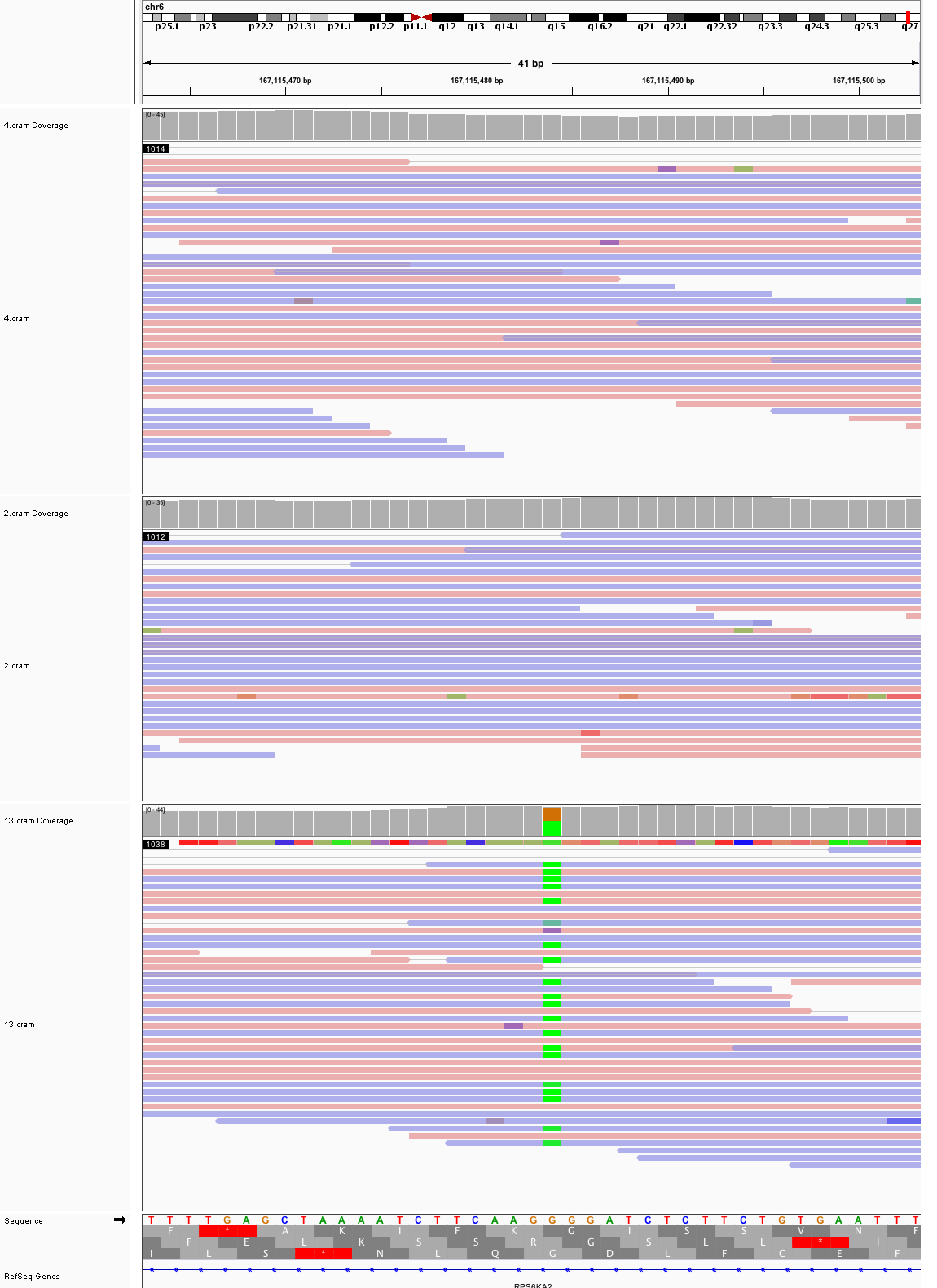

Supplement: Supplementary file 3. — DNMs identified in the third generation In each image, the first two tracks contain alignments from the second-generation parents, and the third track contains the alignments for the third-generation child. Reads with mapping quality <20 are filtered out, as they were not considered by our variant calling pipeline, and mismatched bases are shaded by quality score (more transparent = lower base quality). [file elife-46922-supp3.zip › supp_file_3/chr6_167,115,463_167,115,503.png]

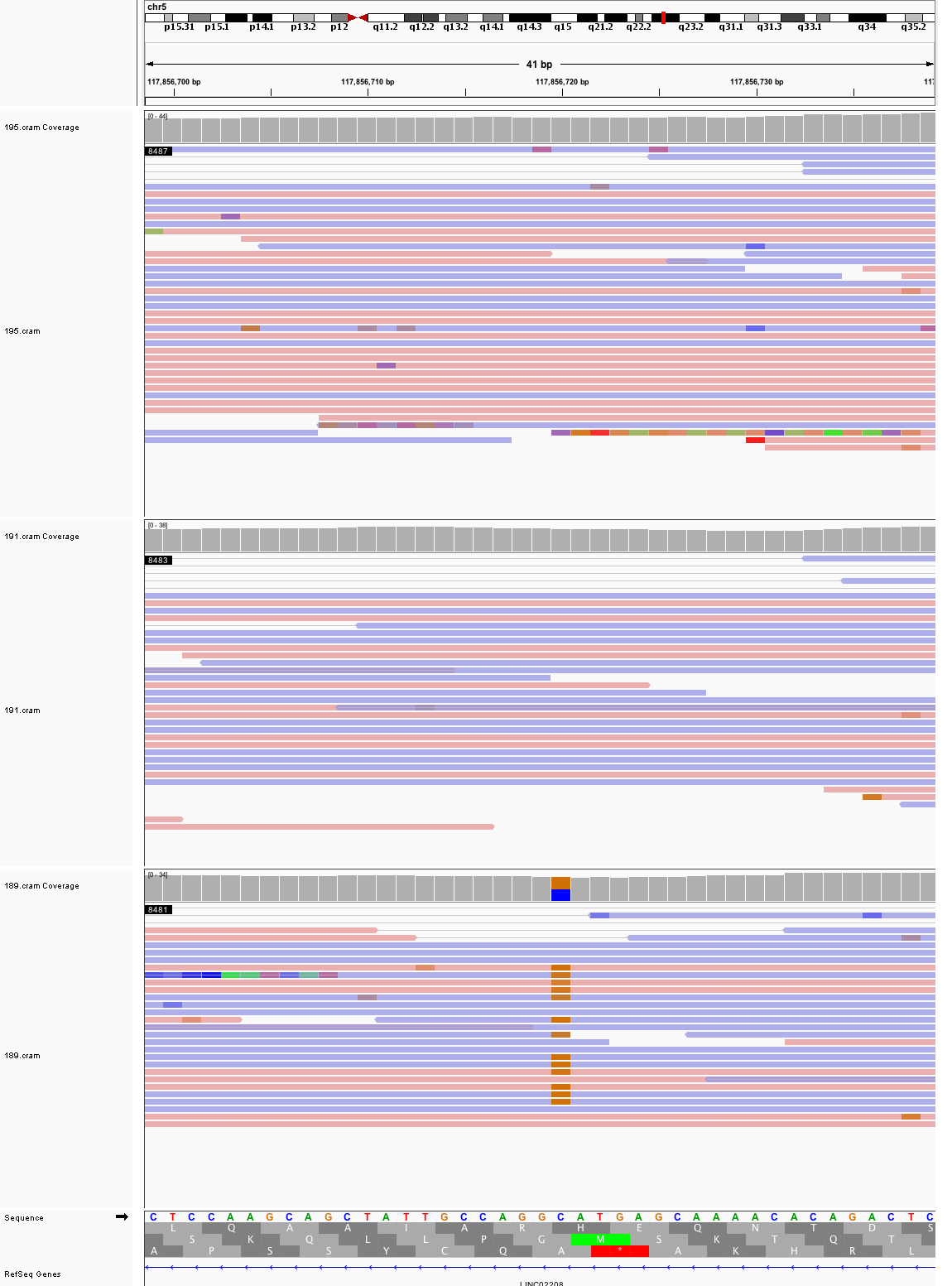

Supplement: Supplementary file 3. — DNMs identified in the third generation In each image, the first two tracks contain alignments from the second-generation parents, and the third track contains the alignments for the third-generation child. Reads with mapping quality <20 are filtered out, as they were not considered by our variant calling pipeline, and mismatched bases are shaded by quality score (more transparent = lower base quality). [file elife-46922-supp3.zip › supp_file_3/chr5_117,856,699_117,856,739.png]

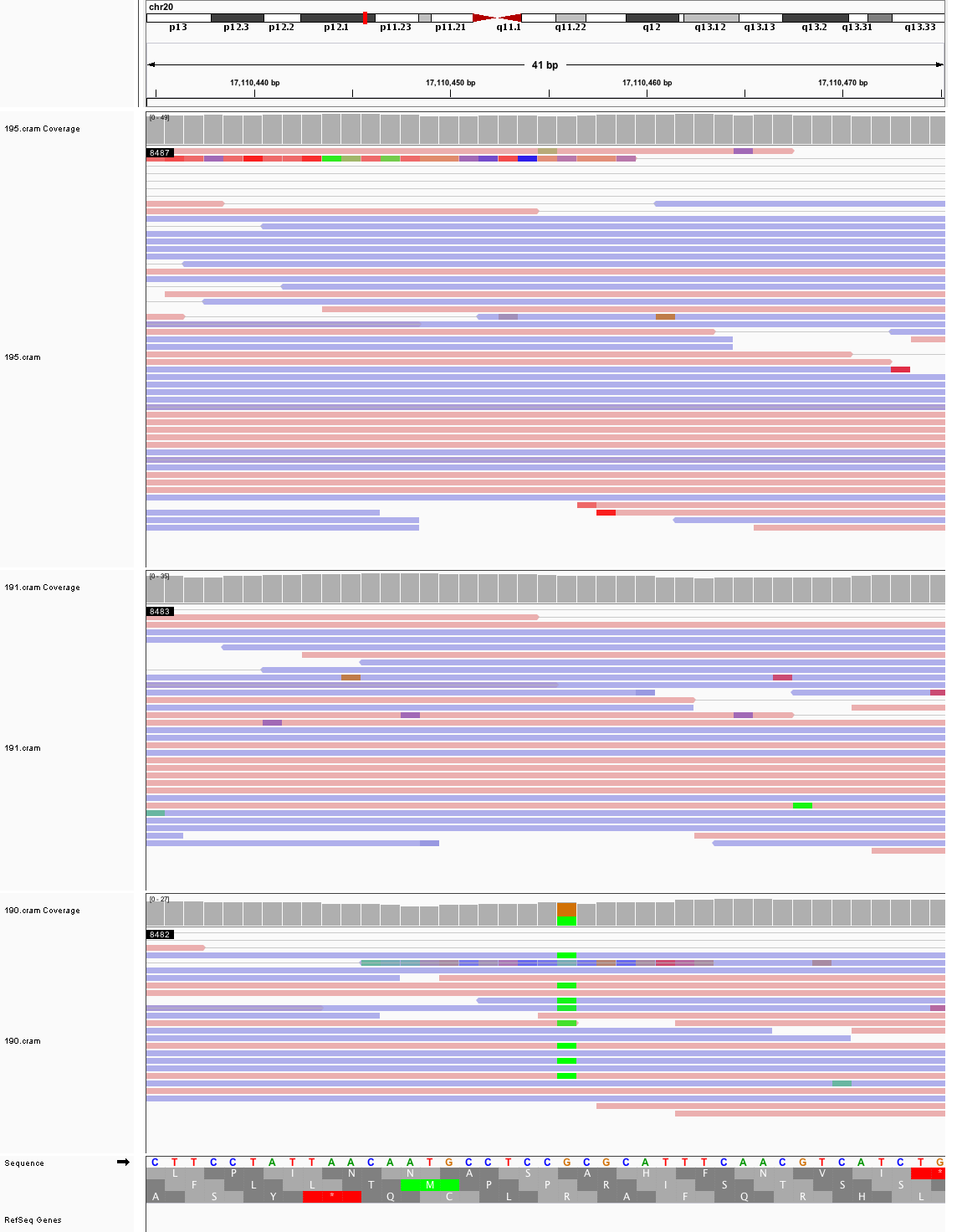

Supplement: Supplementary file 3. — DNMs identified in the third generation In each image, the first two tracks contain alignments from the second-generation parents, and the third track contains the alignments for the third-generation child. Reads with mapping quality <20 are filtered out, as they were not considered by our variant calling pipeline, and mismatched bases are shaded by quality score (more transparent = lower base quality). [file elife-46922-supp3.zip › supp_file_3/chr20_17,110,435_17,110,475.png]

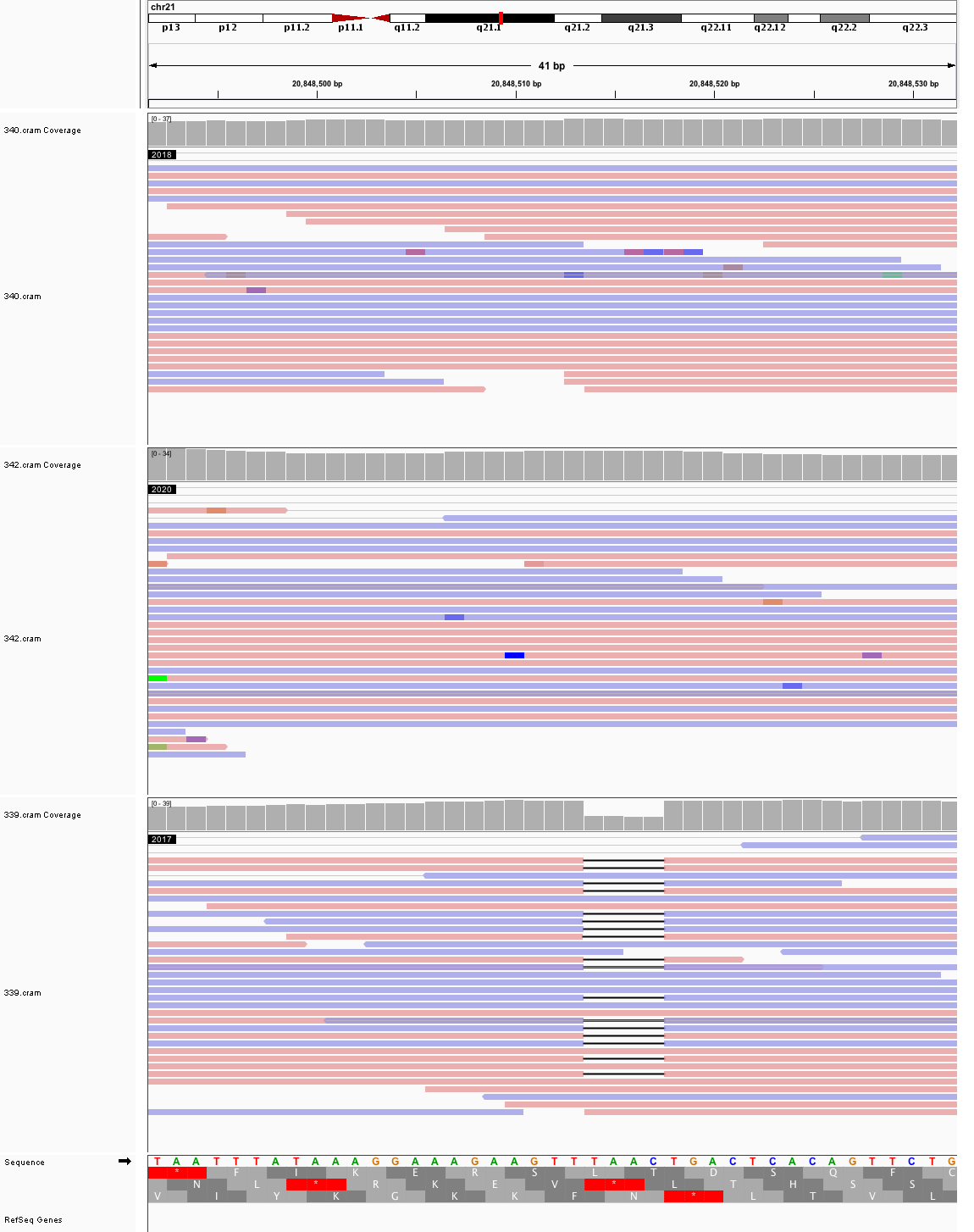

Supplement: Supplementary file 3. — DNMs identified in the third generation In each image, the first two tracks contain alignments from the second-generation parents, and the third track contains the alignments for the third-generation child. Reads with mapping quality <20 are filtered out, as they were not considered by our variant calling pipeline, and mismatched bases are shaded by quality score (more transparent = lower base quality). [file elife-46922-supp3.zip › supp_file_3/chr21_20,848,492_20,848,532.png]

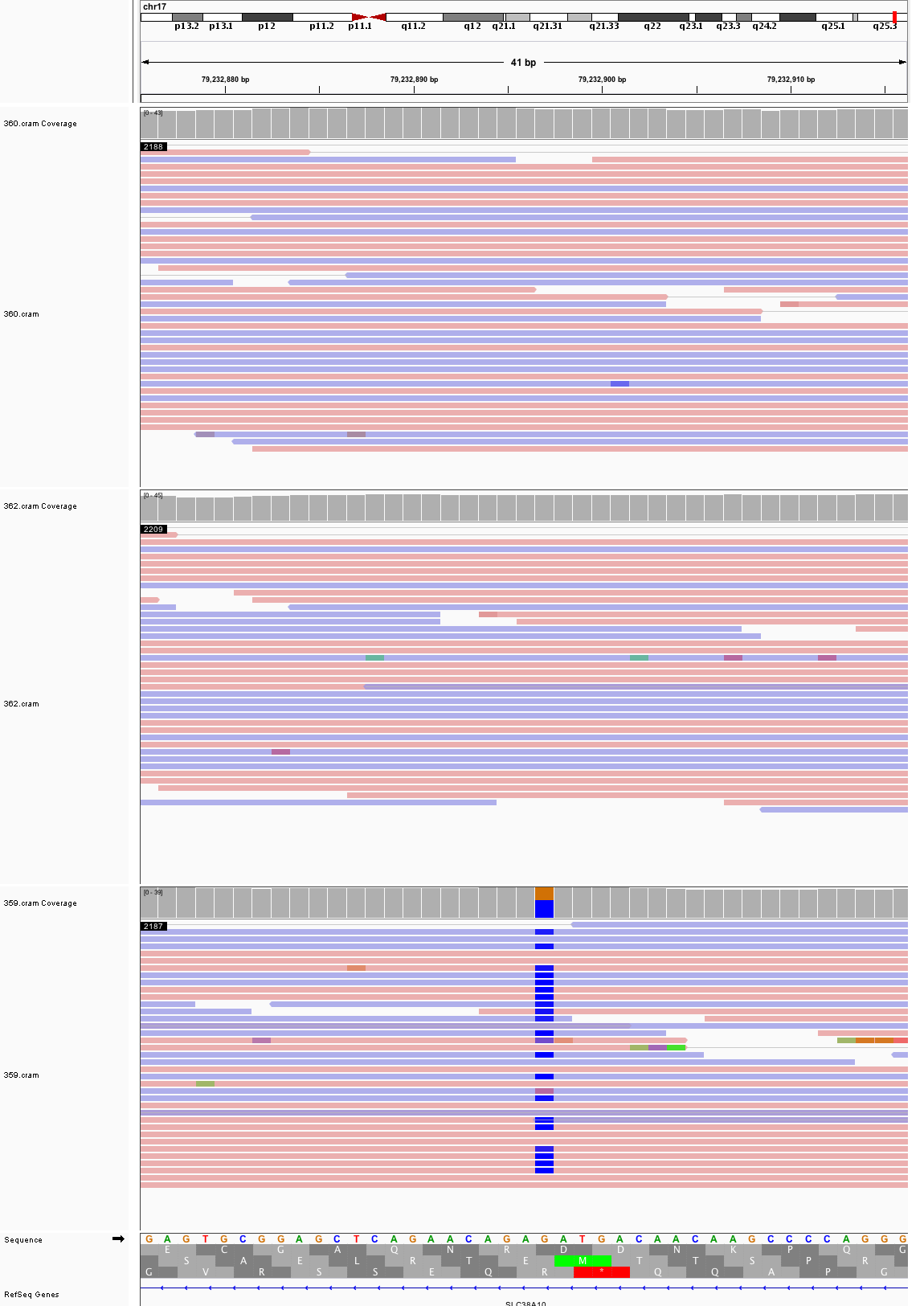

Supplement: Supplementary file 3. — DNMs identified in the third generation In each image, the first two tracks contain alignments from the second-generation parents, and the third track contains the alignments for the third-generation child. Reads with mapping quality <20 are filtered out, as they were not considered by our variant calling pipeline, and mismatched bases are shaded by quality score (more transparent = lower base quality). [file elife-46922-supp3.zip › supp_file_3/chr17_79,232,876_79,232,916.png]

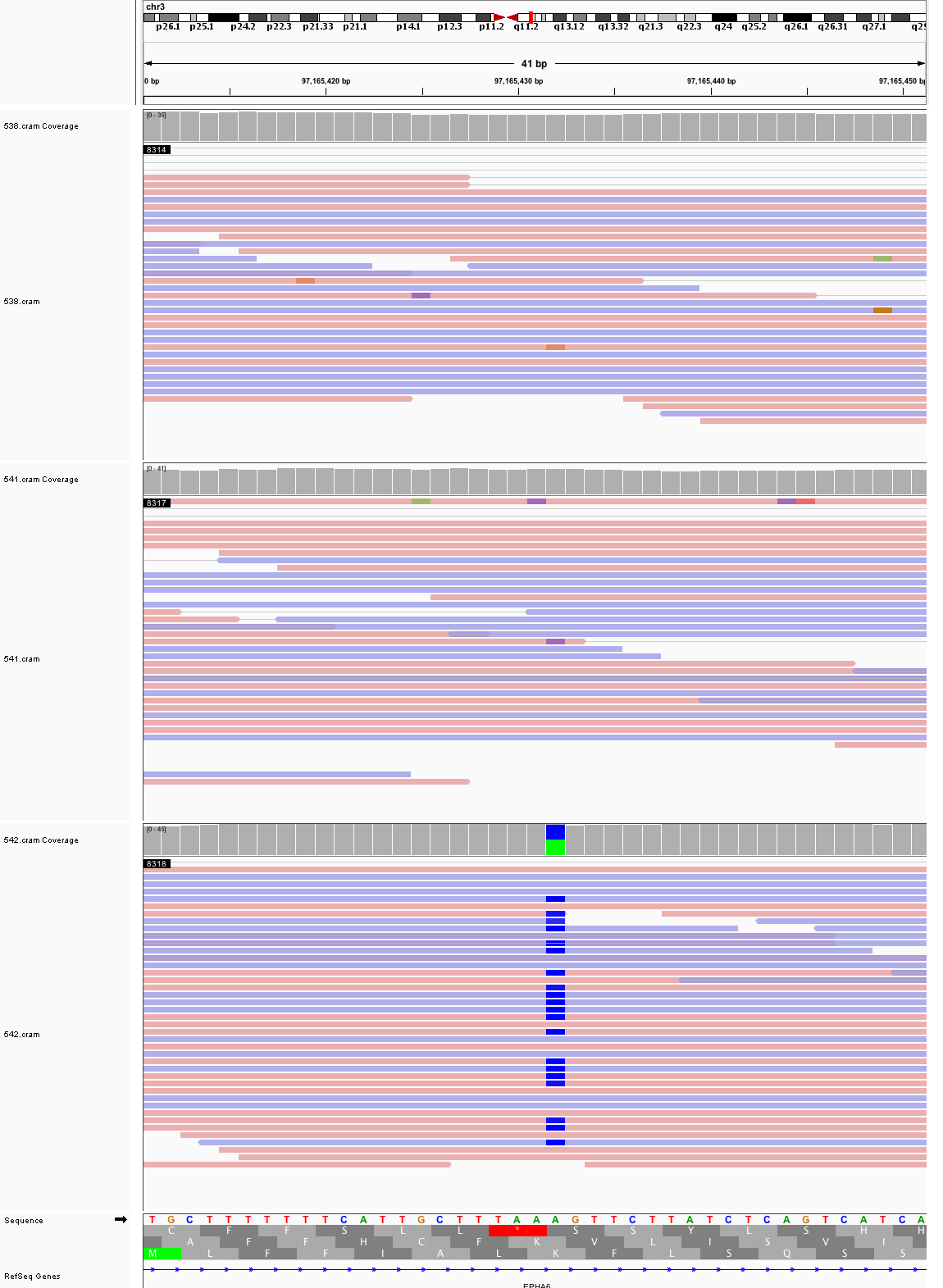

Supplement: Supplementary file 3. — DNMs identified in the third generation In each image, the first two tracks contain alignments from the second-generation parents, and the third track contains the alignments for the third-generation child. Reads with mapping quality <20 are filtered out, as they were not considered by our variant calling pipeline, and mismatched bases are shaded by quality score (more transparent = lower base quality). [file elife-46922-supp3.zip › supp_file_3/chr3_97,165,411_97,165,451.png]

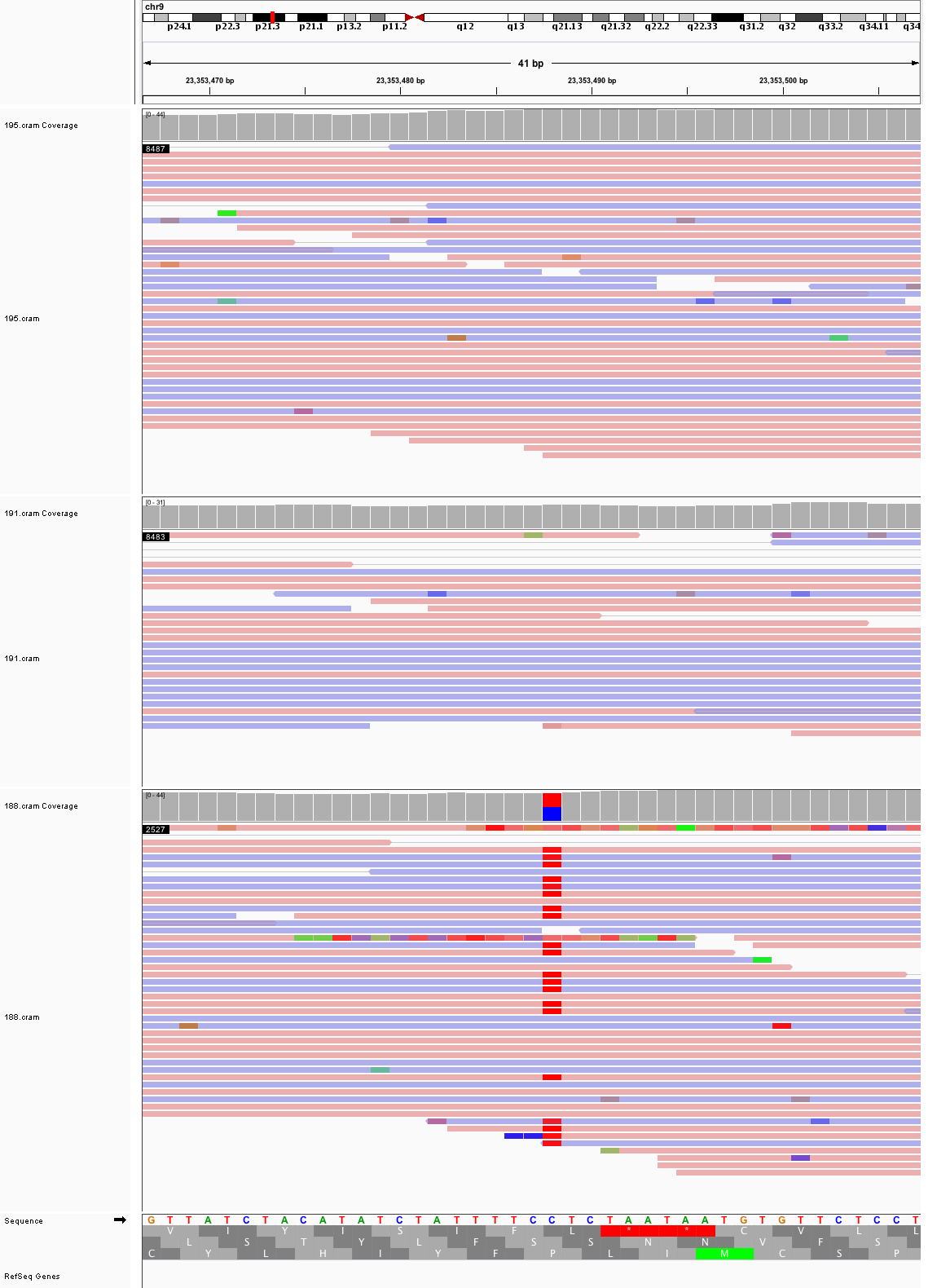

Supplement: Supplementary file 3. — DNMs identified in the third generation In each image, the first two tracks contain alignments from the second-generation parents, and the third track contains the alignments for the third-generation child. Reads with mapping quality <20 are filtered out, as they were not considered by our variant calling pipeline, and mismatched bases are shaded by quality score (more transparent = lower base quality). [file elife-46922-supp3.zip › supp_file_3/chr9_23,353,467_23,353,507.png]

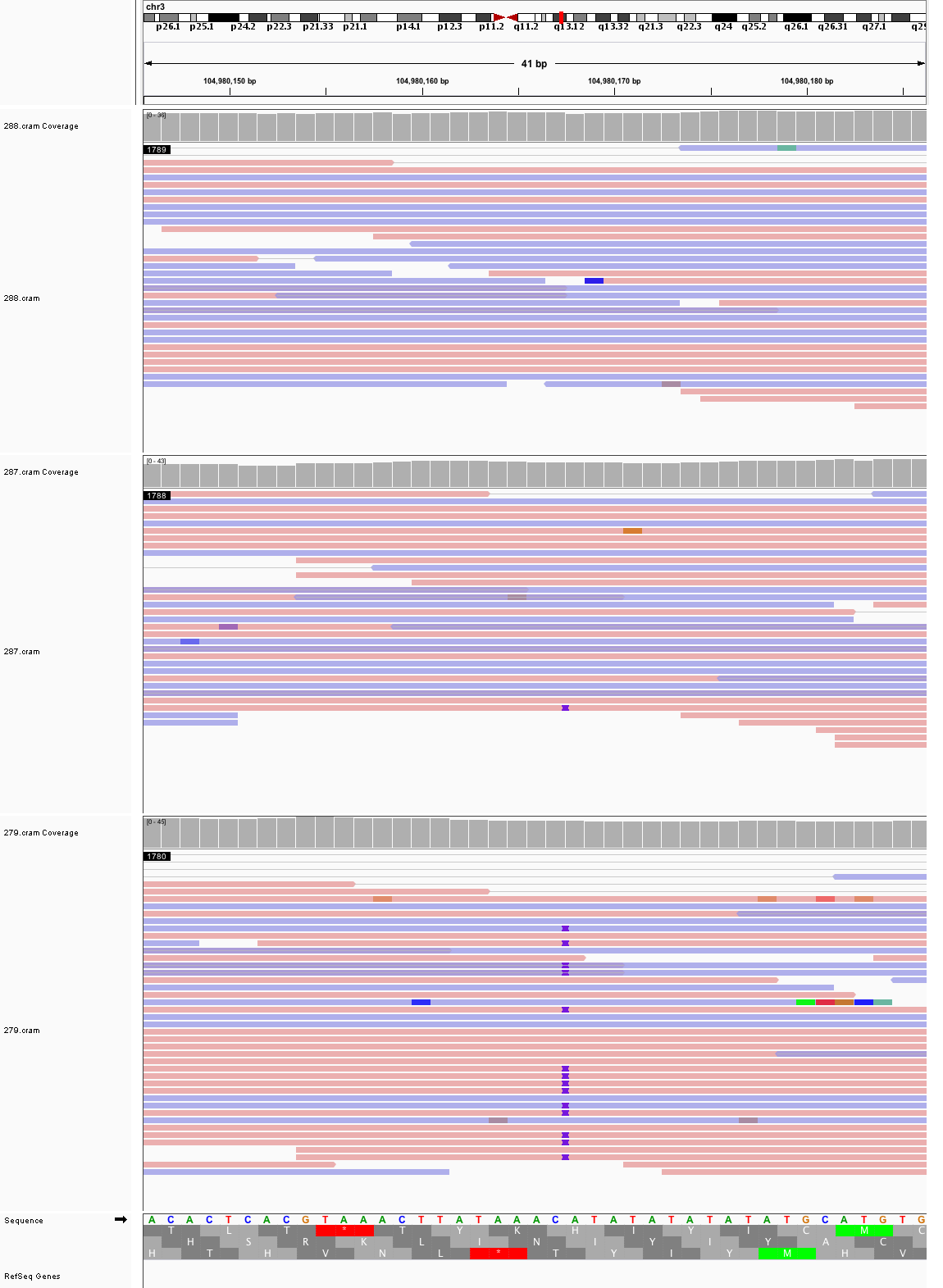

Supplement: Supplementary file 3. — DNMs identified in the third generation In each image, the first two tracks contain alignments from the second-generation parents, and the third track contains the alignments for the third-generation child. Reads with mapping quality <20 are filtered out, as they were not considered by our variant calling pipeline, and mismatched bases are shaded by quality score (more transparent = lower base quality). [file elife-46922-supp3.zip › supp_file_3/chr3_104,980,146_104,980,186.png]

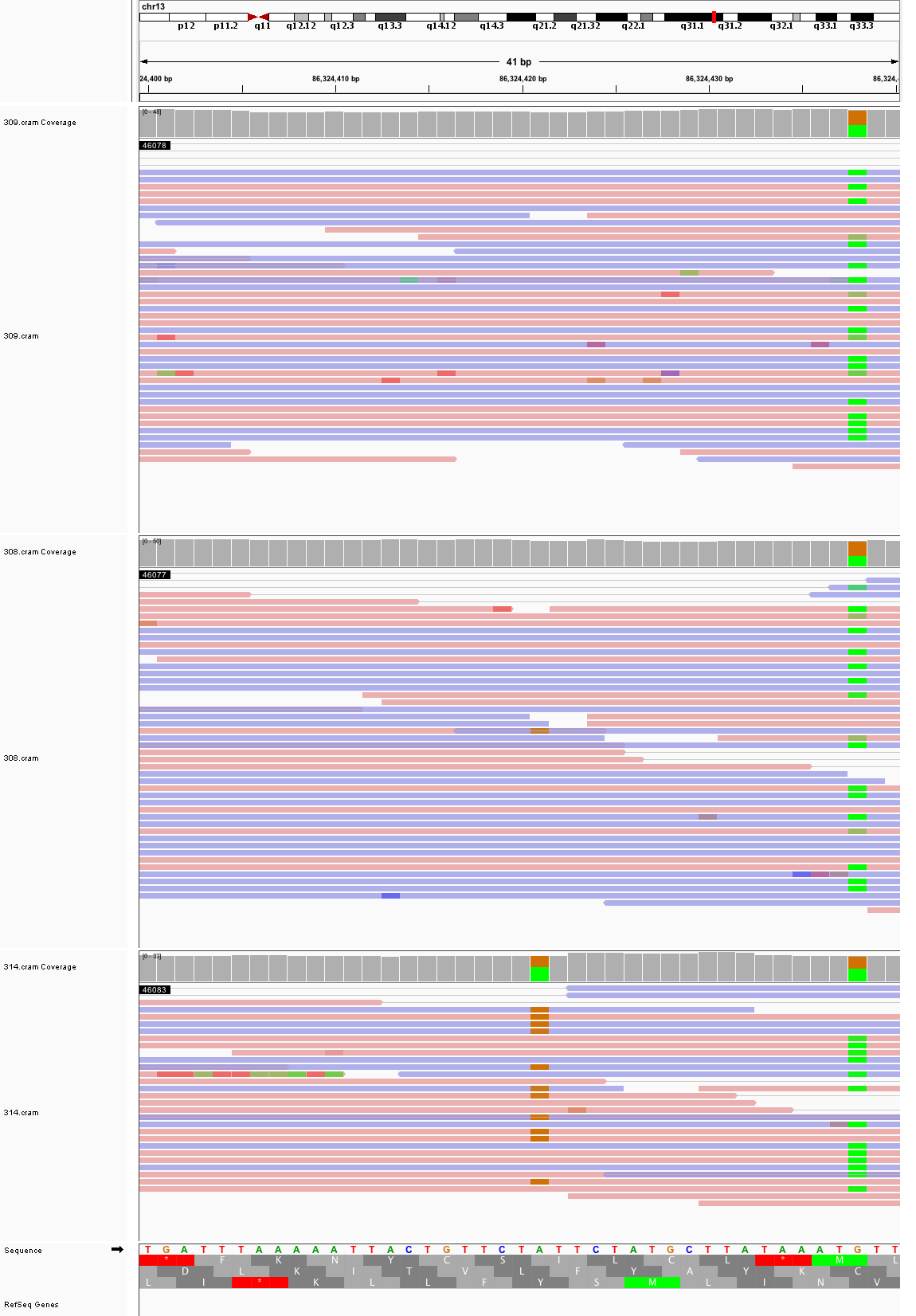

Supplement: Supplementary file 3. — DNMs identified in the third generation In each image, the first two tracks contain alignments from the second-generation parents, and the third track contains the alignments for the third-generation child. Reads with mapping quality <20 are filtered out, as they were not considered by our variant calling pipeline, and mismatched bases are shaded by quality score (more transparent = lower base quality). [file elife-46922-supp3.zip › supp_file_3/chr13_86,324,400_86,324,440.png]

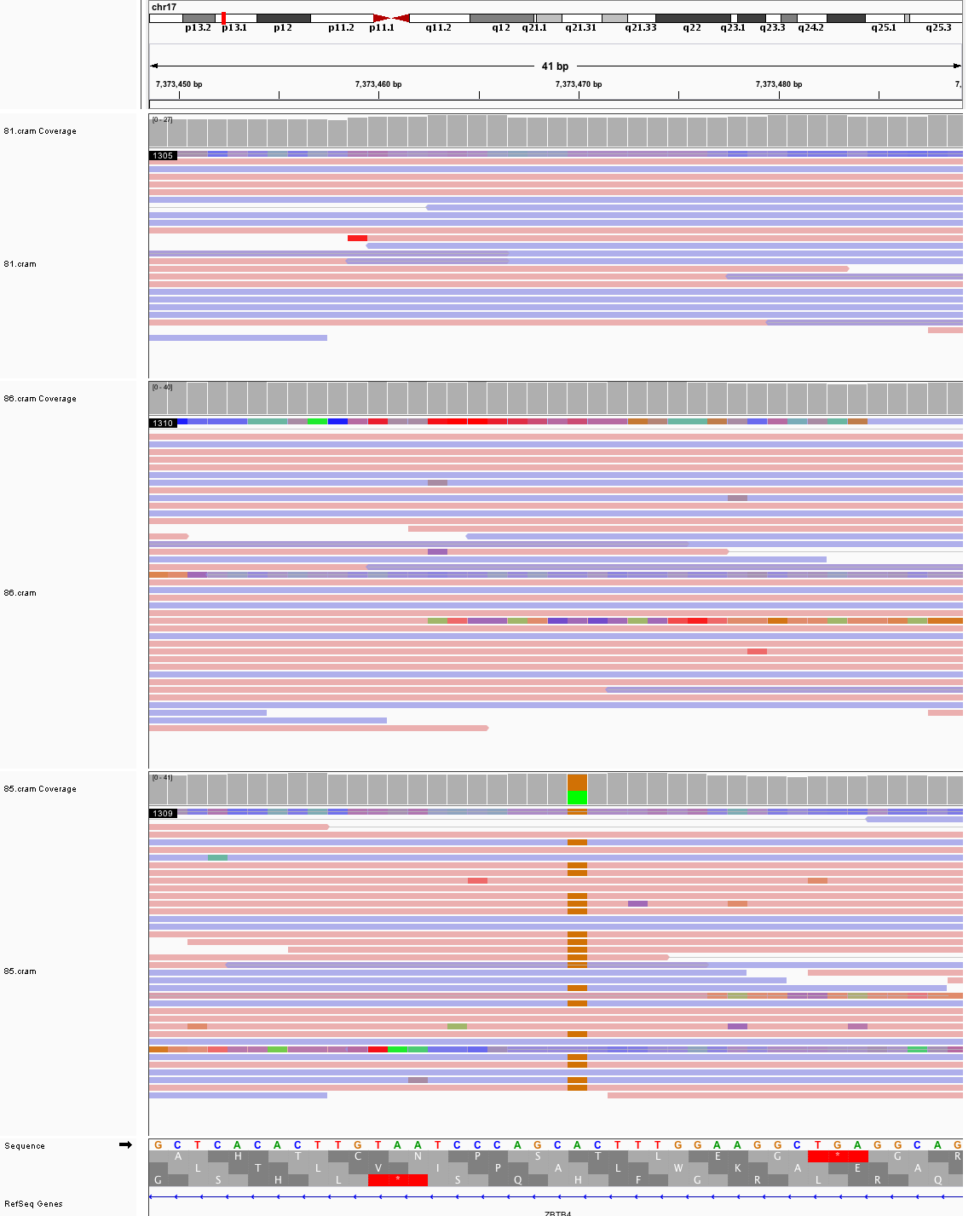

Supplement: Supplementary file 3. — DNMs identified in the third generation In each image, the first two tracks contain alignments from the second-generation parents, and the third track contains the alignments for the third-generation child. Reads with mapping quality <20 are filtered out, as they were not considered by our variant calling pipeline, and mismatched bases are shaded by quality score (more transparent = lower base quality). [file elife-46922-supp3.zip › supp_file_3/chr17_7,373,449_7,373,489.png]

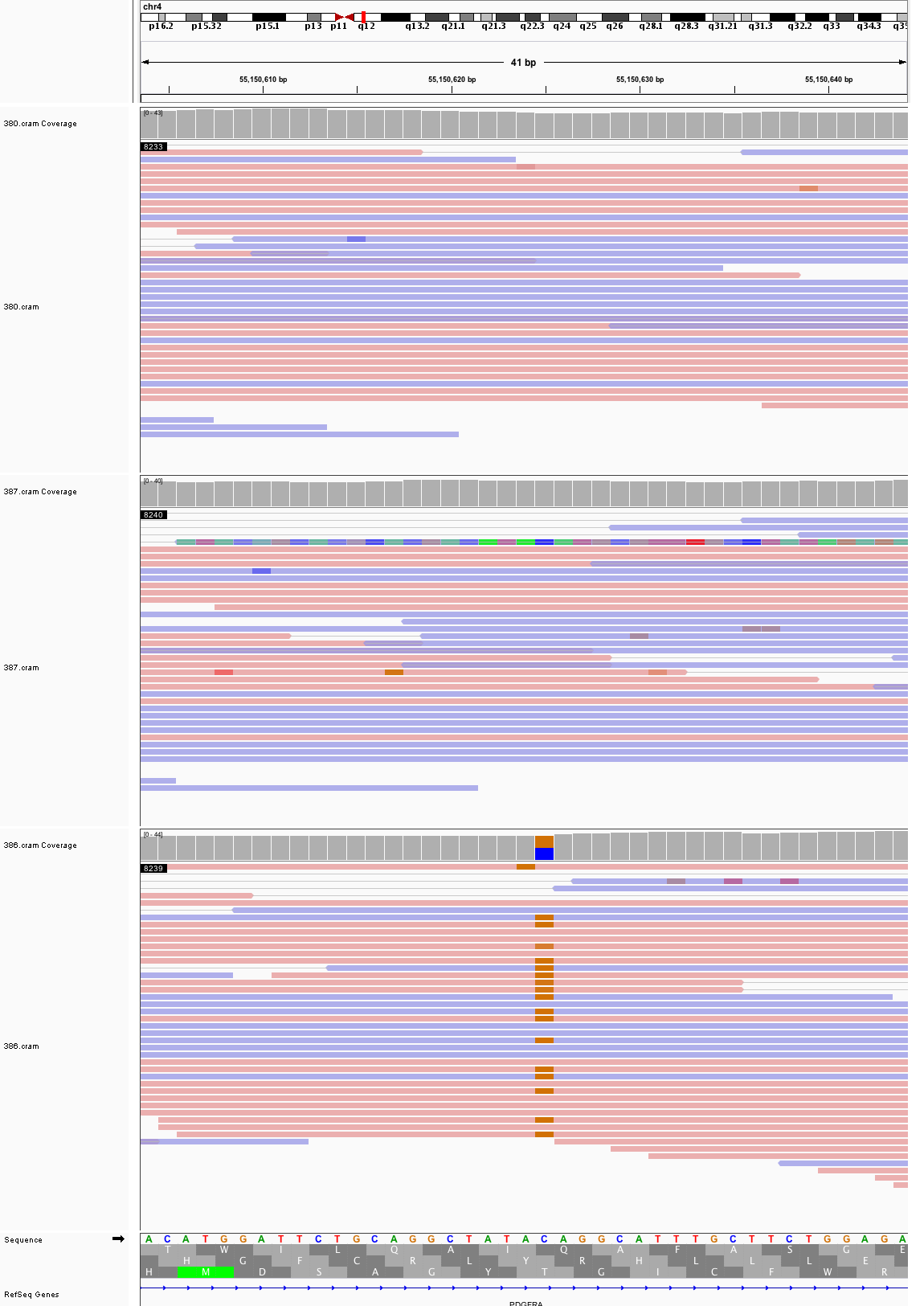

Supplement: Supplementary file 3. — DNMs identified in the third generation In each image, the first two tracks contain alignments from the second-generation parents, and the third track contains the alignments for the third-generation child. Reads with mapping quality <20 are filtered out, as they were not considered by our variant calling pipeline, and mismatched bases are shaded by quality score (more transparent = lower base quality). [file elife-46922-supp3.zip › supp_file_3/chr4_55,150,604_55,150,644.png]

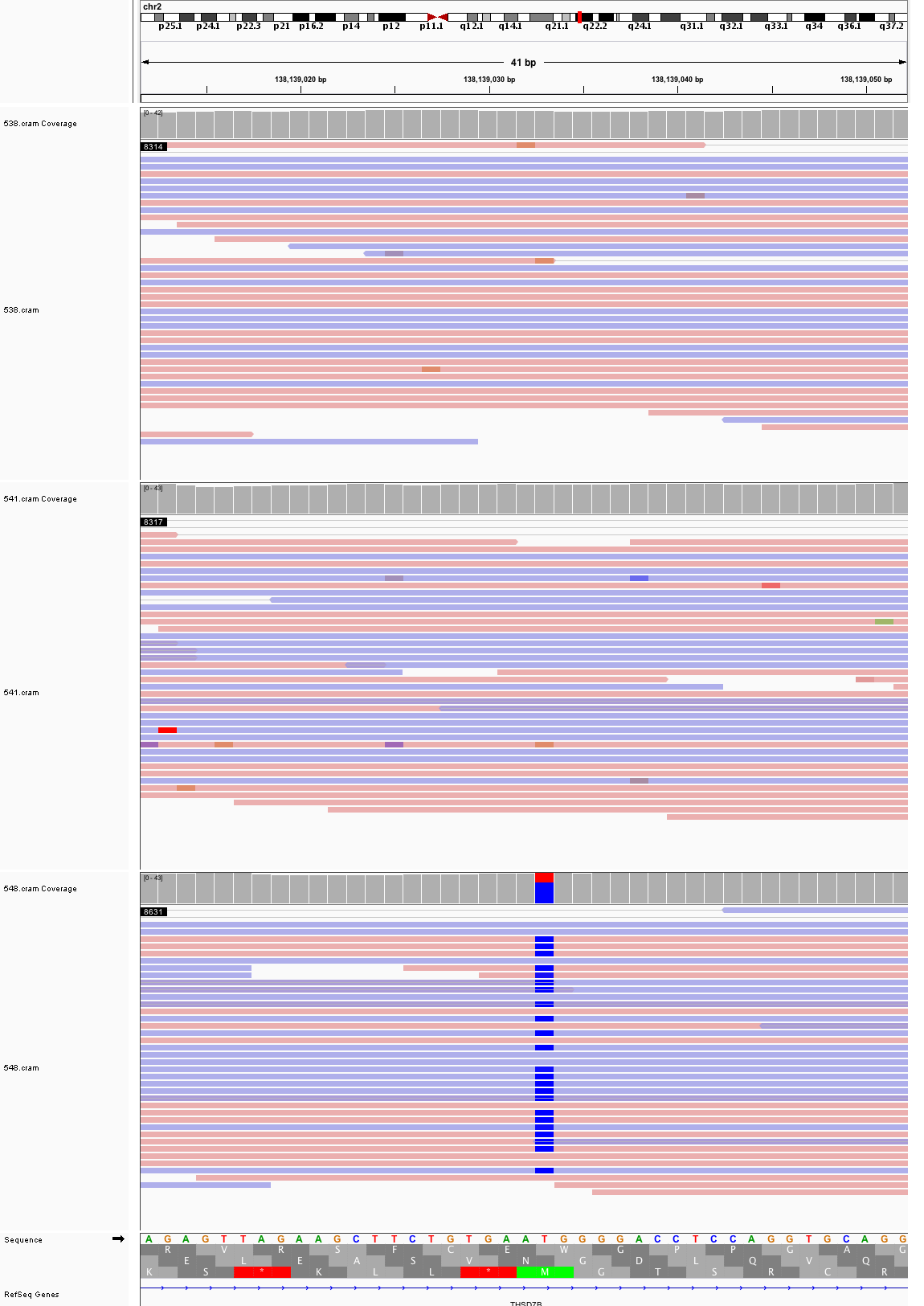

Supplement: Supplementary file 3. — DNMs identified in the third generation In each image, the first two tracks contain alignments from the second-generation parents, and the third track contains the alignments for the third-generation child. Reads with mapping quality <20 are filtered out, as they were not considered by our variant calling pipeline, and mismatched bases are shaded by quality score (more transparent = lower base quality). [file elife-46922-supp3.zip › supp_file_3/chr2_138,139,012_138,139,052.png]

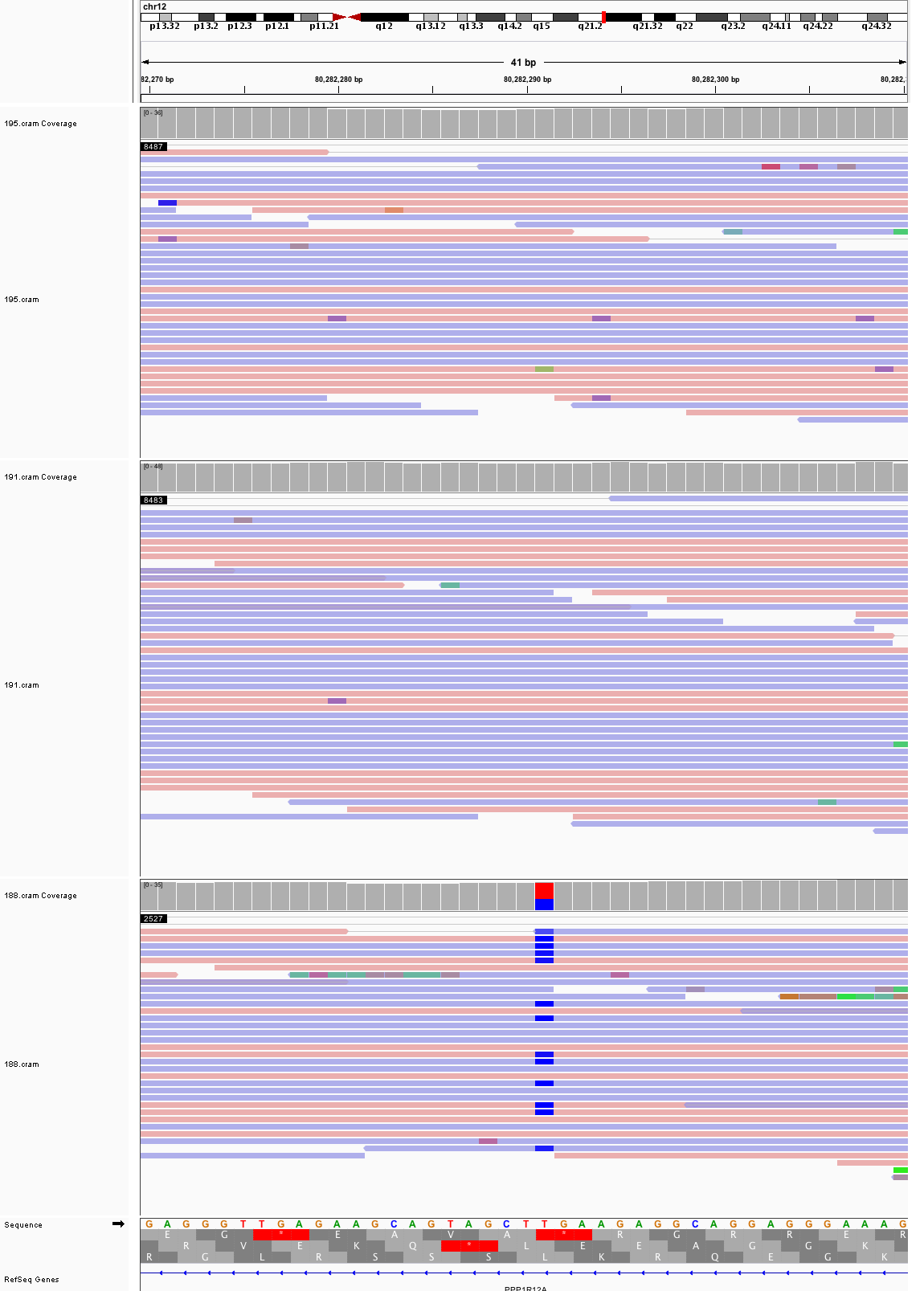

Supplement: Supplementary file 3. — DNMs identified in the third generation In each image, the first two tracks contain alignments from the second-generation parents, and the third track contains the alignments for the third-generation child. Reads with mapping quality <20 are filtered out, as they were not considered by our variant calling pipeline, and mismatched bases are shaded by quality score (more transparent = lower base quality). [file elife-46922-supp3.zip › supp_file_3/chr12_80,282,270_80,282,310.png]

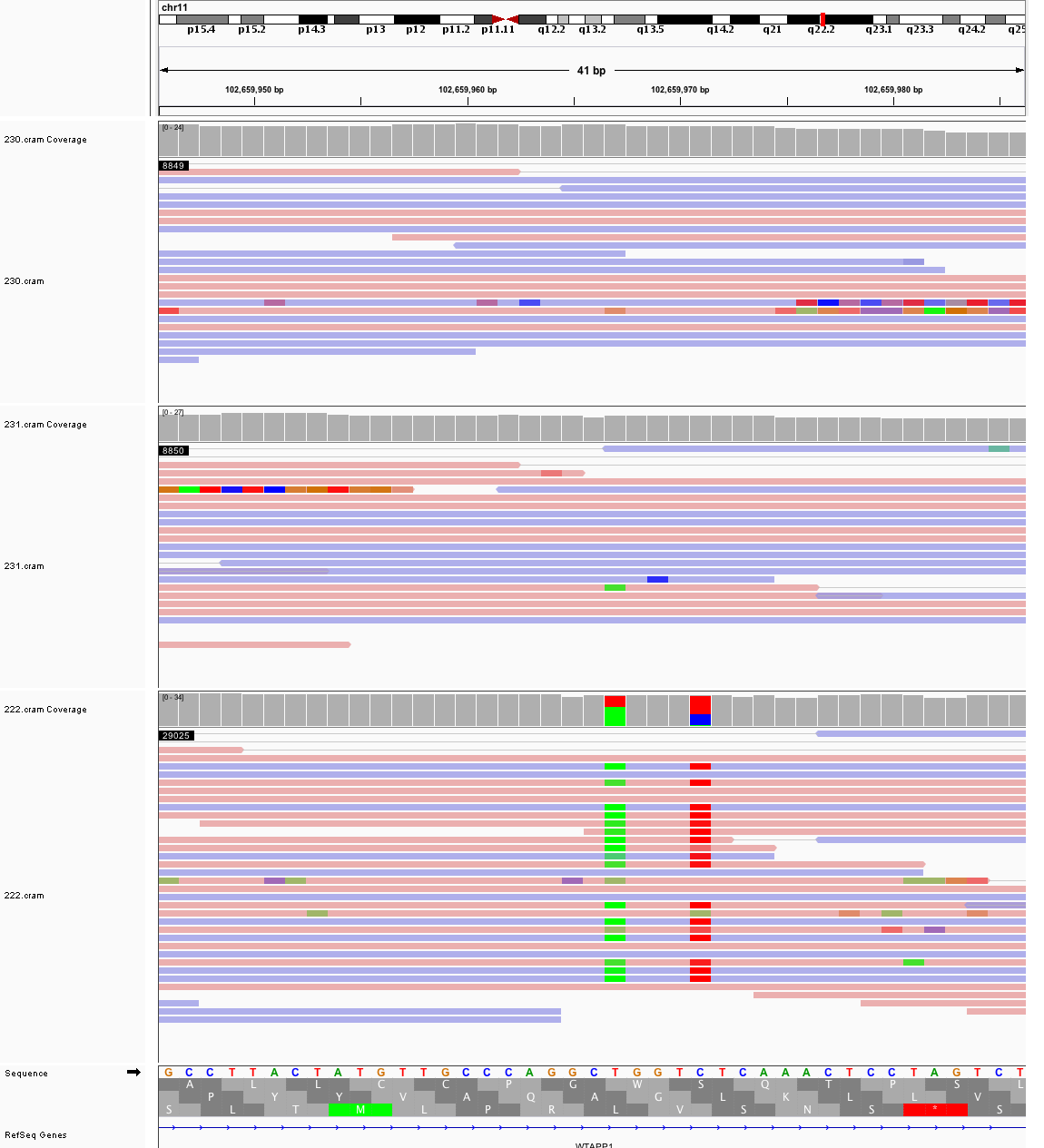

Supplement: Supplementary file 3. — DNMs identified in the third generation In each image, the first two tracks contain alignments from the second-generation parents, and the third track contains the alignments for the third-generation child. Reads with mapping quality <20 are filtered out, as they were not considered by our variant calling pipeline, and mismatched bases are shaded by quality score (more transparent = lower base quality). [file elife-46922-supp3.zip › supp_file_3/chr11_102,659,946_102,659,986.png]

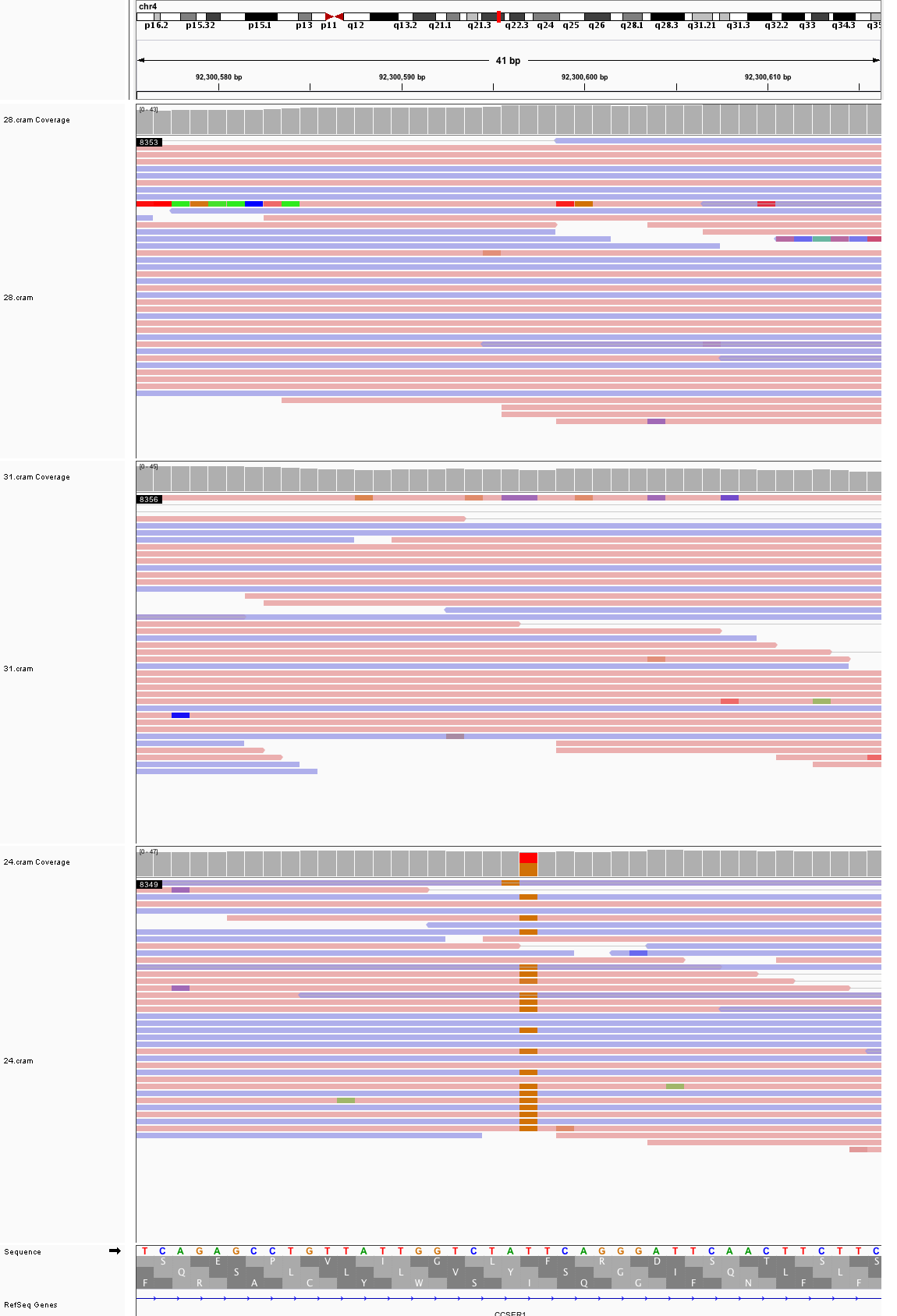

Supplement: Supplementary file 3. — DNMs identified in the third generation In each image, the first two tracks contain alignments from the second-generation parents, and the third track contains the alignments for the third-generation child. Reads with mapping quality <20 are filtered out, as they were not considered by our variant calling pipeline, and mismatched bases are shaded by quality score (more transparent = lower base quality). [file elife-46922-supp3.zip › supp_file_3/chr4_92,300,576_92,300,616.png]

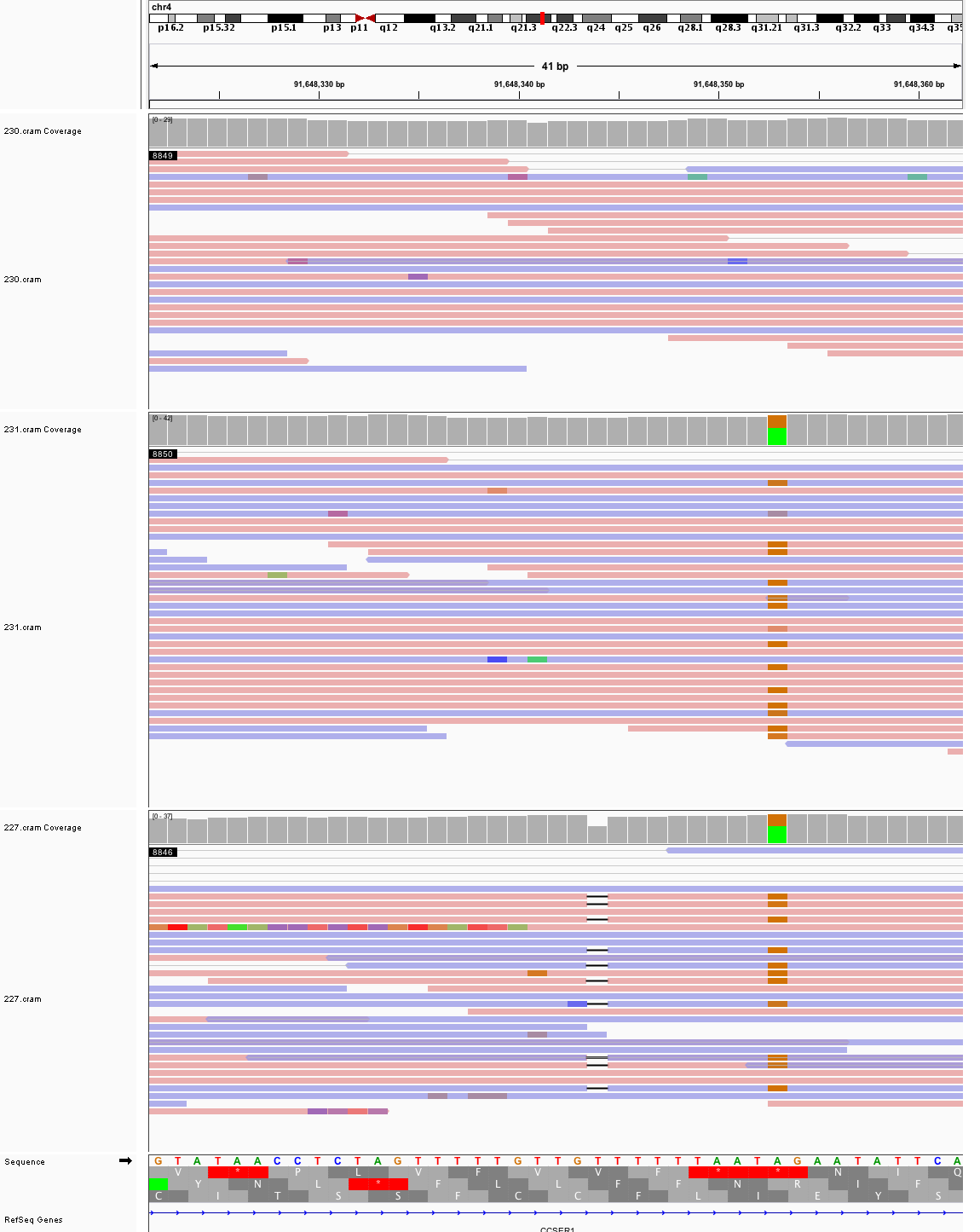

Supplement: Supplementary file 3. — DNMs identified in the third generation In each image, the first two tracks contain alignments from the second-generation parents, and the third track contains the alignments for the third-generation child. Reads with mapping quality <20 are filtered out, as they were not considered by our variant calling pipeline, and mismatched bases are shaded by quality score (more transparent = lower base quality). [file elife-46922-supp3.zip › supp_file_3/chr4_91,648,322_91,648,362.png]

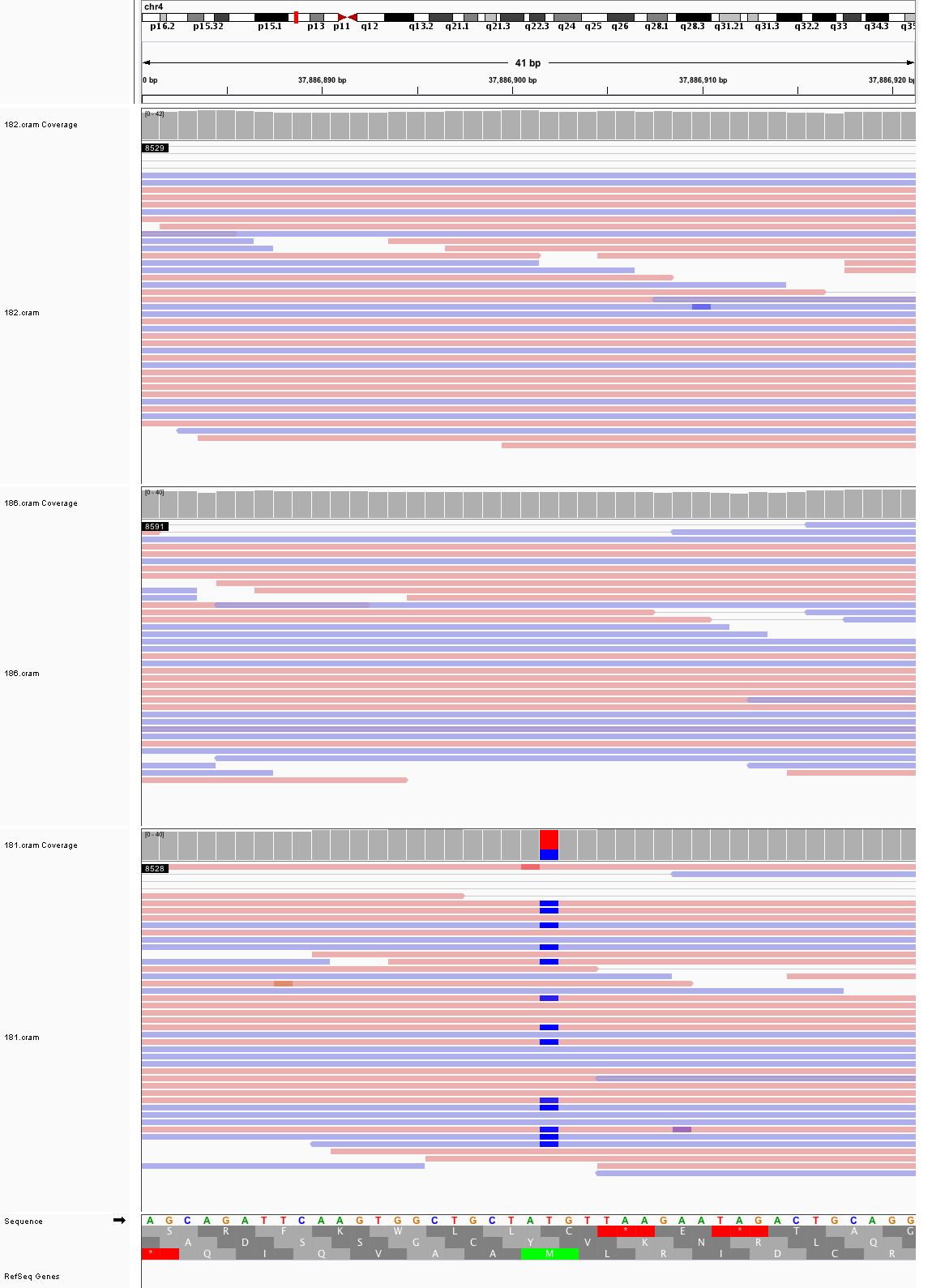

Supplement: Supplementary file 3. — DNMs identified in the third generation In each image, the first two tracks contain alignments from the second-generation parents, and the third track contains the alignments for the third-generation child. Reads with mapping quality <20 are filtered out, as they were not considered by our variant calling pipeline, and mismatched bases are shaded by quality score (more transparent = lower base quality). [file elife-46922-supp3.zip › supp_file_3/chr4_37,886,881_37,886,921.png]

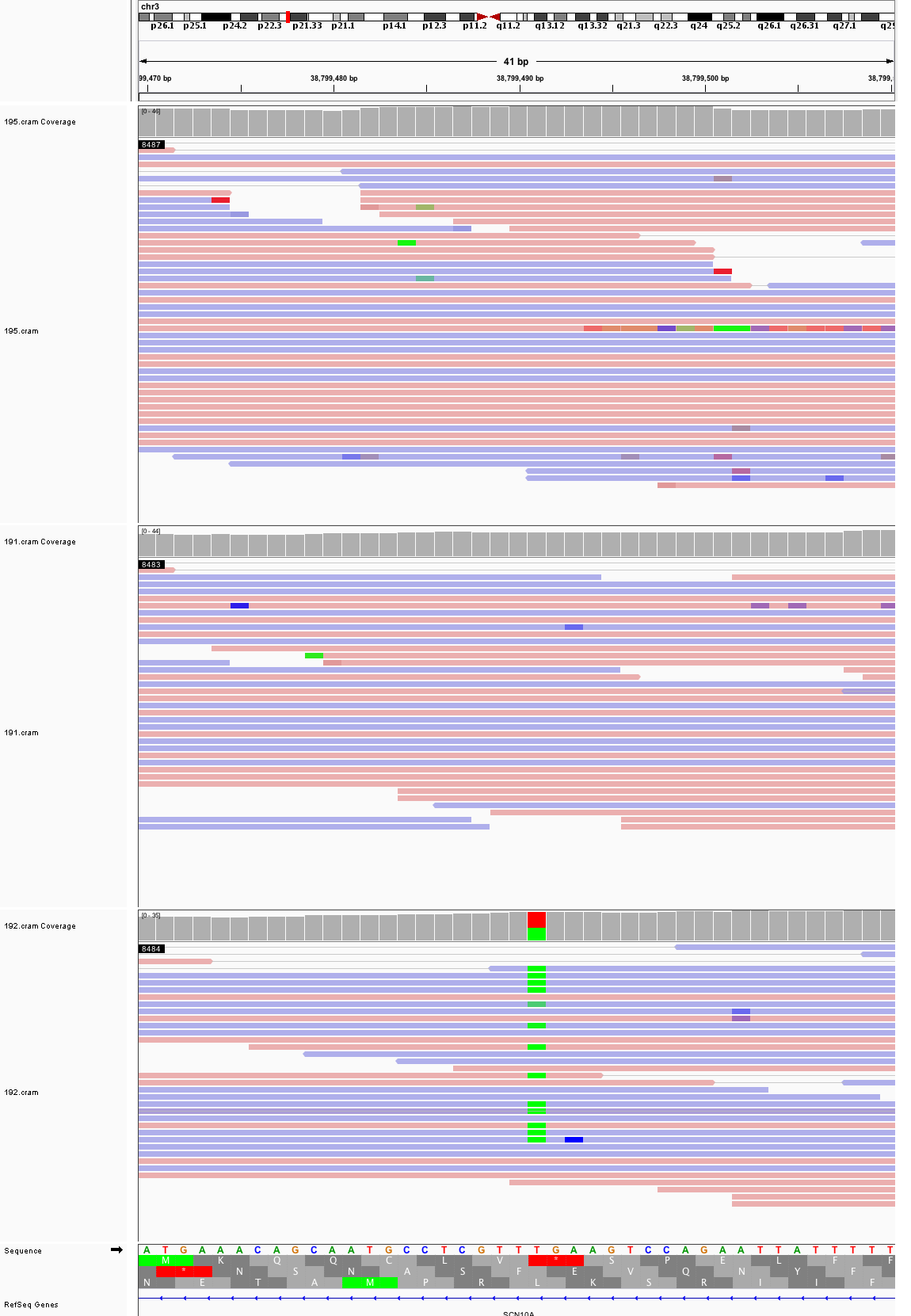

Supplement: Supplementary file 3. — DNMs identified in the third generation In each image, the first two tracks contain alignments from the second-generation parents, and the third track contains the alignments for the third-generation child. Reads with mapping quality <20 are filtered out, as they were not considered by our variant calling pipeline, and mismatched bases are shaded by quality score (more transparent = lower base quality). [file elife-46922-supp3.zip › supp_file_3/chr3_38,799,470_38,799,510.png]

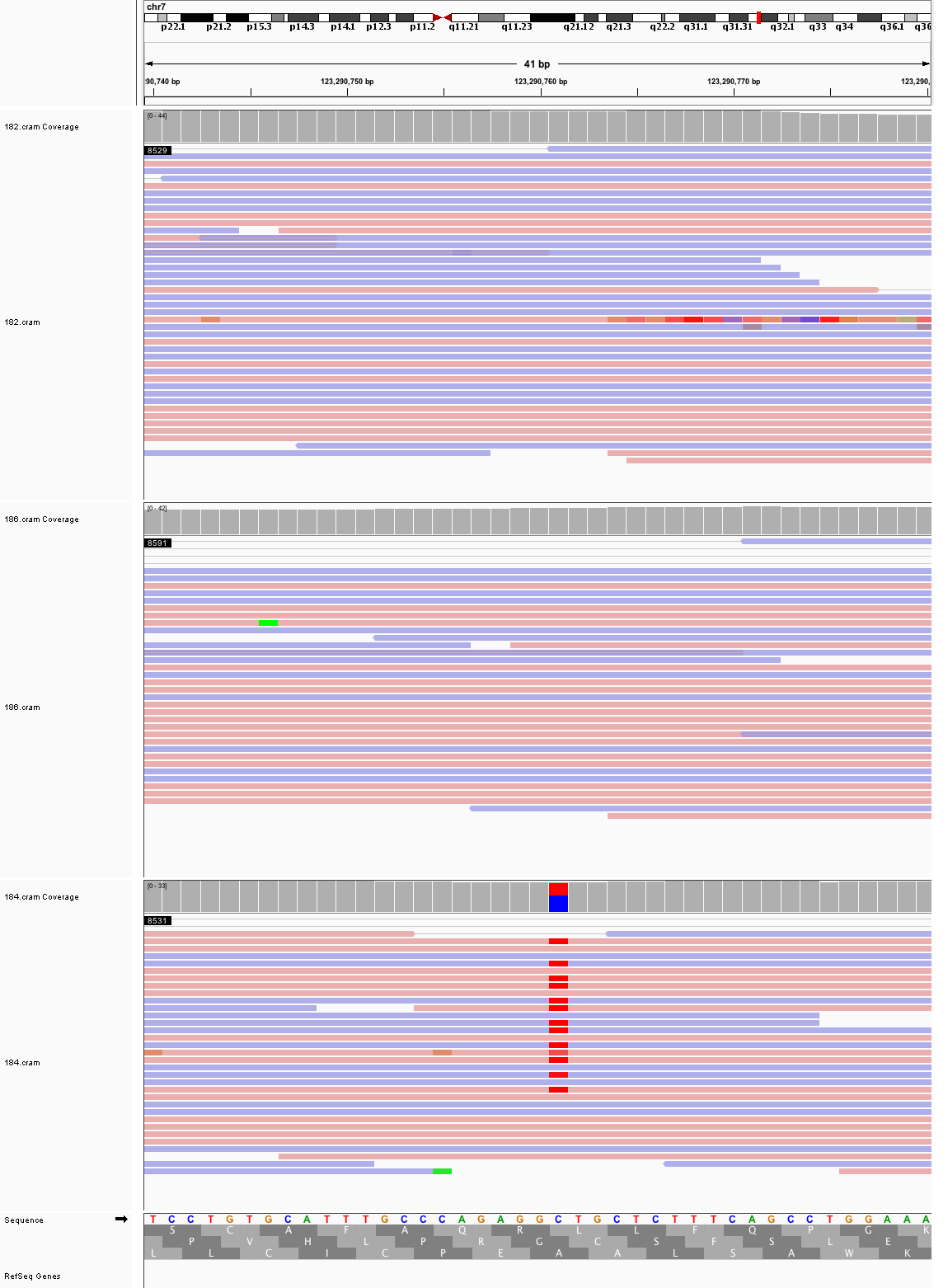

Supplement: Supplementary file 3. — DNMs identified in the third generation In each image, the first two tracks contain alignments from the second-generation parents, and the third track contains the alignments for the third-generation child. Reads with mapping quality <20 are filtered out, as they were not considered by our variant calling pipeline, and mismatched bases are shaded by quality score (more transparent = lower base quality). [file elife-46922-supp3.zip › supp_file_3/chr7_123,290,740_123,290,780.png]

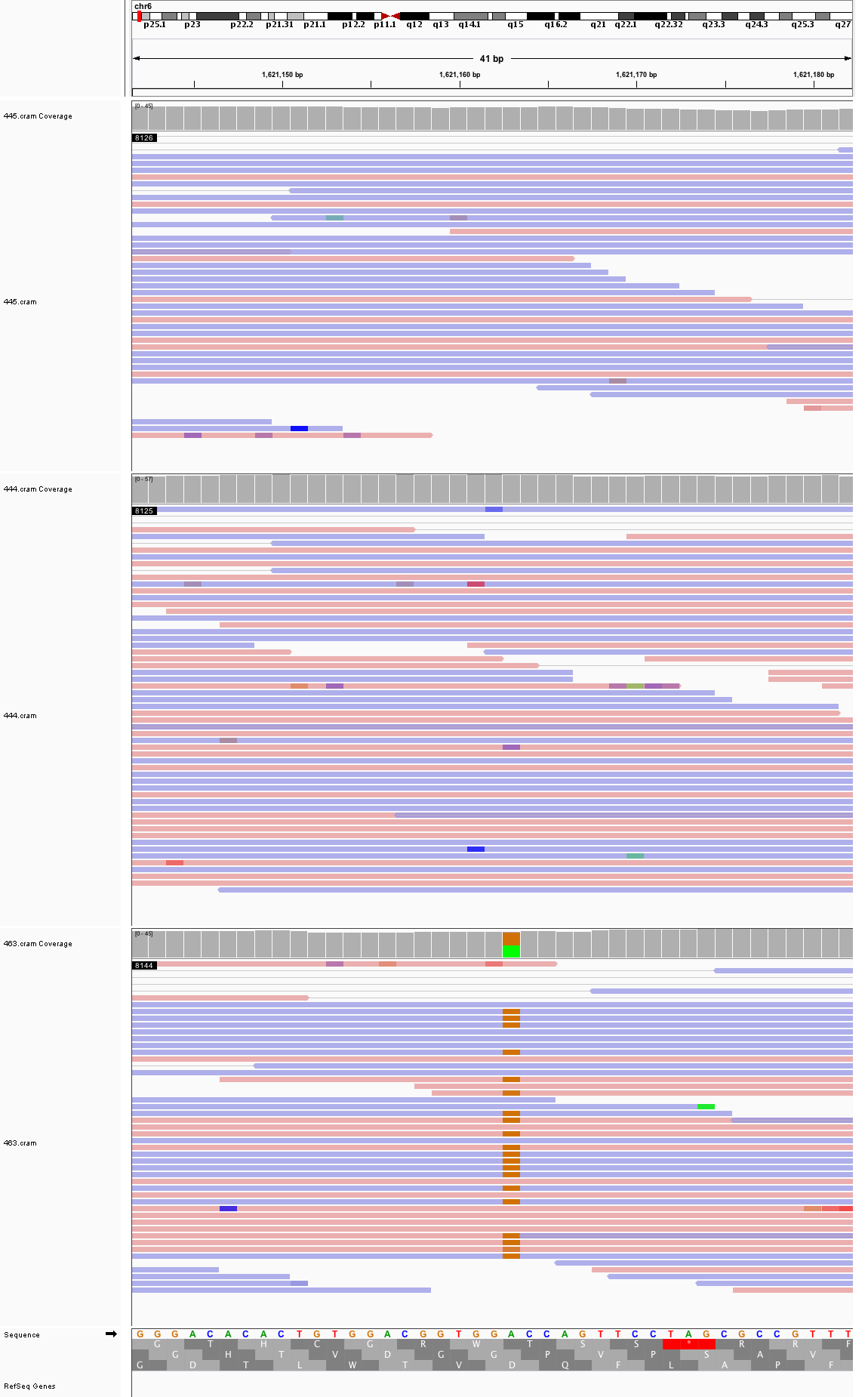

Supplement: Supplementary file 3. — DNMs identified in the third generation In each image, the first two tracks contain alignments from the second-generation parents, and the third track contains the alignments for the third-generation child. Reads with mapping quality <20 are filtered out, as they were not considered by our variant calling pipeline, and mismatched bases are shaded by quality score (more transparent = lower base quality). [file elife-46922-supp3.zip › supp_file_3/chr6_1,621,142_1,621,182.png]

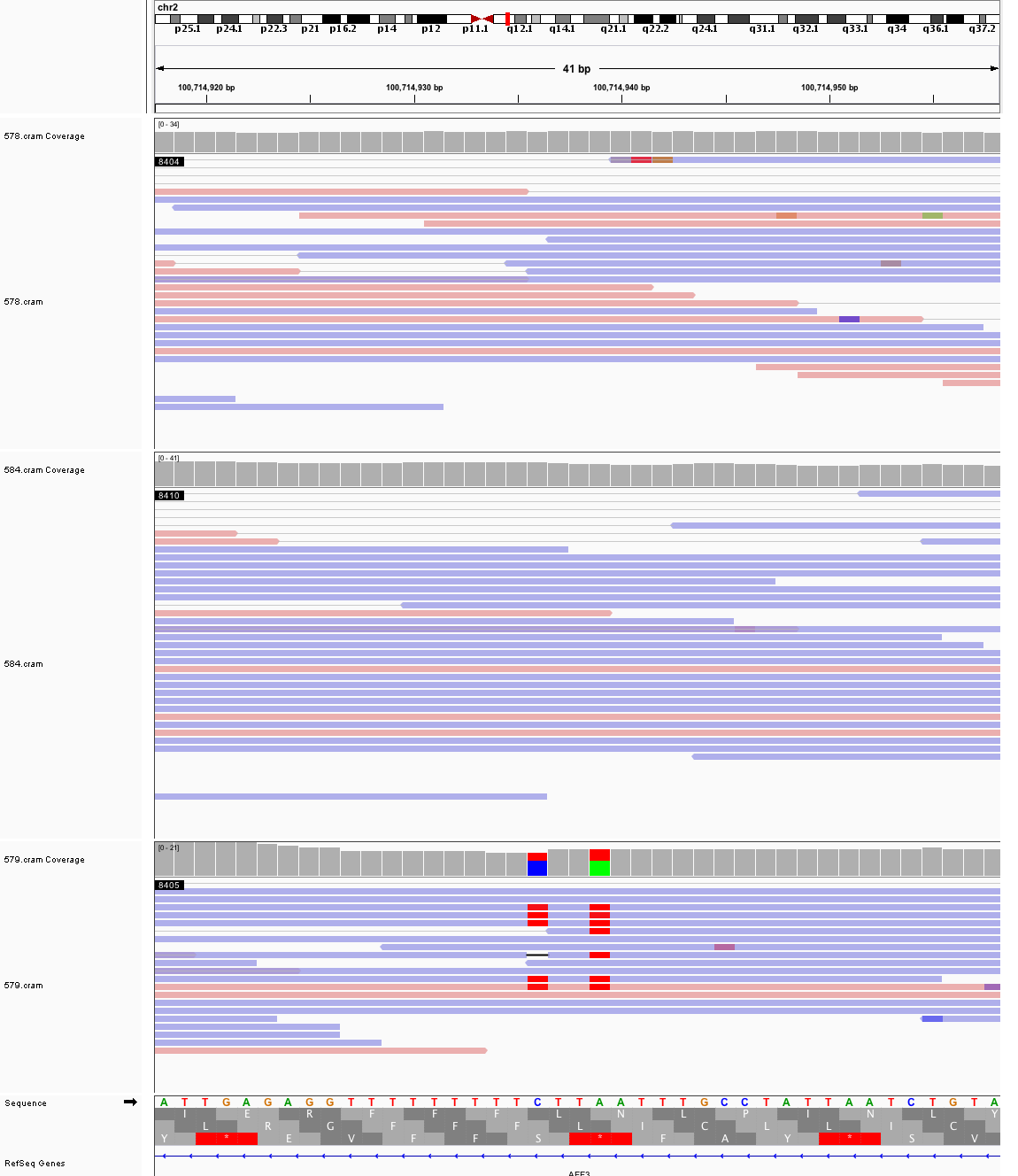

Supplement: Supplementary file 3. — DNMs identified in the third generation In each image, the first two tracks contain alignments from the second-generation parents, and the third track contains the alignments for the third-generation child. Reads with mapping quality <20 are filtered out, as they were not considered by our variant calling pipeline, and mismatched bases are shaded by quality score (more transparent = lower base quality). [file elife-46922-supp3.zip › supp_file_3/chr2_100,714,918_100,714,958.png]

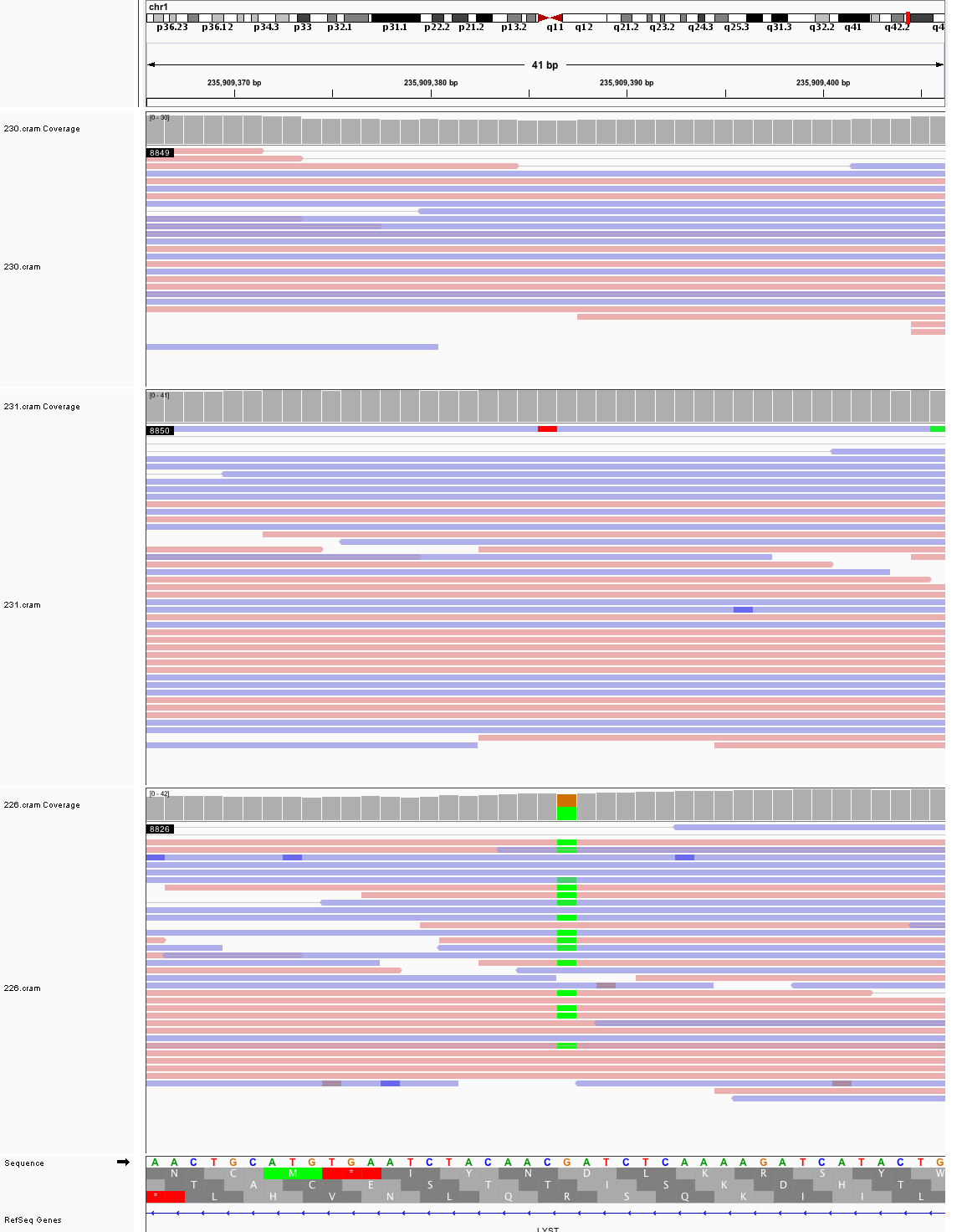

Supplement: Supplementary file 3. — DNMs identified in the third generation In each image, the first two tracks contain alignments from the second-generation parents, and the third track contains the alignments for the third-generation child. Reads with mapping quality <20 are filtered out, as they were not considered by our variant calling pipeline, and mismatched bases are shaded by quality score (more transparent = lower base quality). [file elife-46922-supp3.zip › supp_file_3/chr1_235,909,366_235,909,406.png]

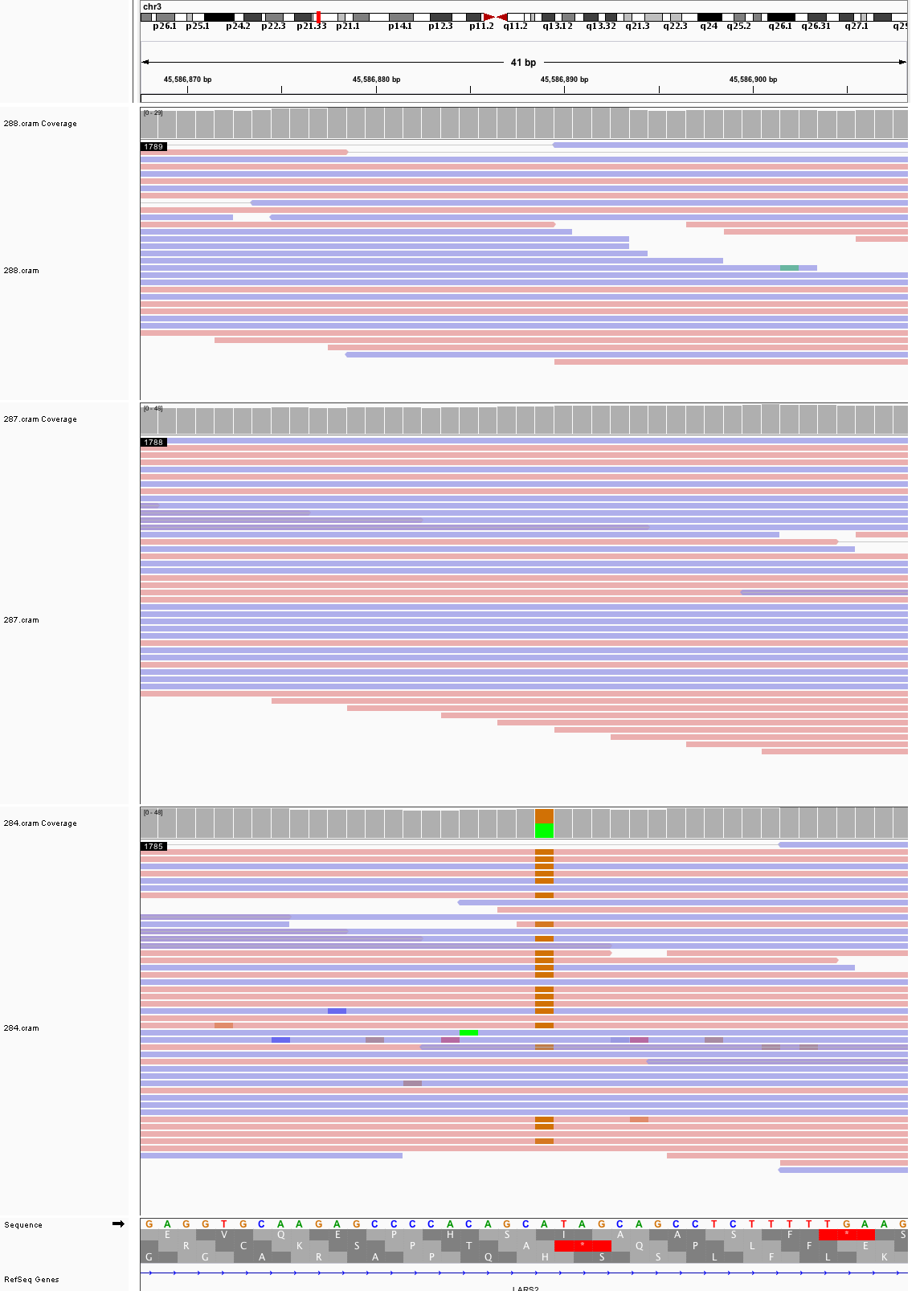

Supplement: Supplementary file 3. — DNMs identified in the third generation In each image, the first two tracks contain alignments from the second-generation parents, and the third track contains the alignments for the third-generation child. Reads with mapping quality <20 are filtered out, as they were not considered by our variant calling pipeline, and mismatched bases are shaded by quality score (more transparent = lower base quality). [file elife-46922-supp3.zip › supp_file_3/chr3_45,586,868_45,586,908.png]

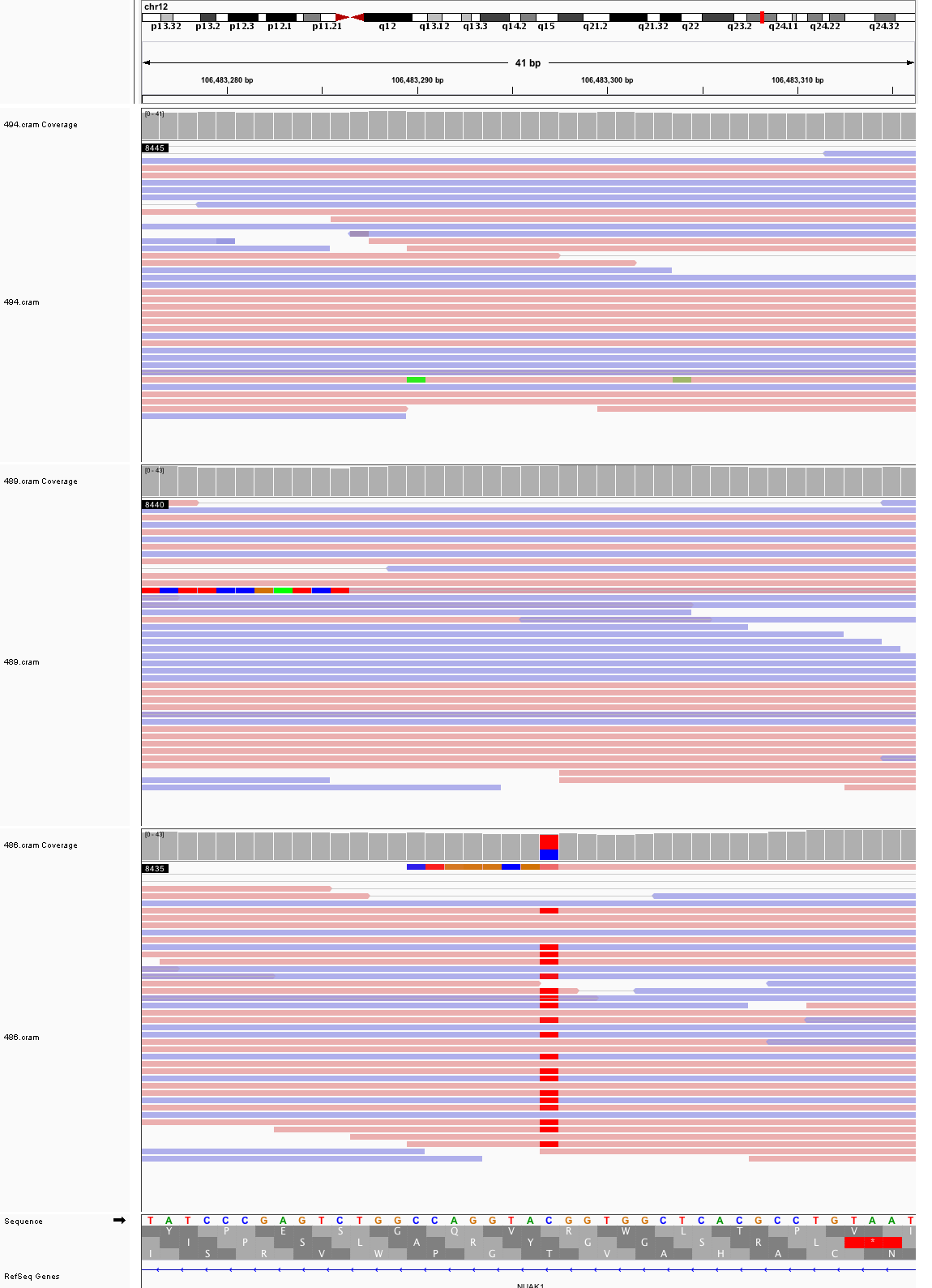

Supplement: Supplementary file 3. — DNMs identified in the third generation In each image, the first two tracks contain alignments from the second-generation parents, and the third track contains the alignments for the third-generation child. Reads with mapping quality <20 are filtered out, as they were not considered by our variant calling pipeline, and mismatched bases are shaded by quality score (more transparent = lower base quality). [file elife-46922-supp3.zip › supp_file_3/chr12_106,483,276_106,483,316.png]

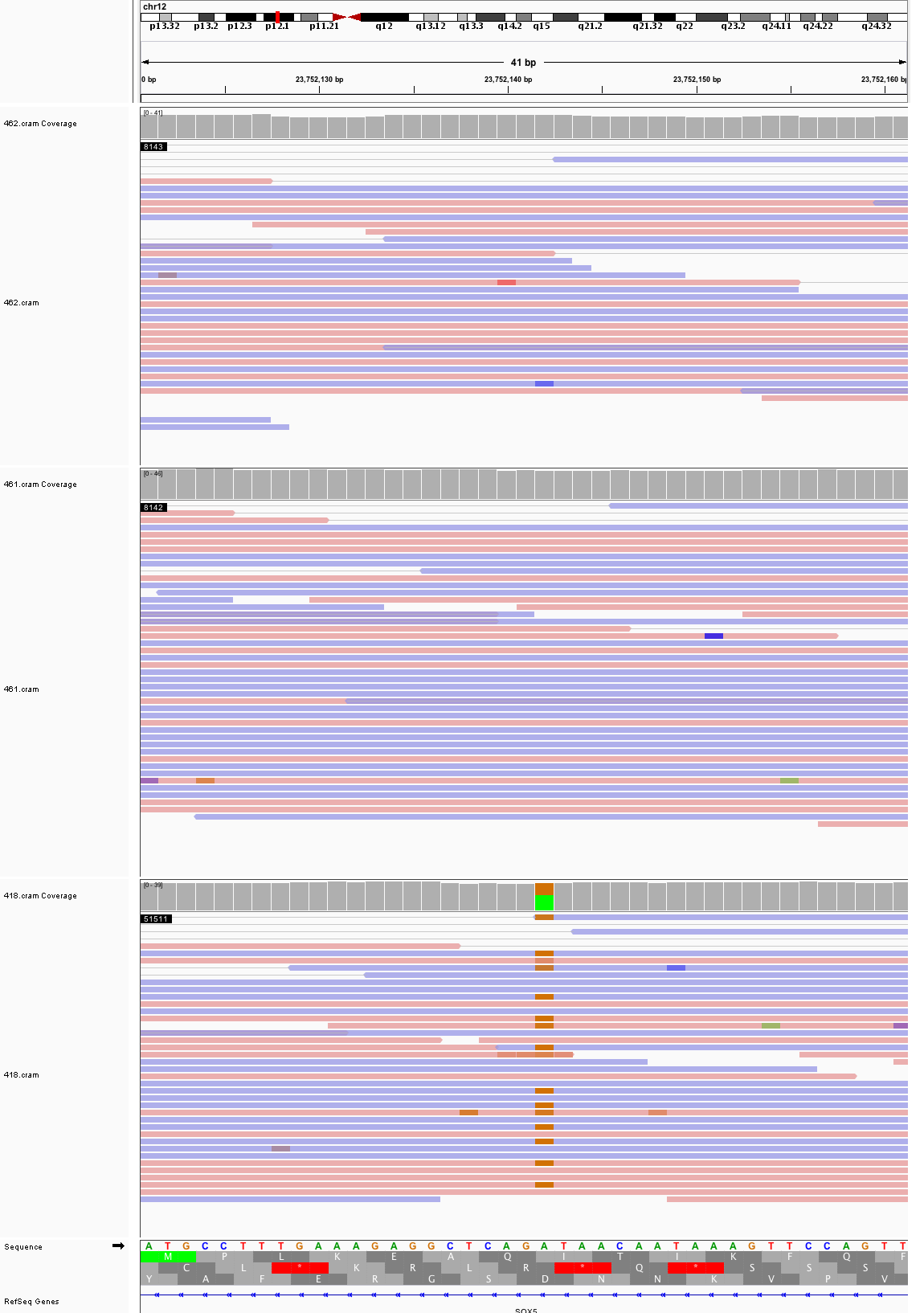

Supplement: Supplementary file 3. — DNMs identified in the third generation In each image, the first two tracks contain alignments from the second-generation parents, and the third track contains the alignments for the third-generation child. Reads with mapping quality <20 are filtered out, as they were not considered by our variant calling pipeline, and mismatched bases are shaded by quality score (more transparent = lower base quality). [file elife-46922-supp3.zip › supp_file_3/chr12_23,752,121_23,752,161.png]

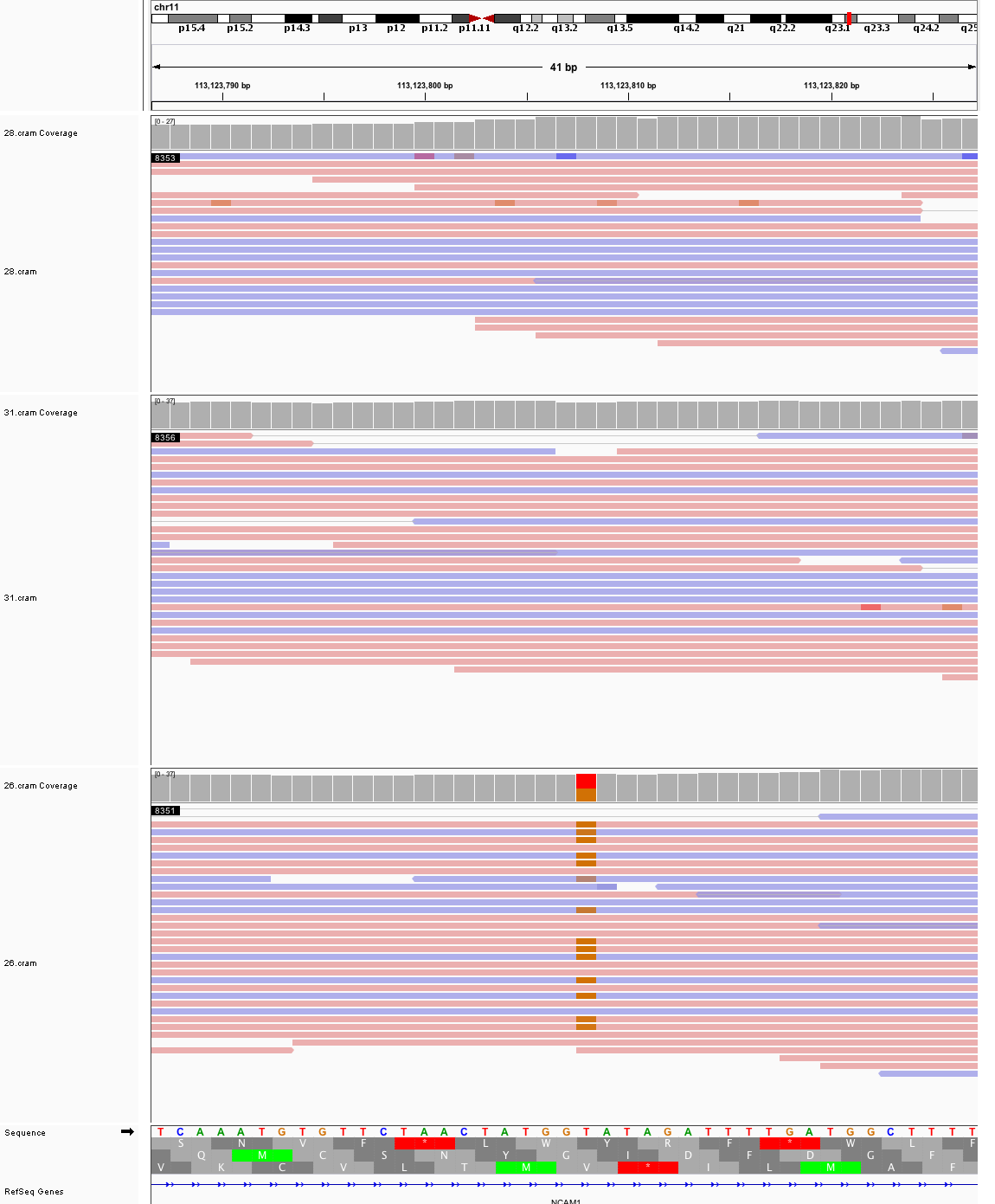

Supplement: Supplementary file 3. — DNMs identified in the third generation In each image, the first two tracks contain alignments from the second-generation parents, and the third track contains the alignments for the third-generation child. Reads with mapping quality <20 are filtered out, as they were not considered by our variant calling pipeline, and mismatched bases are shaded by quality score (more transparent = lower base quality). [file elife-46922-supp3.zip › supp_file_3/chr11_113,123,787_113,123,827.png]

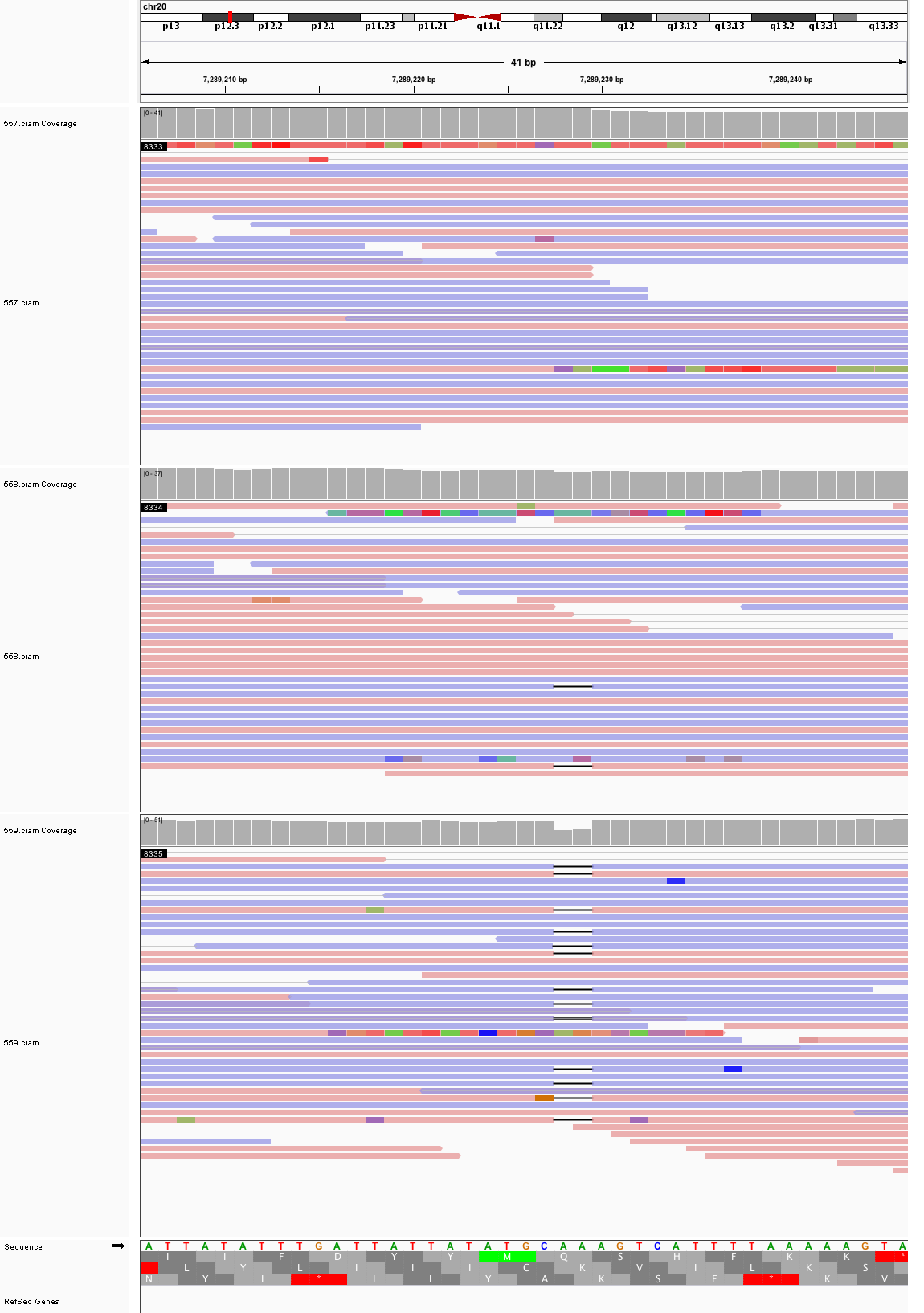

Supplement: Supplementary file 3. — DNMs identified in the third generation In each image, the first two tracks contain alignments from the second-generation parents, and the third track contains the alignments for the third-generation child. Reads with mapping quality <20 are filtered out, as they were not considered by our variant calling pipeline, and mismatched bases are shaded by quality score (more transparent = lower base quality). [file elife-46922-supp3.zip › supp_file_3/chr20_7,289,206_7,289,246.png]

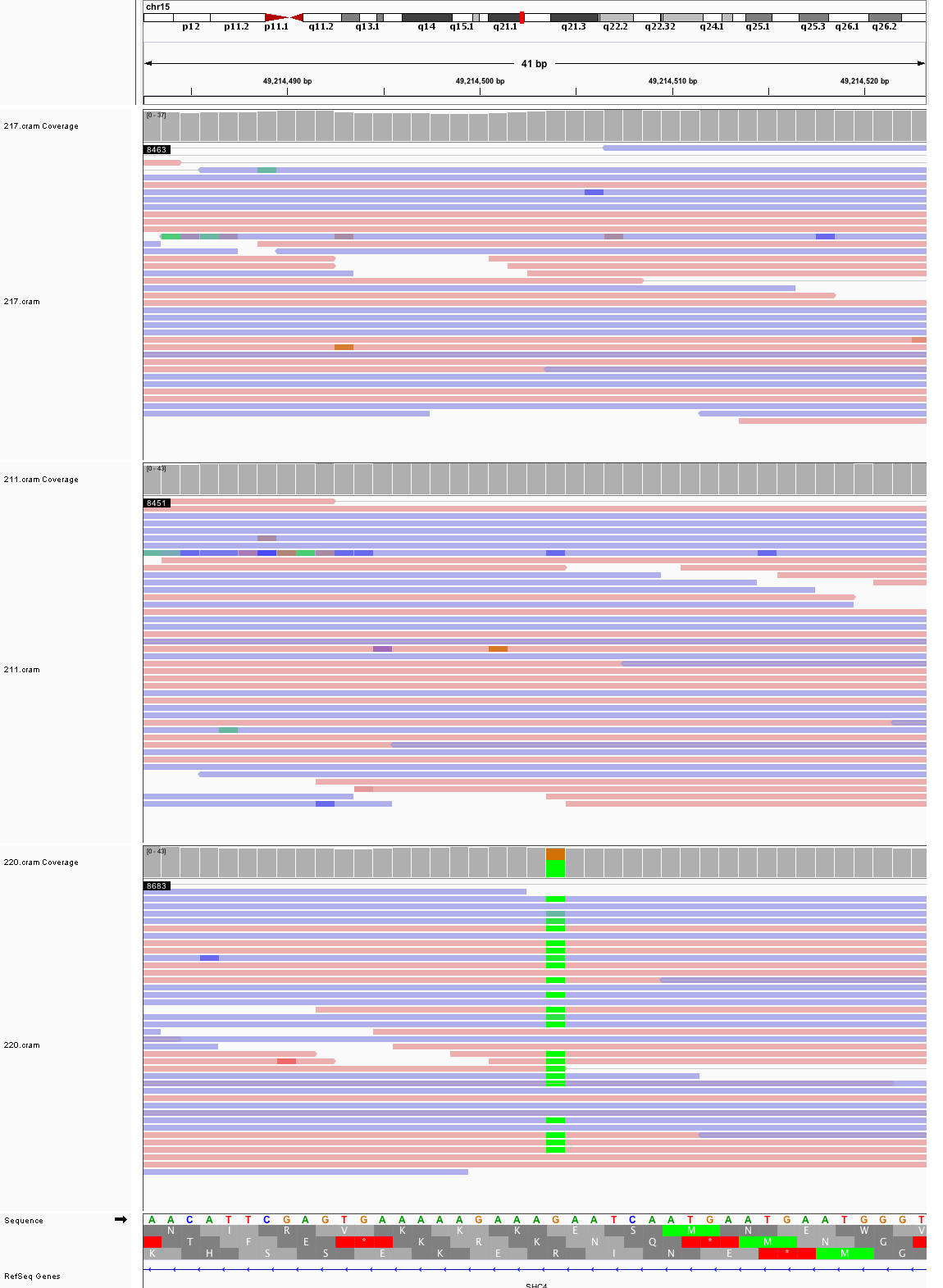

Supplement: Supplementary file 3. — DNMs identified in the third generation In each image, the first two tracks contain alignments from the second-generation parents, and the third track contains the alignments for the third-generation child. Reads with mapping quality <20 are filtered out, as they were not considered by our variant calling pipeline, and mismatched bases are shaded by quality score (more transparent = lower base quality). [file elife-46922-supp3.zip › supp_file_3/chr15_49,214,483_49,214,523.png]

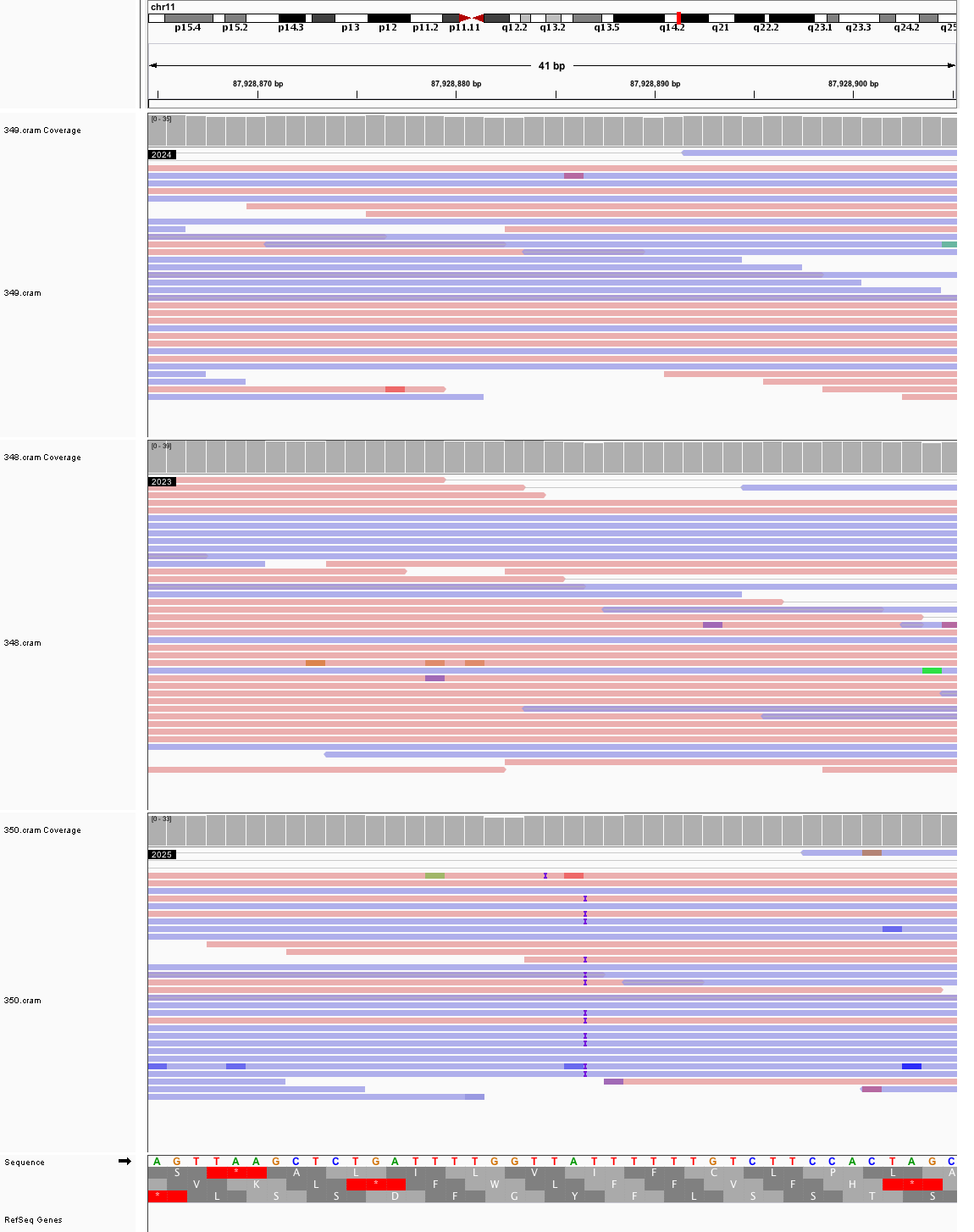

Supplement: Supplementary file 3. — DNMs identified in the third generation In each image, the first two tracks contain alignments from the second-generation parents, and the third track contains the alignments for the third-generation child. Reads with mapping quality <20 are filtered out, as they were not considered by our variant calling pipeline, and mismatched bases are shaded by quality score (more transparent = lower base quality). [file elife-46922-supp3.zip › supp_file_3/chr11_87,928,865_87,928,905.png]

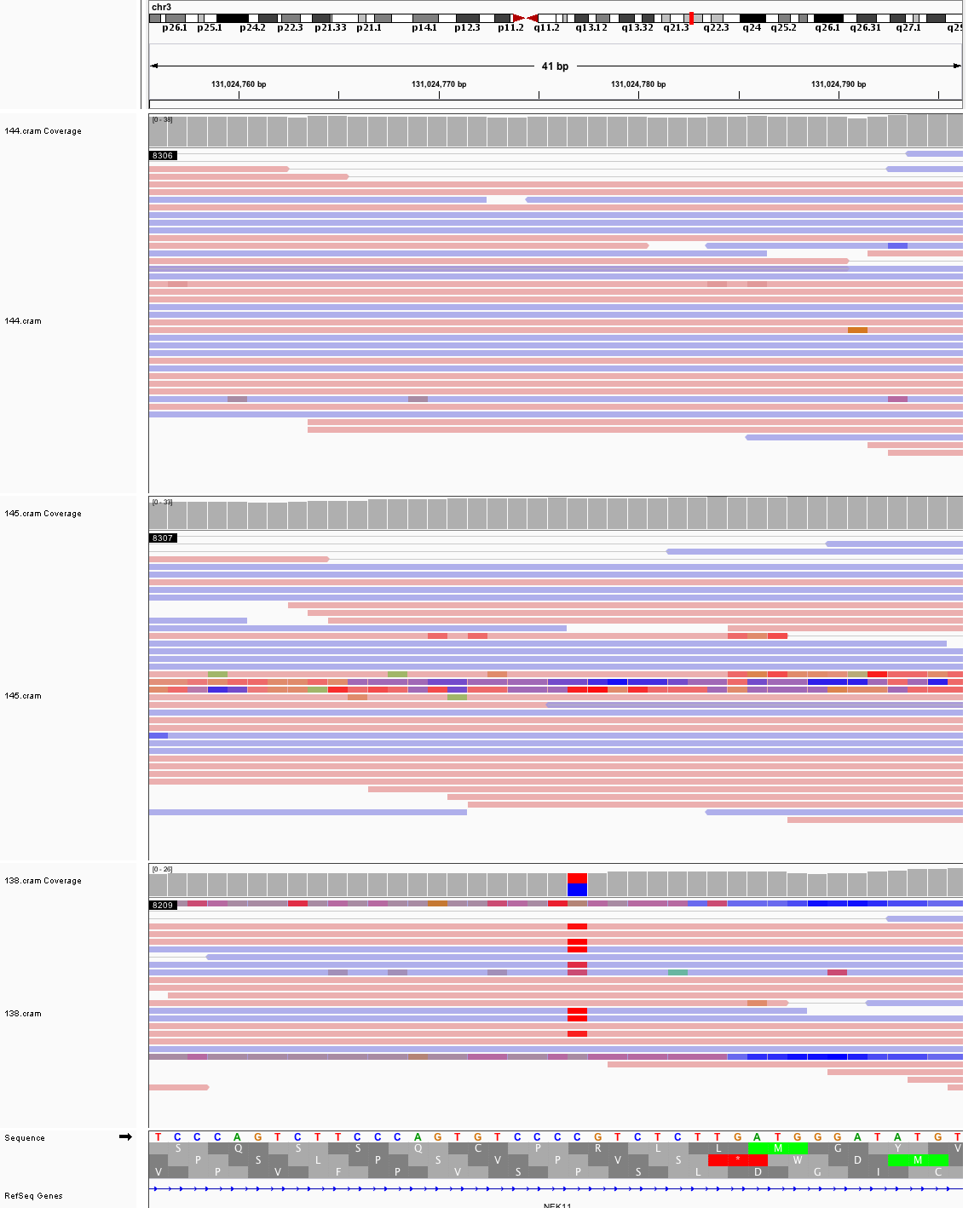

Supplement: Supplementary file 3. — DNMs identified in the third generation In each image, the first two tracks contain alignments from the second-generation parents, and the third track contains the alignments for the third-generation child. Reads with mapping quality <20 are filtered out, as they were not considered by our variant calling pipeline, and mismatched bases are shaded by quality score (more transparent = lower base quality). [file elife-46922-supp3.zip › supp_file_3/chr3_131,024,756_131,024,796.png]

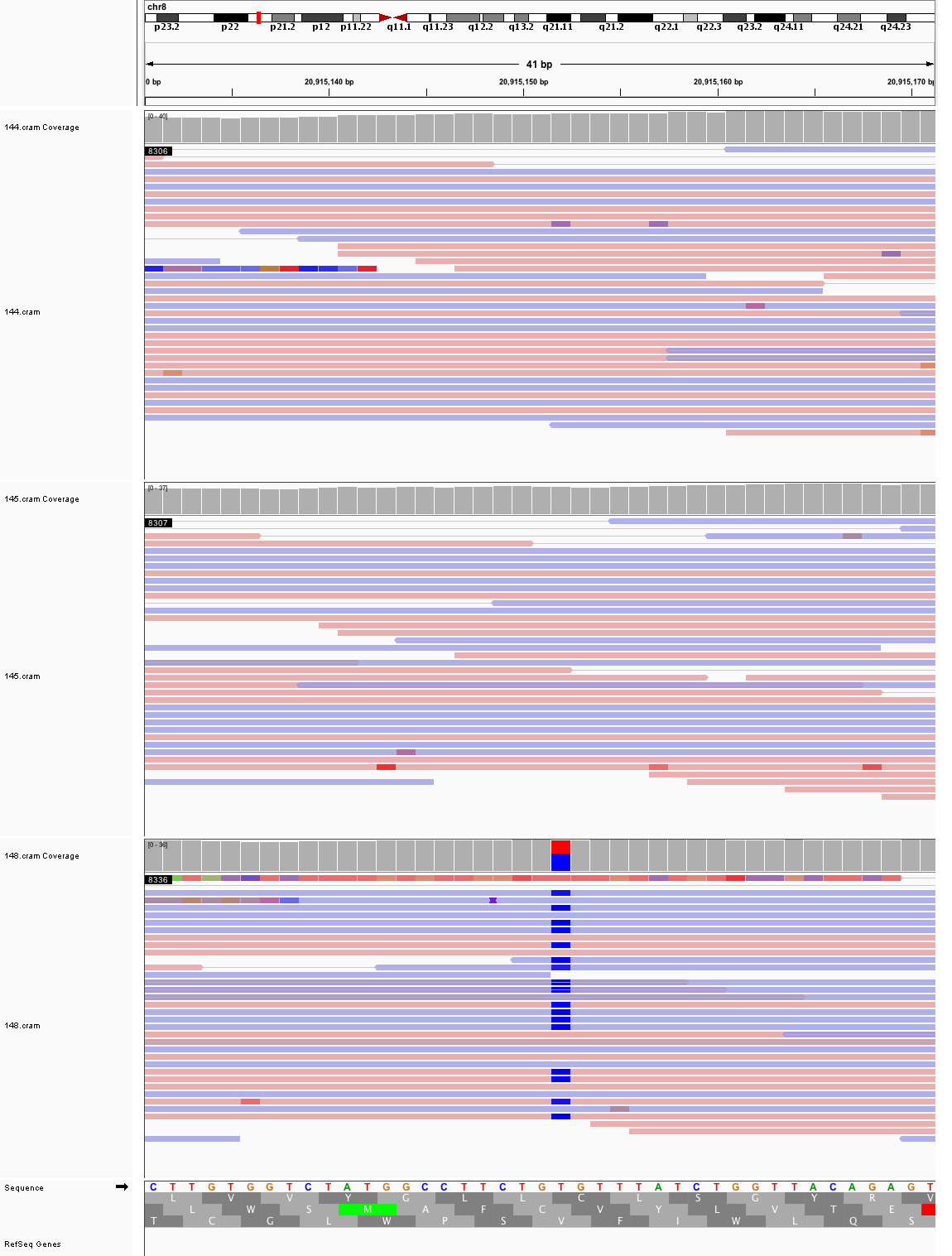

Supplement: Supplementary file 3. — DNMs identified in the third generation In each image, the first two tracks contain alignments from the second-generation parents, and the third track contains the alignments for the third-generation child. Reads with mapping quality <20 are filtered out, as they were not considered by our variant calling pipeline, and mismatched bases are shaded by quality score (more transparent = lower base quality). [file elife-46922-supp3.zip › supp_file_3/chr8_20,915,131_20,915,171.png]

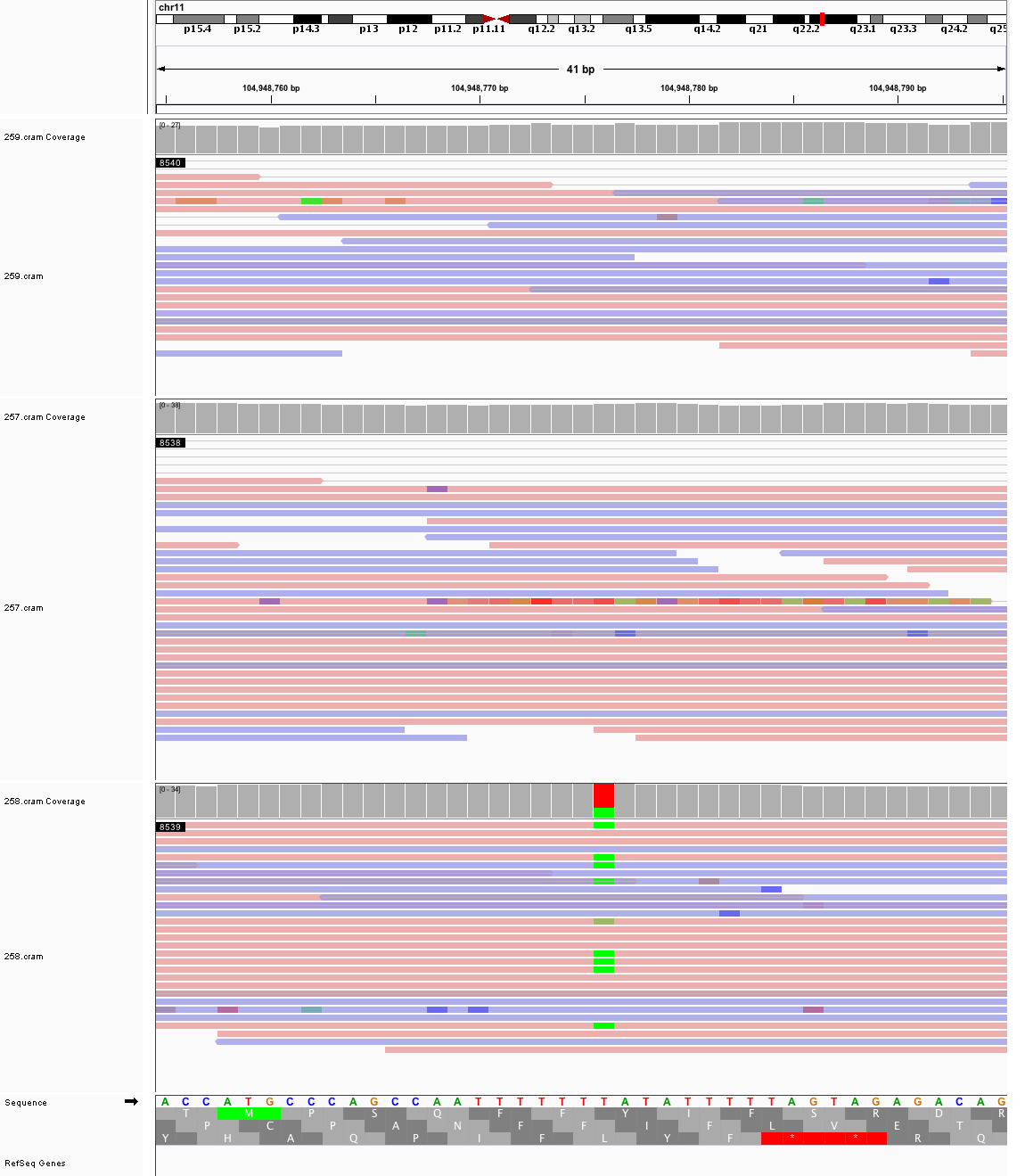

Supplement: Supplementary file 3. — DNMs identified in the third generation In each image, the first two tracks contain alignments from the second-generation parents, and the third track contains the alignments for the third-generation child. Reads with mapping quality <20 are filtered out, as they were not considered by our variant calling pipeline, and mismatched bases are shaded by quality score (more transparent = lower base quality). [file elife-46922-supp3.zip › supp_file_3/chr11_104,948,755_104,948,795.png]

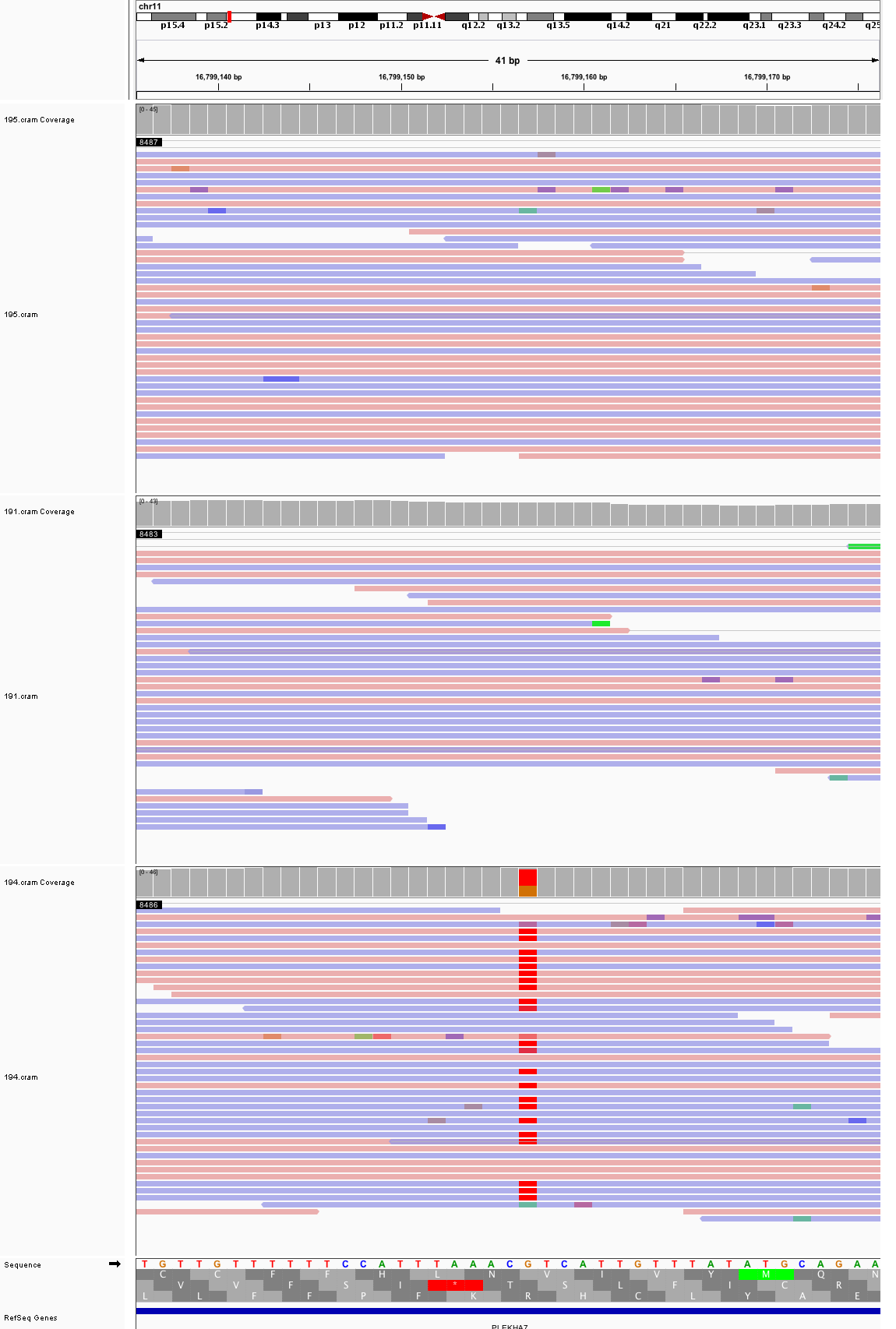

Supplement: Supplementary file 3. — DNMs identified in the third generation In each image, the first two tracks contain alignments from the second-generation parents, and the third track contains the alignments for the third-generation child. Reads with mapping quality <20 are filtered out, as they were not considered by our variant calling pipeline, and mismatched bases are shaded by quality score (more transparent = lower base quality). [file elife-46922-supp3.zip › supp_file_3/chr11_16,799,136_16,799,176.png]

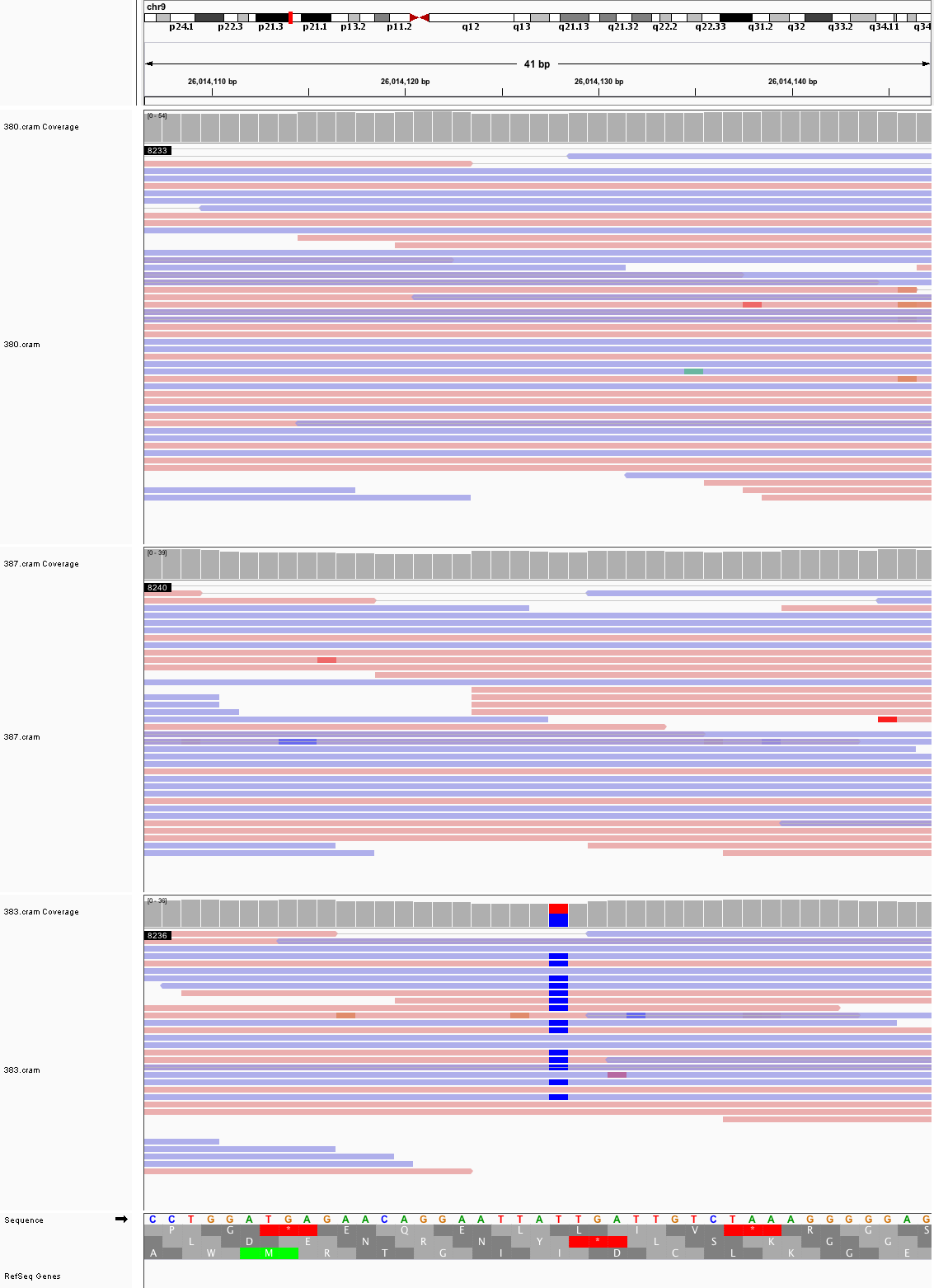

Supplement: Supplementary file 3. — DNMs identified in the third generation In each image, the first two tracks contain alignments from the second-generation parents, and the third track contains the alignments for the third-generation child. Reads with mapping quality <20 are filtered out, as they were not considered by our variant calling pipeline, and mismatched bases are shaded by quality score (more transparent = lower base quality). [file elife-46922-supp3.zip › supp_file_3/chr9_26,014,107_26,014,147.png]

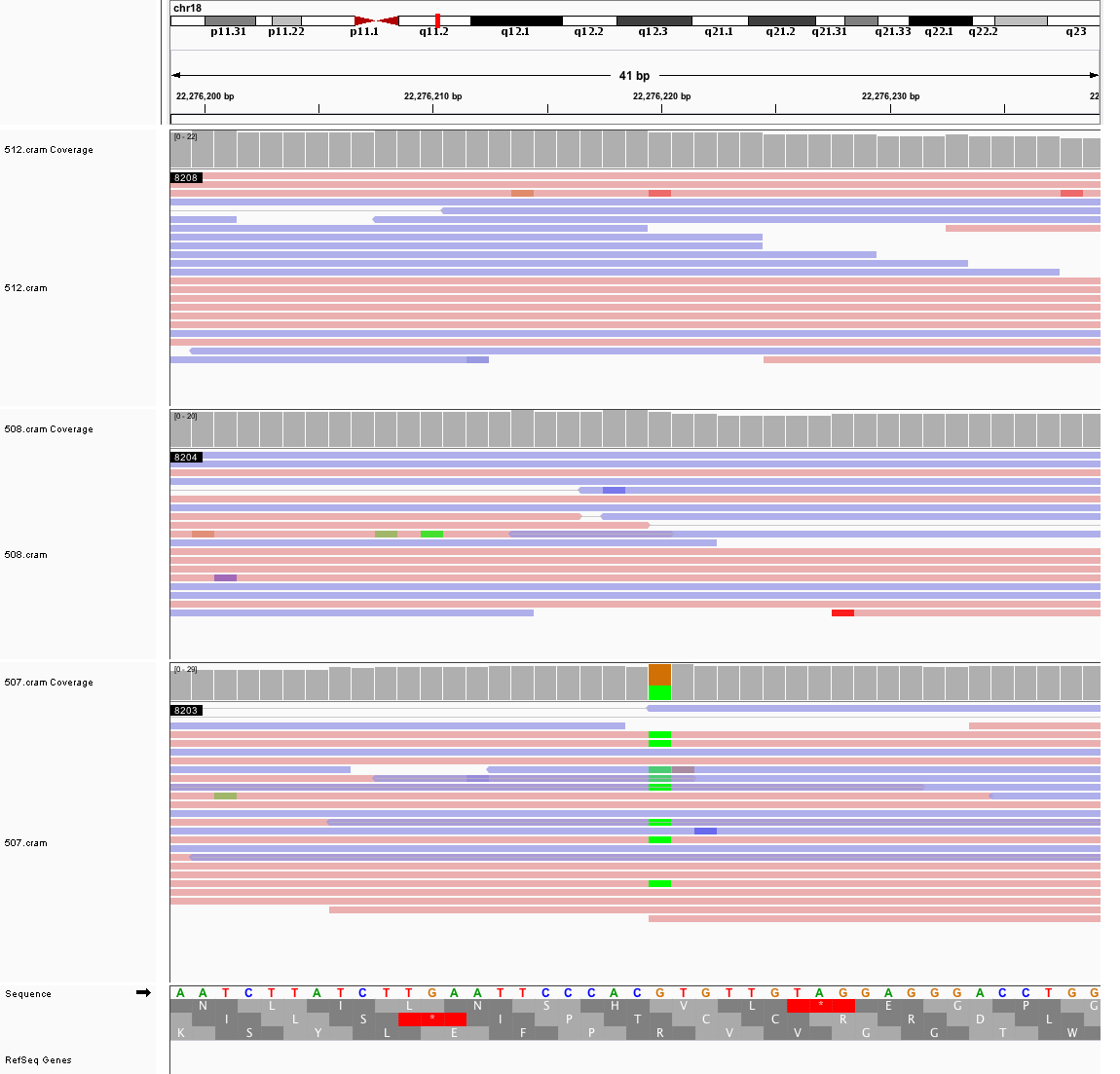

Supplement: Supplementary file 3. — DNMs identified in the third generation In each image, the first two tracks contain alignments from the second-generation parents, and the third track contains the alignments for the third-generation child. Reads with mapping quality <20 are filtered out, as they were not considered by our variant calling pipeline, and mismatched bases are shaded by quality score (more transparent = lower base quality). [file elife-46922-supp3.zip › supp_file_3/chr18_22,276,199_22,276,239.png]

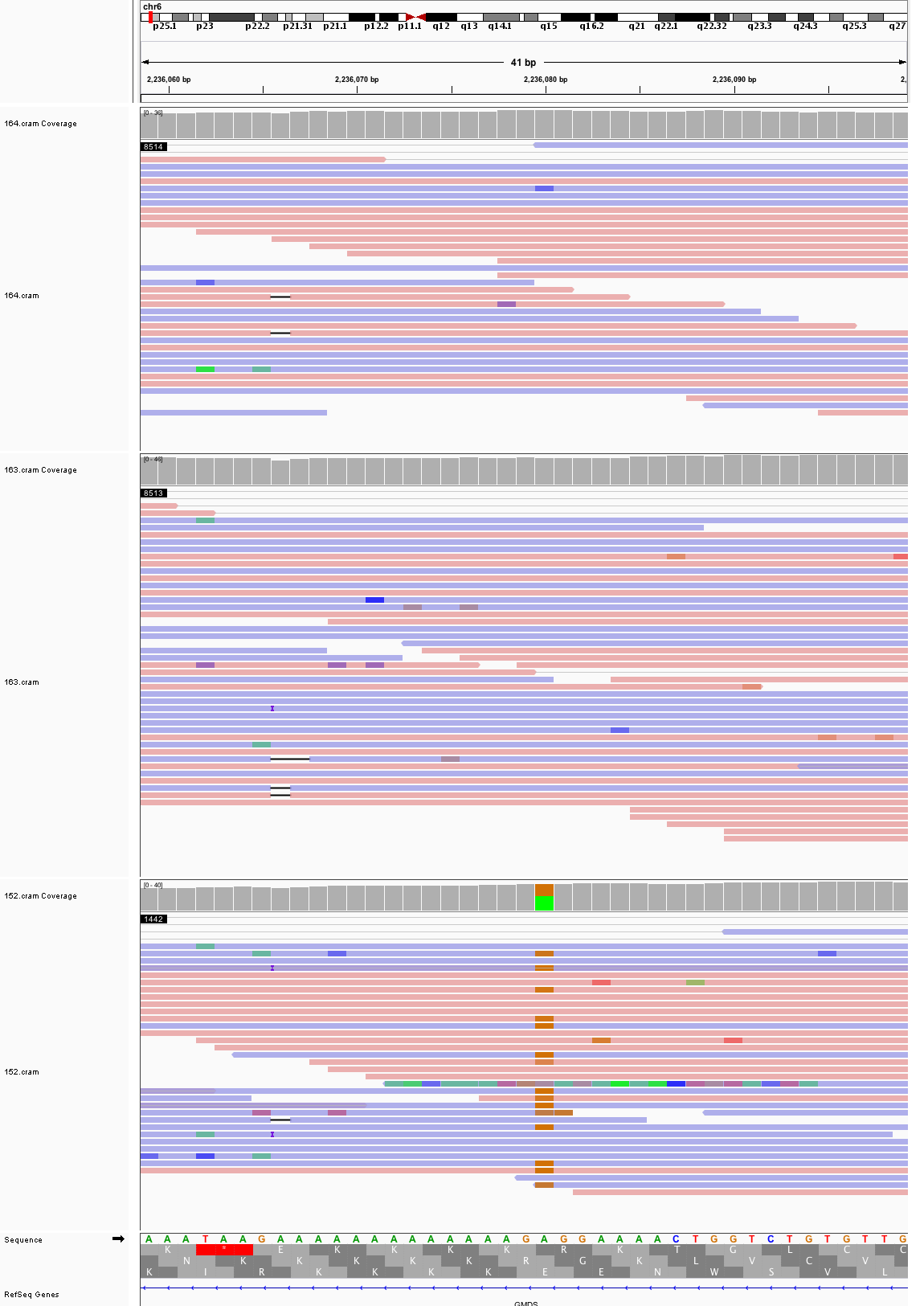

Supplement: Supplementary file 3. — DNMs identified in the third generation In each image, the first two tracks contain alignments from the second-generation parents, and the third track contains the alignments for the third-generation child. Reads with mapping quality <20 are filtered out, as they were not considered by our variant calling pipeline, and mismatched bases are shaded by quality score (more transparent = lower base quality). [file elife-46922-supp3.zip › supp_file_3/chr6_2,236,059_2,236,099.png]

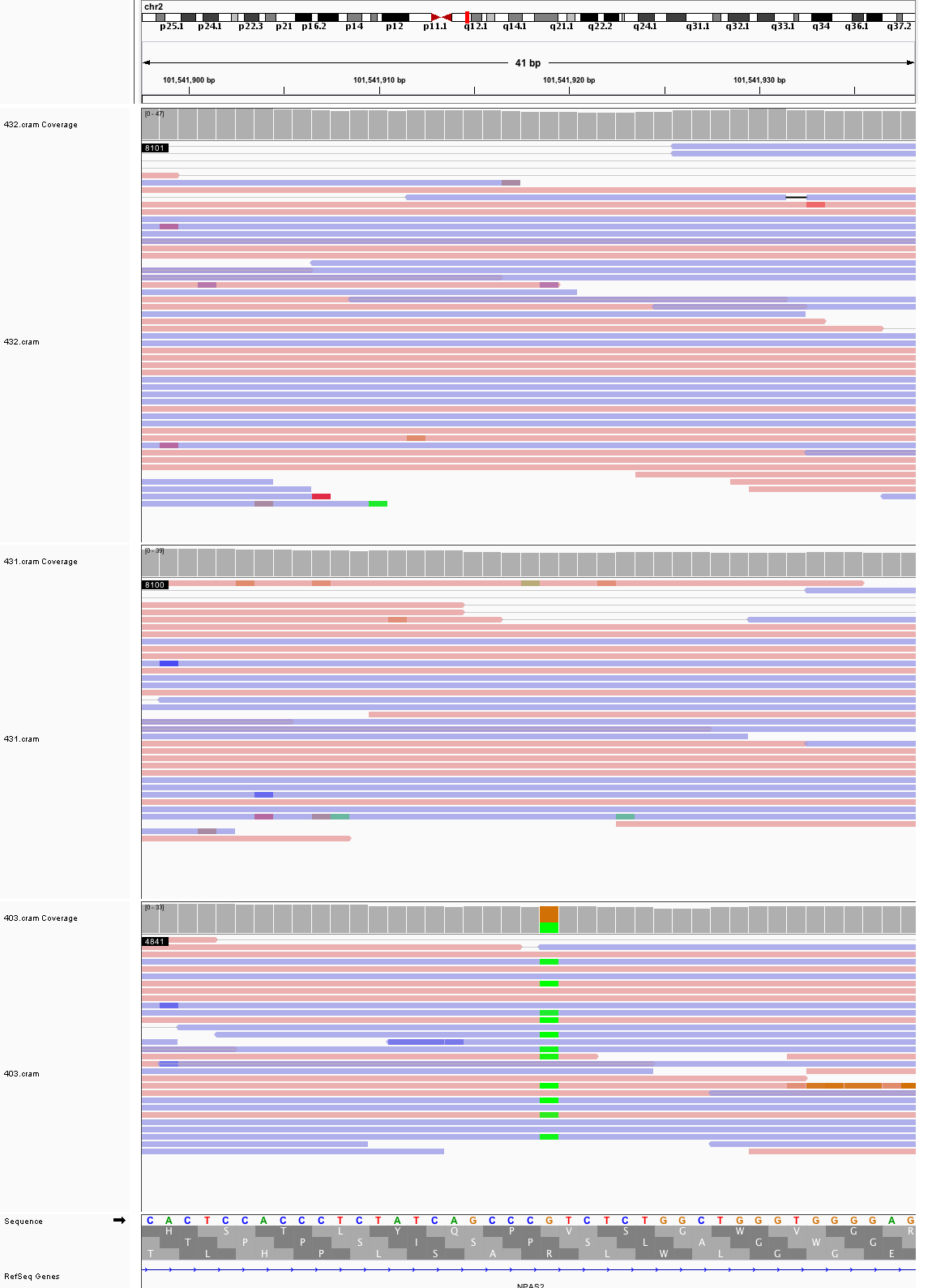

Supplement: Supplementary file 3. — DNMs identified in the third generation In each image, the first two tracks contain alignments from the second-generation parents, and the third track contains the alignments for the third-generation child. Reads with mapping quality <20 are filtered out, as they were not considered by our variant calling pipeline, and mismatched bases are shaded by quality score (more transparent = lower base quality). [file elife-46922-supp3.zip › supp_file_3/chr2_101,541,898_101,541,938.png]

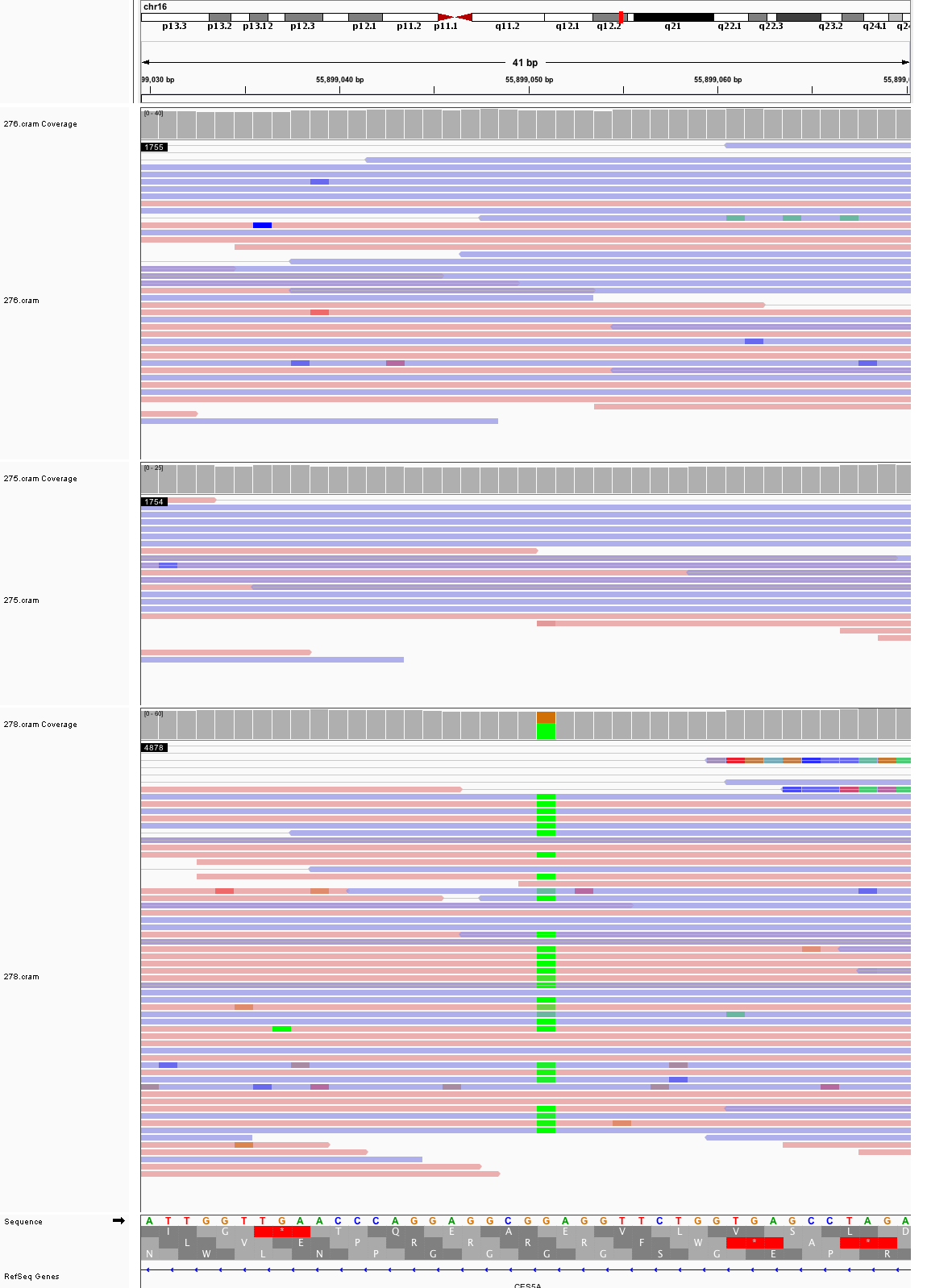

Supplement: Supplementary file 3. — DNMs identified in the third generation In each image, the first two tracks contain alignments from the second-generation parents, and the third track contains the alignments for the third-generation child. Reads with mapping quality <20 are filtered out, as they were not considered by our variant calling pipeline, and mismatched bases are shaded by quality score (more transparent = lower base quality). [file elife-46922-supp3.zip › supp_file_3/chr16_55,899,030_55,899,070.png]

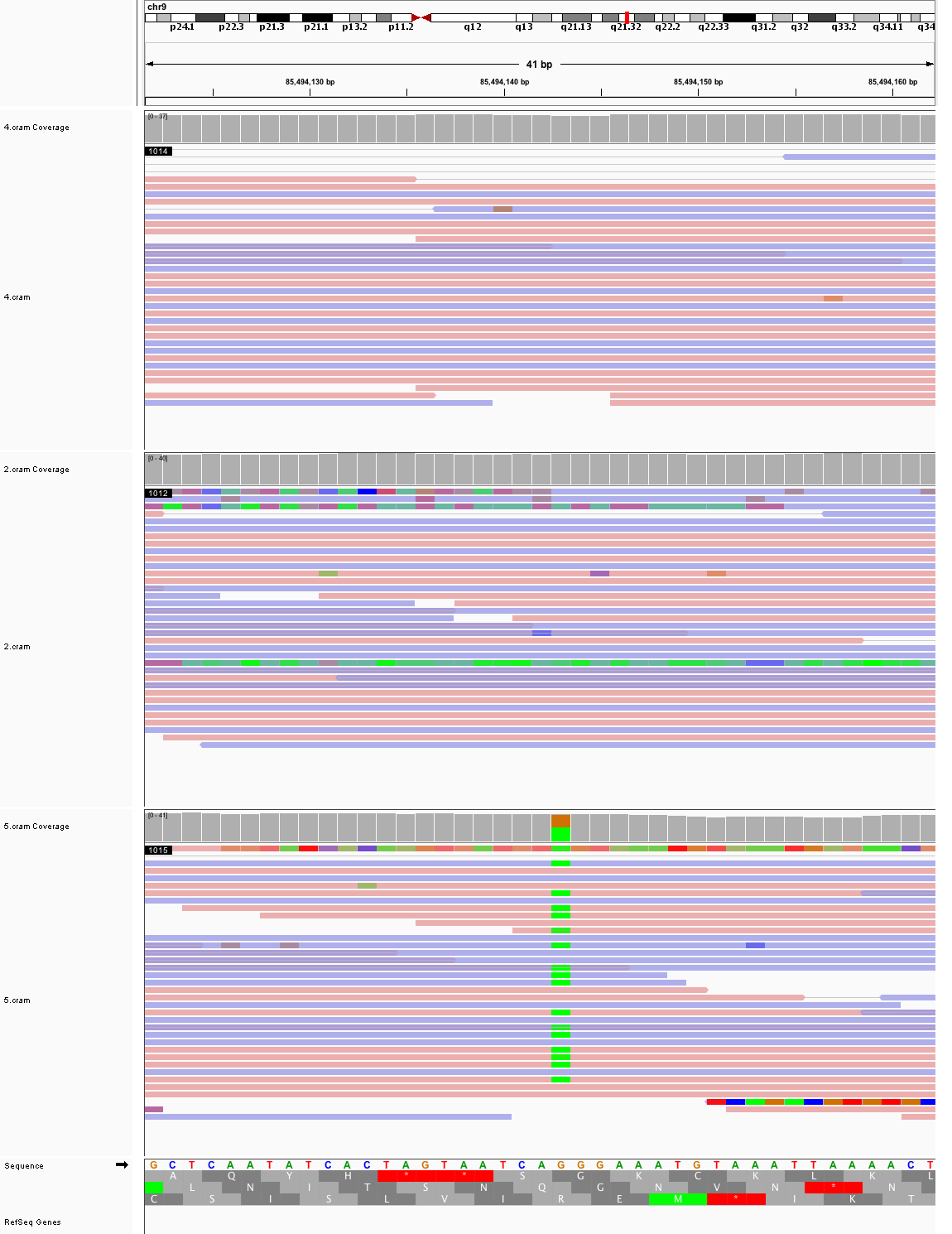

Supplement: Supplementary file 3. — DNMs identified in the third generation In each image, the first two tracks contain alignments from the second-generation parents, and the third track contains the alignments for the third-generation child. Reads with mapping quality <20 are filtered out, as they were not considered by our variant calling pipeline, and mismatched bases are shaded by quality score (more transparent = lower base quality). [file elife-46922-supp3.zip › supp_file_3/chr9_85,494,122_85,494,162.png]

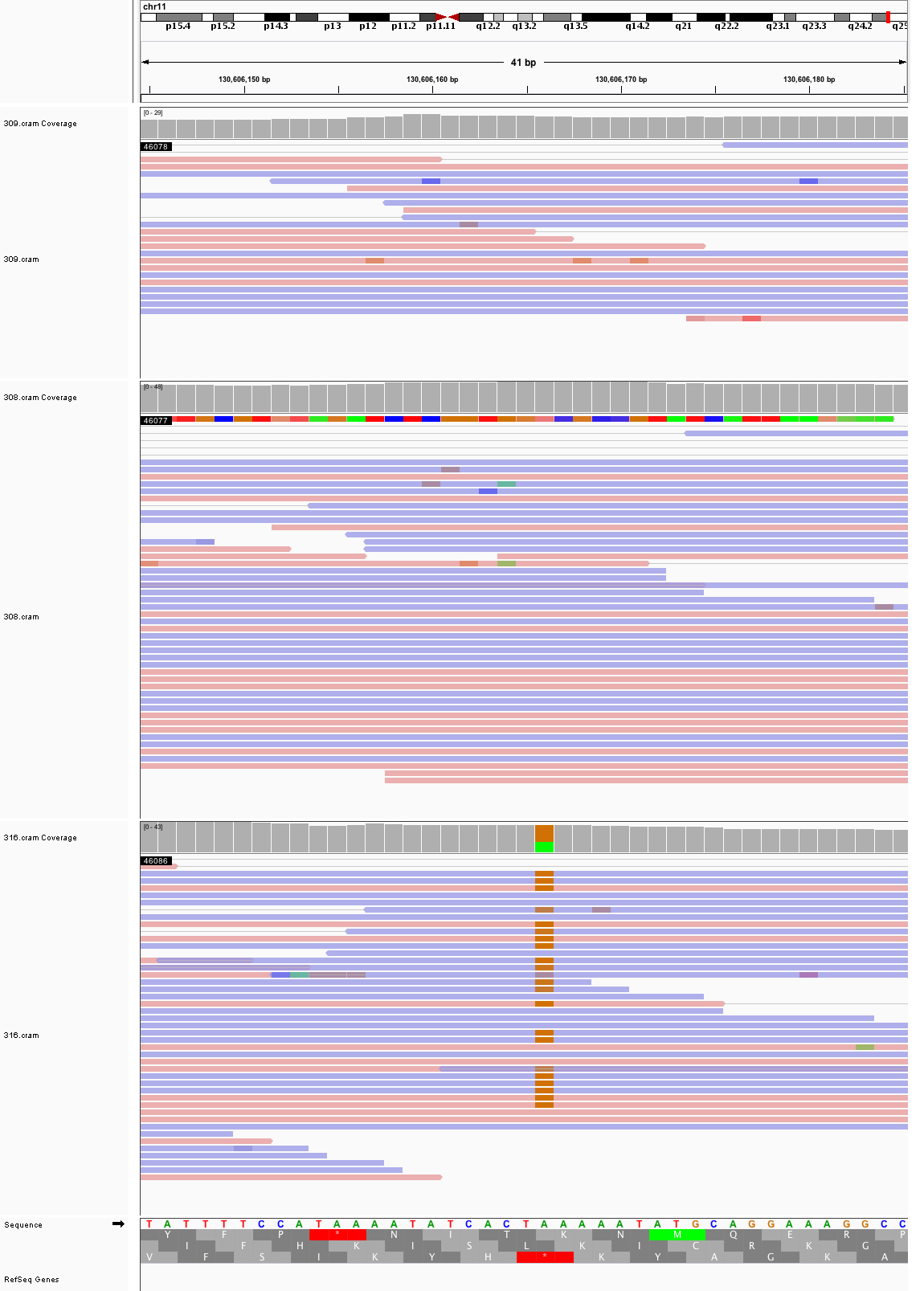

Supplement: Supplementary file 3. — DNMs identified in the third generation In each image, the first two tracks contain alignments from the second-generation parents, and the third track contains the alignments for the third-generation child. Reads with mapping quality <20 are filtered out, as they were not considered by our variant calling pipeline, and mismatched bases are shaded by quality score (more transparent = lower base quality). [file elife-46922-supp3.zip › supp_file_3/chr11_130,606,145_130,606,185.png]

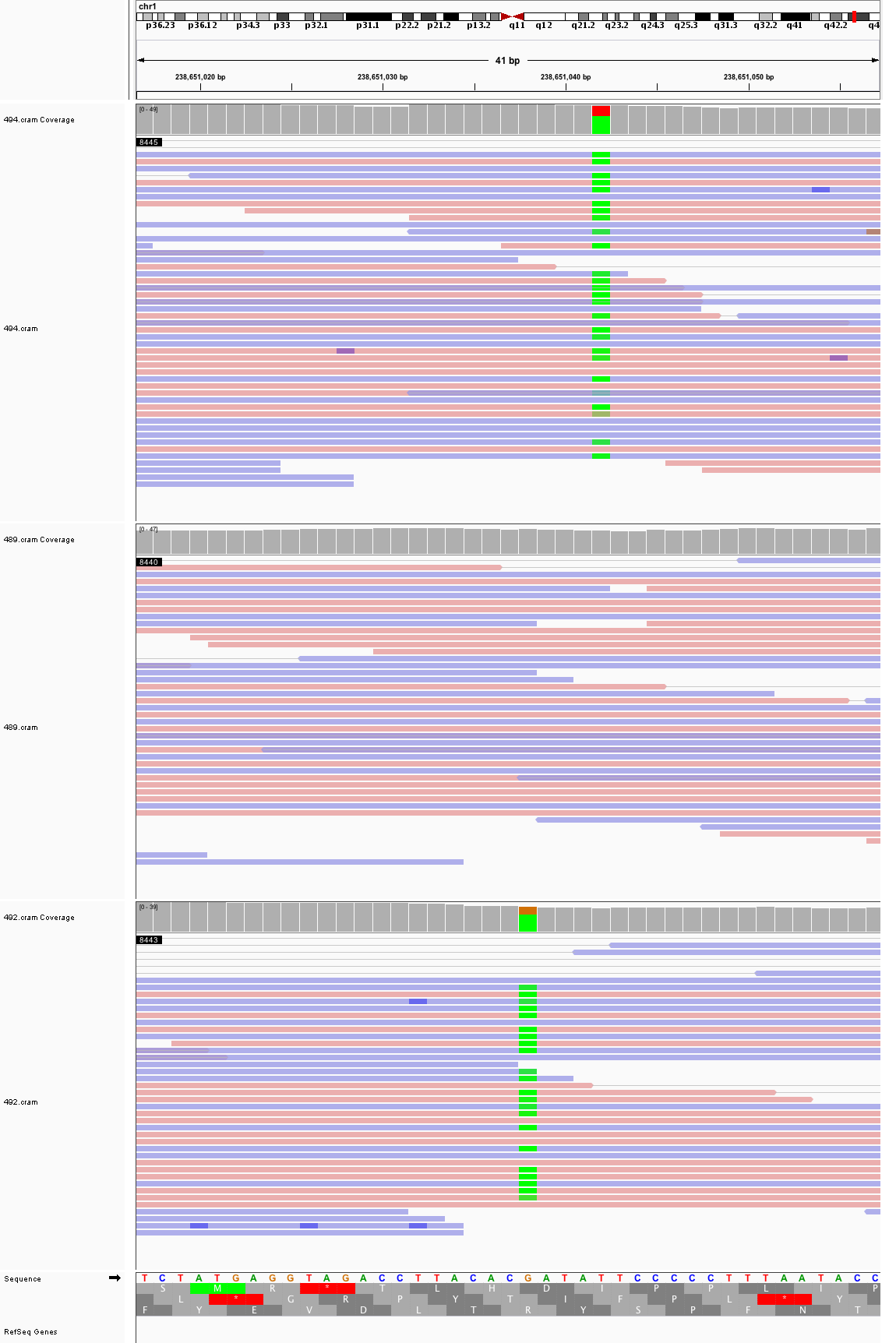

Supplement: Supplementary file 3. — DNMs identified in the third generation In each image, the first two tracks contain alignments from the second-generation parents, and the third track contains the alignments for the third-generation child. Reads with mapping quality <20 are filtered out, as they were not considered by our variant calling pipeline, and mismatched bases are shaded by quality score (more transparent = lower base quality). [file elife-46922-supp3.zip › supp_file_3/chr1_238,651,017_238,651,057.png]

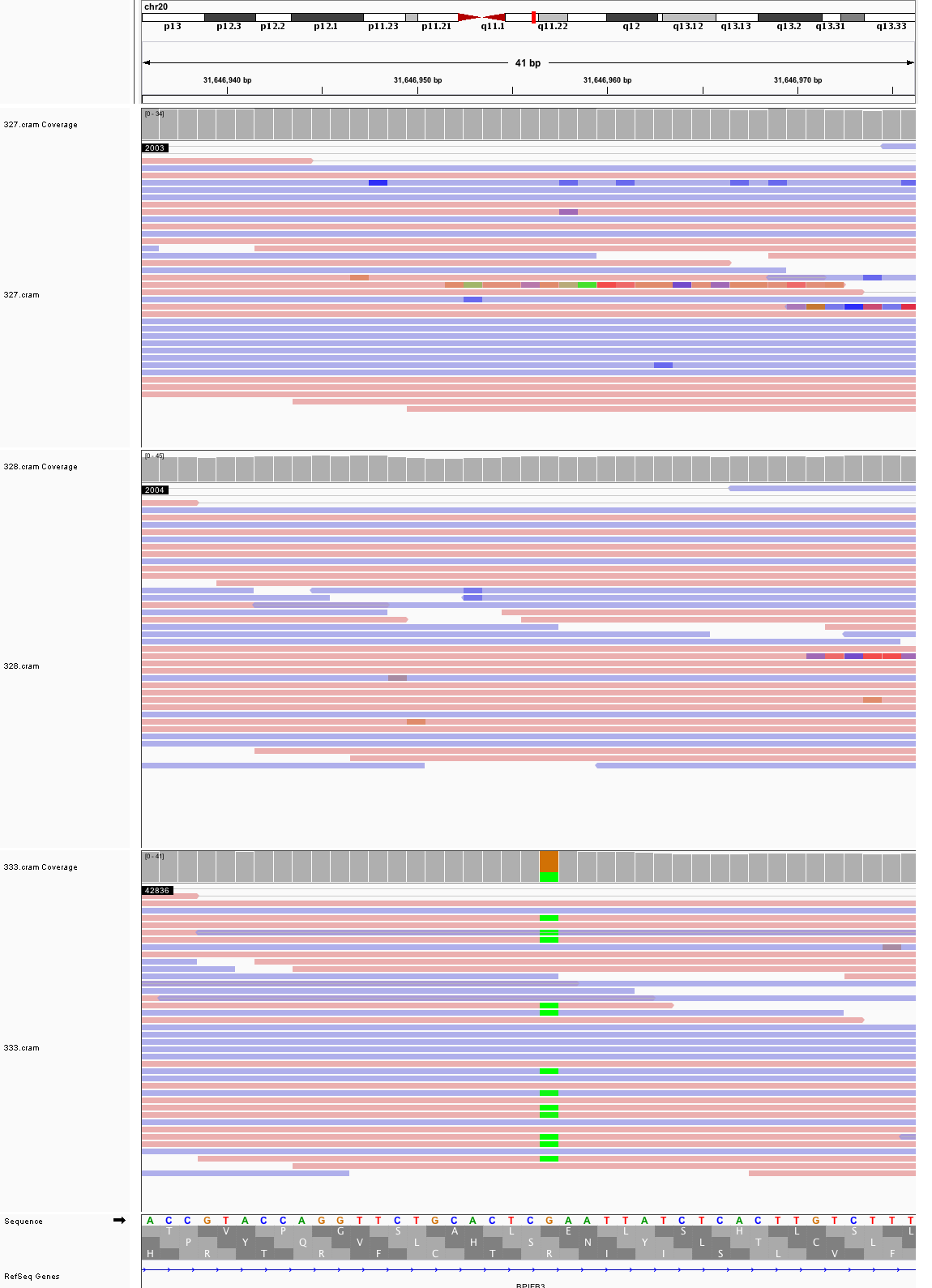

Supplement: Supplementary file 3. — DNMs identified in the third generation In each image, the first two tracks contain alignments from the second-generation parents, and the third track contains the alignments for the third-generation child. Reads with mapping quality <20 are filtered out, as they were not considered by our variant calling pipeline, and mismatched bases are shaded by quality score (more transparent = lower base quality). [file elife-46922-supp3.zip › supp_file_3/chr20_31,646,936_31,646,976.png]

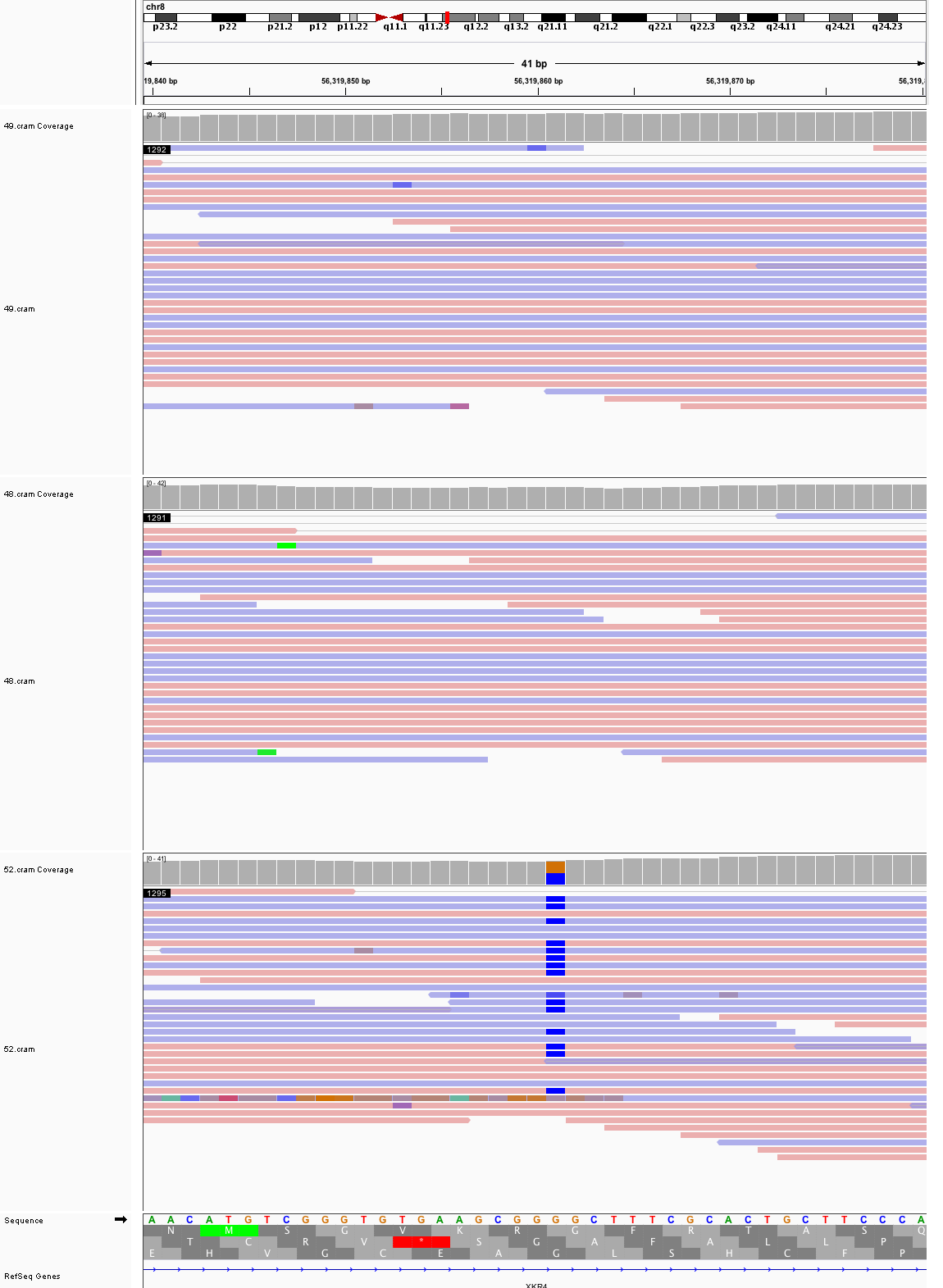

Supplement: Supplementary file 3. — DNMs identified in the third generation In each image, the first two tracks contain alignments from the second-generation parents, and the third track contains the alignments for the third-generation child. Reads with mapping quality <20 are filtered out, as they were not considered by our variant calling pipeline, and mismatched bases are shaded by quality score (more transparent = lower base quality). [file elife-46922-supp3.zip › supp_file_3/chr8_56,319,840_56,319,880.png]

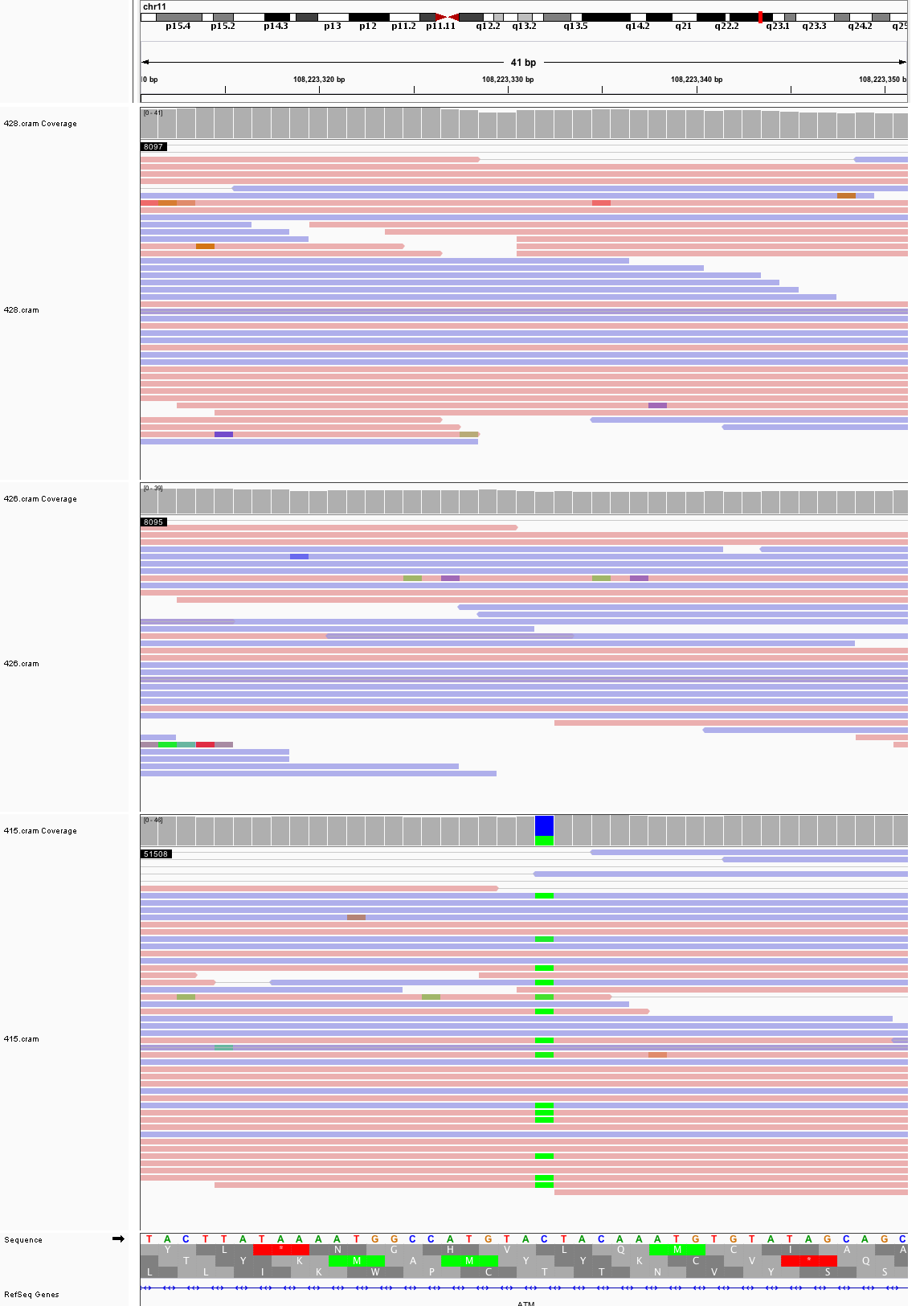

Supplement: Supplementary file 3. — DNMs identified in the third generation In each image, the first two tracks contain alignments from the second-generation parents, and the third track contains the alignments for the third-generation child. Reads with mapping quality <20 are filtered out, as they were not considered by our variant calling pipeline, and mismatched bases are shaded by quality score (more transparent = lower base quality). [file elife-46922-supp3.zip › supp_file_3/chr11_108,223,311_108,223,351.png]

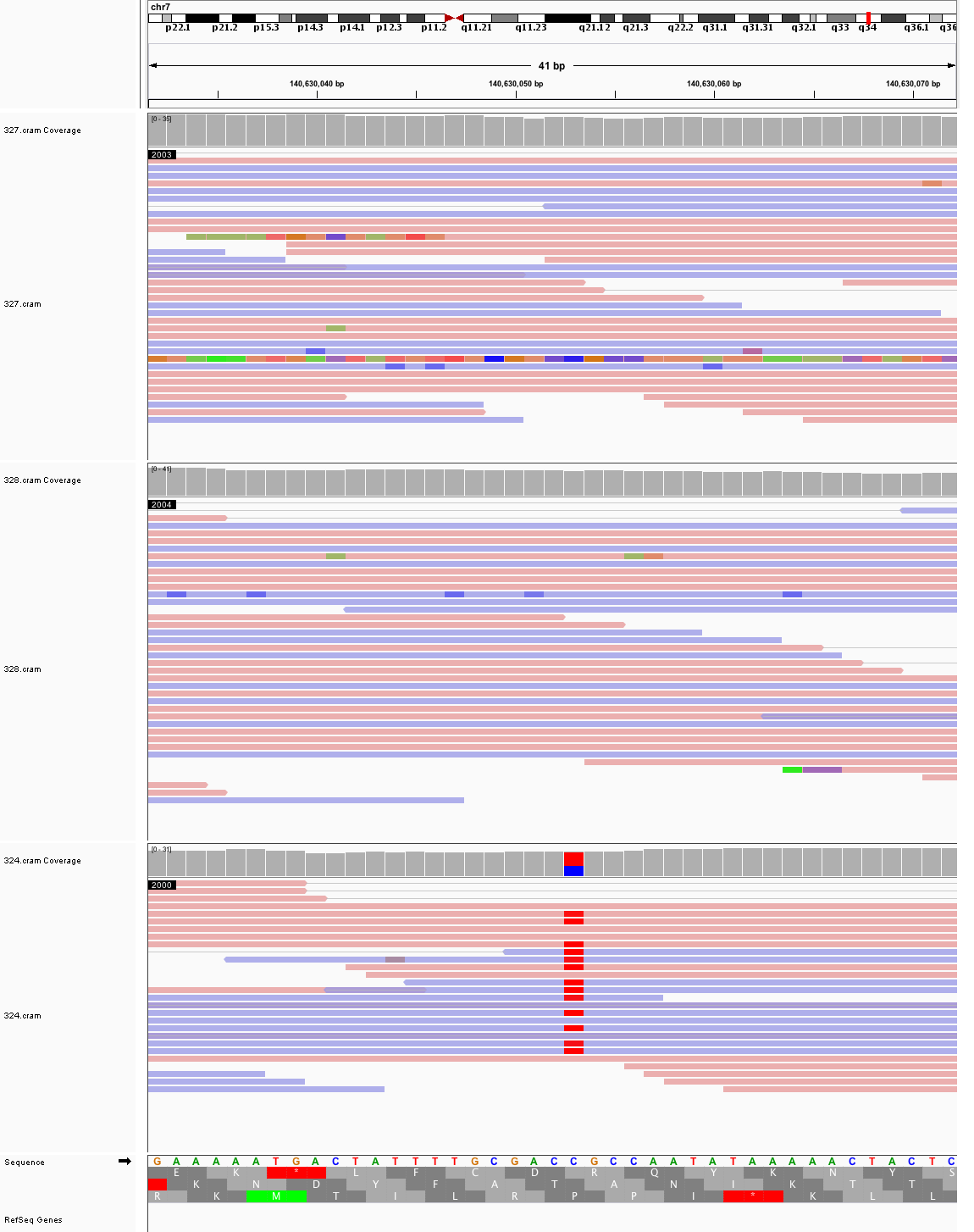

Supplement: Supplementary file 3. — DNMs identified in the third generation In each image, the first two tracks contain alignments from the second-generation parents, and the third track contains the alignments for the third-generation child. Reads with mapping quality <20 are filtered out, as they were not considered by our variant calling pipeline, and mismatched bases are shaded by quality score (more transparent = lower base quality). [file elife-46922-supp3.zip › supp_file_3/chr7_140,630,032_140,630,072.png]
