# Supplementary figures and images for: Large, three-generation human families reveal post-zygotic mosaicism and variability in germline mutation accumulation (part 5 of 7)
Source: eLife. 2019 Sep 24;8:e46922. doi: 10.7554/eLife.46922 (PMC6759356; doi:10.7554/eLife.46922)

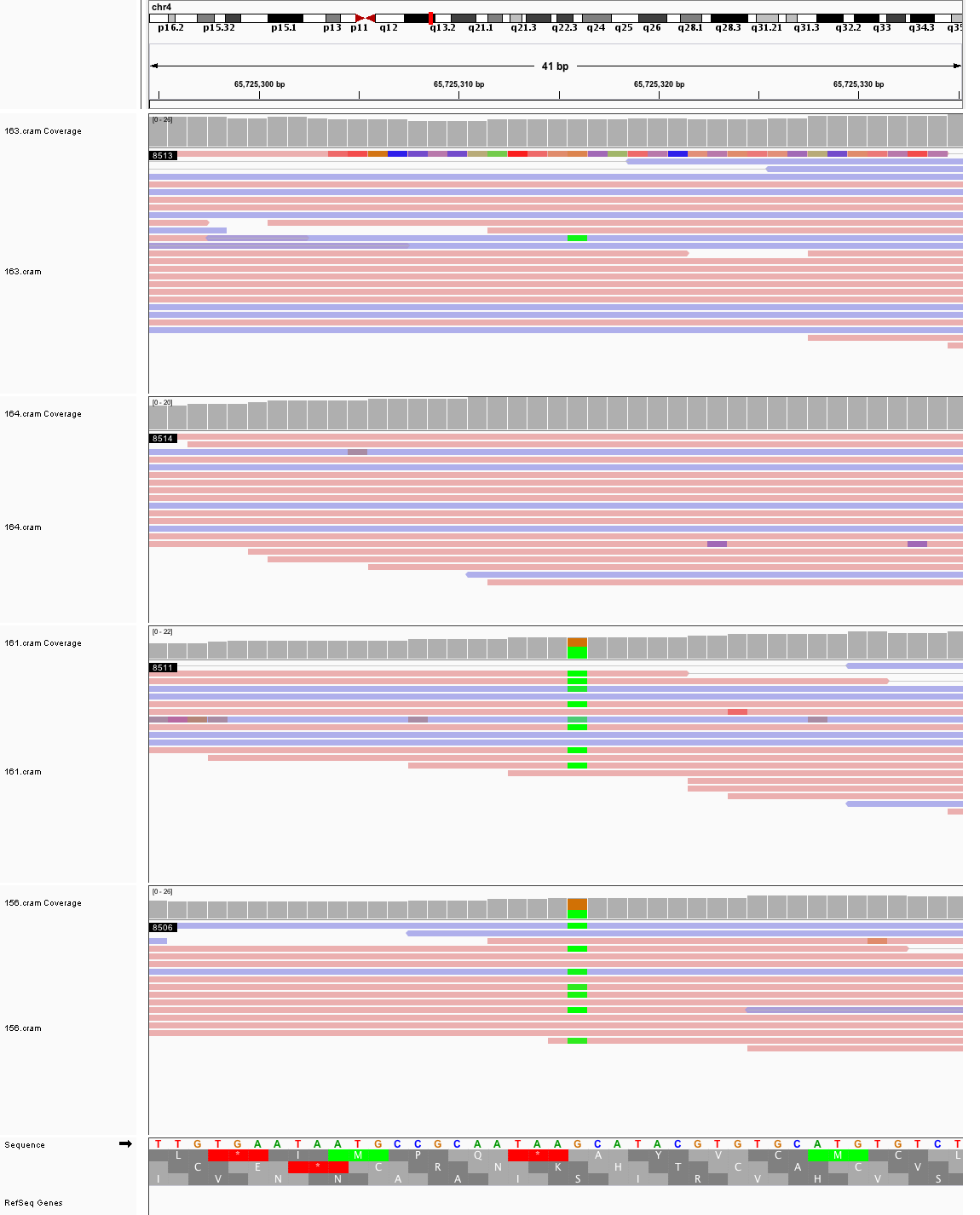

Supplement: Supplementary file 4. — All tracks below contain alignments from the third-generation children that share a DNM at the site. Reads with mapping quality <20 are filtered out, as they were not considered by our variant calling pipeline, and mismatched bases are shaded by quality score (more transparent = lower base quality). [file elife-46922-supp4.zip › supp_file_4/chr4_65,725,295_65,725,335.png]

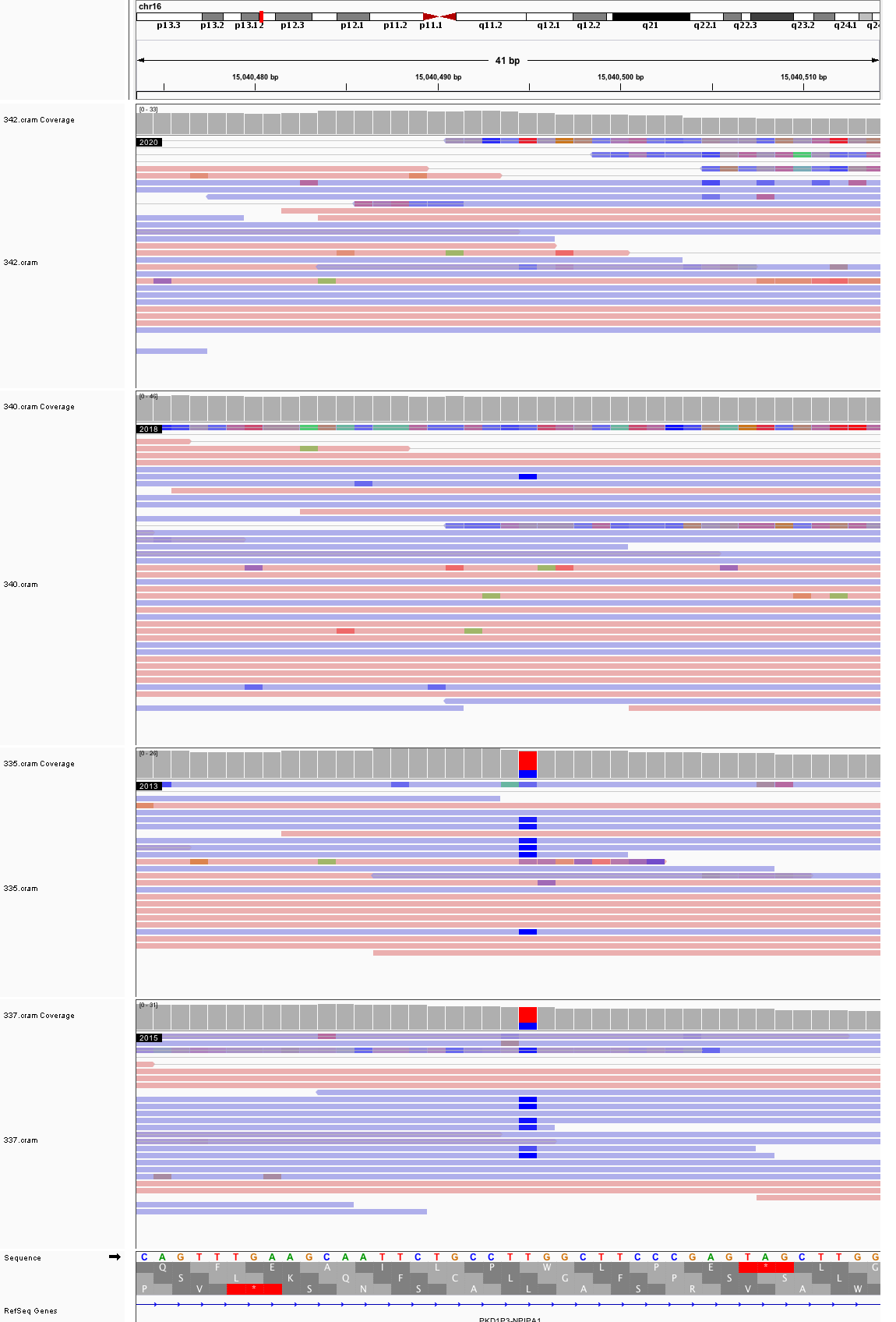

Supplement: Supplementary file 4. — All tracks below contain alignments from the third-generation children that share a DNM at the site. Reads with mapping quality <20 are filtered out, as they were not considered by our variant calling pipeline, and mismatched bases are shaded by quality score (more transparent = lower base quality). [file elife-46922-supp4.zip › supp_file_4/chr16_15,040,474_15,040,514.png]

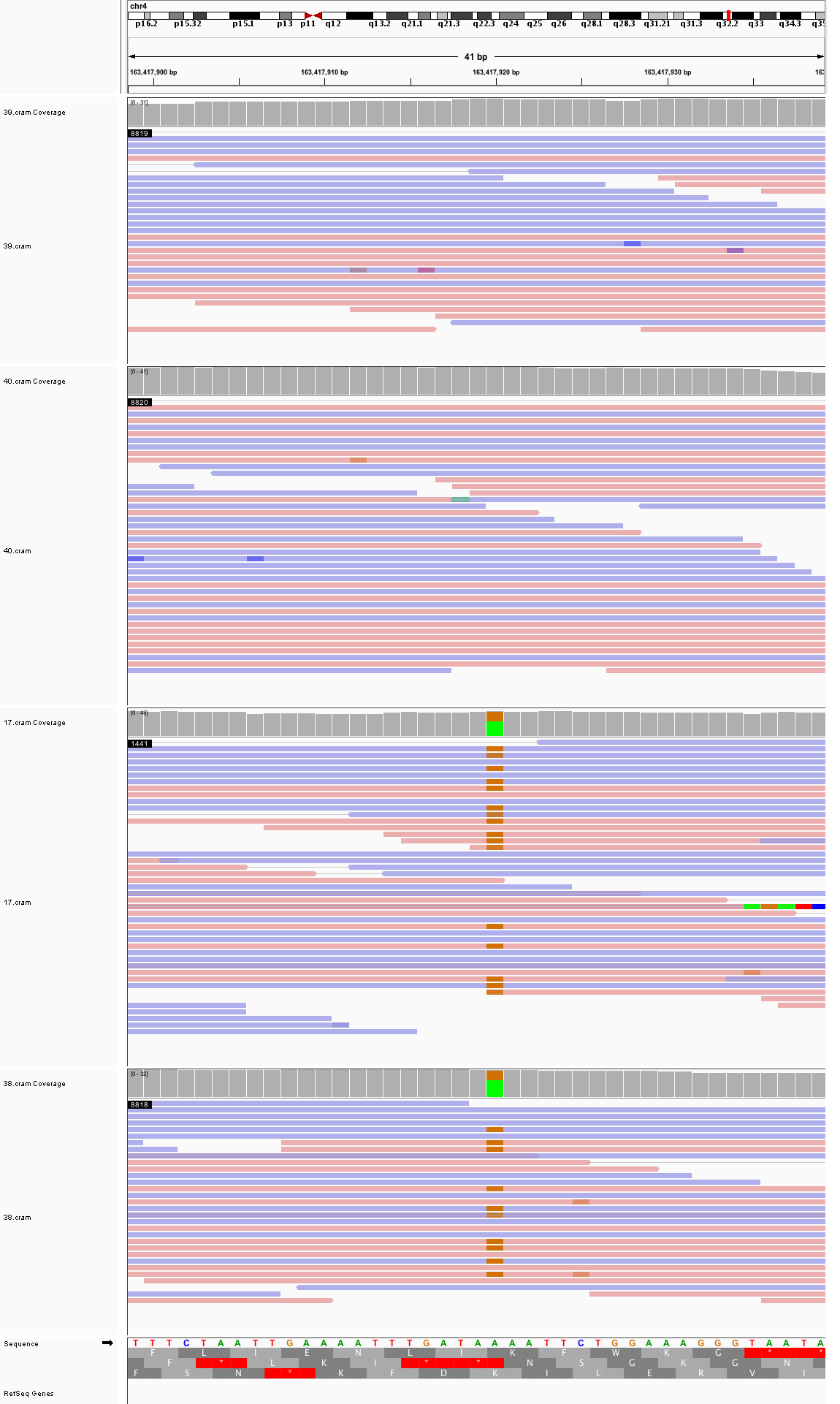

Supplement: Supplementary file 4. — All tracks below contain alignments from the third-generation children that share a DNM at the site. Reads with mapping quality <20 are filtered out, as they were not considered by our variant calling pipeline, and mismatched bases are shaded by quality score (more transparent = lower base quality). [file elife-46922-supp4.zip › supp_file_4/chr4_163,417,899_163,417,939.png]

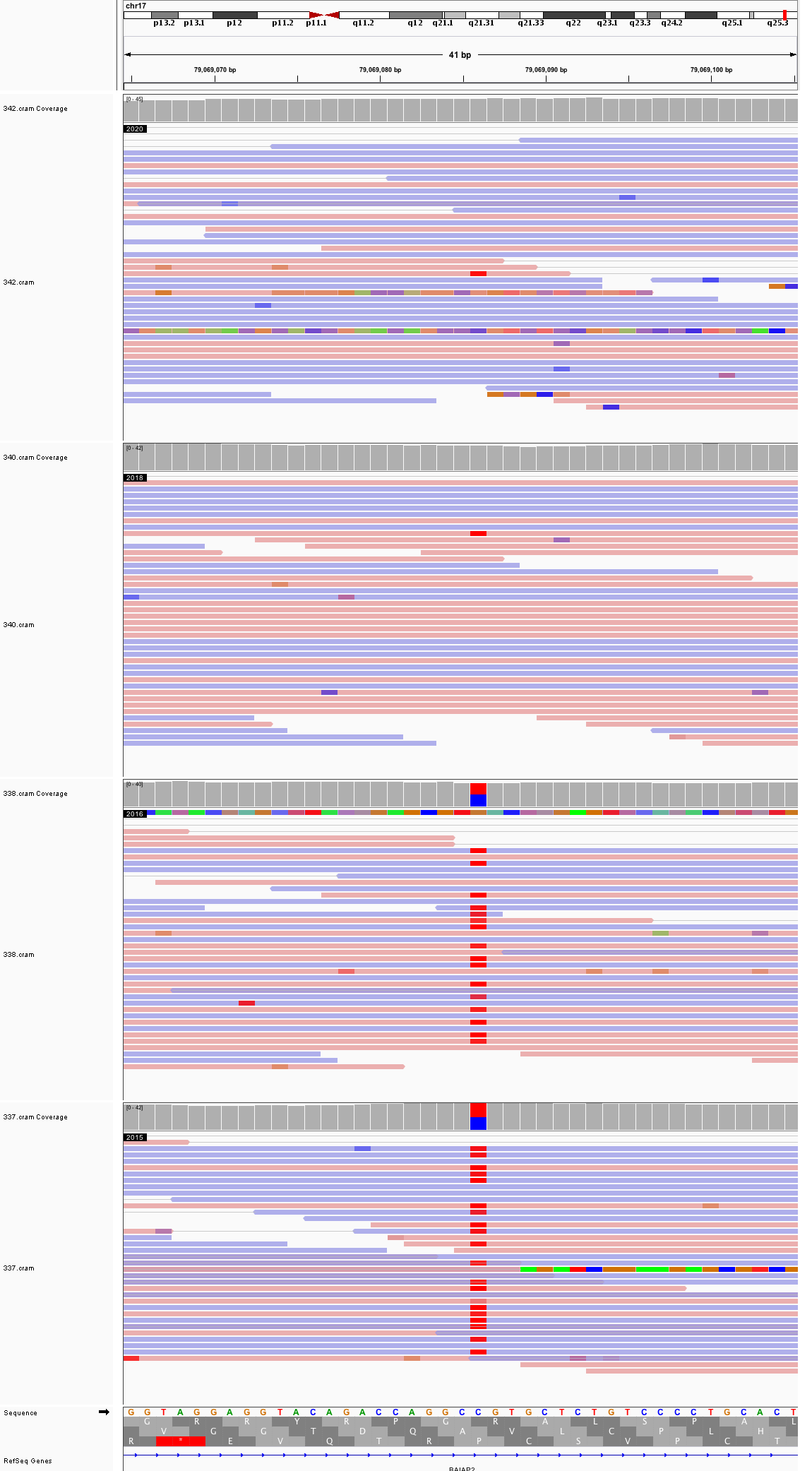

Supplement: Supplementary file 4. — All tracks below contain alignments from the third-generation children that share a DNM at the site. Reads with mapping quality <20 are filtered out, as they were not considered by our variant calling pipeline, and mismatched bases are shaded by quality score (more transparent = lower base quality). [file elife-46922-supp4.zip › supp_file_4/chr17_79,069,065_79,069,105.png]

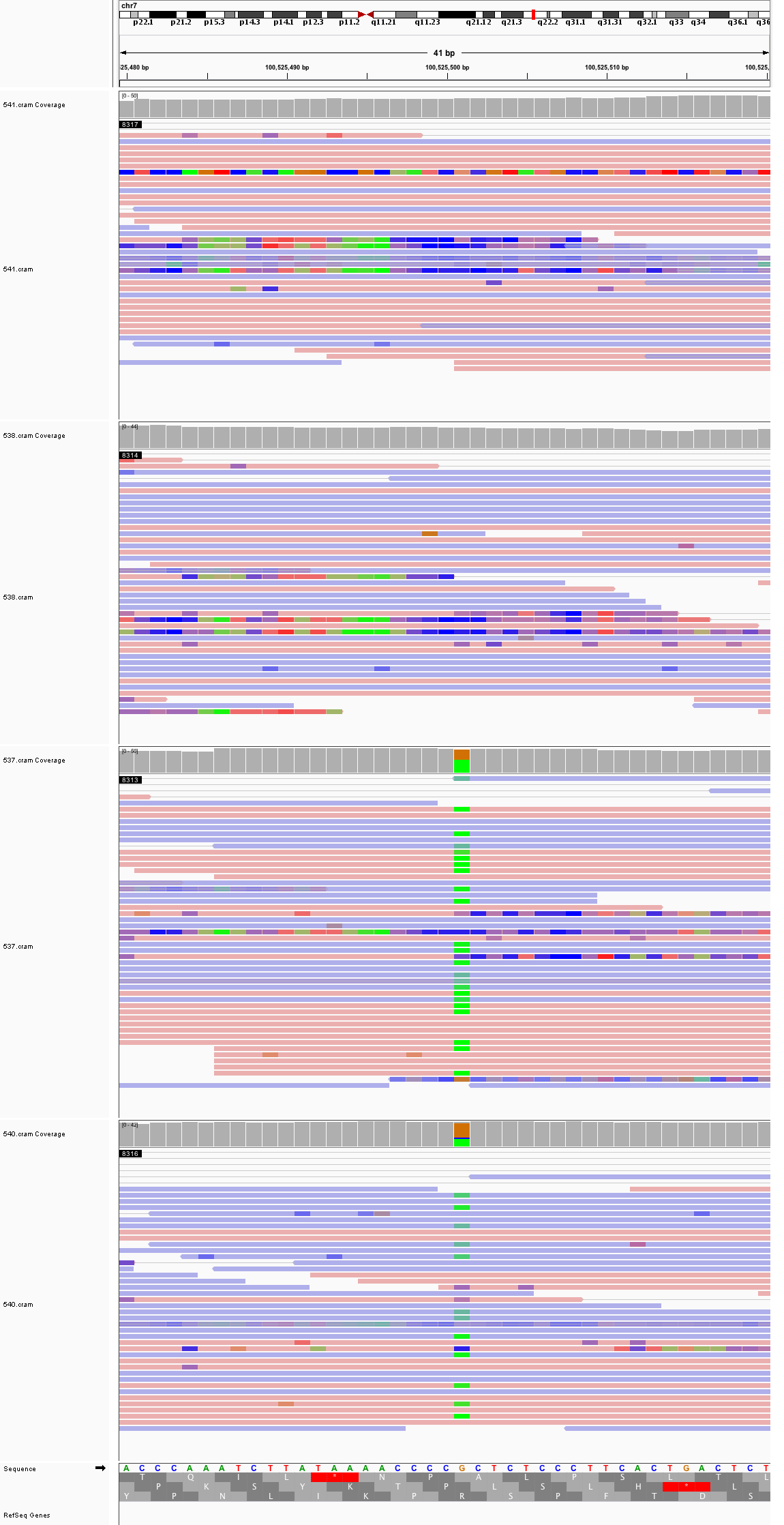

Supplement: Supplementary file 4. — All tracks below contain alignments from the third-generation children that share a DNM at the site. Reads with mapping quality <20 are filtered out, as they were not considered by our variant calling pipeline, and mismatched bases are shaded by quality score (more transparent = lower base quality). [file elife-46922-supp4.zip › supp_file_4/chr7_100,525,480_100,525,520.png]

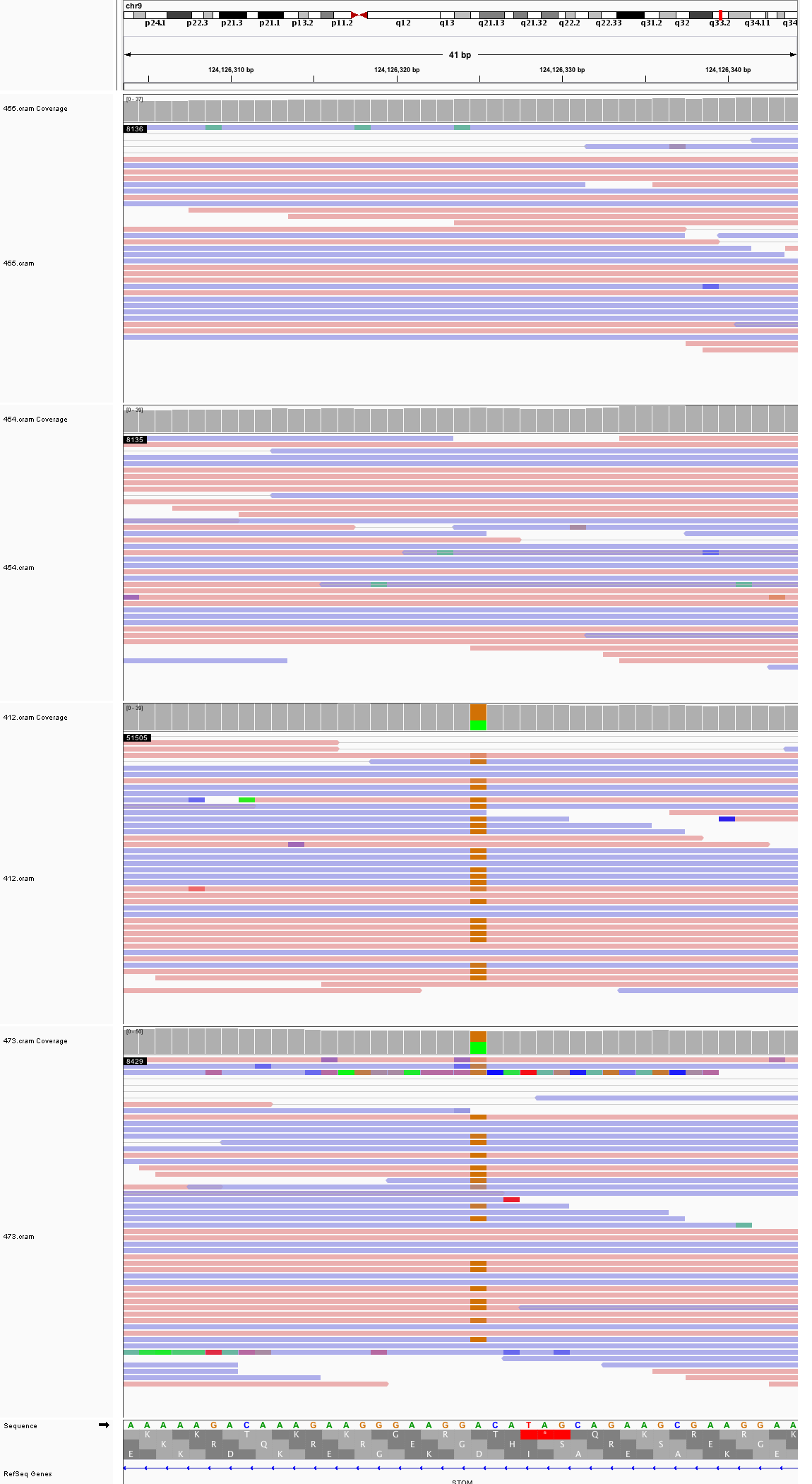

Supplement: Supplementary file 4. — All tracks below contain alignments from the third-generation children that share a DNM at the site. Reads with mapping quality <20 are filtered out, as they were not considered by our variant calling pipeline, and mismatched bases are shaded by quality score (more transparent = lower base quality). [file elife-46922-supp4.zip › supp_file_4/chr9_124,126,304_124,126,344.png]

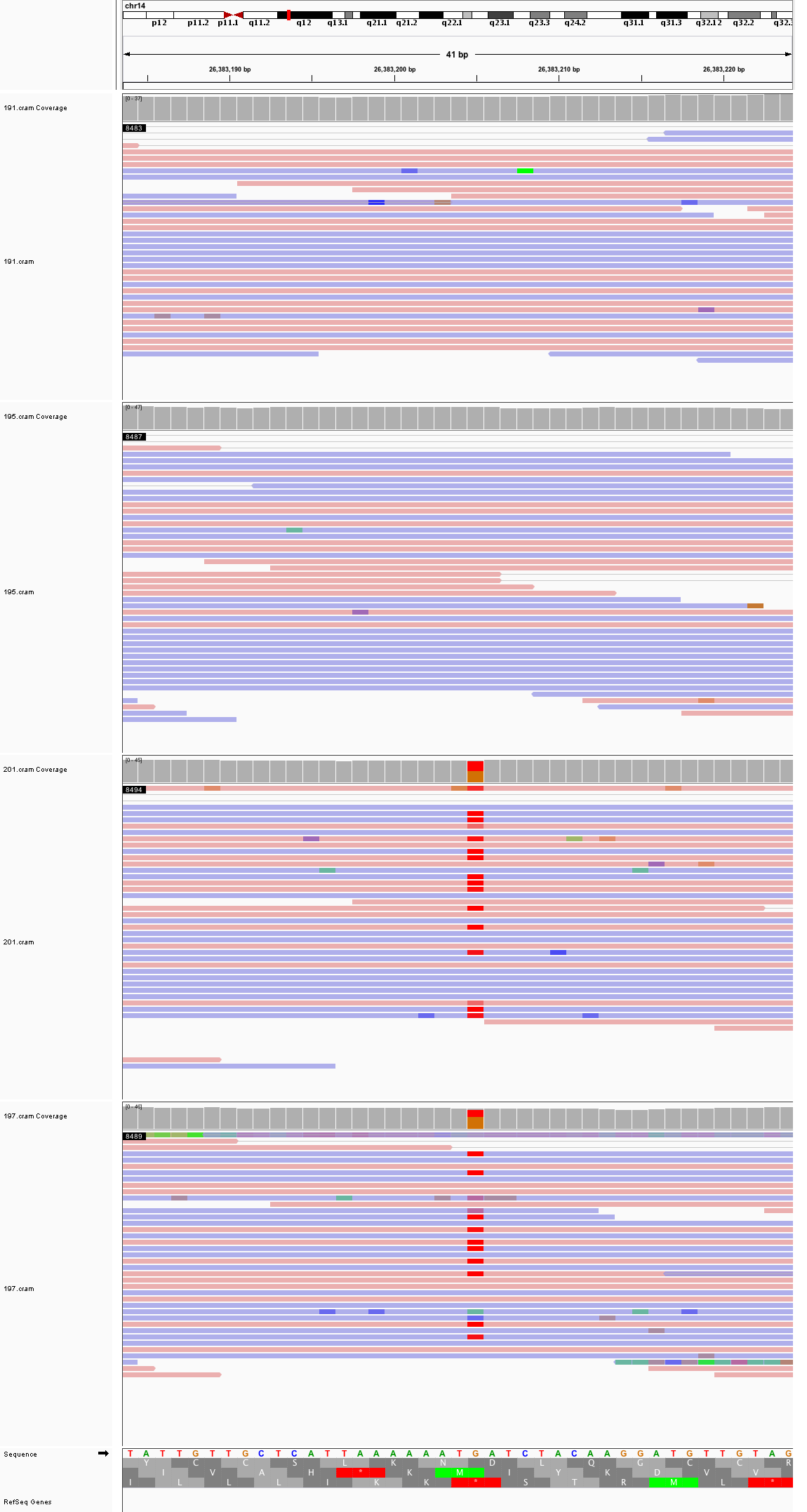

Supplement: Supplementary file 4. — All tracks below contain alignments from the third-generation children that share a DNM at the site. Reads with mapping quality <20 are filtered out, as they were not considered by our variant calling pipeline, and mismatched bases are shaded by quality score (more transparent = lower base quality). [file elife-46922-supp4.zip › supp_file_4/chr14_26,383,184_26,383,224.png]

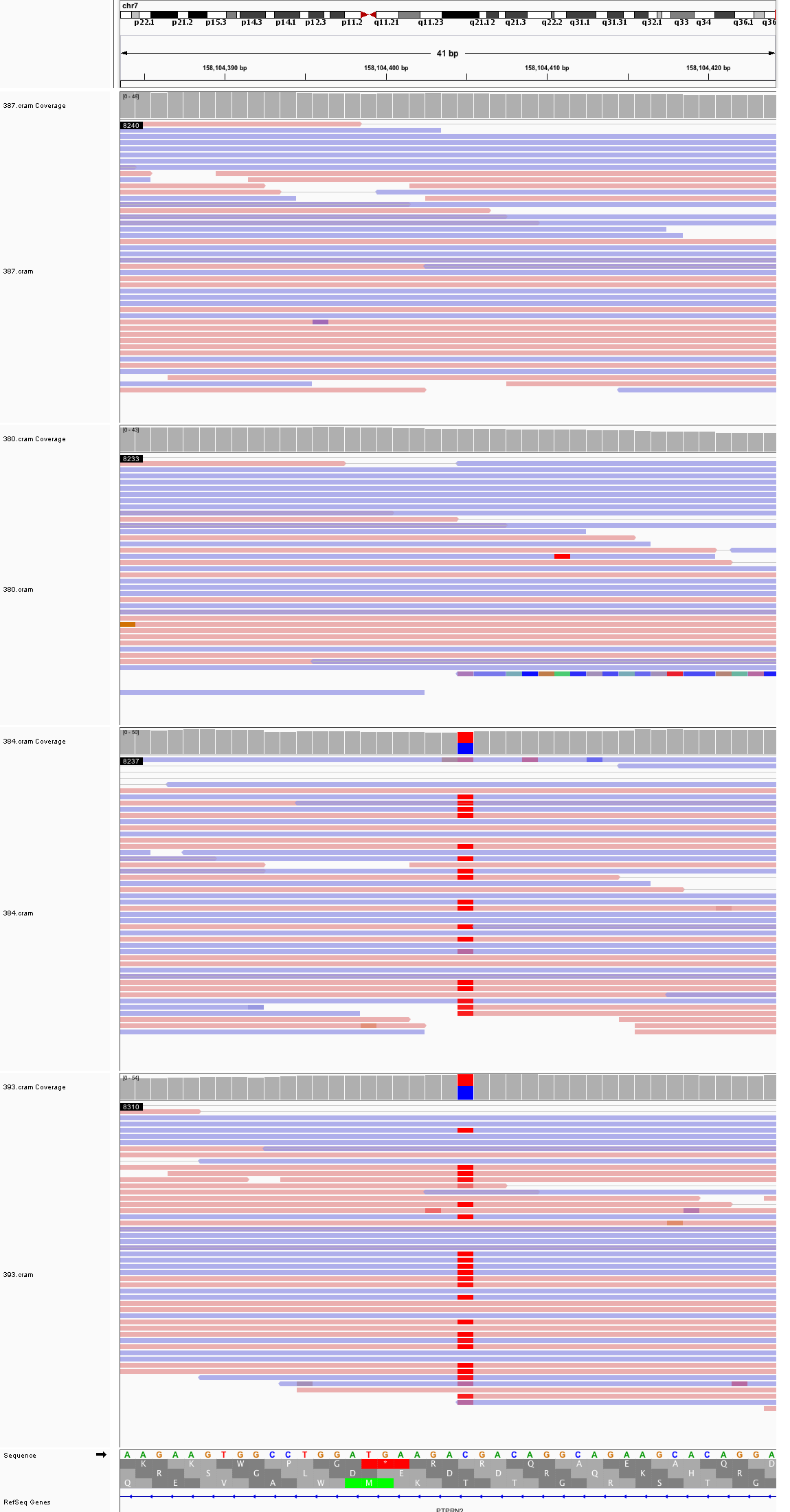

Supplement: Supplementary file 4. — All tracks below contain alignments from the third-generation children that share a DNM at the site. Reads with mapping quality <20 are filtered out, as they were not considered by our variant calling pipeline, and mismatched bases are shaded by quality score (more transparent = lower base quality). [file elife-46922-supp4.zip › supp_file_4/chr7_158,104,384_158,104,424.png]

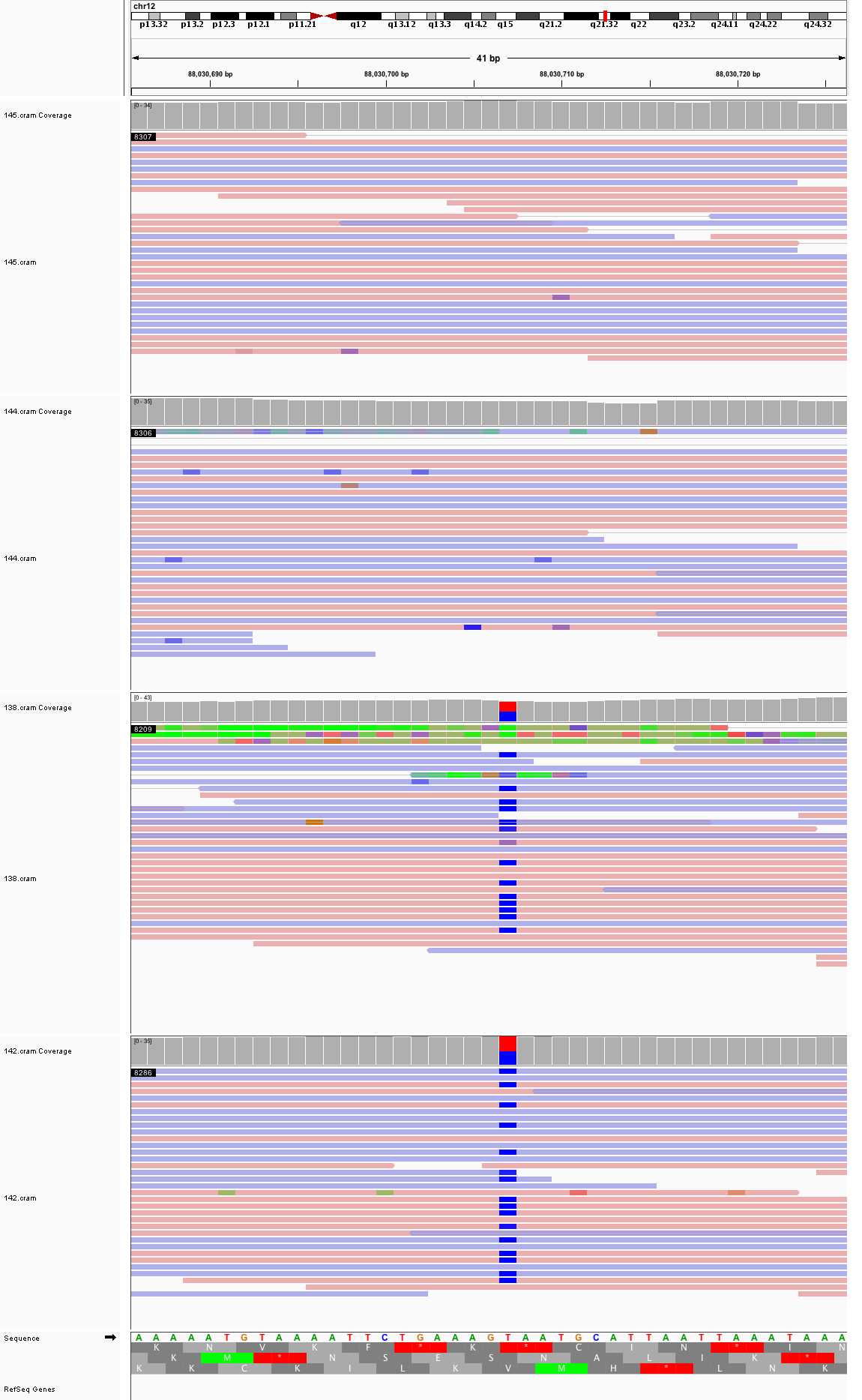

Supplement: Supplementary file 4. — All tracks below contain alignments from the third-generation children that share a DNM at the site. Reads with mapping quality <20 are filtered out, as they were not considered by our variant calling pipeline, and mismatched bases are shaded by quality score (more transparent = lower base quality). [file elife-46922-supp4.zip › supp_file_4/chr12_88,030,686_88,030,726.png]

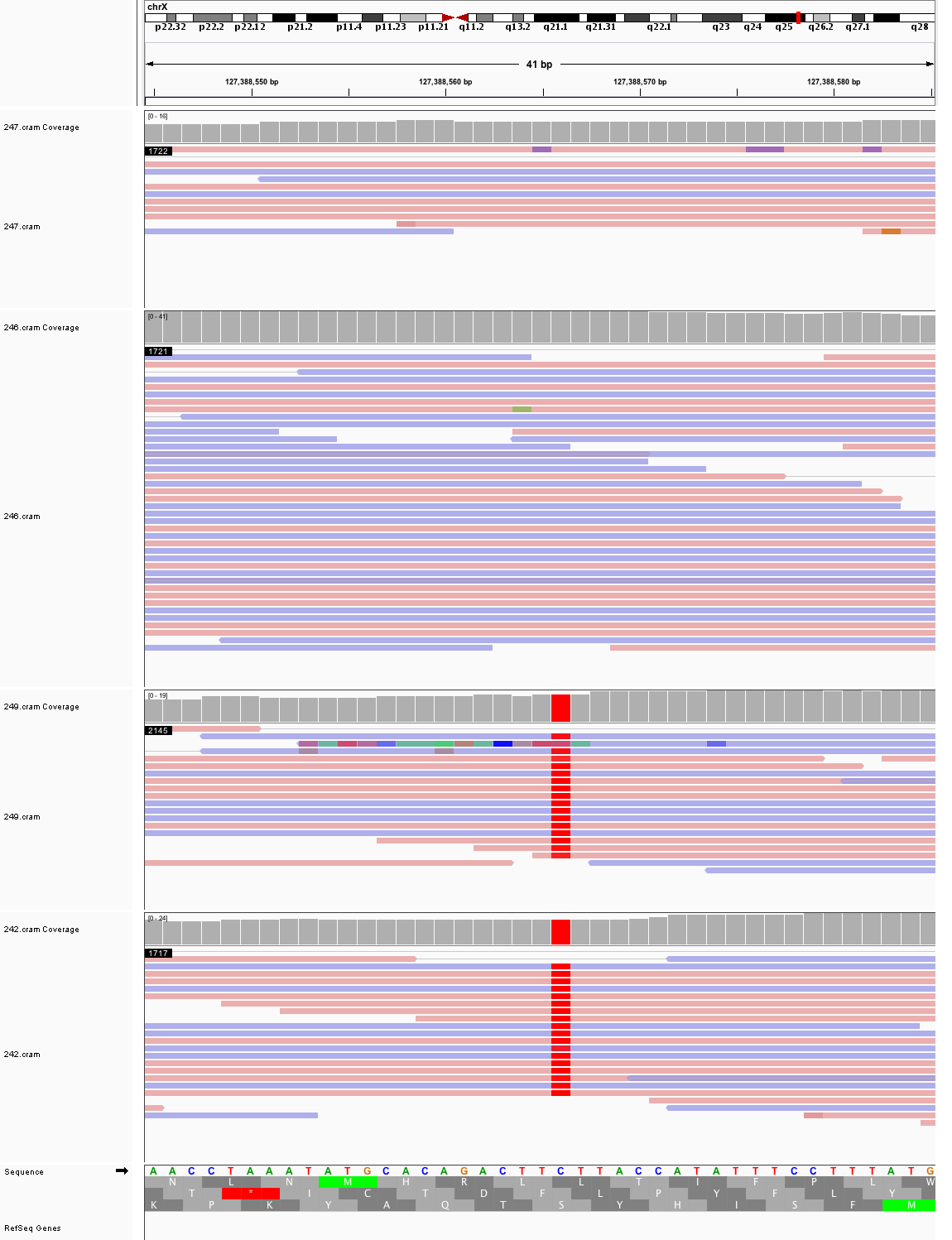

Supplement: Supplementary file 4. — All tracks below contain alignments from the third-generation children that share a DNM at the site. Reads with mapping quality <20 are filtered out, as they were not considered by our variant calling pipeline, and mismatched bases are shaded by quality score (more transparent = lower base quality). [file elife-46922-supp4.zip › supp_file_4/chrX_127,388,545_127,388,585.png]

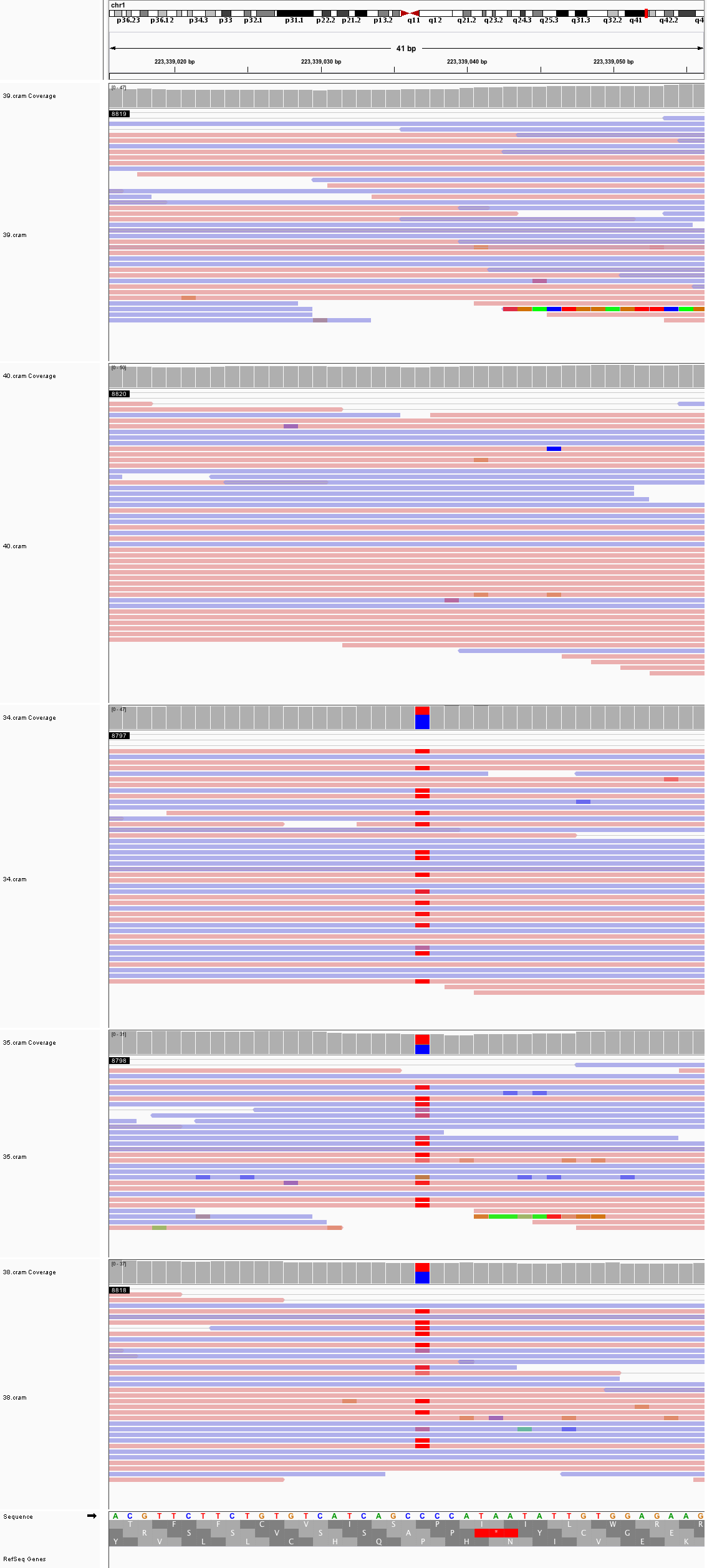

Supplement: Supplementary file 4. — All tracks below contain alignments from the third-generation children that share a DNM at the site. Reads with mapping quality <20 are filtered out, as they were not considered by our variant calling pipeline, and mismatched bases are shaded by quality score (more transparent = lower base quality). [file elife-46922-supp4.zip › supp_file_4/chr1_223,339,016_223,339,056.png]

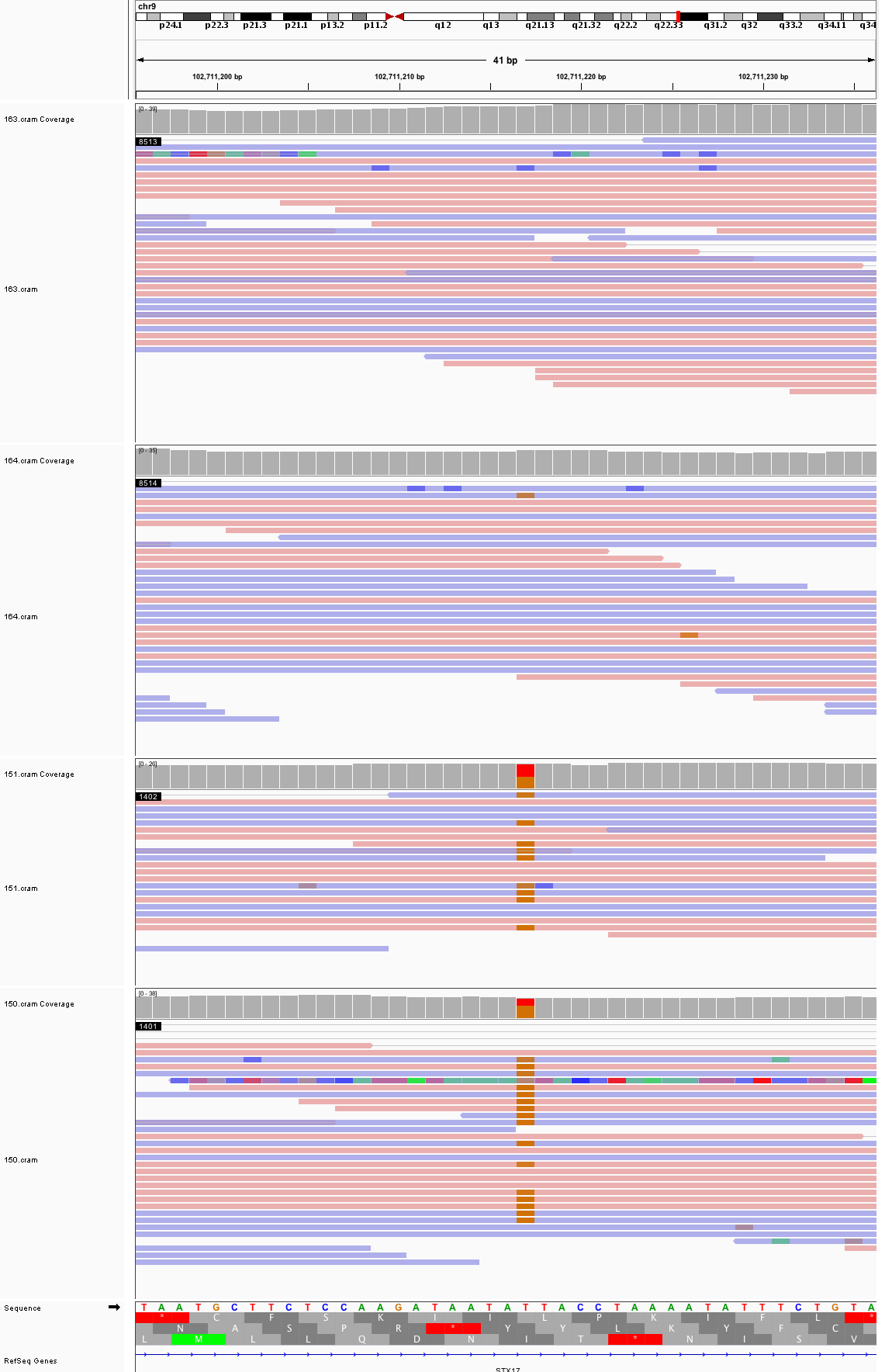

Supplement: Supplementary file 4. — All tracks below contain alignments from the third-generation children that share a DNM at the site. Reads with mapping quality <20 are filtered out, as they were not considered by our variant calling pipeline, and mismatched bases are shaded by quality score (more transparent = lower base quality). [file elife-46922-supp4.zip › supp_file_4/chr9_102,711,196_102,711,236.png]

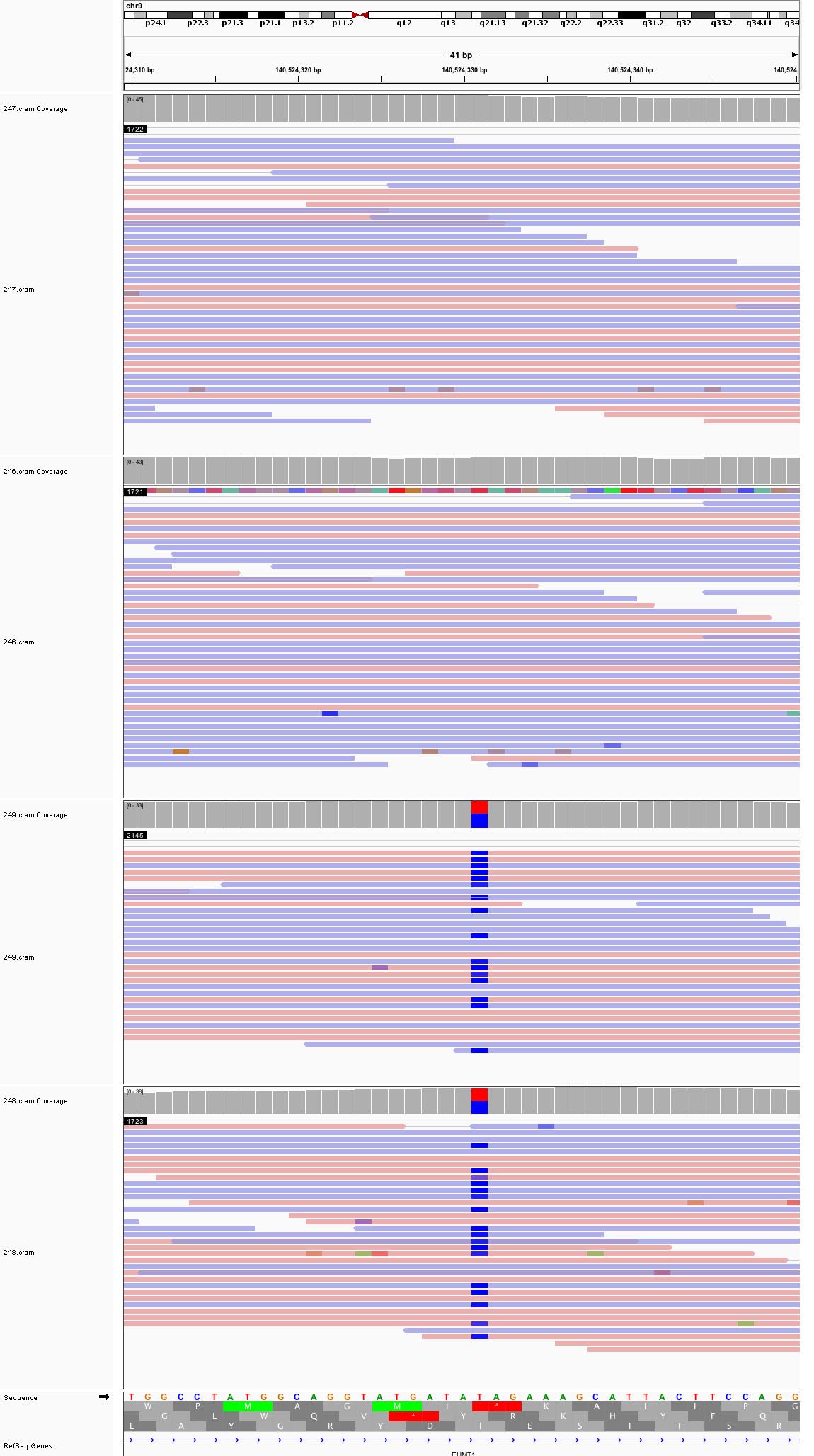

Supplement: Supplementary file 4. — All tracks below contain alignments from the third-generation children that share a DNM at the site. Reads with mapping quality <20 are filtered out, as they were not considered by our variant calling pipeline, and mismatched bases are shaded by quality score (more transparent = lower base quality). [file elife-46922-supp4.zip › supp_file_4/chr9_140,524,310_140,524,350.png]

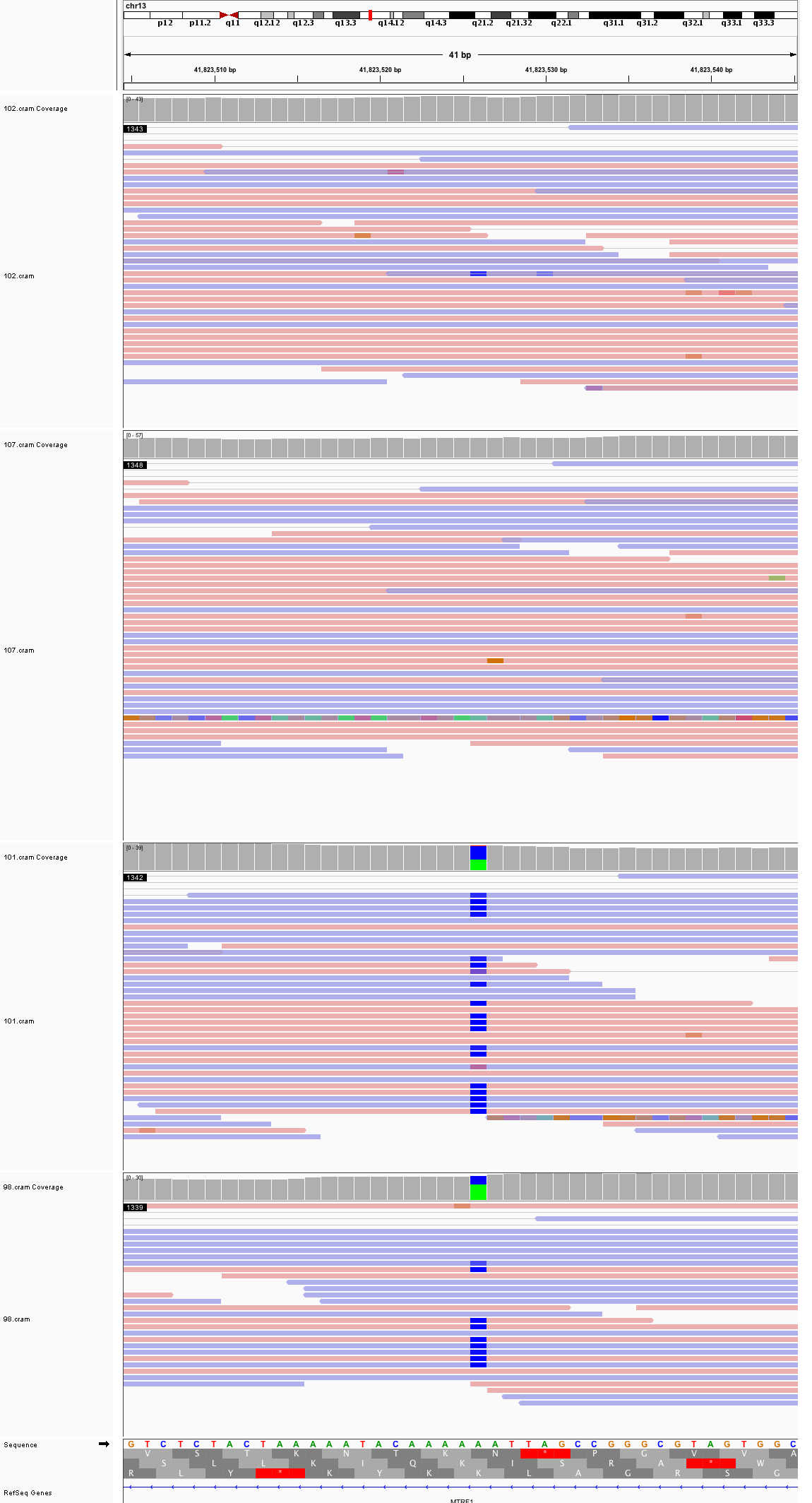

Supplement: Supplementary file 4. — All tracks below contain alignments from the third-generation children that share a DNM at the site. Reads with mapping quality <20 are filtered out, as they were not considered by our variant calling pipeline, and mismatched bases are shaded by quality score (more transparent = lower base quality). [file elife-46922-supp4.zip › supp_file_4/chr13_41,823,505_41,823,545.png]

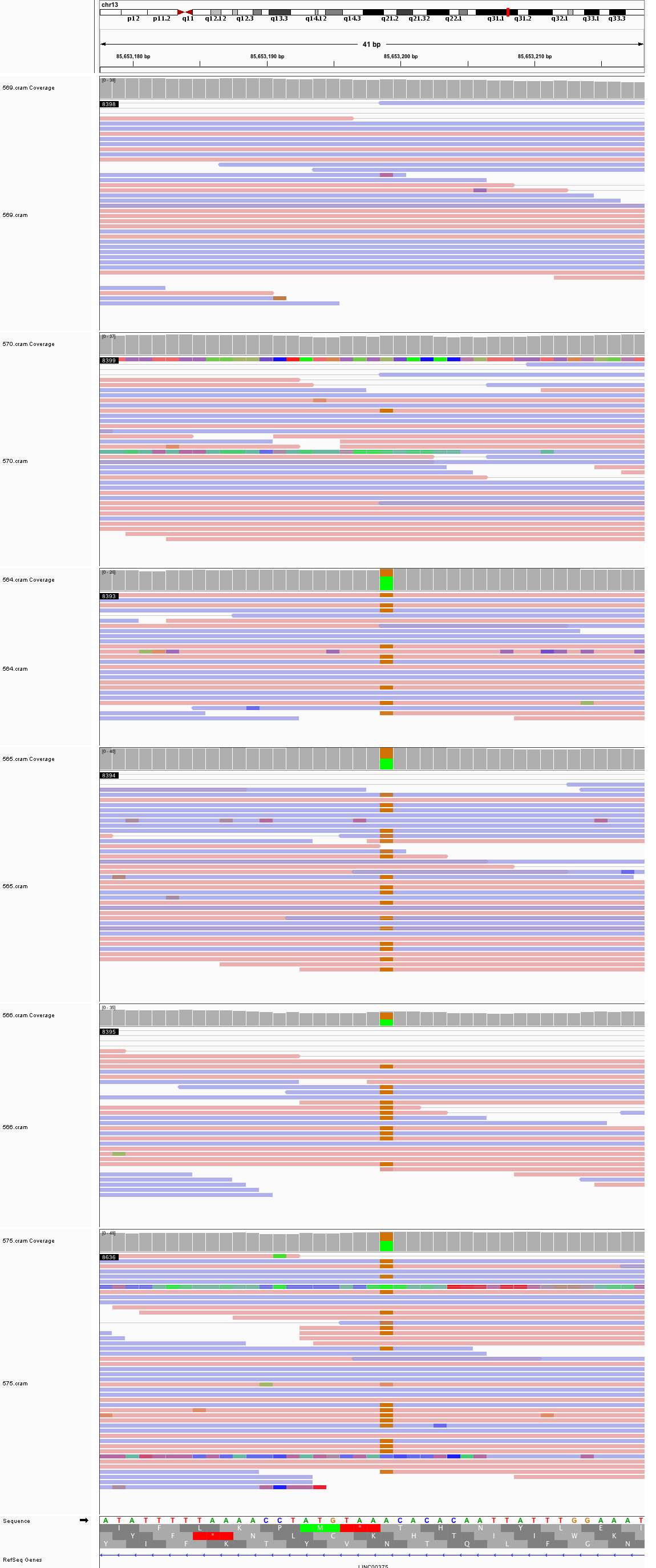

Supplement: Supplementary file 4. — All tracks below contain alignments from the third-generation children that share a DNM at the site. Reads with mapping quality <20 are filtered out, as they were not considered by our variant calling pipeline, and mismatched bases are shaded by quality score (more transparent = lower base quality). [file elife-46922-supp4.zip › supp_file_4/chr13_85,653,178_85,653,218.png]

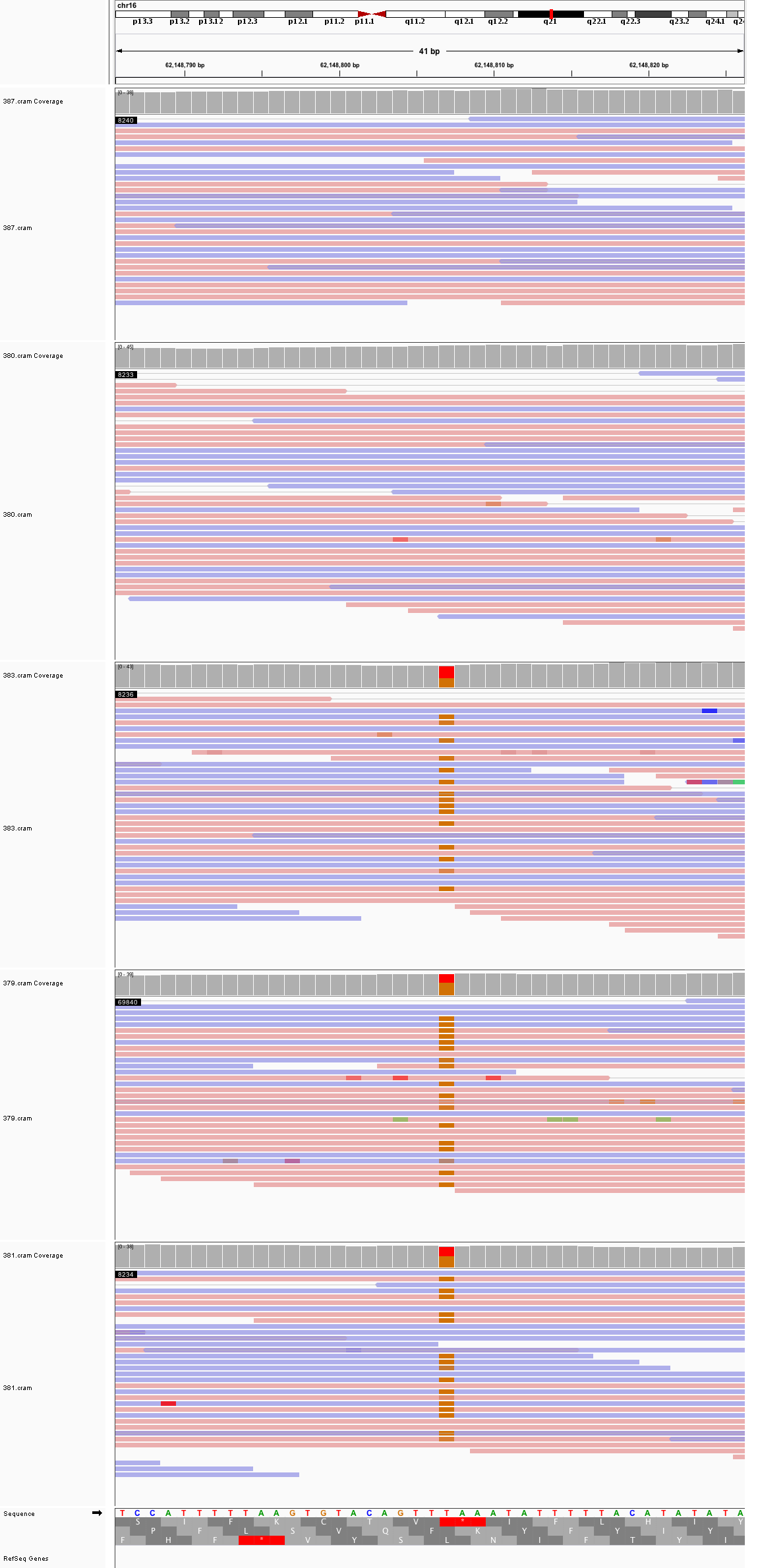

Supplement: Supplementary file 4. — All tracks below contain alignments from the third-generation children that share a DNM at the site. Reads with mapping quality <20 are filtered out, as they were not considered by our variant calling pipeline, and mismatched bases are shaded by quality score (more transparent = lower base quality). [file elife-46922-supp4.zip › supp_file_4/chr16_62,148,786_62,148,826.png]

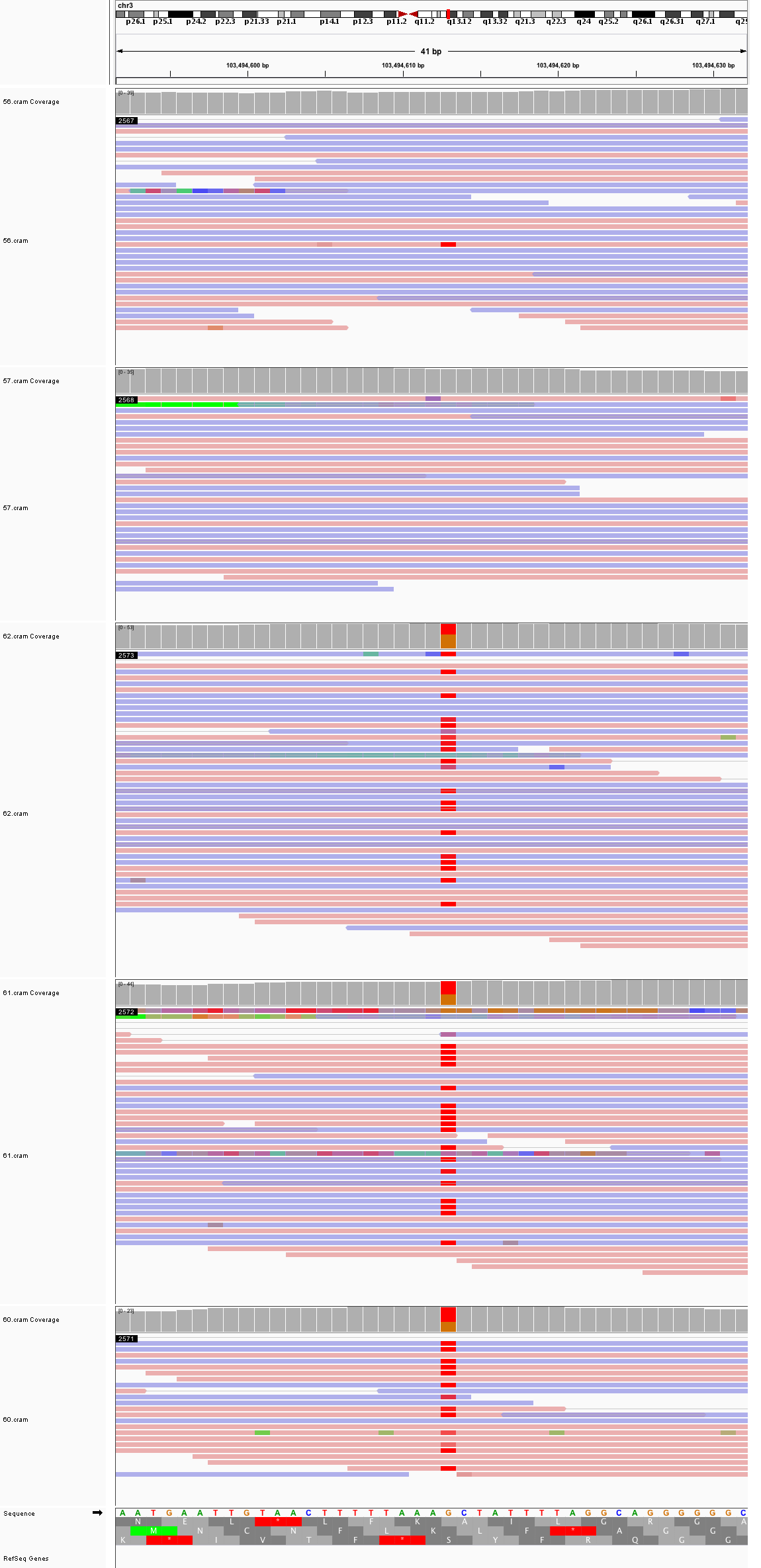

Supplement: Supplementary file 4. — All tracks below contain alignments from the third-generation children that share a DNM at the site. Reads with mapping quality <20 are filtered out, as they were not considered by our variant calling pipeline, and mismatched bases are shaded by quality score (more transparent = lower base quality). [file elife-46922-supp4.zip › supp_file_4/chr3_103,494,592_103,494,632.png]

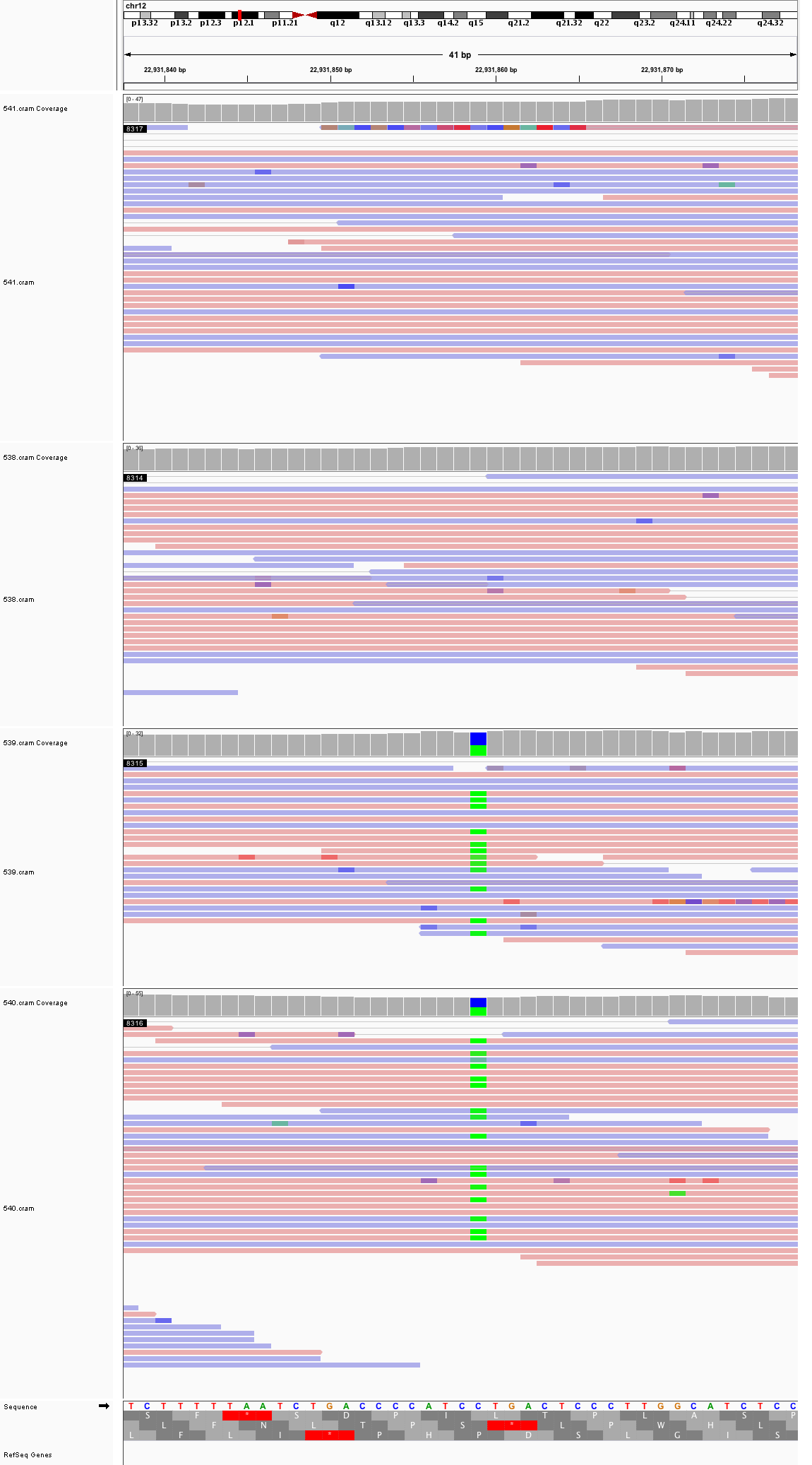

Supplement: Supplementary file 4. — All tracks below contain alignments from the third-generation children that share a DNM at the site. Reads with mapping quality <20 are filtered out, as they were not considered by our variant calling pipeline, and mismatched bases are shaded by quality score (more transparent = lower base quality). [file elife-46922-supp4.zip › supp_file_4/chr12_22,931,838_22,931,878.png]

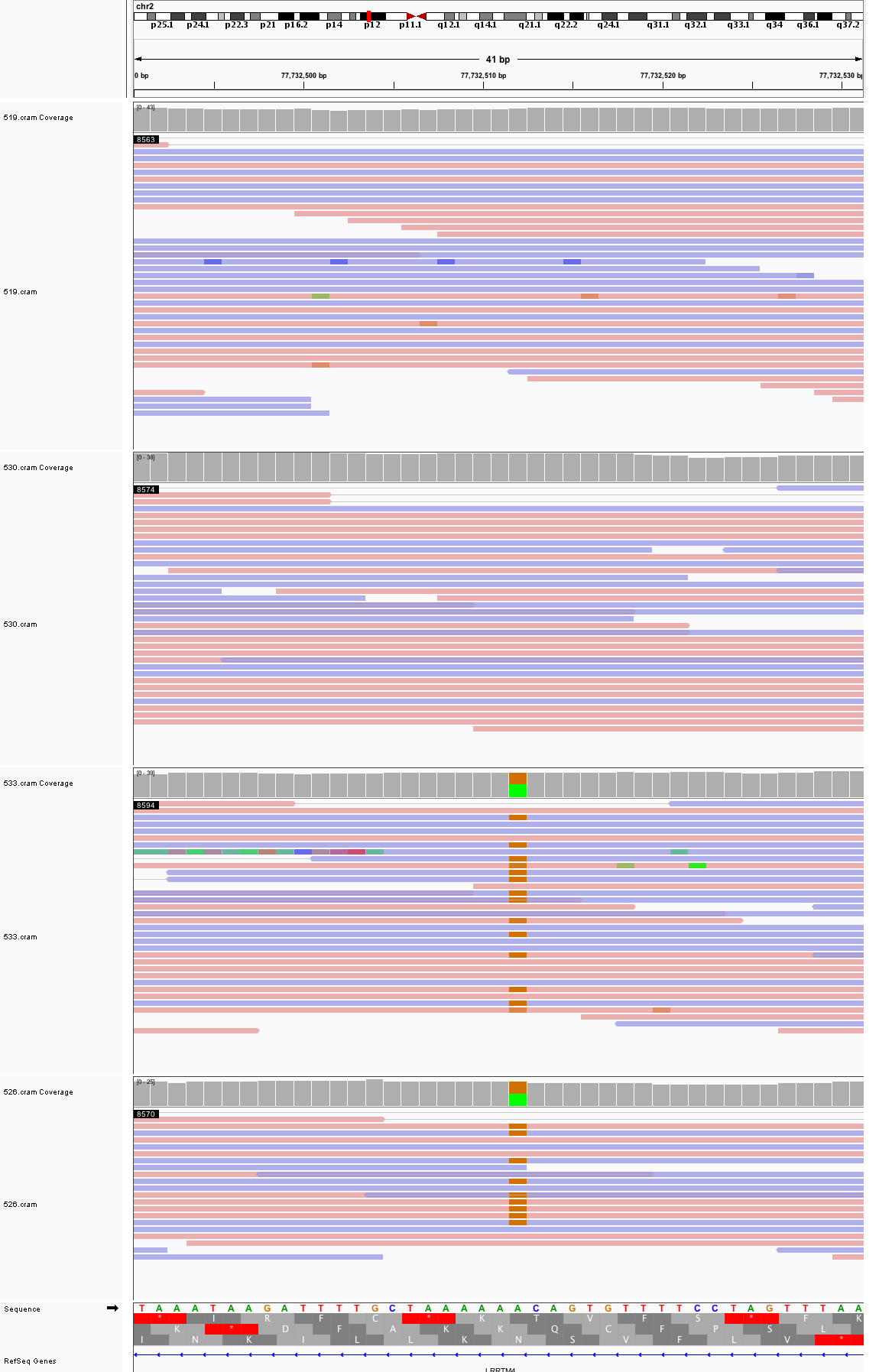

Supplement: Supplementary file 4. — All tracks below contain alignments from the third-generation children that share a DNM at the site. Reads with mapping quality <20 are filtered out, as they were not considered by our variant calling pipeline, and mismatched bases are shaded by quality score (more transparent = lower base quality). [file elife-46922-supp4.zip › supp_file_4/chr2_77,732,491_77,732,531.png]

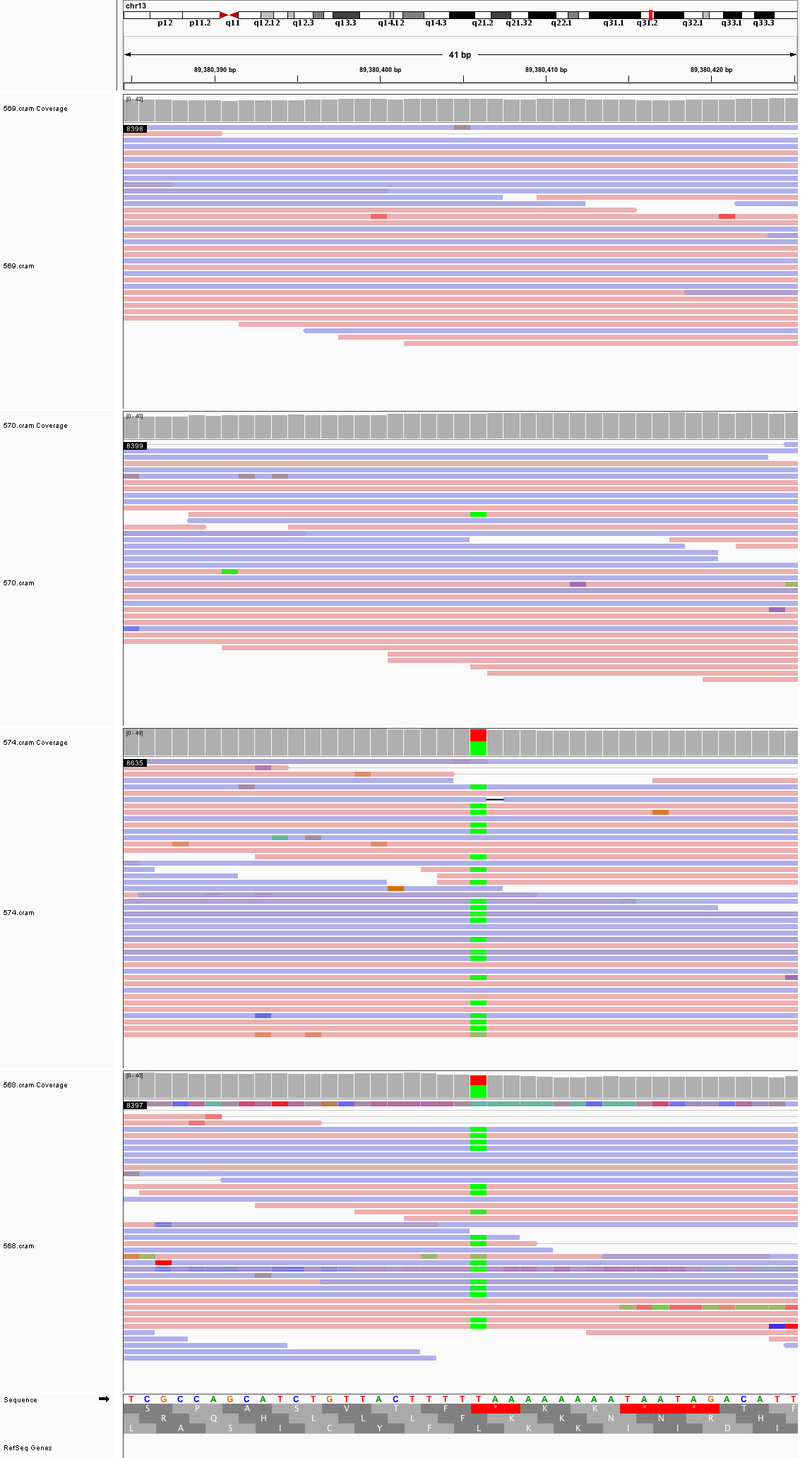

Supplement: Supplementary file 4. — All tracks below contain alignments from the third-generation children that share a DNM at the site. Reads with mapping quality <20 are filtered out, as they were not considered by our variant calling pipeline, and mismatched bases are shaded by quality score (more transparent = lower base quality). [file elife-46922-supp4.zip › supp_file_4/chr13_89,380,385_89,380,425.png]

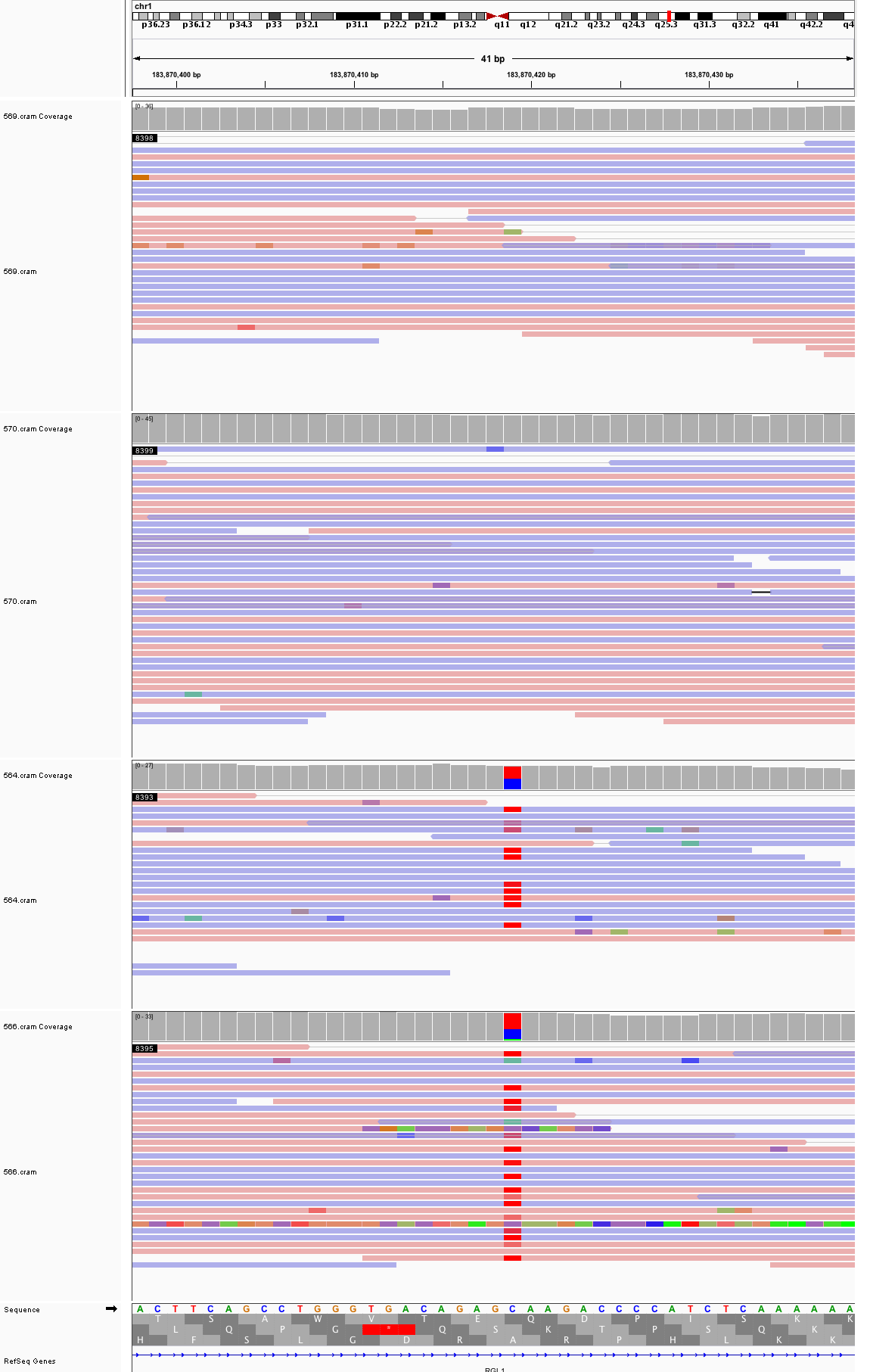

Supplement: Supplementary file 4. — All tracks below contain alignments from the third-generation children that share a DNM at the site. Reads with mapping quality <20 are filtered out, as they were not considered by our variant calling pipeline, and mismatched bases are shaded by quality score (more transparent = lower base quality). [file elife-46922-supp4.zip › supp_file_4/chr1_183,870,398_183,870,438.png]

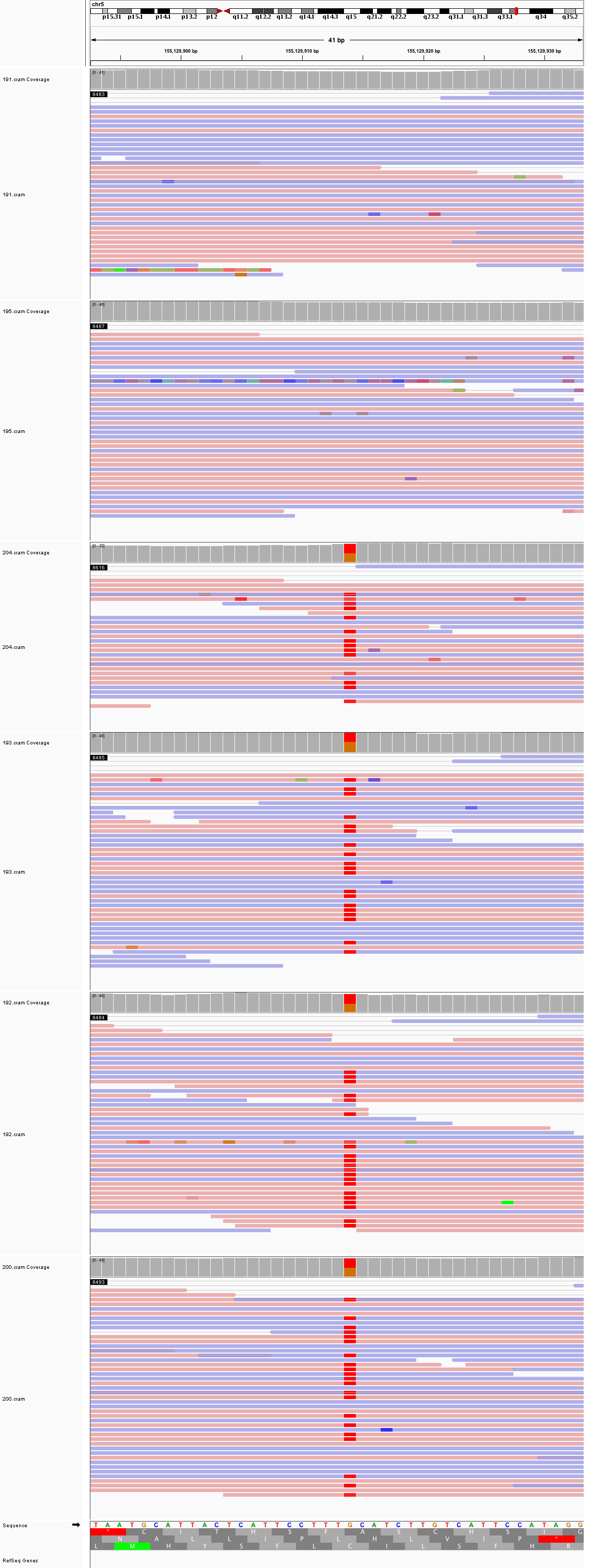

Supplement: Supplementary file 4. — All tracks below contain alignments from the third-generation children that share a DNM at the site. Reads with mapping quality <20 are filtered out, as they were not considered by our variant calling pipeline, and mismatched bases are shaded by quality score (more transparent = lower base quality). [file elife-46922-supp4.zip › supp_file_4/chr5_155,129,893_155,129,933.png]

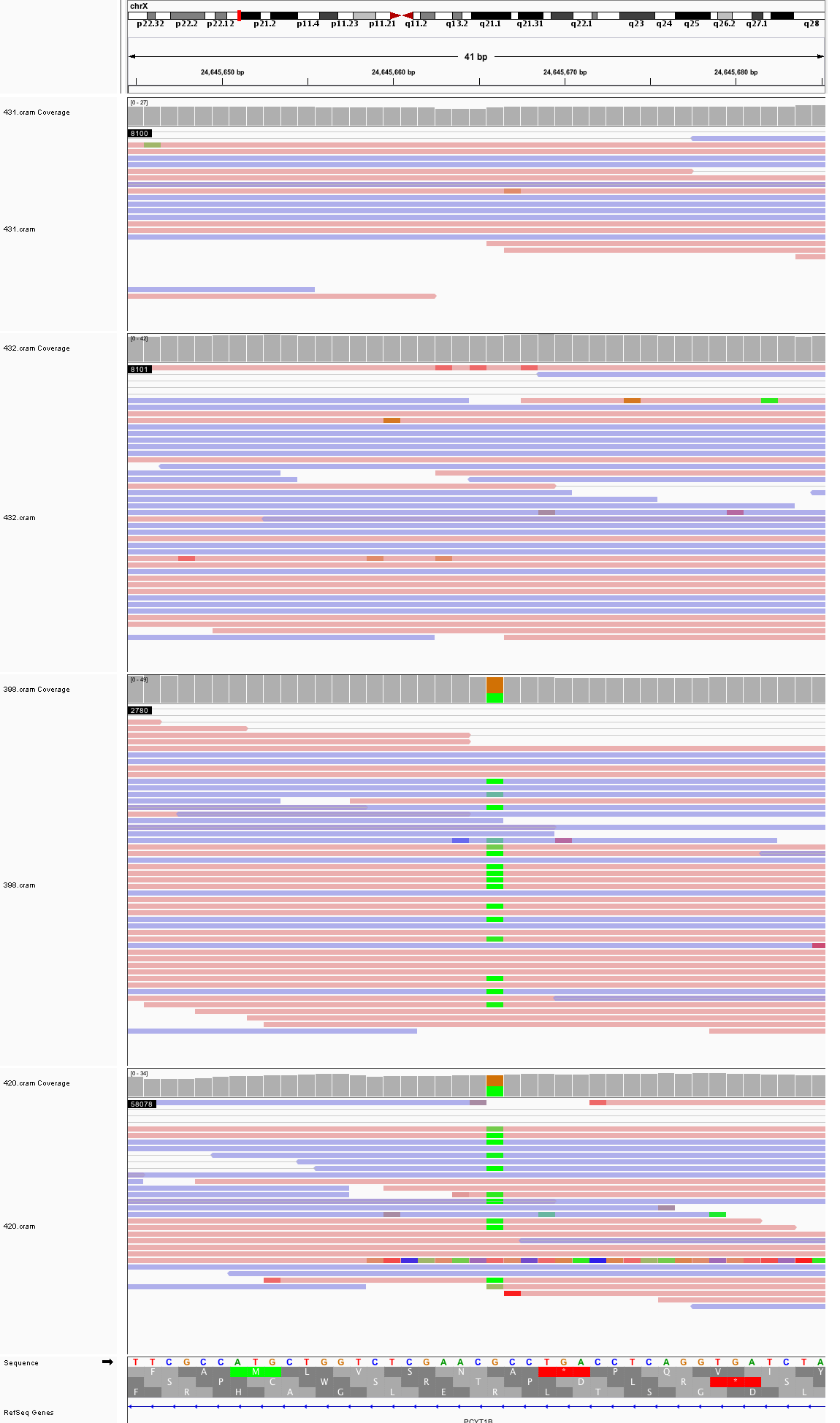

Supplement: Supplementary file 4. — All tracks below contain alignments from the third-generation children that share a DNM at the site. Reads with mapping quality <20 are filtered out, as they were not considered by our variant calling pipeline, and mismatched bases are shaded by quality score (more transparent = lower base quality). [file elife-46922-supp4.zip › supp_file_4/chrX_24,645,645_24,645,685.png]

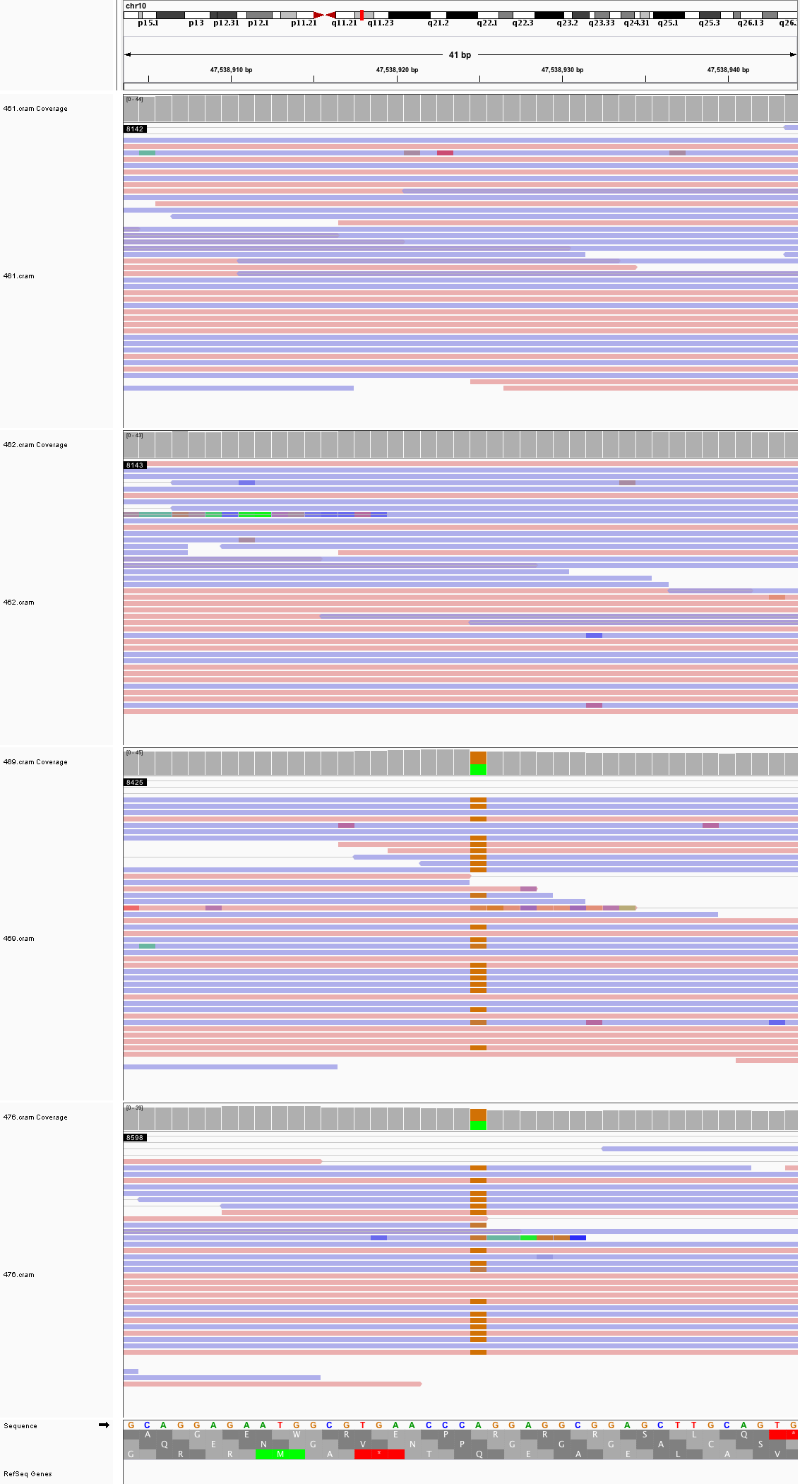

Supplement: Supplementary file 4. — All tracks below contain alignments from the third-generation children that share a DNM at the site. Reads with mapping quality <20 are filtered out, as they were not considered by our variant calling pipeline, and mismatched bases are shaded by quality score (more transparent = lower base quality). [file elife-46922-supp4.zip › supp_file_4/chr10_47,538,904_47,538,944.png]

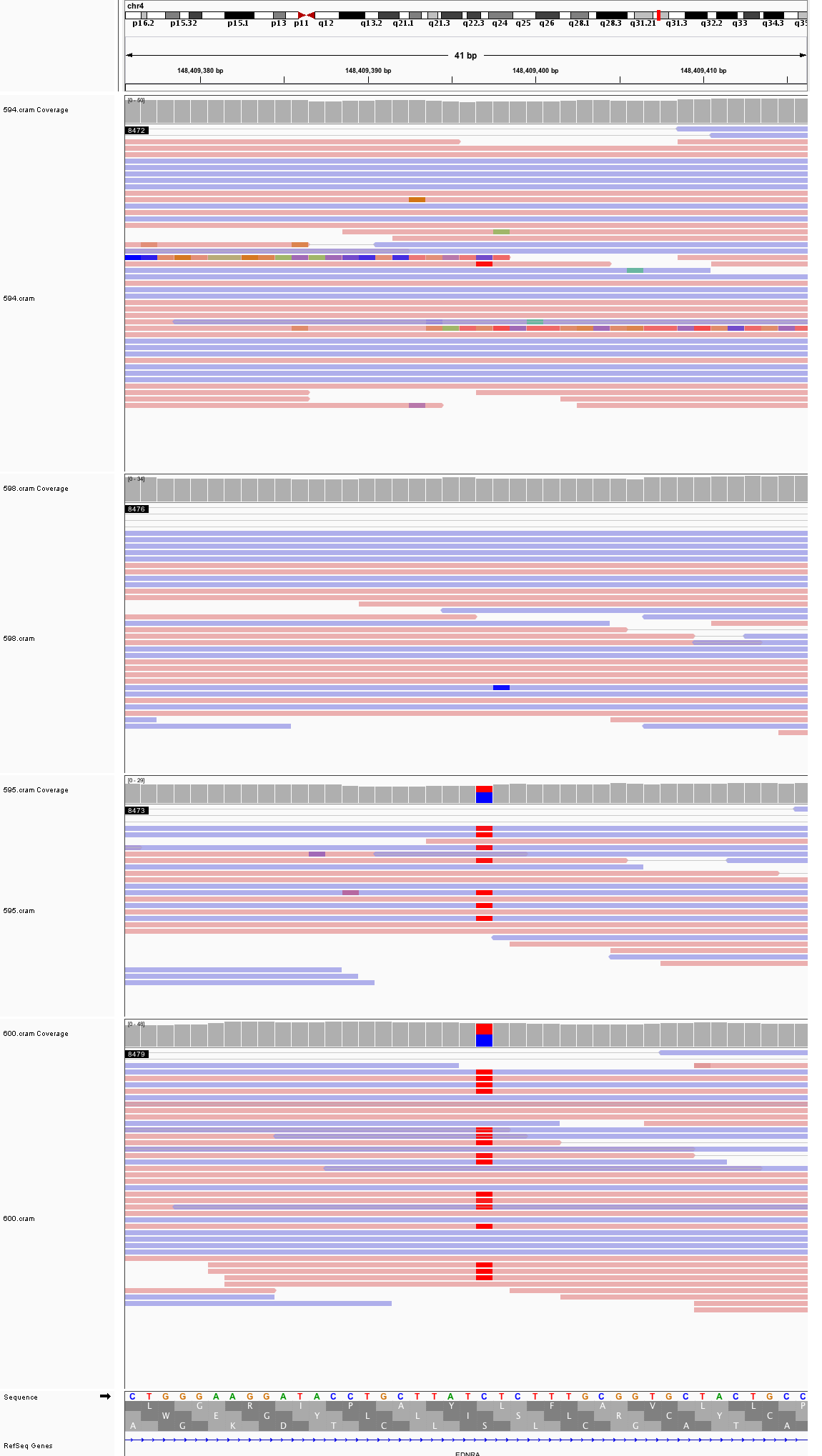

Supplement: Supplementary file 4. — All tracks below contain alignments from the third-generation children that share a DNM at the site. Reads with mapping quality <20 are filtered out, as they were not considered by our variant calling pipeline, and mismatched bases are shaded by quality score (more transparent = lower base quality). [file elife-46922-supp4.zip › supp_file_4/chr4_148,409,376_148,409,416.png]

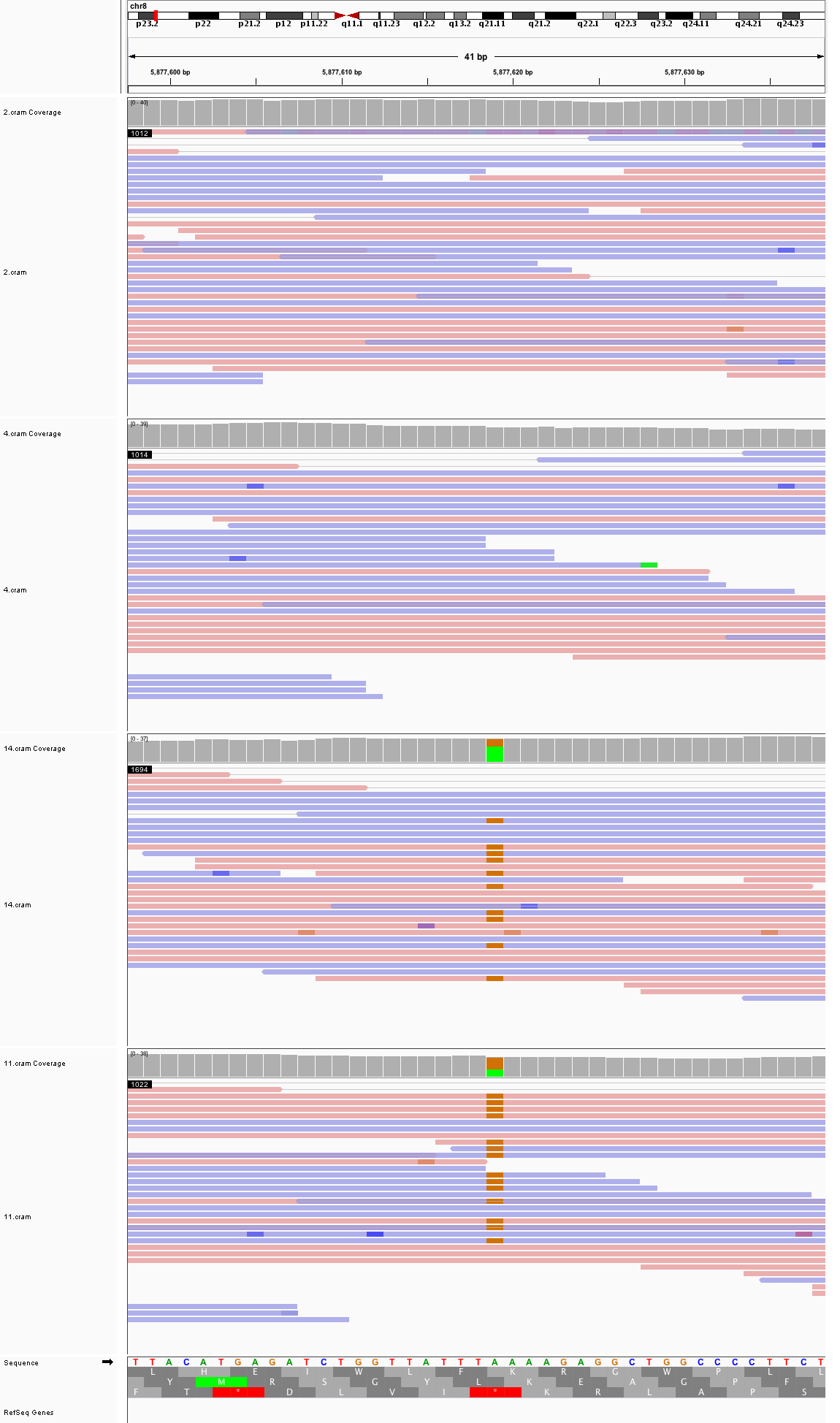

Supplement: Supplementary file 4. — All tracks below contain alignments from the third-generation children that share a DNM at the site. Reads with mapping quality <20 are filtered out, as they were not considered by our variant calling pipeline, and mismatched bases are shaded by quality score (more transparent = lower base quality). [file elife-46922-supp4.zip › supp_file_4/chr8_5,877,598_5,877,638.png]

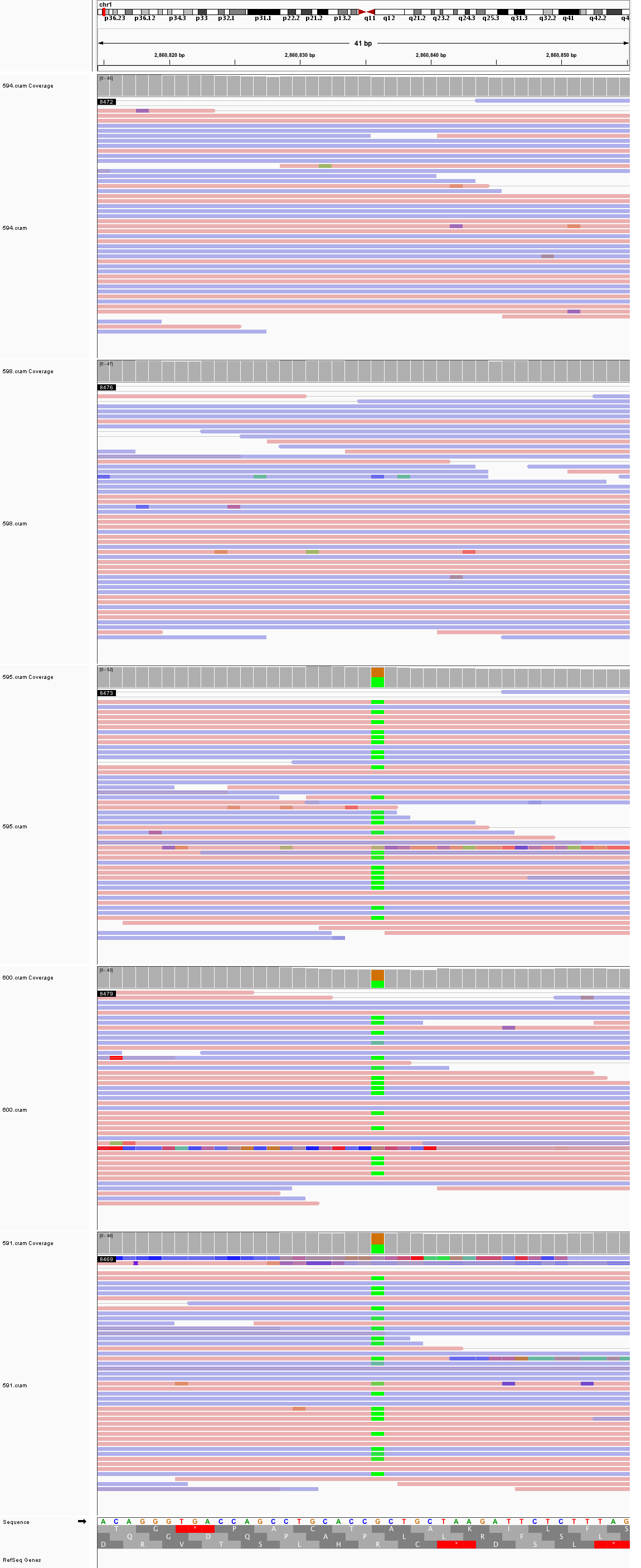

Supplement: Supplementary file 4. — All tracks below contain alignments from the third-generation children that share a DNM at the site. Reads with mapping quality <20 are filtered out, as they were not considered by our variant calling pipeline, and mismatched bases are shaded by quality score (more transparent = lower base quality). [file elife-46922-supp4.zip › supp_file_4/chr1_2,860,815_2,860,855.png]

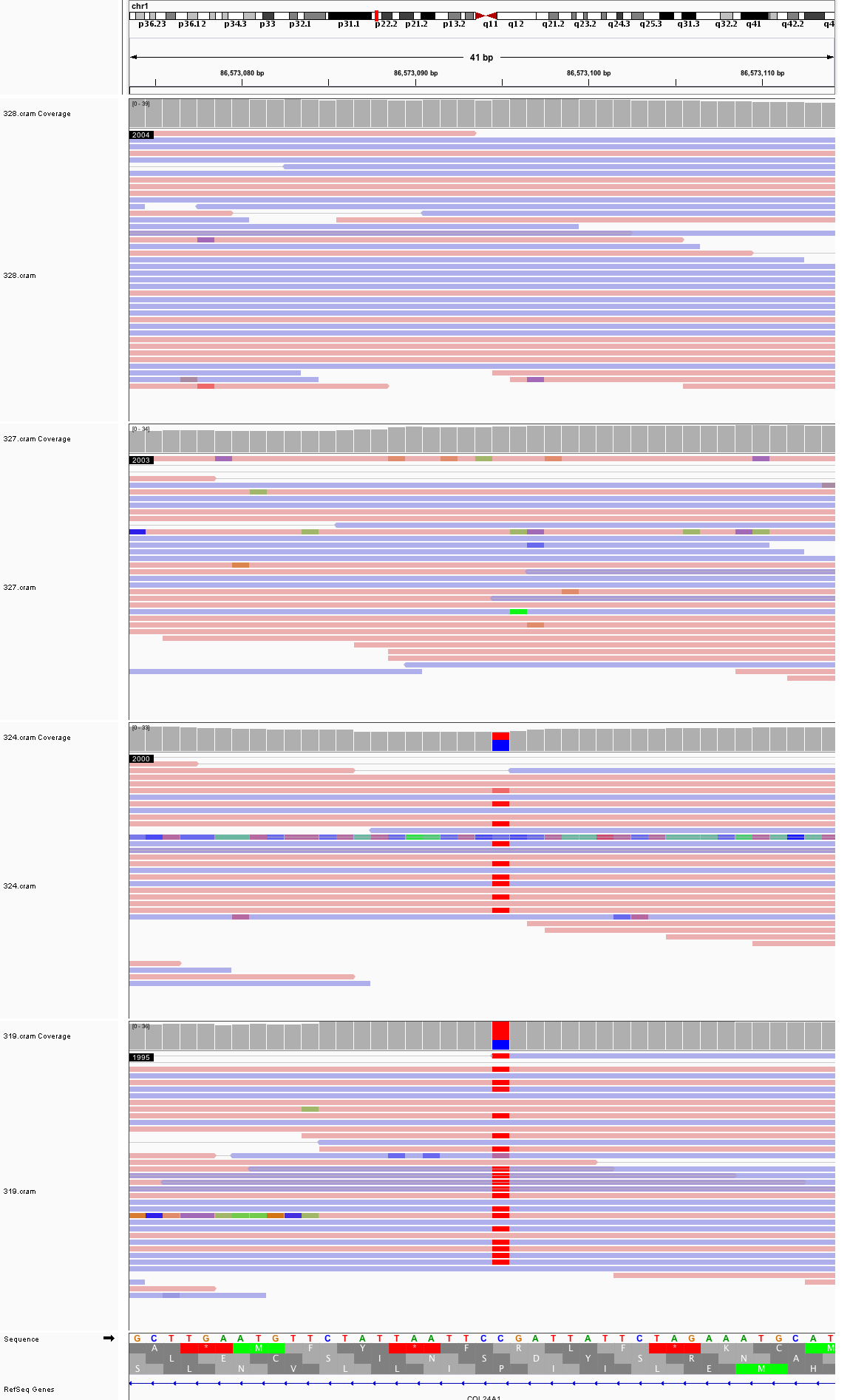

Supplement: Supplementary file 4. — All tracks below contain alignments from the third-generation children that share a DNM at the site. Reads with mapping quality <20 are filtered out, as they were not considered by our variant calling pipeline, and mismatched bases are shaded by quality score (more transparent = lower base quality). [file elife-46922-supp4.zip › supp_file_4/chr1_86,573,074_86,573,114.png]

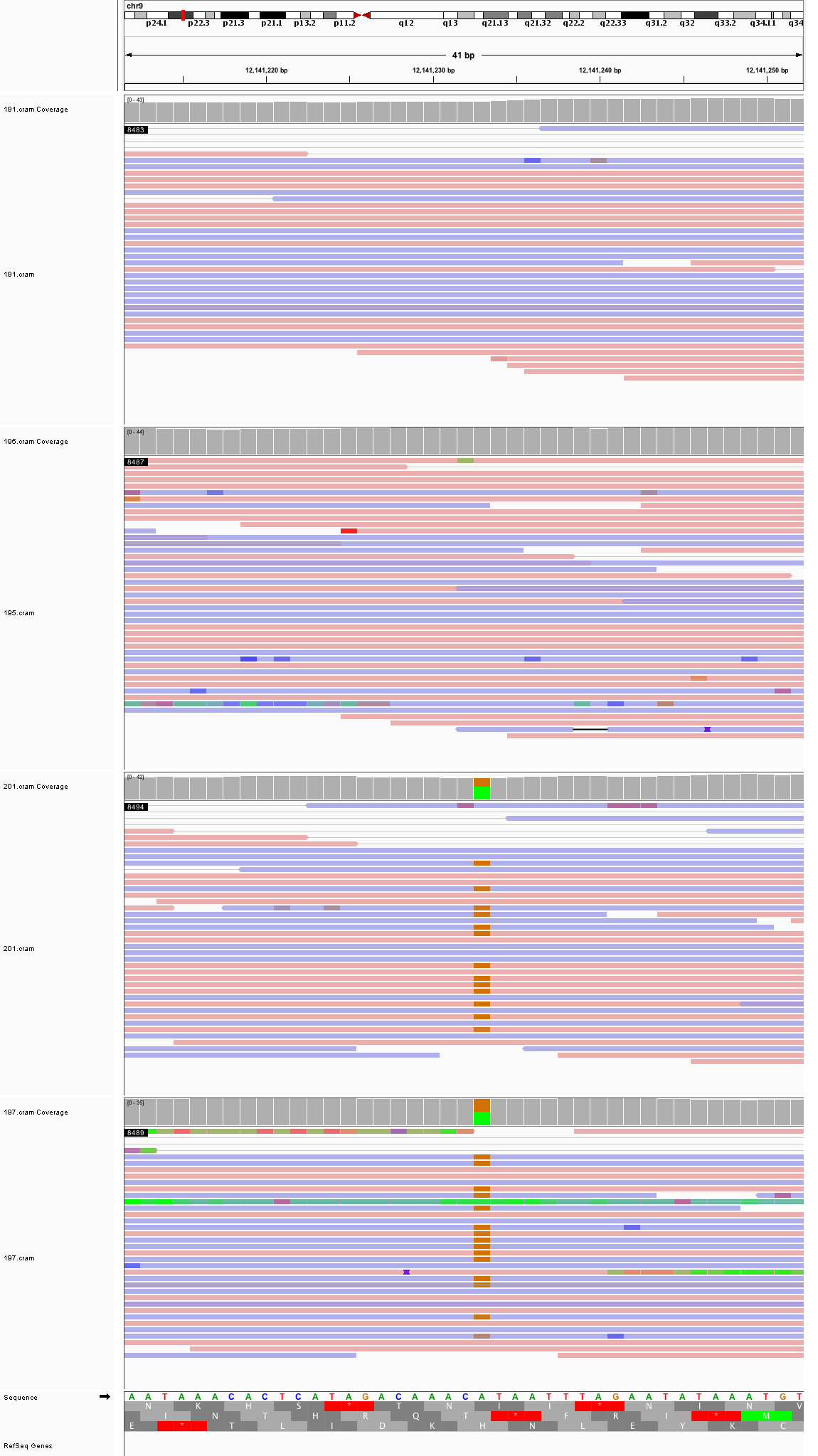

Supplement: Supplementary file 4. — All tracks below contain alignments from the third-generation children that share a DNM at the site. Reads with mapping quality <20 are filtered out, as they were not considered by our variant calling pipeline, and mismatched bases are shaded by quality score (more transparent = lower base quality). [file elife-46922-supp4.zip › supp_file_4/chr9_12,141,212_12,141,252.png]

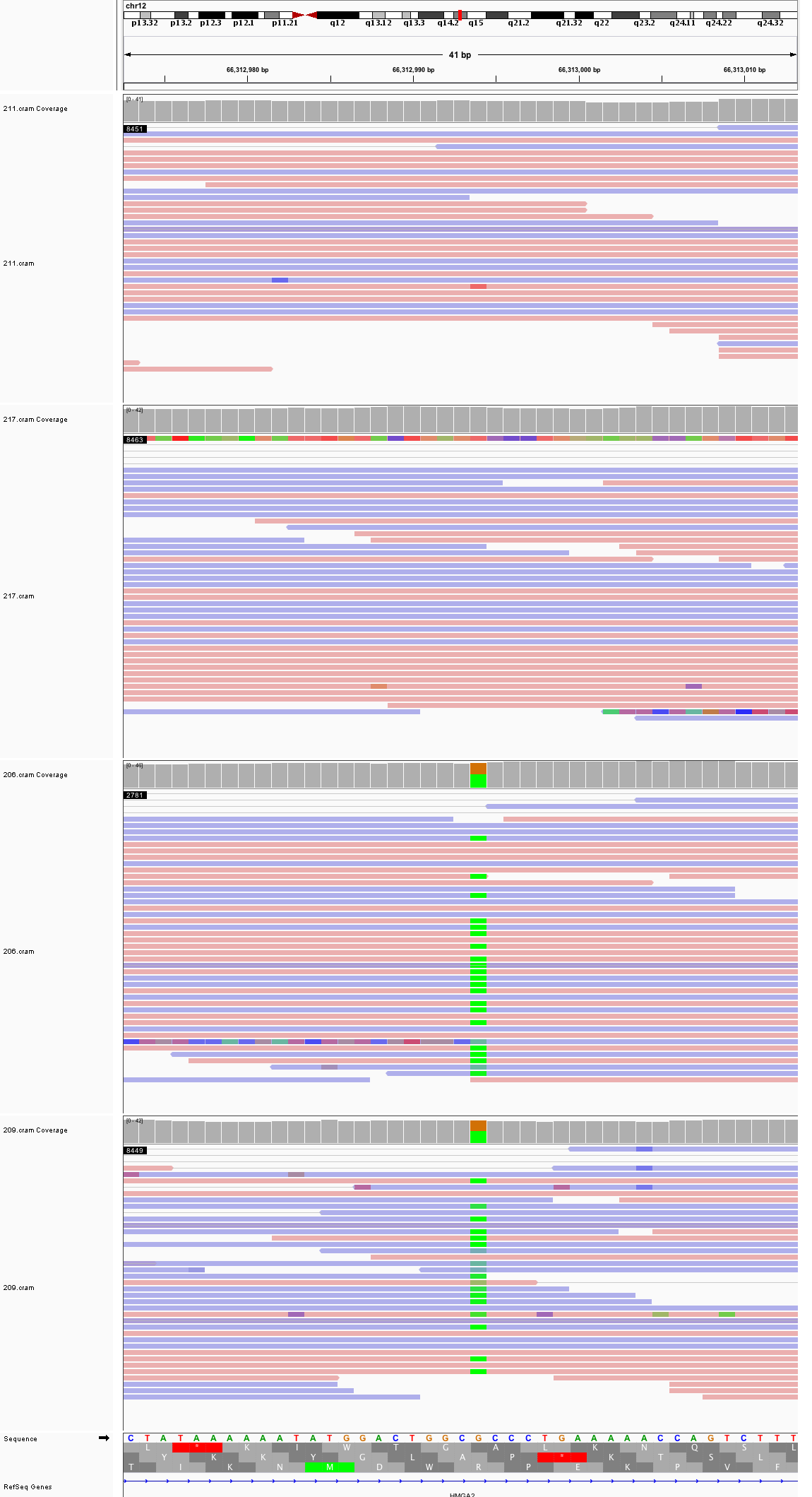

Supplement: Supplementary file 4. — All tracks below contain alignments from the third-generation children that share a DNM at the site. Reads with mapping quality <20 are filtered out, as they were not considered by our variant calling pipeline, and mismatched bases are shaded by quality score (more transparent = lower base quality). [file elife-46922-supp4.zip › supp_file_4/chr12_66,312,973_66,313,013.png]

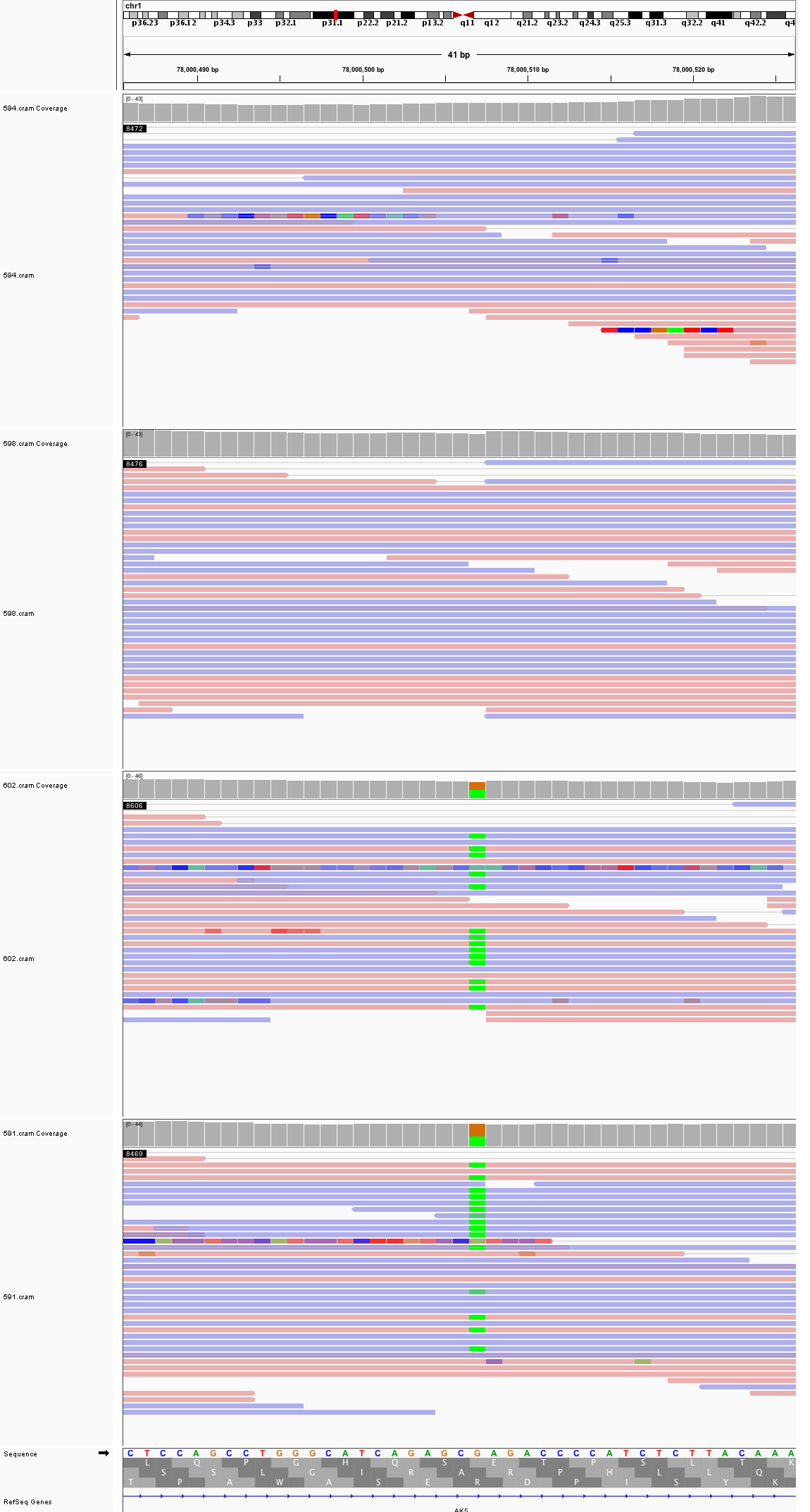

Supplement: Supplementary file 4. — All tracks below contain alignments from the third-generation children that share a DNM at the site. Reads with mapping quality <20 are filtered out, as they were not considered by our variant calling pipeline, and mismatched bases are shaded by quality score (more transparent = lower base quality). [file elife-46922-supp4.zip › supp_file_4/chr1_78,000,486_78,000,526.png]

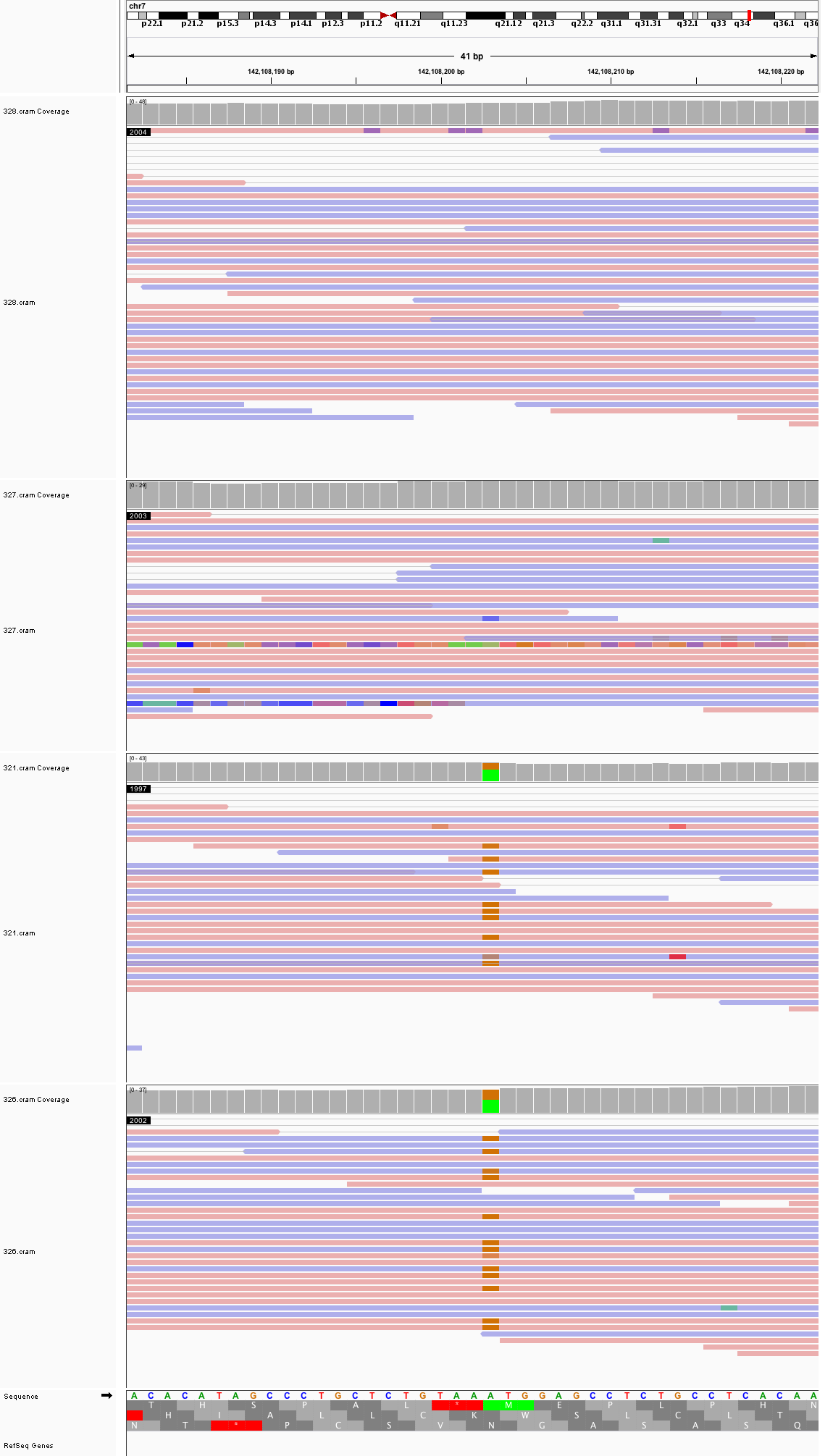

Supplement: Supplementary file 4. — All tracks below contain alignments from the third-generation children that share a DNM at the site. Reads with mapping quality <20 are filtered out, as they were not considered by our variant calling pipeline, and mismatched bases are shaded by quality score (more transparent = lower base quality). [file elife-46922-supp4.zip › supp_file_4/chr7_142,108,182_142,108,222.png]

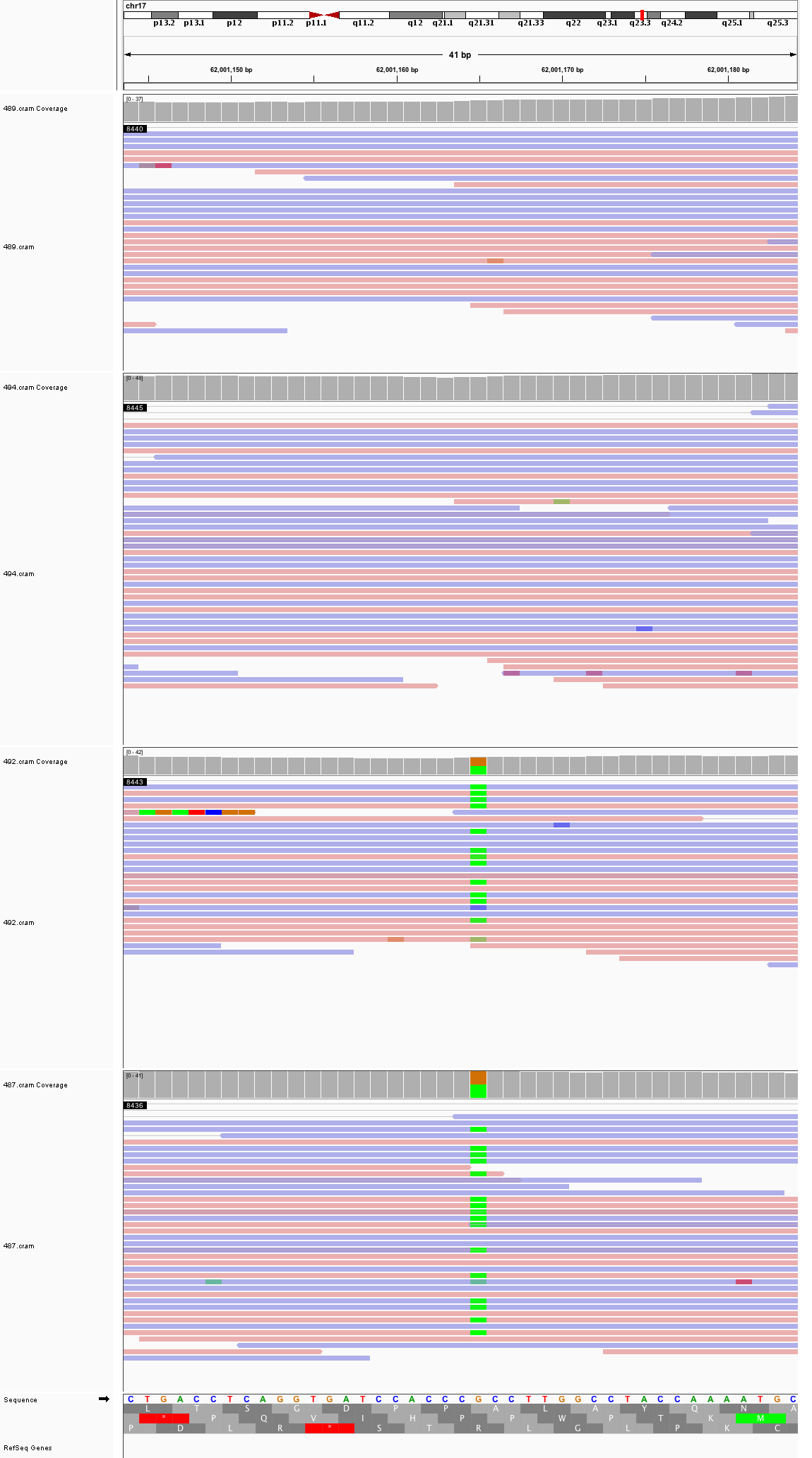

Supplement: Supplementary file 4. — All tracks below contain alignments from the third-generation children that share a DNM at the site. Reads with mapping quality <20 are filtered out, as they were not considered by our variant calling pipeline, and mismatched bases are shaded by quality score (more transparent = lower base quality). [file elife-46922-supp4.zip › supp_file_4/chr17_62,001,144_62,001,184.png]

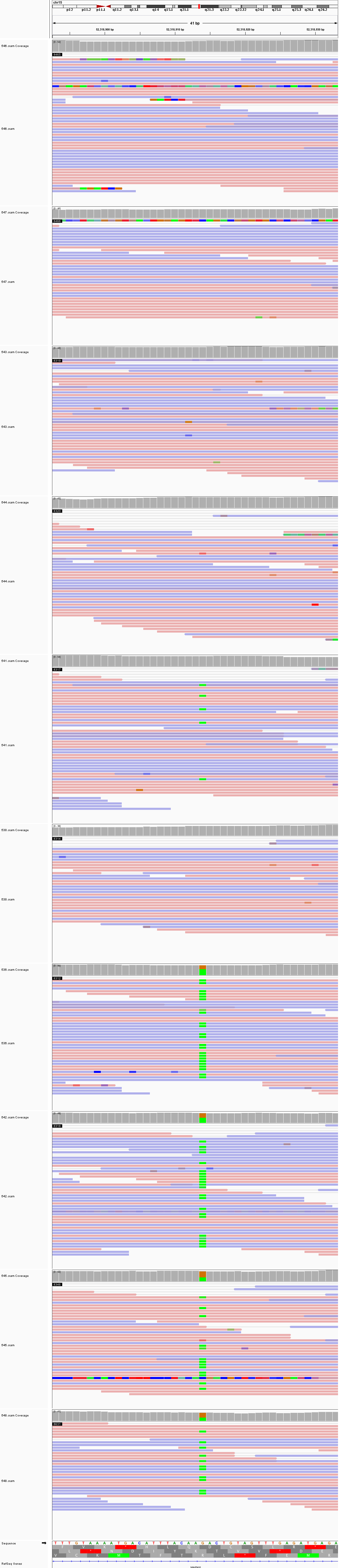

Supplement: Supplementary file 5. — In each image, the first two, three, or four tracks contain alignments from the grandparents in the pedigree (i.e., paternal grandmother and grandfather, maternal grandmother and grandfather). In some families, one or two of the first-generation grandparents were not sequenced (see Supplementary file 1). The two tracks below contain alignments from the second-generation individual with the putative gonosomal mutation and that second-generation individual’s spouse. The remaining tracks below contain alignments from the third-generation individuals that inherited the gonosomal mutation. Reads with mapping quality <20 are filtered out, as they were not considered by our variant calling pipeline, and mismatched bases are shaded by quality score (more transparent = lower base quality). [file elife-46922-supp5.zip › supp_file_5/chr15_52,318,893_52,318,933.png]

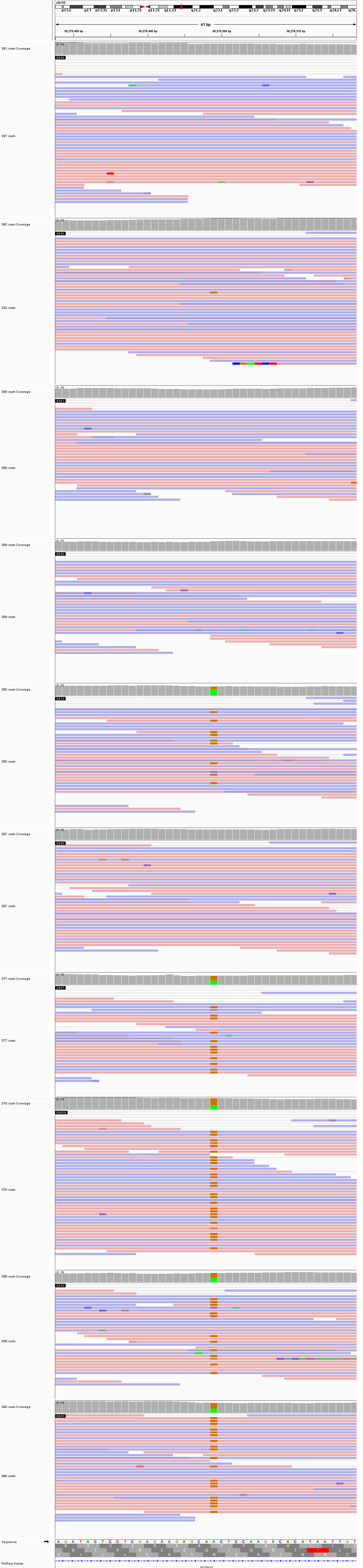

Supplement: Supplementary file 5. — In each image, the first two, three, or four tracks contain alignments from the grandparents in the pedigree (i.e., paternal grandmother and grandfather, maternal grandmother and grandfather). In some families, one or two of the first-generation grandparents were not sequenced (see Supplementary file 1). The two tracks below contain alignments from the second-generation individual with the putative gonosomal mutation and that second-generation individual’s spouse. The remaining tracks below contain alignments from the third-generation individuals that inherited the gonosomal mutation. Reads with mapping quality <20 are filtered out, as they were not considered by our variant calling pipeline, and mismatched bases are shaded by quality score (more transparent = lower base quality). [file elife-46922-supp5.zip › supp_file_5/chr10_56,276,478_56,276,518.png]

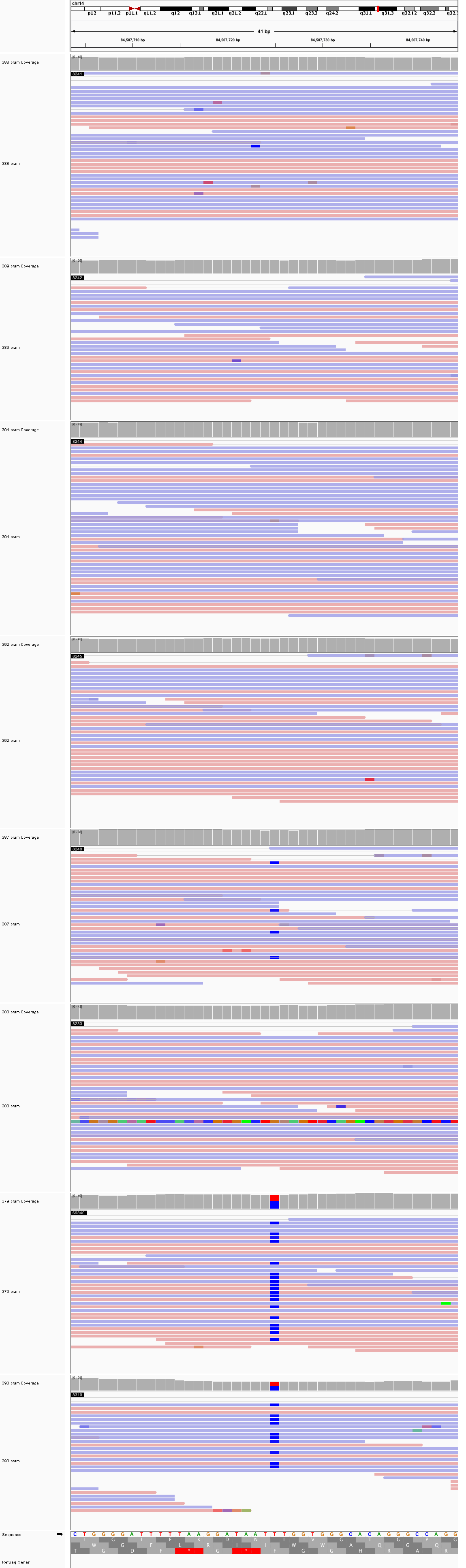

Supplement: Supplementary file 5. — In each image, the first two, three, or four tracks contain alignments from the grandparents in the pedigree (i.e., paternal grandmother and grandfather, maternal grandmother and grandfather). In some families, one or two of the first-generation grandparents were not sequenced (see Supplementary file 1). The two tracks below contain alignments from the second-generation individual with the putative gonosomal mutation and that second-generation individual’s spouse. The remaining tracks below contain alignments from the third-generation individuals that inherited the gonosomal mutation. Reads with mapping quality <20 are filtered out, as they were not considered by our variant calling pipeline, and mismatched bases are shaded by quality score (more transparent = lower base quality). [file elife-46922-supp5.zip › supp_file_5/chr14_84,507,704_84,507,744.png]

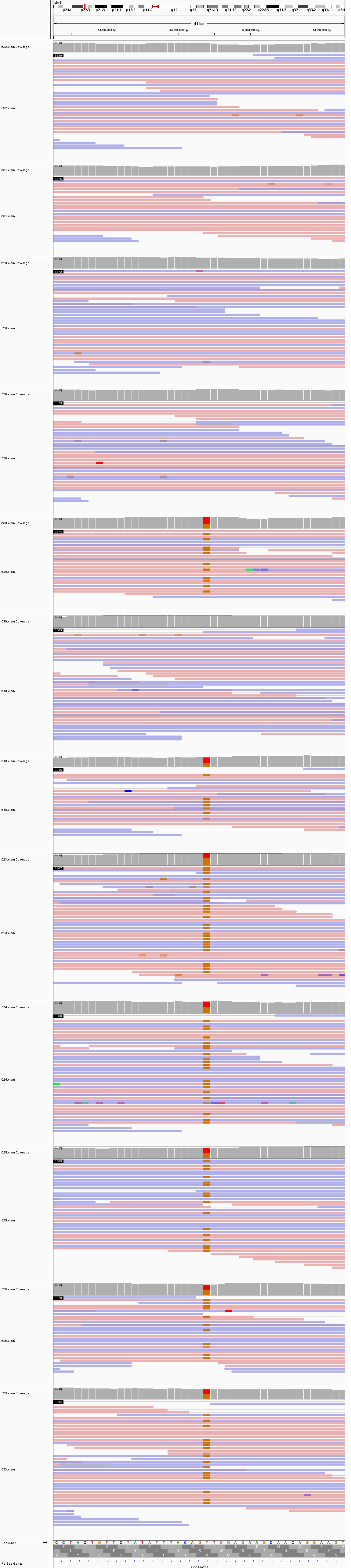

Supplement: Supplementary file 5. — In each image, the first two, three, or four tracks contain alignments from the grandparents in the pedigree (i.e., paternal grandmother and grandfather, maternal grandmother and grandfather). In some families, one or two of the first-generation grandparents were not sequenced (see Supplementary file 1). The two tracks below contain alignments from the second-generation individual with the putative gonosomal mutation and that second-generation individual’s spouse. The remaining tracks below contain alignments from the third-generation individuals that inherited the gonosomal mutation. Reads with mapping quality <20 are filtered out, as they were not considered by our variant calling pipeline, and mismatched bases are shaded by quality score (more transparent = lower base quality). [file elife-46922-supp5.zip › supp_file_5/chr9_14,994,863_14,994,903.png]

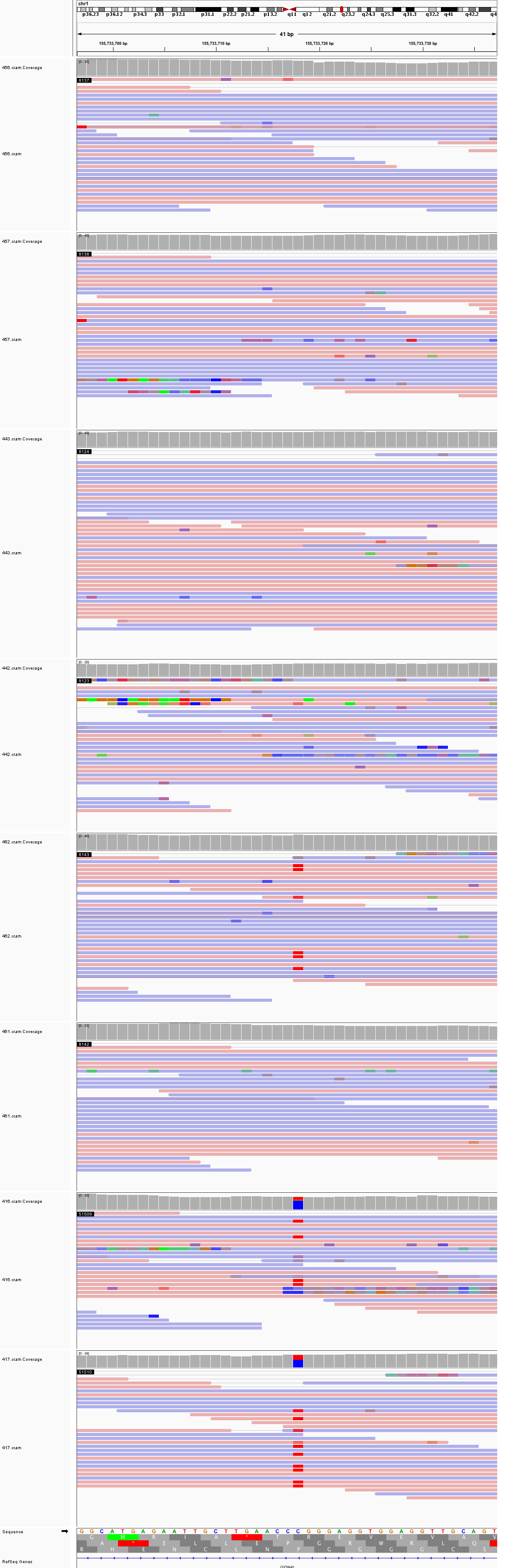

Supplement: Supplementary file 5. — In each image, the first two, three, or four tracks contain alignments from the grandparents in the pedigree (i.e., paternal grandmother and grandfather, maternal grandmother and grandfather). In some families, one or two of the first-generation grandparents were not sequenced (see Supplementary file 1). The two tracks below contain alignments from the second-generation individual with the putative gonosomal mutation and that second-generation individual’s spouse. The remaining tracks below contain alignments from the third-generation individuals that inherited the gonosomal mutation. Reads with mapping quality <20 are filtered out, as they were not considered by our variant calling pipeline, and mismatched bases are shaded by quality score (more transparent = lower base quality). [file elife-46922-supp5.zip › supp_file_5/chr1_155,733,697_155,733,737.png]

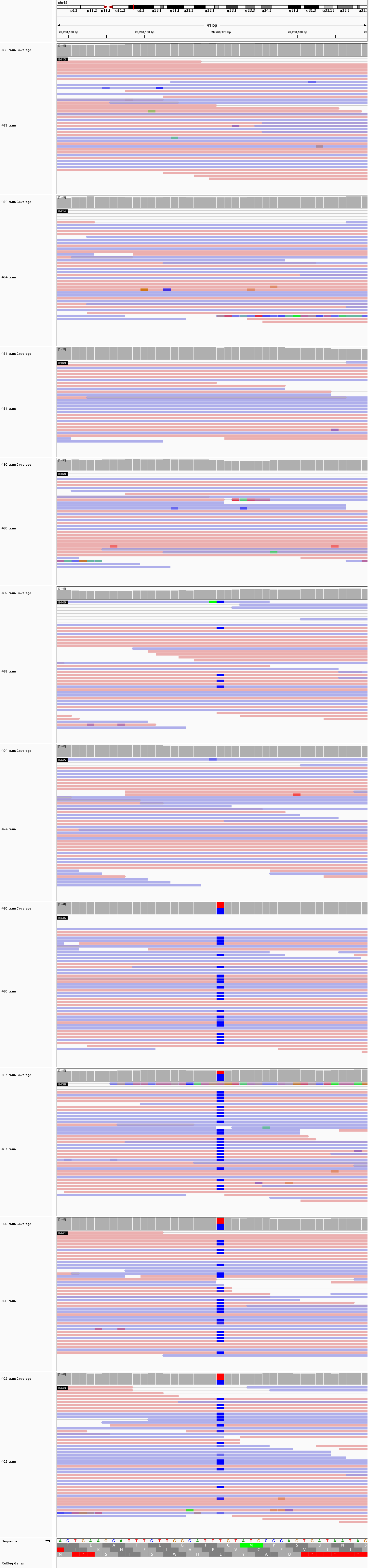

Supplement: Supplementary file 5. — In each image, the first two, three, or four tracks contain alignments from the grandparents in the pedigree (i.e., paternal grandmother and grandfather, maternal grandmother and grandfather). In some families, one or two of the first-generation grandparents were not sequenced (see Supplementary file 1). The two tracks below contain alignments from the second-generation individual with the putative gonosomal mutation and that second-generation individual’s spouse. The remaining tracks below contain alignments from the third-generation individuals that inherited the gonosomal mutation. Reads with mapping quality <20 are filtered out, as they were not considered by our variant calling pipeline, and mismatched bases are shaded by quality score (more transparent = lower base quality). [file elife-46922-supp5.zip › supp_file_5/chr14_26,268,149_26,268,189.png]

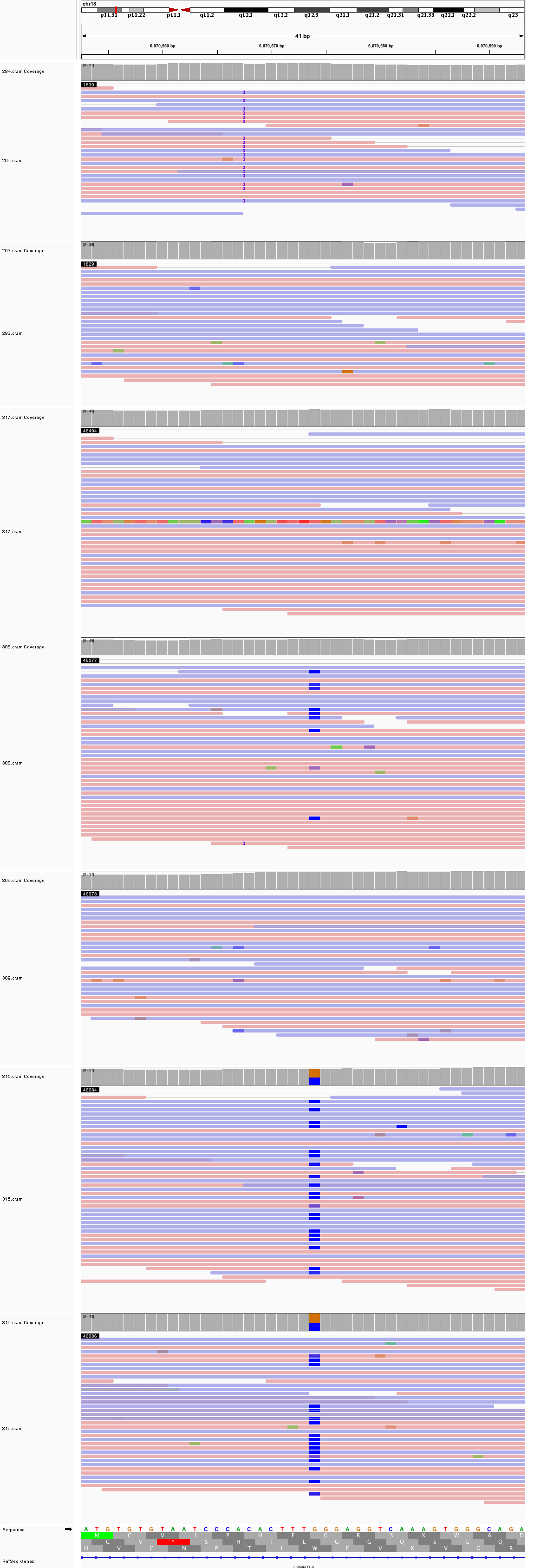

Supplement: Supplementary file 5. — In each image, the first two, three, or four tracks contain alignments from the grandparents in the pedigree (i.e., paternal grandmother and grandfather, maternal grandmother and grandfather). In some families, one or two of the first-generation grandparents were not sequenced (see Supplementary file 1). The two tracks below contain alignments from the second-generation individual with the putative gonosomal mutation and that second-generation individual’s spouse. The remaining tracks below contain alignments from the third-generation individuals that inherited the gonosomal mutation. Reads with mapping quality <20 are filtered out, as they were not considered by our variant calling pipeline, and mismatched bases are shaded by quality score (more transparent = lower base quality). [file elife-46922-supp5.zip › supp_file_5/chr18_6,070,553_6,070,593.png]

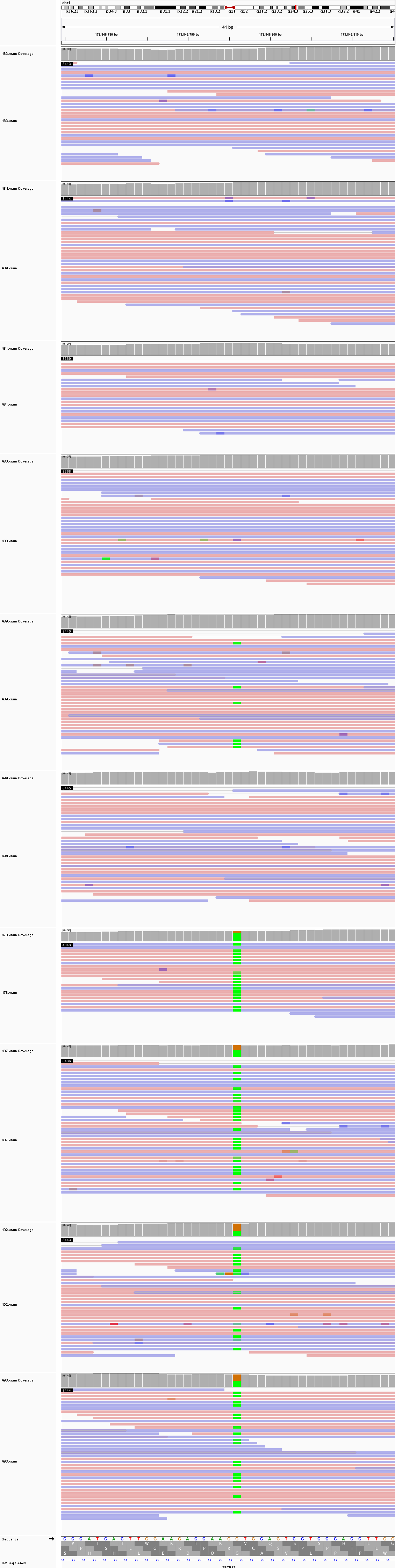

Supplement: Supplementary file 5. — In each image, the first two, three, or four tracks contain alignments from the grandparents in the pedigree (i.e., paternal grandmother and grandfather, maternal grandmother and grandfather). In some families, one or two of the first-generation grandparents were not sequenced (see Supplementary file 1). The two tracks below contain alignments from the second-generation individual with the putative gonosomal mutation and that second-generation individual’s spouse. The remaining tracks below contain alignments from the third-generation individuals that inherited the gonosomal mutation. Reads with mapping quality <20 are filtered out, as they were not considered by our variant calling pipeline, and mismatched bases are shaded by quality score (more transparent = lower base quality). [file elife-46922-supp5.zip › supp_file_5/chr1_173,846,775_173,846,815.png]

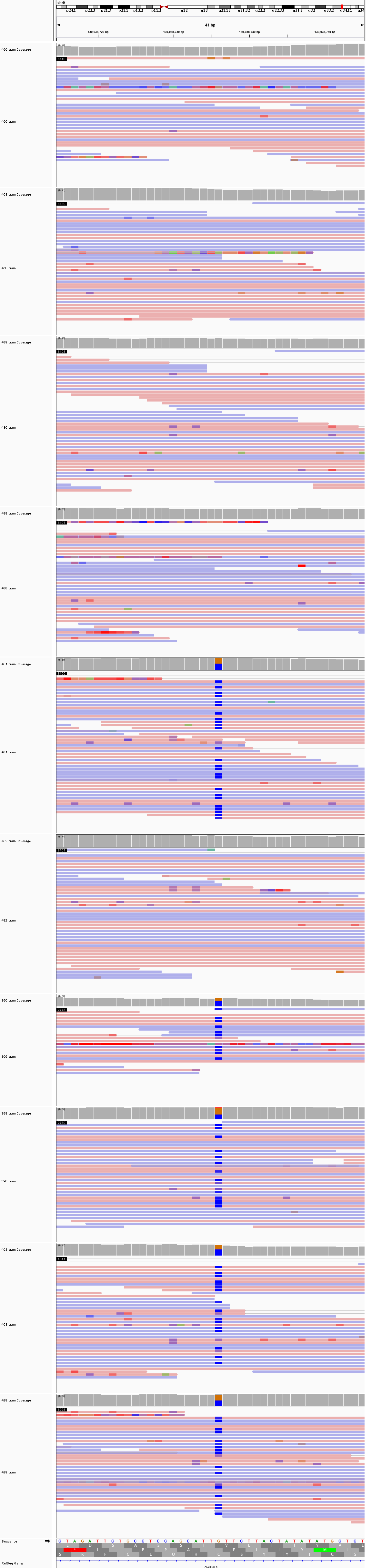

Supplement: Supplementary file 5. — In each image, the first two, three, or four tracks contain alignments from the grandparents in the pedigree (i.e., paternal grandmother and grandfather, maternal grandmother and grandfather). In some families, one or two of the first-generation grandparents were not sequenced (see Supplementary file 1). The two tracks below contain alignments from the second-generation individual with the putative gonosomal mutation and that second-generation individual’s spouse. The remaining tracks below contain alignments from the third-generation individuals that inherited the gonosomal mutation. Reads with mapping quality <20 are filtered out, as they were not considered by our variant calling pipeline, and mismatched bases are shaded by quality score (more transparent = lower base quality). [file elife-46922-supp5.zip › supp_file_5/chr9_130,038,715_130,038,755.png]

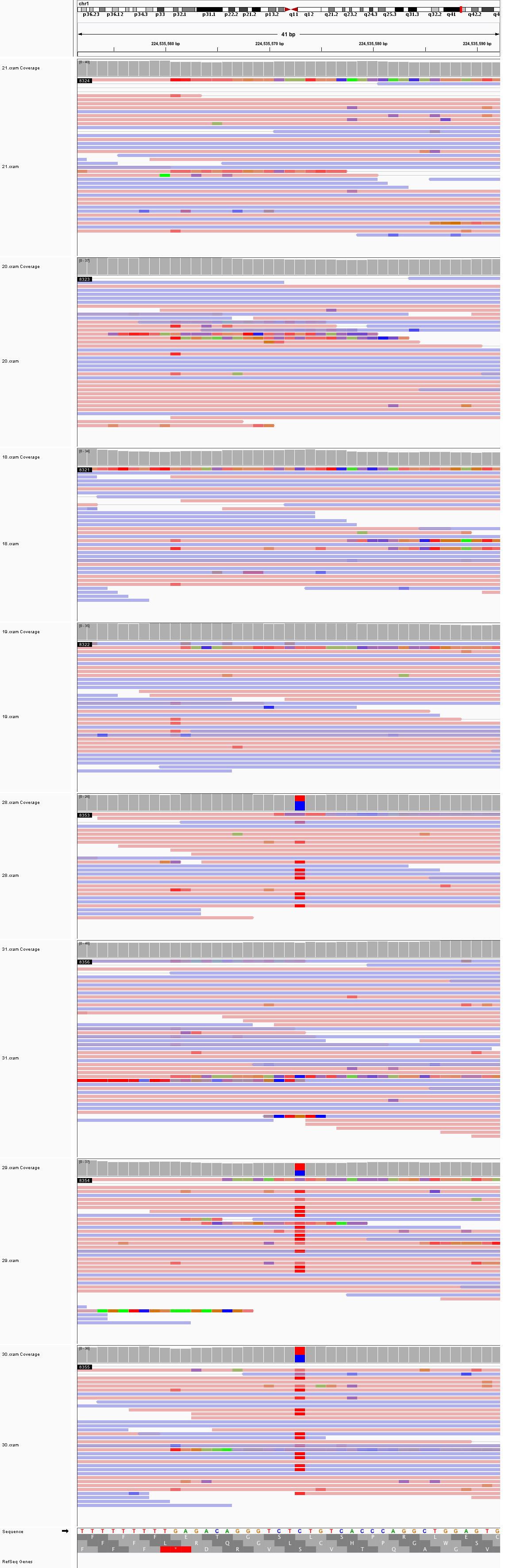

Supplement: Supplementary file 5. — In each image, the first two, three, or four tracks contain alignments from the grandparents in the pedigree (i.e., paternal grandmother and grandfather, maternal grandmother and grandfather). In some families, one or two of the first-generation grandparents were not sequenced (see Supplementary file 1). The two tracks below contain alignments from the second-generation individual with the putative gonosomal mutation and that second-generation individual’s spouse. The remaining tracks below contain alignments from the third-generation individuals that inherited the gonosomal mutation. Reads with mapping quality <20 are filtered out, as they were not considered by our variant calling pipeline, and mismatched bases are shaded by quality score (more transparent = lower base quality). [file elife-46922-supp5.zip › supp_file_5/chr1_224,535,552_224,535,592.png]

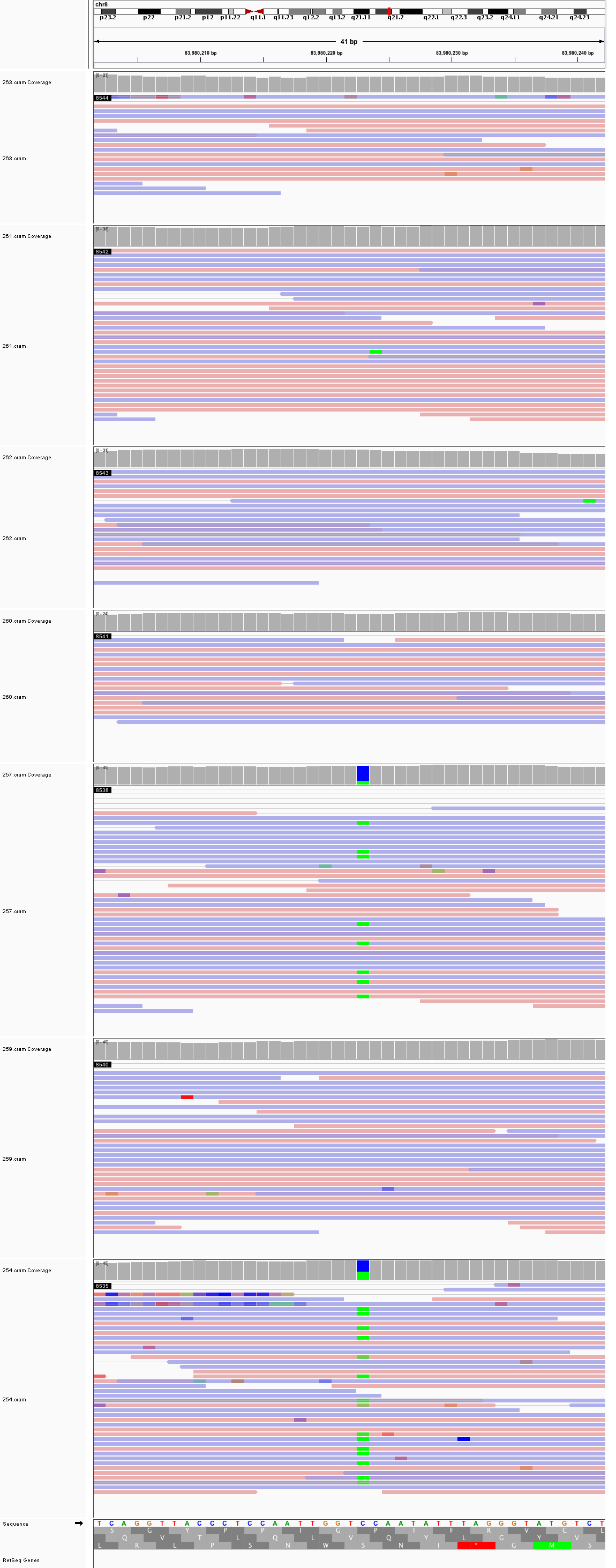

Supplement: Supplementary file 5. — In each image, the first two, three, or four tracks contain alignments from the grandparents in the pedigree (i.e., paternal grandmother and grandfather, maternal grandmother and grandfather). In some families, one or two of the first-generation grandparents were not sequenced (see Supplementary file 1). The two tracks below contain alignments from the second-generation individual with the putative gonosomal mutation and that second-generation individual’s spouse. The remaining tracks below contain alignments from the third-generation individuals that inherited the gonosomal mutation. Reads with mapping quality <20 are filtered out, as they were not considered by our variant calling pipeline, and mismatched bases are shaded by quality score (more transparent = lower base quality). [file elife-46922-supp5.zip › supp_file_5/chr8_83,980,202_83,980,242.png]

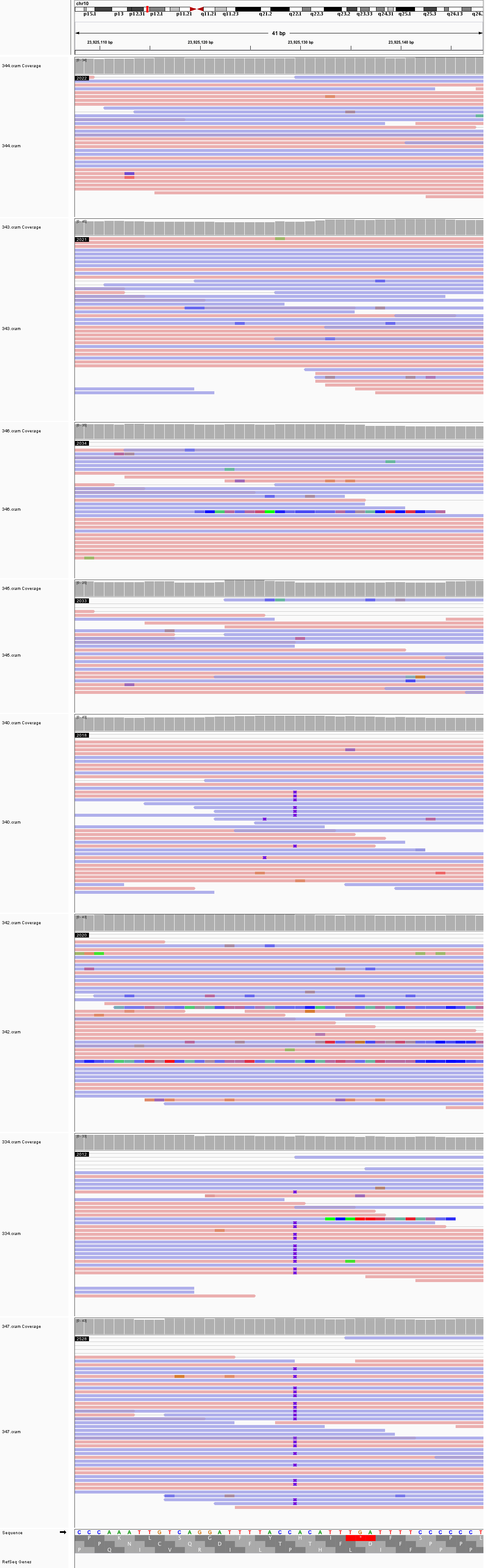

Supplement: Supplementary file 5. — In each image, the first two, three, or four tracks contain alignments from the grandparents in the pedigree (i.e., paternal grandmother and grandfather, maternal grandmother and grandfather). In some families, one or two of the first-generation grandparents were not sequenced (see Supplementary file 1). The two tracks below contain alignments from the second-generation individual with the putative gonosomal mutation and that second-generation individual’s spouse. The remaining tracks below contain alignments from the third-generation individuals that inherited the gonosomal mutation. Reads with mapping quality <20 are filtered out, as they were not considered by our variant calling pipeline, and mismatched bases are shaded by quality score (more transparent = lower base quality). [file elife-46922-supp5.zip › supp_file_5/chr10_23,925,108_23,925,148.png]

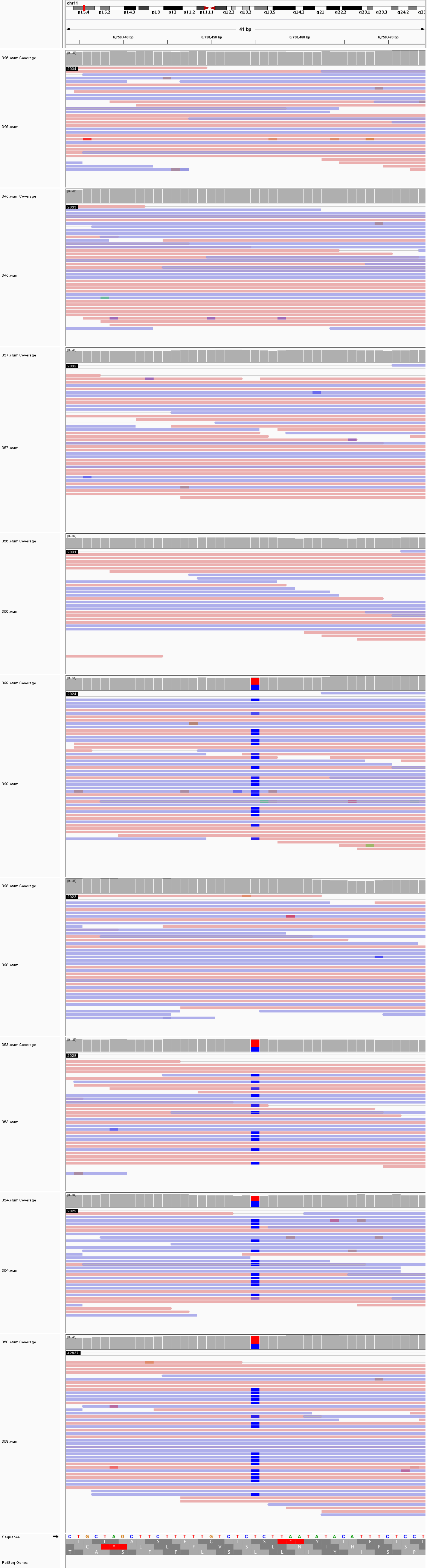

Supplement: Supplementary file 5. — In each image, the first two, three, or four tracks contain alignments from the grandparents in the pedigree (i.e., paternal grandmother and grandfather, maternal grandmother and grandfather). In some families, one or two of the first-generation grandparents were not sequenced (see Supplementary file 1). The two tracks below contain alignments from the second-generation individual with the putative gonosomal mutation and that second-generation individual’s spouse. The remaining tracks below contain alignments from the third-generation individuals that inherited the gonosomal mutation. Reads with mapping quality <20 are filtered out, as they were not considered by our variant calling pipeline, and mismatched bases are shaded by quality score (more transparent = lower base quality). [file elife-46922-supp5.zip › supp_file_5/chr11_6,750,434_6,750,474.png]

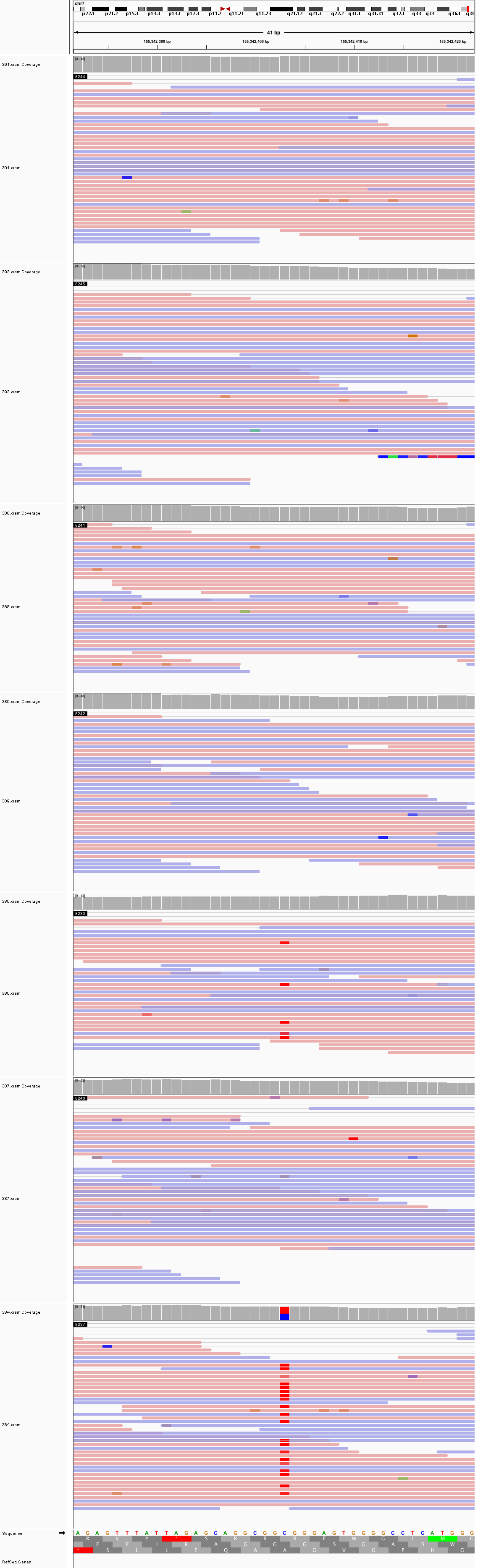

Supplement: Supplementary file 5. — In each image, the first two, three, or four tracks contain alignments from the grandparents in the pedigree (i.e., paternal grandmother and grandfather, maternal grandmother and grandfather). In some families, one or two of the first-generation grandparents were not sequenced (see Supplementary file 1). The two tracks below contain alignments from the second-generation individual with the putative gonosomal mutation and that second-generation individual’s spouse. The remaining tracks below contain alignments from the third-generation individuals that inherited the gonosomal mutation. Reads with mapping quality <20 are filtered out, as they were not considered by our variant calling pipeline, and mismatched bases are shaded by quality score (more transparent = lower base quality). [file elife-46922-supp5.zip › supp_file_5/chr7_155,342,382_155,342,422.png]

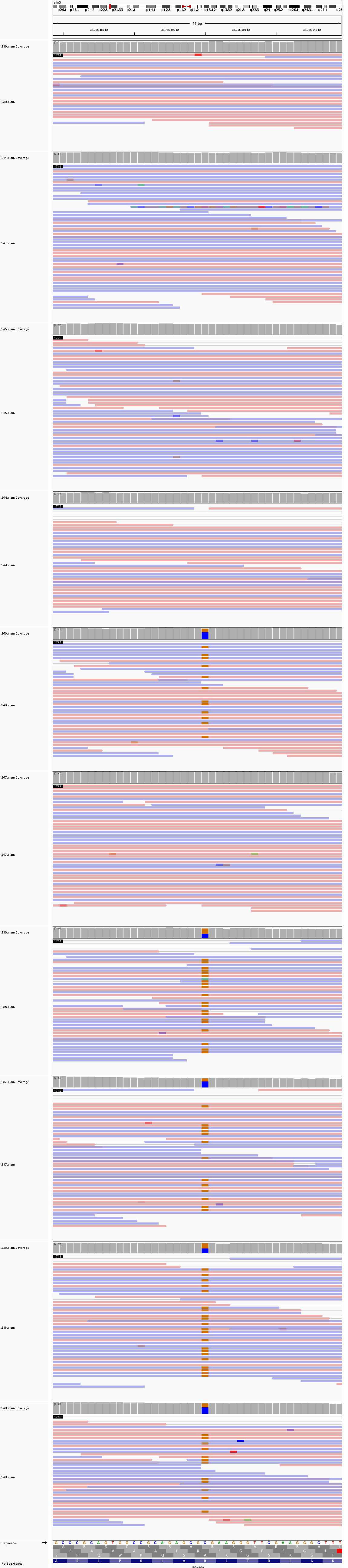

Supplement: Supplementary file 5. — In each image, the first two, three, or four tracks contain alignments from the grandparents in the pedigree (i.e., paternal grandmother and grandfather, maternal grandmother and grandfather). In some families, one or two of the first-generation grandparents were not sequenced (see Supplementary file 1). The two tracks below contain alignments from the second-generation individual with the putative gonosomal mutation and that second-generation individual’s spouse. The remaining tracks below contain alignments from the third-generation individuals that inherited the gonosomal mutation. Reads with mapping quality <20 are filtered out, as they were not considered by our variant calling pipeline, and mismatched bases are shaded by quality score (more transparent = lower base quality). [file elife-46922-supp5.zip › supp_file_5/chr3_38,755,474_38,755,514.png]

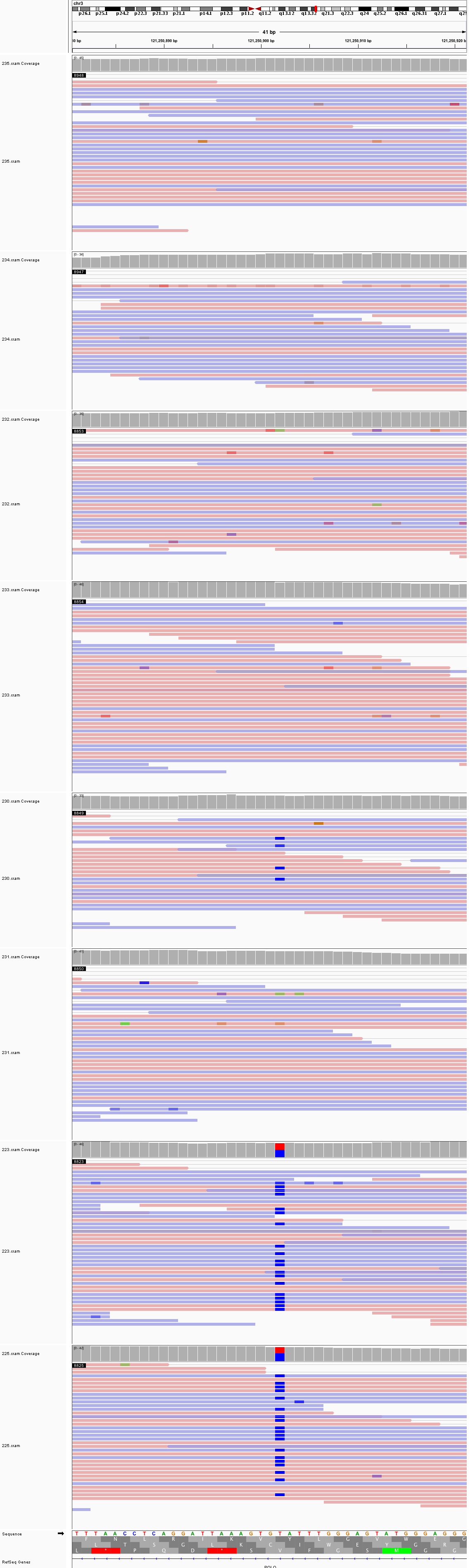

Supplement: Supplementary file 5. — In each image, the first two, three, or four tracks contain alignments from the grandparents in the pedigree (i.e., paternal grandmother and grandfather, maternal grandmother and grandfather). In some families, one or two of the first-generation grandparents were not sequenced (see Supplementary file 1). The two tracks below contain alignments from the second-generation individual with the putative gonosomal mutation and that second-generation individual’s spouse. The remaining tracks below contain alignments from the third-generation individuals that inherited the gonosomal mutation. Reads with mapping quality <20 are filtered out, as they were not considered by our variant calling pipeline, and mismatched bases are shaded by quality score (more transparent = lower base quality). [file elife-46922-supp5.zip › supp_file_5/chr3_121,250,881_121,250,921.png]

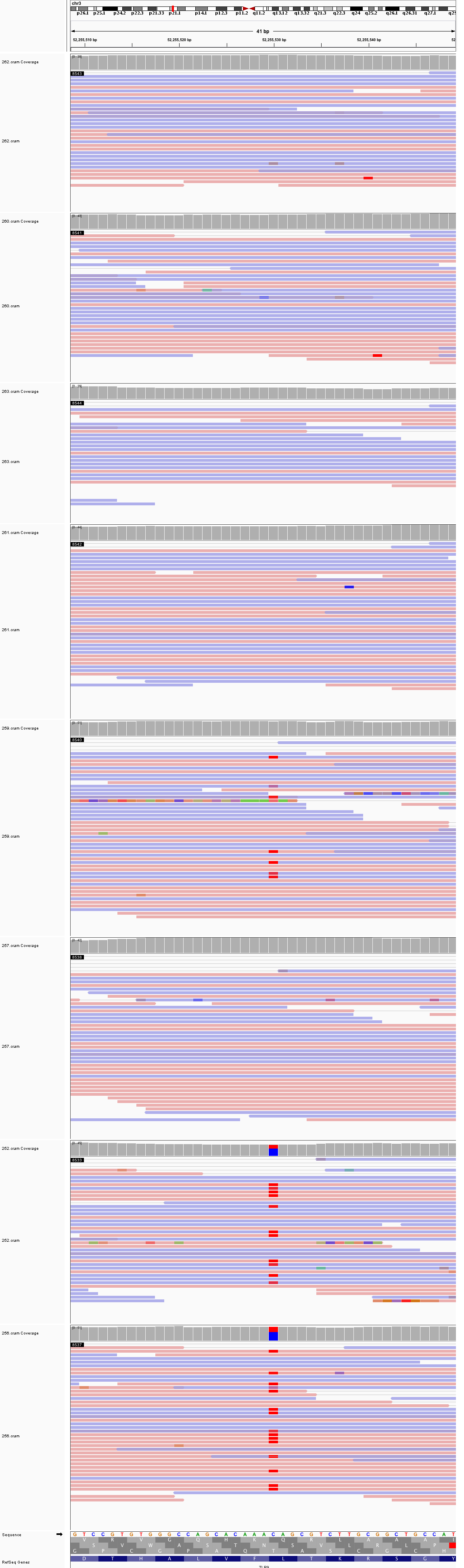

Supplement: Supplementary file 5. — In each image, the first two, three, or four tracks contain alignments from the grandparents in the pedigree (i.e., paternal grandmother and grandfather, maternal grandmother and grandfather). In some families, one or two of the first-generation grandparents were not sequenced (see Supplementary file 1). The two tracks below contain alignments from the second-generation individual with the putative gonosomal mutation and that second-generation individual’s spouse. The remaining tracks below contain alignments from the third-generation individuals that inherited the gonosomal mutation. Reads with mapping quality <20 are filtered out, as they were not considered by our variant calling pipeline, and mismatched bases are shaded by quality score (more transparent = lower base quality). [file elife-46922-supp5.zip › supp_file_5/chr3_52,255,509_52,255,549.png]

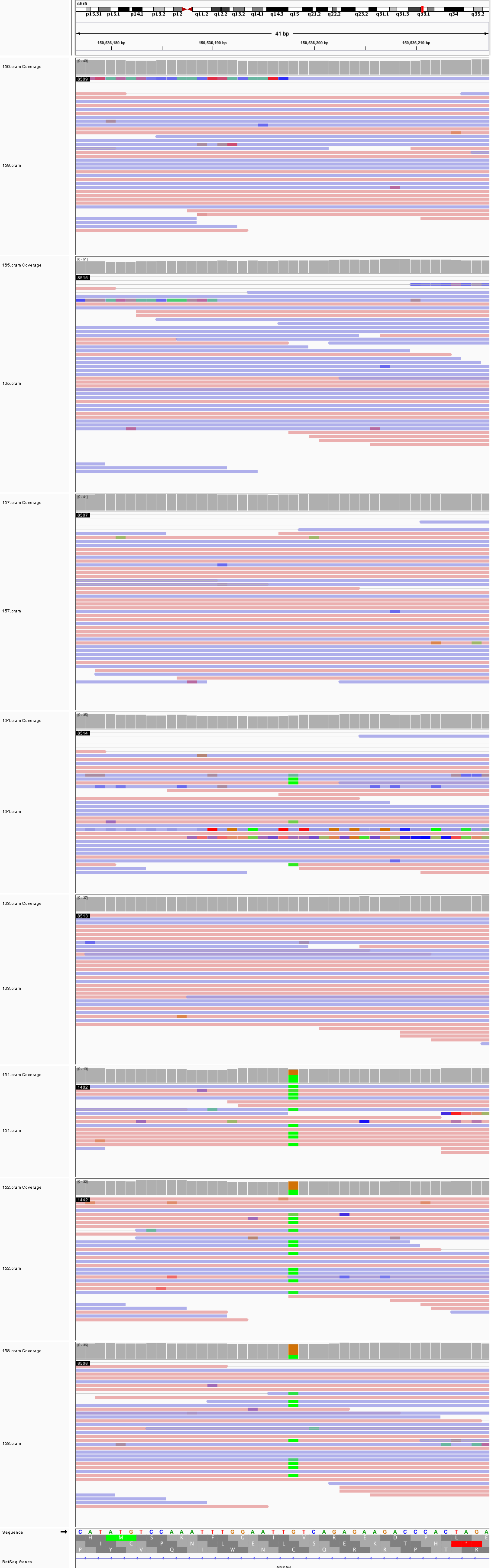

Supplement: Supplementary file 5. — In each image, the first two, three, or four tracks contain alignments from the grandparents in the pedigree (i.e., paternal grandmother and grandfather, maternal grandmother and grandfather). In some families, one or two of the first-generation grandparents were not sequenced (see Supplementary file 1). The two tracks below contain alignments from the second-generation individual with the putative gonosomal mutation and that second-generation individual’s spouse. The remaining tracks below contain alignments from the third-generation individuals that inherited the gonosomal mutation. Reads with mapping quality <20 are filtered out, as they were not considered by our variant calling pipeline, and mismatched bases are shaded by quality score (more transparent = lower base quality). [file elife-46922-supp5.zip › supp_file_5/chr5_150,536,177_150,536,217.png]

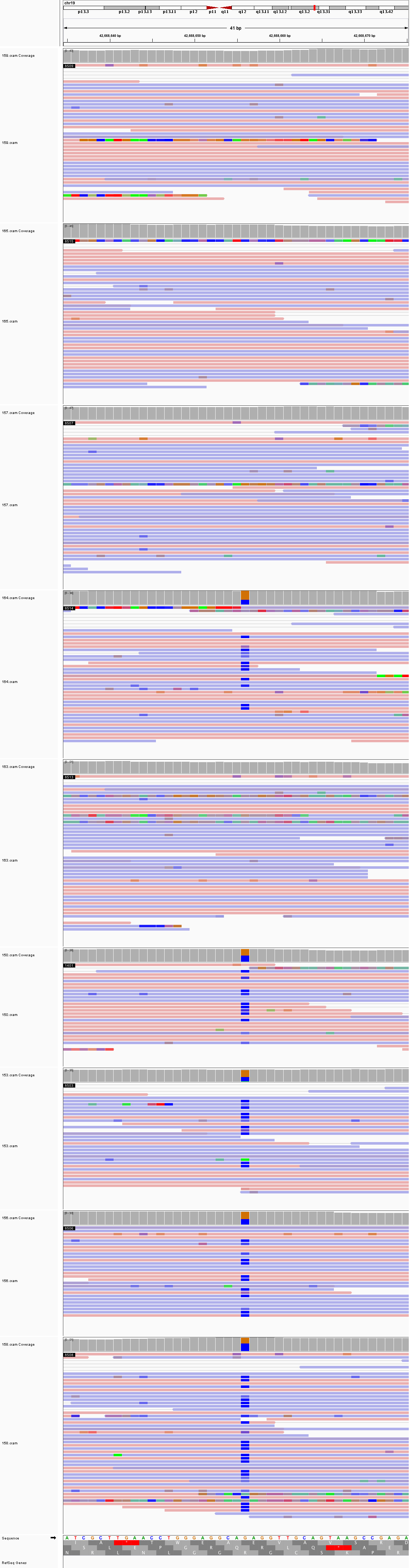

Supplement: Supplementary file 5. — In each image, the first two, three, or four tracks contain alignments from the grandparents in the pedigree (i.e., paternal grandmother and grandfather, maternal grandmother and grandfather). In some families, one or two of the first-generation grandparents were not sequenced (see Supplementary file 1). The two tracks below contain alignments from the second-generation individual with the putative gonosomal mutation and that second-generation individual’s spouse. The remaining tracks below contain alignments from the third-generation individuals that inherited the gonosomal mutation. Reads with mapping quality <20 are filtered out, as they were not considered by our variant calling pipeline, and mismatched bases are shaded by quality score (more transparent = lower base quality). [file elife-46922-supp5.zip › supp_file_5/chr19_42,668,635_42,668,675.png]

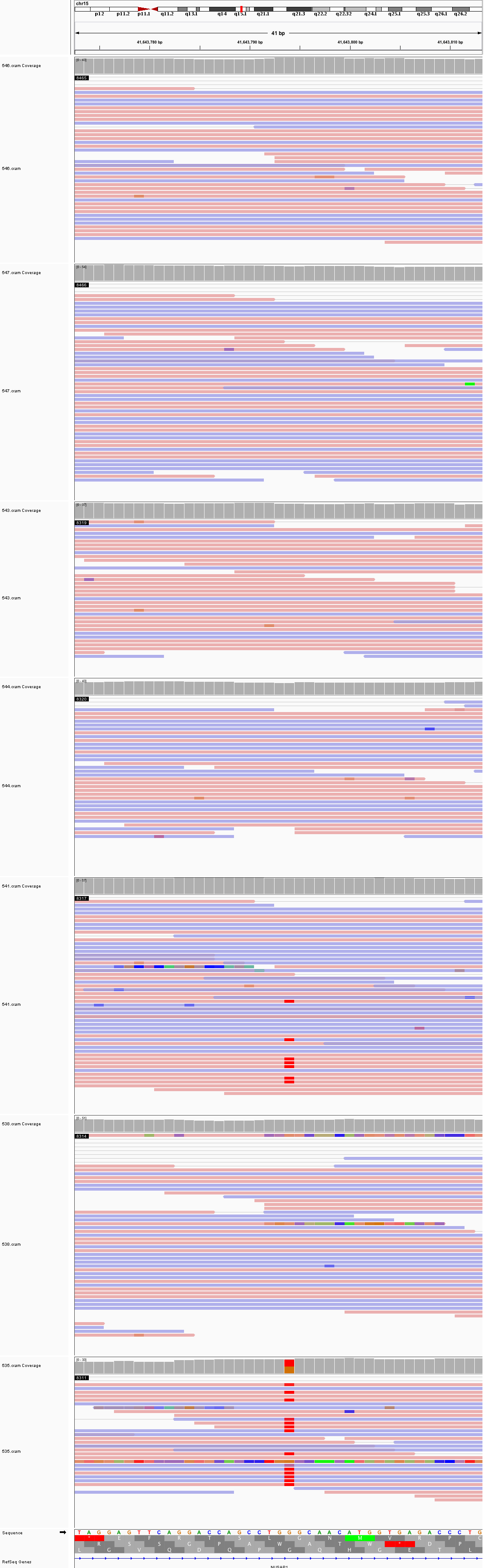

Supplement: Supplementary file 5. — In each image, the first two, three, or four tracks contain alignments from the grandparents in the pedigree (i.e., paternal grandmother and grandfather, maternal grandmother and grandfather). In some families, one or two of the first-generation grandparents were not sequenced (see Supplementary file 1). The two tracks below contain alignments from the second-generation individual with the putative gonosomal mutation and that second-generation individual’s spouse. The remaining tracks below contain alignments from the third-generation individuals that inherited the gonosomal mutation. Reads with mapping quality <20 are filtered out, as they were not considered by our variant calling pipeline, and mismatched bases are shaded by quality score (more transparent = lower base quality). [file elife-46922-supp5.zip › supp_file_5/chr15_41,643,773_41,643,813.png]

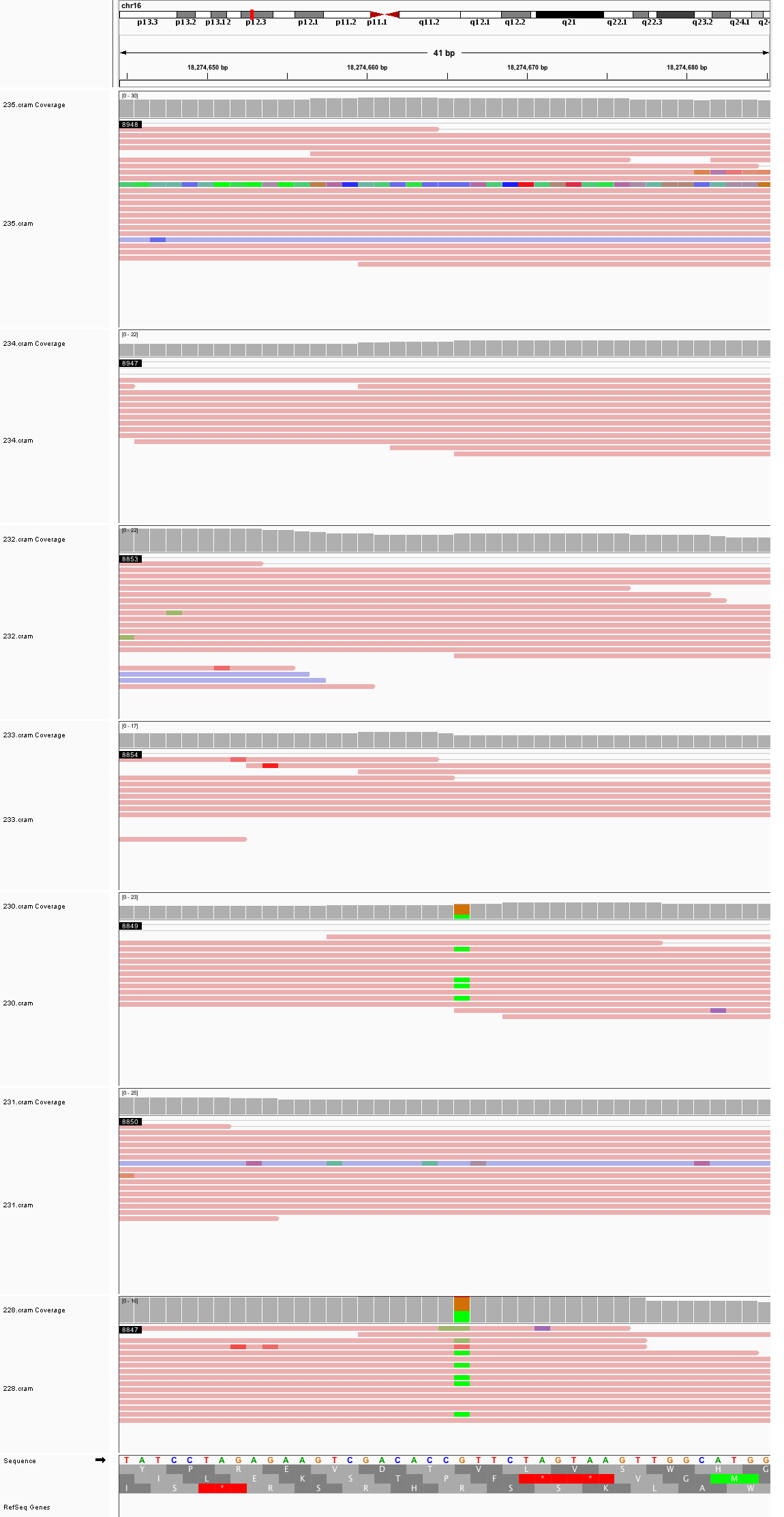

Supplement: Supplementary file 5. — In each image, the first two, three, or four tracks contain alignments from the grandparents in the pedigree (i.e., paternal grandmother and grandfather, maternal grandmother and grandfather). In some families, one or two of the first-generation grandparents were not sequenced (see Supplementary file 1). The two tracks below contain alignments from the second-generation individual with the putative gonosomal mutation and that second-generation individual’s spouse. The remaining tracks below contain alignments from the third-generation individuals that inherited the gonosomal mutation. Reads with mapping quality <20 are filtered out, as they were not considered by our variant calling pipeline, and mismatched bases are shaded by quality score (more transparent = lower base quality). [file elife-46922-supp5.zip › supp_file_5/chr16_18,274,645_18,274,685.png]

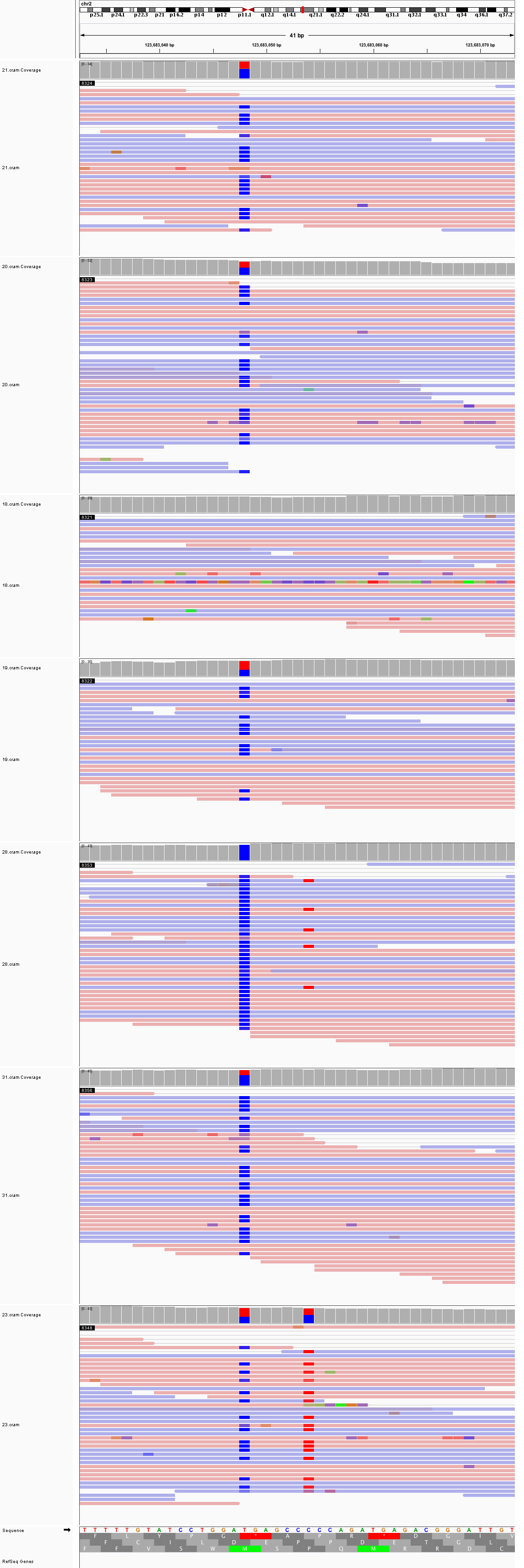

Supplement: Supplementary file 5. — In each image, the first two, three, or four tracks contain alignments from the grandparents in the pedigree (i.e., paternal grandmother and grandfather, maternal grandmother and grandfather). In some families, one or two of the first-generation grandparents were not sequenced (see Supplementary file 1). The two tracks below contain alignments from the second-generation individual with the putative gonosomal mutation and that second-generation individual’s spouse. The remaining tracks below contain alignments from the third-generation individuals that inherited the gonosomal mutation. Reads with mapping quality <20 are filtered out, as they were not considered by our variant calling pipeline, and mismatched bases are shaded by quality score (more transparent = lower base quality). [file elife-46922-supp5.zip › supp_file_5/chr2_123,683,033_123,683,073.png]

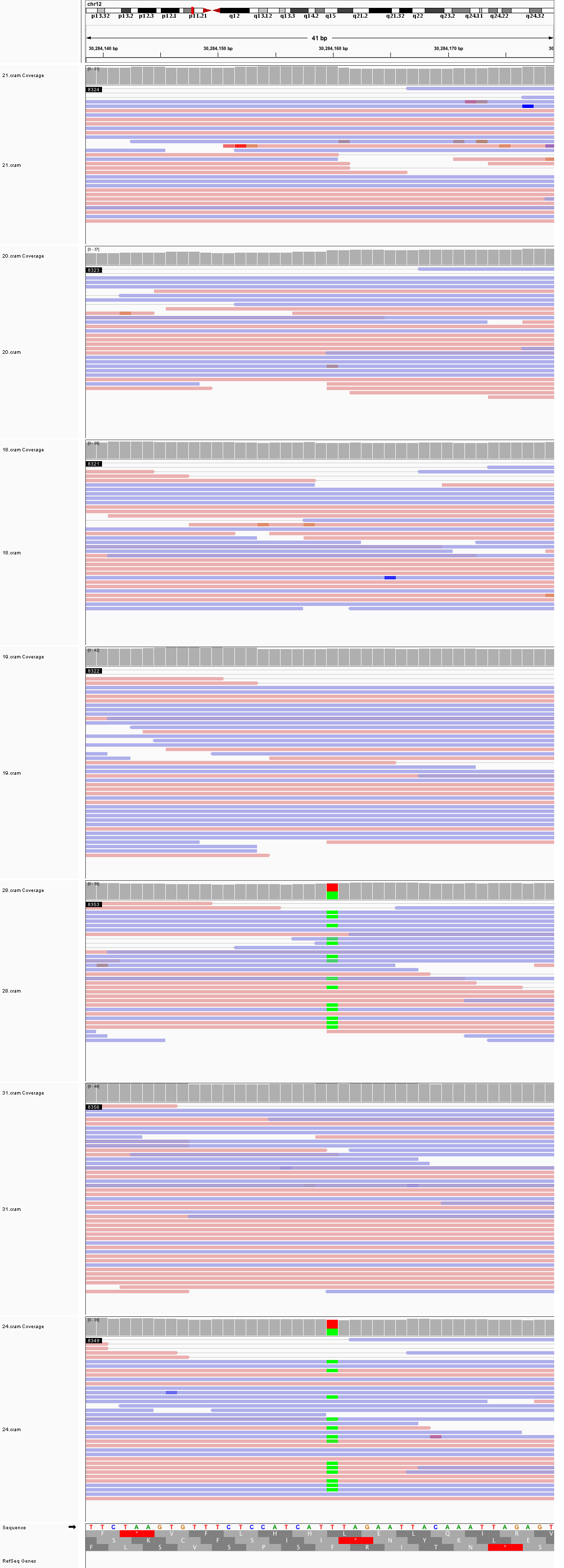

Supplement: Supplementary file 5. — In each image, the first two, three, or four tracks contain alignments from the grandparents in the pedigree (i.e., paternal grandmother and grandfather, maternal grandmother and grandfather). In some families, one or two of the first-generation grandparents were not sequenced (see Supplementary file 1). The two tracks below contain alignments from the second-generation individual with the putative gonosomal mutation and that second-generation individual’s spouse. The remaining tracks below contain alignments from the third-generation individuals that inherited the gonosomal mutation. Reads with mapping quality <20 are filtered out, as they were not considered by our variant calling pipeline, and mismatched bases are shaded by quality score (more transparent = lower base quality). [file elife-46922-supp5.zip › supp_file_5/chr12_30,284,139_30,284,179.png]

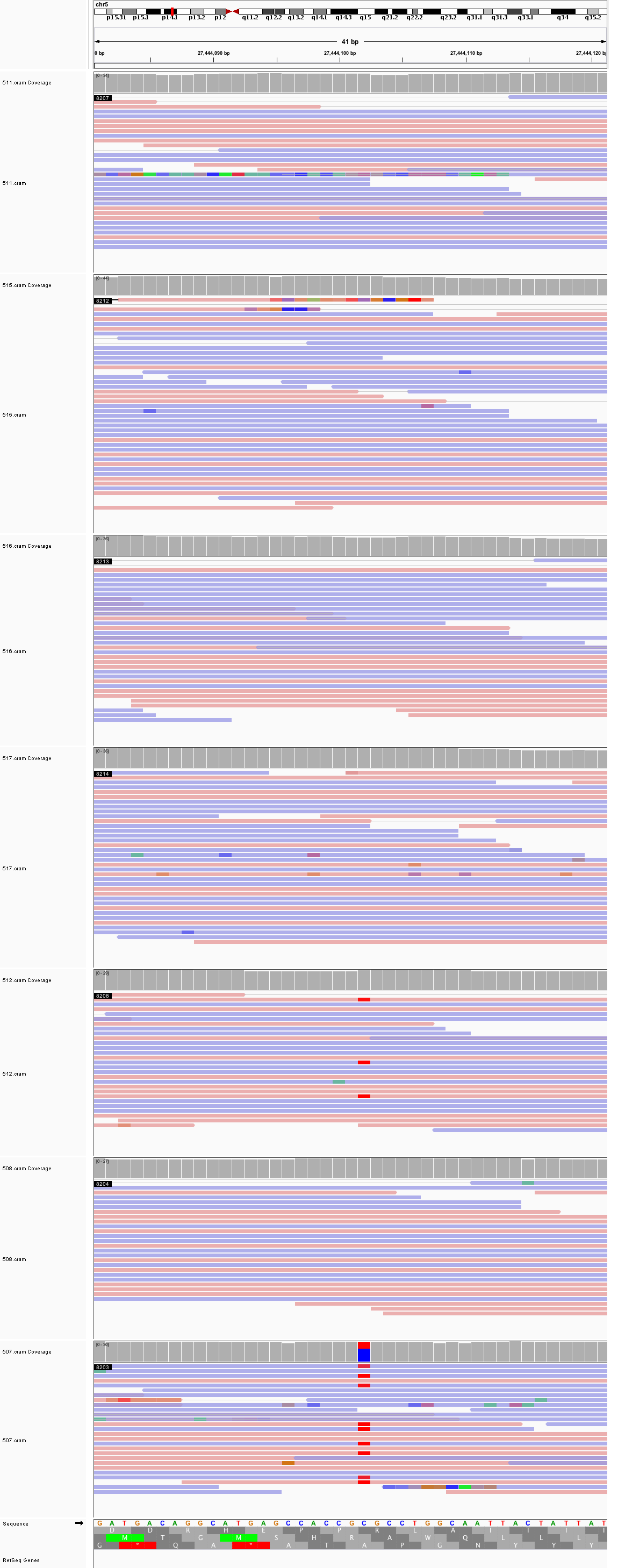

Supplement: Supplementary file 5. — In each image, the first two, three, or four tracks contain alignments from the grandparents in the pedigree (i.e., paternal grandmother and grandfather, maternal grandmother and grandfather). In some families, one or two of the first-generation grandparents were not sequenced (see Supplementary file 1). The two tracks below contain alignments from the second-generation individual with the putative gonosomal mutation and that second-generation individual’s spouse. The remaining tracks below contain alignments from the third-generation individuals that inherited the gonosomal mutation. Reads with mapping quality <20 are filtered out, as they were not considered by our variant calling pipeline, and mismatched bases are shaded by quality score (more transparent = lower base quality). [file elife-46922-supp5.zip › supp_file_5/chr5_27,444,081_27,444,121.png]

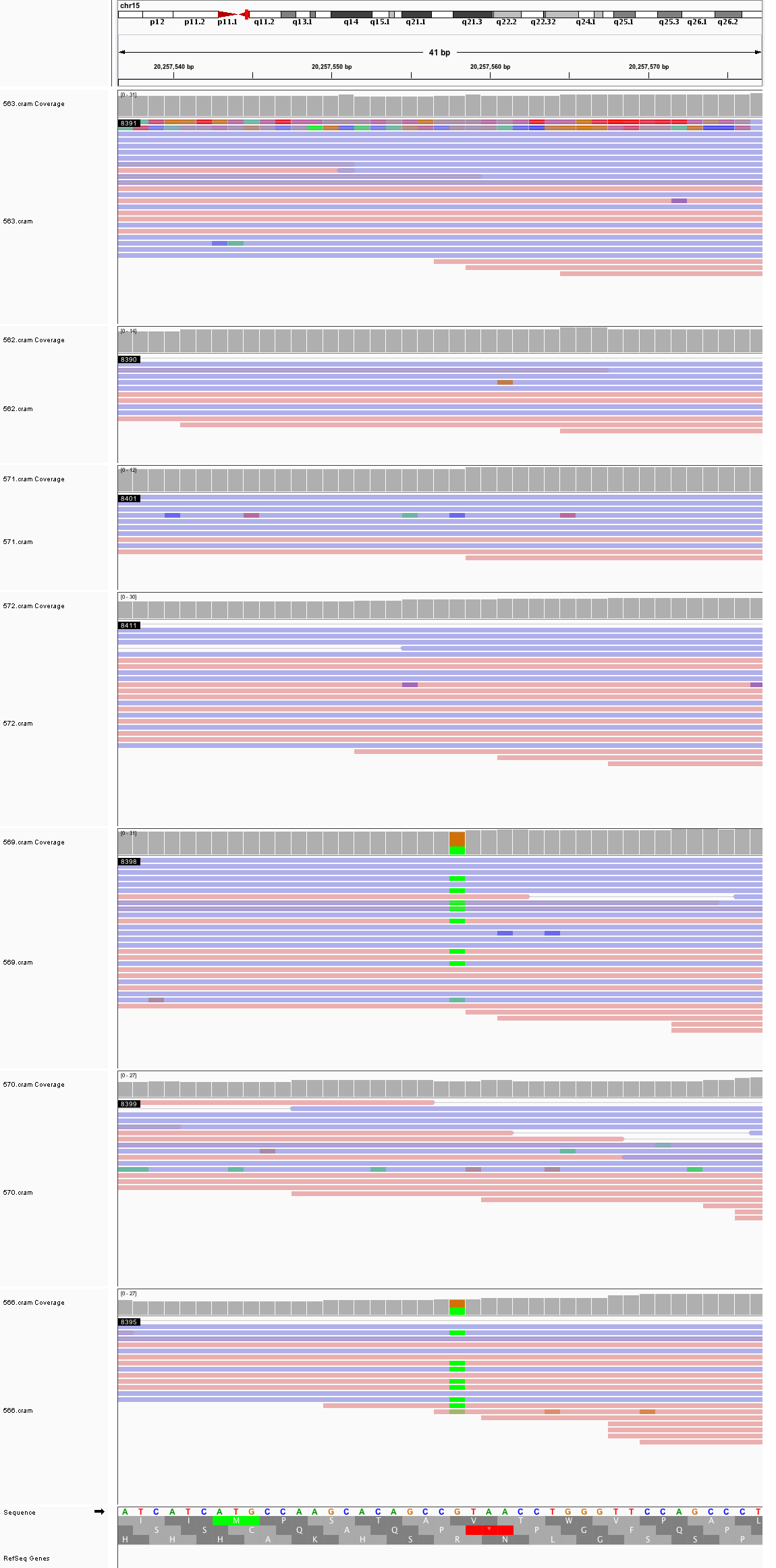

Supplement: Supplementary file 5. — In each image, the first two, three, or four tracks contain alignments from the grandparents in the pedigree (i.e., paternal grandmother and grandfather, maternal grandmother and grandfather). In some families, one or two of the first-generation grandparents were not sequenced (see Supplementary file 1). The two tracks below contain alignments from the second-generation individual with the putative gonosomal mutation and that second-generation individual’s spouse. The remaining tracks below contain alignments from the third-generation individuals that inherited the gonosomal mutation. Reads with mapping quality <20 are filtered out, as they were not considered by our variant calling pipeline, and mismatched bases are shaded by quality score (more transparent = lower base quality). [file elife-46922-supp5.zip › supp_file_5/chr15_20,257,537_20,257,577.png]

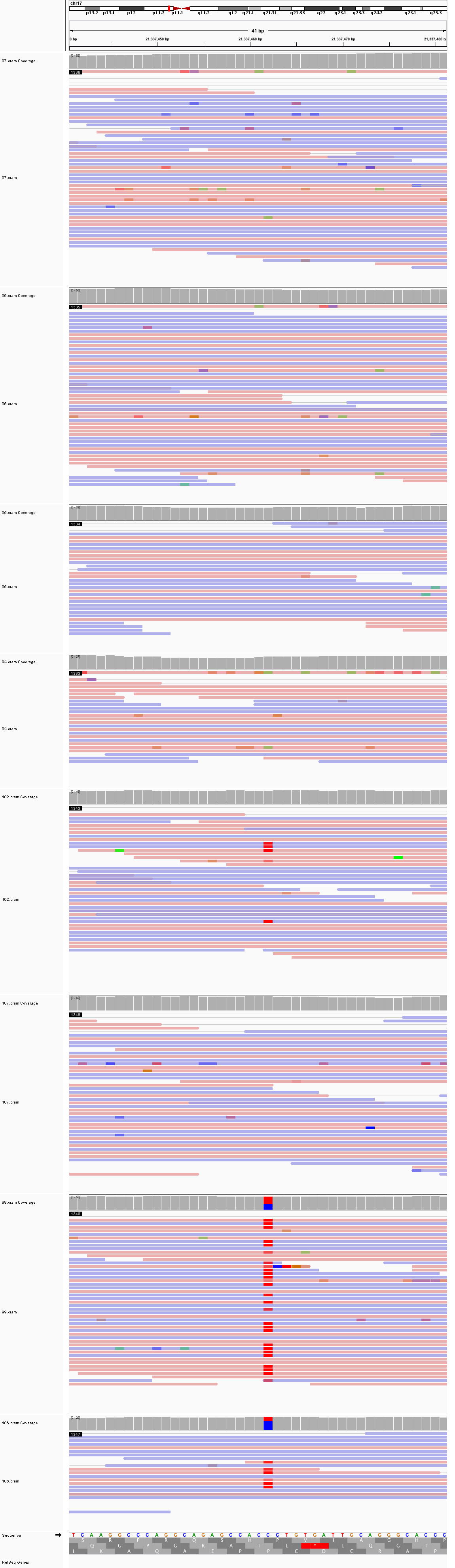

Supplement: Supplementary file 5. — In each image, the first two, three, or four tracks contain alignments from the grandparents in the pedigree (i.e., paternal grandmother and grandfather, maternal grandmother and grandfather). In some families, one or two of the first-generation grandparents were not sequenced (see Supplementary file 1). The two tracks below contain alignments from the second-generation individual with the putative gonosomal mutation and that second-generation individual’s spouse. The remaining tracks below contain alignments from the third-generation individuals that inherited the gonosomal mutation. Reads with mapping quality <20 are filtered out, as they were not considered by our variant calling pipeline, and mismatched bases are shaded by quality score (more transparent = lower base quality). [file elife-46922-supp5.zip › supp_file_5/chr17_21,337,441_21,337,481.png]

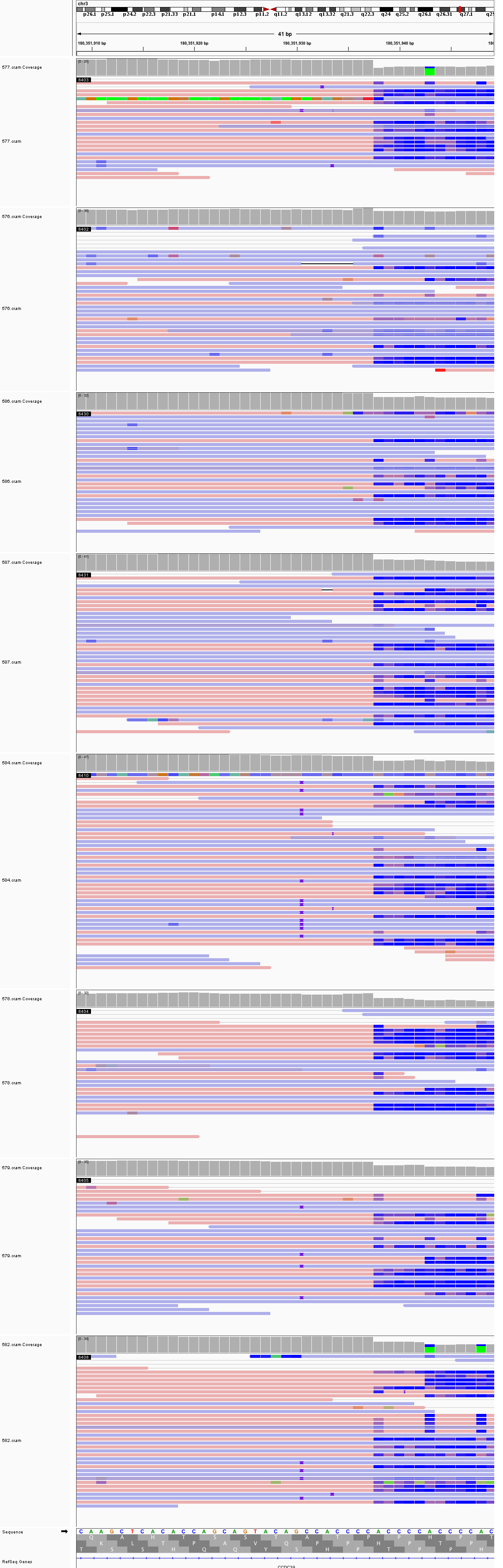

Supplement: Supplementary file 5. — In each image, the first two, three, or four tracks contain alignments from the grandparents in the pedigree (i.e., paternal grandmother and grandfather, maternal grandmother and grandfather). In some families, one or two of the first-generation grandparents were not sequenced (see Supplementary file 1). The two tracks below contain alignments from the second-generation individual with the putative gonosomal mutation and that second-generation individual’s spouse. The remaining tracks below contain alignments from the third-generation individuals that inherited the gonosomal mutation. Reads with mapping quality <20 are filtered out, as they were not considered by our variant calling pipeline, and mismatched bases are shaded by quality score (more transparent = lower base quality). [file elife-46922-supp5.zip › supp_file_5/chr3_180,351,909_180,351,949.png]

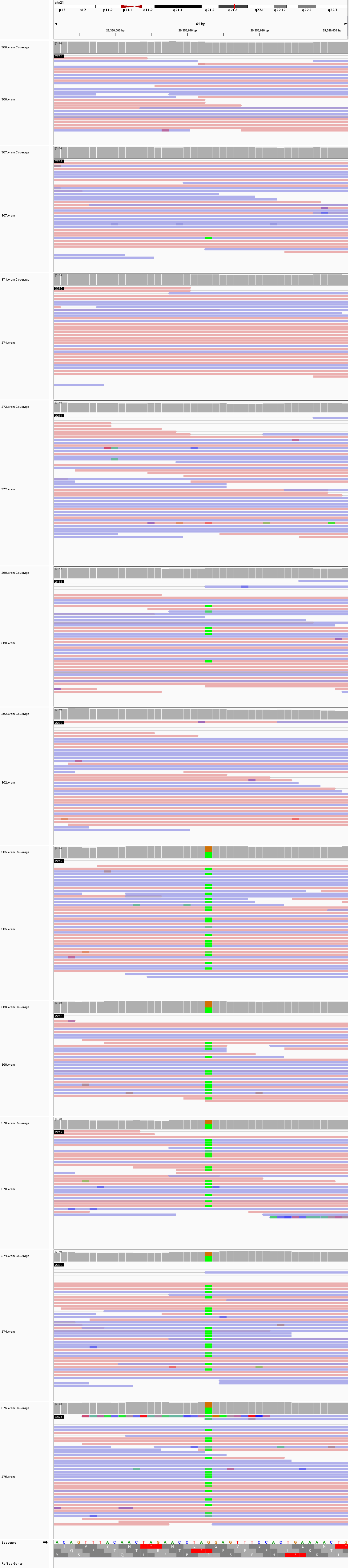

Supplement: Supplementary file 5. — In each image, the first two, three, or four tracks contain alignments from the grandparents in the pedigree (i.e., paternal grandmother and grandfather, maternal grandmother and grandfather). In some families, one or two of the first-generation grandparents were not sequenced (see Supplementary file 1). The two tracks below contain alignments from the second-generation individual with the putative gonosomal mutation and that second-generation individual’s spouse. The remaining tracks below contain alignments from the third-generation individuals that inherited the gonosomal mutation. Reads with mapping quality <20 are filtered out, as they were not considered by our variant calling pipeline, and mismatched bases are shaded by quality score (more transparent = lower base quality). [file elife-46922-supp5.zip › supp_file_5/chr21_29,349,992_29,350,032.png]

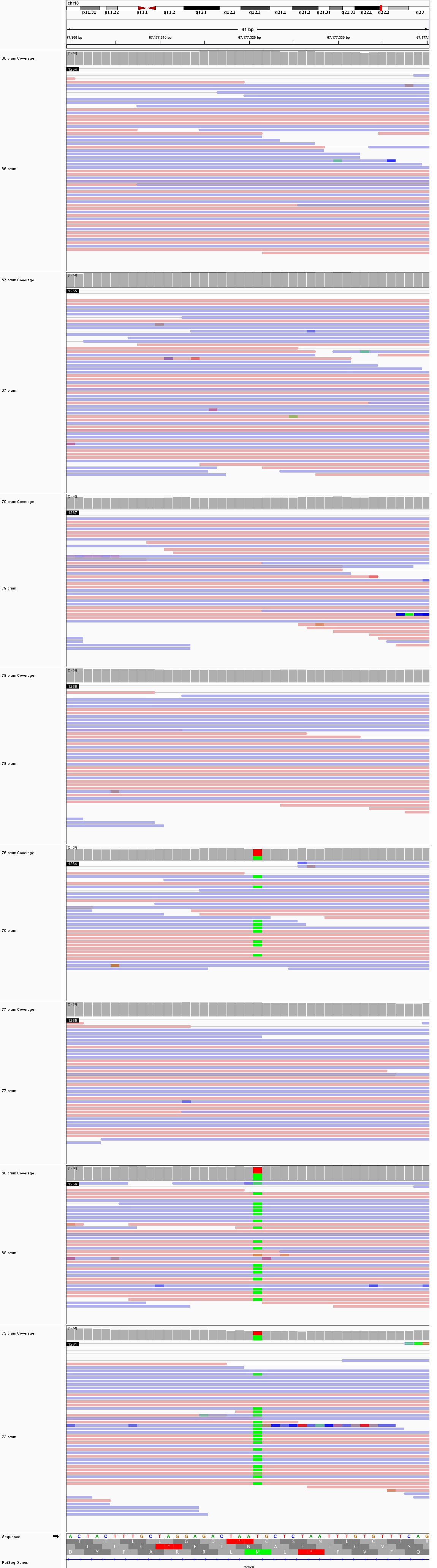

Supplement: Supplementary file 5. — In each image, the first two, three, or four tracks contain alignments from the grandparents in the pedigree (i.e., paternal grandmother and grandfather, maternal grandmother and grandfather). In some families, one or two of the first-generation grandparents were not sequenced (see Supplementary file 1). The two tracks below contain alignments from the second-generation individual with the putative gonosomal mutation and that second-generation individual’s spouse. The remaining tracks below contain alignments from the third-generation individuals that inherited the gonosomal mutation. Reads with mapping quality <20 are filtered out, as they were not considered by our variant calling pipeline, and mismatched bases are shaded by quality score (more transparent = lower base quality). [file elife-46922-supp5.zip › supp_file_5/chr18_67,177,300_67,177,340.png]

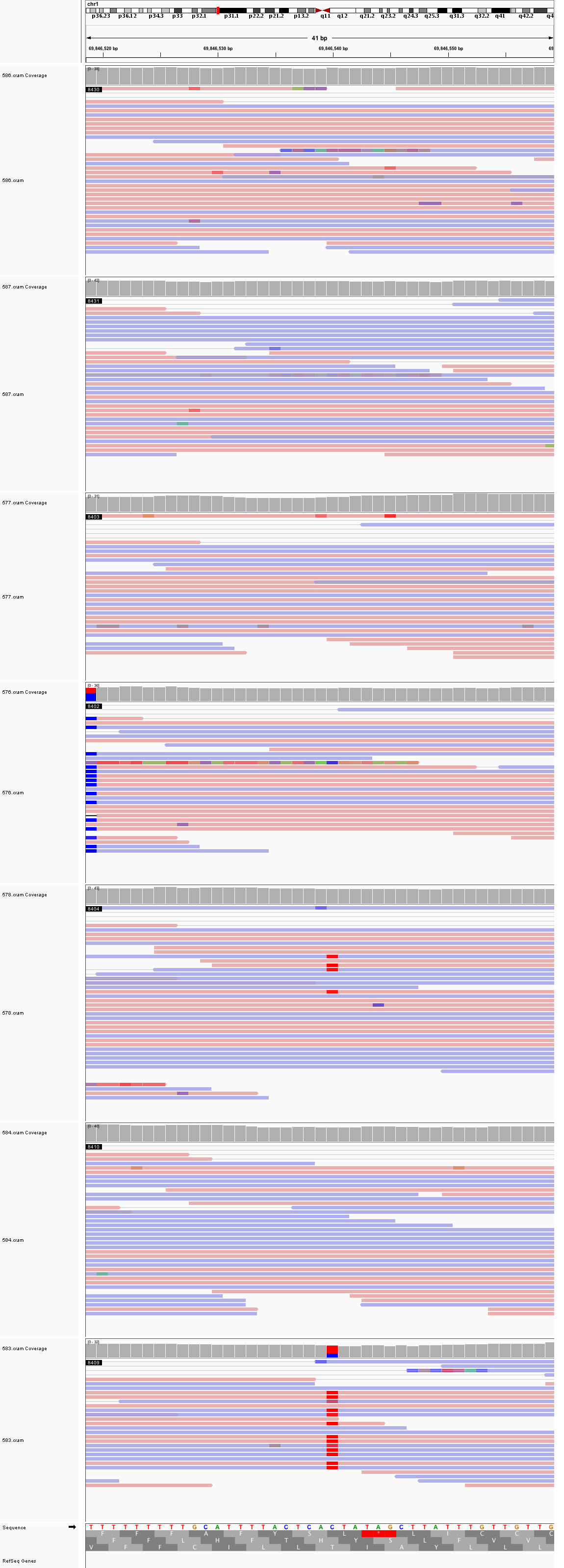

Supplement: Supplementary file 5. — In each image, the first two, three, or four tracks contain alignments from the grandparents in the pedigree (i.e., paternal grandmother and grandfather, maternal grandmother and grandfather). In some families, one or two of the first-generation grandparents were not sequenced (see Supplementary file 1). The two tracks below contain alignments from the second-generation individual with the putative gonosomal mutation and that second-generation individual’s spouse. The remaining tracks below contain alignments from the third-generation individuals that inherited the gonosomal mutation. Reads with mapping quality <20 are filtered out, as they were not considered by our variant calling pipeline, and mismatched bases are shaded by quality score (more transparent = lower base quality). [file elife-46922-supp5.zip › supp_file_5/chr1_69,846,519_69,846,559.png]

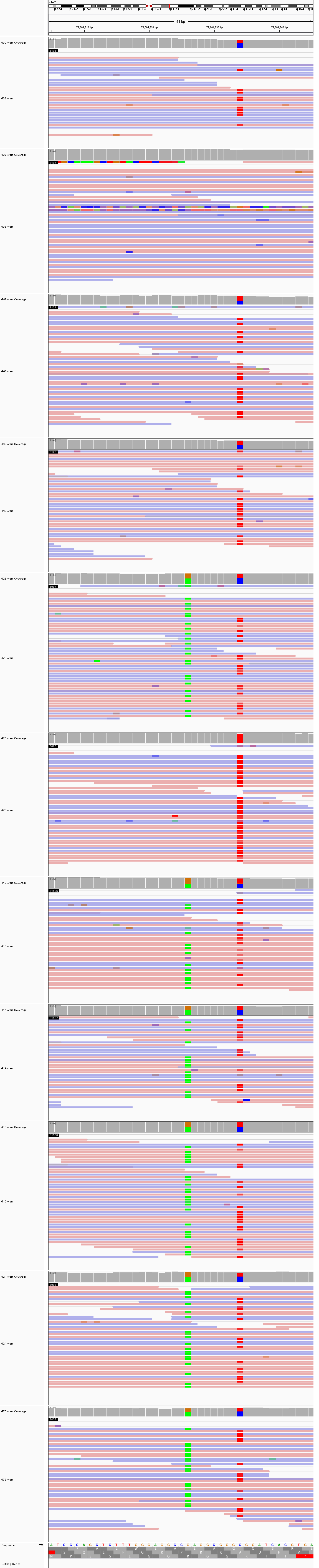

Supplement: Supplementary file 5. — In each image, the first two, three, or four tracks contain alignments from the grandparents in the pedigree (i.e., paternal grandmother and grandfather, maternal grandmother and grandfather). In some families, one or two of the first-generation grandparents were not sequenced (see Supplementary file 1). The two tracks below contain alignments from the second-generation individual with the putative gonosomal mutation and that second-generation individual’s spouse. The remaining tracks below contain alignments from the third-generation individuals that inherited the gonosomal mutation. Reads with mapping quality <20 are filtered out, as they were not considered by our variant calling pipeline, and mismatched bases are shaded by quality score (more transparent = lower base quality). [file elife-46922-supp5.zip › supp_file_5/chr7_72,004,505_72,004,545.png]

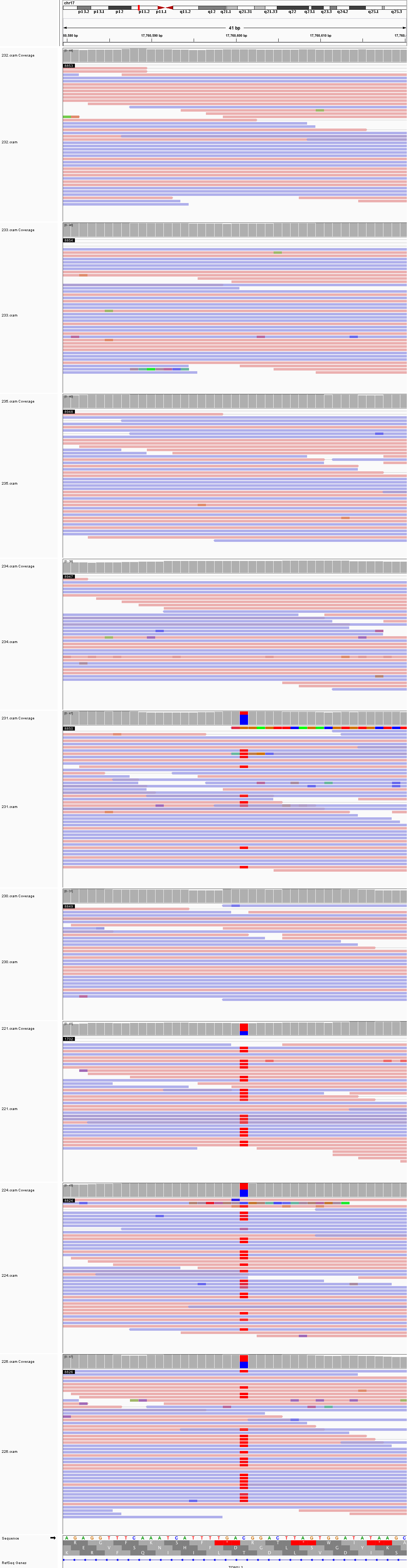

Supplement: Supplementary file 5. — In each image, the first two, three, or four tracks contain alignments from the grandparents in the pedigree (i.e., paternal grandmother and grandfather, maternal grandmother and grandfather). In some families, one or two of the first-generation grandparents were not sequenced (see Supplementary file 1). The two tracks below contain alignments from the second-generation individual with the putative gonosomal mutation and that second-generation individual’s spouse. The remaining tracks below contain alignments from the third-generation individuals that inherited the gonosomal mutation. Reads with mapping quality <20 are filtered out, as they were not considered by our variant calling pipeline, and mismatched bases are shaded by quality score (more transparent = lower base quality). [file elife-46922-supp5.zip › supp_file_5/chr17_17,760,580_17,760,620.png]

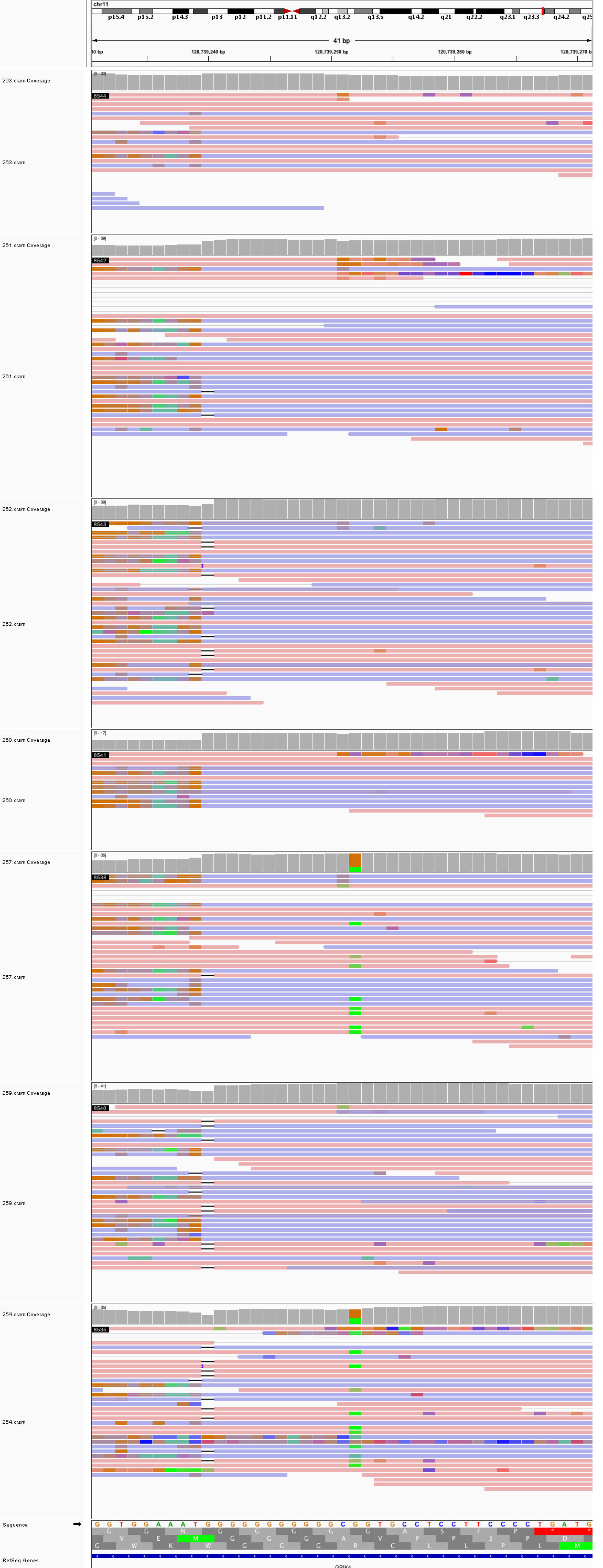

Supplement: Supplementary file 5. — In each image, the first two, three, or four tracks contain alignments from the grandparents in the pedigree (i.e., paternal grandmother and grandfather, maternal grandmother and grandfather). In some families, one or two of the first-generation grandparents were not sequenced (see Supplementary file 1). The two tracks below contain alignments from the second-generation individual with the putative gonosomal mutation and that second-generation individual’s spouse. The remaining tracks below contain alignments from the third-generation individuals that inherited the gonosomal mutation. Reads with mapping quality <20 are filtered out, as they were not considered by our variant calling pipeline, and mismatched bases are shaded by quality score (more transparent = lower base quality). [file elife-46922-supp5.zip › supp_file_5/chr11_120,739,231_120,739,271.png]

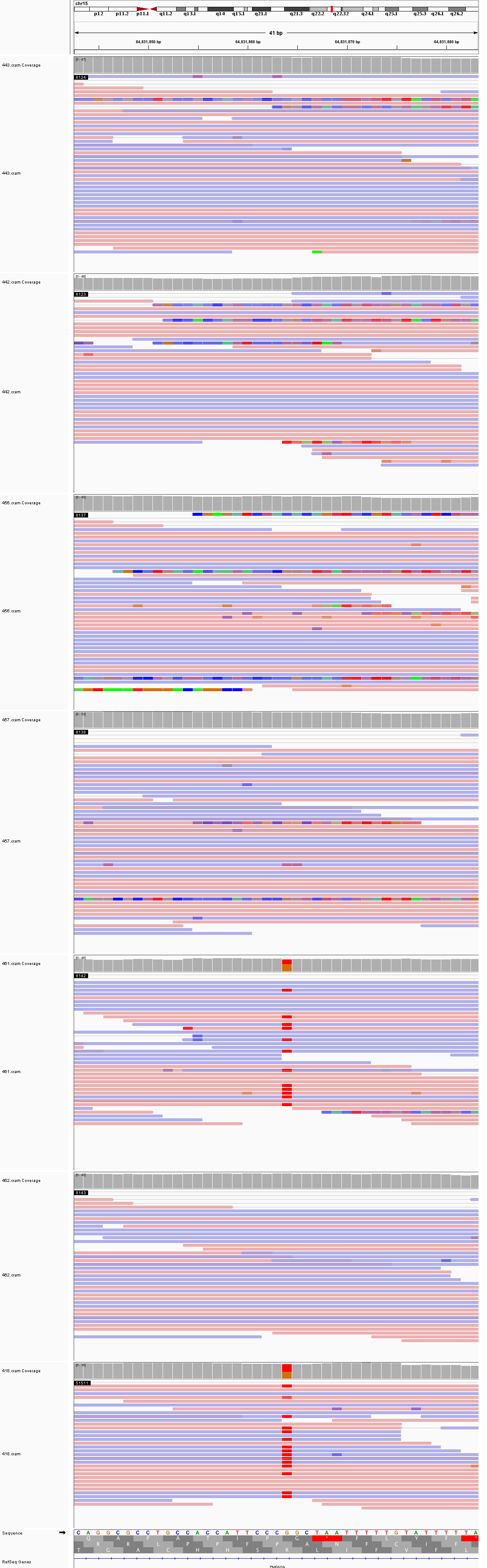

Supplement: Supplementary file 5. — In each image, the first two, three, or four tracks contain alignments from the grandparents in the pedigree (i.e., paternal grandmother and grandfather, maternal grandmother and grandfather). In some families, one or two of the first-generation grandparents were not sequenced (see Supplementary file 1). The two tracks below contain alignments from the second-generation individual with the putative gonosomal mutation and that second-generation individual’s spouse. The remaining tracks below contain alignments from the third-generation individuals that inherited the gonosomal mutation. Reads with mapping quality <20 are filtered out, as they were not considered by our variant calling pipeline, and mismatched bases are shaded by quality score (more transparent = lower base quality). [file elife-46922-supp5.zip › supp_file_5/chr15_64,831,843_64,831,883.png]

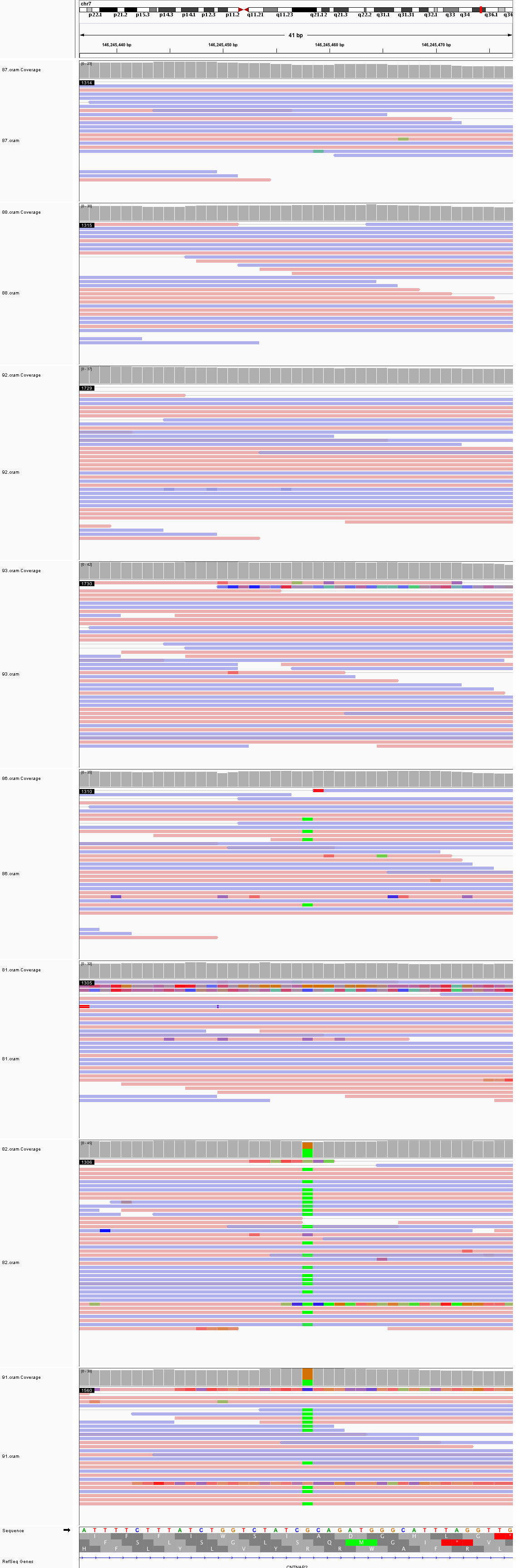

Supplement: Supplementary file 5. — In each image, the first two, three, or four tracks contain alignments from the grandparents in the pedigree (i.e., paternal grandmother and grandfather, maternal grandmother and grandfather). In some families, one or two of the first-generation grandparents were not sequenced (see Supplementary file 1). The two tracks below contain alignments from the second-generation individual with the putative gonosomal mutation and that second-generation individual’s spouse. The remaining tracks below contain alignments from the third-generation individuals that inherited the gonosomal mutation. Reads with mapping quality <20 are filtered out, as they were not considered by our variant calling pipeline, and mismatched bases are shaded by quality score (more transparent = lower base quality). [file elife-46922-supp5.zip › supp_file_5/chr7_146,245,437_146,245,477.png]

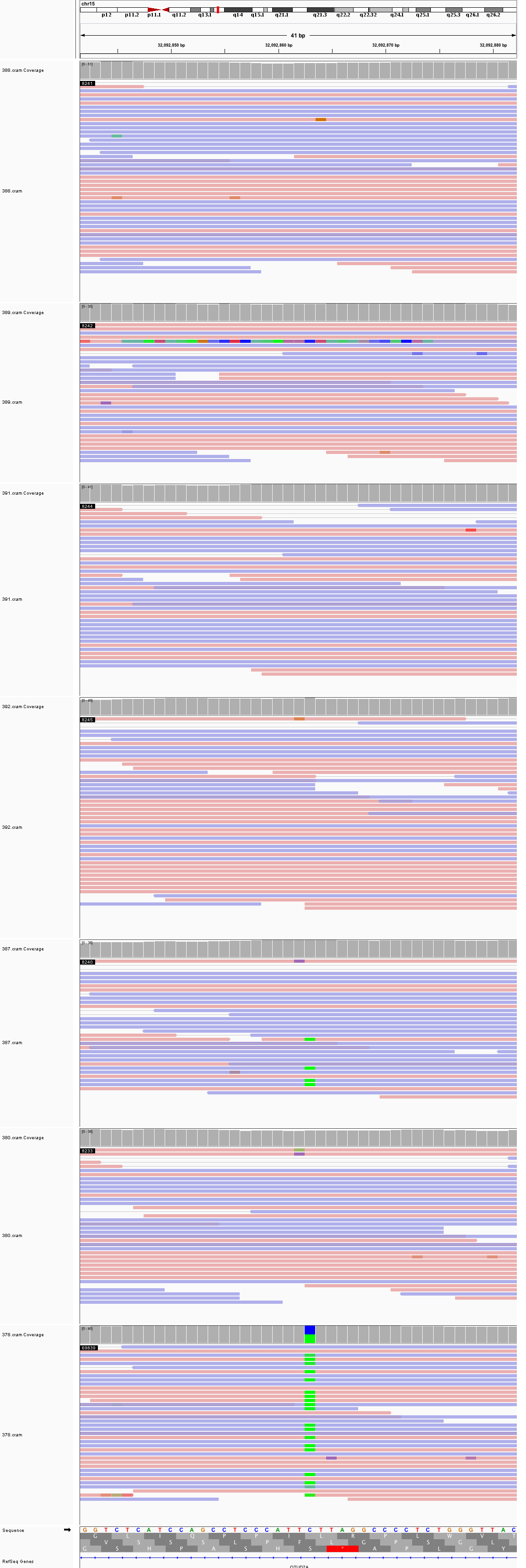

Supplement: Supplementary file 5. — In each image, the first two, three, or four tracks contain alignments from the grandparents in the pedigree (i.e., paternal grandmother and grandfather, maternal grandmother and grandfather). In some families, one or two of the first-generation grandparents were not sequenced (see Supplementary file 1). The two tracks below contain alignments from the second-generation individual with the putative gonosomal mutation and that second-generation individual’s spouse. The remaining tracks below contain alignments from the third-generation individuals that inherited the gonosomal mutation. Reads with mapping quality <20 are filtered out, as they were not considered by our variant calling pipeline, and mismatched bases are shaded by quality score (more transparent = lower base quality). [file elife-46922-supp5.zip › supp_file_5/chr15_32,092,842_32,092,882.png]

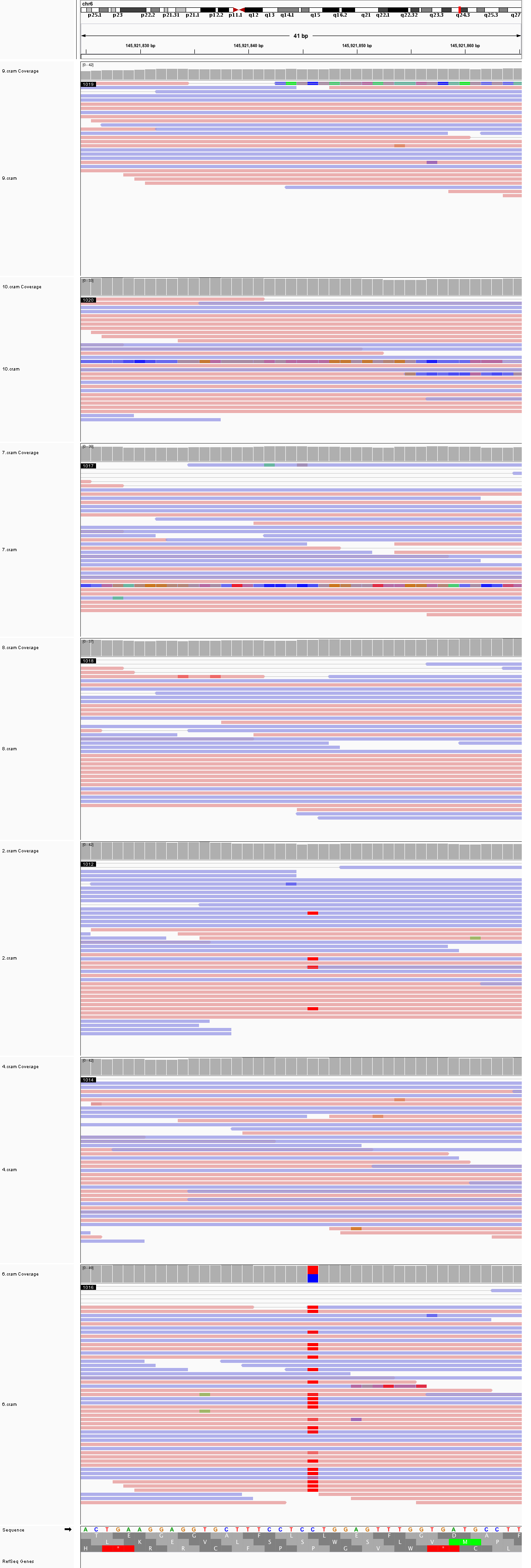

Supplement: Supplementary file 5. — In each image, the first two, three, or four tracks contain alignments from the grandparents in the pedigree (i.e., paternal grandmother and grandfather, maternal grandmother and grandfather). In some families, one or two of the first-generation grandparents were not sequenced (see Supplementary file 1). The two tracks below contain alignments from the second-generation individual with the putative gonosomal mutation and that second-generation individual’s spouse. The remaining tracks below contain alignments from the third-generation individuals that inherited the gonosomal mutation. Reads with mapping quality <20 are filtered out, as they were not considered by our variant calling pipeline, and mismatched bases are shaded by quality score (more transparent = lower base quality). [file elife-46922-supp5.zip › supp_file_5/chr6_145,921,825_145,921,865.png]

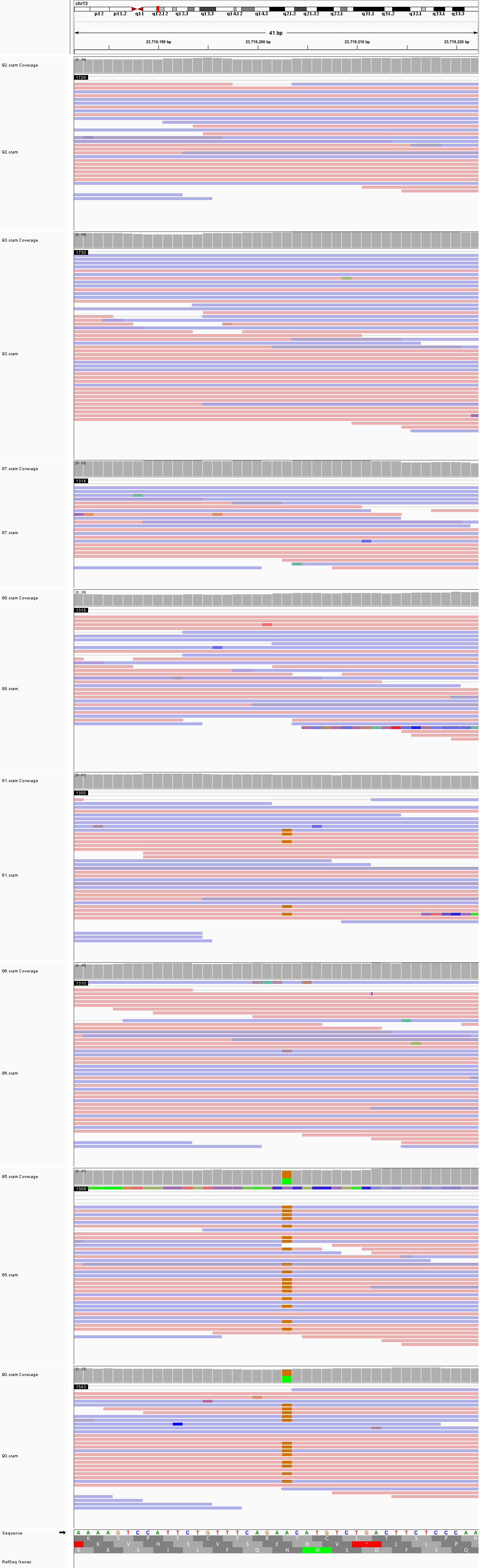

Supplement: Supplementary file 5. — In each image, the first two, three, or four tracks contain alignments from the grandparents in the pedigree (i.e., paternal grandmother and grandfather, maternal grandmother and grandfather). In some families, one or two of the first-generation grandparents were not sequenced (see Supplementary file 1). The two tracks below contain alignments from the second-generation individual with the putative gonosomal mutation and that second-generation individual’s spouse. The remaining tracks below contain alignments from the third-generation individuals that inherited the gonosomal mutation. Reads with mapping quality <20 are filtered out, as they were not considered by our variant calling pipeline, and mismatched bases are shaded by quality score (more transparent = lower base quality). [file elife-46922-supp5.zip › supp_file_5/chr13_23,719,182_23,719,222.png]

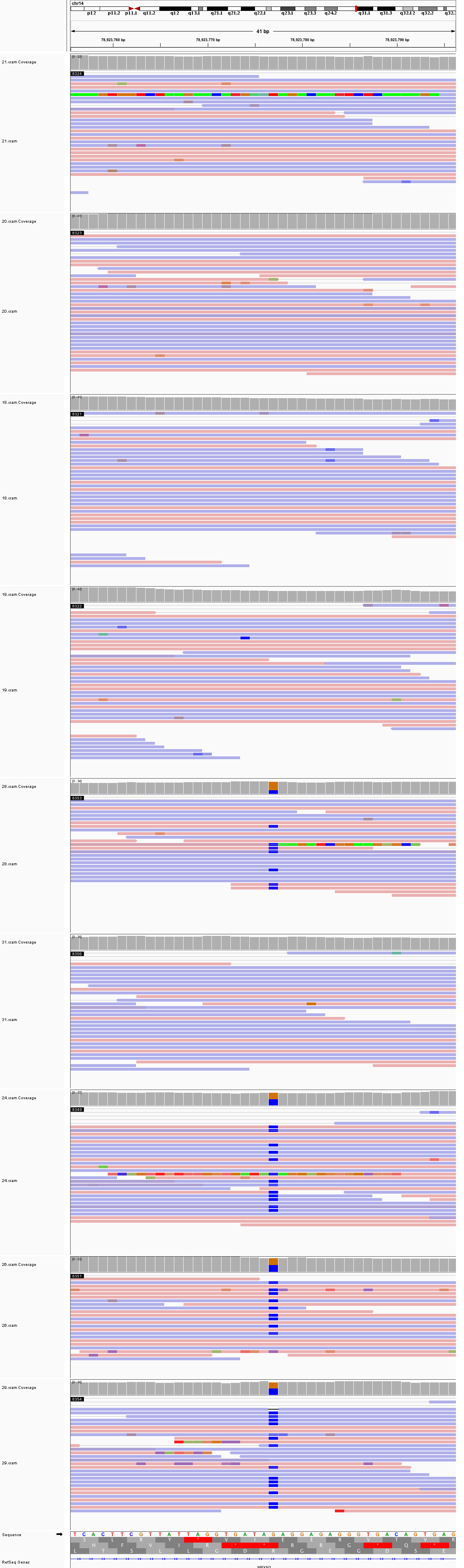

Supplement: Supplementary file 5. — In each image, the first two, three, or four tracks contain alignments from the grandparents in the pedigree (i.e., paternal grandmother and grandfather, maternal grandmother and grandfather). In some families, one or two of the first-generation grandparents were not sequenced (see Supplementary file 1). The two tracks below contain alignments from the second-generation individual with the putative gonosomal mutation and that second-generation individual’s spouse. The remaining tracks below contain alignments from the third-generation individuals that inherited the gonosomal mutation. Reads with mapping quality <20 are filtered out, as they were not considered by our variant calling pipeline, and mismatched bases are shaded by quality score (more transparent = lower base quality). [file elife-46922-supp5.zip › supp_file_5/chr14_78,923,756_78,923,796.png]

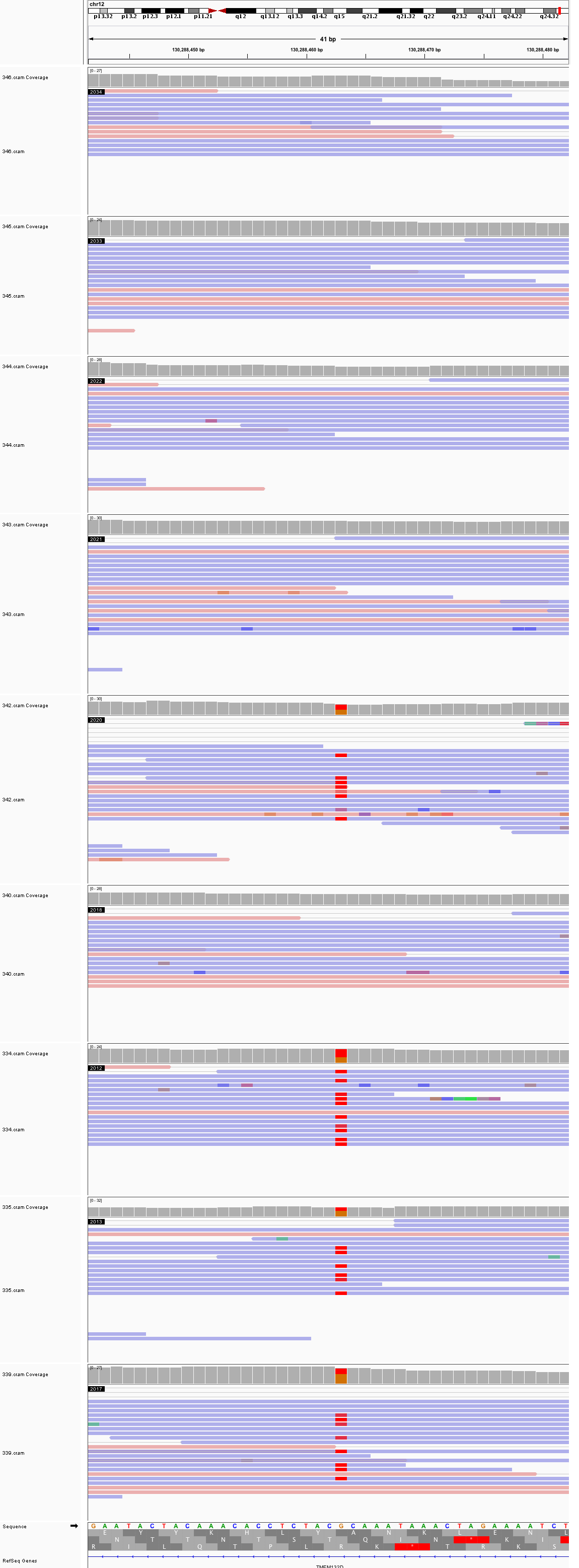

Supplement: Supplementary file 5. — In each image, the first two, three, or four tracks contain alignments from the grandparents in the pedigree (i.e., paternal grandmother and grandfather, maternal grandmother and grandfather). In some families, one or two of the first-generation grandparents were not sequenced (see Supplementary file 1). The two tracks below contain alignments from the second-generation individual with the putative gonosomal mutation and that second-generation individual’s spouse. The remaining tracks below contain alignments from the third-generation individuals that inherited the gonosomal mutation. Reads with mapping quality <20 are filtered out, as they were not considered by our variant calling pipeline, and mismatched bases are shaded by quality score (more transparent = lower base quality). [file elife-46922-supp5.zip › supp_file_5/chr12_130,288,442_130,288,482.png]

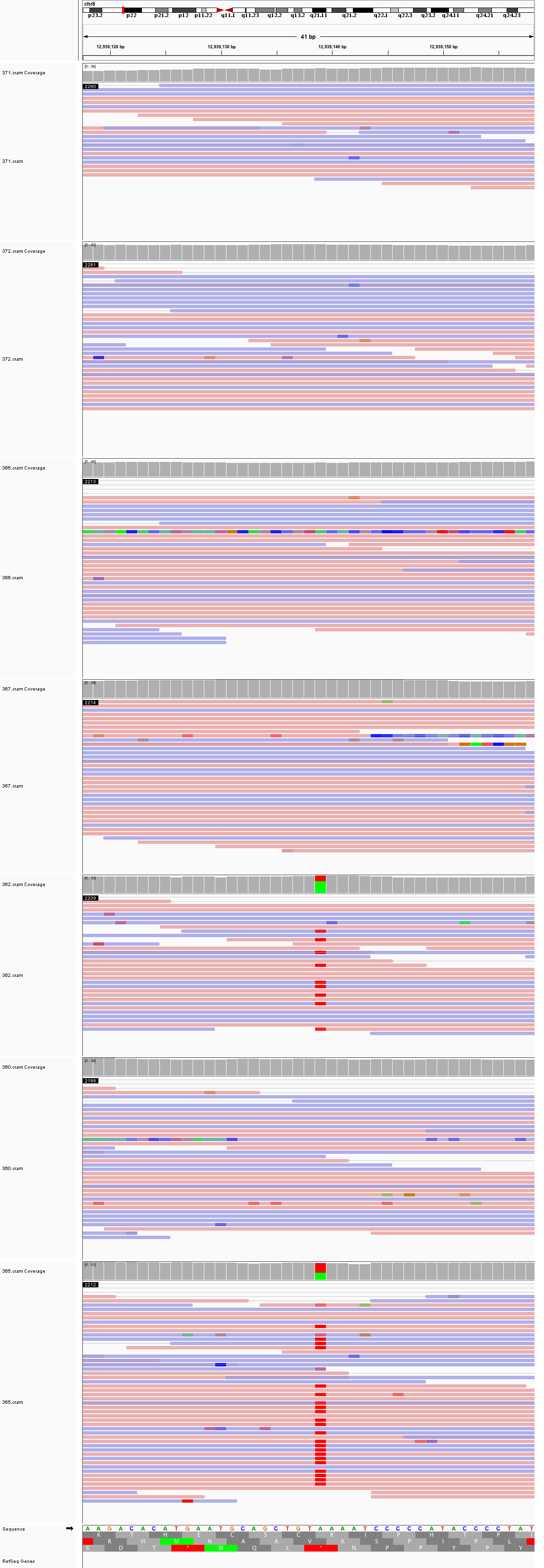

Supplement: Supplementary file 5. — In each image, the first two, three, or four tracks contain alignments from the grandparents in the pedigree (i.e., paternal grandmother and grandfather, maternal grandmother and grandfather). In some families, one or two of the first-generation grandparents were not sequenced (see Supplementary file 1). The two tracks below contain alignments from the second-generation individual with the putative gonosomal mutation and that second-generation individual’s spouse. The remaining tracks below contain alignments from the third-generation individuals that inherited the gonosomal mutation. Reads with mapping quality <20 are filtered out, as they were not considered by our variant calling pipeline, and mismatched bases are shaded by quality score (more transparent = lower base quality). [file elife-46922-supp5.zip › supp_file_5/chr8_12,939,118_12,939,158.png]

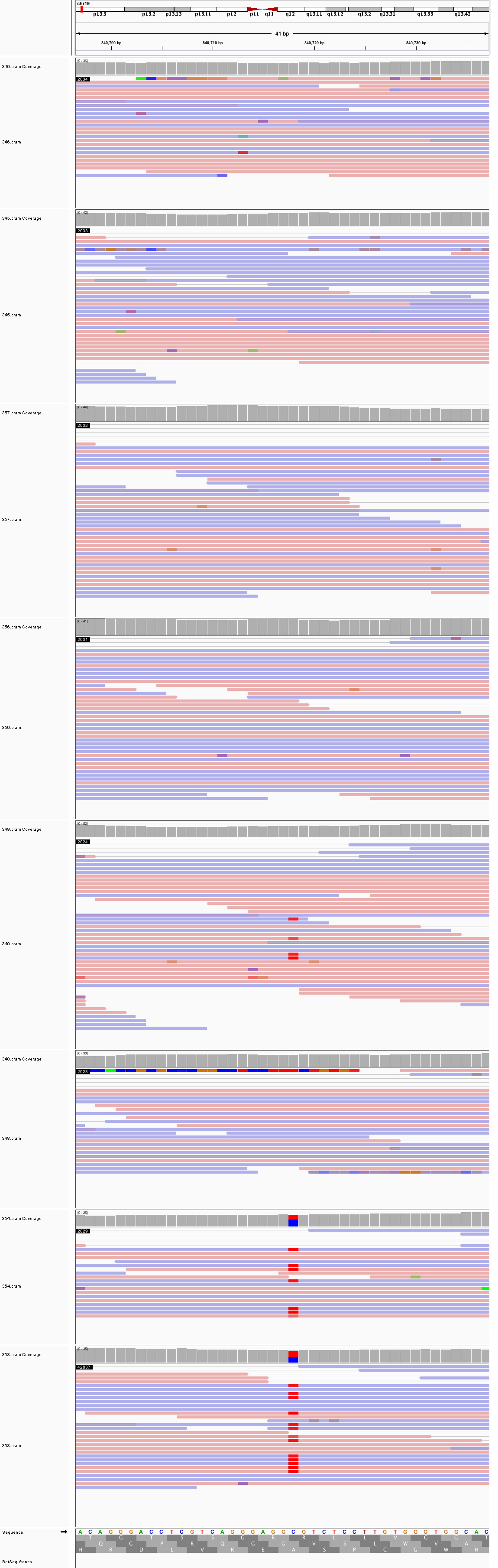

Supplement: Supplementary file 5. — In each image, the first two, three, or four tracks contain alignments from the grandparents in the pedigree (i.e., paternal grandmother and grandfather, maternal grandmother and grandfather). In some families, one or two of the first-generation grandparents were not sequenced (see Supplementary file 1). The two tracks below contain alignments from the second-generation individual with the putative gonosomal mutation and that second-generation individual’s spouse. The remaining tracks below contain alignments from the third-generation individuals that inherited the gonosomal mutation. Reads with mapping quality <20 are filtered out, as they were not considered by our variant calling pipeline, and mismatched bases are shaded by quality score (more transparent = lower base quality). [file elife-46922-supp5.zip › supp_file_5/chr19_840,697_840,737.png]

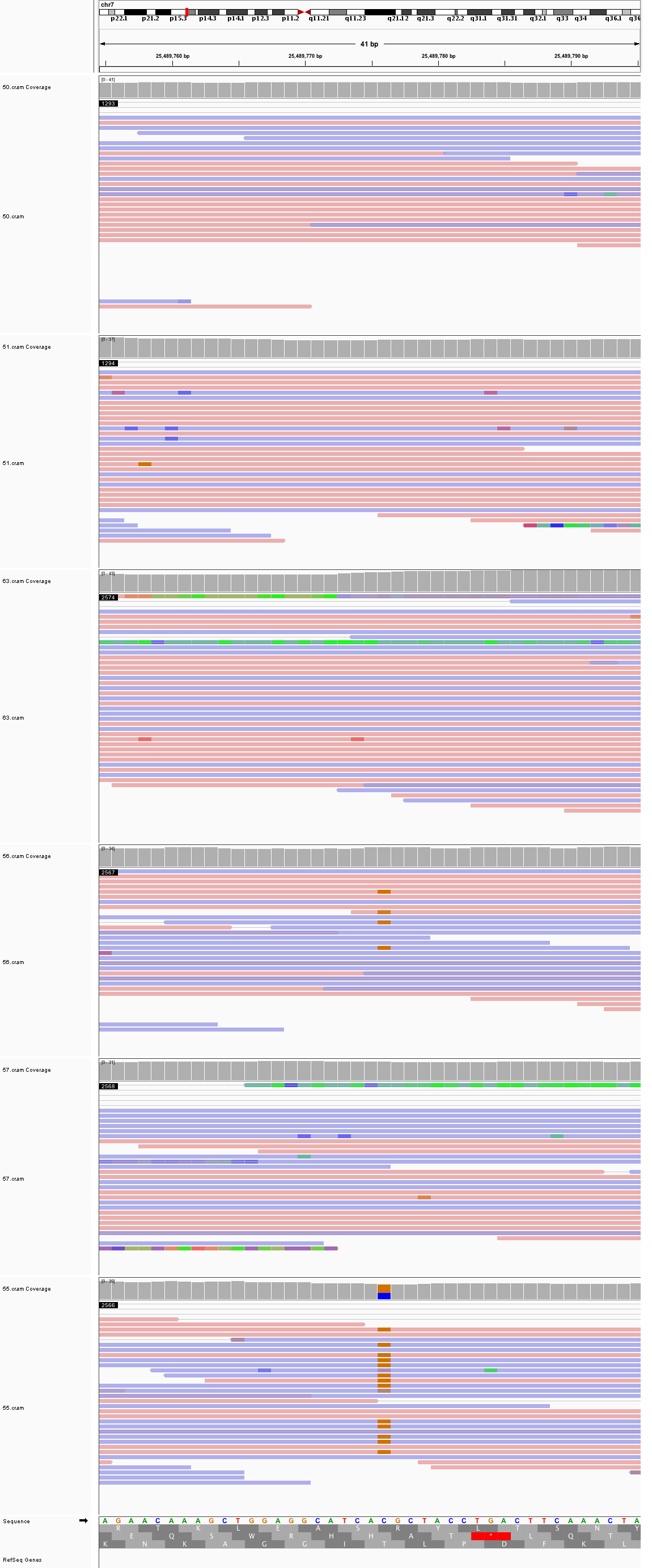

Supplement: Supplementary file 5. — In each image, the first two, three, or four tracks contain alignments from the grandparents in the pedigree (i.e., paternal grandmother and grandfather, maternal grandmother and grandfather). In some families, one or two of the first-generation grandparents were not sequenced (see Supplementary file 1). The two tracks below contain alignments from the second-generation individual with the putative gonosomal mutation and that second-generation individual’s spouse. The remaining tracks below contain alignments from the third-generation individuals that inherited the gonosomal mutation. Reads with mapping quality <20 are filtered out, as they were not considered by our variant calling pipeline, and mismatched bases are shaded by quality score (more transparent = lower base quality). [file elife-46922-supp5.zip › supp_file_5/chr7_25,489,755_25,489,795.png]

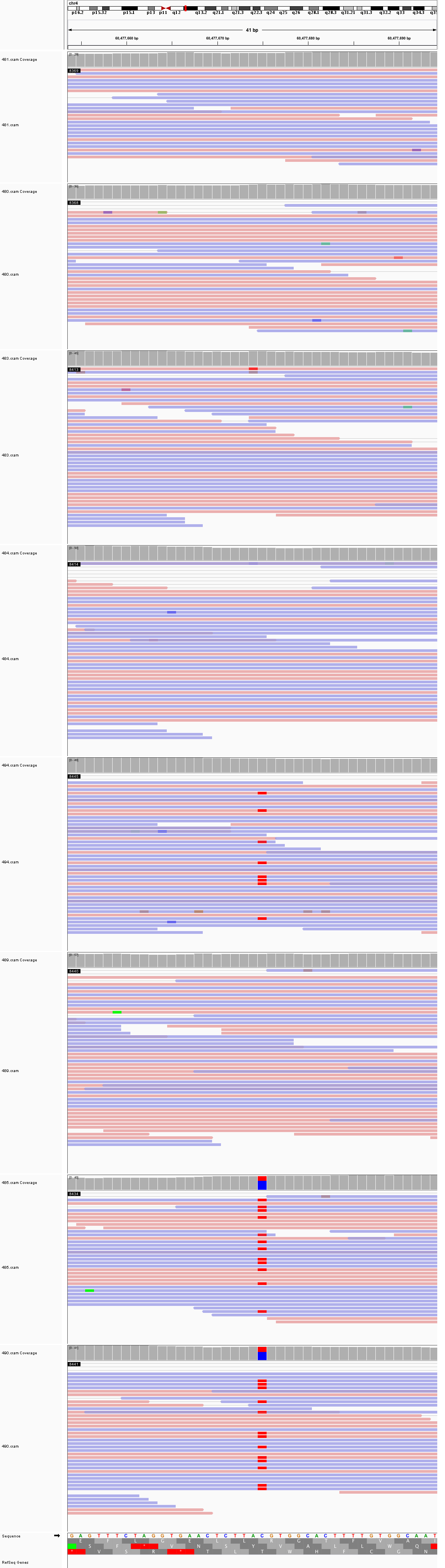

Supplement: Supplementary file 5. — In each image, the first two, three, or four tracks contain alignments from the grandparents in the pedigree (i.e., paternal grandmother and grandfather, maternal grandmother and grandfather). In some families, one or two of the first-generation grandparents were not sequenced (see Supplementary file 1). The two tracks below contain alignments from the second-generation individual with the putative gonosomal mutation and that second-generation individual’s spouse. The remaining tracks below contain alignments from the third-generation individuals that inherited the gonosomal mutation. Reads with mapping quality <20 are filtered out, as they were not considered by our variant calling pipeline, and mismatched bases are shaded by quality score (more transparent = lower base quality). [file elife-46922-supp5.zip › supp_file_5/chr4_60,477,654_60,477,694.png]

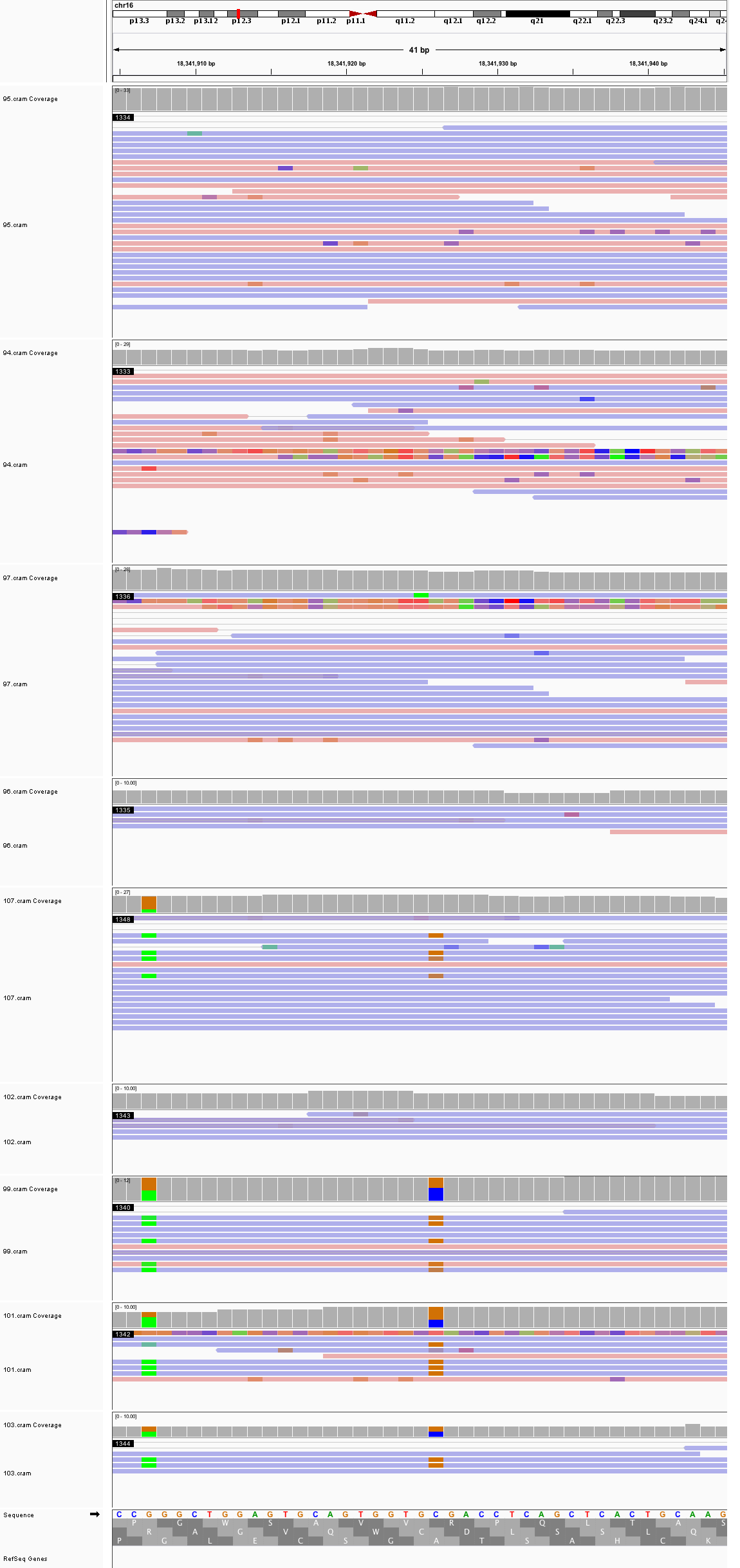

Supplement: Supplementary file 5. — In each image, the first two, three, or four tracks contain alignments from the grandparents in the pedigree (i.e., paternal grandmother and grandfather, maternal grandmother and grandfather). In some families, one or two of the first-generation grandparents were not sequenced (see Supplementary file 1). The two tracks below contain alignments from the second-generation individual with the putative gonosomal mutation and that second-generation individual’s spouse. The remaining tracks below contain alignments from the third-generation individuals that inherited the gonosomal mutation. Reads with mapping quality <20 are filtered out, as they were not considered by our variant calling pipeline, and mismatched bases are shaded by quality score (more transparent = lower base quality). [file elife-46922-supp5.zip › supp_file_5/chr16_18,341,905_18,341,945.png]

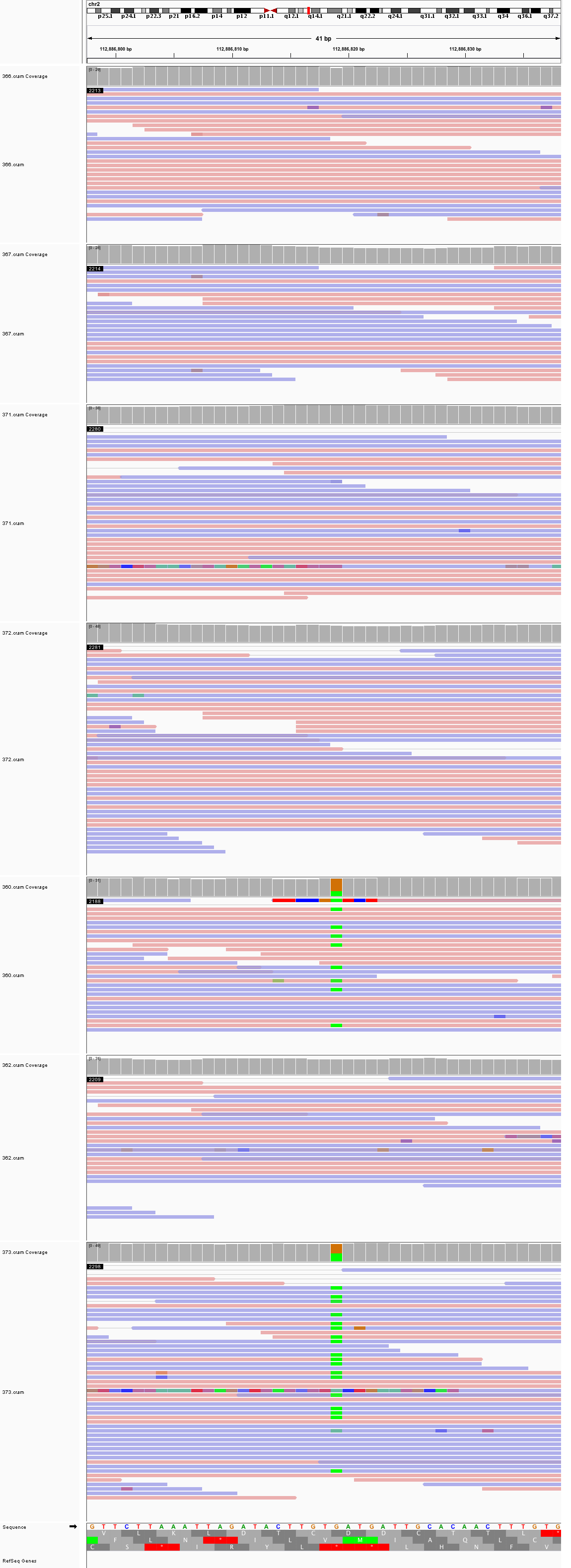

Supplement: Supplementary file 5. — In each image, the first two, three, or four tracks contain alignments from the grandparents in the pedigree (i.e., paternal grandmother and grandfather, maternal grandmother and grandfather). In some families, one or two of the first-generation grandparents were not sequenced (see Supplementary file 1). The two tracks below contain alignments from the second-generation individual with the putative gonosomal mutation and that second-generation individual’s spouse. The remaining tracks below contain alignments from the third-generation individuals that inherited the gonosomal mutation. Reads with mapping quality <20 are filtered out, as they were not considered by our variant calling pipeline, and mismatched bases are shaded by quality score (more transparent = lower base quality). [file elife-46922-supp5.zip › supp_file_5/chr2_112,886,798_112,886,838.png]

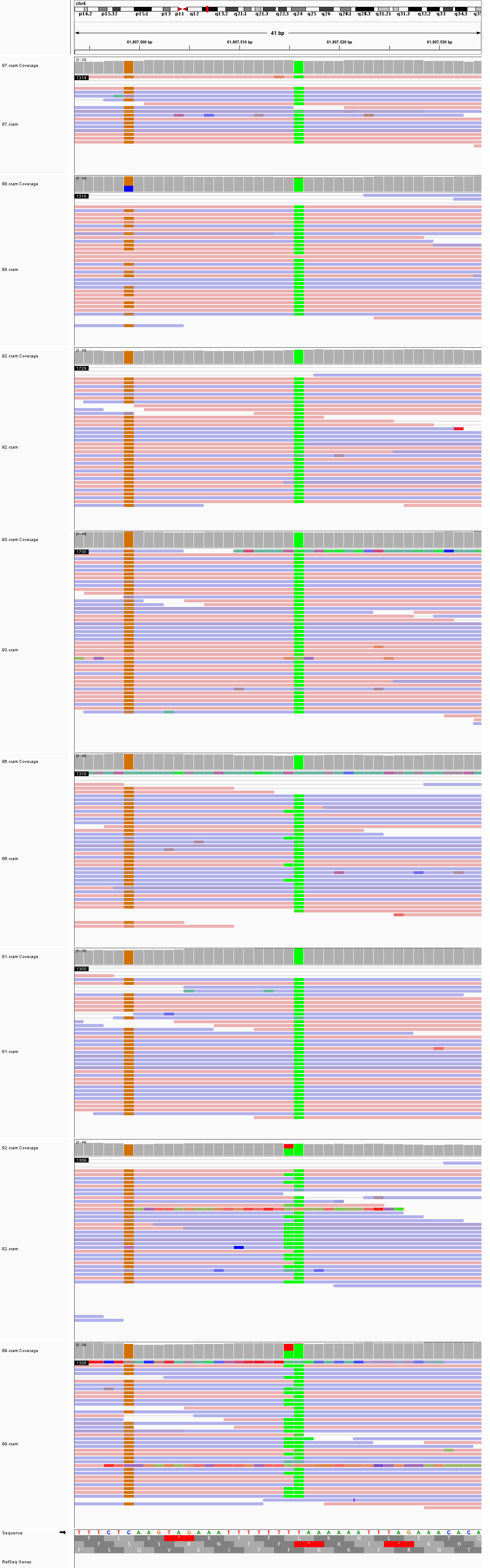

Supplement: Supplementary file 5. — In each image, the first two, three, or four tracks contain alignments from the grandparents in the pedigree (i.e., paternal grandmother and grandfather, maternal grandmother and grandfather). In some families, one or two of the first-generation grandparents were not sequenced (see Supplementary file 1). The two tracks below contain alignments from the second-generation individual with the putative gonosomal mutation and that second-generation individual’s spouse. The remaining tracks below contain alignments from the third-generation individuals that inherited the gonosomal mutation. Reads with mapping quality <20 are filtered out, as they were not considered by our variant calling pipeline, and mismatched bases are shaded by quality score (more transparent = lower base quality). [file elife-46922-supp5.zip › supp_file_5/chr4_61,807,494_61,807,534.png]

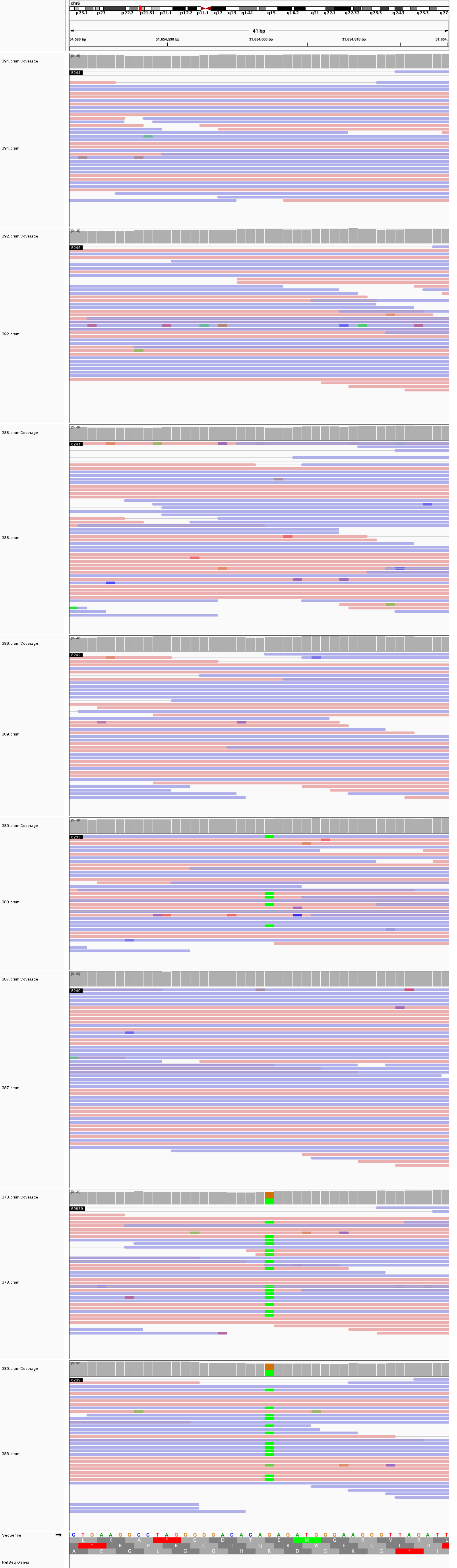

Supplement: Supplementary file 5. — In each image, the first two, three, or four tracks contain alignments from the grandparents in the pedigree (i.e., paternal grandmother and grandfather, maternal grandmother and grandfather). In some families, one or two of the first-generation grandparents were not sequenced (see Supplementary file 1). The two tracks below contain alignments from the second-generation individual with the putative gonosomal mutation and that second-generation individual’s spouse. The remaining tracks below contain alignments from the third-generation individuals that inherited the gonosomal mutation. Reads with mapping quality <20 are filtered out, as they were not considered by our variant calling pipeline, and mismatched bases are shaded by quality score (more transparent = lower base quality). [file elife-46922-supp5.zip › supp_file_5/chr6_31,654,580_31,654,620.png]

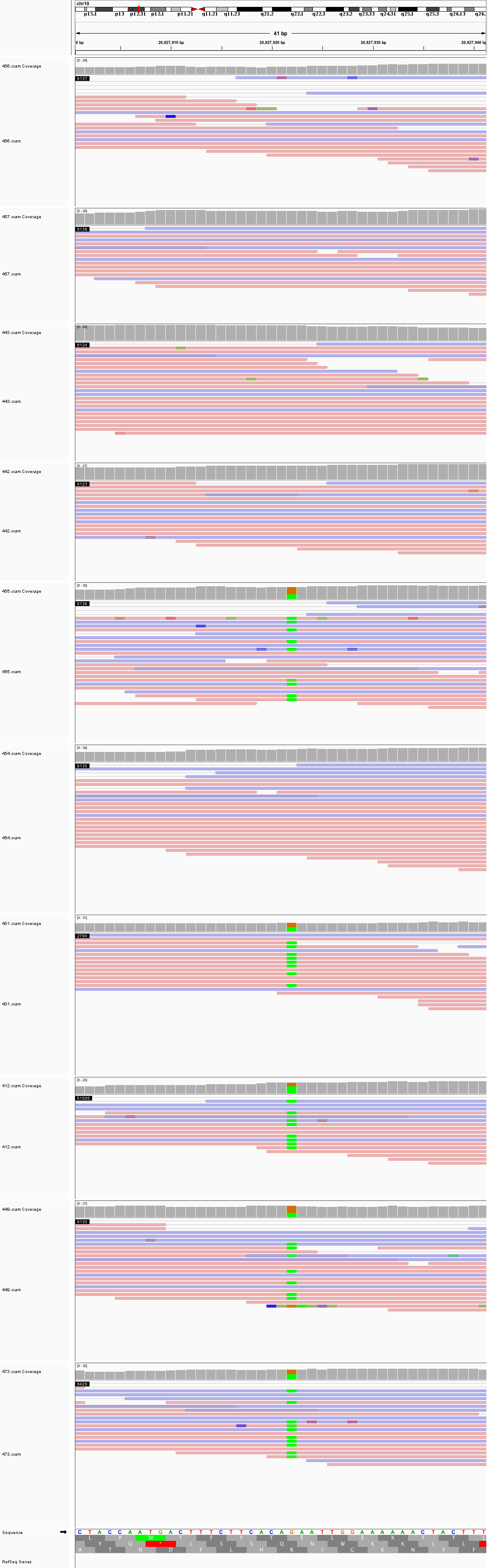

Supplement: Supplementary file 5. — In each image, the first two, three, or four tracks contain alignments from the grandparents in the pedigree (i.e., paternal grandmother and grandfather, maternal grandmother and grandfather). In some families, one or two of the first-generation grandparents were not sequenced (see Supplementary file 1). The two tracks below contain alignments from the second-generation individual with the putative gonosomal mutation and that second-generation individual’s spouse. The remaining tracks below contain alignments from the third-generation individuals that inherited the gonosomal mutation. Reads with mapping quality <20 are filtered out, as they were not considered by our variant calling pipeline, and mismatched bases are shaded by quality score (more transparent = lower base quality). [file elife-46922-supp5.zip › supp_file_5/chr10_20,827,901_20,827,941.png]

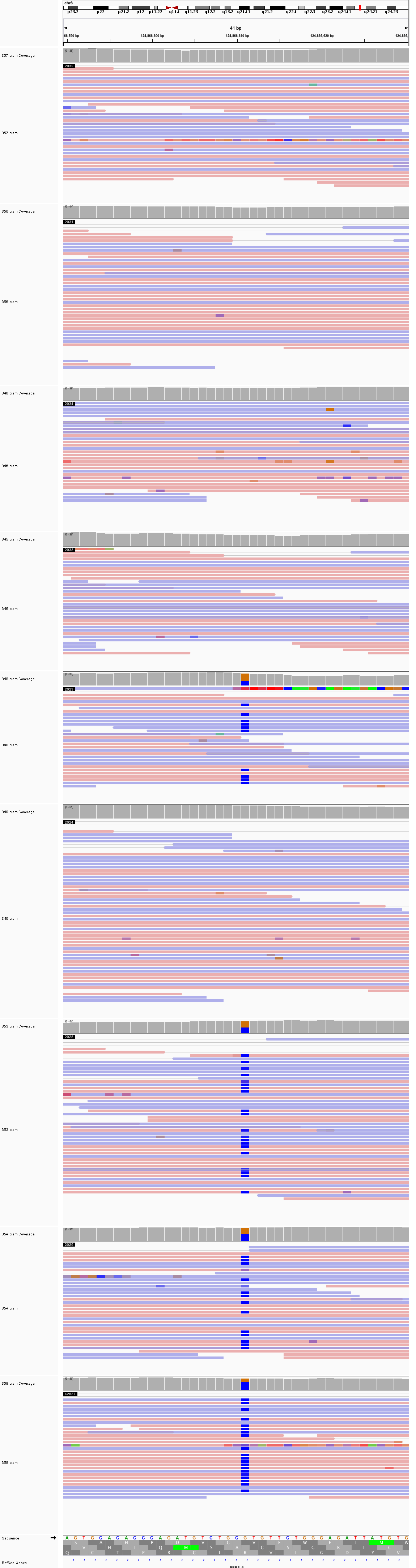

Supplement: Supplementary file 5. — In each image, the first two, three, or four tracks contain alignments from the grandparents in the pedigree (i.e., paternal grandmother and grandfather, maternal grandmother and grandfather). In some families, one or two of the first-generation grandparents were not sequenced (see Supplementary file 1). The two tracks below contain alignments from the second-generation individual with the putative gonosomal mutation and that second-generation individual’s spouse. The remaining tracks below contain alignments from the third-generation individuals that inherited the gonosomal mutation. Reads with mapping quality <20 are filtered out, as they were not considered by our variant calling pipeline, and mismatched bases are shaded by quality score (more transparent = lower base quality). [file elife-46922-supp5.zip › supp_file_5/chr8_124,866,590_124,866,630.png]

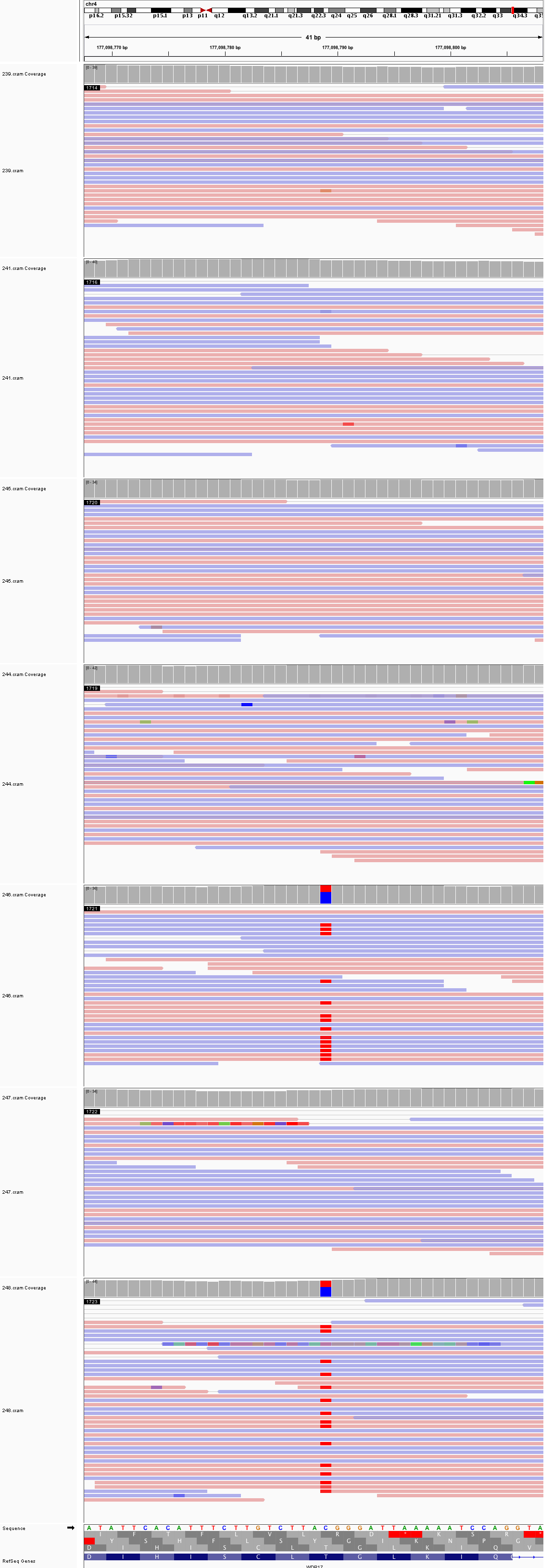

Supplement: Supplementary file 5. — In each image, the first two, three, or four tracks contain alignments from the grandparents in the pedigree (i.e., paternal grandmother and grandfather, maternal grandmother and grandfather). In some families, one or two of the first-generation grandparents were not sequenced (see Supplementary file 1). The two tracks below contain alignments from the second-generation individual with the putative gonosomal mutation and that second-generation individual’s spouse. The remaining tracks below contain alignments from the third-generation individuals that inherited the gonosomal mutation. Reads with mapping quality <20 are filtered out, as they were not considered by our variant calling pipeline, and mismatched bases are shaded by quality score (more transparent = lower base quality). [file elife-46922-supp5.zip › supp_file_5/chr4_177,098,768_177,098,808.png]

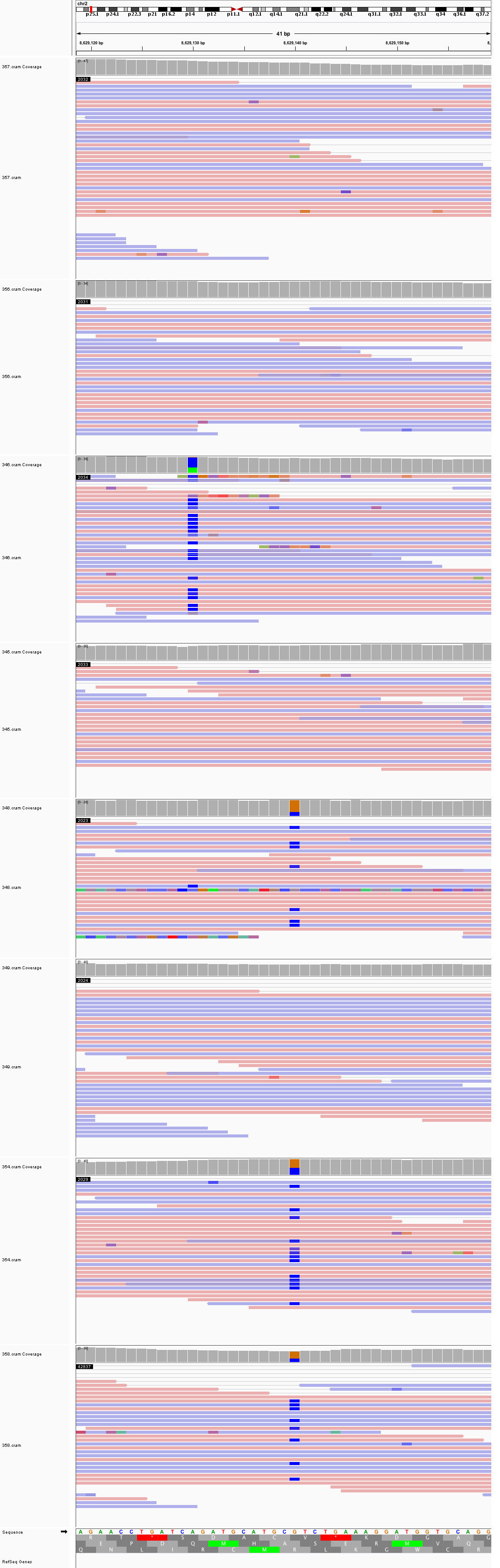

Supplement: Supplementary file 5. — In each image, the first two, three, or four tracks contain alignments from the grandparents in the pedigree (i.e., paternal grandmother and grandfather, maternal grandmother and grandfather). In some families, one or two of the first-generation grandparents were not sequenced (see Supplementary file 1). The two tracks below contain alignments from the second-generation individual with the putative gonosomal mutation and that second-generation individual’s spouse. The remaining tracks below contain alignments from the third-generation individuals that inherited the gonosomal mutation. Reads with mapping quality <20 are filtered out, as they were not considered by our variant calling pipeline, and mismatched bases are shaded by quality score (more transparent = lower base quality). [file elife-46922-supp5.zip › supp_file_5/chr2_8,629,119_8,629,159.png]

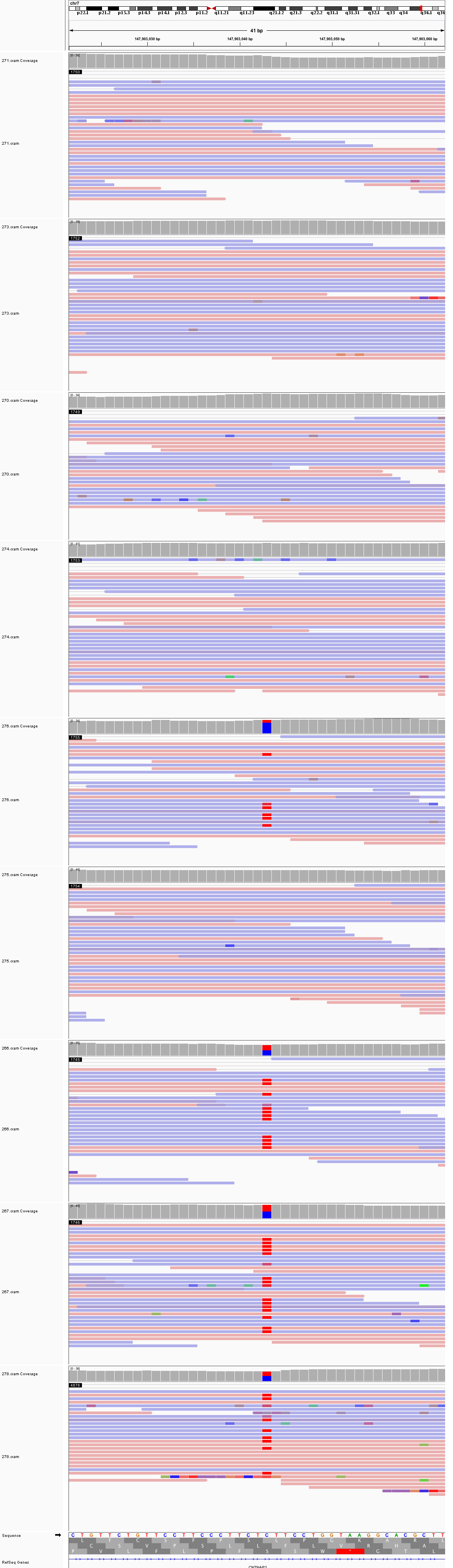

Supplement: Supplementary file 5. — In each image, the first two, three, or four tracks contain alignments from the grandparents in the pedigree (i.e., paternal grandmother and grandfather, maternal grandmother and grandfather). In some families, one or two of the first-generation grandparents were not sequenced (see Supplementary file 1). The two tracks below contain alignments from the second-generation individual with the putative gonosomal mutation and that second-generation individual’s spouse. The remaining tracks below contain alignments from the third-generation individuals that inherited the gonosomal mutation. Reads with mapping quality <20 are filtered out, as they were not considered by our variant calling pipeline, and mismatched bases are shaded by quality score (more transparent = lower base quality). [file elife-46922-supp5.zip › supp_file_5/chr7_147,903,022_147,903,062.png]

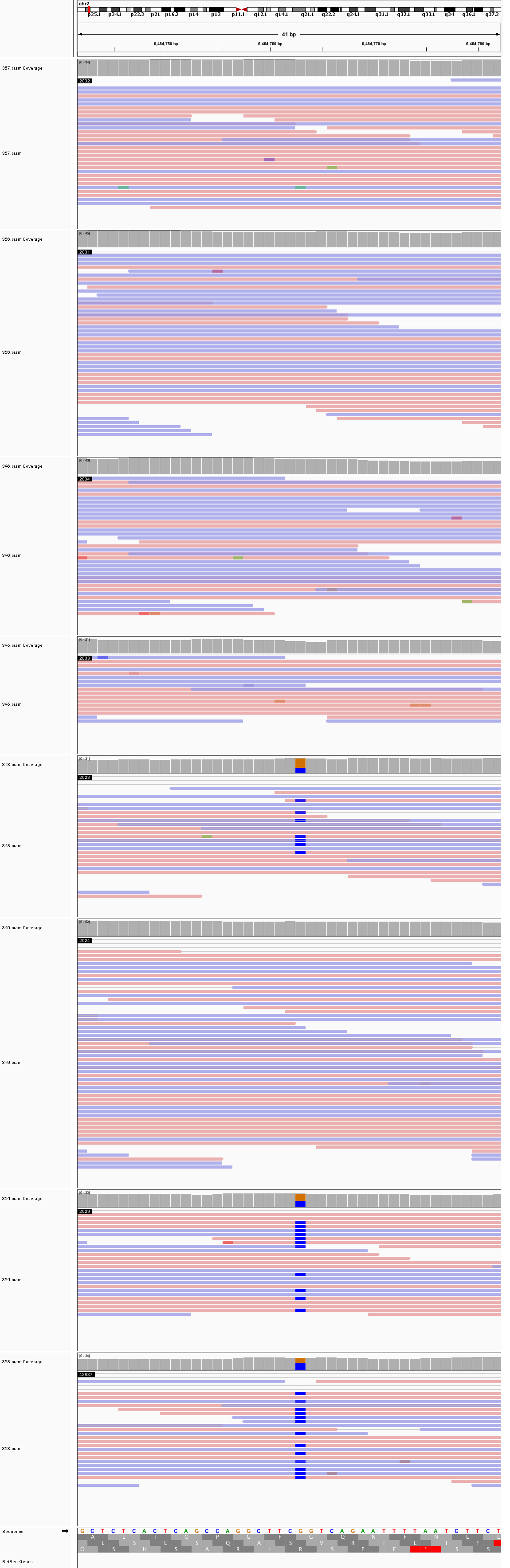

Supplement: Supplementary file 5. — In each image, the first two, three, or four tracks contain alignments from the grandparents in the pedigree (i.e., paternal grandmother and grandfather, maternal grandmother and grandfather). In some families, one or two of the first-generation grandparents were not sequenced (see Supplementary file 1). The two tracks below contain alignments from the second-generation individual with the putative gonosomal mutation and that second-generation individual’s spouse. The remaining tracks below contain alignments from the third-generation individuals that inherited the gonosomal mutation. Reads with mapping quality <20 are filtered out, as they were not considered by our variant calling pipeline, and mismatched bases are shaded by quality score (more transparent = lower base quality). [file elife-46922-supp5.zip › supp_file_5/chr2_6,464,742_6,464,782.png]

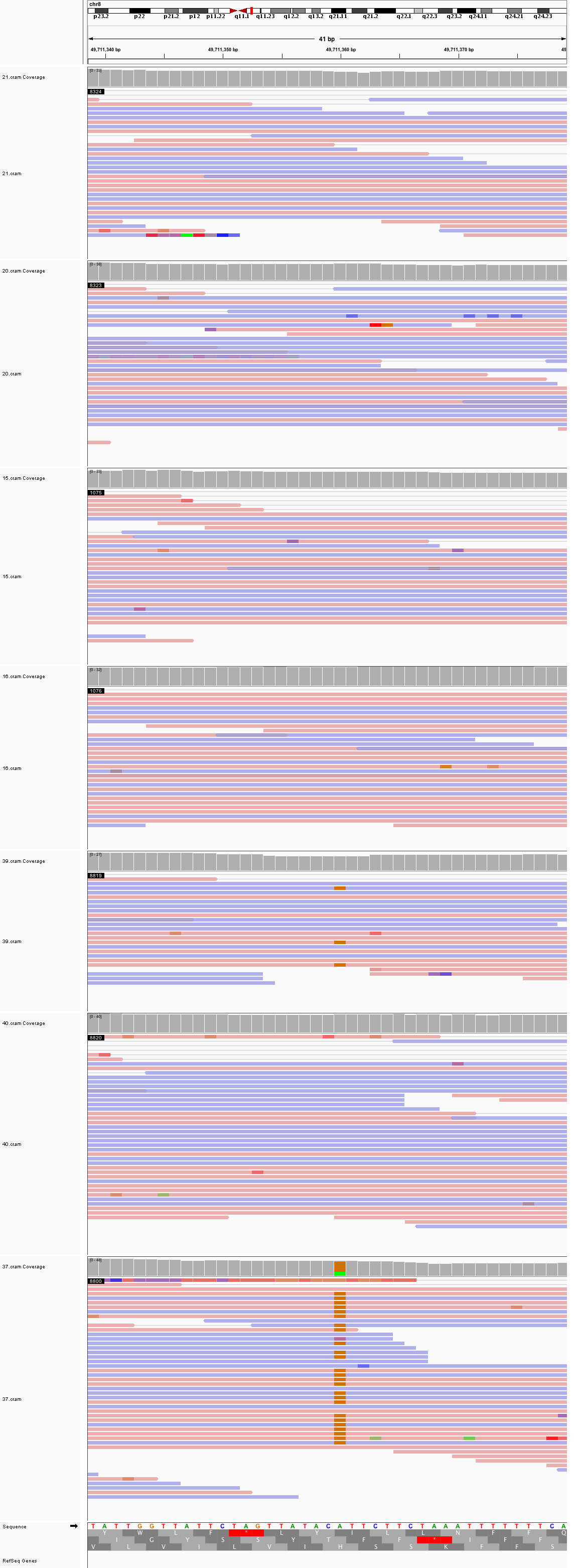

Supplement: Supplementary file 5. — In each image, the first two, three, or four tracks contain alignments from the grandparents in the pedigree (i.e., paternal grandmother and grandfather, maternal grandmother and grandfather). In some families, one or two of the first-generation grandparents were not sequenced (see Supplementary file 1). The two tracks below contain alignments from the second-generation individual with the putative gonosomal mutation and that second-generation individual’s spouse. The remaining tracks below contain alignments from the third-generation individuals that inherited the gonosomal mutation. Reads with mapping quality <20 are filtered out, as they were not considered by our variant calling pipeline, and mismatched bases are shaded by quality score (more transparent = lower base quality). [file elife-46922-supp5.zip › supp_file_5/chr8_49,711,339_49,711,379.png]

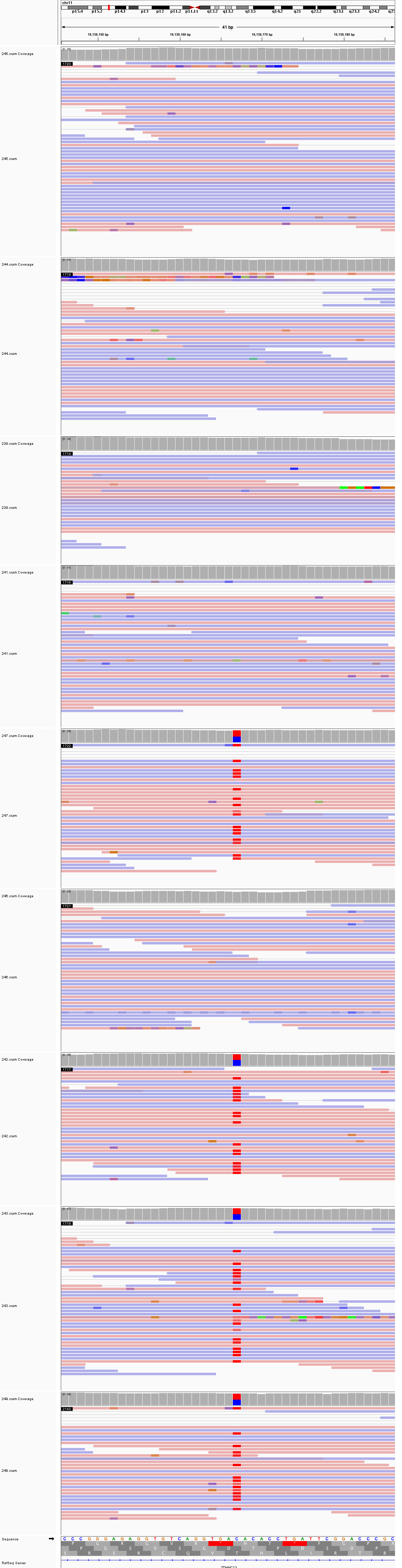

Supplement: Supplementary file 5. — In each image, the first two, three, or four tracks contain alignments from the grandparents in the pedigree (i.e., paternal grandmother and grandfather, maternal grandmother and grandfather). In some families, one or two of the first-generation grandparents were not sequenced (see Supplementary file 1). The two tracks below contain alignments from the second-generation individual with the putative gonosomal mutation and that second-generation individual’s spouse. The remaining tracks below contain alignments from the third-generation individuals that inherited the gonosomal mutation. Reads with mapping quality <20 are filtered out, as they were not considered by our variant calling pipeline, and mismatched bases are shaded by quality score (more transparent = lower base quality). [file elife-46922-supp5.zip › supp_file_5/chr11_19,139,146_19,139,186.png]

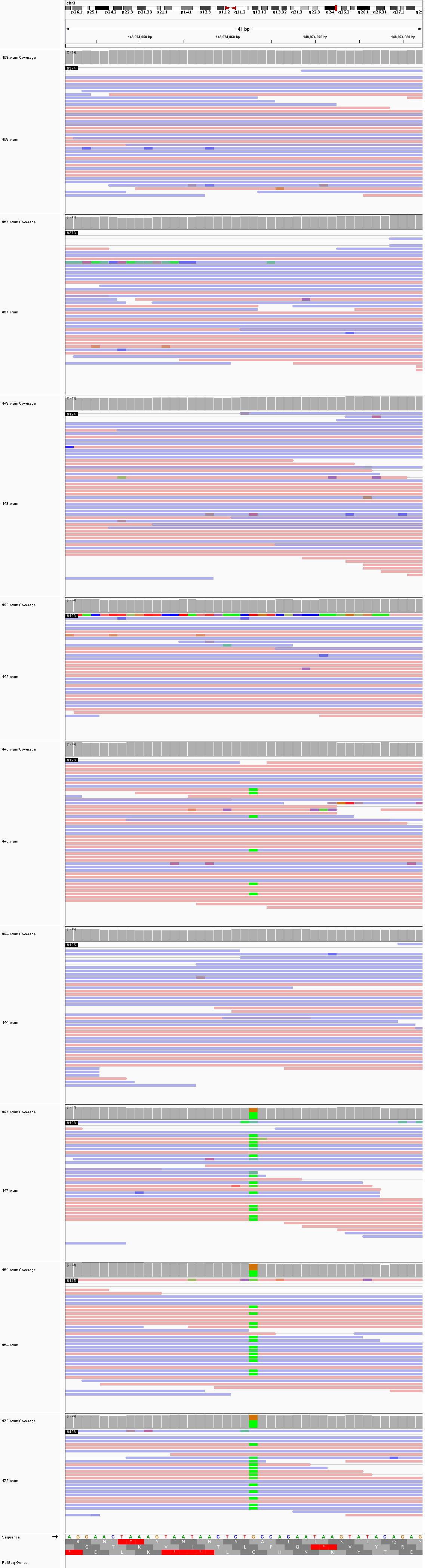

Supplement: Supplementary file 5. — In each image, the first two, three, or four tracks contain alignments from the grandparents in the pedigree (i.e., paternal grandmother and grandfather, maternal grandmother and grandfather). In some families, one or two of the first-generation grandparents were not sequenced (see Supplementary file 1). The two tracks below contain alignments from the second-generation individual with the putative gonosomal mutation and that second-generation individual’s spouse. The remaining tracks below contain alignments from the third-generation individuals that inherited the gonosomal mutation. Reads with mapping quality <20 are filtered out, as they were not considered by our variant calling pipeline, and mismatched bases are shaded by quality score (more transparent = lower base quality). [file elife-46922-supp5.zip › supp_file_5/chr3_148,974,042_148,974,082.png]

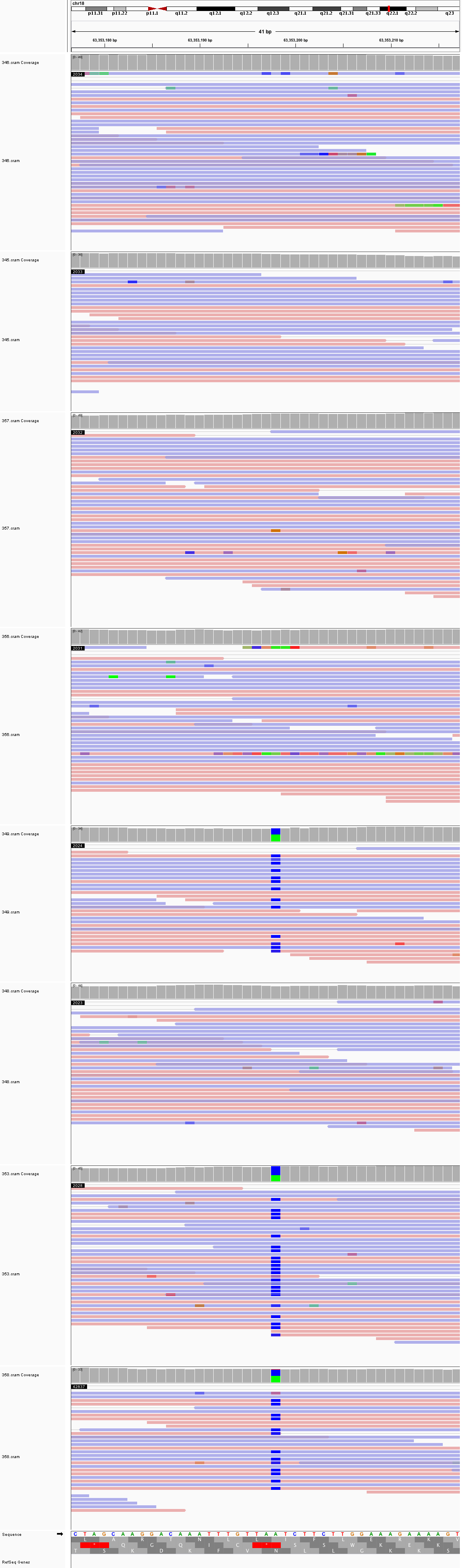

Supplement: Supplementary file 5. — In each image, the first two, three, or four tracks contain alignments from the grandparents in the pedigree (i.e., paternal grandmother and grandfather, maternal grandmother and grandfather). In some families, one or two of the first-generation grandparents were not sequenced (see Supplementary file 1). The two tracks below contain alignments from the second-generation individual with the putative gonosomal mutation and that second-generation individual’s spouse. The remaining tracks below contain alignments from the third-generation individuals that inherited the gonosomal mutation. Reads with mapping quality <20 are filtered out, as they were not considered by our variant calling pipeline, and mismatched bases are shaded by quality score (more transparent = lower base quality). [file elife-46922-supp5.zip › supp_file_5/chr18_63,353,177_63,353,217.png]

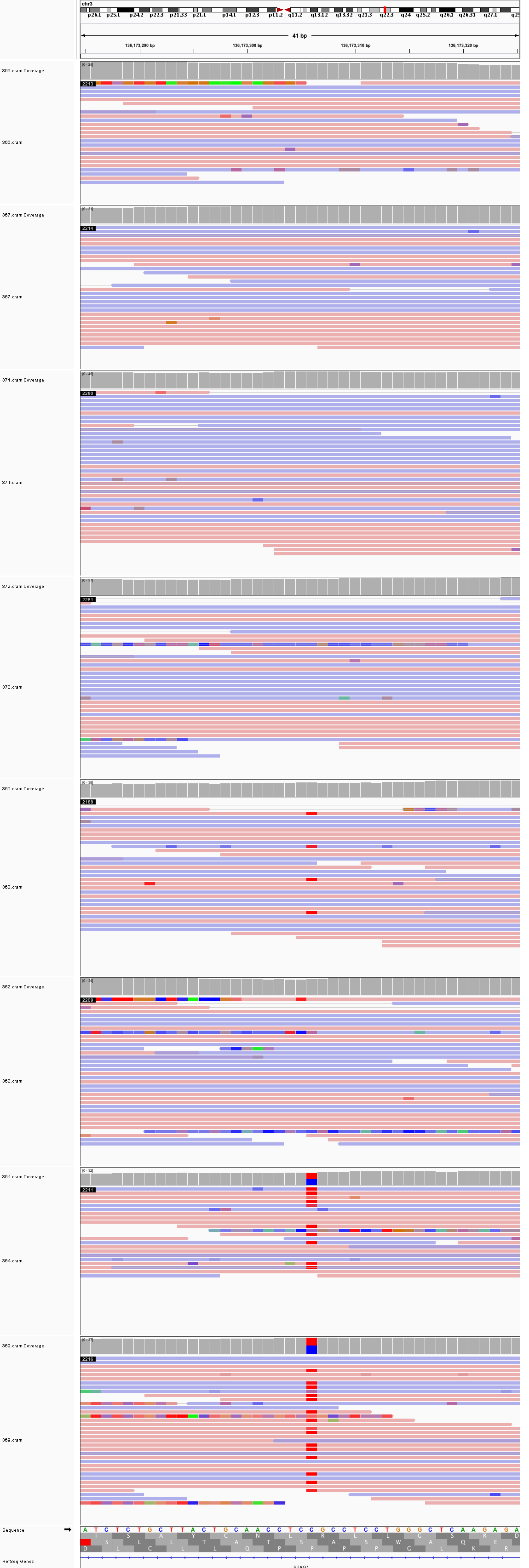

Supplement: Supplementary file 5. — In each image, the first two, three, or four tracks contain alignments from the grandparents in the pedigree (i.e., paternal grandmother and grandfather, maternal grandmother and grandfather). In some families, one or two of the first-generation grandparents were not sequenced (see Supplementary file 1). The two tracks below contain alignments from the second-generation individual with the putative gonosomal mutation and that second-generation individual’s spouse. The remaining tracks below contain alignments from the third-generation individuals that inherited the gonosomal mutation. Reads with mapping quality <20 are filtered out, as they were not considered by our variant calling pipeline, and mismatched bases are shaded by quality score (more transparent = lower base quality). [file elife-46922-supp5.zip › supp_file_5/chr3_136,173,285_136,173,325.png]

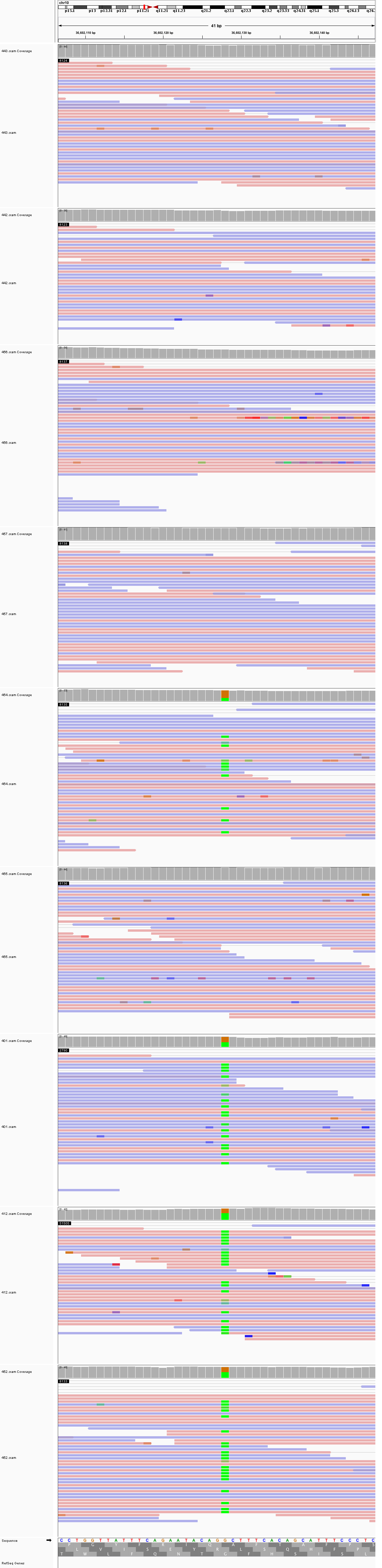

Supplement: Supplementary file 5. — In each image, the first two, three, or four tracks contain alignments from the grandparents in the pedigree (i.e., paternal grandmother and grandfather, maternal grandmother and grandfather). In some families, one or two of the first-generation grandparents were not sequenced (see Supplementary file 1). The two tracks below contain alignments from the second-generation individual with the putative gonosomal mutation and that second-generation individual’s spouse. The remaining tracks below contain alignments from the third-generation individuals that inherited the gonosomal mutation. Reads with mapping quality <20 are filtered out, as they were not considered by our variant calling pipeline, and mismatched bases are shaded by quality score (more transparent = lower base quality). [file elife-46922-supp5.zip › supp_file_5/chr10_36,602,107_36,602,147.png]

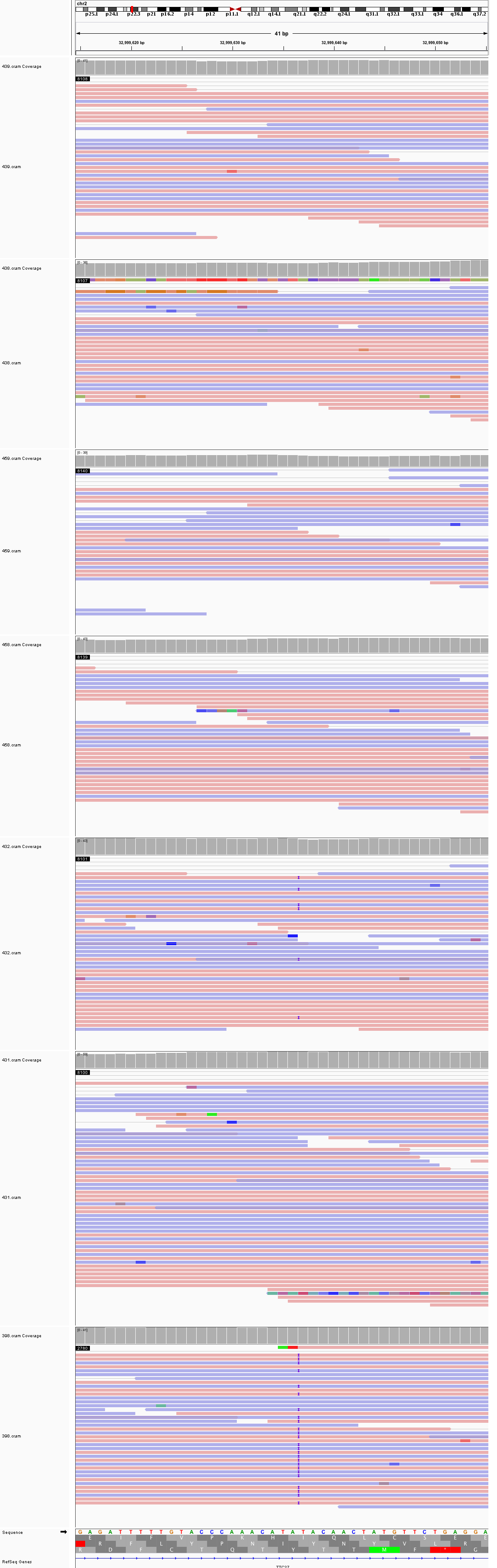

Supplement: Supplementary file 5. — In each image, the first two, three, or four tracks contain alignments from the grandparents in the pedigree (i.e., paternal grandmother and grandfather, maternal grandmother and grandfather). In some families, one or two of the first-generation grandparents were not sequenced (see Supplementary file 1). The two tracks below contain alignments from the second-generation individual with the putative gonosomal mutation and that second-generation individual’s spouse. The remaining tracks below contain alignments from the third-generation individuals that inherited the gonosomal mutation. Reads with mapping quality <20 are filtered out, as they were not considered by our variant calling pipeline, and mismatched bases are shaded by quality score (more transparent = lower base quality). [file elife-46922-supp5.zip › supp_file_5/chr2_32,999,615_32,999,655.png]

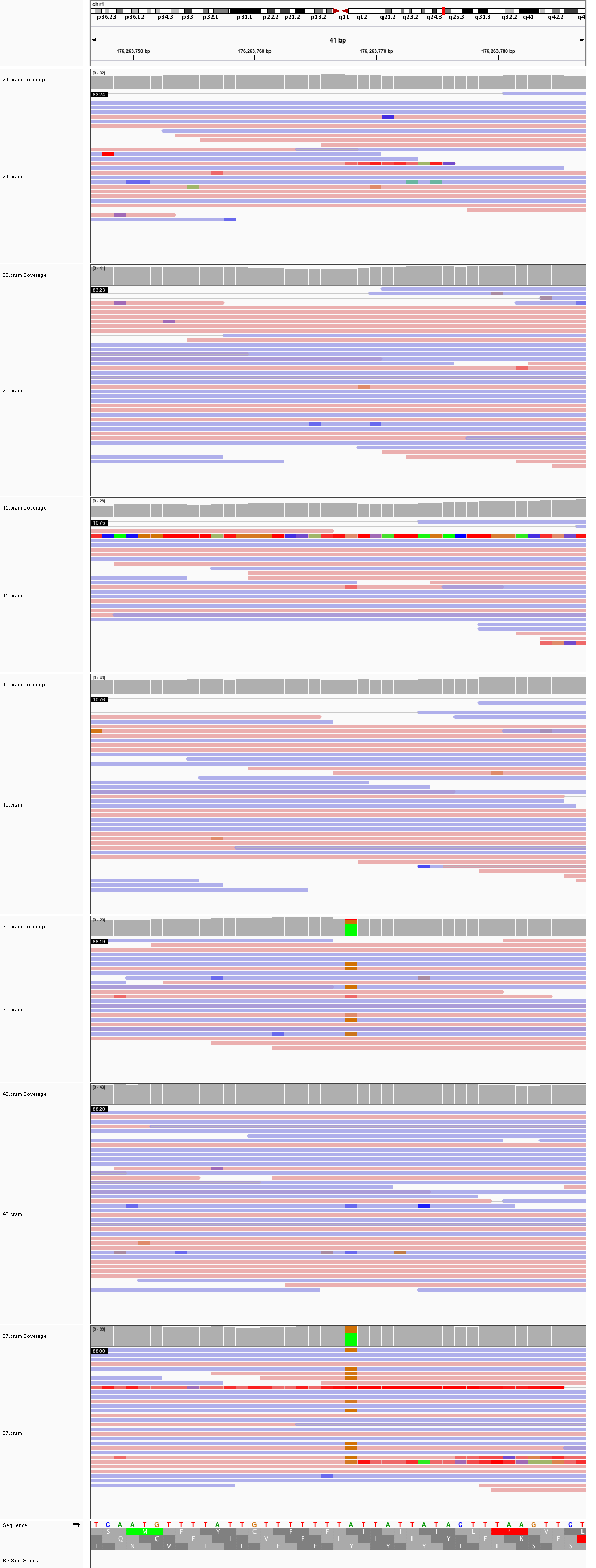

Supplement: Supplementary file 5. — In each image, the first two, three, or four tracks contain alignments from the grandparents in the pedigree (i.e., paternal grandmother and grandfather, maternal grandmother and grandfather). In some families, one or two of the first-generation grandparents were not sequenced (see Supplementary file 1). The two tracks below contain alignments from the second-generation individual with the putative gonosomal mutation and that second-generation individual’s spouse. The remaining tracks below contain alignments from the third-generation individuals that inherited the gonosomal mutation. Reads with mapping quality <20 are filtered out, as they were not considered by our variant calling pipeline, and mismatched bases are shaded by quality score (more transparent = lower base quality). [file elife-46922-supp5.zip › supp_file_5/chr1_176,263,747_176,263,787.png]

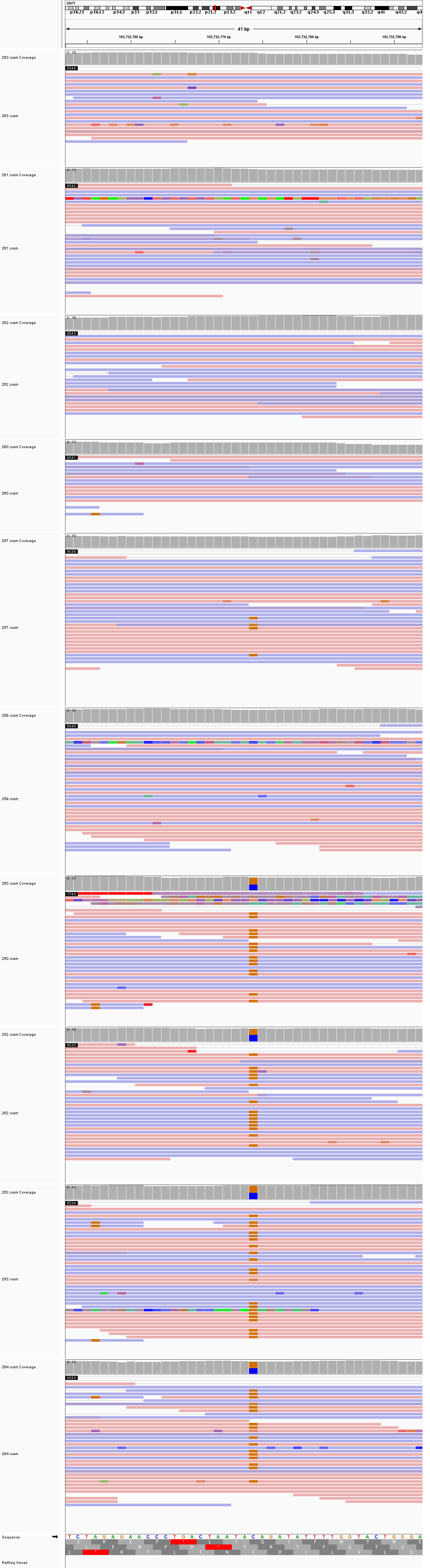

Supplement: Supplementary file 5. — In each image, the first two, three, or four tracks contain alignments from the grandparents in the pedigree (i.e., paternal grandmother and grandfather, maternal grandmother and grandfather). In some families, one or two of the first-generation grandparents were not sequenced (see Supplementary file 1). The two tracks below contain alignments from the second-generation individual with the putative gonosomal mutation and that second-generation individual’s spouse. The remaining tracks below contain alignments from the third-generation individuals that inherited the gonosomal mutation. Reads with mapping quality <20 are filtered out, as they were not considered by our variant calling pipeline, and mismatched bases are shaded by quality score (more transparent = lower base quality). [file elife-46922-supp5.zip › supp_file_5/chr1_103,732,753_103,732,793.png]

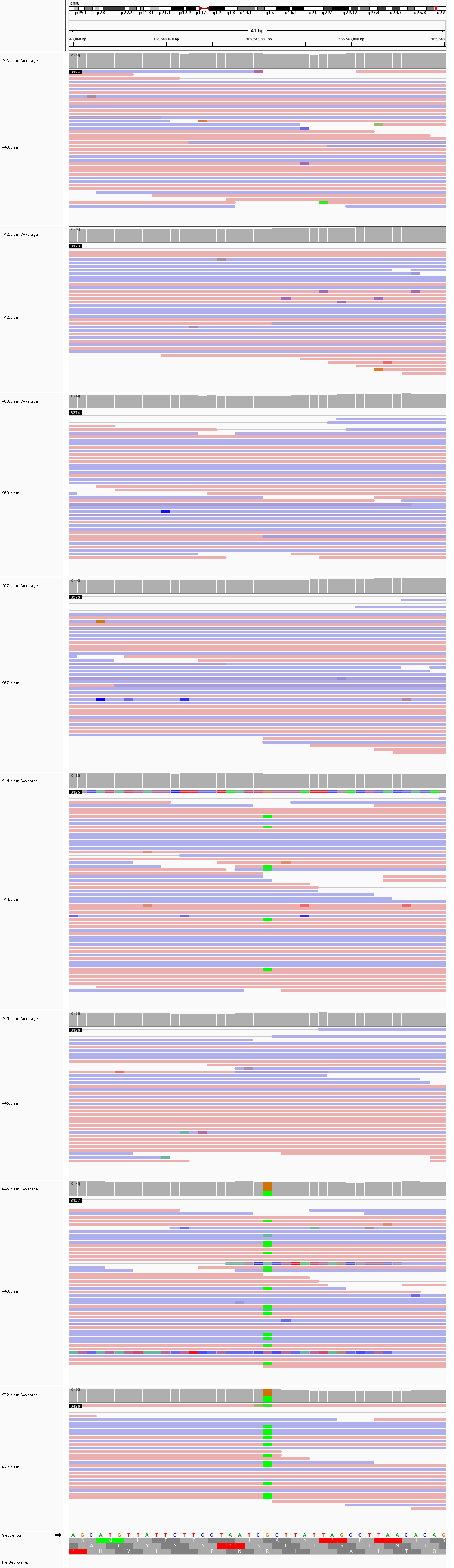

Supplement: Supplementary file 5. — In each image, the first two, three, or four tracks contain alignments from the grandparents in the pedigree (i.e., paternal grandmother and grandfather, maternal grandmother and grandfather). In some families, one or two of the first-generation grandparents were not sequenced (see Supplementary file 1). The two tracks below contain alignments from the second-generation individual with the putative gonosomal mutation and that second-generation individual’s spouse. The remaining tracks below contain alignments from the third-generation individuals that inherited the gonosomal mutation. Reads with mapping quality <20 are filtered out, as they were not considered by our variant calling pipeline, and mismatched bases are shaded by quality score (more transparent = lower base quality). [file elife-46922-supp5.zip › supp_file_5/chr6_165,543,860_165,543,900.png]

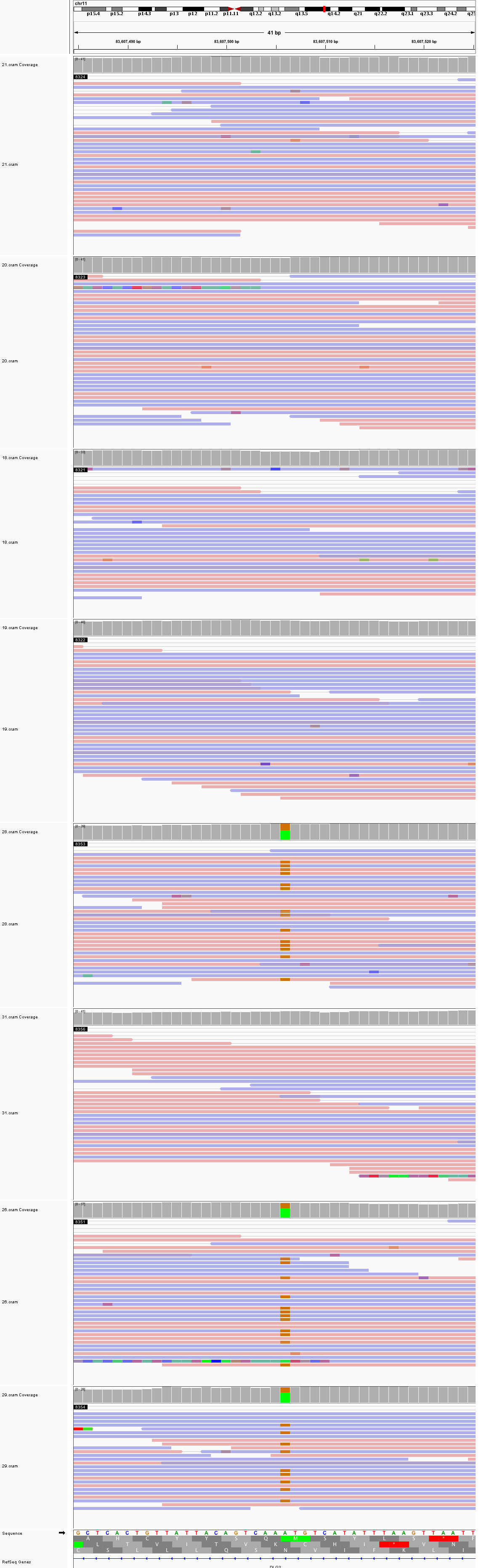

Supplement: Supplementary file 5. — In each image, the first two, three, or four tracks contain alignments from the grandparents in the pedigree (i.e., paternal grandmother and grandfather, maternal grandmother and grandfather). In some families, one or two of the first-generation grandparents were not sequenced (see Supplementary file 1). The two tracks below contain alignments from the second-generation individual with the putative gonosomal mutation and that second-generation individual’s spouse. The remaining tracks below contain alignments from the third-generation individuals that inherited the gonosomal mutation. Reads with mapping quality <20 are filtered out, as they were not considered by our variant calling pipeline, and mismatched bases are shaded by quality score (more transparent = lower base quality). [file elife-46922-supp5.zip › supp_file_5/chr11_83,607,485_83,607,525.png]

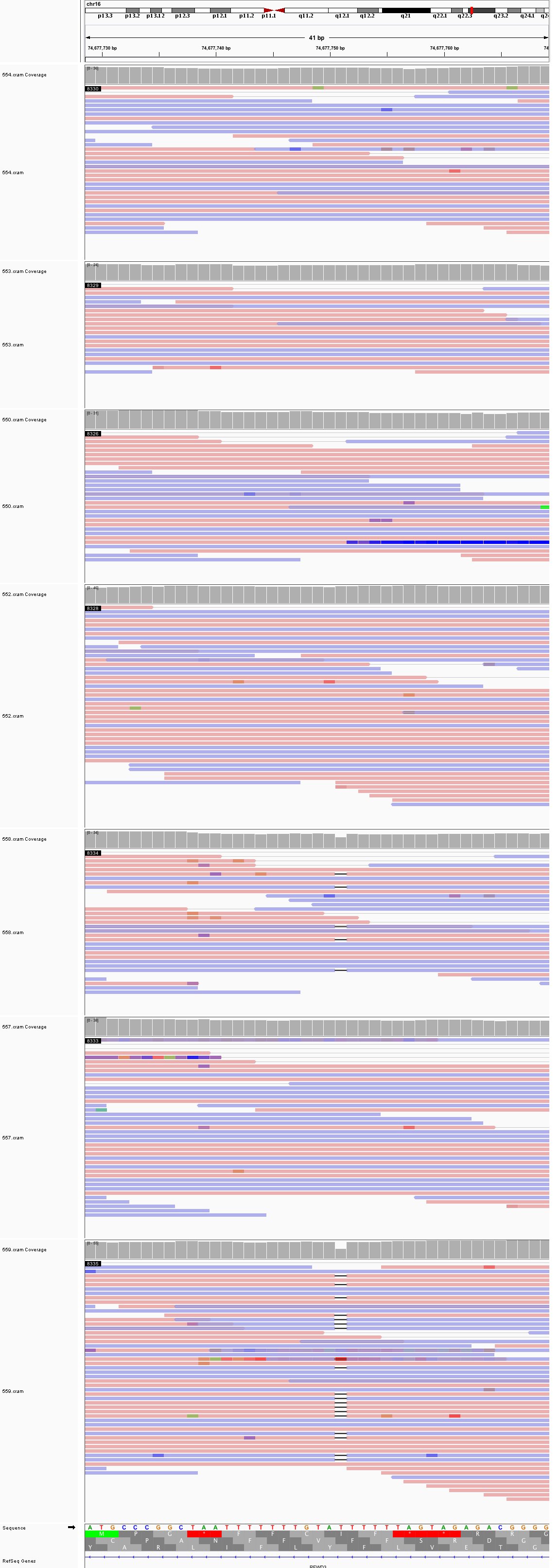

Supplement: Supplementary file 5. — In each image, the first two, three, or four tracks contain alignments from the grandparents in the pedigree (i.e., paternal grandmother and grandfather, maternal grandmother and grandfather). In some families, one or two of the first-generation grandparents were not sequenced (see Supplementary file 1). The two tracks below contain alignments from the second-generation individual with the putative gonosomal mutation and that second-generation individual’s spouse. The remaining tracks below contain alignments from the third-generation individuals that inherited the gonosomal mutation. Reads with mapping quality <20 are filtered out, as they were not considered by our variant calling pipeline, and mismatched bases are shaded by quality score (more transparent = lower base quality). [file elife-46922-supp5.zip › supp_file_5/chr16_74,677,729_74,677,769.png]

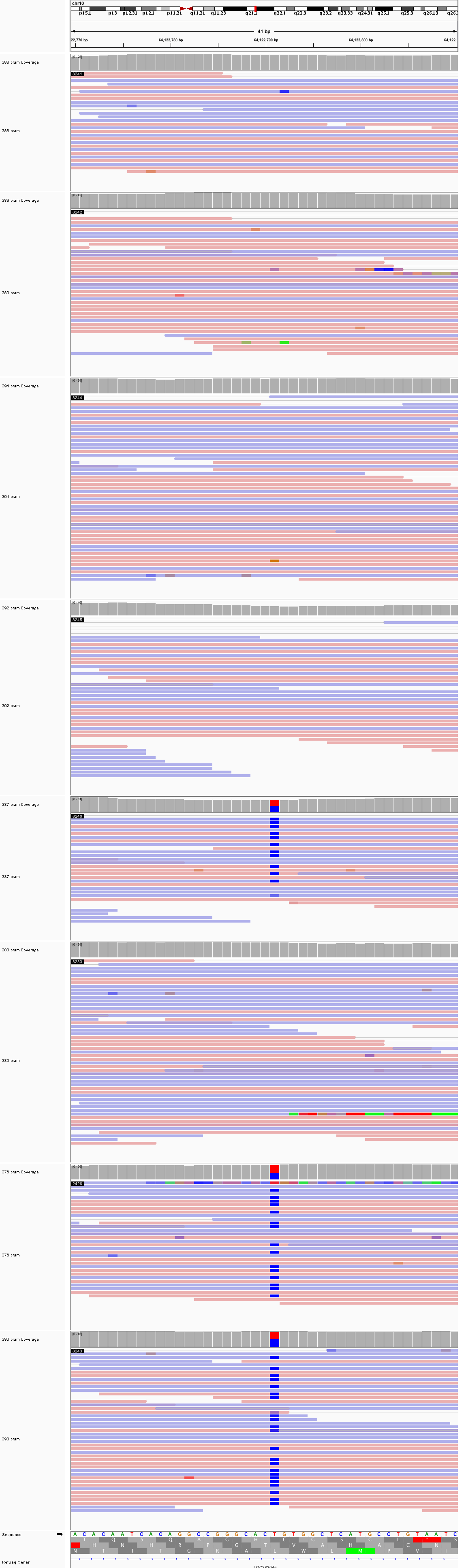

Supplement: Supplementary file 5. — In each image, the first two, three, or four tracks contain alignments from the grandparents in the pedigree (i.e., paternal grandmother and grandfather, maternal grandmother and grandfather). In some families, one or two of the first-generation grandparents were not sequenced (see Supplementary file 1). The two tracks below contain alignments from the second-generation individual with the putative gonosomal mutation and that second-generation individual’s spouse. The remaining tracks below contain alignments from the third-generation individuals that inherited the gonosomal mutation. Reads with mapping quality <20 are filtered out, as they were not considered by our variant calling pipeline, and mismatched bases are shaded by quality score (more transparent = lower base quality). [file elife-46922-supp5.zip › supp_file_5/chr10_64,122,770_64,122,810.png]
