# Supplementary figures and images for: Large, three-generation human families reveal post-zygotic mosaicism and variability in germline mutation accumulation (part 4 of 7)
Source: eLife. 2019 Sep 24;8:e46922. doi: 10.7554/eLife.46922 (PMC6759356; doi:10.7554/eLife.46922)

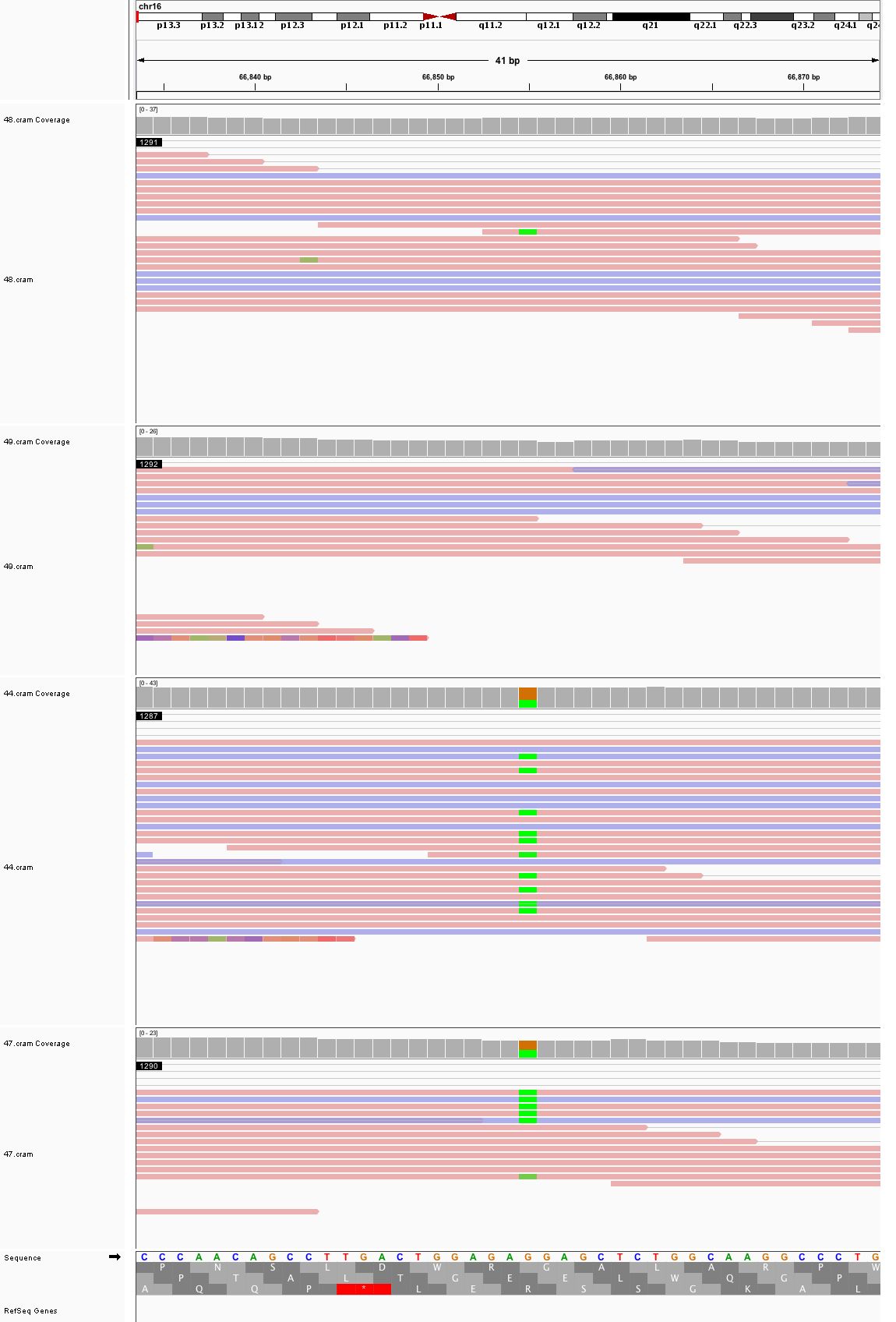

Supplement: Supplementary file 4. — All tracks below contain alignments from the third-generation children that share a DNM at the site. Reads with mapping quality <20 are filtered out, as they were not considered by our variant calling pipeline, and mismatched bases are shaded by quality score (more transparent = lower base quality). [file elife-46922-supp4.zip › supp_file_4/chr16_66,834_66,874.png]

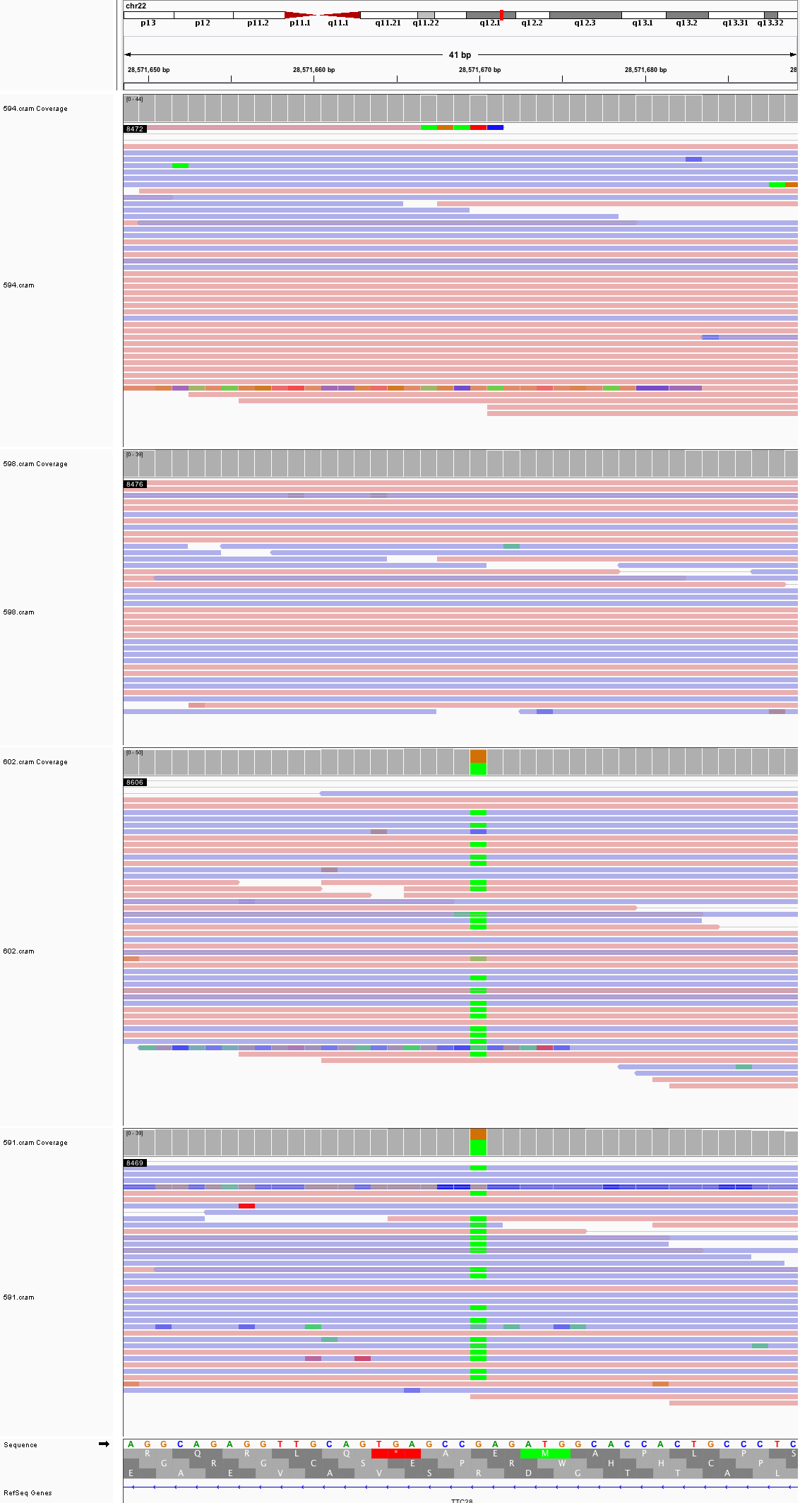

Supplement: Supplementary file 4. — All tracks below contain alignments from the third-generation children that share a DNM at the site. Reads with mapping quality <20 are filtered out, as they were not considered by our variant calling pipeline, and mismatched bases are shaded by quality score (more transparent = lower base quality). [file elife-46922-supp4.zip › supp_file_4/chr22_28,571,649_28,571,689.png]

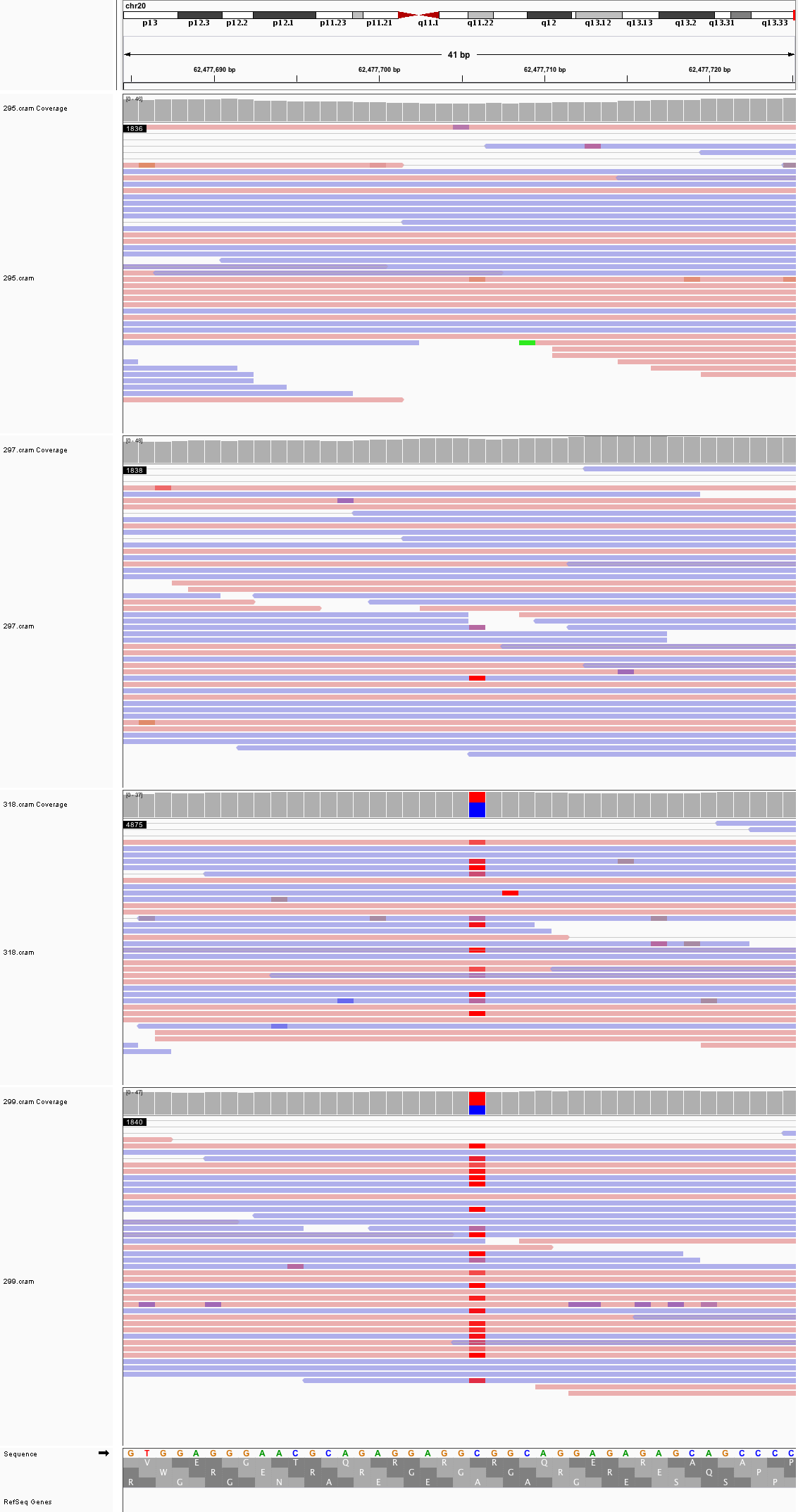

Supplement: Supplementary file 4. — All tracks below contain alignments from the third-generation children that share a DNM at the site. Reads with mapping quality <20 are filtered out, as they were not considered by our variant calling pipeline, and mismatched bases are shaded by quality score (more transparent = lower base quality). [file elife-46922-supp4.zip › supp_file_4/chr20_62,477,685_62,477,725.png]

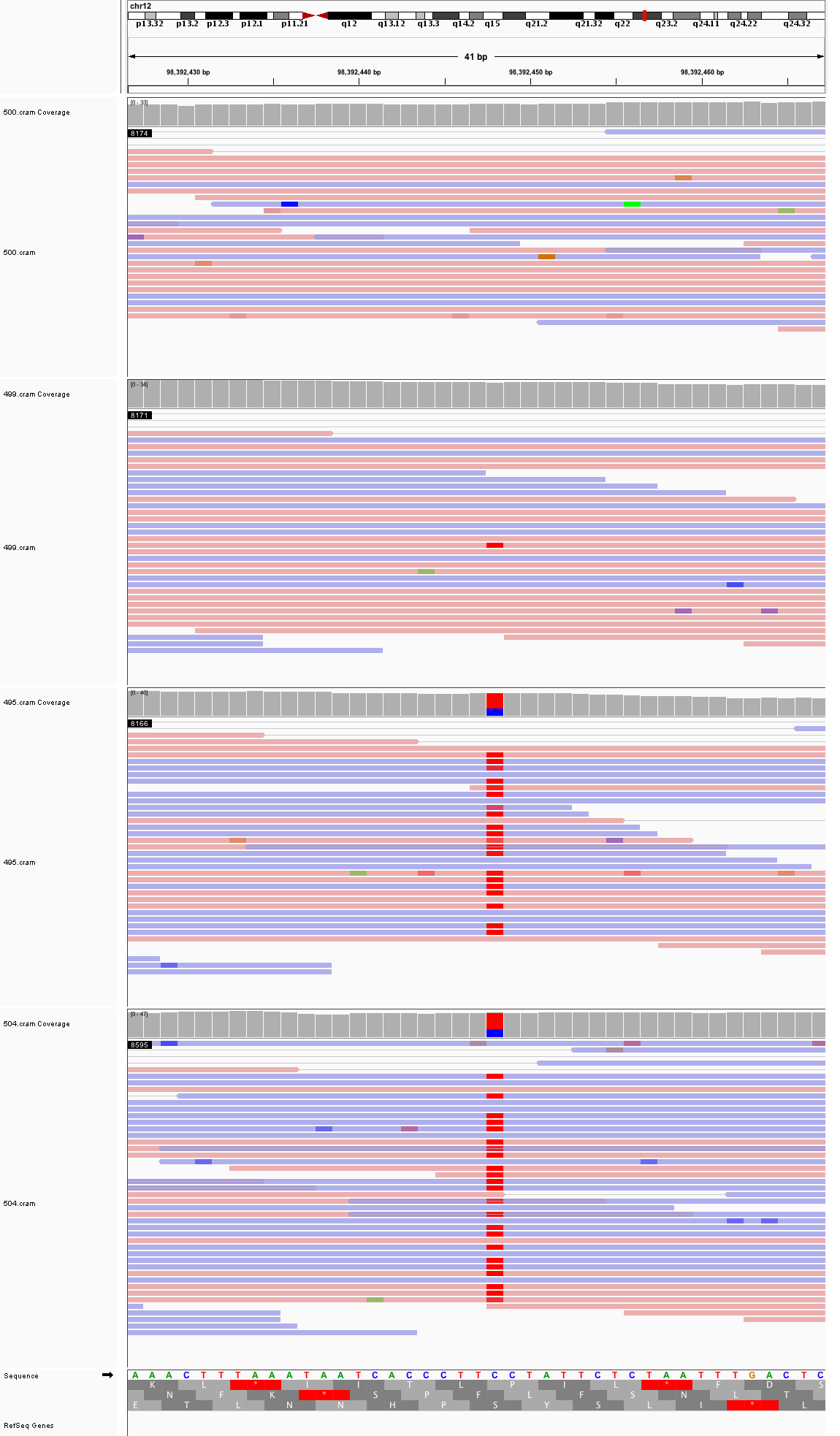

Supplement: Supplementary file 4. — All tracks below contain alignments from the third-generation children that share a DNM at the site. Reads with mapping quality <20 are filtered out, as they were not considered by our variant calling pipeline, and mismatched bases are shaded by quality score (more transparent = lower base quality). [file elife-46922-supp4.zip › supp_file_4/chr12_98,392,427_98,392,467.png]

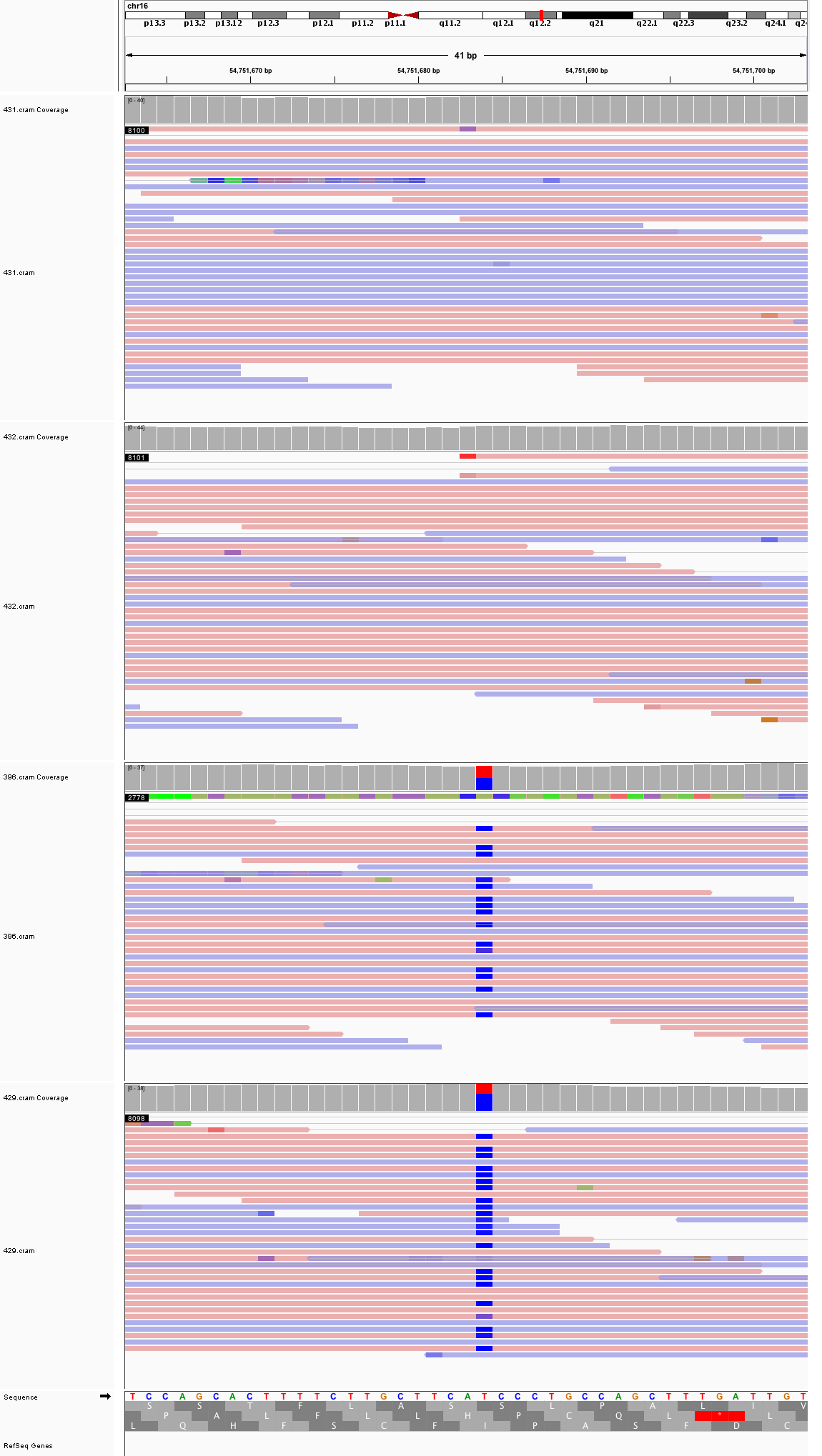

Supplement: Supplementary file 4. — All tracks below contain alignments from the third-generation children that share a DNM at the site. Reads with mapping quality <20 are filtered out, as they were not considered by our variant calling pipeline, and mismatched bases are shaded by quality score (more transparent = lower base quality). [file elife-46922-supp4.zip › supp_file_4/chr16_54,751,663_54,751,703.png]

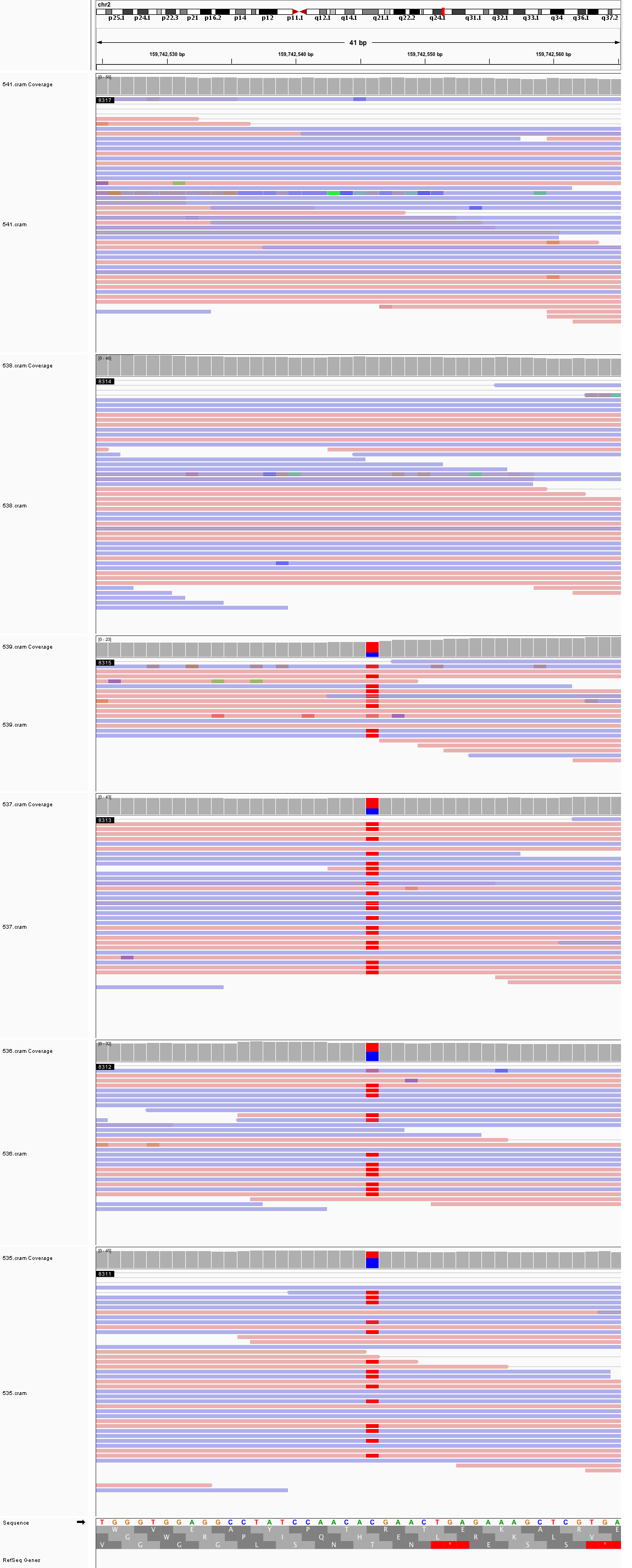

Supplement: Supplementary file 4. — All tracks below contain alignments from the third-generation children that share a DNM at the site. Reads with mapping quality <20 are filtered out, as they were not considered by our variant calling pipeline, and mismatched bases are shaded by quality score (more transparent = lower base quality). [file elife-46922-supp4.zip › supp_file_4/chr2_159,742,525_159,742,565.png]

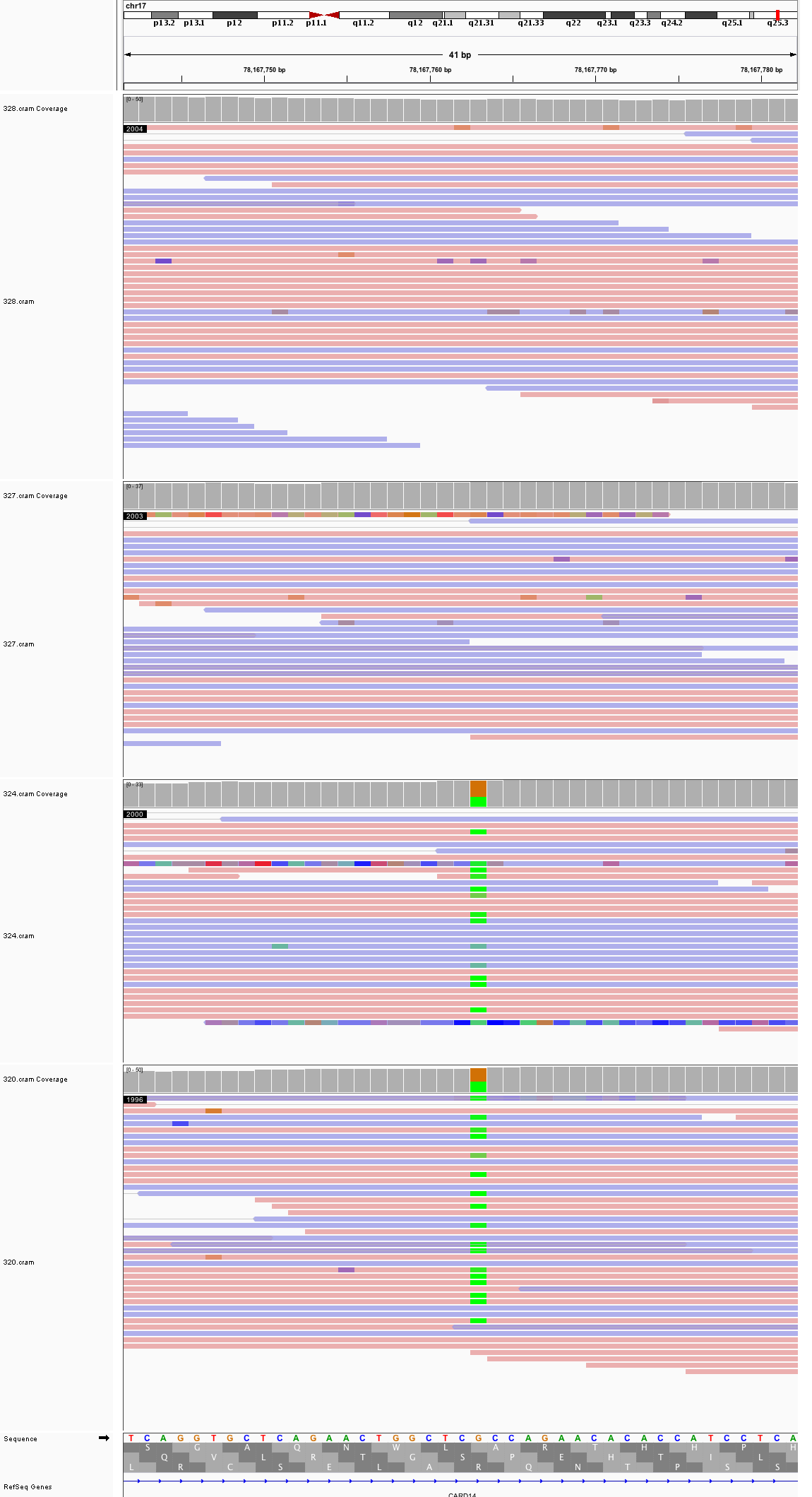

Supplement: Supplementary file 4. — All tracks below contain alignments from the third-generation children that share a DNM at the site. Reads with mapping quality <20 are filtered out, as they were not considered by our variant calling pipeline, and mismatched bases are shaded by quality score (more transparent = lower base quality). [file elife-46922-supp4.zip › supp_file_4/chr17_78,167,742_78,167,782.png]

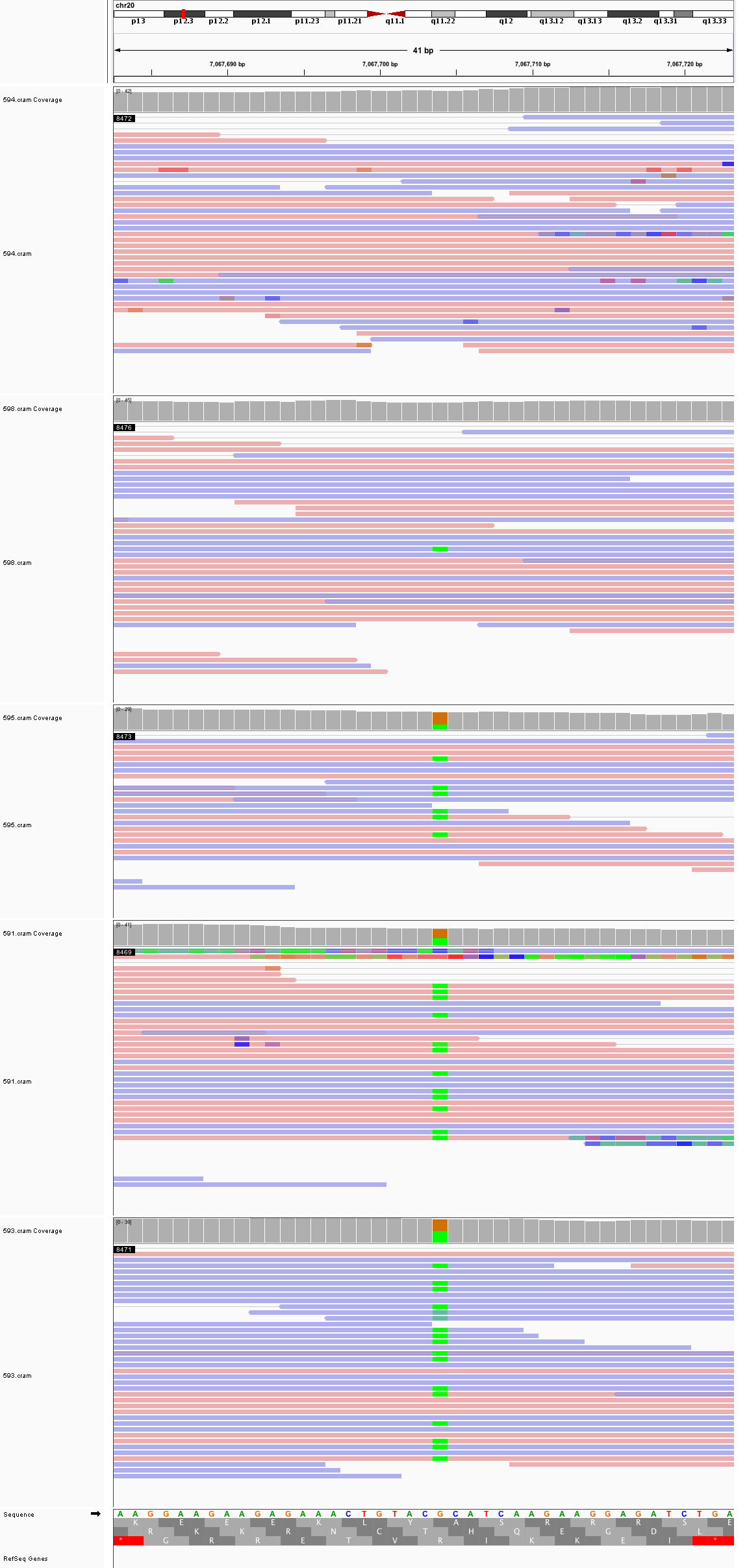

Supplement: Supplementary file 4. — All tracks below contain alignments from the third-generation children that share a DNM at the site. Reads with mapping quality <20 are filtered out, as they were not considered by our variant calling pipeline, and mismatched bases are shaded by quality score (more transparent = lower base quality). [file elife-46922-supp4.zip › supp_file_4/chr20_7,067,683_7,067,723.png]

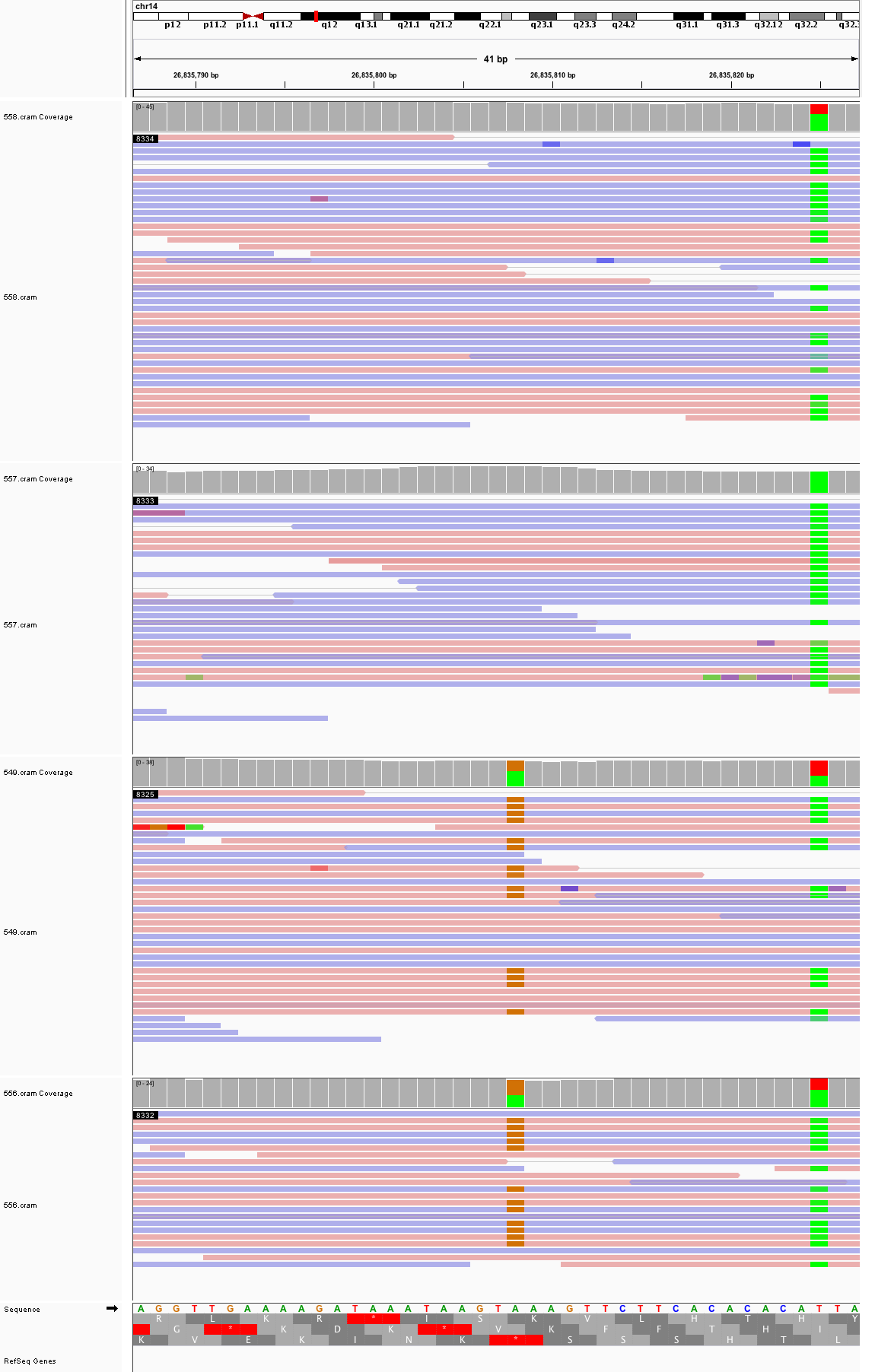

Supplement: Supplementary file 4. — All tracks below contain alignments from the third-generation children that share a DNM at the site. Reads with mapping quality <20 are filtered out, as they were not considered by our variant calling pipeline, and mismatched bases are shaded by quality score (more transparent = lower base quality). [file elife-46922-supp4.zip › supp_file_4/chr14_26,835,787_26,835,827.png]

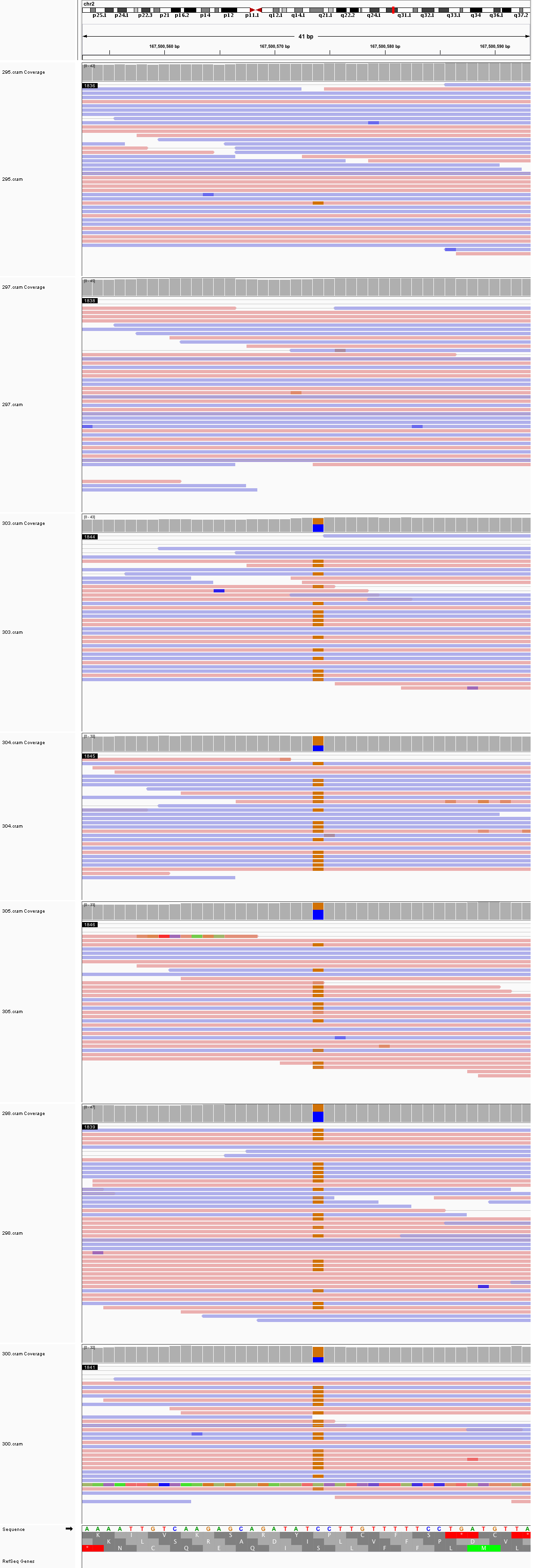

Supplement: Supplementary file 4. — All tracks below contain alignments from the third-generation children that share a DNM at the site. Reads with mapping quality <20 are filtered out, as they were not considered by our variant calling pipeline, and mismatched bases are shaded by quality score (more transparent = lower base quality). [file elife-46922-supp4.zip › supp_file_4/chr2_167,500,553_167,500,593.png]

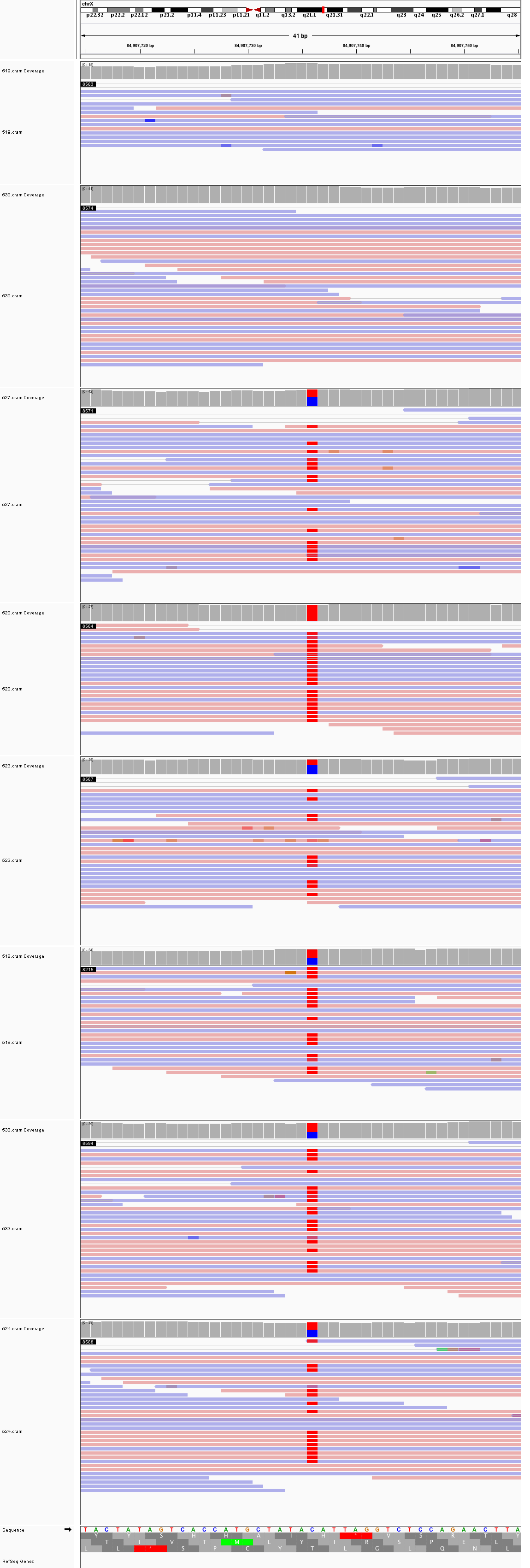

Supplement: Supplementary file 4. — All tracks below contain alignments from the third-generation children that share a DNM at the site. Reads with mapping quality <20 are filtered out, as they were not considered by our variant calling pipeline, and mismatched bases are shaded by quality score (more transparent = lower base quality). [file elife-46922-supp4.zip › supp_file_4/chrX_84,907,715_84,907,755.png]

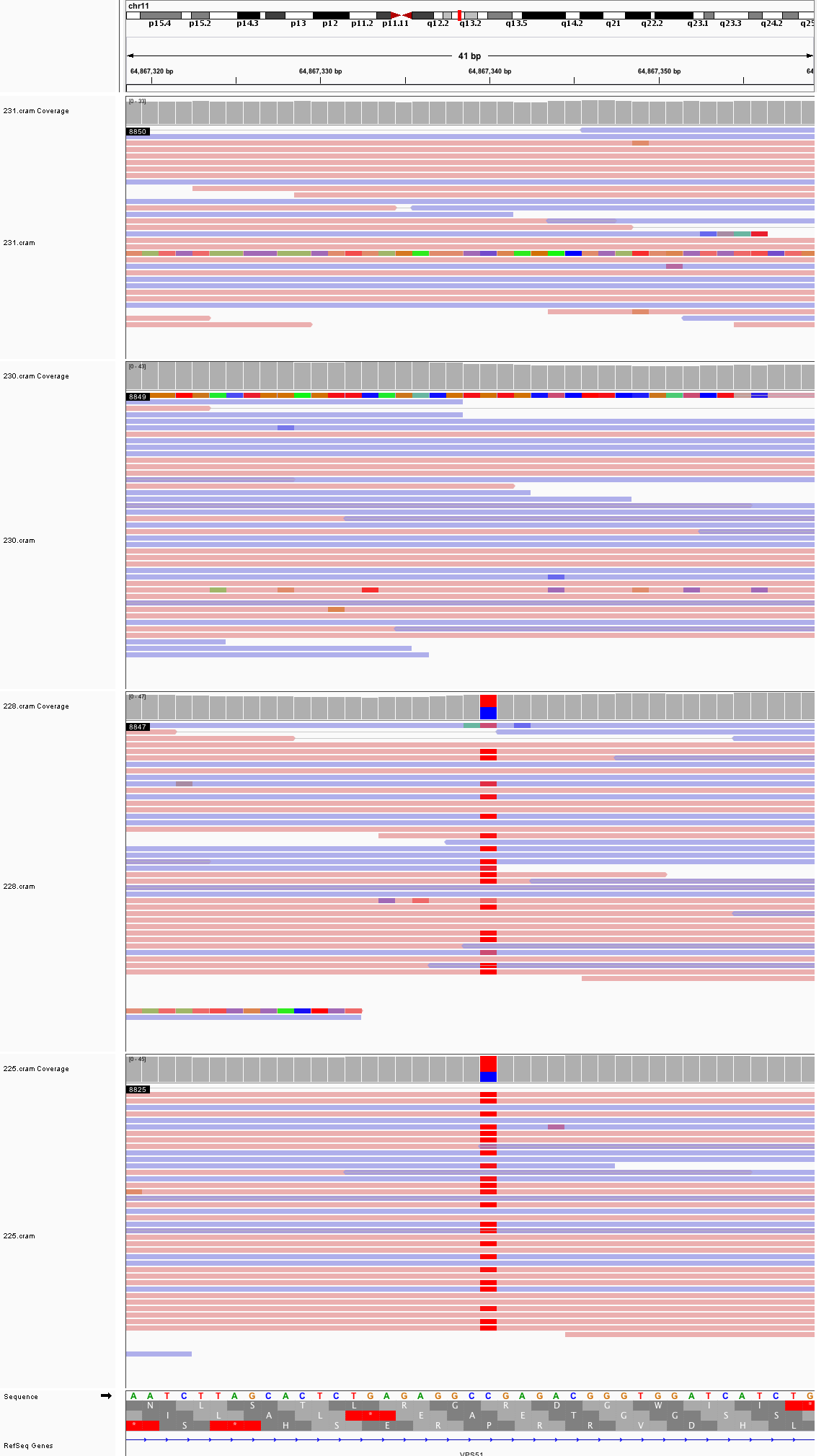

Supplement: Supplementary file 4. — All tracks below contain alignments from the third-generation children that share a DNM at the site. Reads with mapping quality <20 are filtered out, as they were not considered by our variant calling pipeline, and mismatched bases are shaded by quality score (more transparent = lower base quality). [file elife-46922-supp4.zip › supp_file_4/chr11_64,867,319_64,867,359.png]

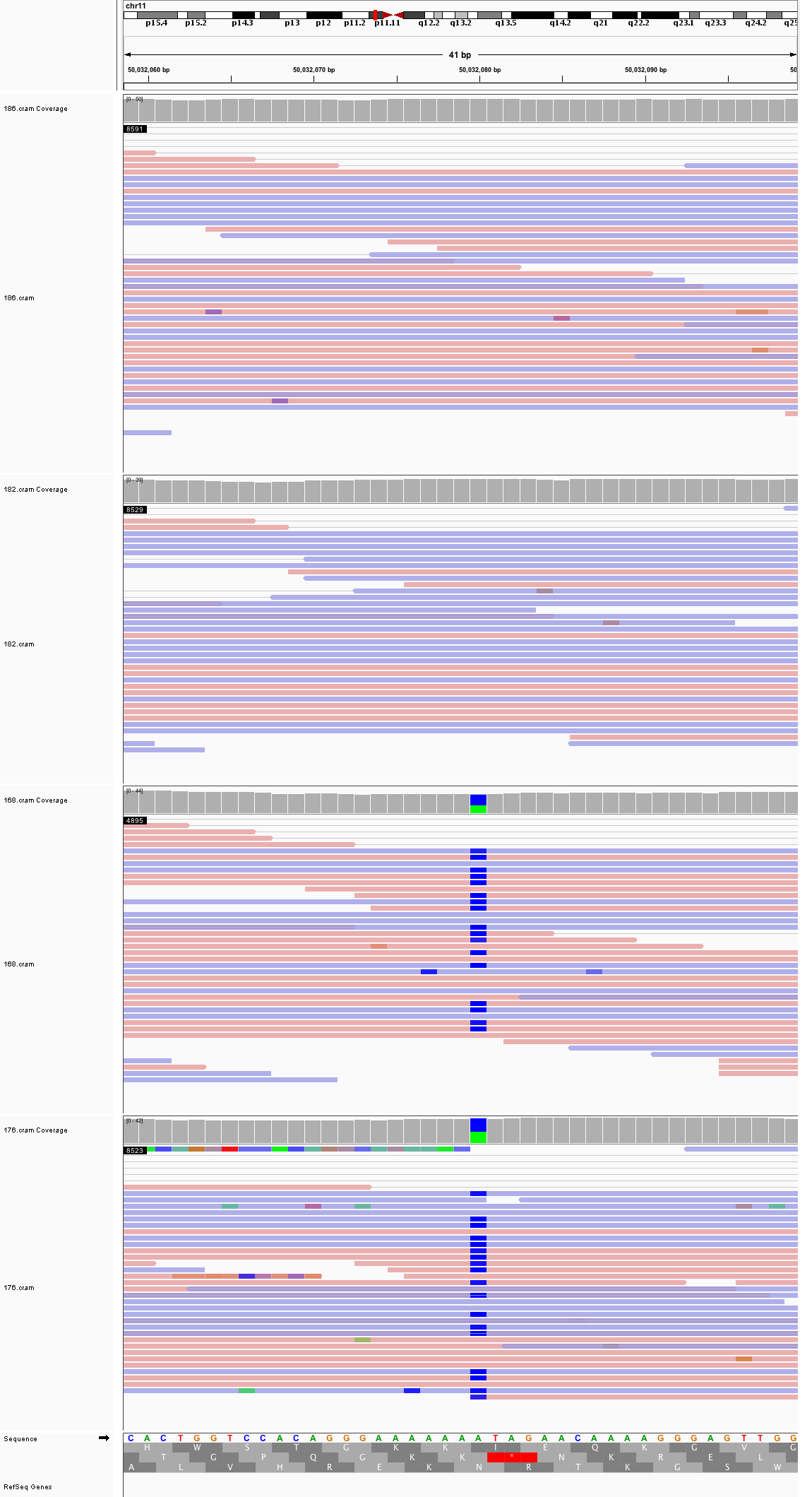

Supplement: Supplementary file 4. — All tracks below contain alignments from the third-generation children that share a DNM at the site. Reads with mapping quality <20 are filtered out, as they were not considered by our variant calling pipeline, and mismatched bases are shaded by quality score (more transparent = lower base quality). [file elife-46922-supp4.zip › supp_file_4/chr11_50,032,059_50,032,099.png]

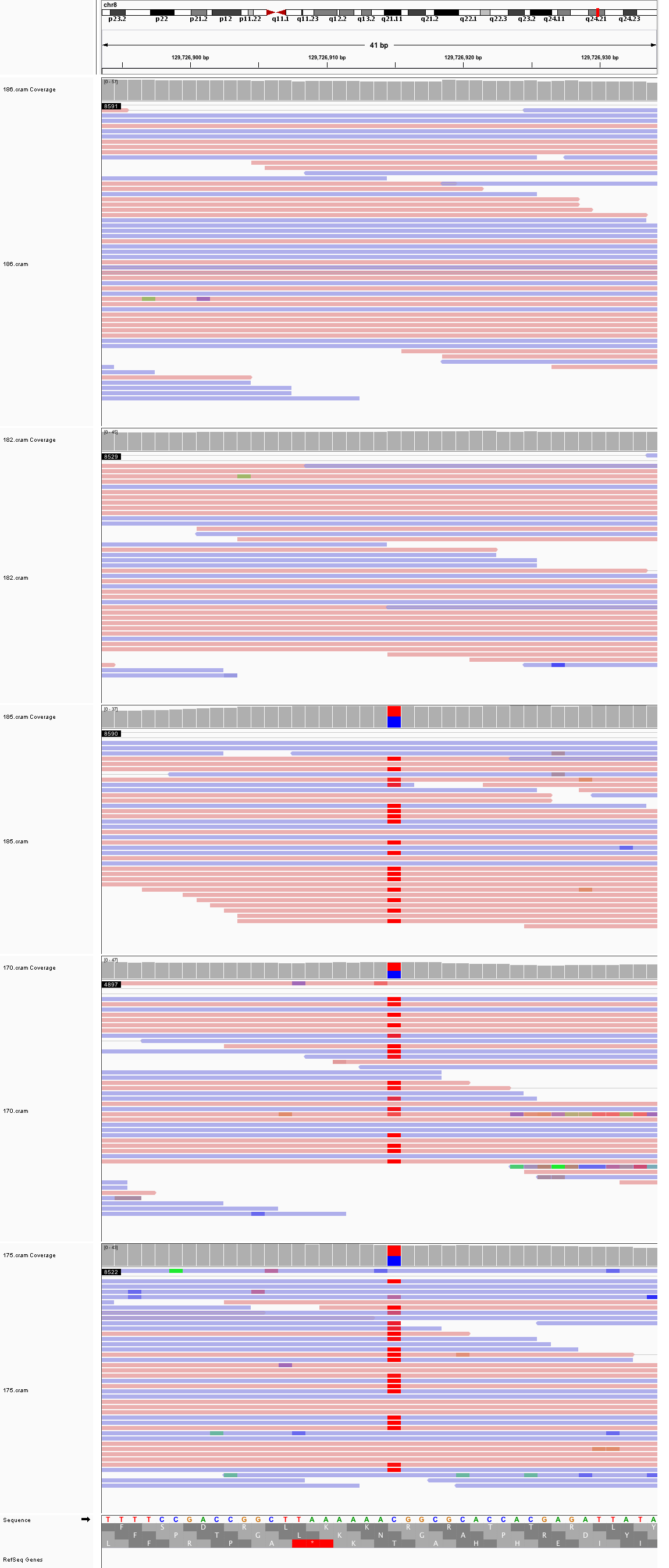

Supplement: Supplementary file 4. — All tracks below contain alignments from the third-generation children that share a DNM at the site. Reads with mapping quality <20 are filtered out, as they were not considered by our variant calling pipeline, and mismatched bases are shaded by quality score (more transparent = lower base quality). [file elife-46922-supp4.zip › supp_file_4/chr8_129,726,894_129,726,934.png]

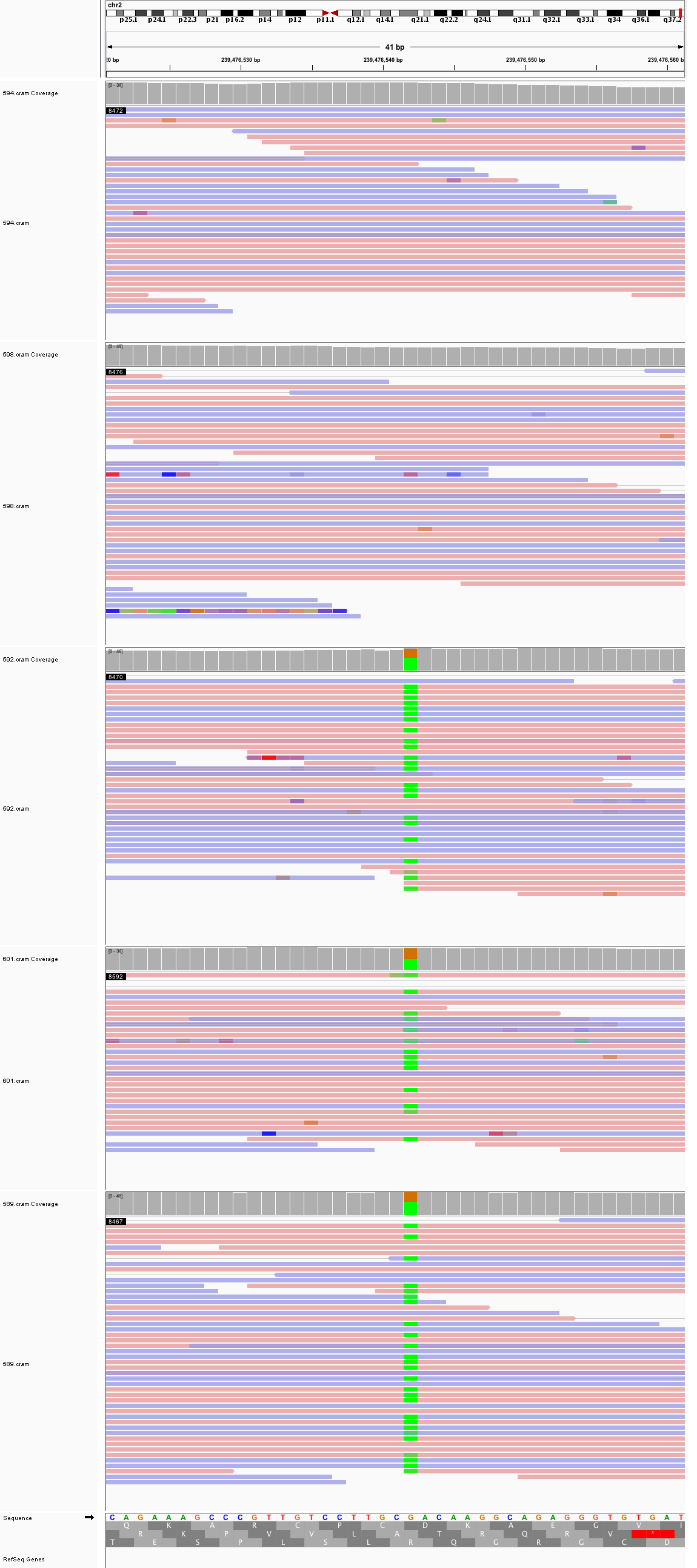

Supplement: Supplementary file 4. — All tracks below contain alignments from the third-generation children that share a DNM at the site. Reads with mapping quality <20 are filtered out, as they were not considered by our variant calling pipeline, and mismatched bases are shaded by quality score (more transparent = lower base quality). [file elife-46922-supp4.zip › supp_file_4/chr2_239,476,521_239,476,561.png]

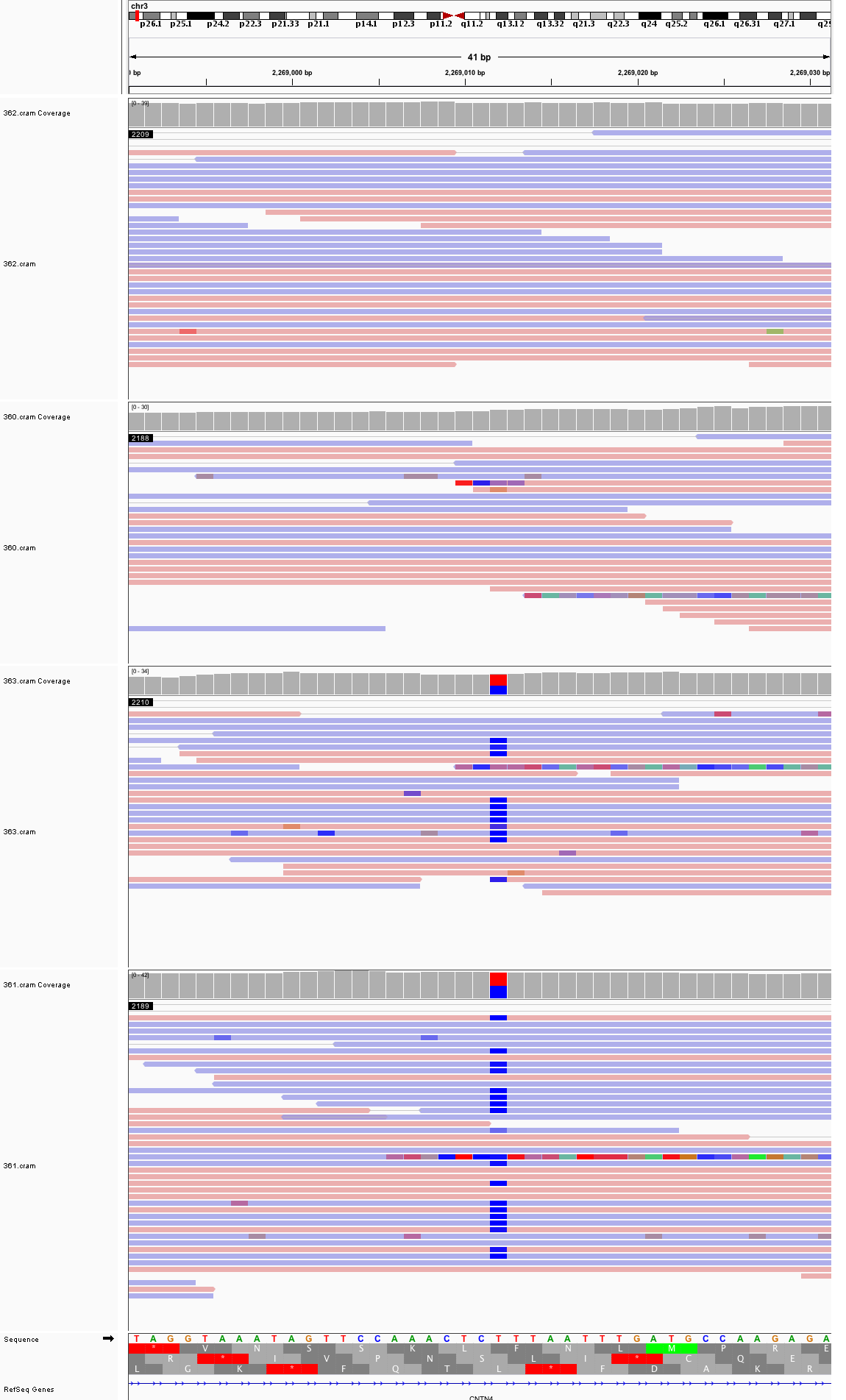

Supplement: Supplementary file 4. — All tracks below contain alignments from the third-generation children that share a DNM at the site. Reads with mapping quality <20 are filtered out, as they were not considered by our variant calling pipeline, and mismatched bases are shaded by quality score (more transparent = lower base quality). [file elife-46922-supp4.zip › supp_file_4/chr3_2,268,991_2,269,031.png]

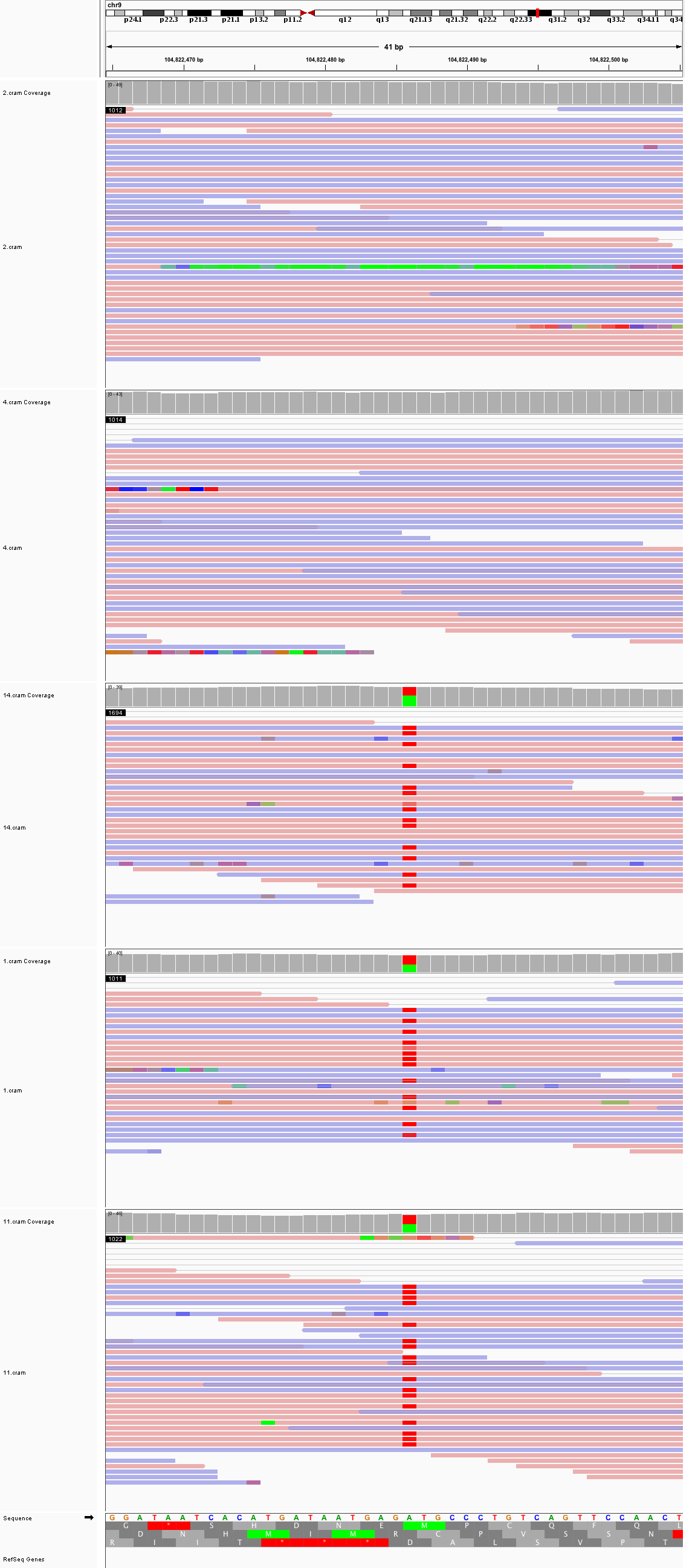

Supplement: Supplementary file 4. — All tracks below contain alignments from the third-generation children that share a DNM at the site. Reads with mapping quality <20 are filtered out, as they were not considered by our variant calling pipeline, and mismatched bases are shaded by quality score (more transparent = lower base quality). [file elife-46922-supp4.zip › supp_file_4/chr9_104,822,465_104,822,505.png]

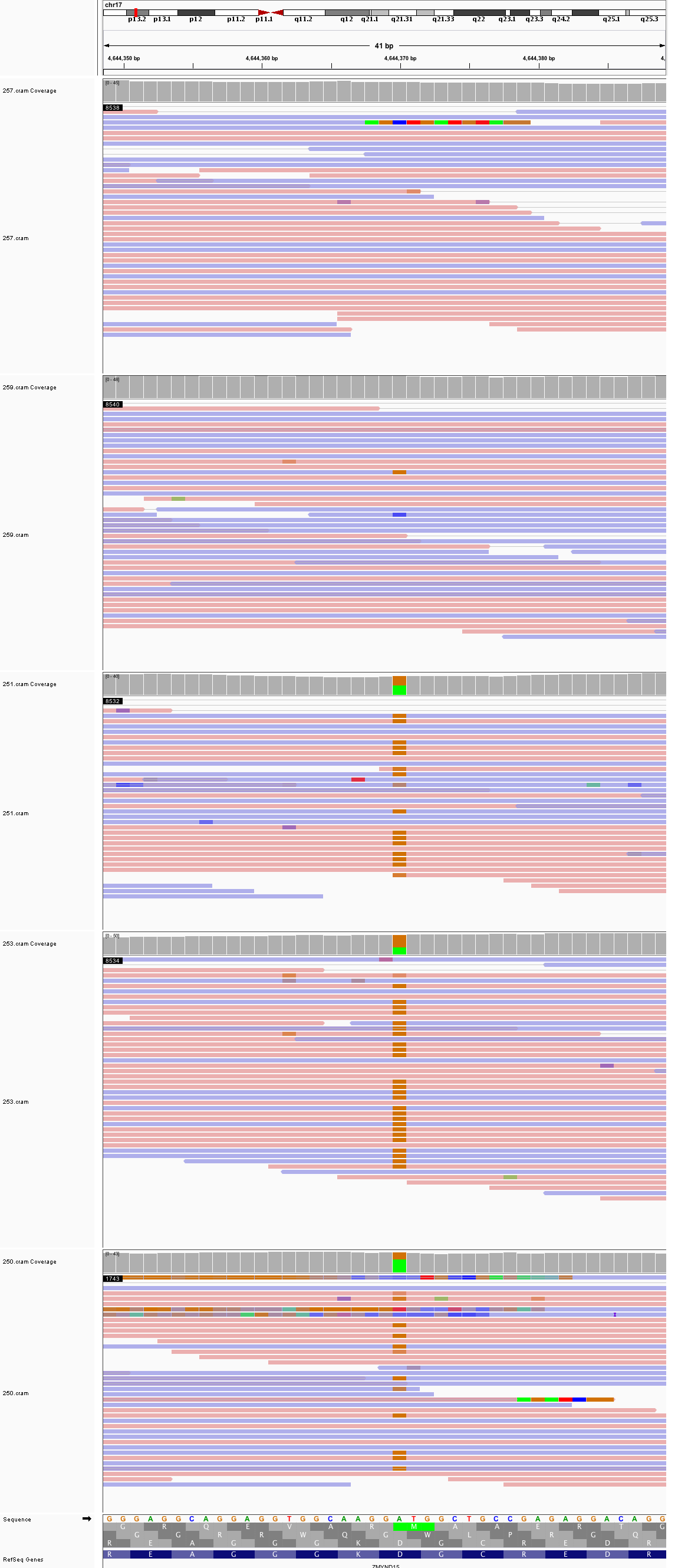

Supplement: Supplementary file 4. — All tracks below contain alignments from the third-generation children that share a DNM at the site. Reads with mapping quality <20 are filtered out, as they were not considered by our variant calling pipeline, and mismatched bases are shaded by quality score (more transparent = lower base quality). [file elife-46922-supp4.zip › supp_file_4/chr17_4,644,349_4,644,389.png]

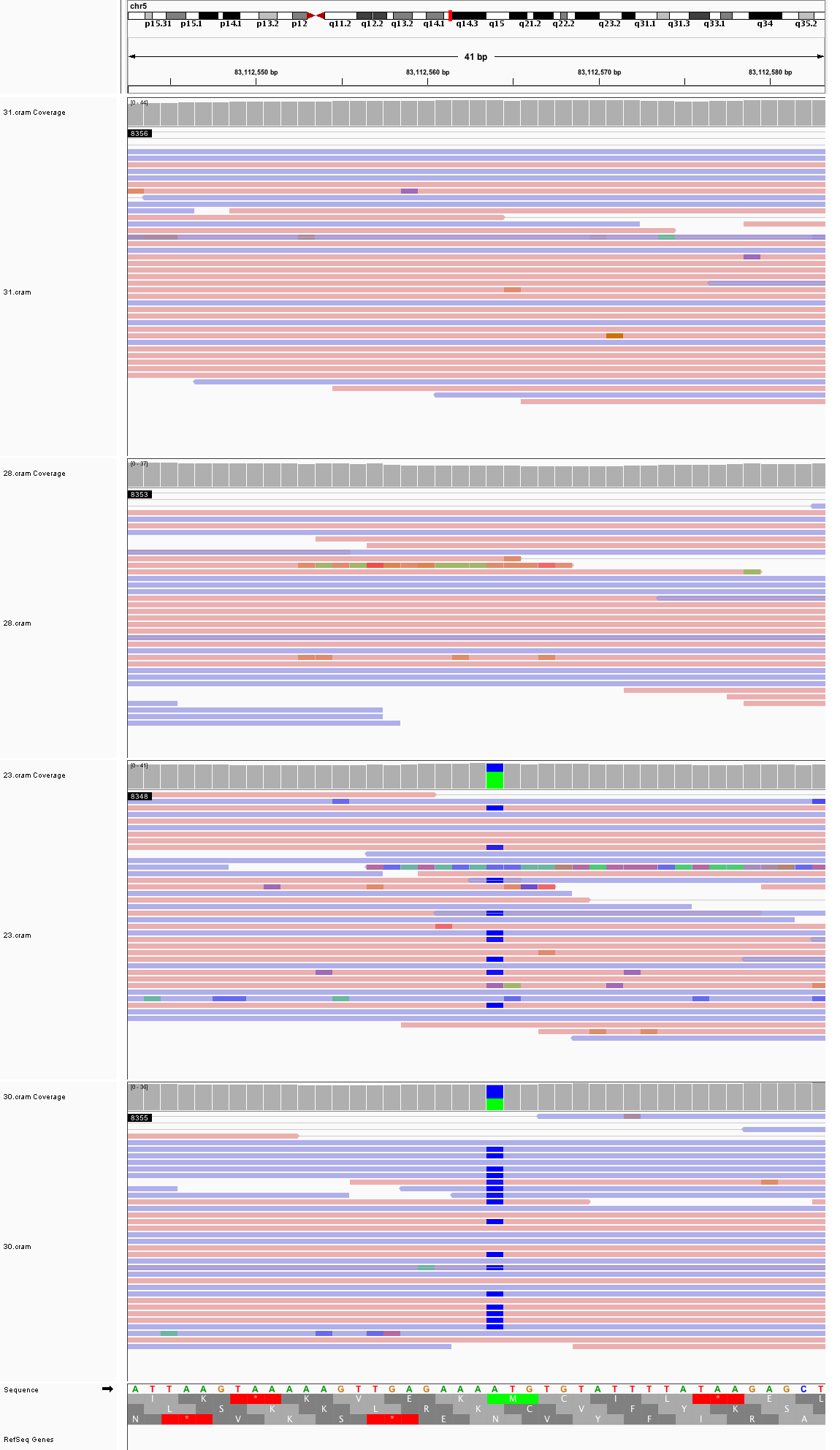

Supplement: Supplementary file 4. — All tracks below contain alignments from the third-generation children that share a DNM at the site. Reads with mapping quality <20 are filtered out, as they were not considered by our variant calling pipeline, and mismatched bases are shaded by quality score (more transparent = lower base quality). [file elife-46922-supp4.zip › supp_file_4/chr5_83,112,543_83,112,583.png]

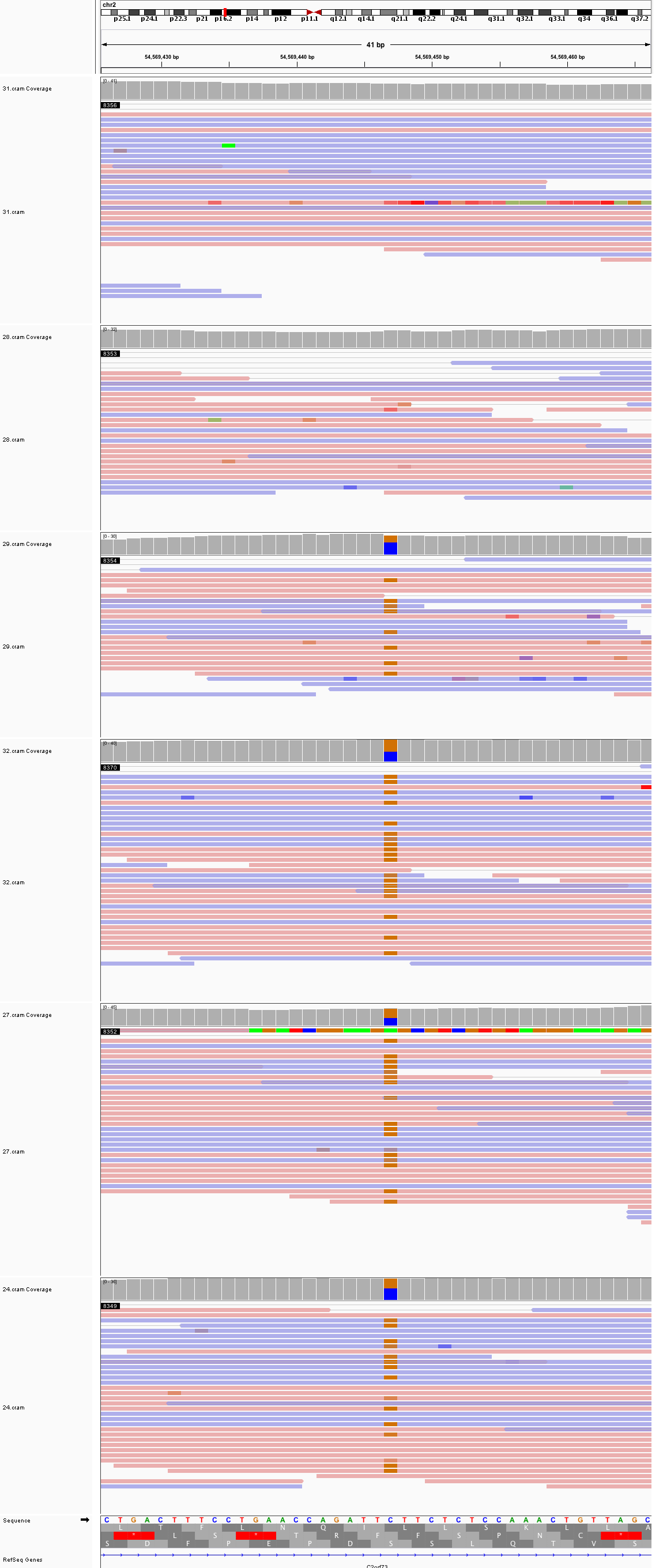

Supplement: Supplementary file 4. — All tracks below contain alignments from the third-generation children that share a DNM at the site. Reads with mapping quality <20 are filtered out, as they were not considered by our variant calling pipeline, and mismatched bases are shaded by quality score (more transparent = lower base quality). [file elife-46922-supp4.zip › supp_file_4/chr2_54,569,426_54,569,466.png]

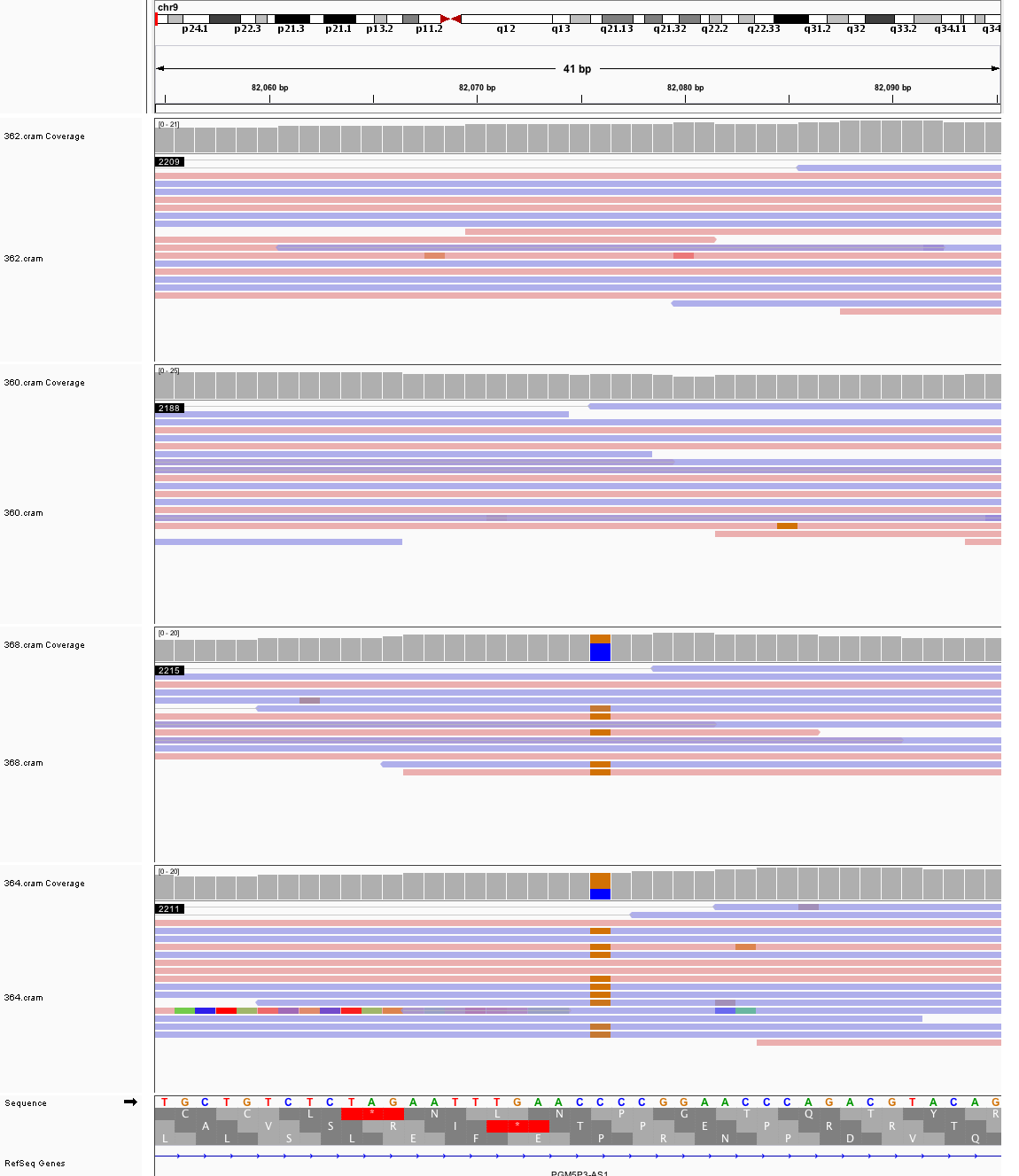

Supplement: Supplementary file 4. — All tracks below contain alignments from the third-generation children that share a DNM at the site. Reads with mapping quality <20 are filtered out, as they were not considered by our variant calling pipeline, and mismatched bases are shaded by quality score (more transparent = lower base quality). [file elife-46922-supp4.zip › supp_file_4/chr9_82,055_82,095.png]

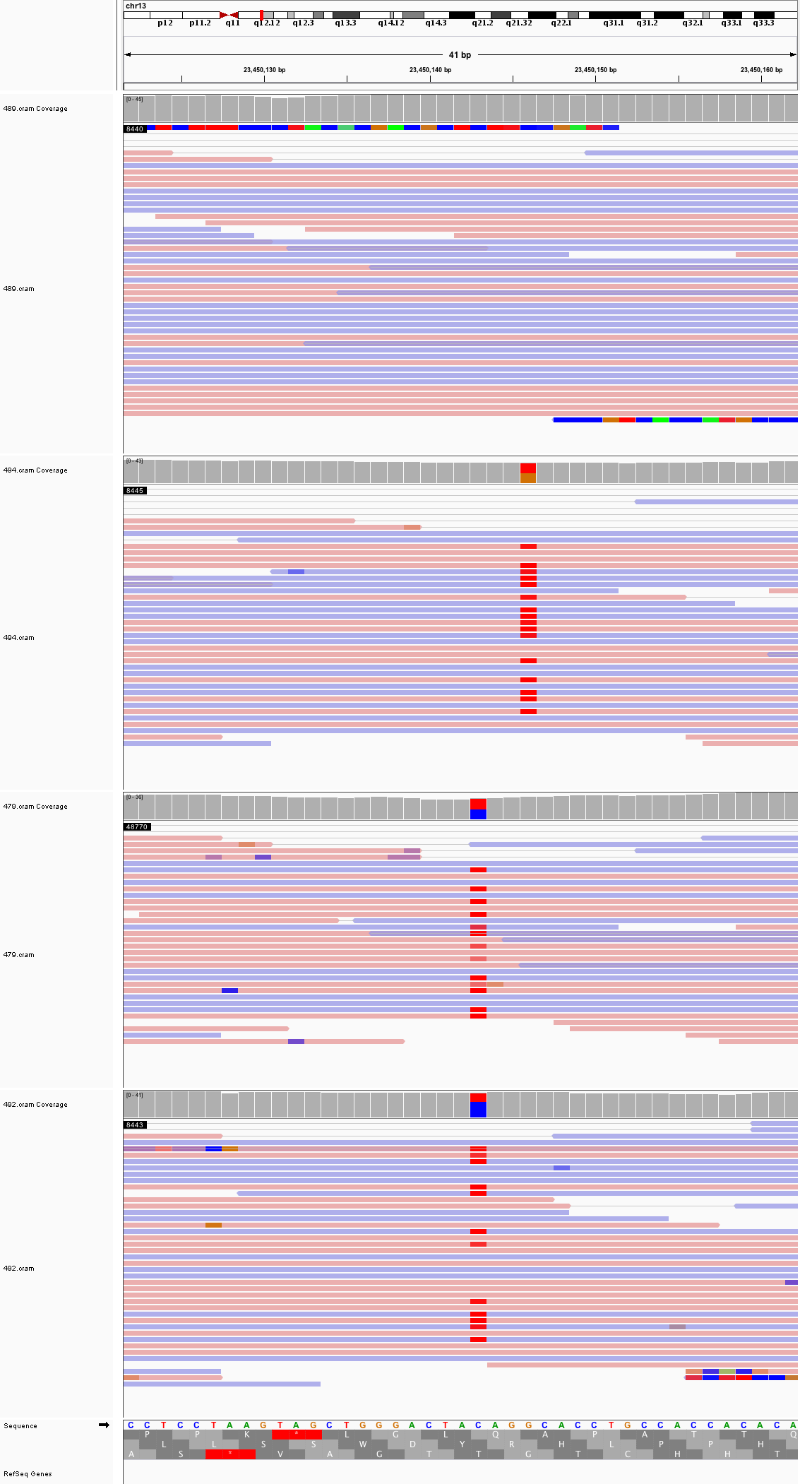

Supplement: Supplementary file 4. — All tracks below contain alignments from the third-generation children that share a DNM at the site. Reads with mapping quality <20 are filtered out, as they were not considered by our variant calling pipeline, and mismatched bases are shaded by quality score (more transparent = lower base quality). [file elife-46922-supp4.zip › supp_file_4/chr13_23,450,122_23,450,162.png]

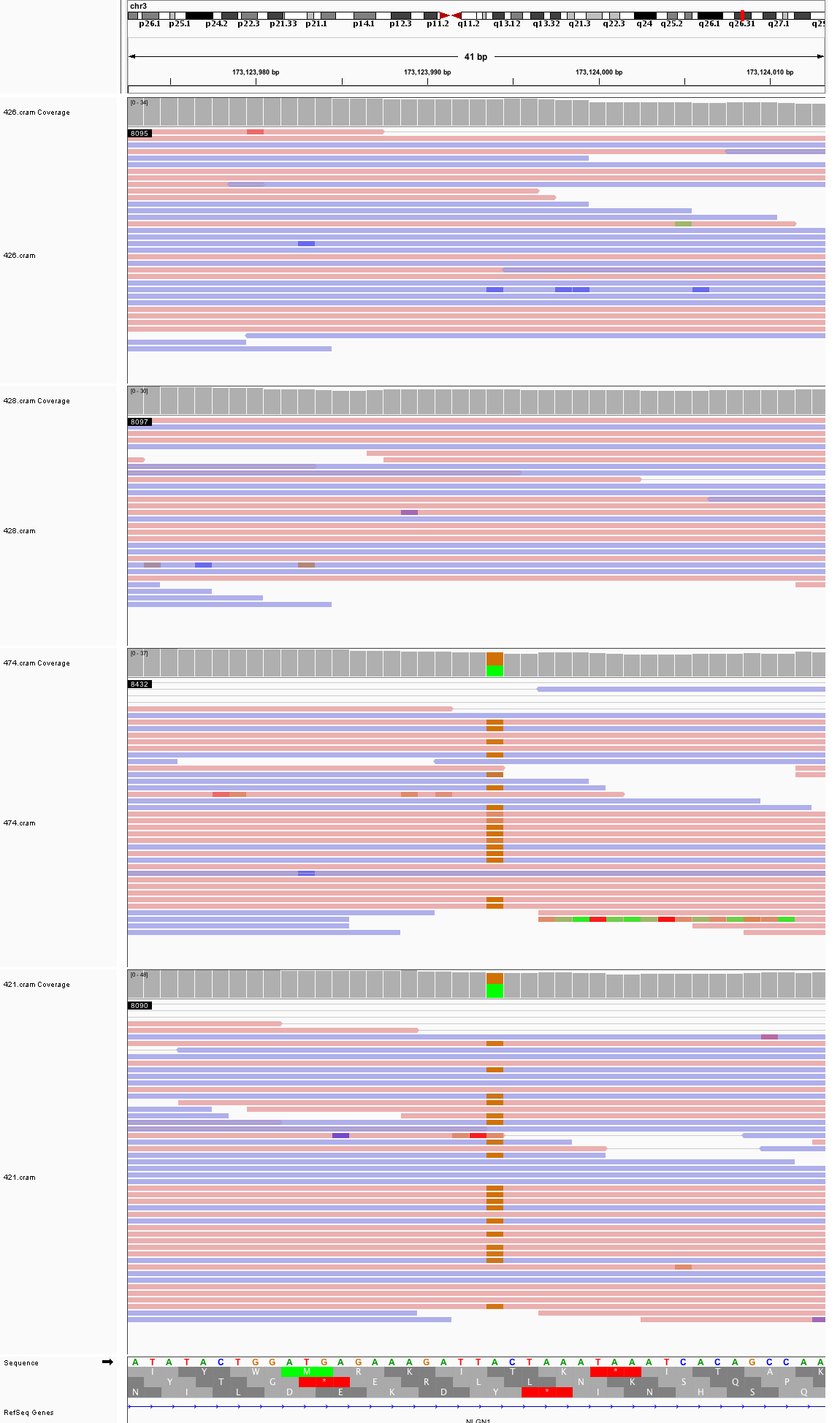

Supplement: Supplementary file 4. — All tracks below contain alignments from the third-generation children that share a DNM at the site. Reads with mapping quality <20 are filtered out, as they were not considered by our variant calling pipeline, and mismatched bases are shaded by quality score (more transparent = lower base quality). [file elife-46922-supp4.zip › supp_file_4/chr3_173,123,973_173,124,013.png]

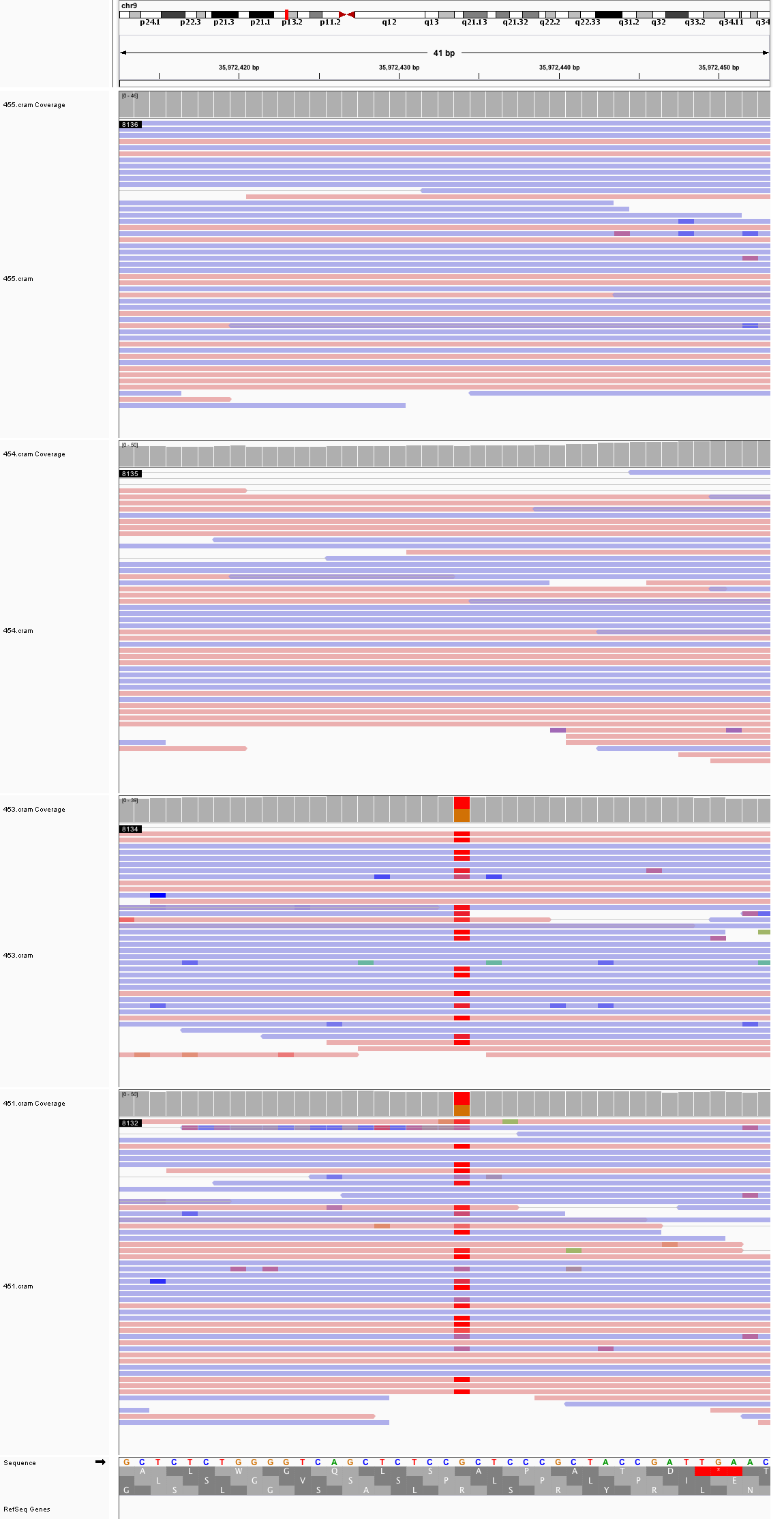

Supplement: Supplementary file 4. — All tracks below contain alignments from the third-generation children that share a DNM at the site. Reads with mapping quality <20 are filtered out, as they were not considered by our variant calling pipeline, and mismatched bases are shaded by quality score (more transparent = lower base quality). [file elife-46922-supp4.zip › supp_file_4/chr9_35,972,413_35,972,453.png]

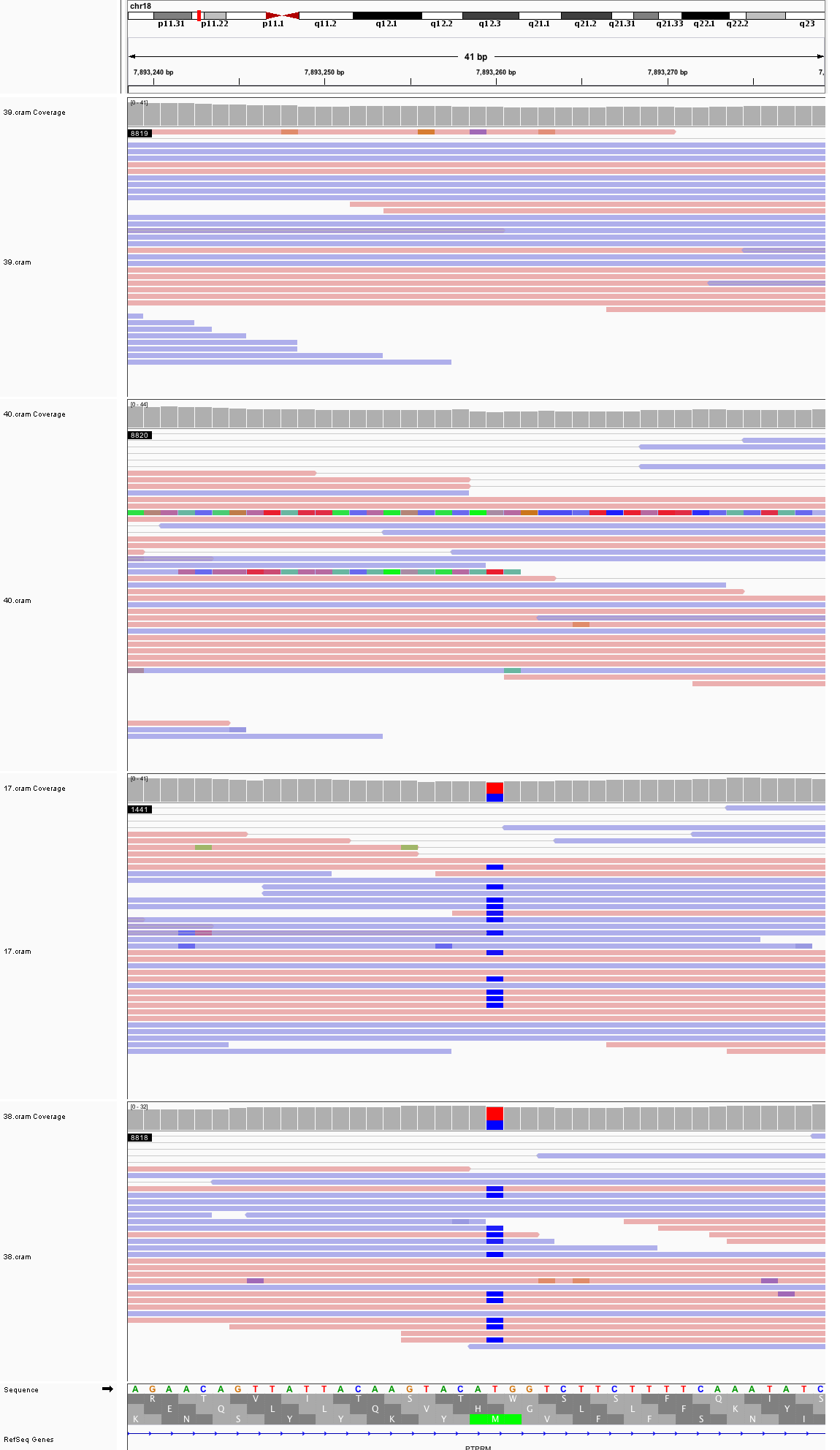

Supplement: Supplementary file 4. — All tracks below contain alignments from the third-generation children that share a DNM at the site. Reads with mapping quality <20 are filtered out, as they were not considered by our variant calling pipeline, and mismatched bases are shaded by quality score (more transparent = lower base quality). [file elife-46922-supp4.zip › supp_file_4/chr18_7,893,239_7,893,279.png]

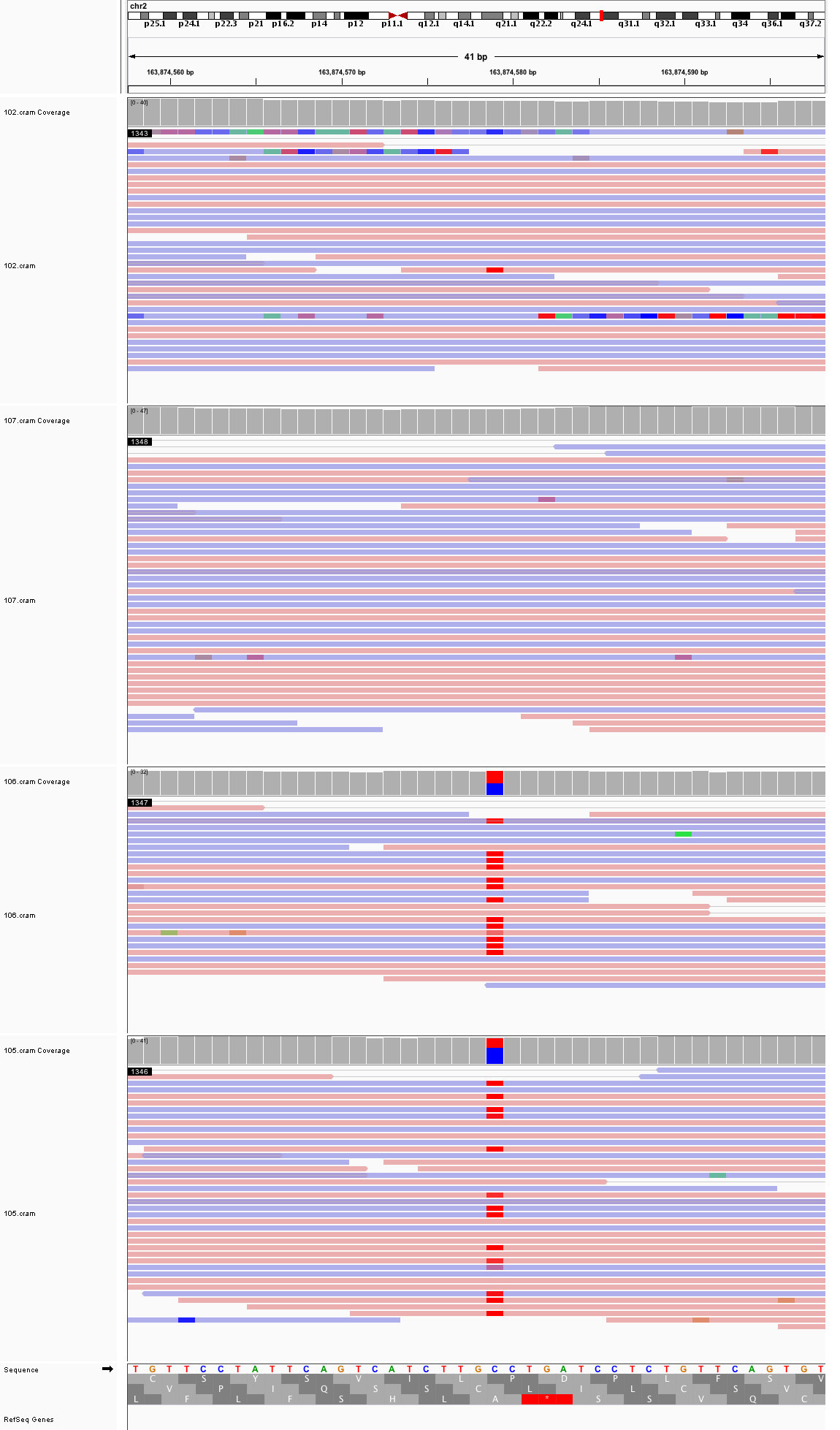

Supplement: Supplementary file 4. — All tracks below contain alignments from the third-generation children that share a DNM at the site. Reads with mapping quality <20 are filtered out, as they were not considered by our variant calling pipeline, and mismatched bases are shaded by quality score (more transparent = lower base quality). [file elife-46922-supp4.zip › supp_file_4/chr2_163,874,558_163,874,598.png]

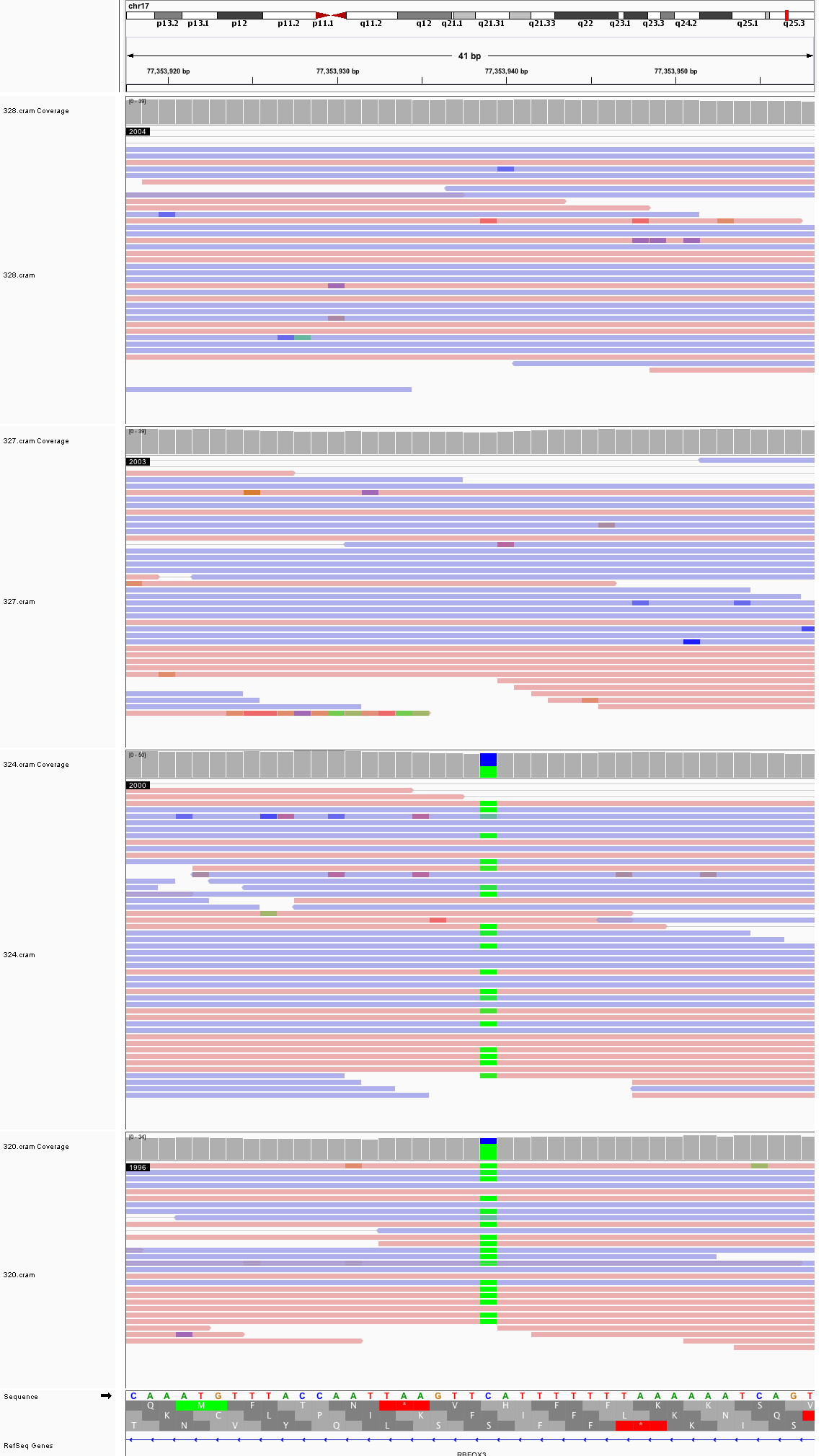

Supplement: Supplementary file 4. — All tracks below contain alignments from the third-generation children that share a DNM at the site. Reads with mapping quality <20 are filtered out, as they were not considered by our variant calling pipeline, and mismatched bases are shaded by quality score (more transparent = lower base quality). [file elife-46922-supp4.zip › supp_file_4/chr17_77,353,918_77,353,958.png]

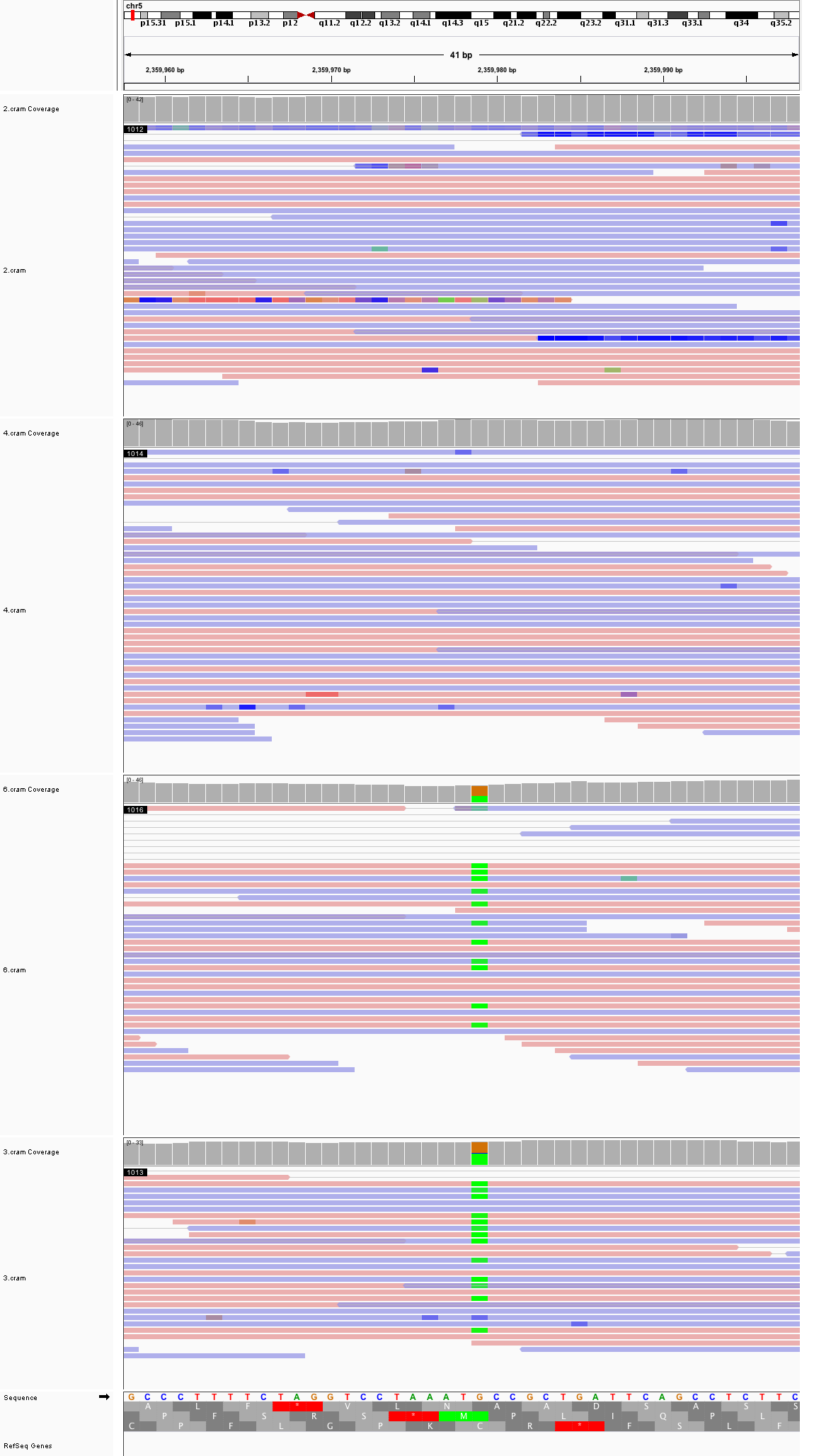

Supplement: Supplementary file 4. — All tracks below contain alignments from the third-generation children that share a DNM at the site. Reads with mapping quality <20 are filtered out, as they were not considered by our variant calling pipeline, and mismatched bases are shaded by quality score (more transparent = lower base quality). [file elife-46922-supp4.zip › supp_file_4/chr5_2,359,958_2,359,998.png]

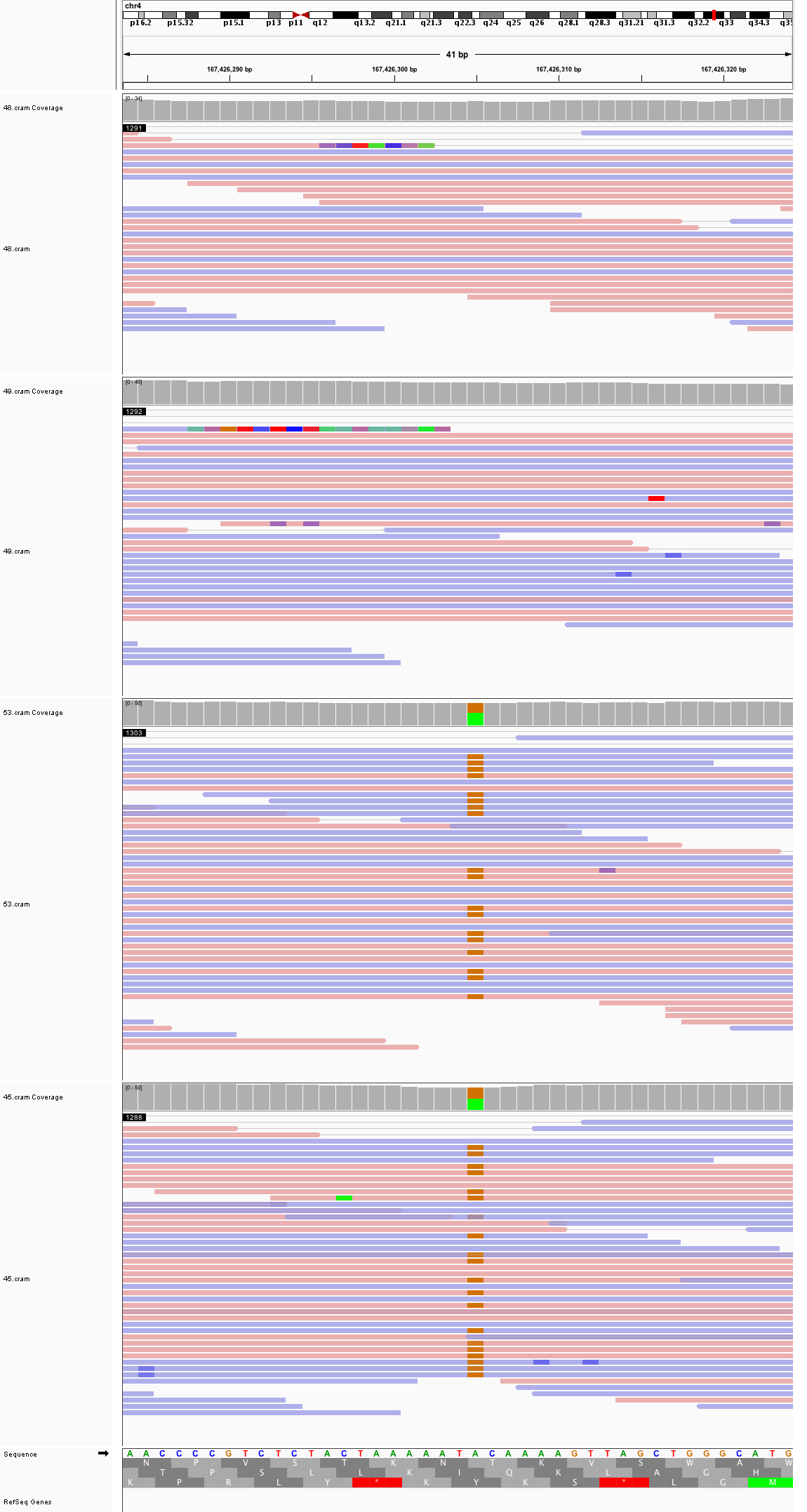

Supplement: Supplementary file 4. — All tracks below contain alignments from the third-generation children that share a DNM at the site. Reads with mapping quality <20 are filtered out, as they were not considered by our variant calling pipeline, and mismatched bases are shaded by quality score (more transparent = lower base quality). [file elife-46922-supp4.zip › supp_file_4/chr4_167,426,284_167,426,324.png]

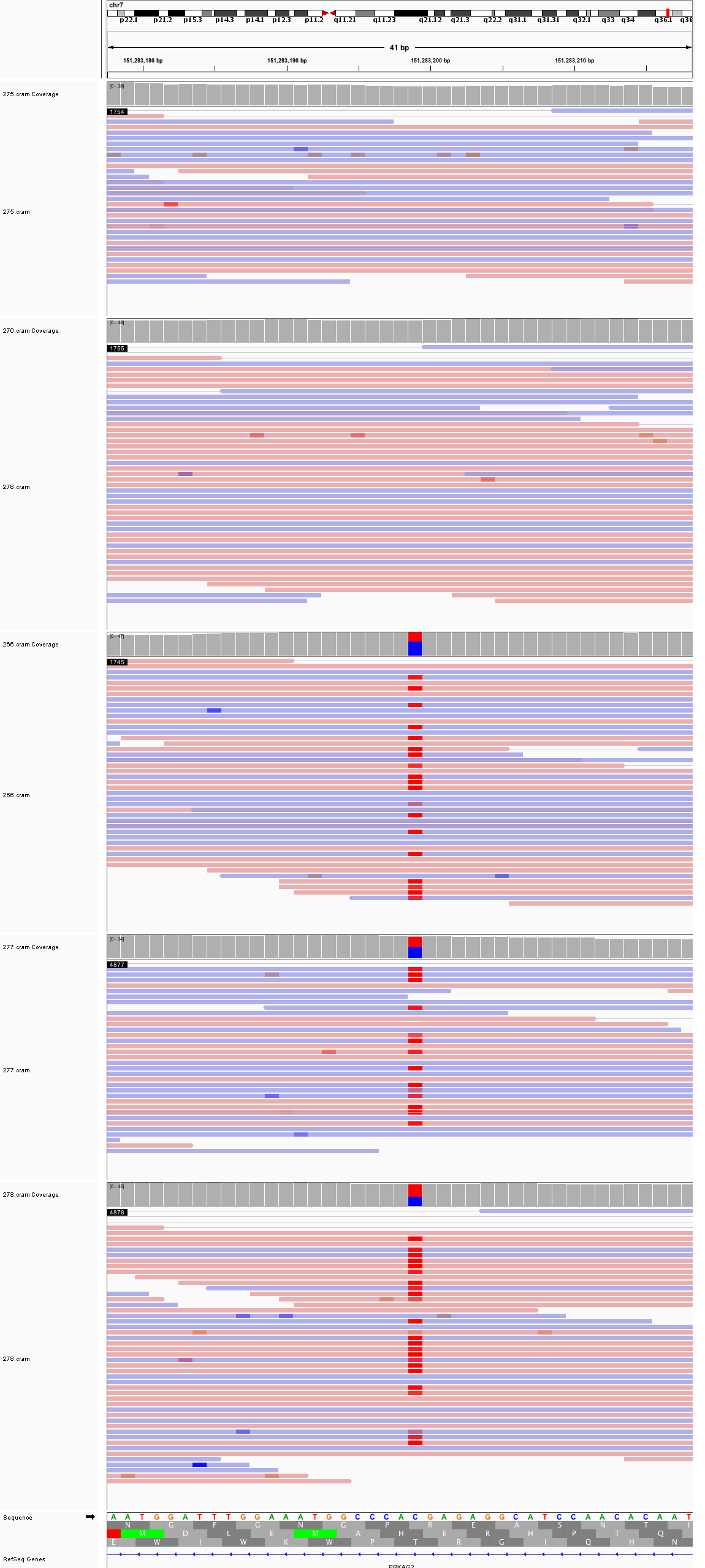

Supplement: Supplementary file 4. — All tracks below contain alignments from the third-generation children that share a DNM at the site. Reads with mapping quality <20 are filtered out, as they were not considered by our variant calling pipeline, and mismatched bases are shaded by quality score (more transparent = lower base quality). [file elife-46922-supp4.zip › supp_file_4/chr7_151,283,178_151,283,218.png]

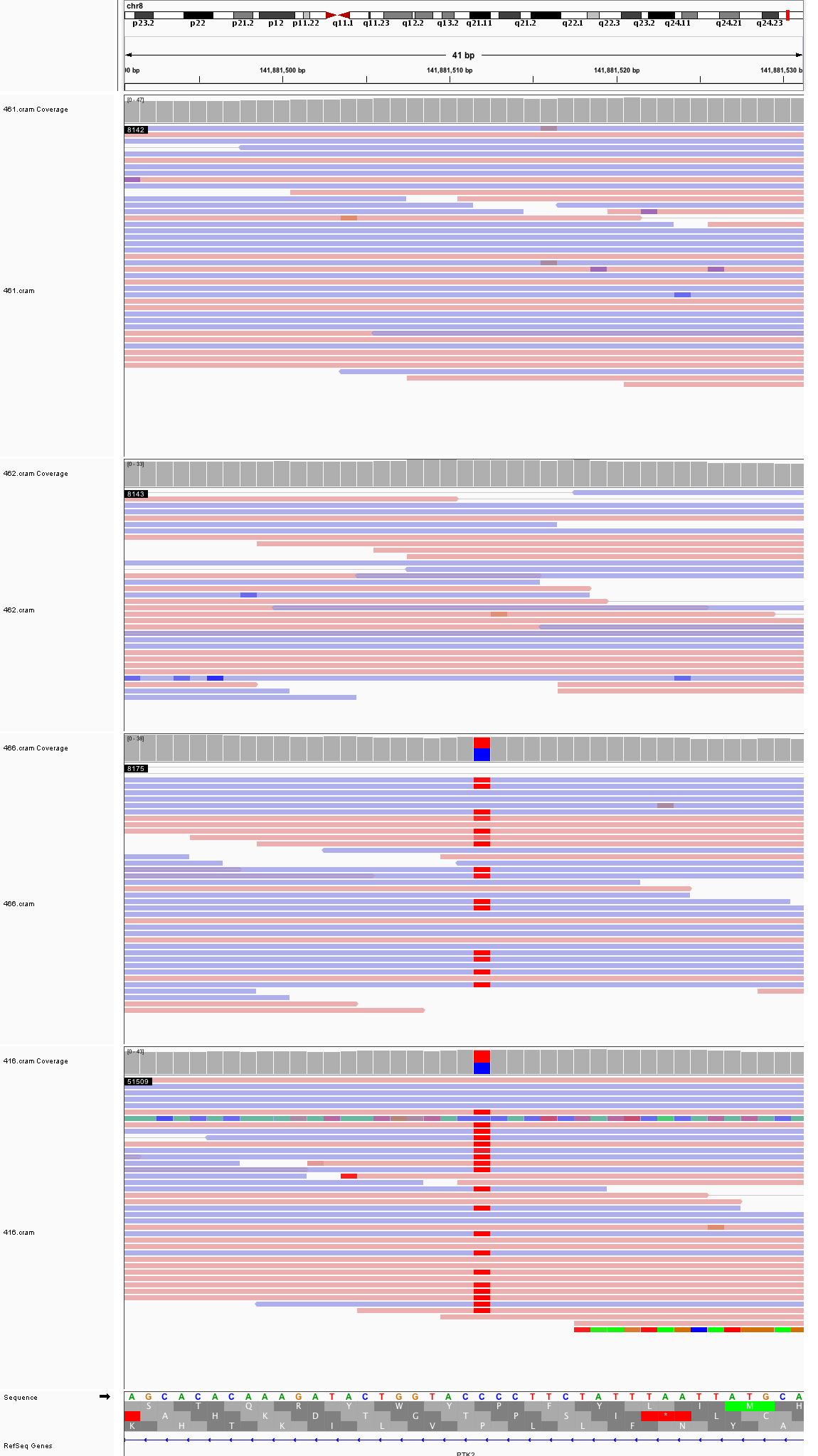

Supplement: Supplementary file 4. — All tracks below contain alignments from the third-generation children that share a DNM at the site. Reads with mapping quality <20 are filtered out, as they were not considered by our variant calling pipeline, and mismatched bases are shaded by quality score (more transparent = lower base quality). [file elife-46922-supp4.zip › supp_file_4/chr8_141,881,491_141,881,531.png]

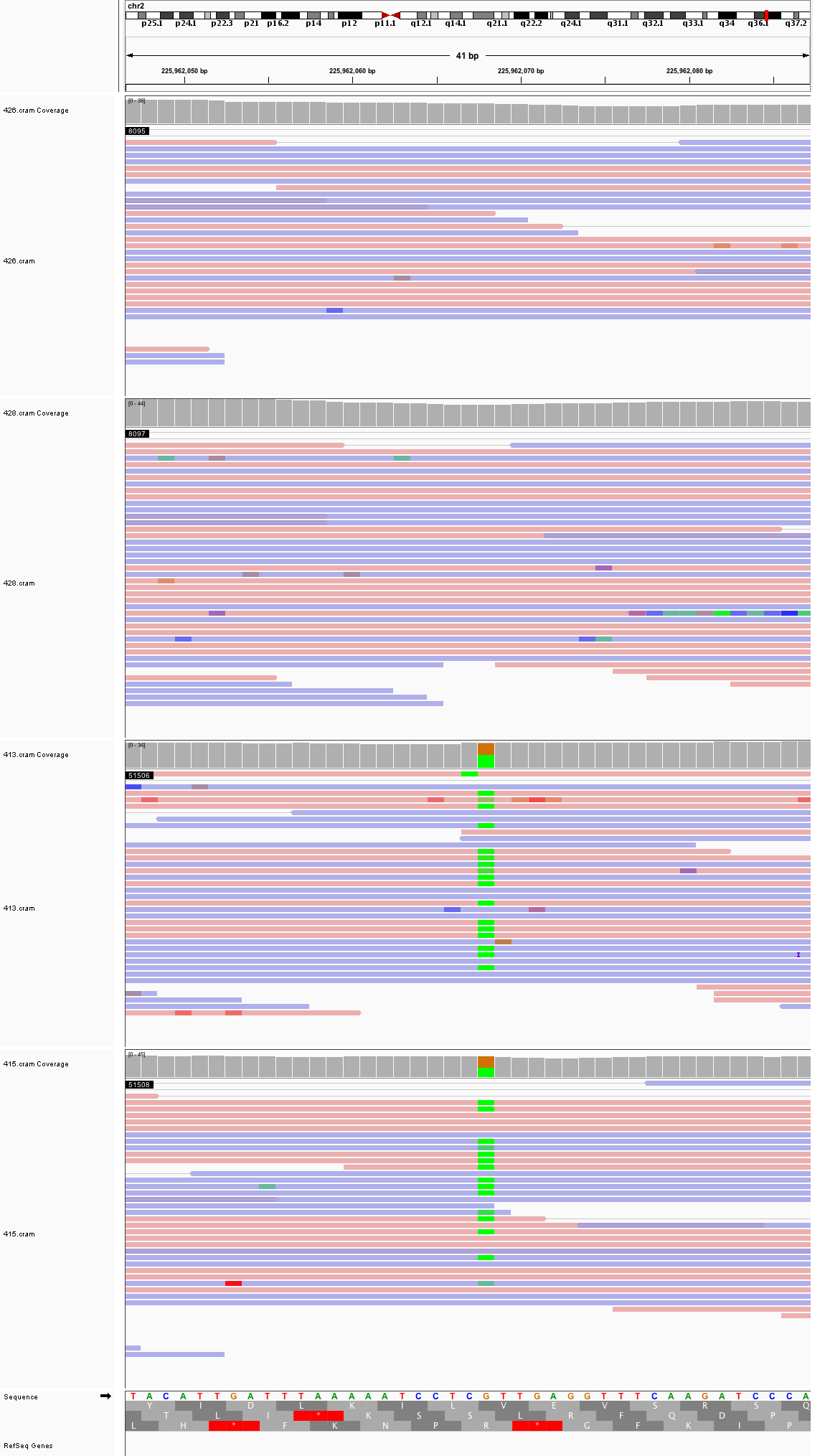

Supplement: Supplementary file 4. — All tracks below contain alignments from the third-generation children that share a DNM at the site. Reads with mapping quality <20 are filtered out, as they were not considered by our variant calling pipeline, and mismatched bases are shaded by quality score (more transparent = lower base quality). [file elife-46922-supp4.zip › supp_file_4/chr2_225,962,047_225,962,087.png]

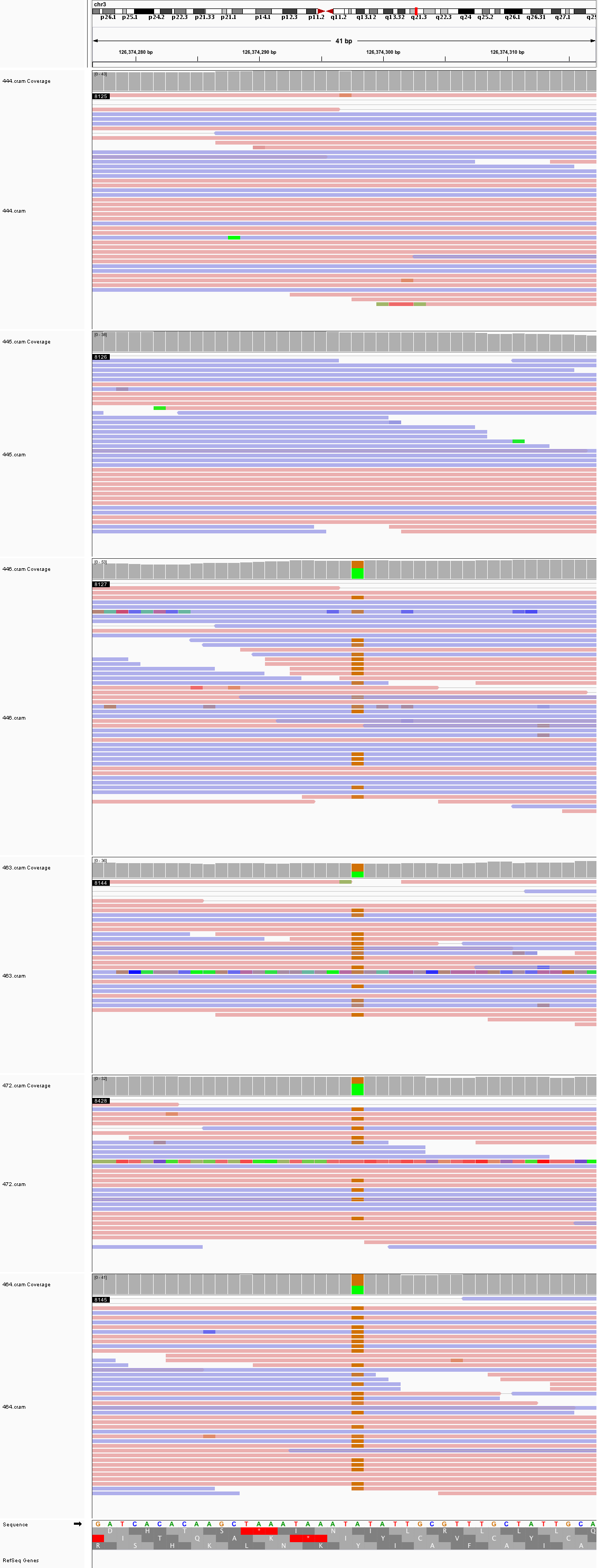

Supplement: Supplementary file 4. — All tracks below contain alignments from the third-generation children that share a DNM at the site. Reads with mapping quality <20 are filtered out, as they were not considered by our variant calling pipeline, and mismatched bases are shaded by quality score (more transparent = lower base quality). [file elife-46922-supp4.zip › supp_file_4/chr3_126,374,277_126,374,317.png]

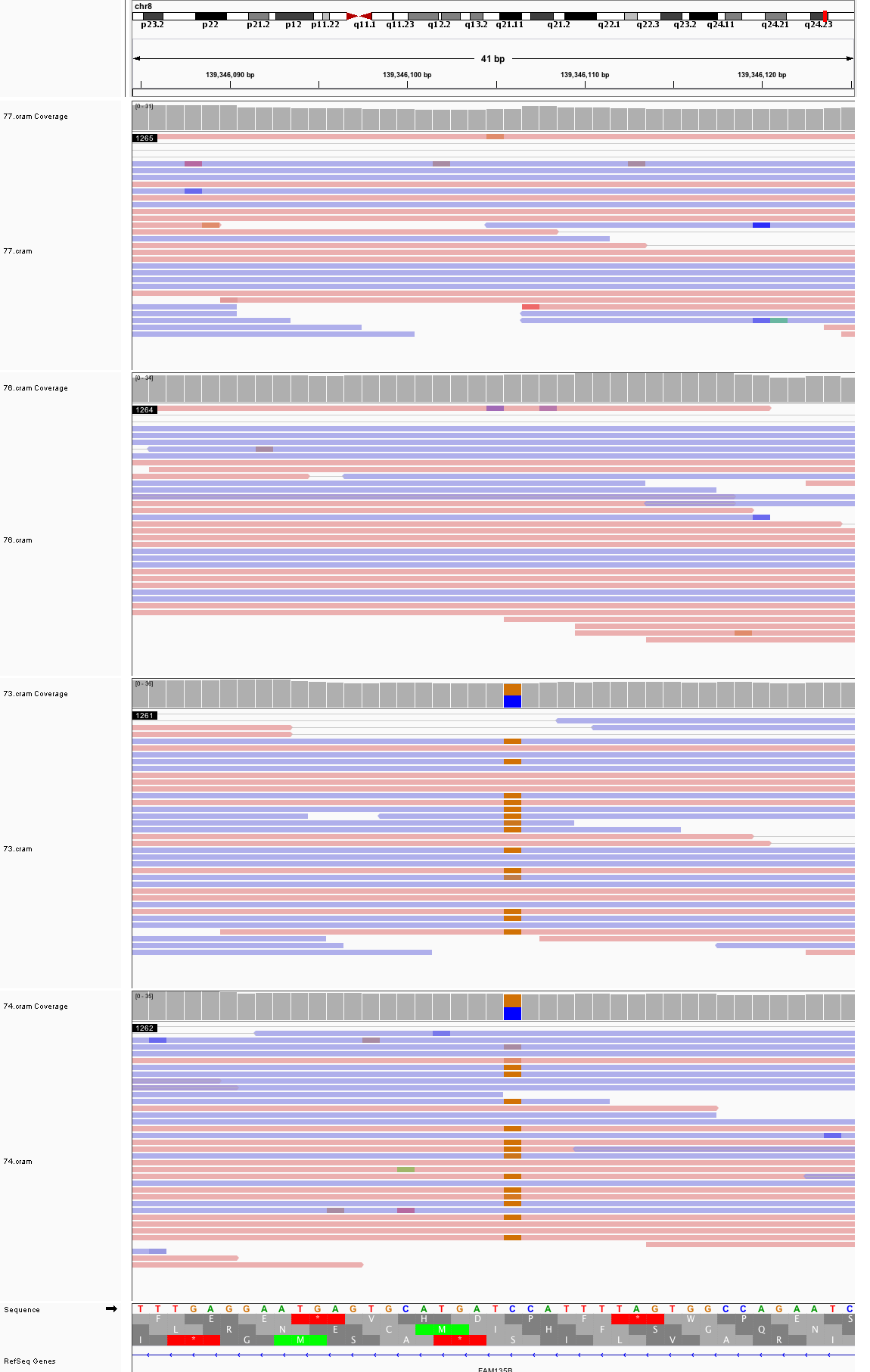

Supplement: Supplementary file 4. — All tracks below contain alignments from the third-generation children that share a DNM at the site. Reads with mapping quality <20 are filtered out, as they were not considered by our variant calling pipeline, and mismatched bases are shaded by quality score (more transparent = lower base quality). [file elife-46922-supp4.zip › supp_file_4/chr8_139,346,085_139,346,125.png]

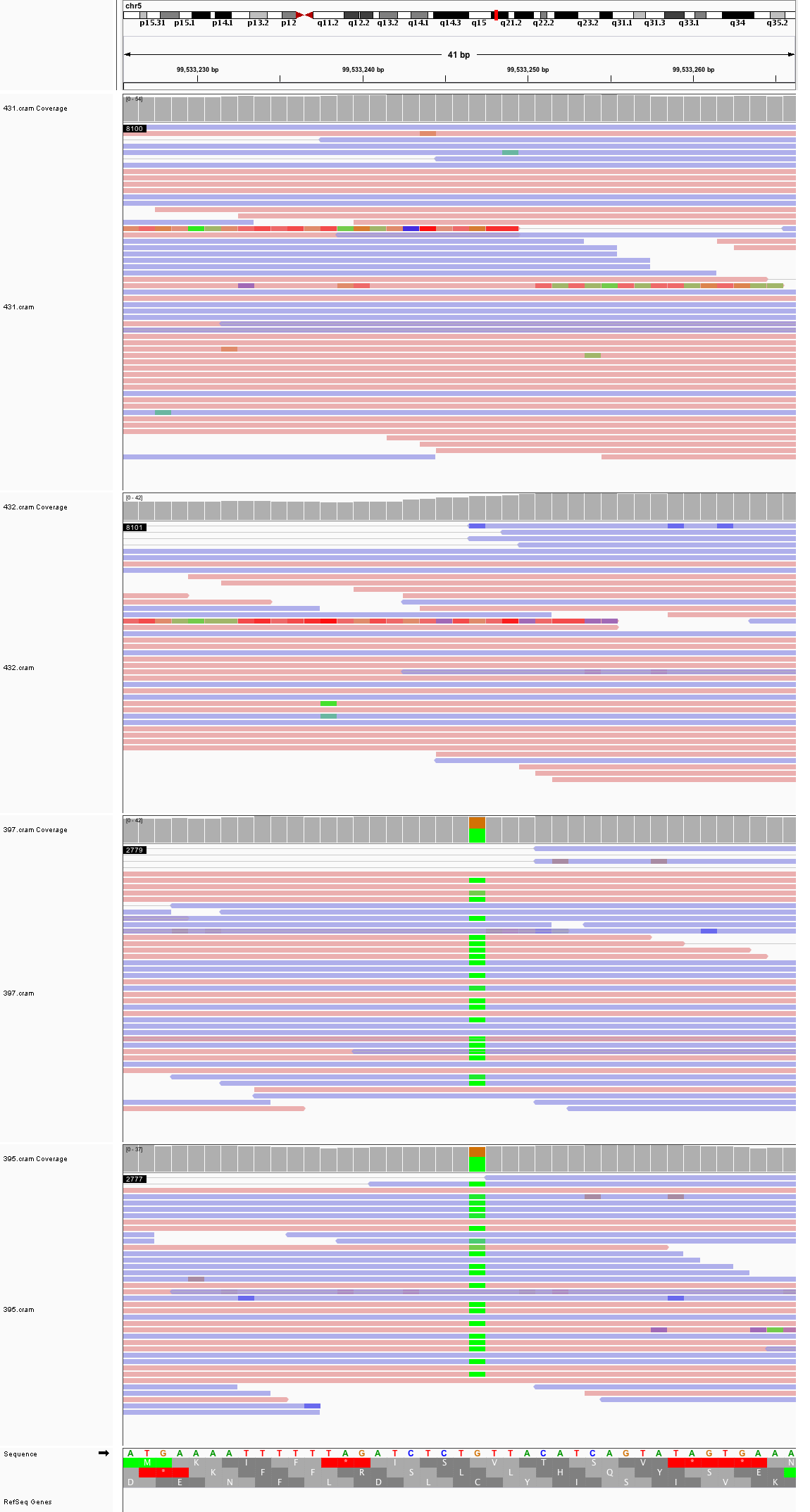

Supplement: Supplementary file 4. — All tracks below contain alignments from the third-generation children that share a DNM at the site. Reads with mapping quality <20 are filtered out, as they were not considered by our variant calling pipeline, and mismatched bases are shaded by quality score (more transparent = lower base quality). [file elife-46922-supp4.zip › supp_file_4/chr5_99,533,226_99,533,266.png]

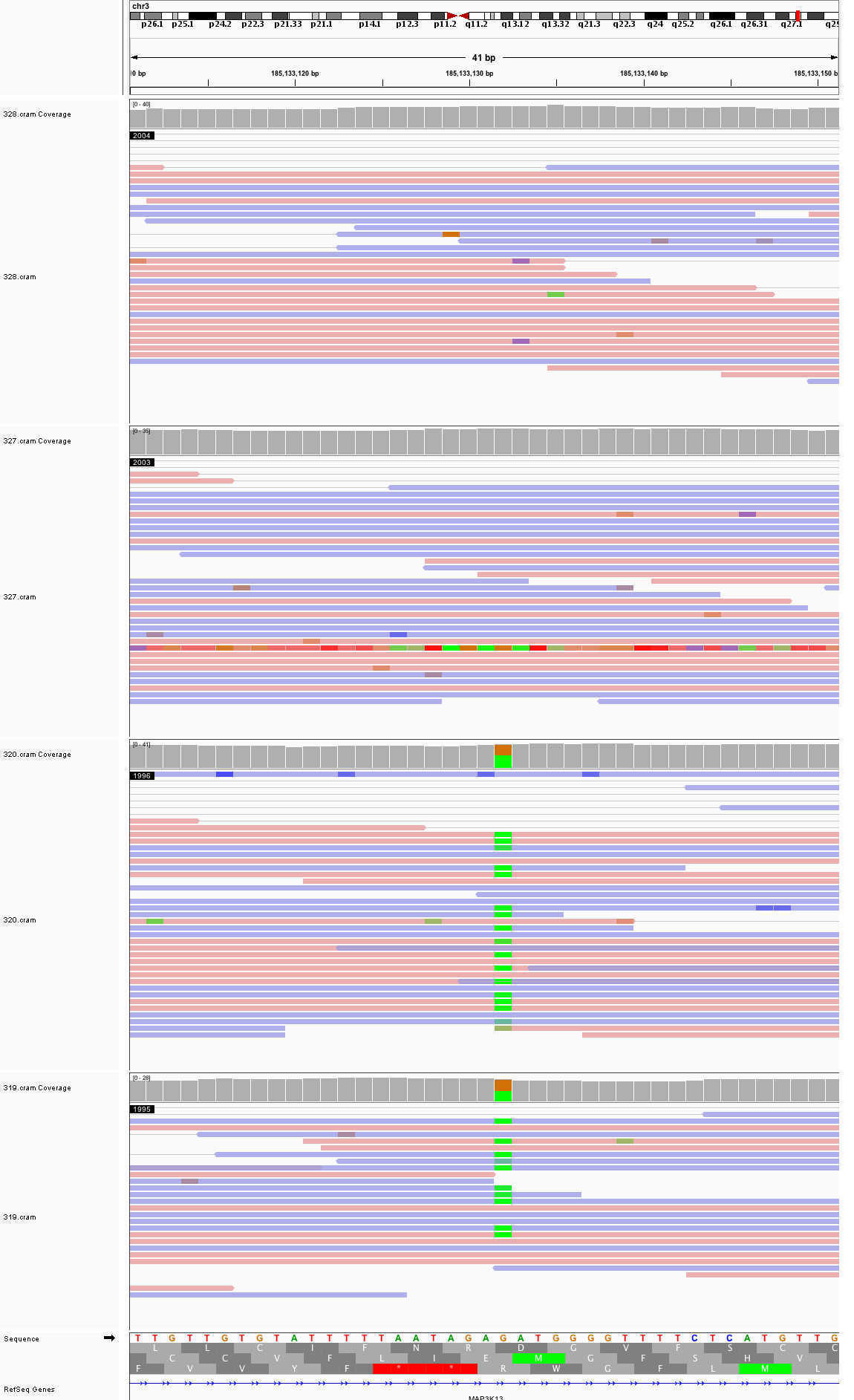

Supplement: Supplementary file 4. — All tracks below contain alignments from the third-generation children that share a DNM at the site. Reads with mapping quality <20 are filtered out, as they were not considered by our variant calling pipeline, and mismatched bases are shaded by quality score (more transparent = lower base quality). [file elife-46922-supp4.zip › supp_file_4/chr3_185,133,111_185,133,151.png]

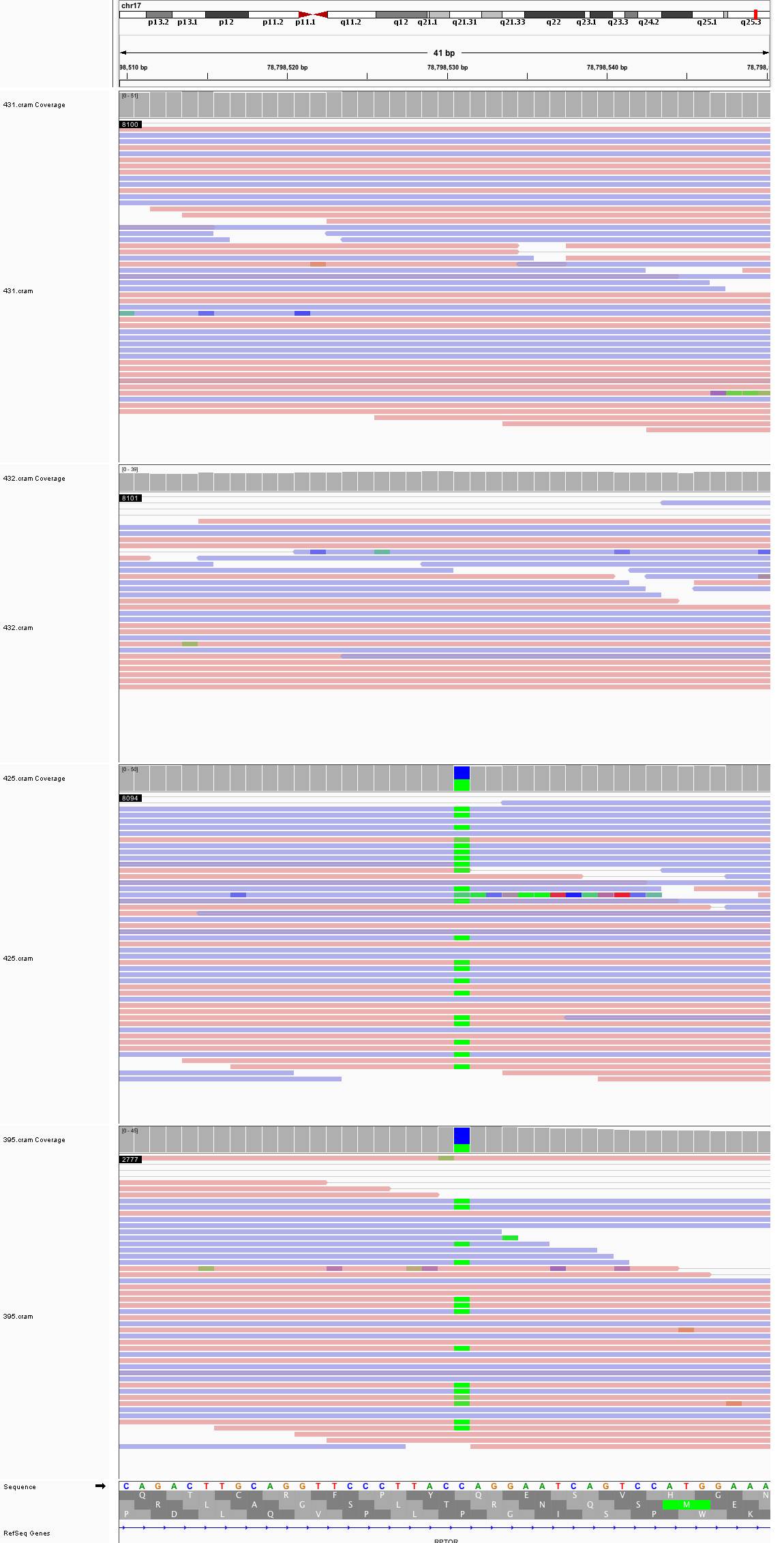

Supplement: Supplementary file 4. — All tracks below contain alignments from the third-generation children that share a DNM at the site. Reads with mapping quality <20 are filtered out, as they were not considered by our variant calling pipeline, and mismatched bases are shaded by quality score (more transparent = lower base quality). [file elife-46922-supp4.zip › supp_file_4/chr17_78,798,510_78,798,550.png]

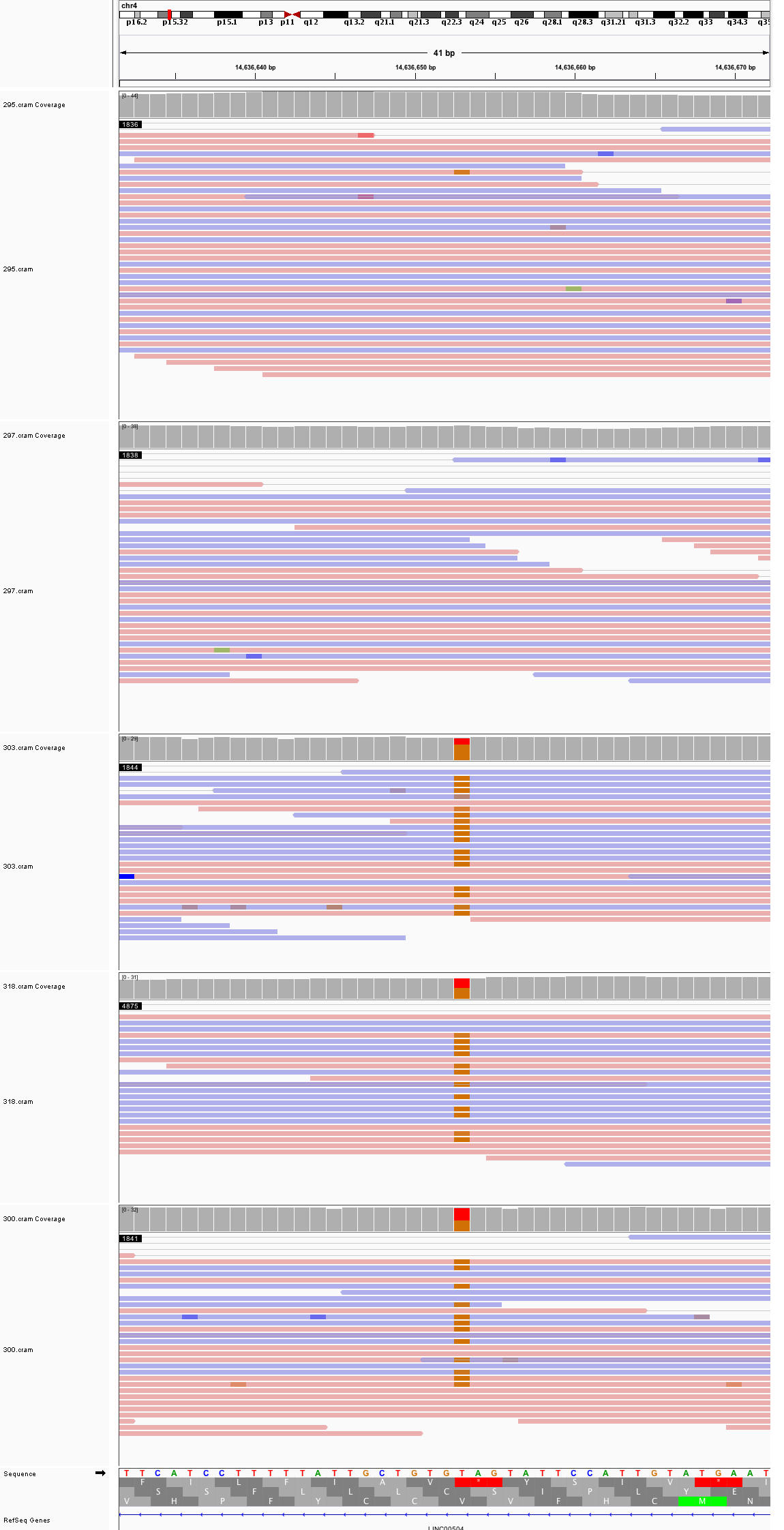

Supplement: Supplementary file 4. — All tracks below contain alignments from the third-generation children that share a DNM at the site. Reads with mapping quality <20 are filtered out, as they were not considered by our variant calling pipeline, and mismatched bases are shaded by quality score (more transparent = lower base quality). [file elife-46922-supp4.zip › supp_file_4/chr4_14,636,632_14,636,672.png]

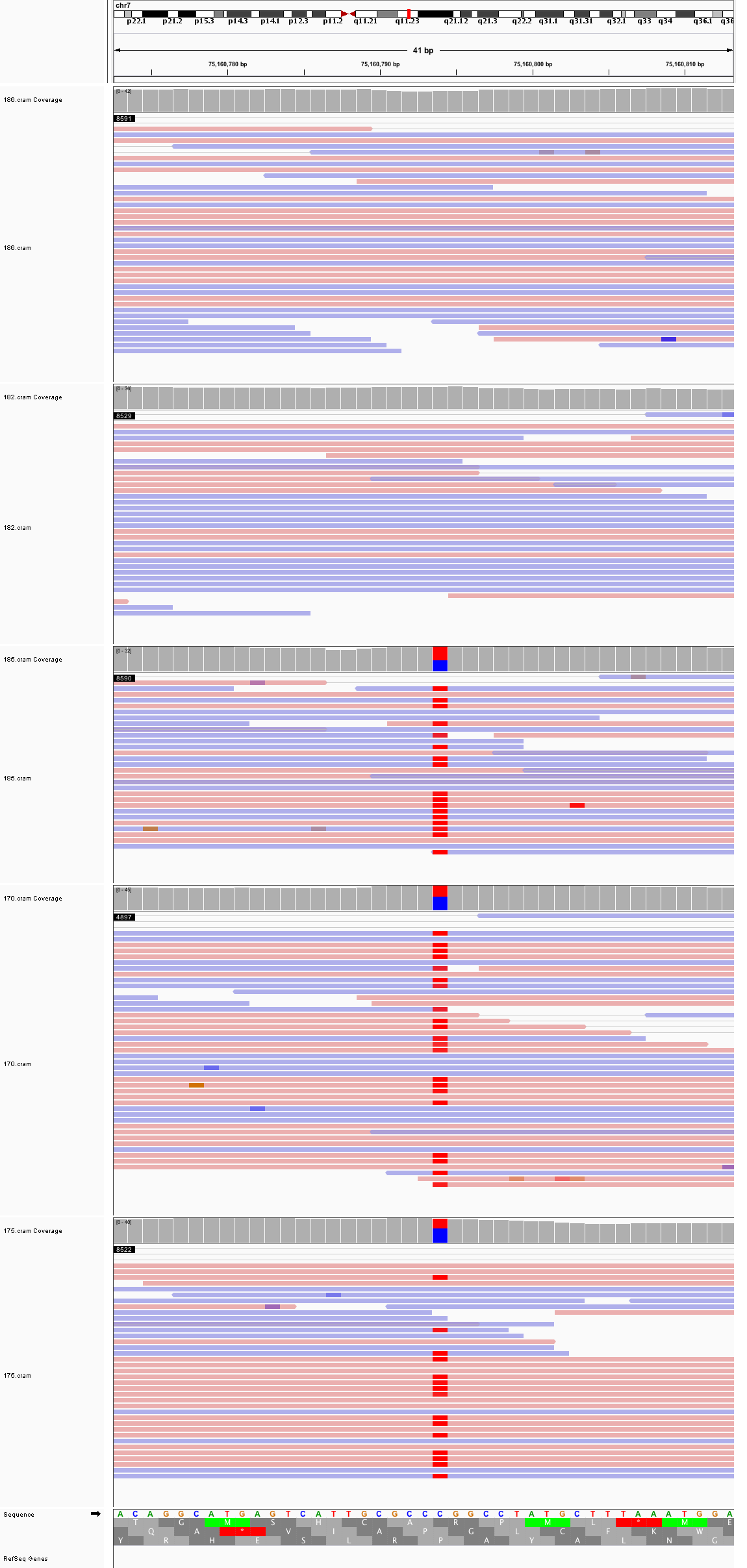

Supplement: Supplementary file 4. — All tracks below contain alignments from the third-generation children that share a DNM at the site. Reads with mapping quality <20 are filtered out, as they were not considered by our variant calling pipeline, and mismatched bases are shaded by quality score (more transparent = lower base quality). [file elife-46922-supp4.zip › supp_file_4/chr7_75,160,773_75,160,813.png]

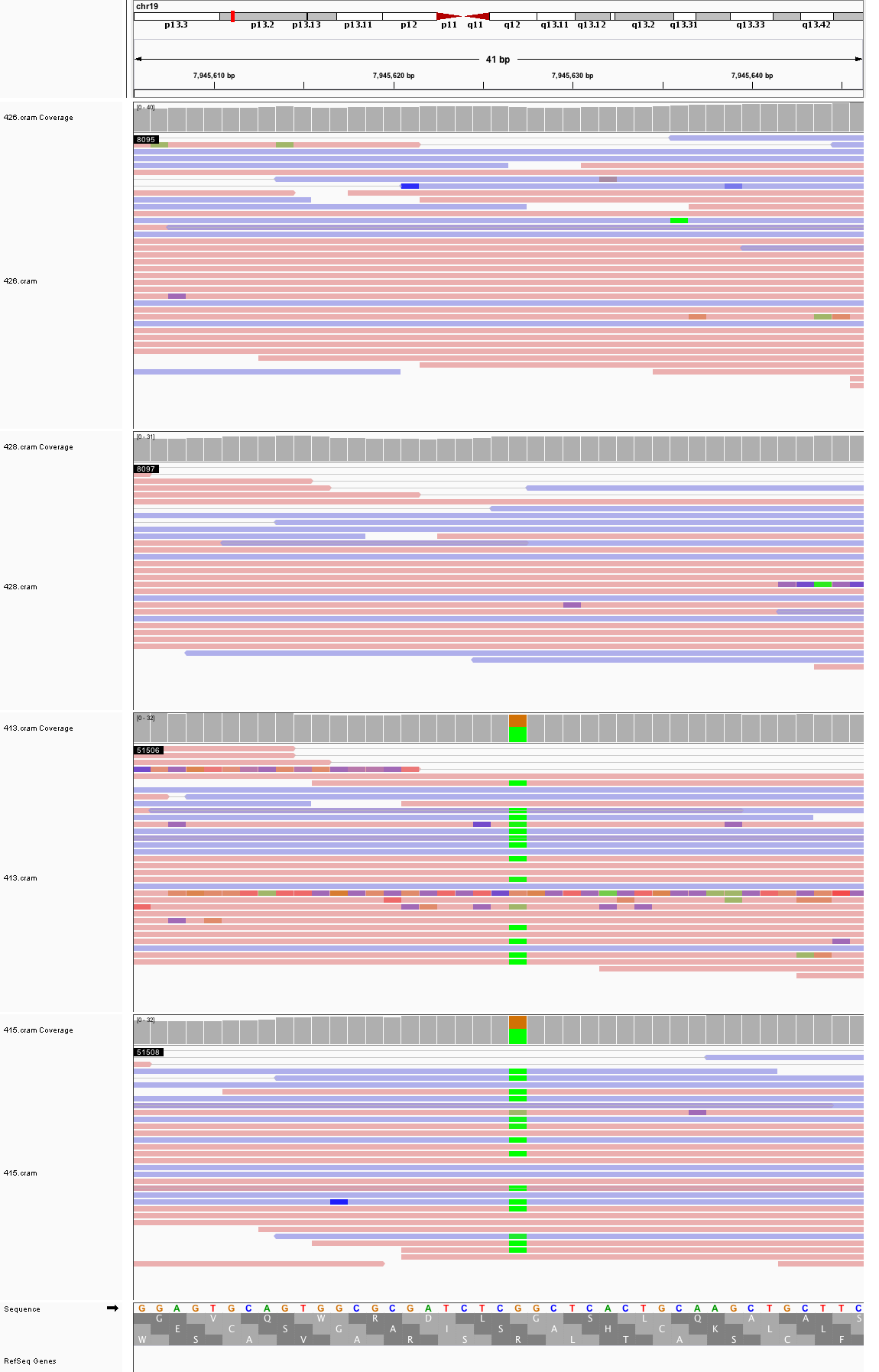

Supplement: Supplementary file 4. — All tracks below contain alignments from the third-generation children that share a DNM at the site. Reads with mapping quality <20 are filtered out, as they were not considered by our variant calling pipeline, and mismatched bases are shaded by quality score (more transparent = lower base quality). [file elife-46922-supp4.zip › supp_file_4/chr19_7,945,606_7,945,646.png]

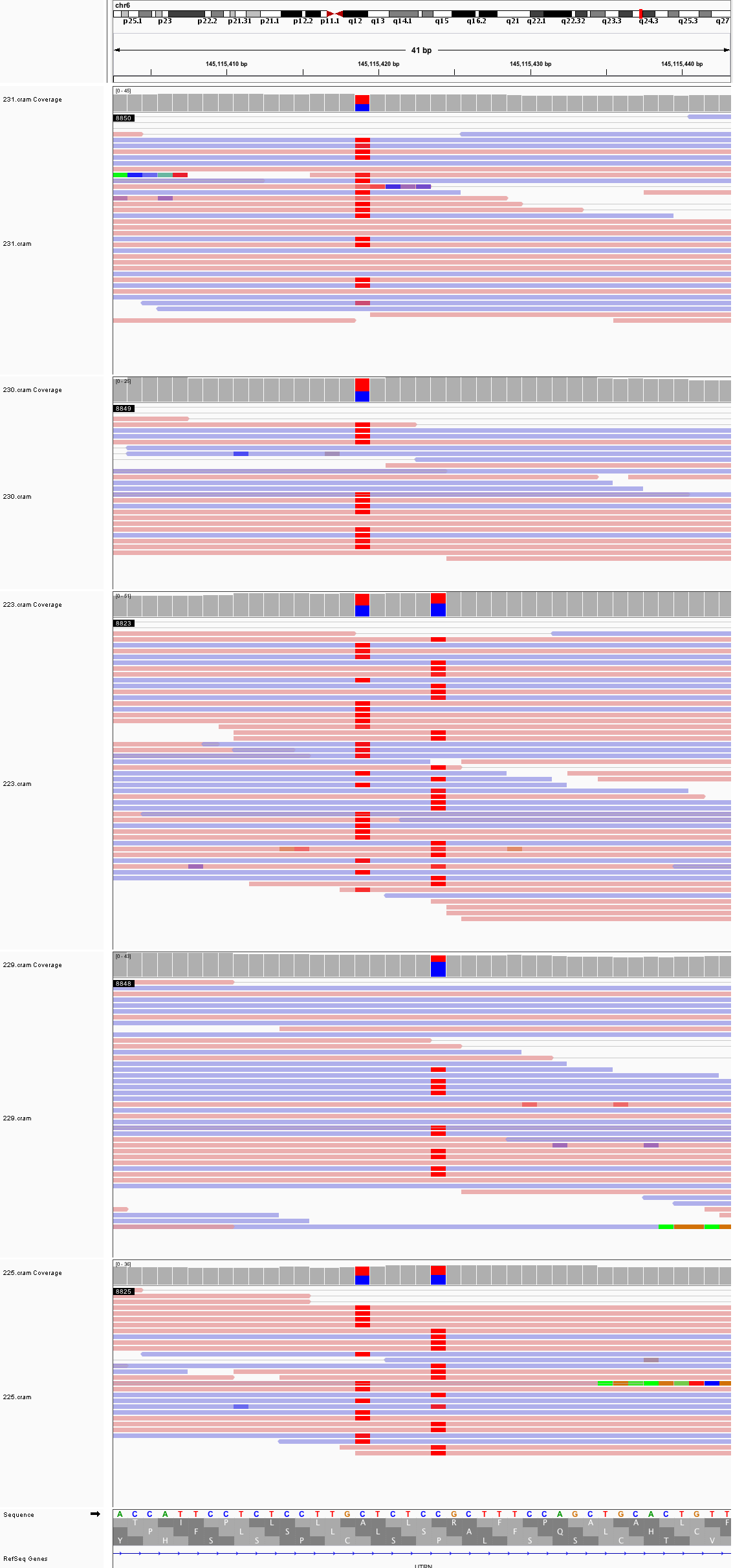

Supplement: Supplementary file 4. — All tracks below contain alignments from the third-generation children that share a DNM at the site. Reads with mapping quality <20 are filtered out, as they were not considered by our variant calling pipeline, and mismatched bases are shaded by quality score (more transparent = lower base quality). [file elife-46922-supp4.zip › supp_file_4/chr6_145,115,403_145,115,443.png]

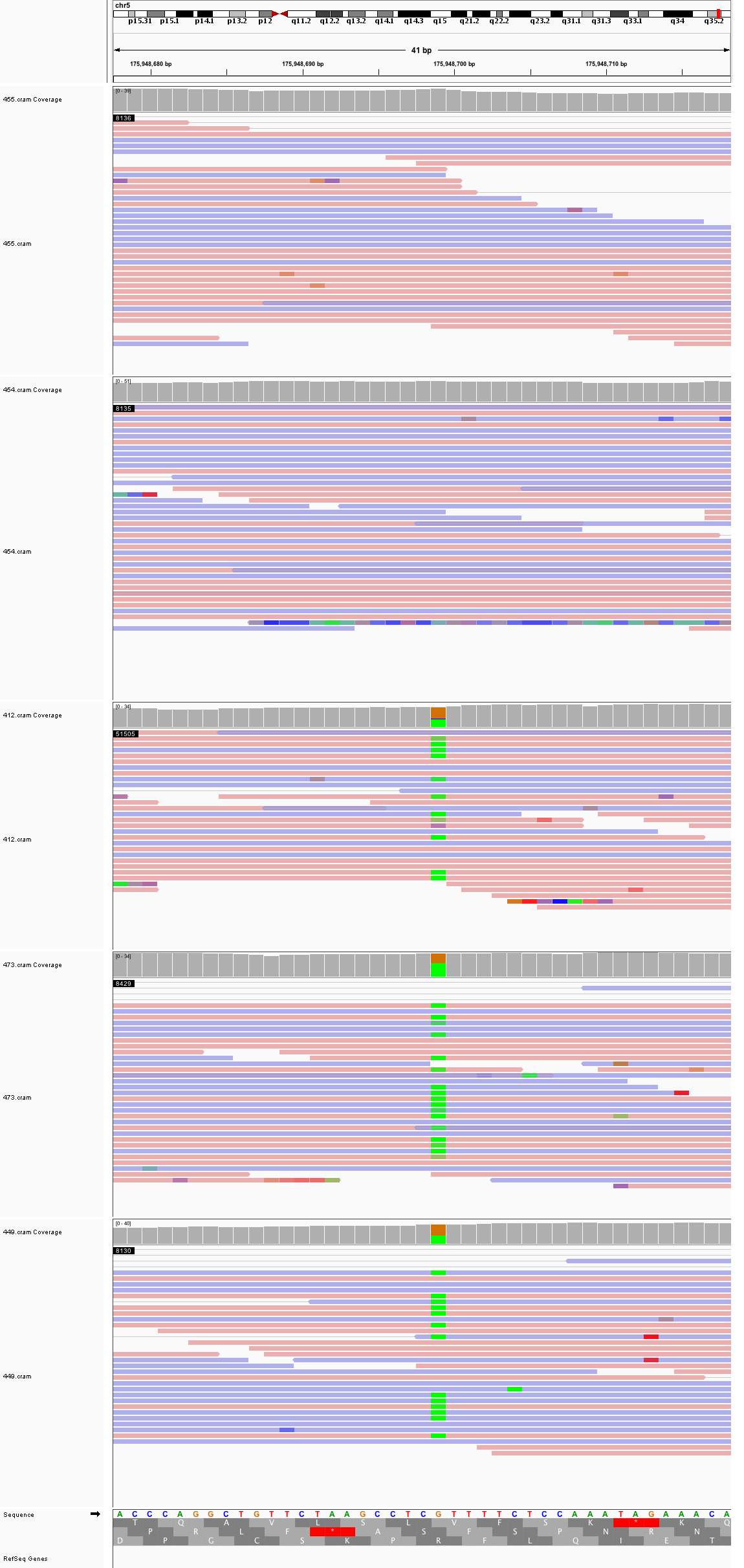

Supplement: Supplementary file 4. — All tracks below contain alignments from the third-generation children that share a DNM at the site. Reads with mapping quality <20 are filtered out, as they were not considered by our variant calling pipeline, and mismatched bases are shaded by quality score (more transparent = lower base quality). [file elife-46922-supp4.zip › supp_file_4/chr5_175,948,678_175,948,718.png]

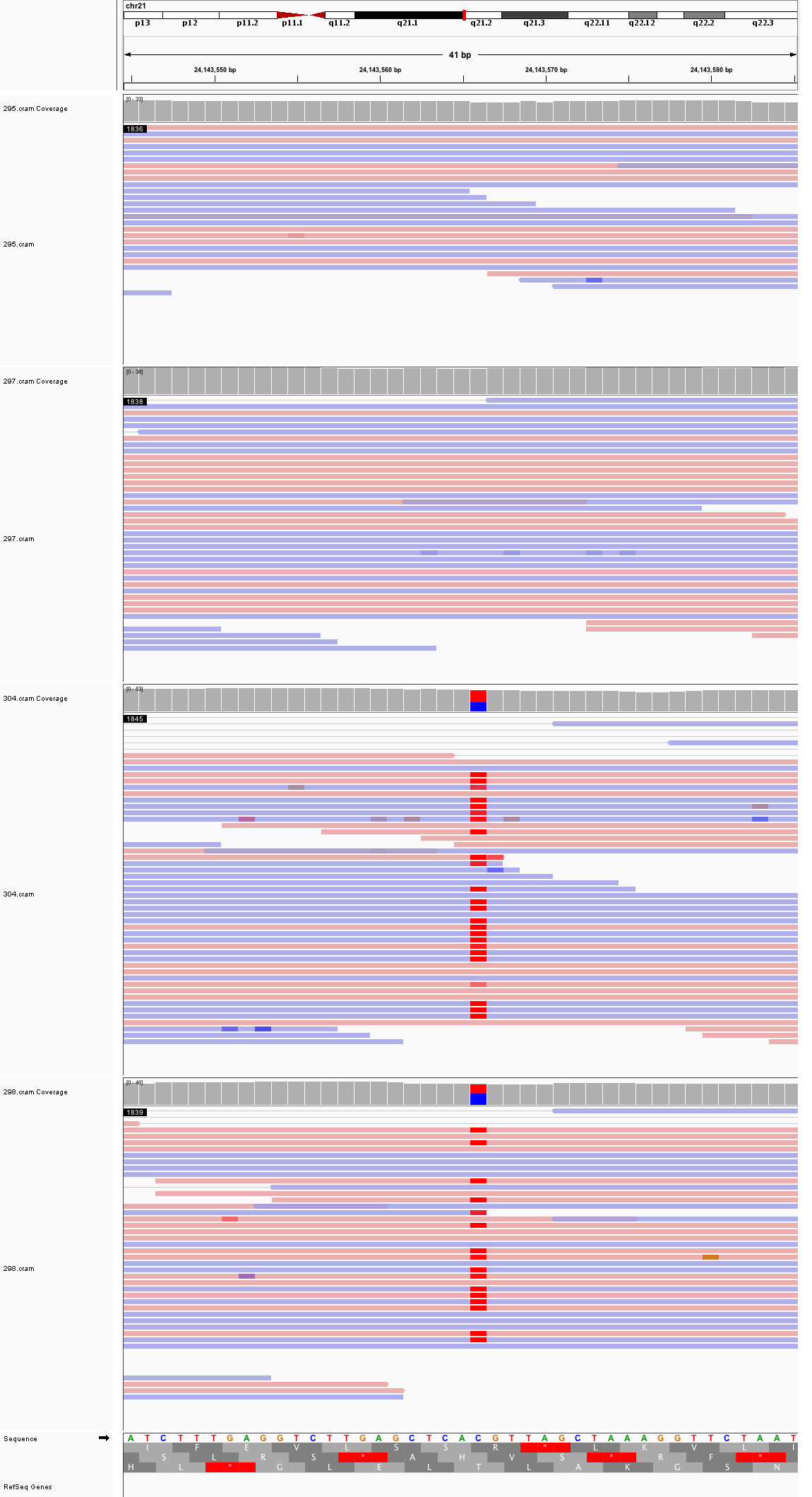

Supplement: Supplementary file 4. — All tracks below contain alignments from the third-generation children that share a DNM at the site. Reads with mapping quality <20 are filtered out, as they were not considered by our variant calling pipeline, and mismatched bases are shaded by quality score (more transparent = lower base quality). [file elife-46922-supp4.zip › supp_file_4/chr21_24,143,545_24,143,585.png]

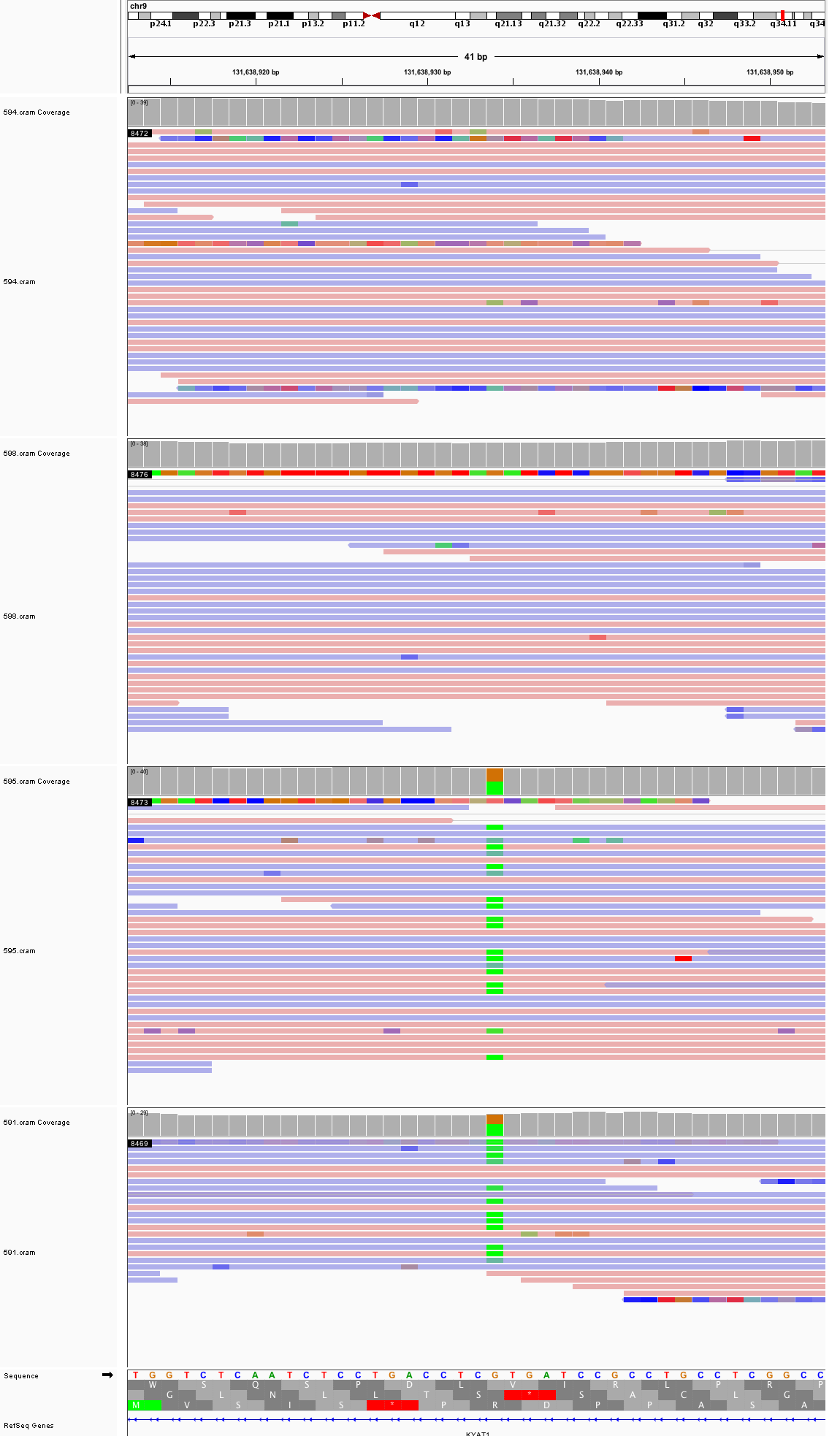

Supplement: Supplementary file 4. — All tracks below contain alignments from the third-generation children that share a DNM at the site. Reads with mapping quality <20 are filtered out, as they were not considered by our variant calling pipeline, and mismatched bases are shaded by quality score (more transparent = lower base quality). [file elife-46922-supp4.zip › supp_file_4/chr9_131,638,913_131,638,953.png]

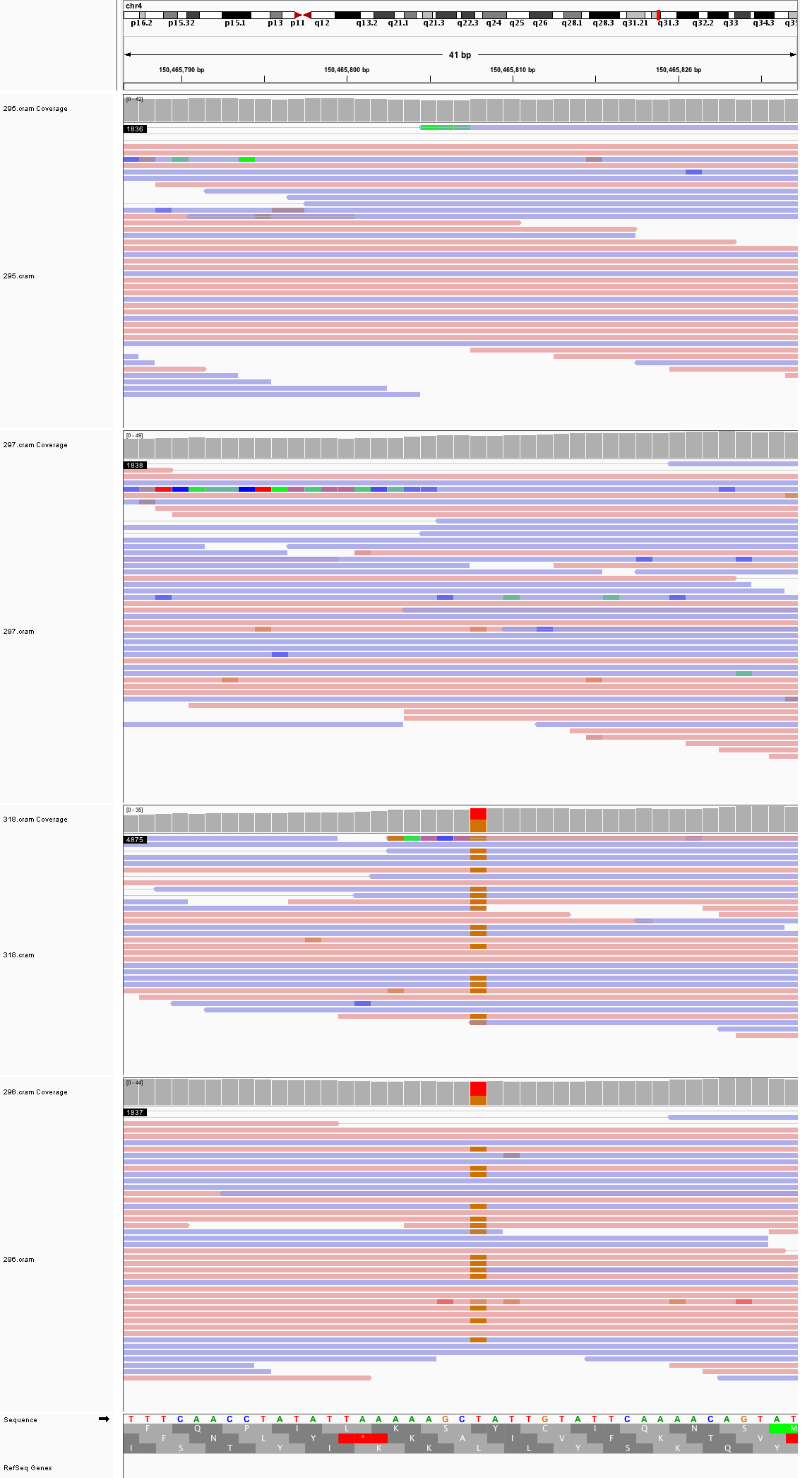

Supplement: Supplementary file 4. — All tracks below contain alignments from the third-generation children that share a DNM at the site. Reads with mapping quality <20 are filtered out, as they were not considered by our variant calling pipeline, and mismatched bases are shaded by quality score (more transparent = lower base quality). [file elife-46922-supp4.zip › supp_file_4/chr4_150,465,787_150,465,827.png]

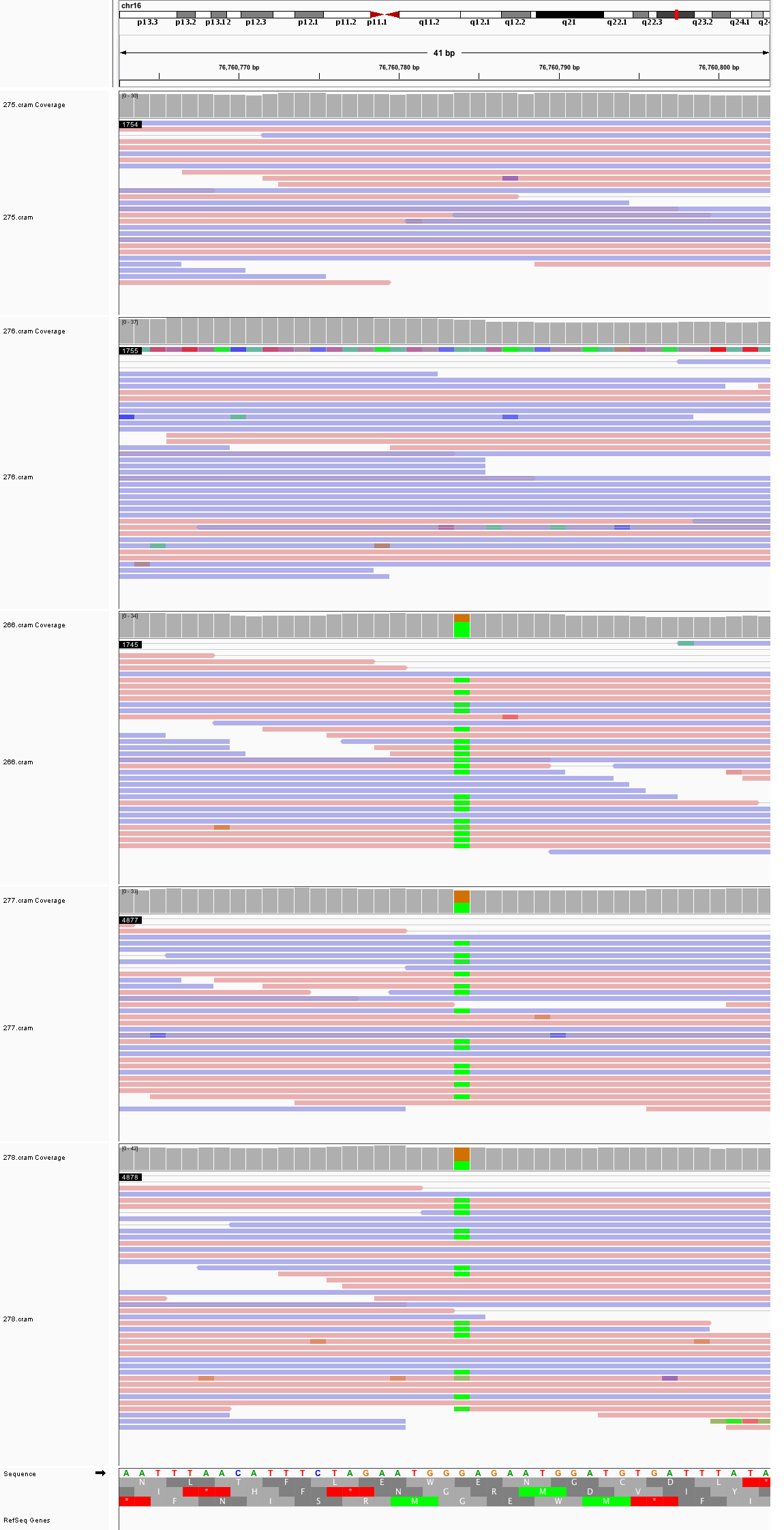

Supplement: Supplementary file 4. — All tracks below contain alignments from the third-generation children that share a DNM at the site. Reads with mapping quality <20 are filtered out, as they were not considered by our variant calling pipeline, and mismatched bases are shaded by quality score (more transparent = lower base quality). [file elife-46922-supp4.zip › supp_file_4/chr16_76,760,763_76,760,803.png]

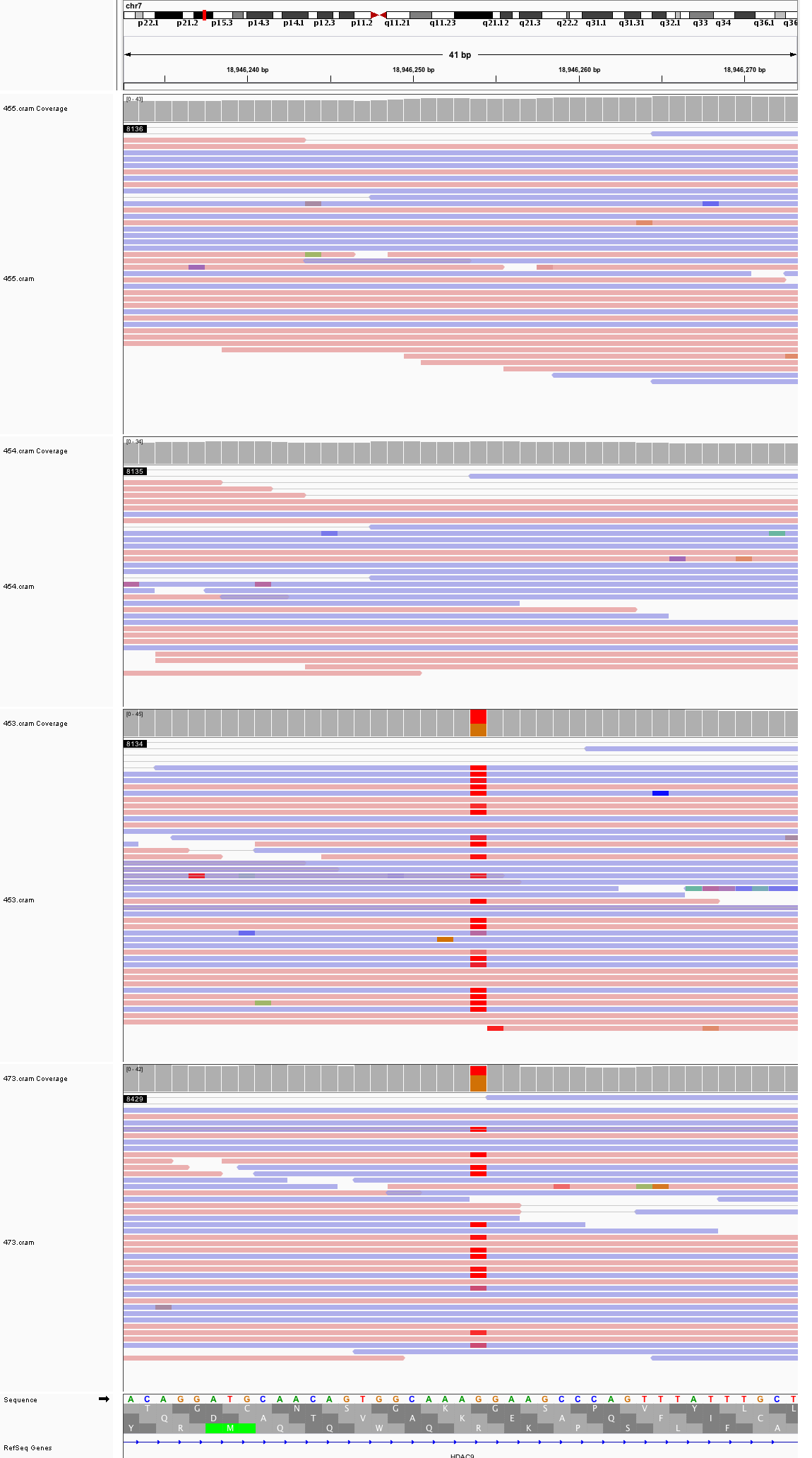

Supplement: Supplementary file 4. — All tracks below contain alignments from the third-generation children that share a DNM at the site. Reads with mapping quality <20 are filtered out, as they were not considered by our variant calling pipeline, and mismatched bases are shaded by quality score (more transparent = lower base quality). [file elife-46922-supp4.zip › supp_file_4/chr7_18,946,233_18,946,273.png]

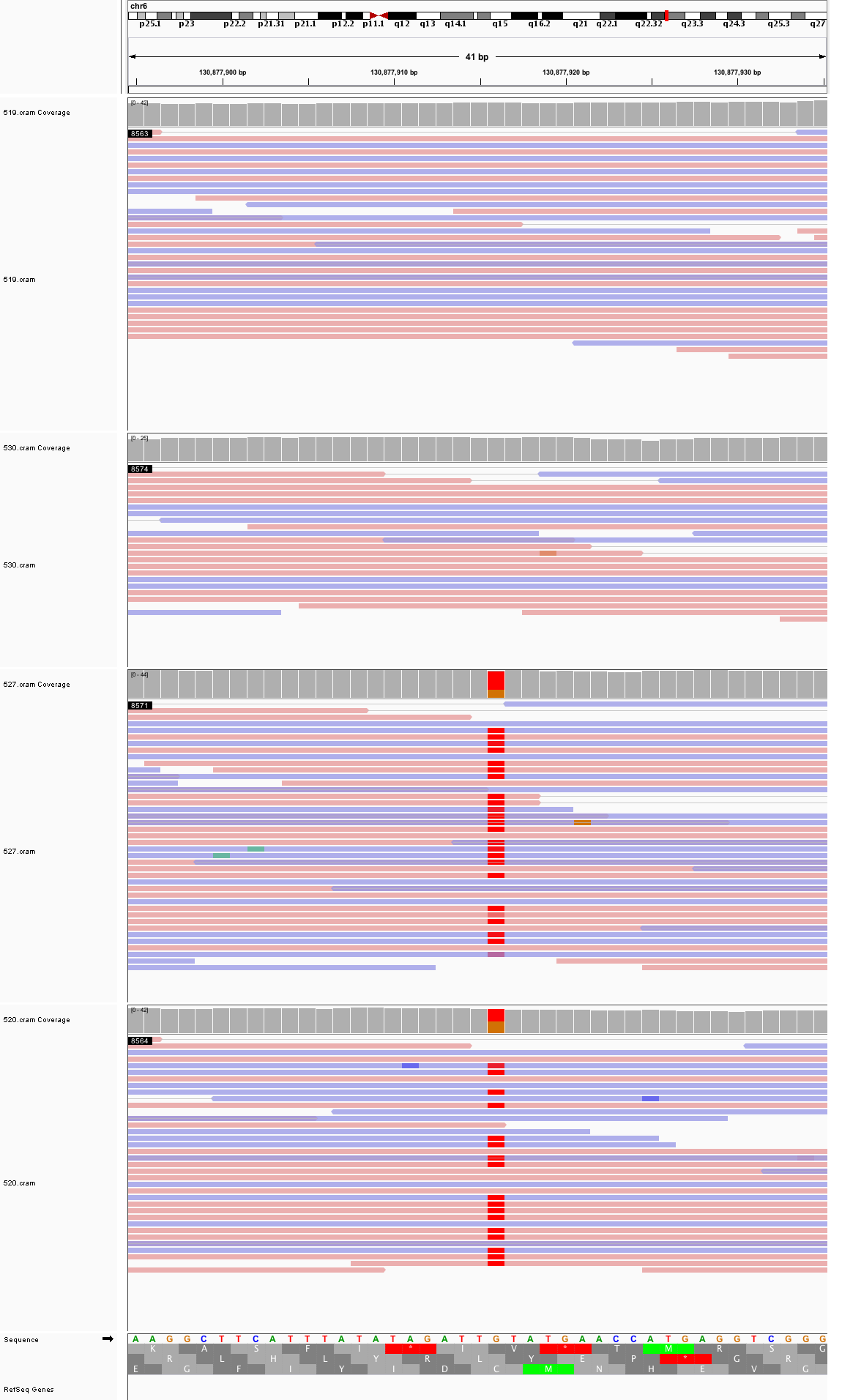

Supplement: Supplementary file 4. — All tracks below contain alignments from the third-generation children that share a DNM at the site. Reads with mapping quality <20 are filtered out, as they were not considered by our variant calling pipeline, and mismatched bases are shaded by quality score (more transparent = lower base quality). [file elife-46922-supp4.zip › supp_file_4/chr6_130,877,895_130,877,935.png]

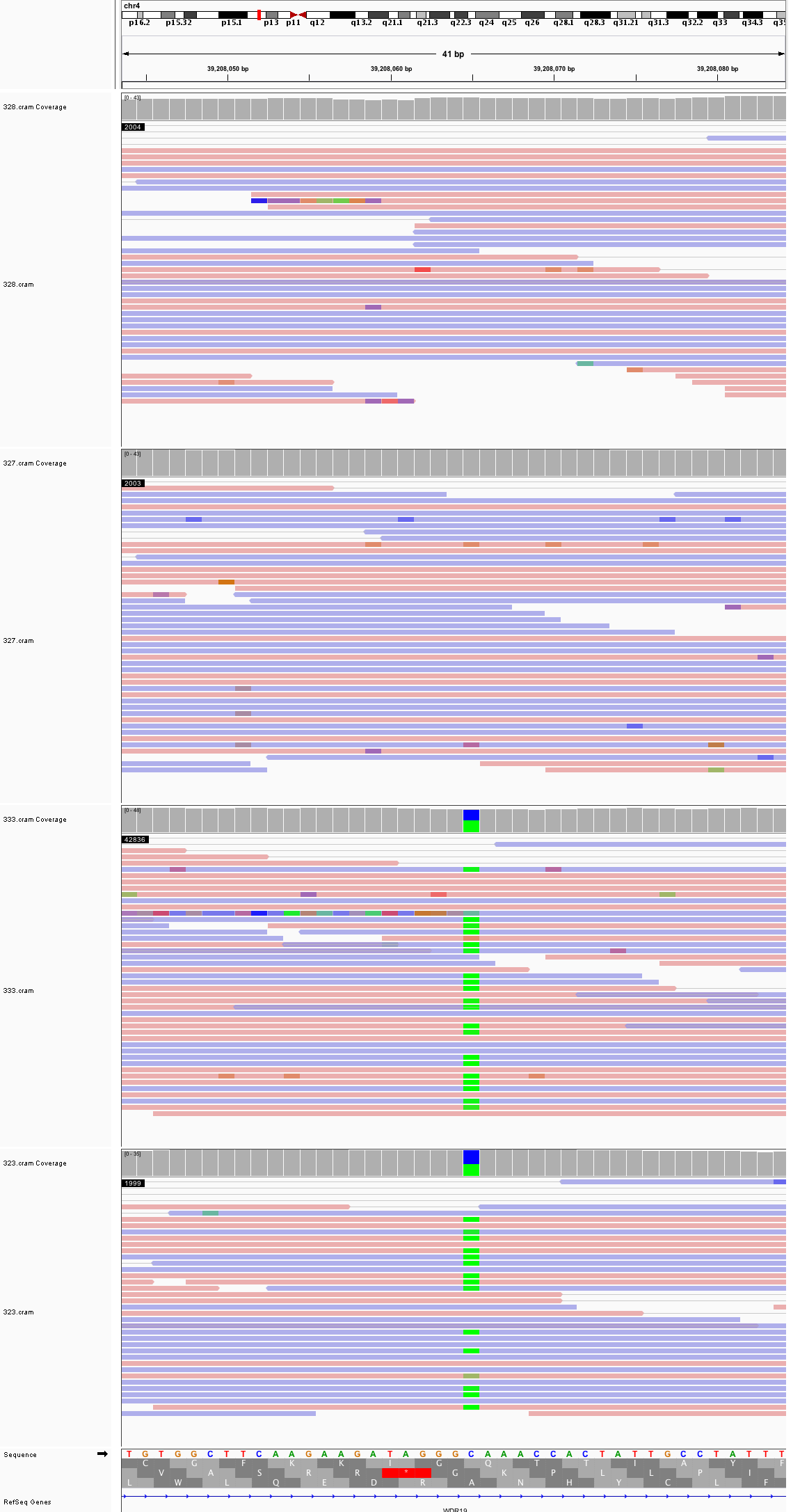

Supplement: Supplementary file 4. — All tracks below contain alignments from the third-generation children that share a DNM at the site. Reads with mapping quality <20 are filtered out, as they were not considered by our variant calling pipeline, and mismatched bases are shaded by quality score (more transparent = lower base quality). [file elife-46922-supp4.zip › supp_file_4/chr4_39,208,044_39,208,084.png]

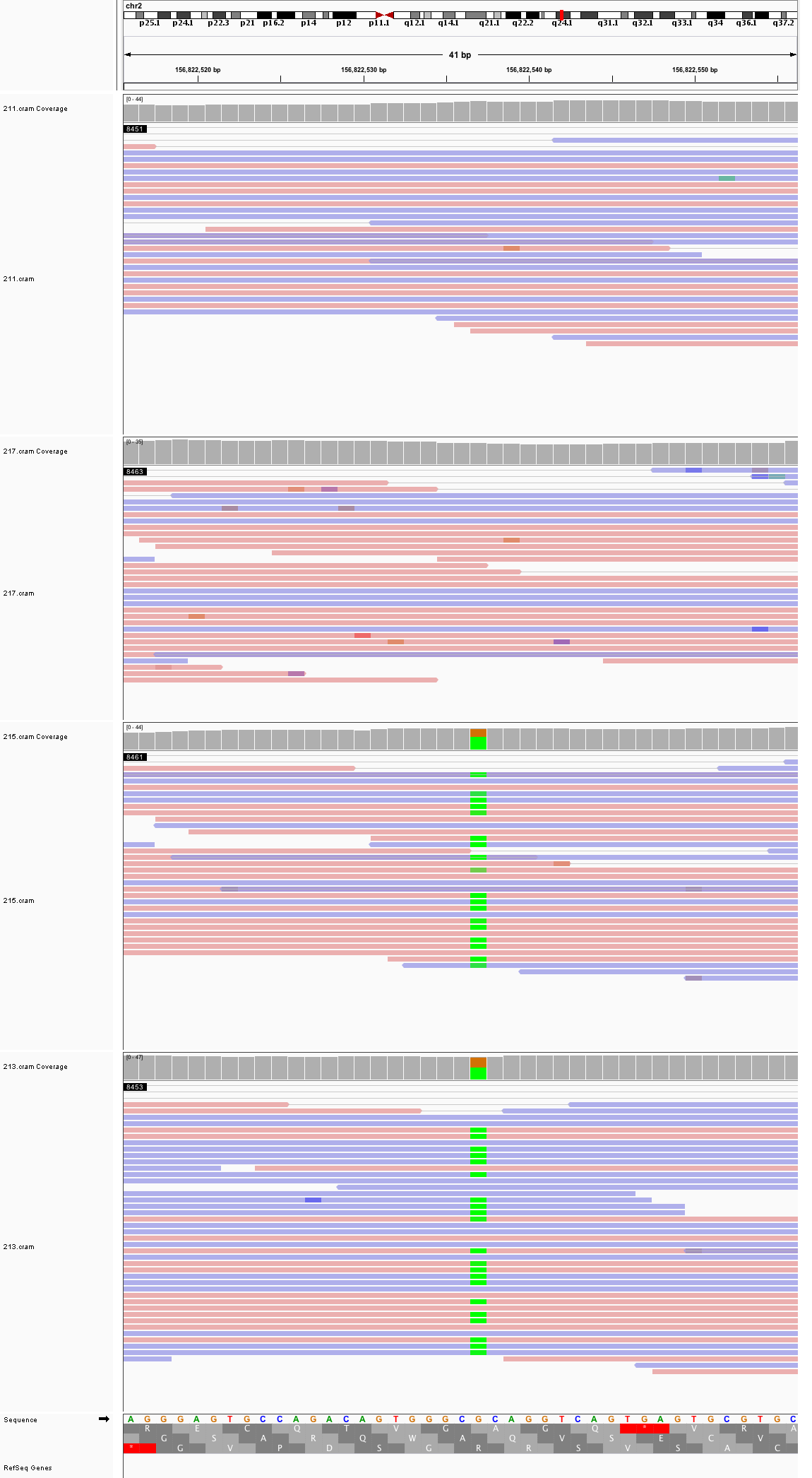

Supplement: Supplementary file 4. — All tracks below contain alignments from the third-generation children that share a DNM at the site. Reads with mapping quality <20 are filtered out, as they were not considered by our variant calling pipeline, and mismatched bases are shaded by quality score (more transparent = lower base quality). [file elife-46922-supp4.zip › supp_file_4/chr2_156,822,516_156,822,556.png]

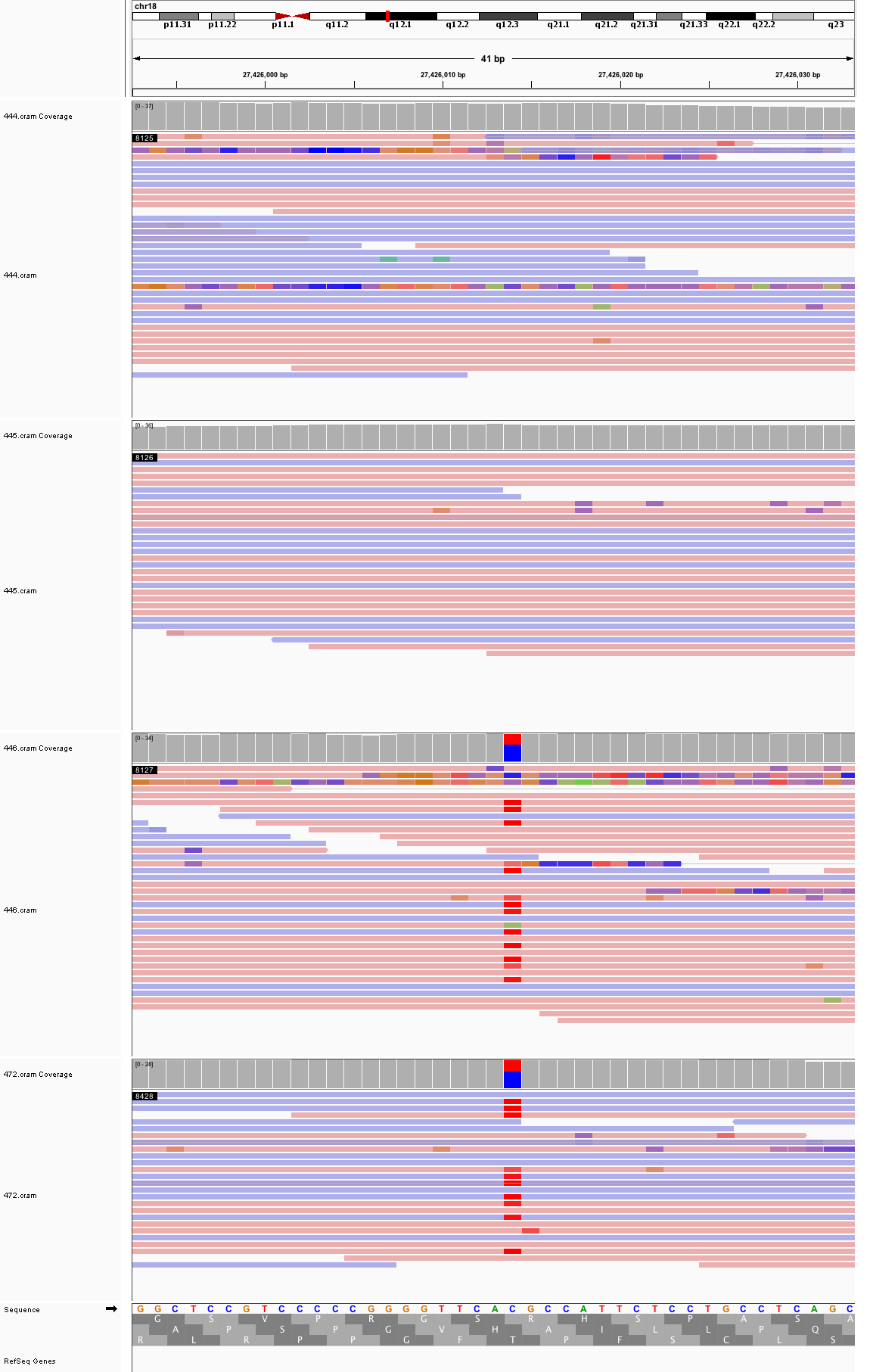

Supplement: Supplementary file 4. — All tracks below contain alignments from the third-generation children that share a DNM at the site. Reads with mapping quality <20 are filtered out, as they were not considered by our variant calling pipeline, and mismatched bases are shaded by quality score (more transparent = lower base quality). [file elife-46922-supp4.zip › supp_file_4/chr18_27,425,993_27,426,033.png]

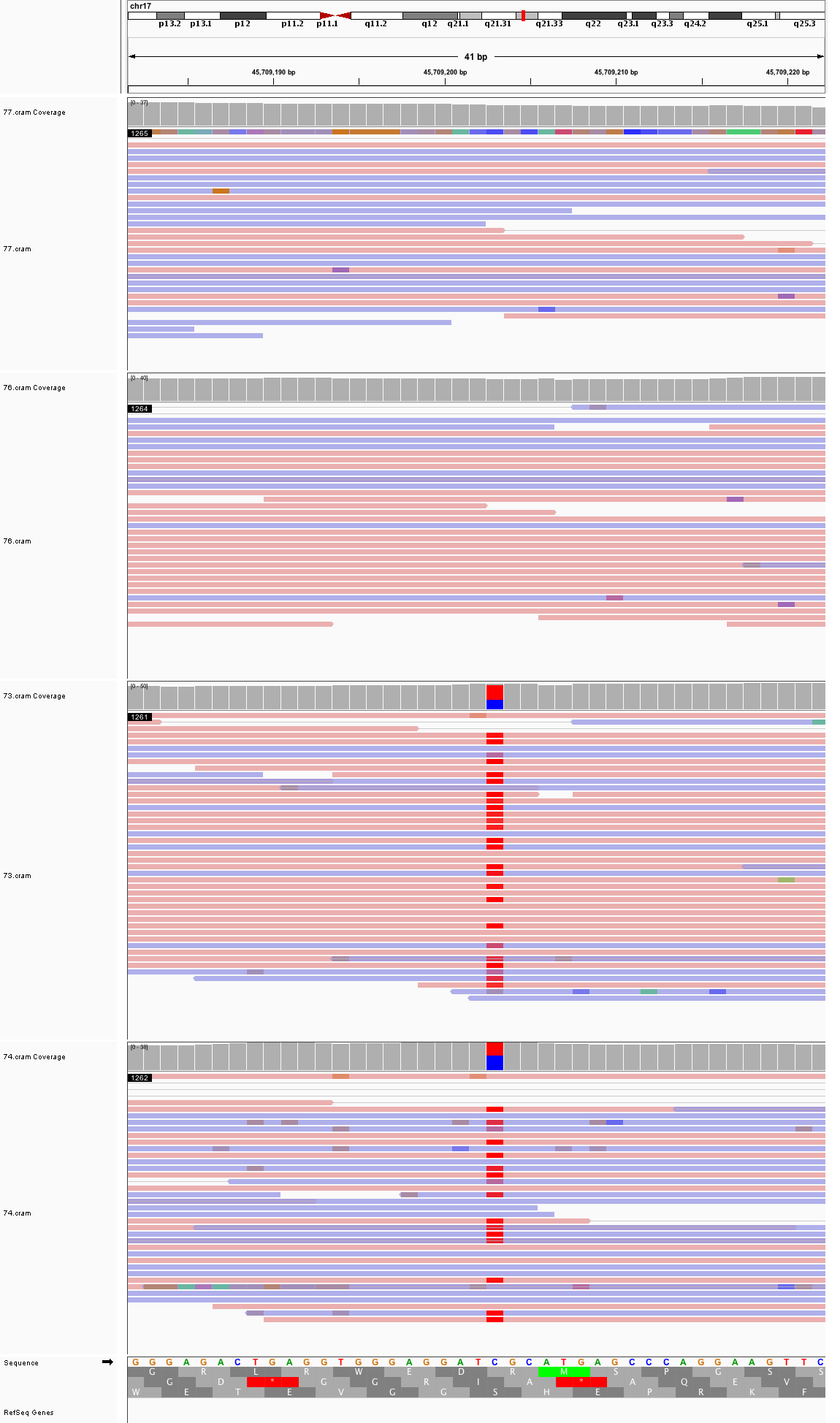

Supplement: Supplementary file 4. — All tracks below contain alignments from the third-generation children that share a DNM at the site. Reads with mapping quality <20 are filtered out, as they were not considered by our variant calling pipeline, and mismatched bases are shaded by quality score (more transparent = lower base quality). [file elife-46922-supp4.zip › supp_file_4/chr17_45,709,182_45,709,222.png]

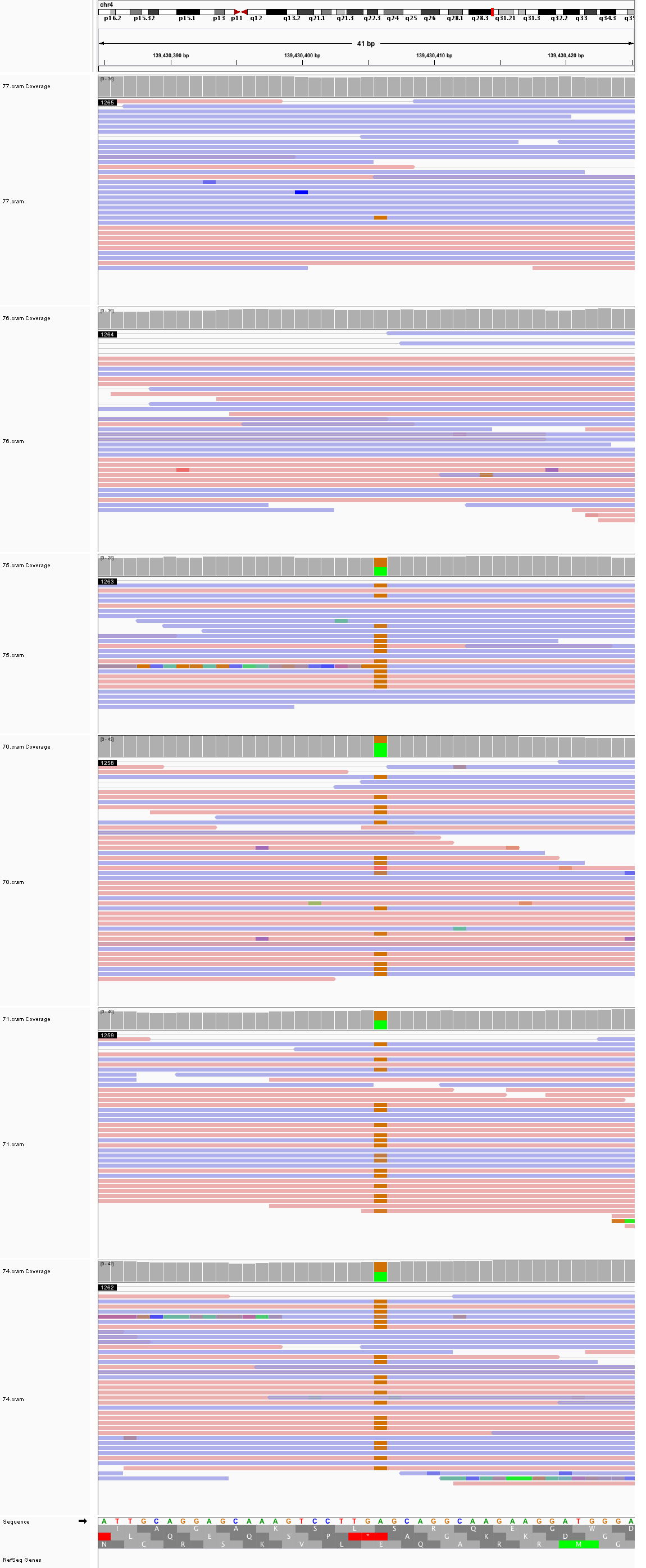

Supplement: Supplementary file 4. — All tracks below contain alignments from the third-generation children that share a DNM at the site. Reads with mapping quality <20 are filtered out, as they were not considered by our variant calling pipeline, and mismatched bases are shaded by quality score (more transparent = lower base quality). [file elife-46922-supp4.zip › supp_file_4/chr4_139,430,385_139,430,425.png]

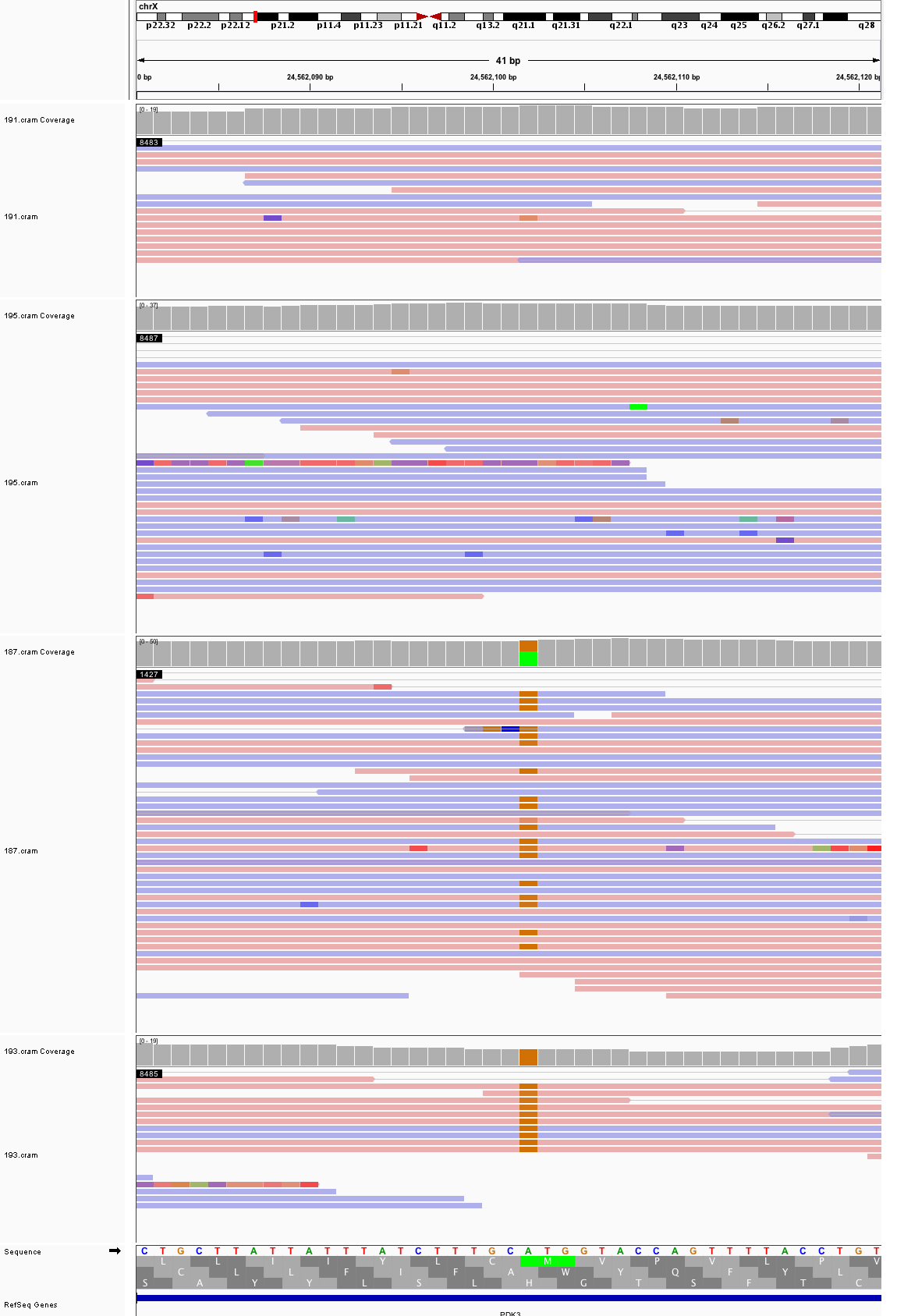

Supplement: Supplementary file 4. — All tracks below contain alignments from the third-generation children that share a DNM at the site. Reads with mapping quality <20 are filtered out, as they were not considered by our variant calling pipeline, and mismatched bases are shaded by quality score (more transparent = lower base quality). [file elife-46922-supp4.zip › supp_file_4/chrX_24,562,081_24,562,121.png]

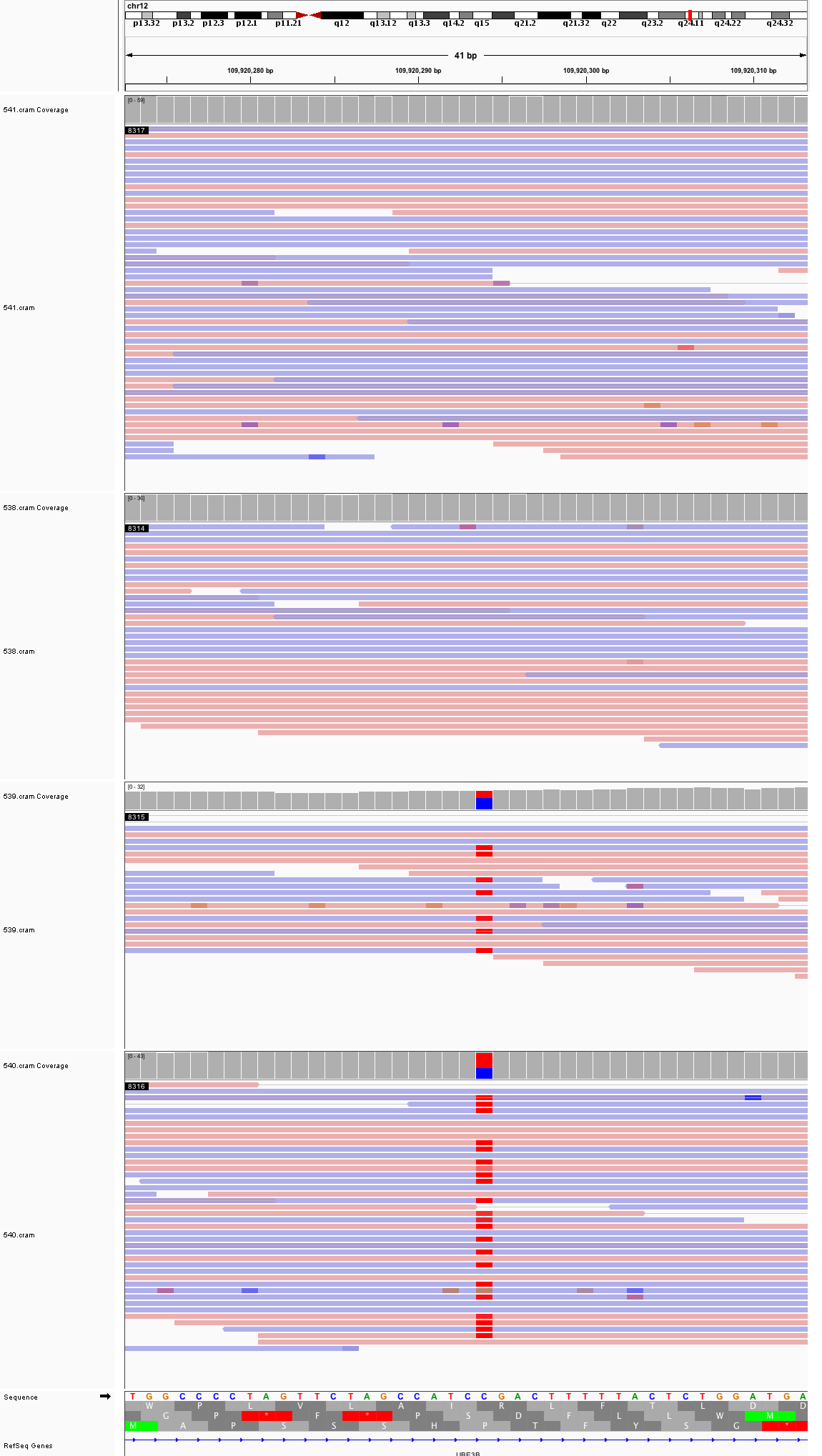

Supplement: Supplementary file 4. — All tracks below contain alignments from the third-generation children that share a DNM at the site. Reads with mapping quality <20 are filtered out, as they were not considered by our variant calling pipeline, and mismatched bases are shaded by quality score (more transparent = lower base quality). [file elife-46922-supp4.zip › supp_file_4/chr12_109,920,273_109,920,313.png]

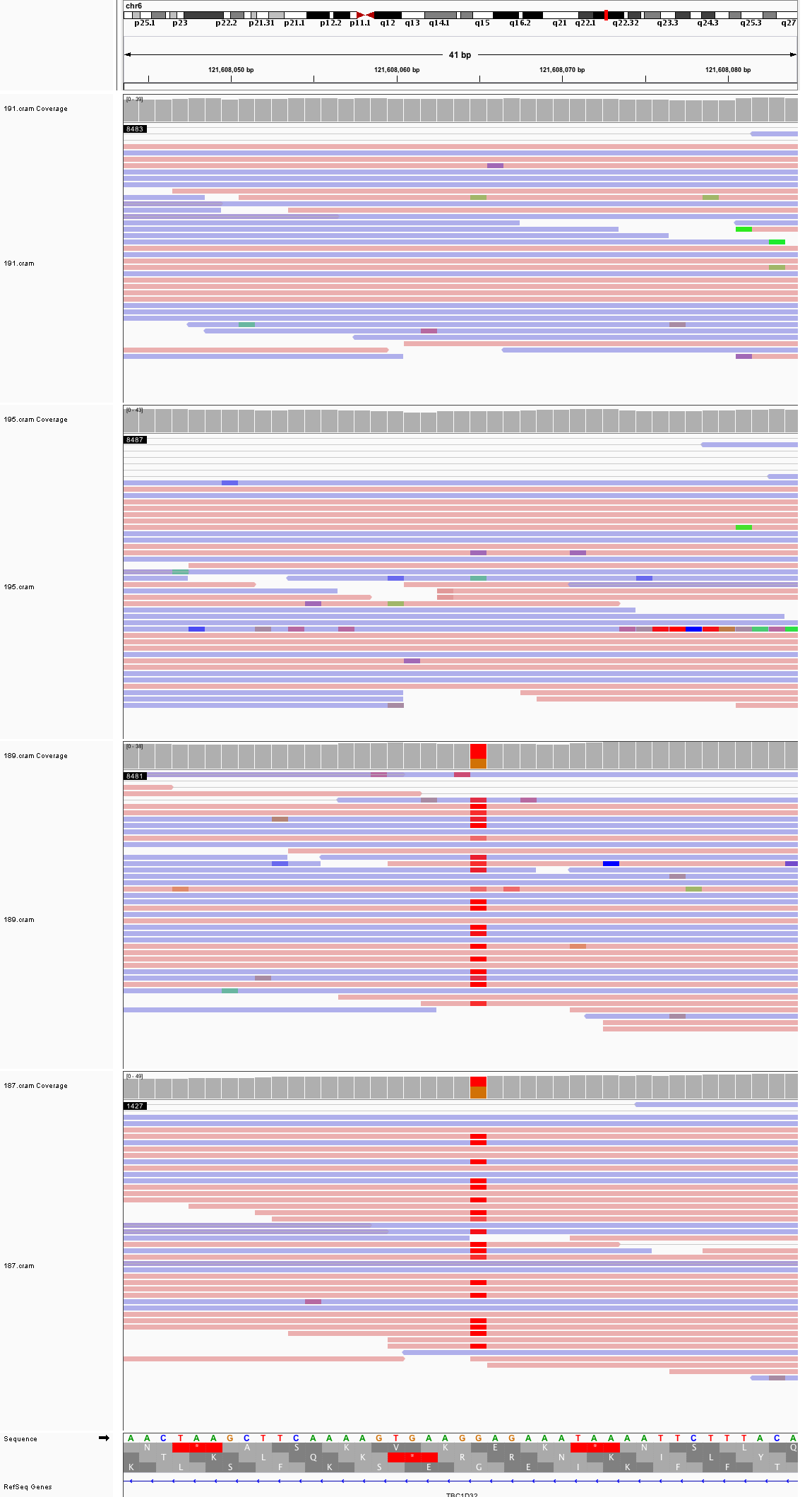

Supplement: Supplementary file 4. — All tracks below contain alignments from the third-generation children that share a DNM at the site. Reads with mapping quality <20 are filtered out, as they were not considered by our variant calling pipeline, and mismatched bases are shaded by quality score (more transparent = lower base quality). [file elife-46922-supp4.zip › supp_file_4/chr6_121,608,044_121,608,084.png]

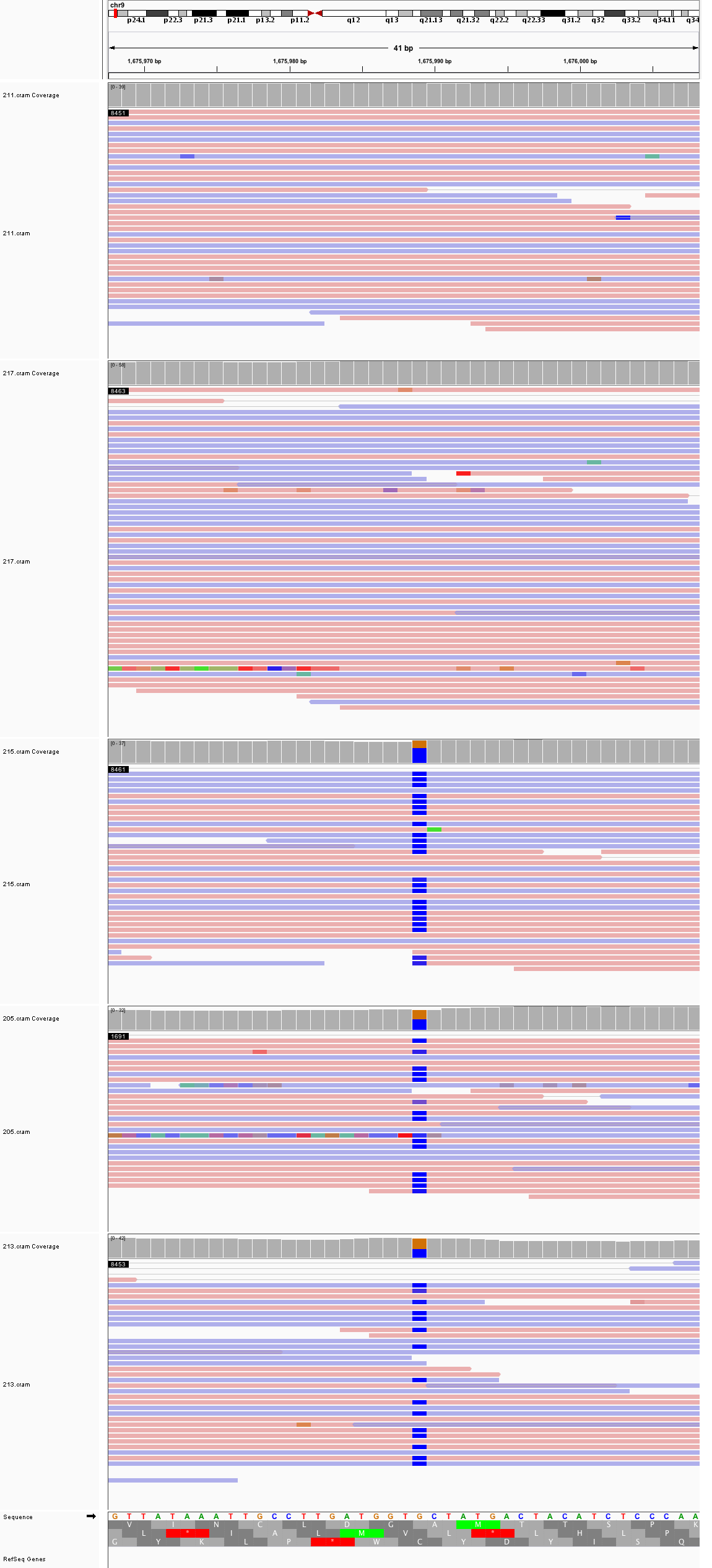

Supplement: Supplementary file 4. — All tracks below contain alignments from the third-generation children that share a DNM at the site. Reads with mapping quality <20 are filtered out, as they were not considered by our variant calling pipeline, and mismatched bases are shaded by quality score (more transparent = lower base quality). [file elife-46922-supp4.zip › supp_file_4/chr9_1,675,968_1,676,008.png]

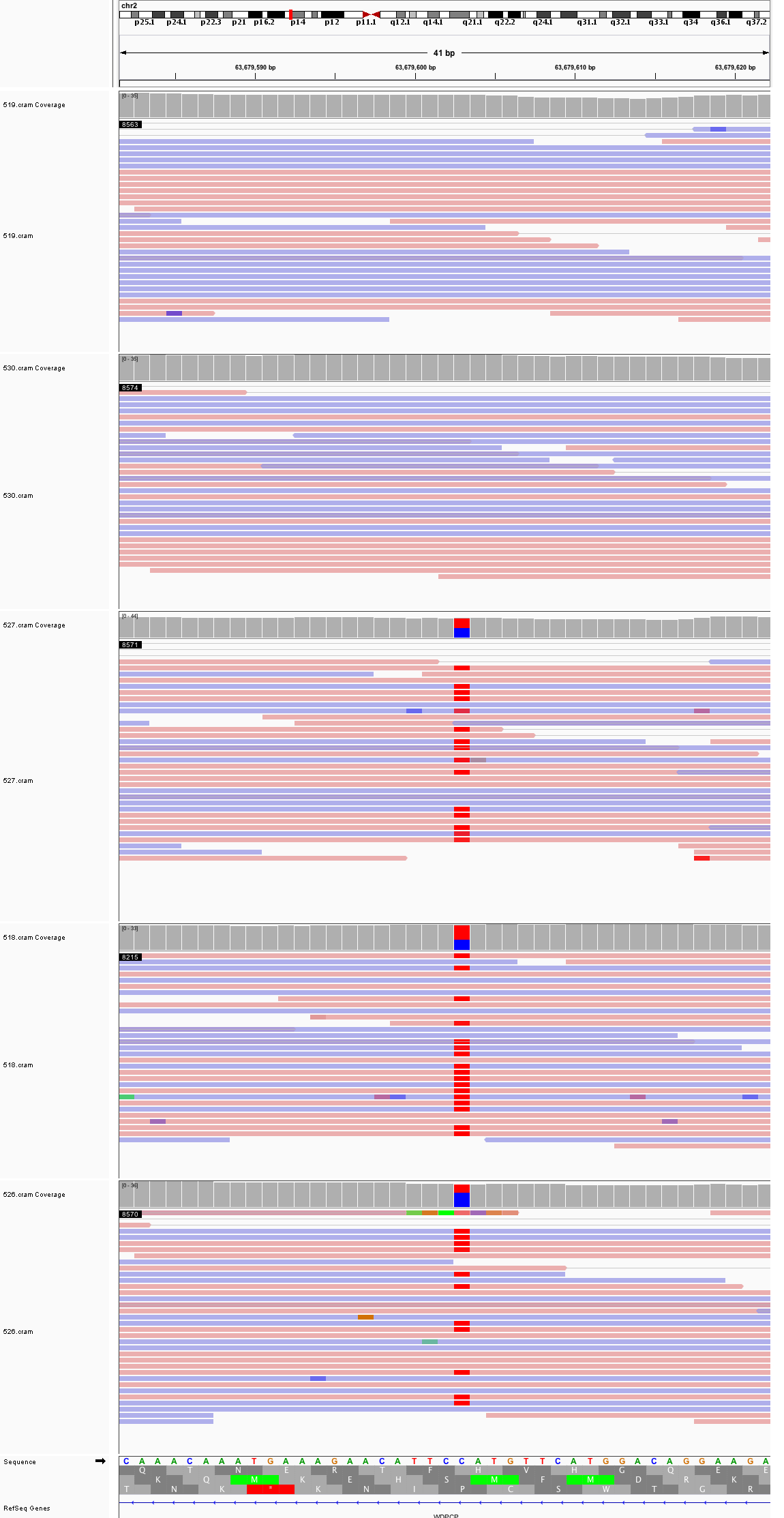

Supplement: Supplementary file 4. — All tracks below contain alignments from the third-generation children that share a DNM at the site. Reads with mapping quality <20 are filtered out, as they were not considered by our variant calling pipeline, and mismatched bases are shaded by quality score (more transparent = lower base quality). [file elife-46922-supp4.zip › supp_file_4/chr2_63,679,582_63,679,622.png]

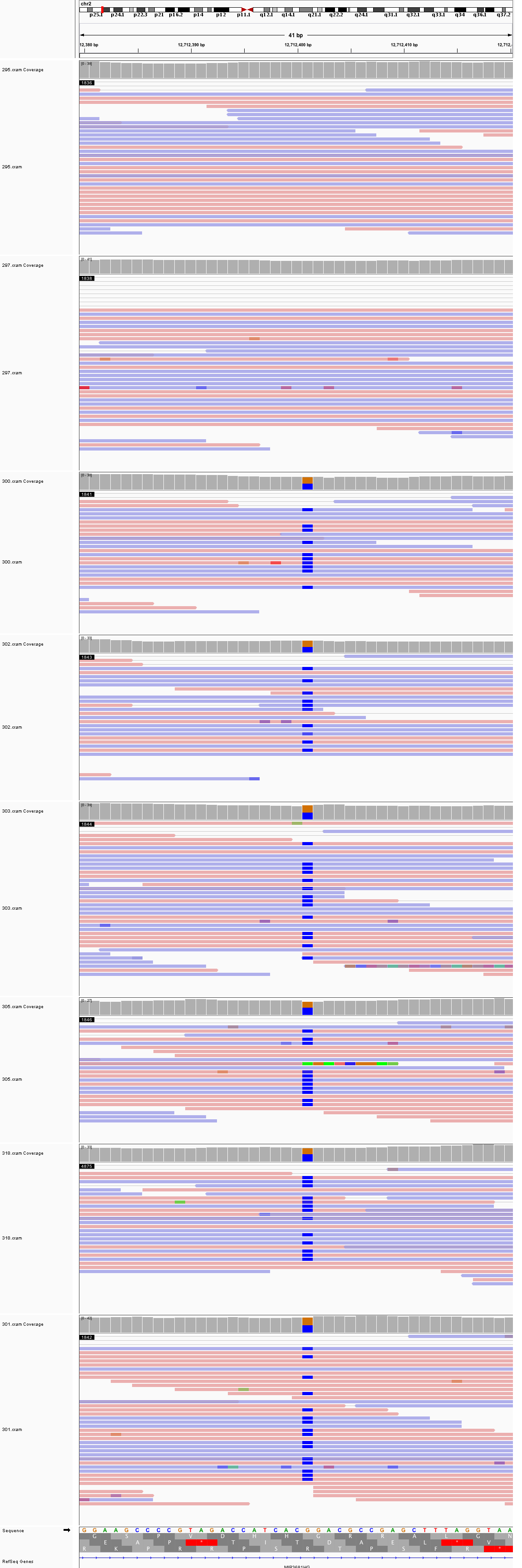

Supplement: Supplementary file 4. — All tracks below contain alignments from the third-generation children that share a DNM at the site. Reads with mapping quality <20 are filtered out, as they were not considered by our variant calling pipeline, and mismatched bases are shaded by quality score (more transparent = lower base quality). [file elife-46922-supp4.zip › supp_file_4/chr2_12,712,380_12,712,420.png]

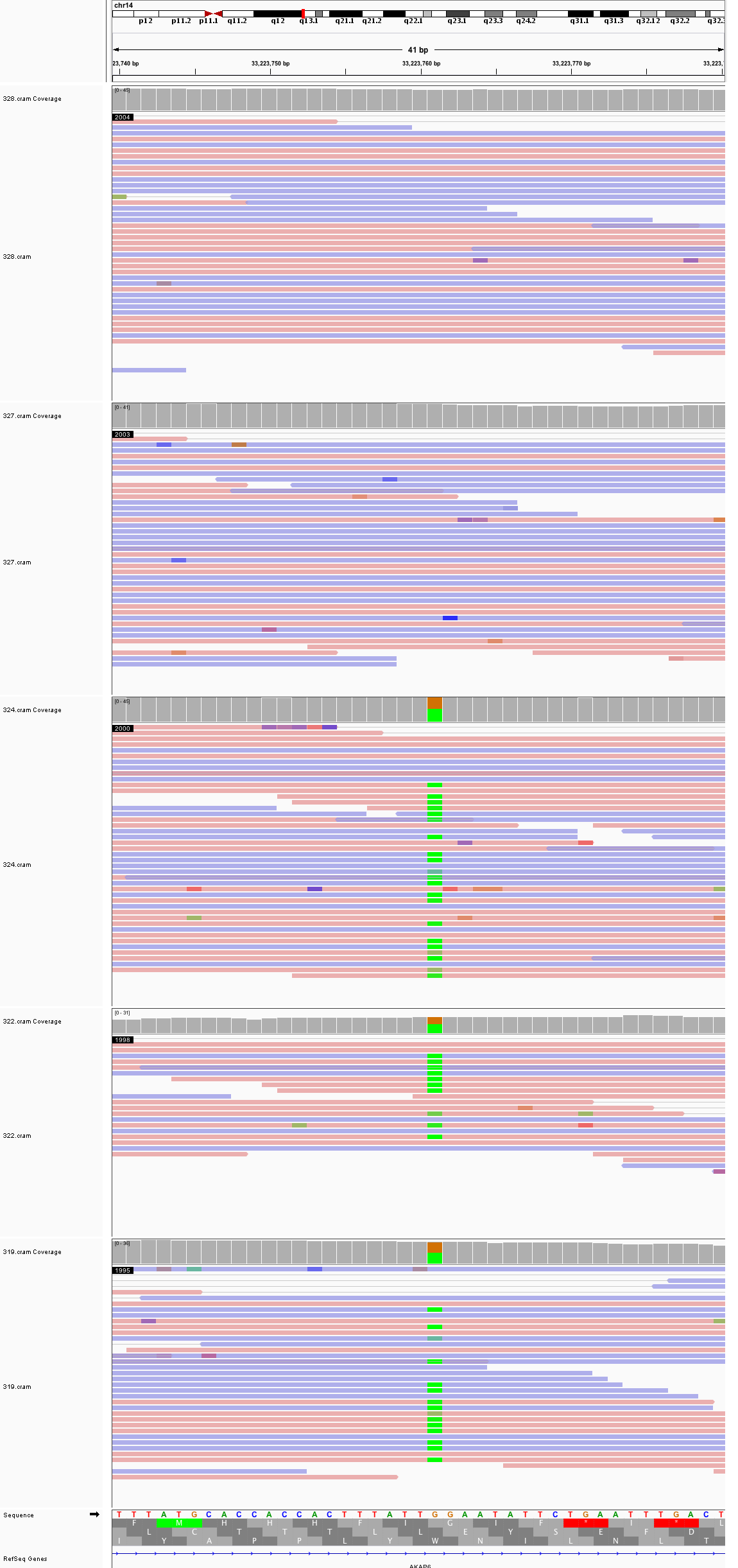

Supplement: Supplementary file 4. — All tracks below contain alignments from the third-generation children that share a DNM at the site. Reads with mapping quality <20 are filtered out, as they were not considered by our variant calling pipeline, and mismatched bases are shaded by quality score (more transparent = lower base quality). [file elife-46922-supp4.zip › supp_file_4/chr14_33,223,740_33,223,780.png]

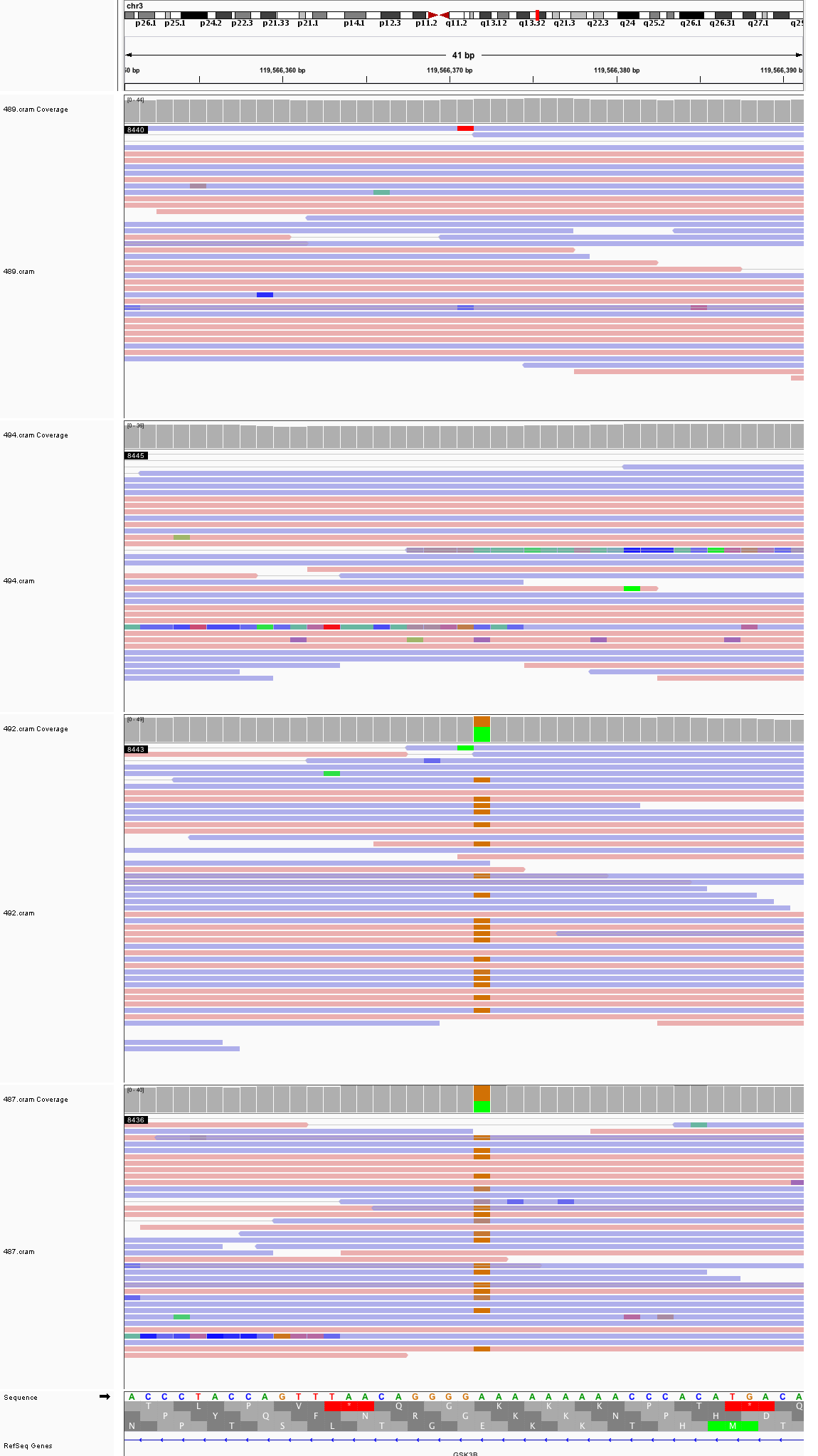

Supplement: Supplementary file 4. — All tracks below contain alignments from the third-generation children that share a DNM at the site. Reads with mapping quality <20 are filtered out, as they were not considered by our variant calling pipeline, and mismatched bases are shaded by quality score (more transparent = lower base quality). [file elife-46922-supp4.zip › supp_file_4/chr3_119,566,351_119,566,391.png]

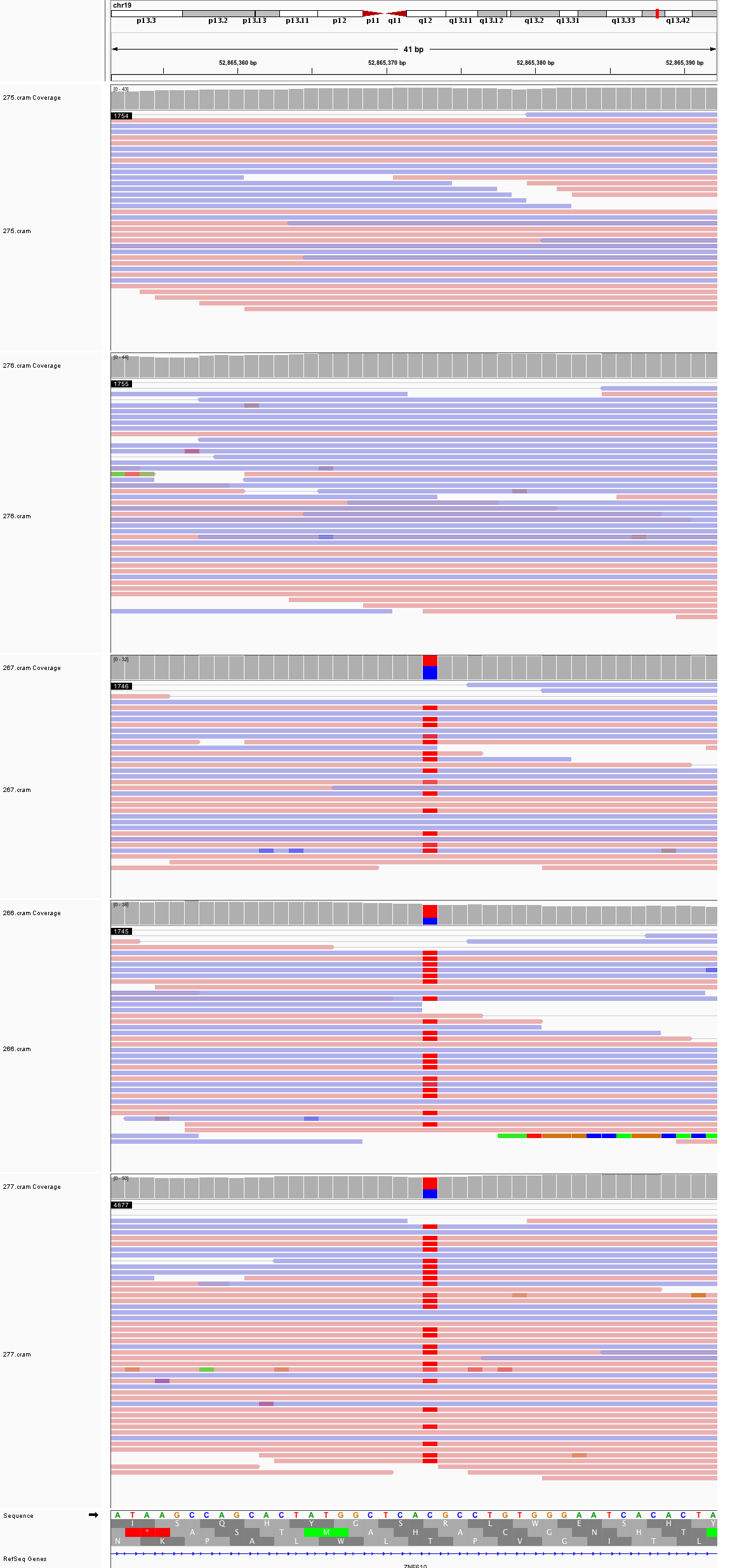

Supplement: Supplementary file 4. — All tracks below contain alignments from the third-generation children that share a DNM at the site. Reads with mapping quality <20 are filtered out, as they were not considered by our variant calling pipeline, and mismatched bases are shaded by quality score (more transparent = lower base quality). [file elife-46922-supp4.zip › supp_file_4/chr19_52,865,352_52,865,392.png]

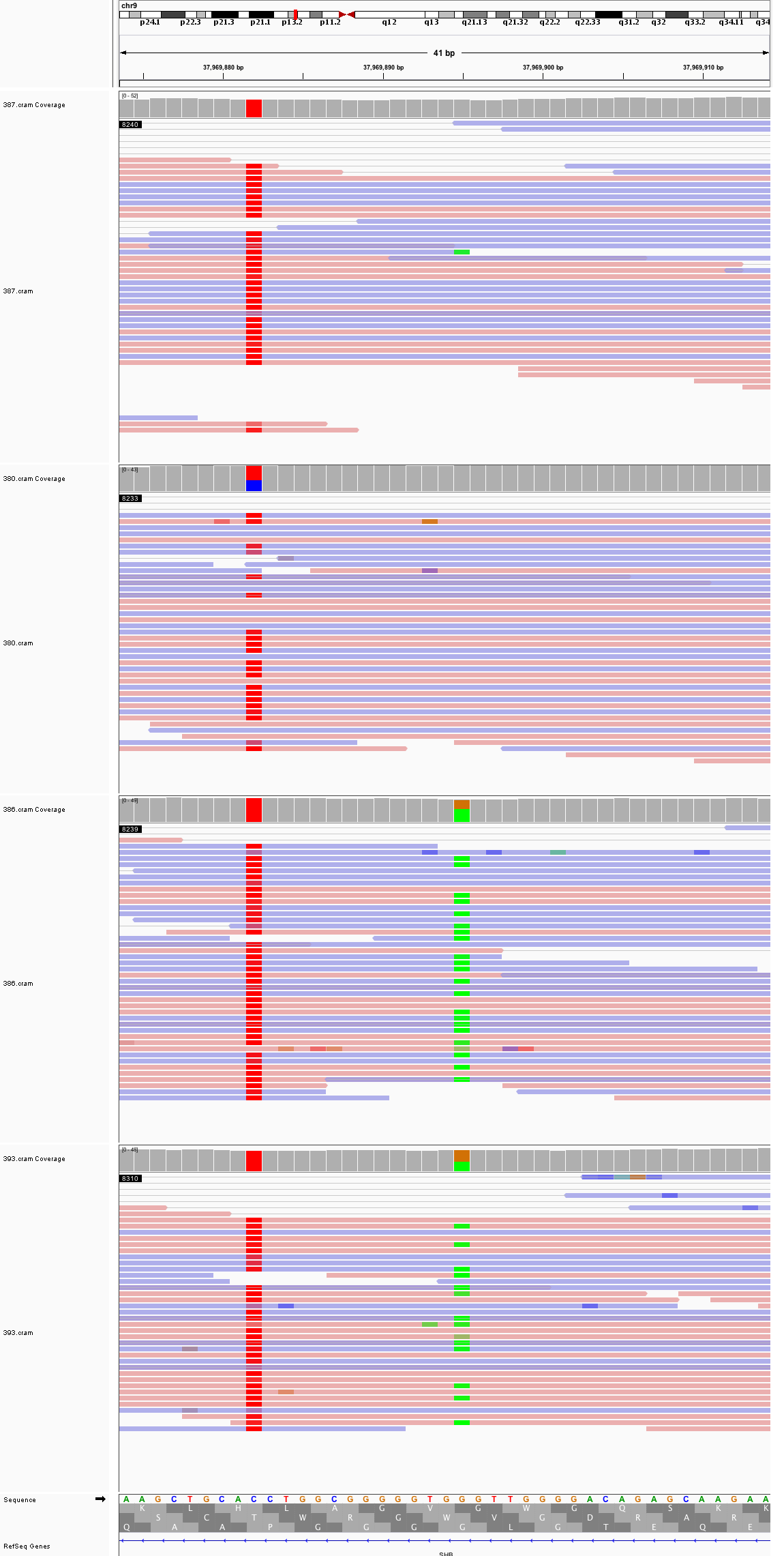

Supplement: Supplementary file 4. — All tracks below contain alignments from the third-generation children that share a DNM at the site. Reads with mapping quality <20 are filtered out, as they were not considered by our variant calling pipeline, and mismatched bases are shaded by quality score (more transparent = lower base quality). [file elife-46922-supp4.zip › supp_file_4/chr9_37,969,874_37,969,914.png]

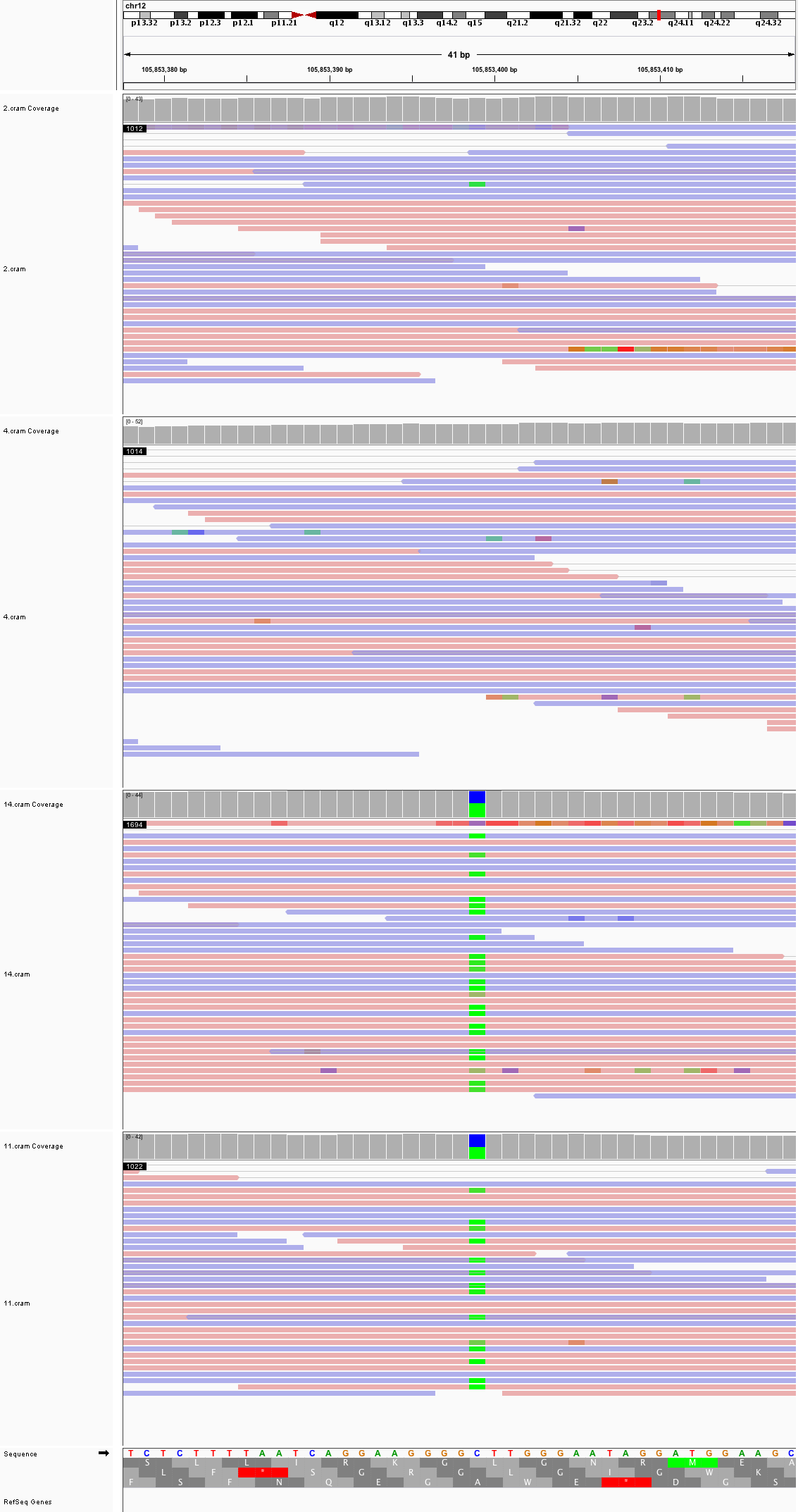

Supplement: Supplementary file 4. — All tracks below contain alignments from the third-generation children that share a DNM at the site. Reads with mapping quality <20 are filtered out, as they were not considered by our variant calling pipeline, and mismatched bases are shaded by quality score (more transparent = lower base quality). [file elife-46922-supp4.zip › supp_file_4/chr12_105,853,378_105,853,418.png]

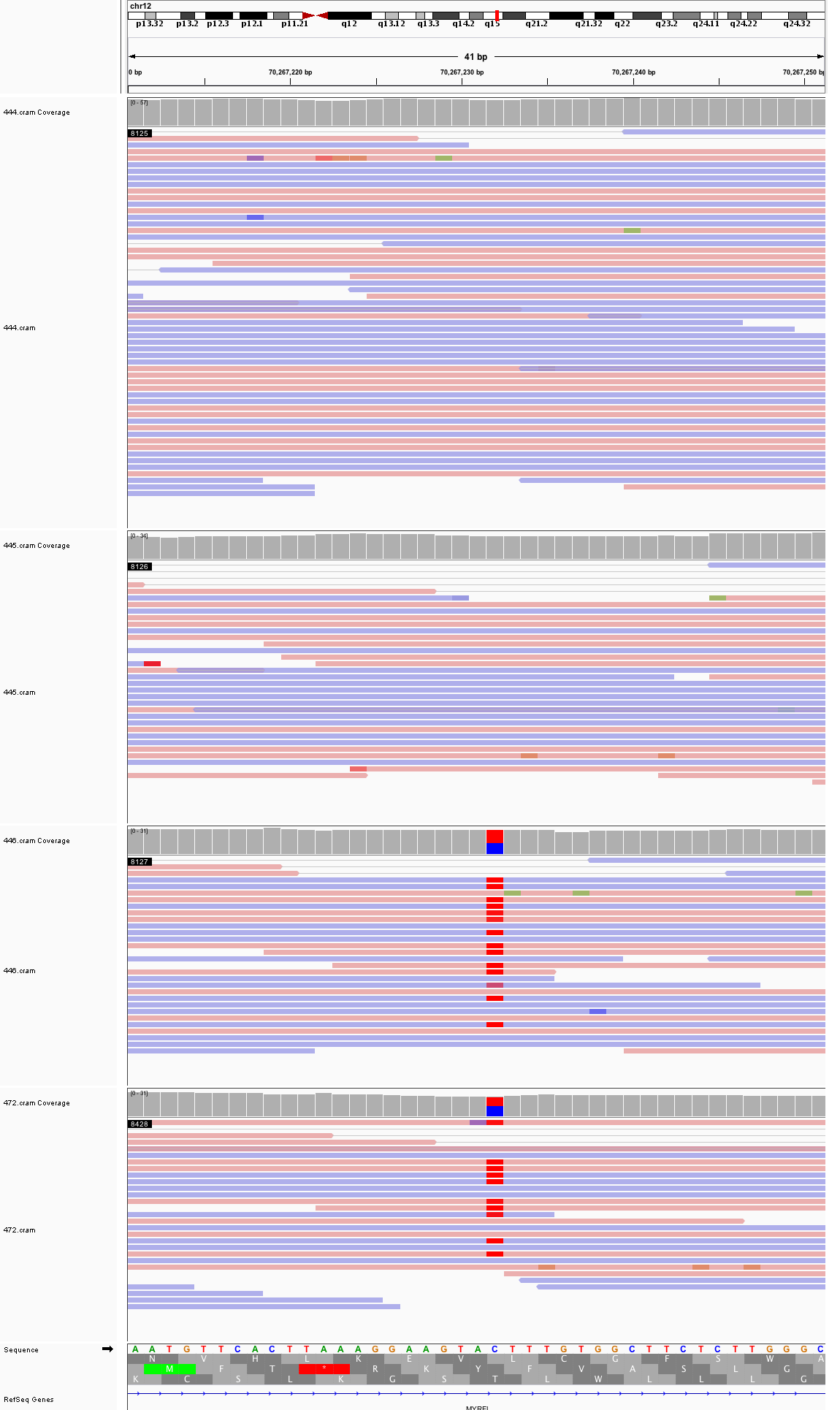

Supplement: Supplementary file 4. — All tracks below contain alignments from the third-generation children that share a DNM at the site. Reads with mapping quality <20 are filtered out, as they were not considered by our variant calling pipeline, and mismatched bases are shaded by quality score (more transparent = lower base quality). [file elife-46922-supp4.zip › supp_file_4/chr12_70,267,211_70,267,251.png]

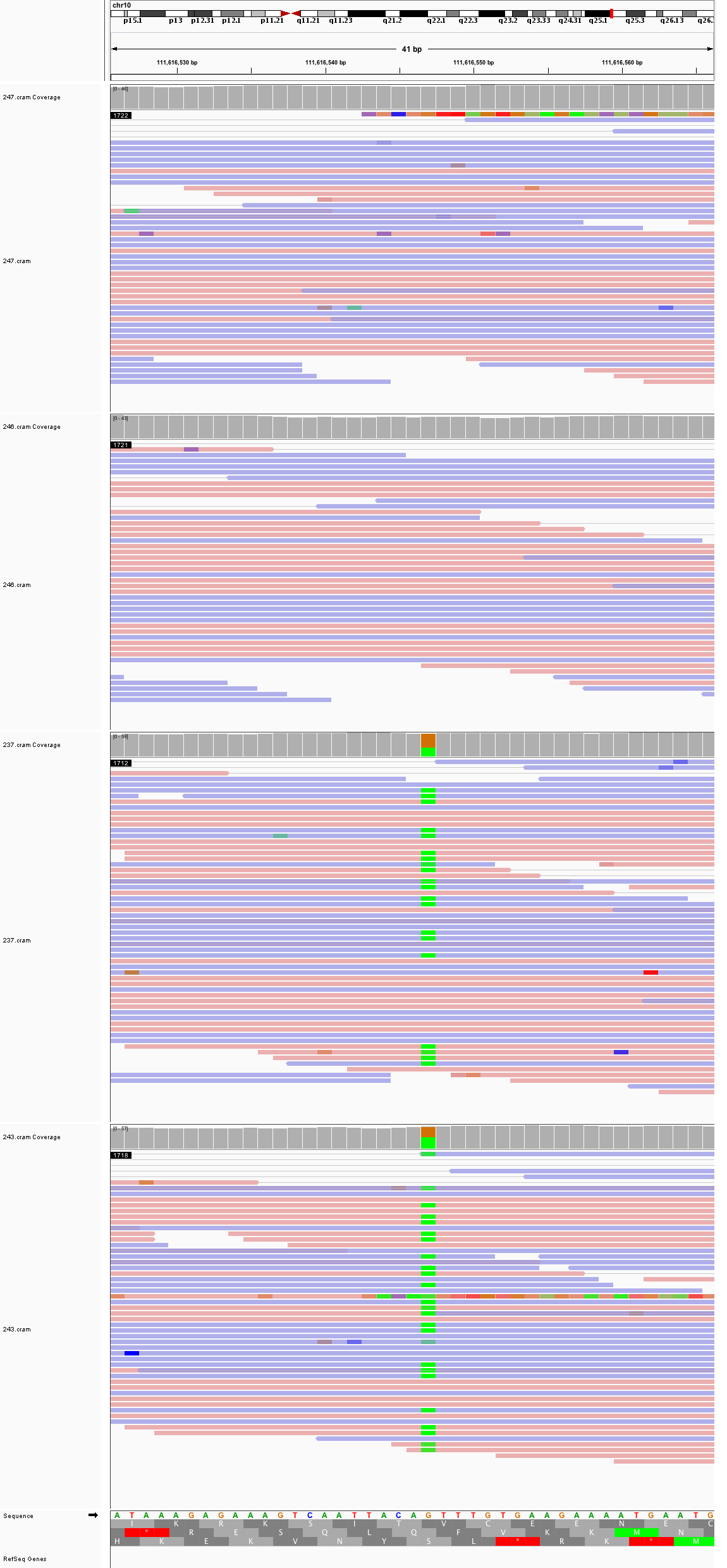

Supplement: Supplementary file 4. — All tracks below contain alignments from the third-generation children that share a DNM at the site. Reads with mapping quality <20 are filtered out, as they were not considered by our variant calling pipeline, and mismatched bases are shaded by quality score (more transparent = lower base quality). [file elife-46922-supp4.zip › supp_file_4/chr10_111,616,526_111,616,566.png]

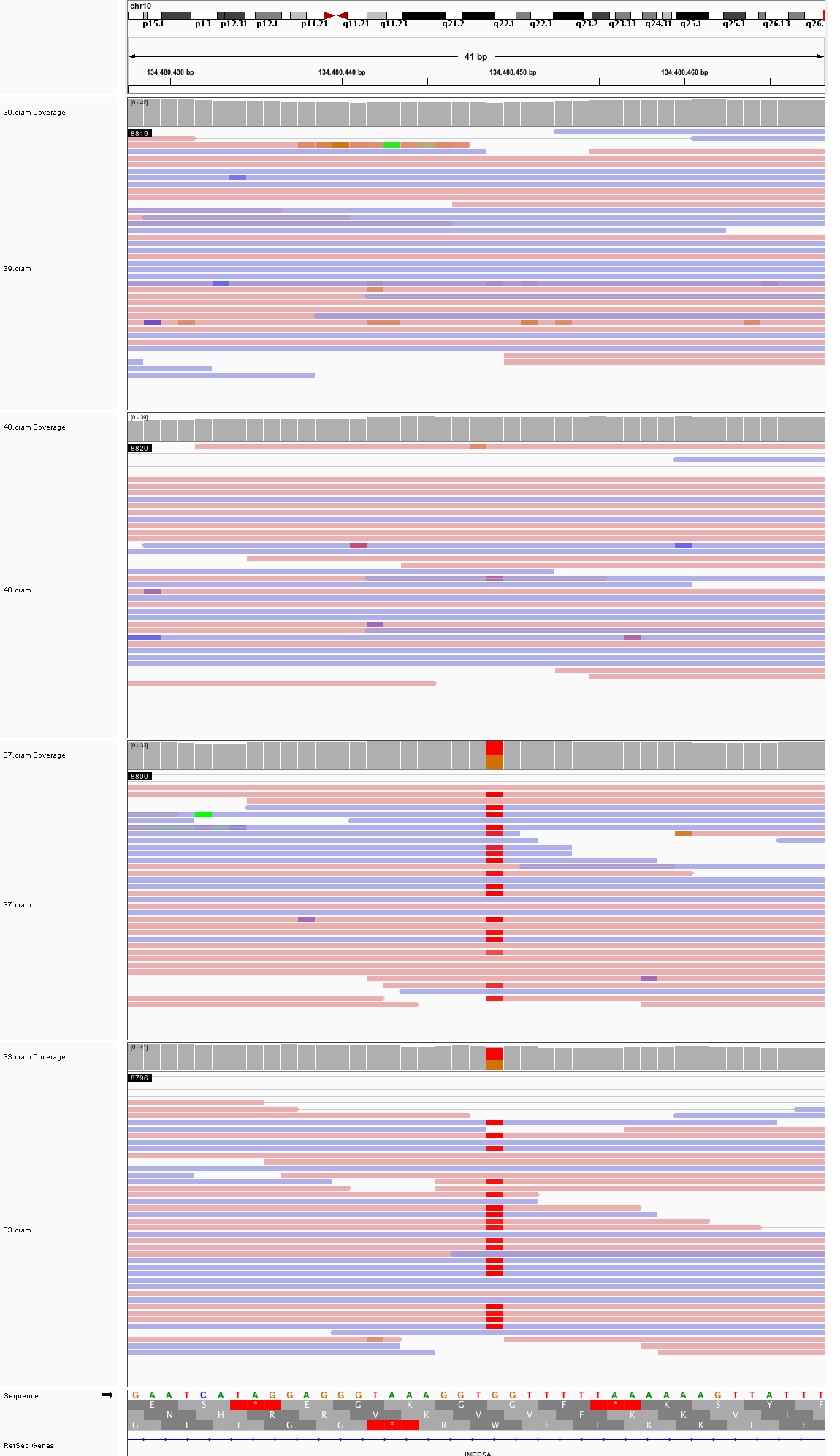

Supplement: Supplementary file 4. — All tracks below contain alignments from the third-generation children that share a DNM at the site. Reads with mapping quality <20 are filtered out, as they were not considered by our variant calling pipeline, and mismatched bases are shaded by quality score (more transparent = lower base quality). [file elife-46922-supp4.zip › supp_file_4/chr10_134,480,428_134,480,468.png]

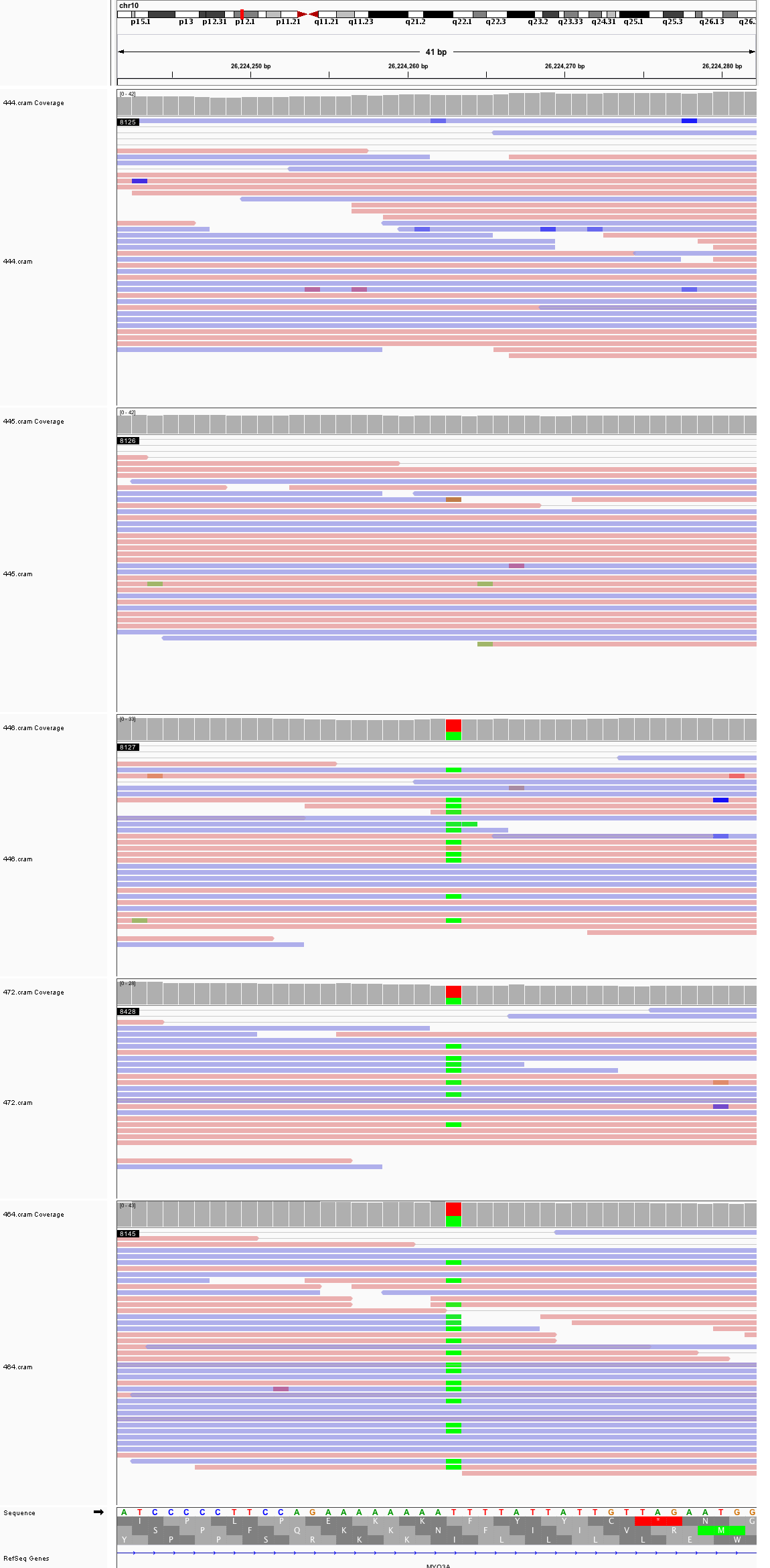

Supplement: Supplementary file 4. — All tracks below contain alignments from the third-generation children that share a DNM at the site. Reads with mapping quality <20 are filtered out, as they were not considered by our variant calling pipeline, and mismatched bases are shaded by quality score (more transparent = lower base quality). [file elife-46922-supp4.zip › supp_file_4/chr10_26,224,242_26,224,282.png]

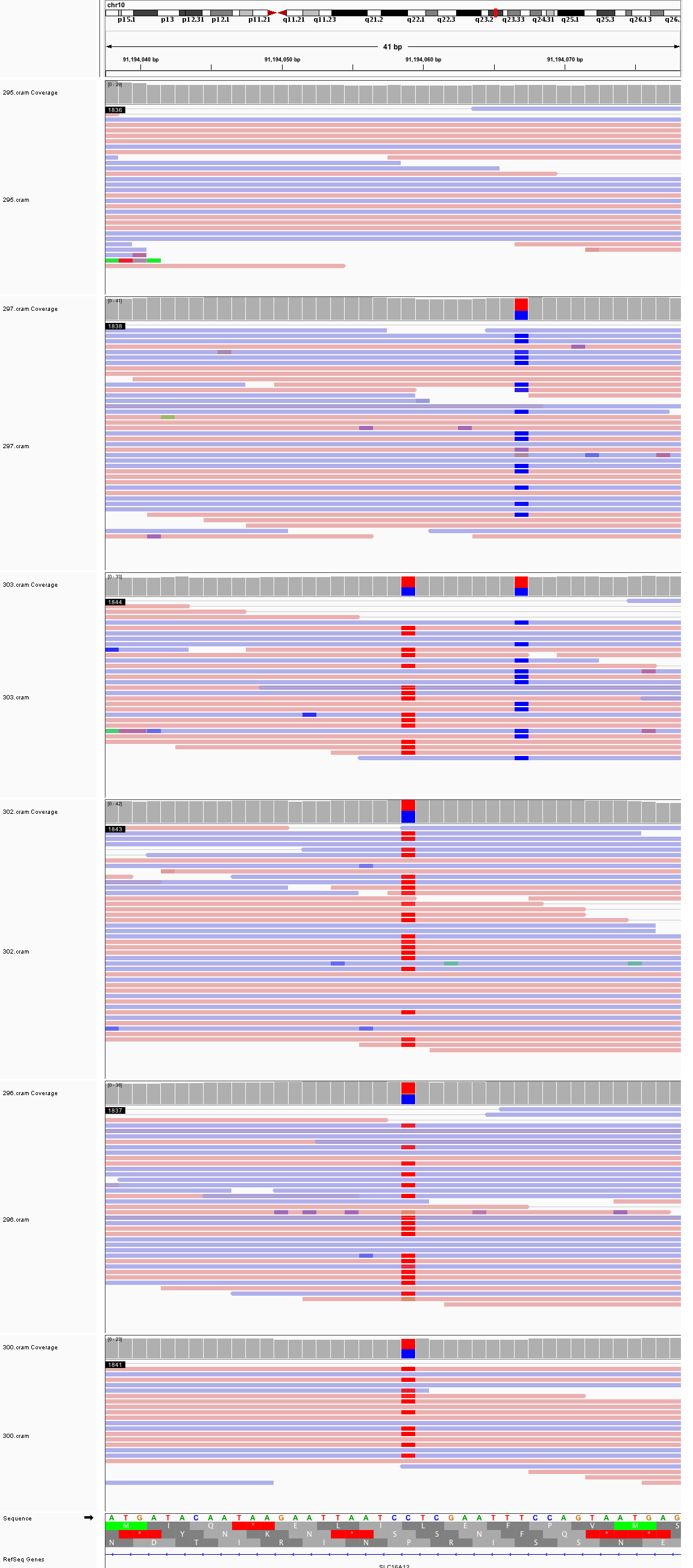

Supplement: Supplementary file 4. — All tracks below contain alignments from the third-generation children that share a DNM at the site. Reads with mapping quality <20 are filtered out, as they were not considered by our variant calling pipeline, and mismatched bases are shaded by quality score (more transparent = lower base quality). [file elife-46922-supp4.zip › supp_file_4/chr10_91,194,038_91,194,078.png]

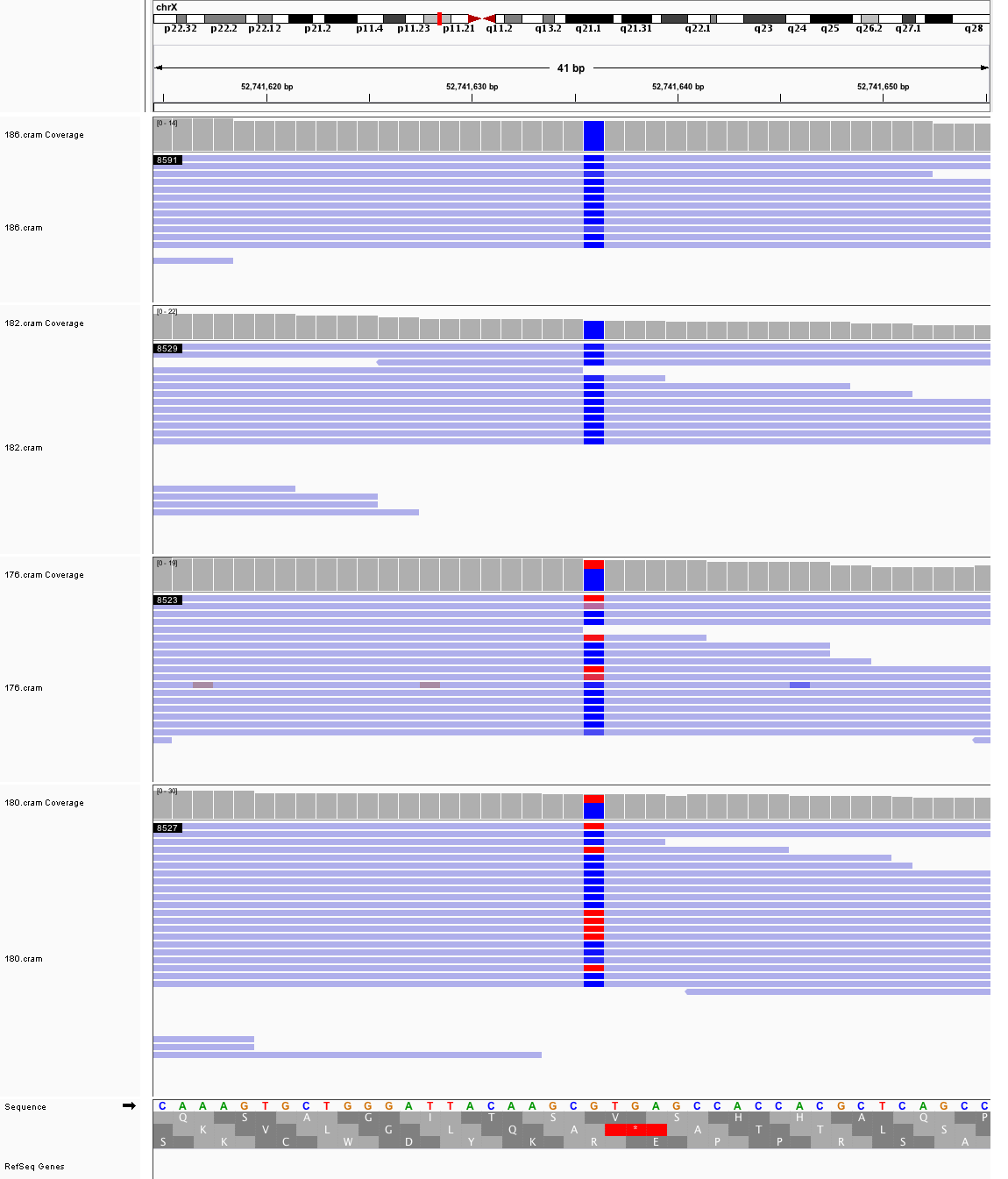

Supplement: Supplementary file 4. — All tracks below contain alignments from the third-generation children that share a DNM at the site. Reads with mapping quality <20 are filtered out, as they were not considered by our variant calling pipeline, and mismatched bases are shaded by quality score (more transparent = lower base quality). [file elife-46922-supp4.zip › supp_file_4/chrX_52,741,615_52,741,655.png]

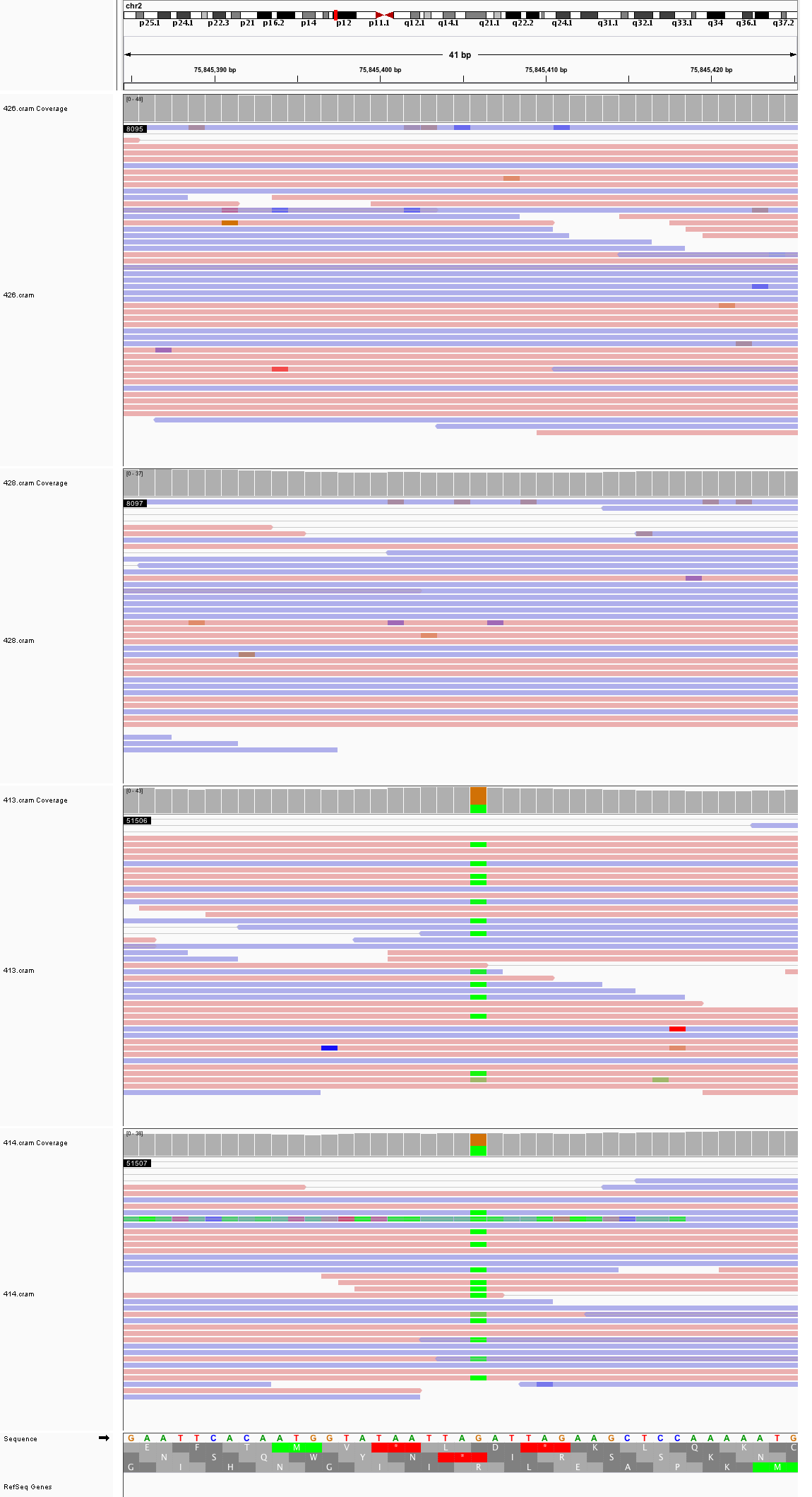

Supplement: Supplementary file 4. — All tracks below contain alignments from the third-generation children that share a DNM at the site. Reads with mapping quality <20 are filtered out, as they were not considered by our variant calling pipeline, and mismatched bases are shaded by quality score (more transparent = lower base quality). [file elife-46922-supp4.zip › supp_file_4/chr2_75,845,385_75,845,425.png]

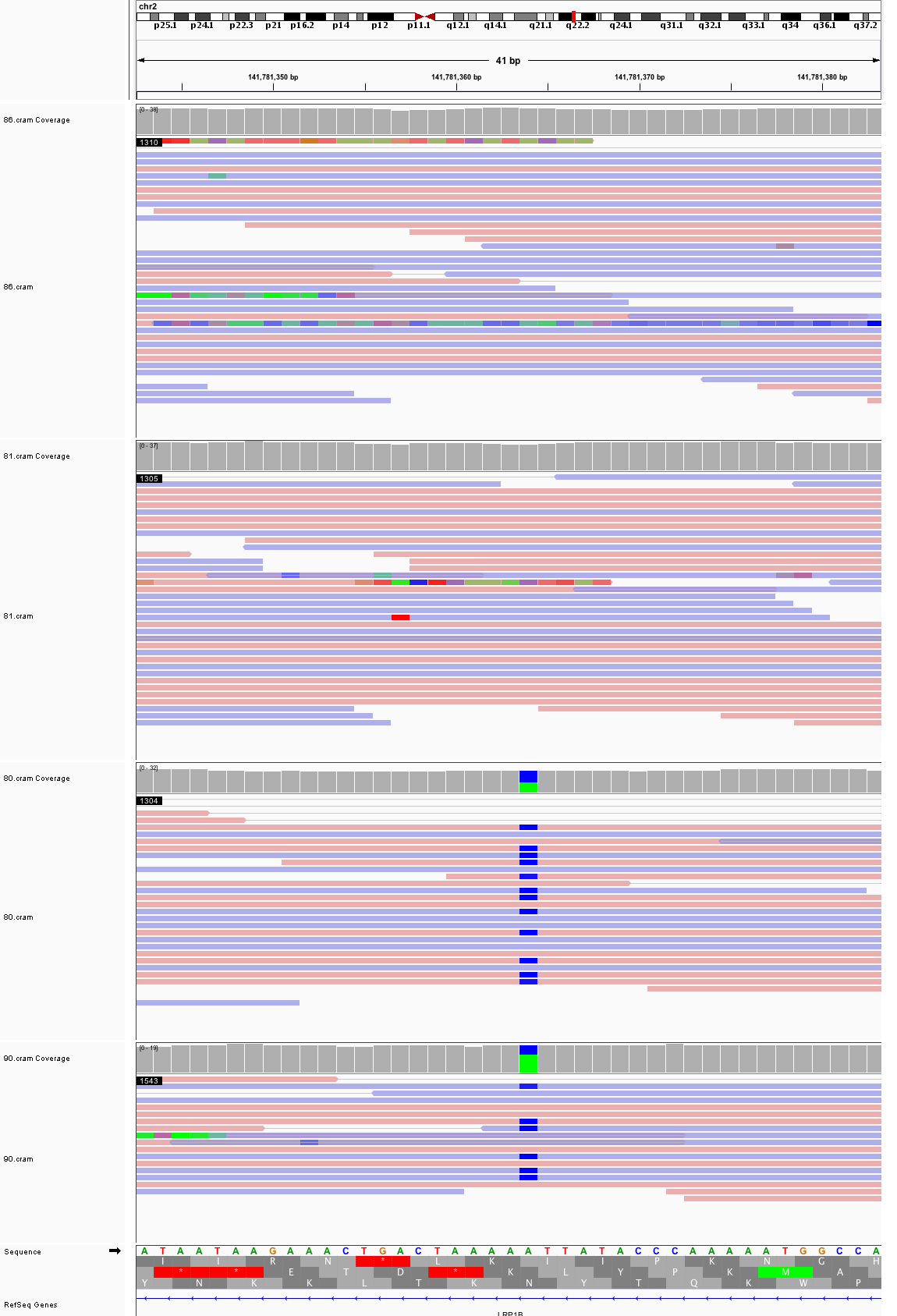

Supplement: Supplementary file 4. — All tracks below contain alignments from the third-generation children that share a DNM at the site. Reads with mapping quality <20 are filtered out, as they were not considered by our variant calling pipeline, and mismatched bases are shaded by quality score (more transparent = lower base quality). [file elife-46922-supp4.zip › supp_file_4/chr2_141,781,343_141,781,383.png]

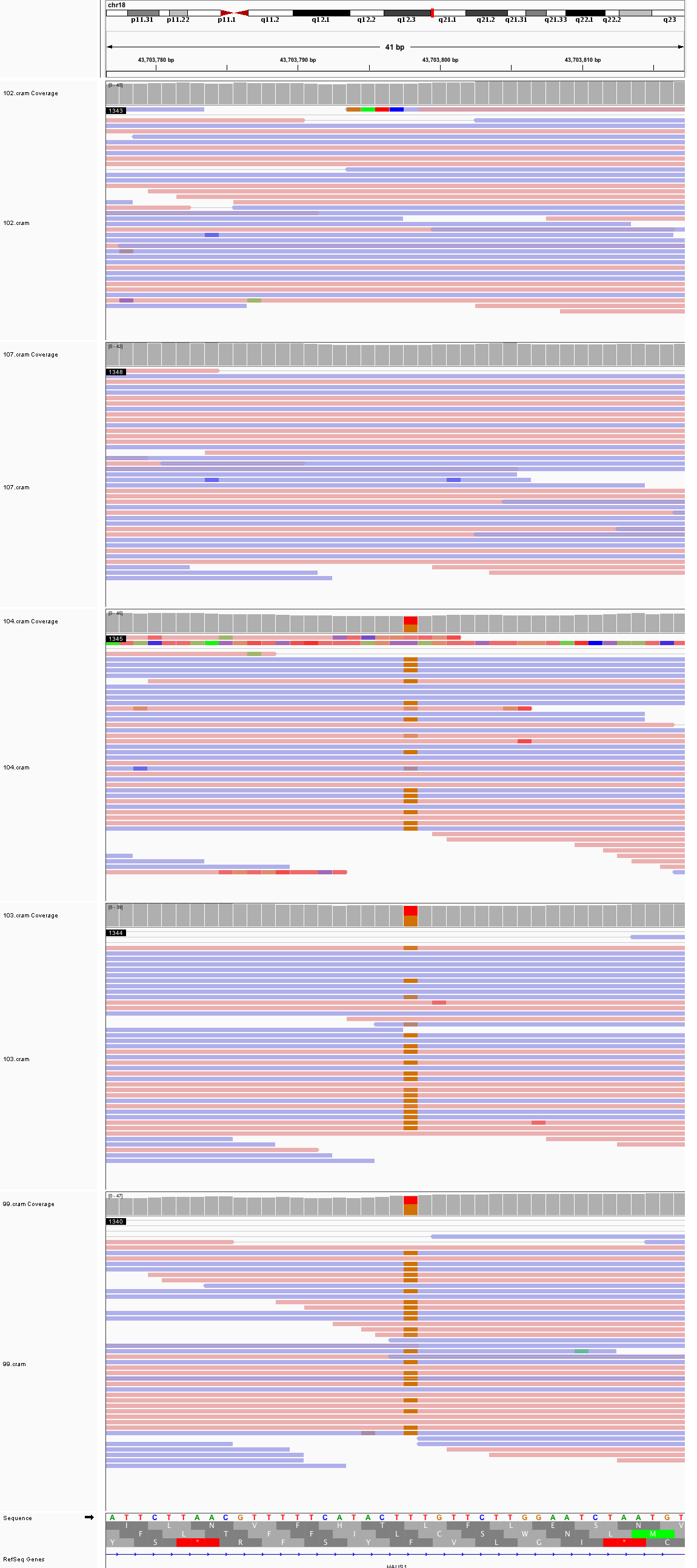

Supplement: Supplementary file 4. — All tracks below contain alignments from the third-generation children that share a DNM at the site. Reads with mapping quality <20 are filtered out, as they were not considered by our variant calling pipeline, and mismatched bases are shaded by quality score (more transparent = lower base quality). [file elife-46922-supp4.zip › supp_file_4/chr18_43,703,777_43,703,817.png]

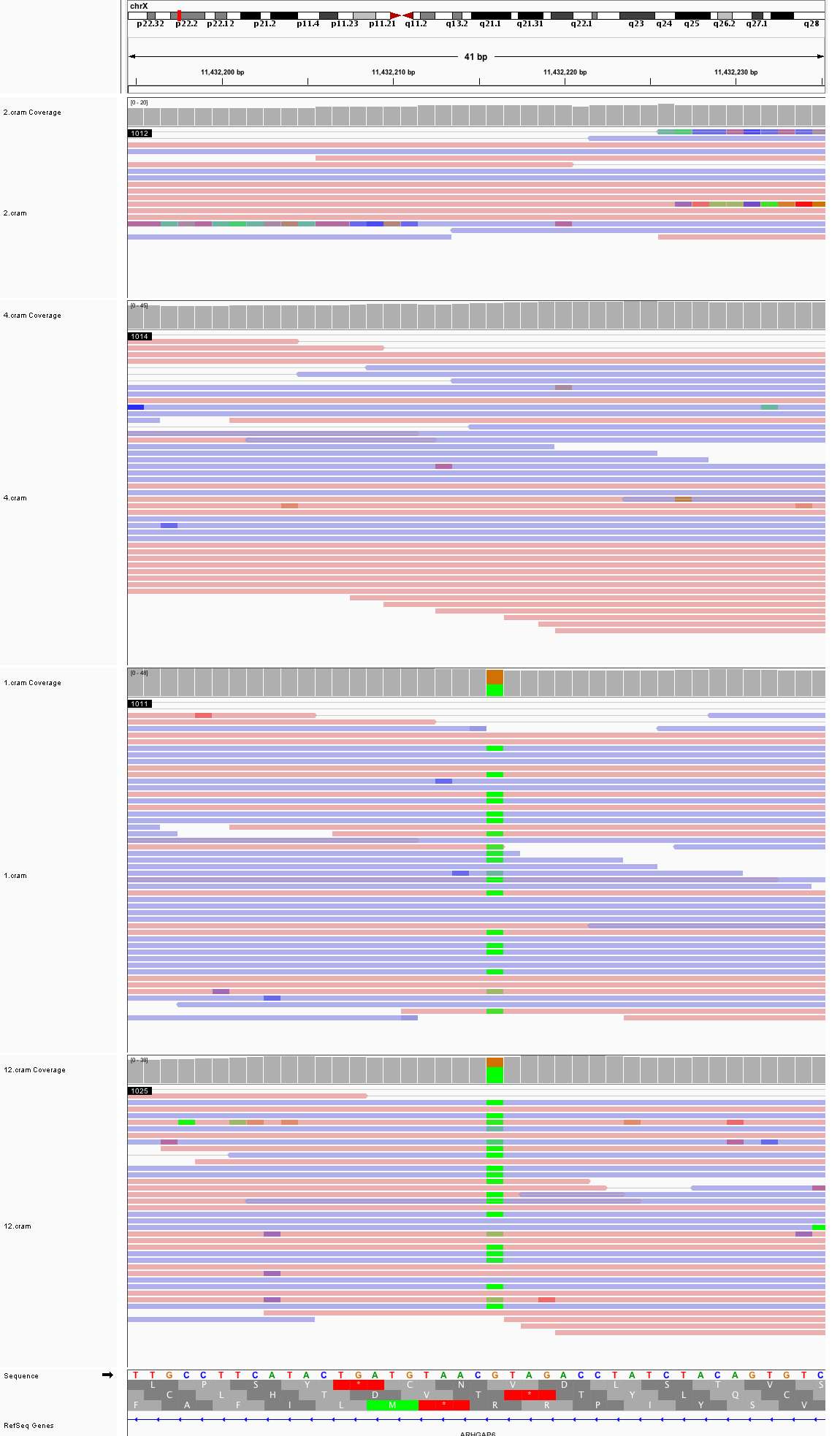

Supplement: Supplementary file 4. — All tracks below contain alignments from the third-generation children that share a DNM at the site. Reads with mapping quality <20 are filtered out, as they were not considered by our variant calling pipeline, and mismatched bases are shaded by quality score (more transparent = lower base quality). [file elife-46922-supp4.zip › supp_file_4/chrX_11,432,195_11,432,235.png]

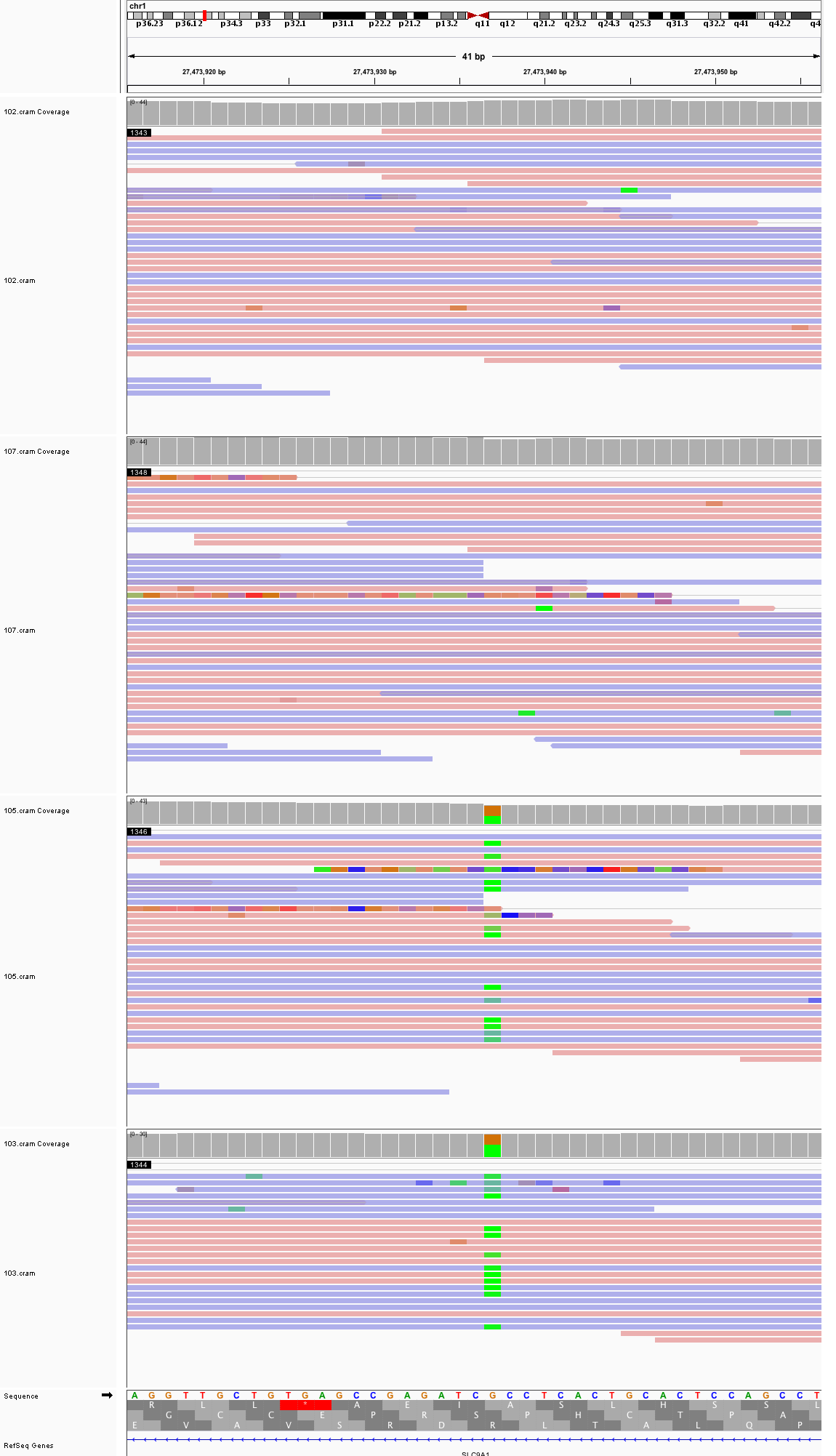

Supplement: Supplementary file 4. — All tracks below contain alignments from the third-generation children that share a DNM at the site. Reads with mapping quality <20 are filtered out, as they were not considered by our variant calling pipeline, and mismatched bases are shaded by quality score (more transparent = lower base quality). [file elife-46922-supp4.zip › supp_file_4/chr1_27,473,916_27,473,956.png]

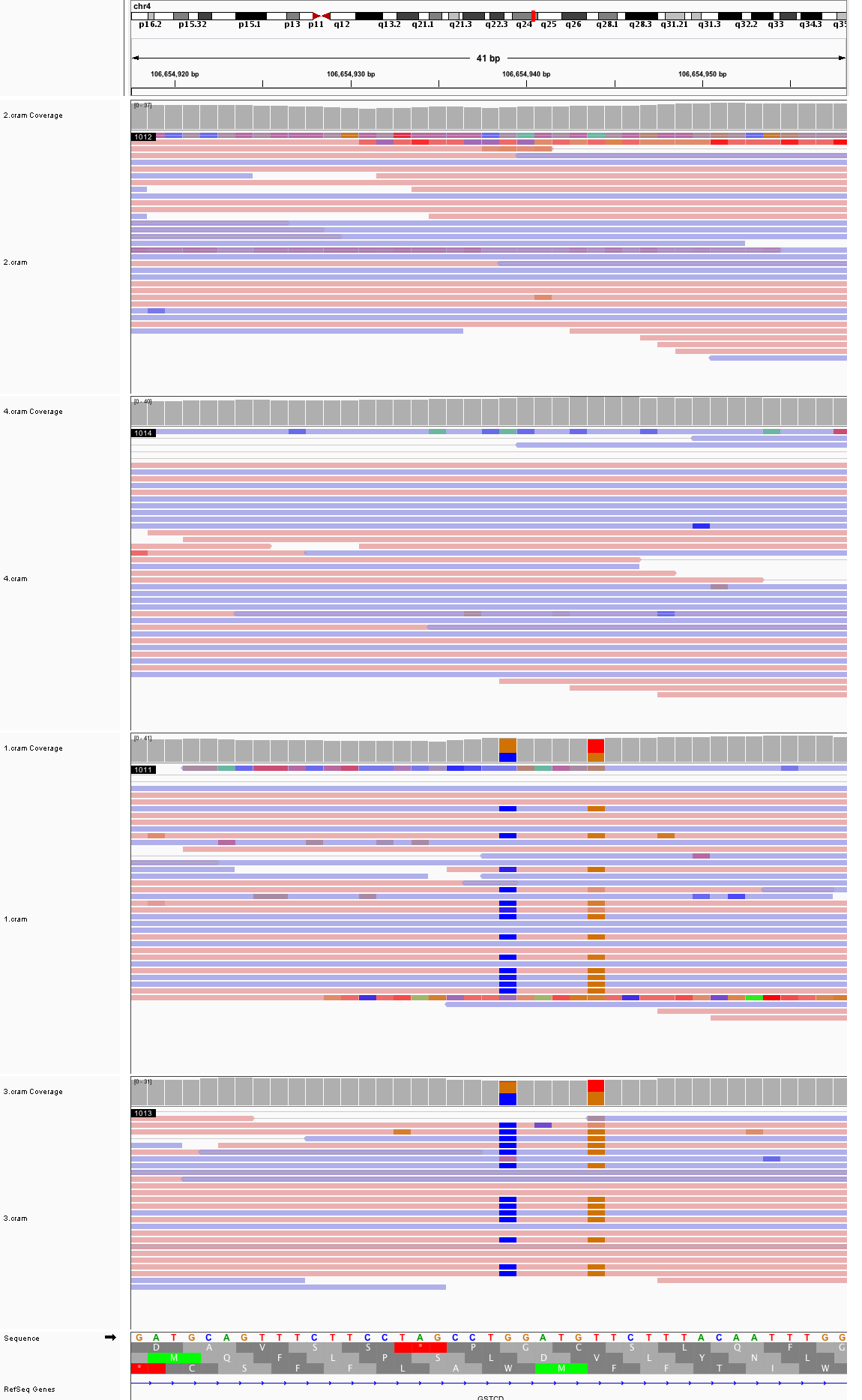

Supplement: Supplementary file 4. — All tracks below contain alignments from the third-generation children that share a DNM at the site. Reads with mapping quality <20 are filtered out, as they were not considered by our variant calling pipeline, and mismatched bases are shaded by quality score (more transparent = lower base quality). [file elife-46922-supp4.zip › supp_file_4/chr4_106,654,918_106,654,958.png]

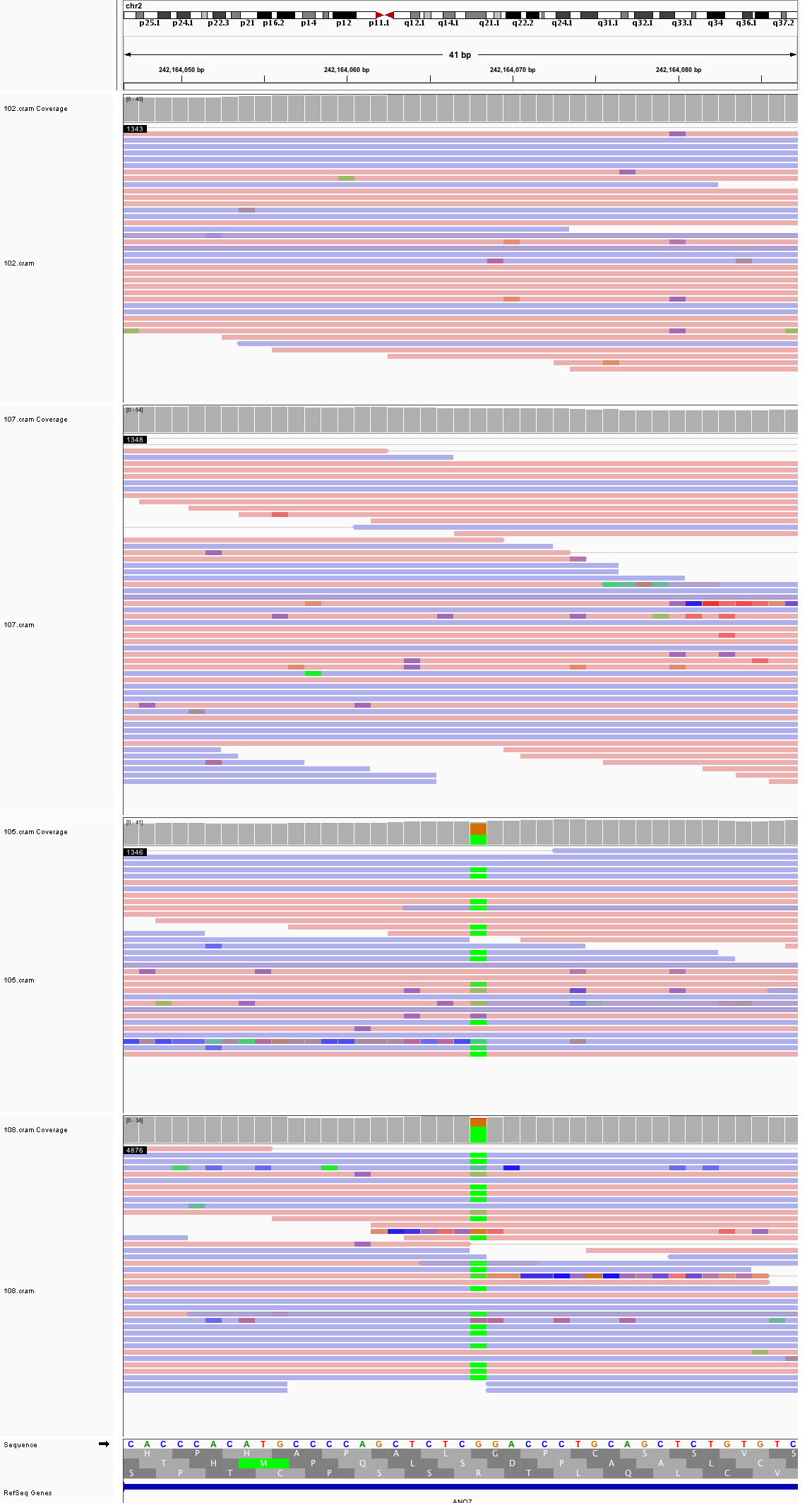

Supplement: Supplementary file 4. — All tracks below contain alignments from the third-generation children that share a DNM at the site. Reads with mapping quality <20 are filtered out, as they were not considered by our variant calling pipeline, and mismatched bases are shaded by quality score (more transparent = lower base quality). [file elife-46922-supp4.zip › supp_file_4/chr2_242,164,047_242,164,087.png]

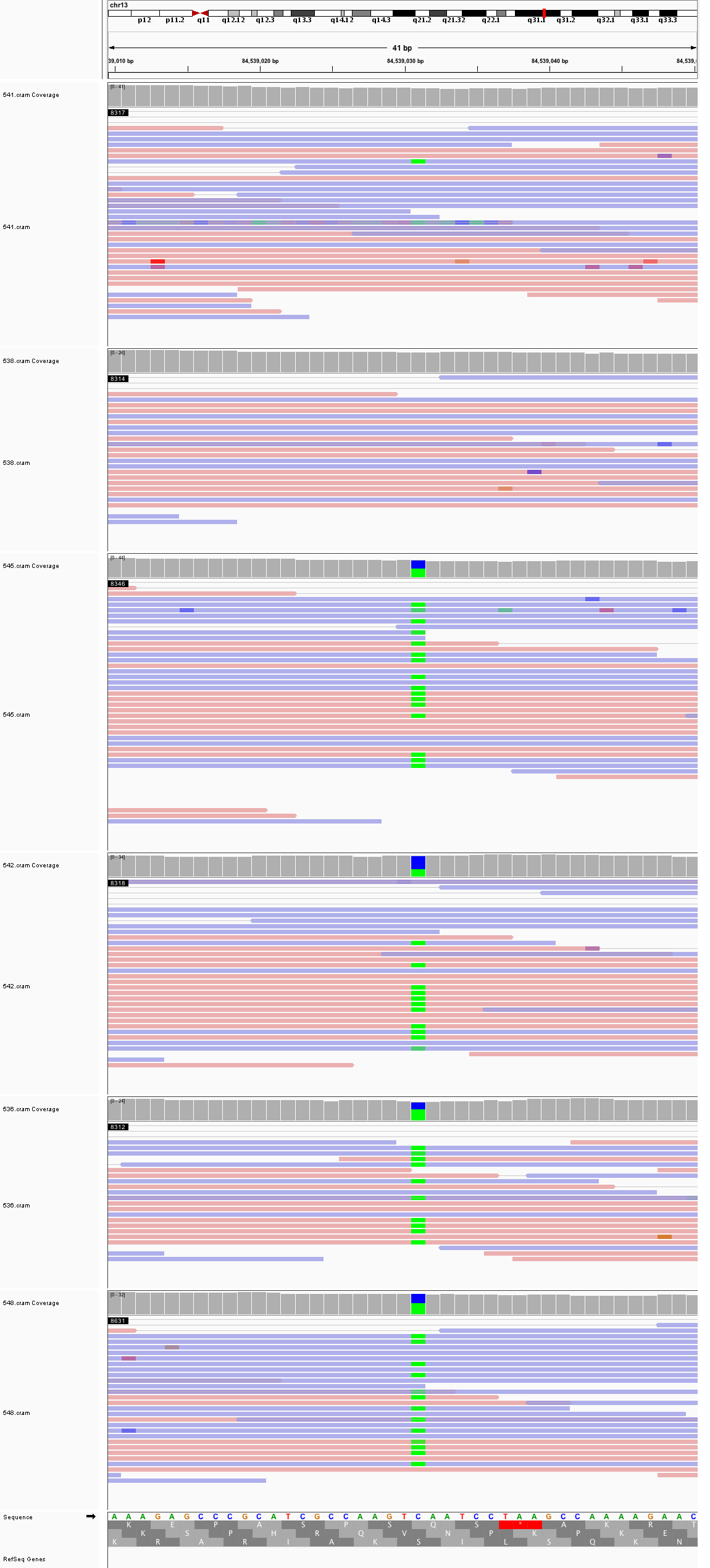

Supplement: Supplementary file 4. — All tracks below contain alignments from the third-generation children that share a DNM at the site. Reads with mapping quality <20 are filtered out, as they were not considered by our variant calling pipeline, and mismatched bases are shaded by quality score (more transparent = lower base quality). [file elife-46922-supp4.zip › supp_file_4/chr13_84,539,010_84,539,050.png]

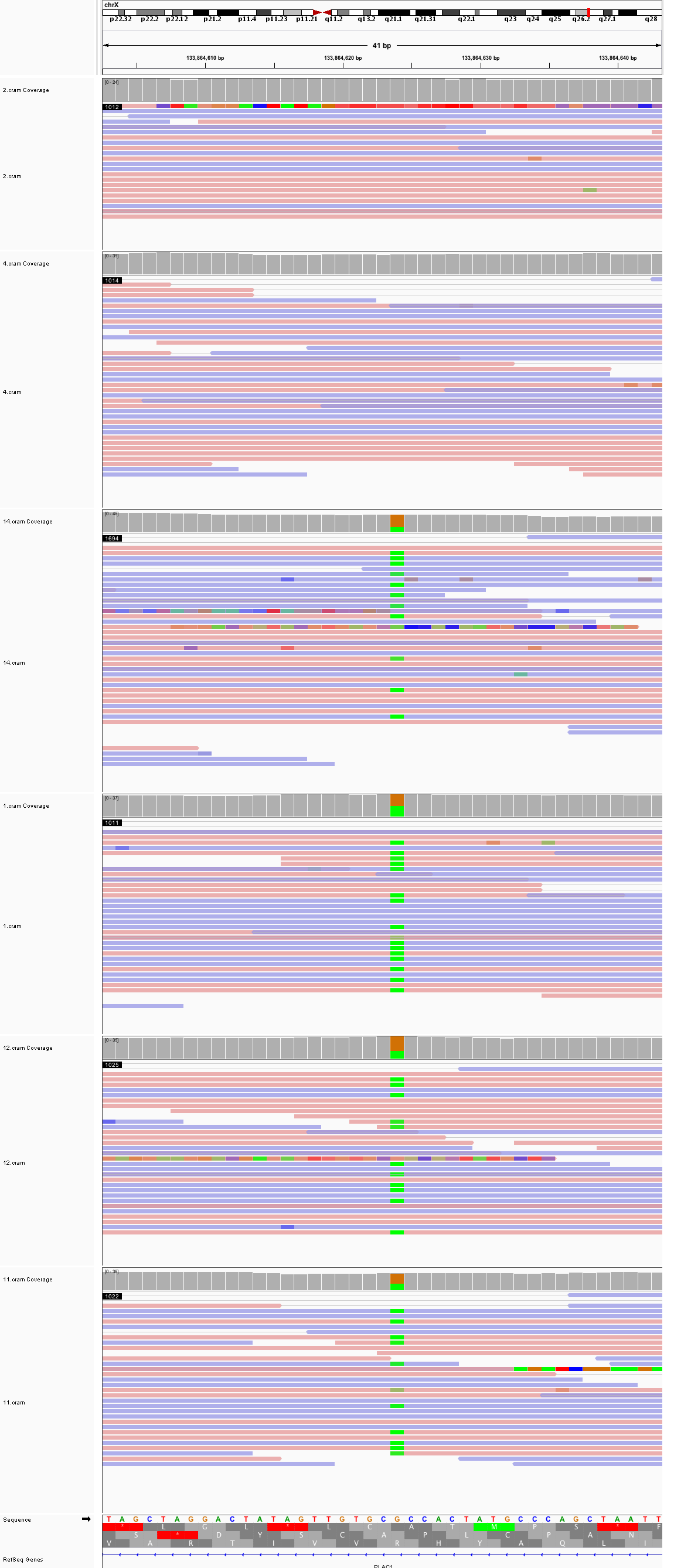

Supplement: Supplementary file 4. — All tracks below contain alignments from the third-generation children that share a DNM at the site. Reads with mapping quality <20 are filtered out, as they were not considered by our variant calling pipeline, and mismatched bases are shaded by quality score (more transparent = lower base quality). [file elife-46922-supp4.zip › supp_file_4/chrX_133,864,603_133,864,643.png]

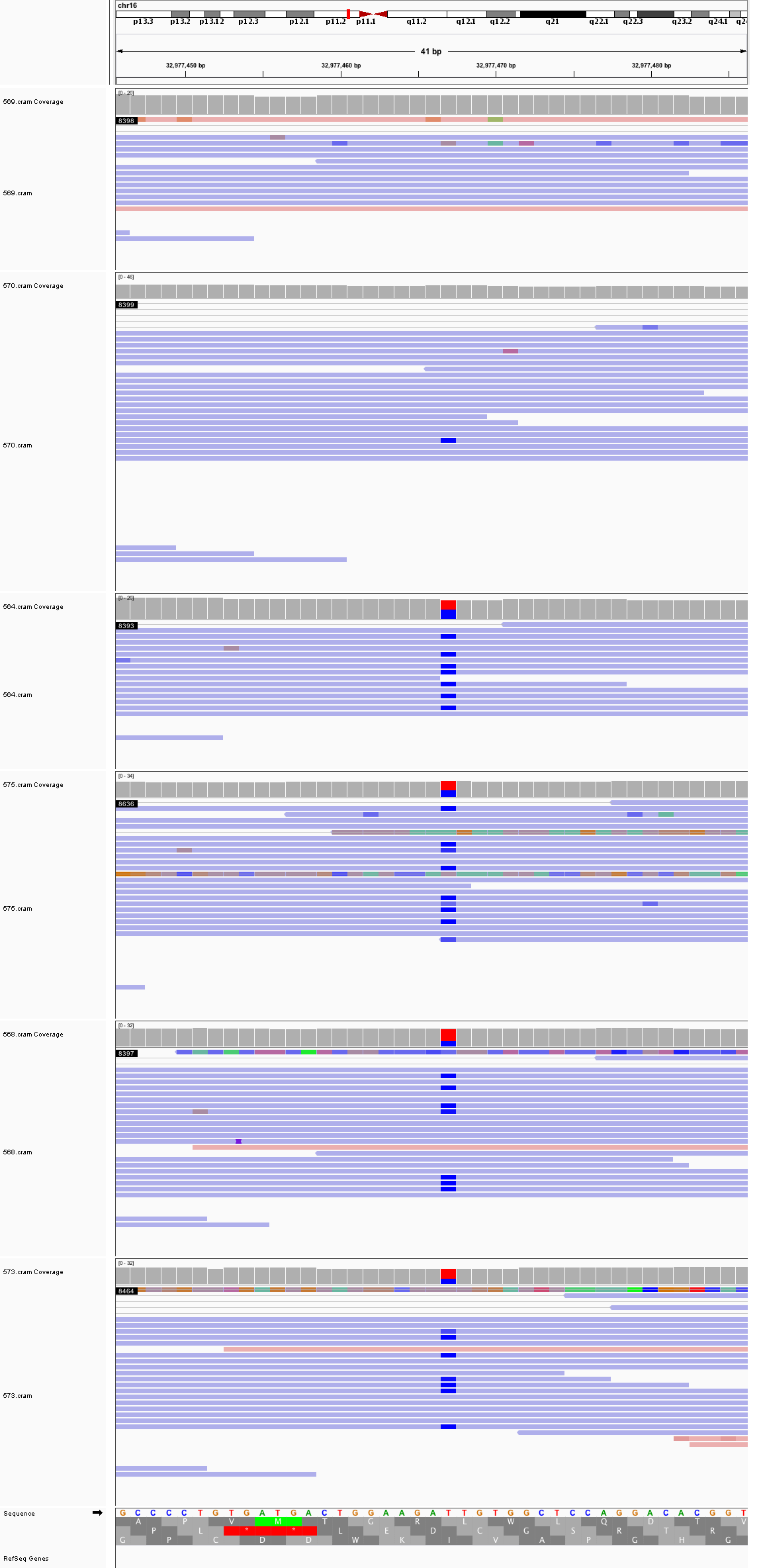

Supplement: Supplementary file 4. — All tracks below contain alignments from the third-generation children that share a DNM at the site. Reads with mapping quality <20 are filtered out, as they were not considered by our variant calling pipeline, and mismatched bases are shaded by quality score (more transparent = lower base quality). [file elife-46922-supp4.zip › supp_file_4/chr16_32,977,446_32,977,486.png]

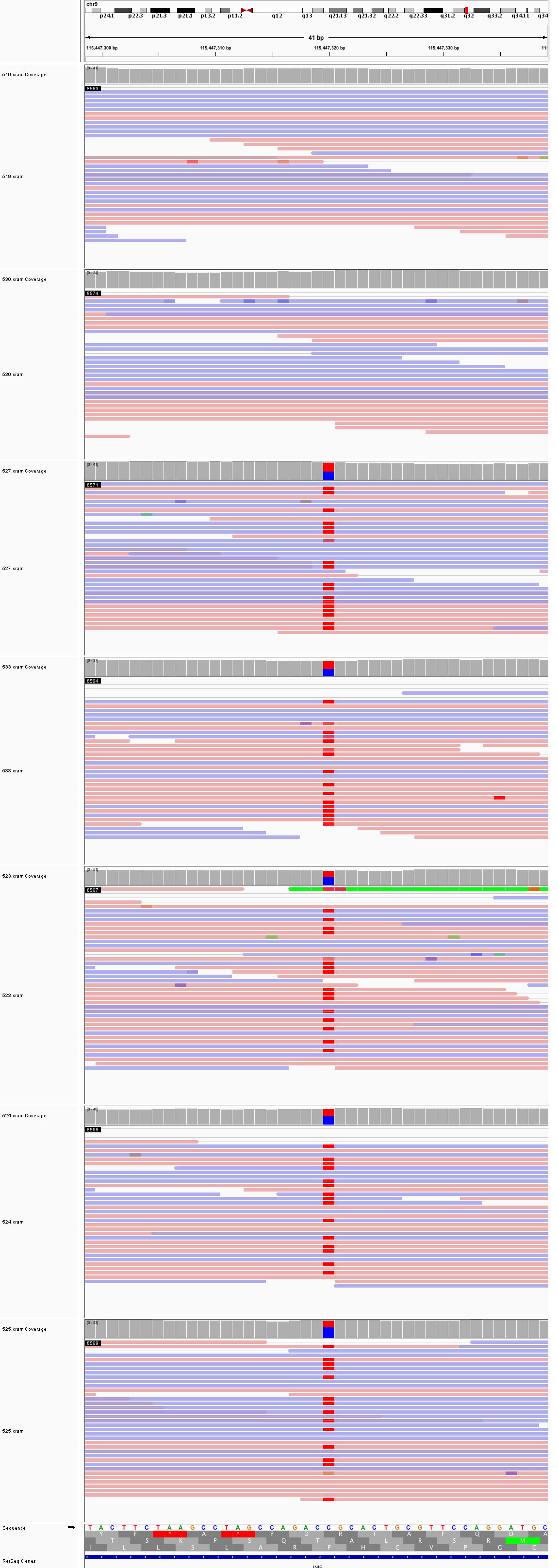

Supplement: Supplementary file 4. — All tracks below contain alignments from the third-generation children that share a DNM at the site. Reads with mapping quality <20 are filtered out, as they were not considered by our variant calling pipeline, and mismatched bases are shaded by quality score (more transparent = lower base quality). [file elife-46922-supp4.zip › supp_file_4/chr9_115,447,299_115,447,339.png]

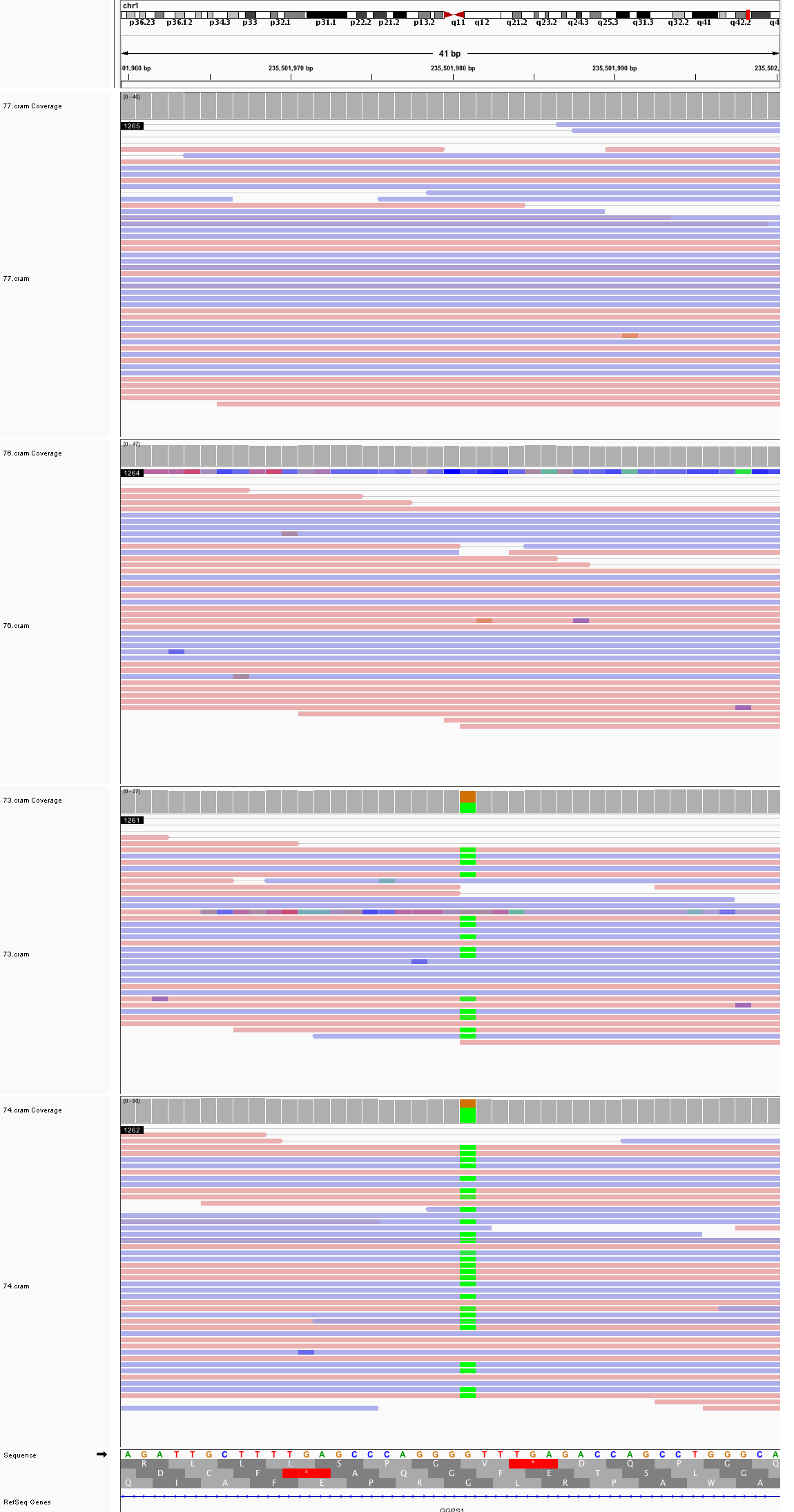

Supplement: Supplementary file 4. — All tracks below contain alignments from the third-generation children that share a DNM at the site. Reads with mapping quality <20 are filtered out, as they were not considered by our variant calling pipeline, and mismatched bases are shaded by quality score (more transparent = lower base quality). [file elife-46922-supp4.zip › supp_file_4/chr1_235,501,960_235,502,000.png]

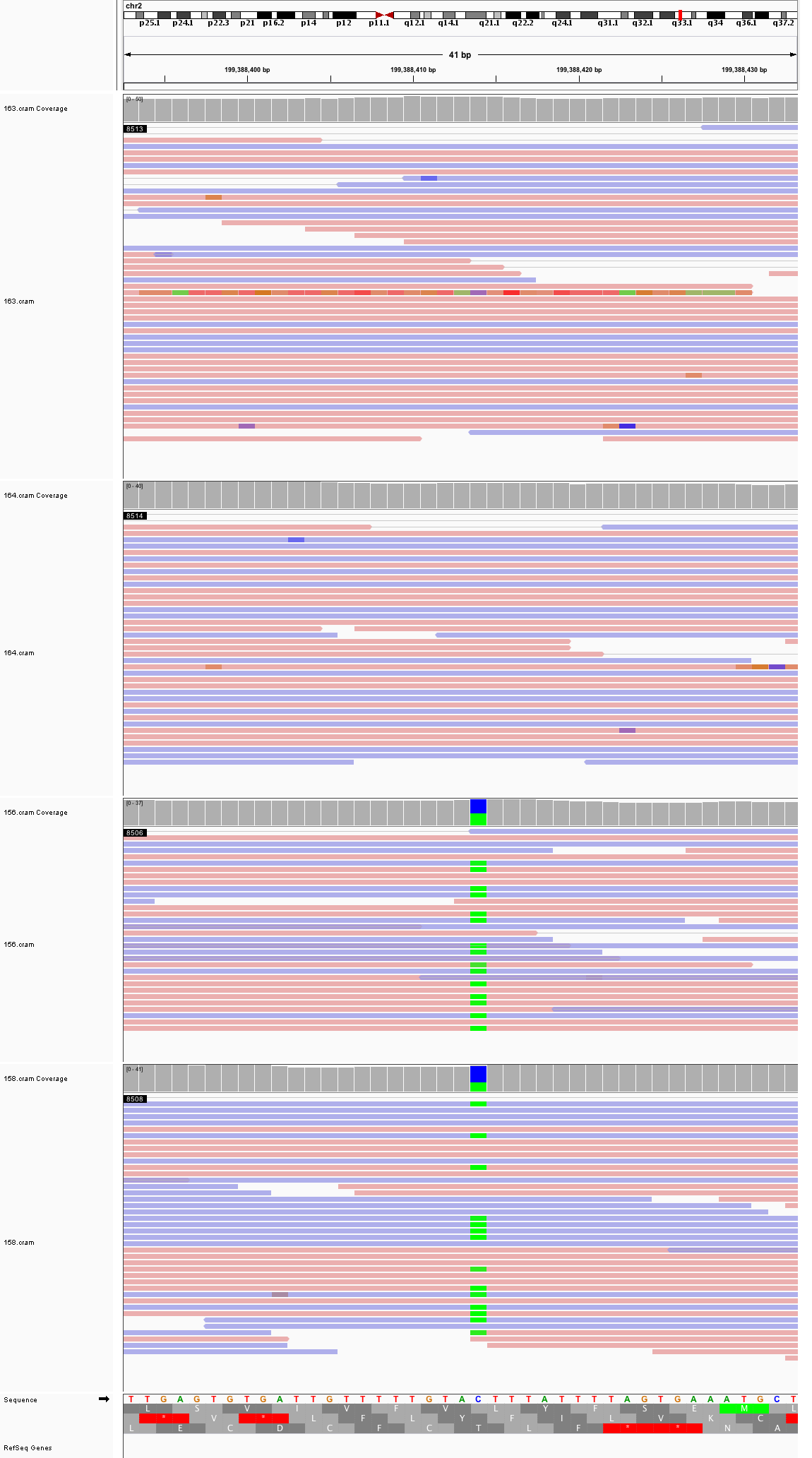

Supplement: Supplementary file 4. — All tracks below contain alignments from the third-generation children that share a DNM at the site. Reads with mapping quality <20 are filtered out, as they were not considered by our variant calling pipeline, and mismatched bases are shaded by quality score (more transparent = lower base quality). [file elife-46922-supp4.zip › supp_file_4/chr2_199,388,393_199,388,433.png]

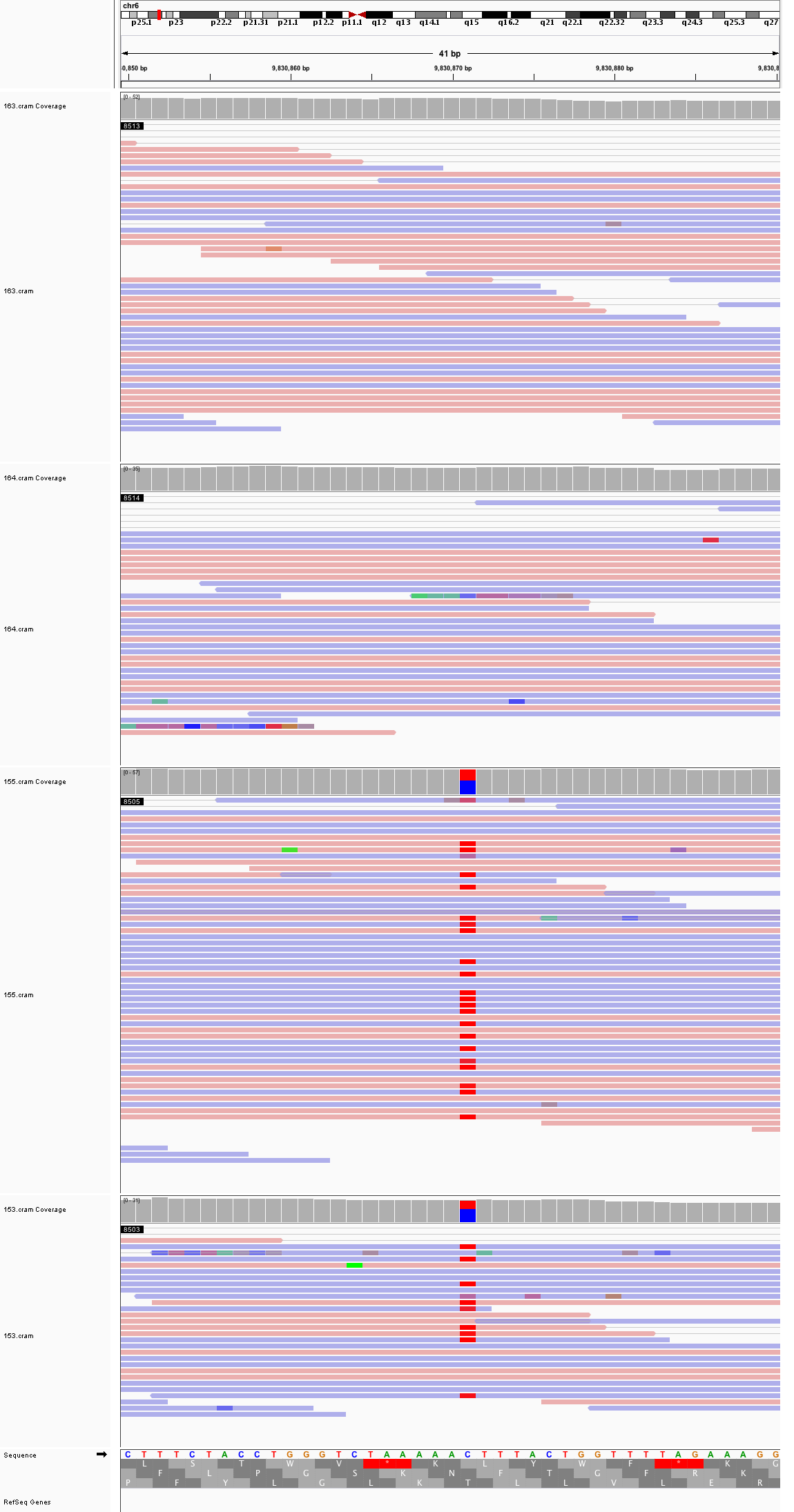

Supplement: Supplementary file 4. — All tracks below contain alignments from the third-generation children that share a DNM at the site. Reads with mapping quality <20 are filtered out, as they were not considered by our variant calling pipeline, and mismatched bases are shaded by quality score (more transparent = lower base quality). [file elife-46922-supp4.zip › supp_file_4/chr6_9,830,850_9,830,890.png]

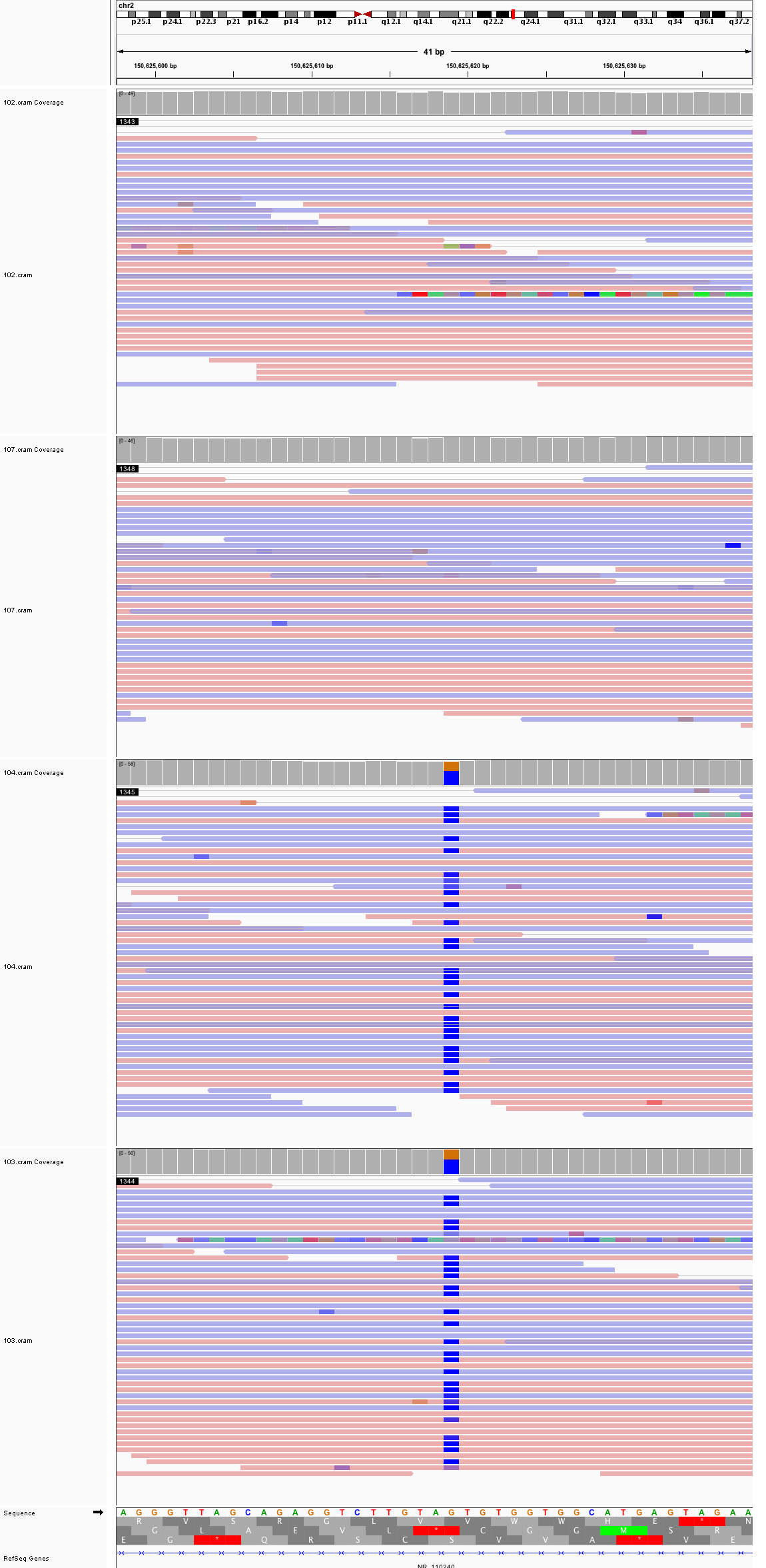

Supplement: Supplementary file 4. — All tracks below contain alignments from the third-generation children that share a DNM at the site. Reads with mapping quality <20 are filtered out, as they were not considered by our variant calling pipeline, and mismatched bases are shaded by quality score (more transparent = lower base quality). [file elife-46922-supp4.zip › supp_file_4/chr2_150,625,598_150,625,638.png]

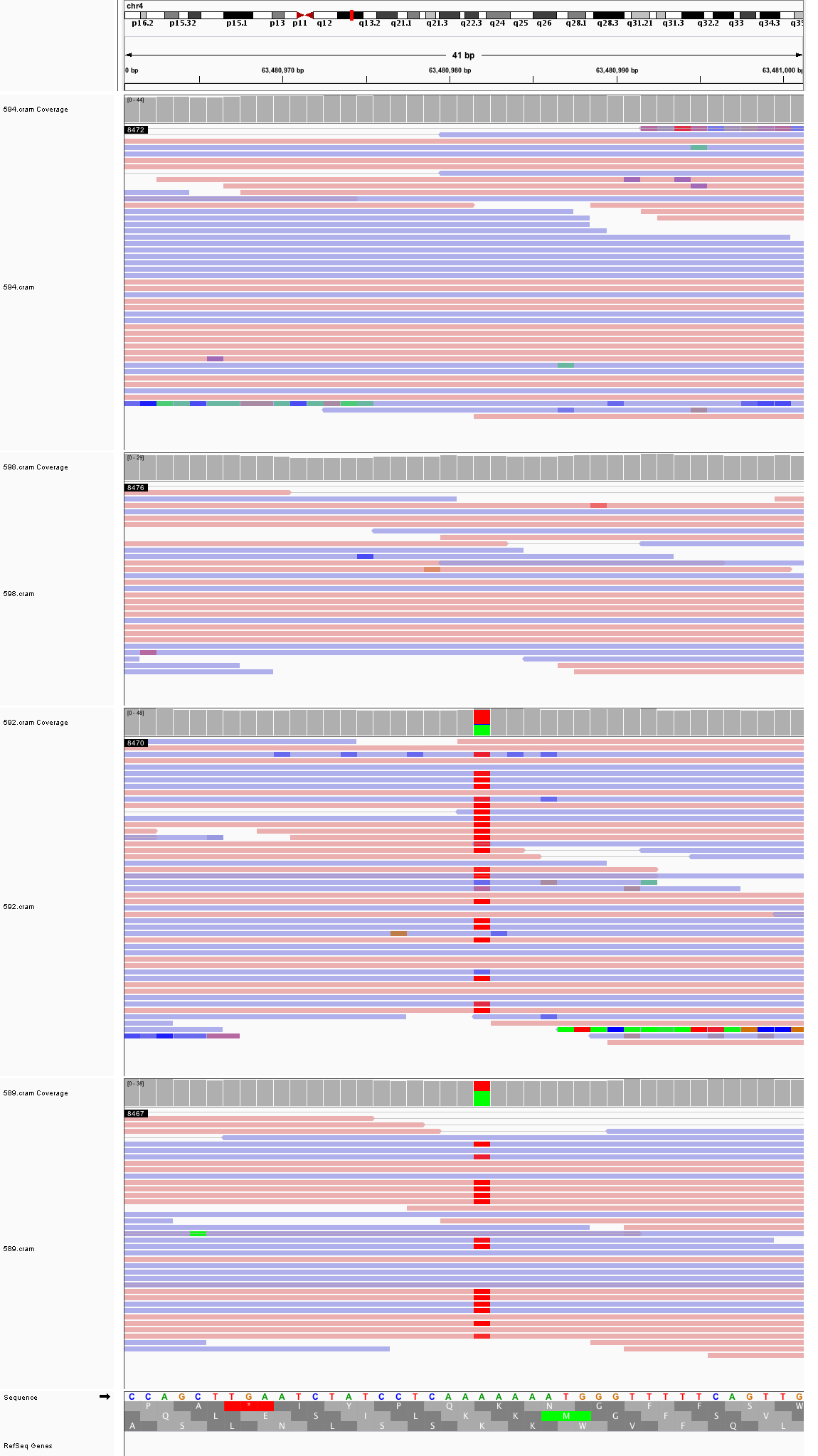

Supplement: Supplementary file 4. — All tracks below contain alignments from the third-generation children that share a DNM at the site. Reads with mapping quality <20 are filtered out, as they were not considered by our variant calling pipeline, and mismatched bases are shaded by quality score (more transparent = lower base quality). [file elife-46922-supp4.zip › supp_file_4/chr4_63,480,961_63,481,001.png]

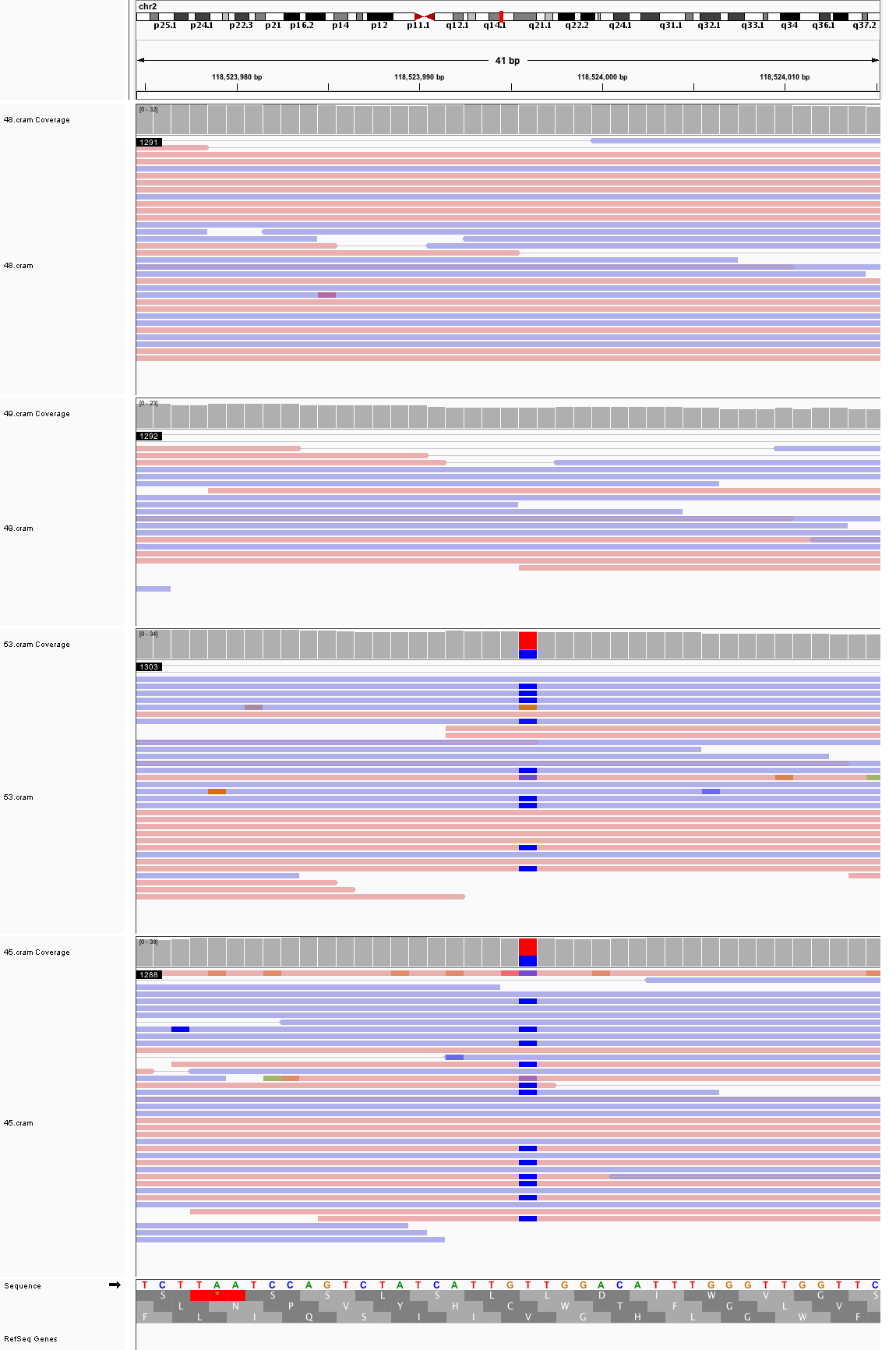

Supplement: Supplementary file 4. — All tracks below contain alignments from the third-generation children that share a DNM at the site. Reads with mapping quality <20 are filtered out, as they were not considered by our variant calling pipeline, and mismatched bases are shaded by quality score (more transparent = lower base quality). [file elife-46922-supp4.zip › supp_file_4/chr2_118,523,975_118,524,015.png]

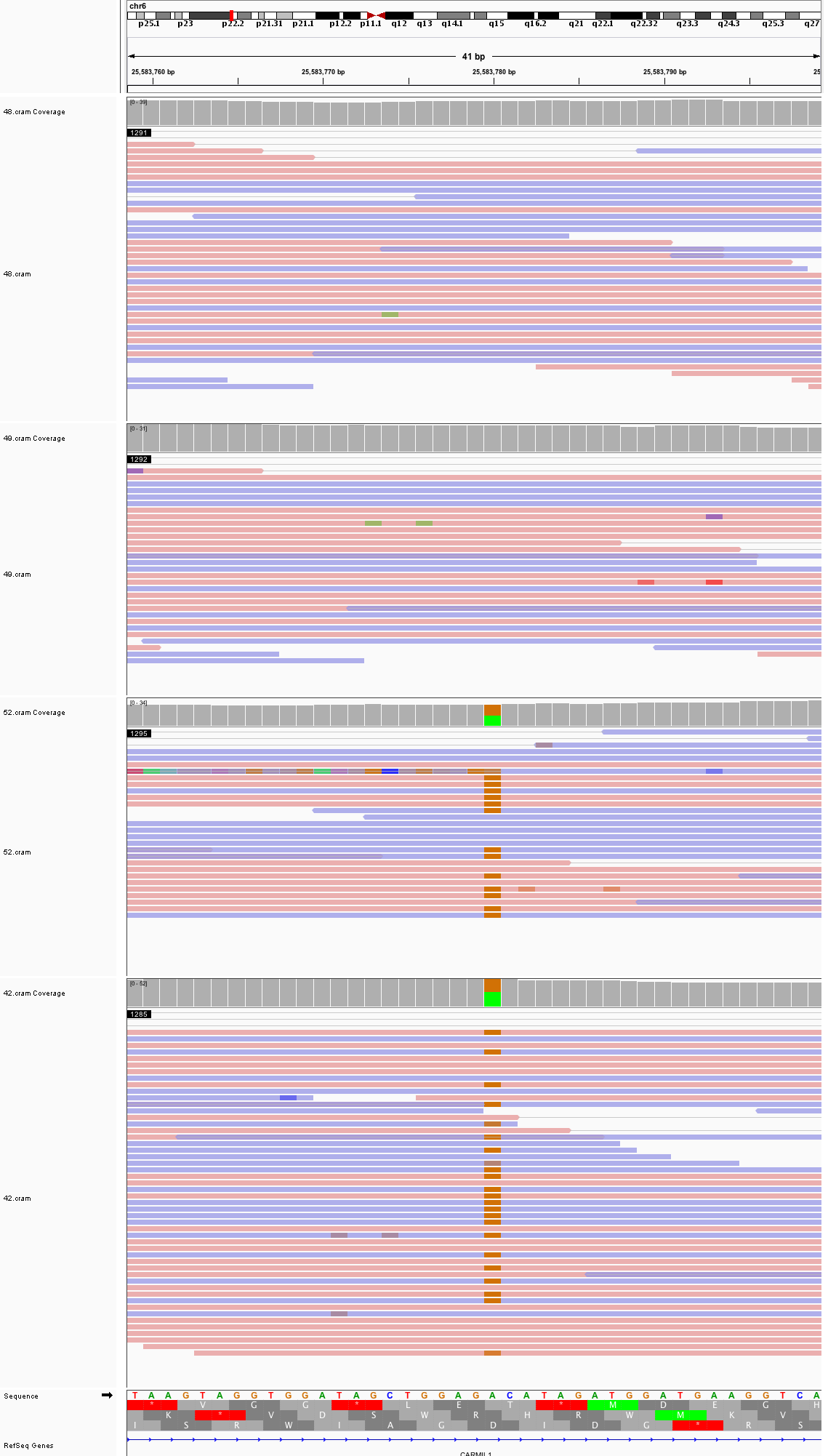

Supplement: Supplementary file 4. — All tracks below contain alignments from the third-generation children that share a DNM at the site. Reads with mapping quality <20 are filtered out, as they were not considered by our variant calling pipeline, and mismatched bases are shaded by quality score (more transparent = lower base quality). [file elife-46922-supp4.zip › supp_file_4/chr6_25,583,759_25,583,799.png]

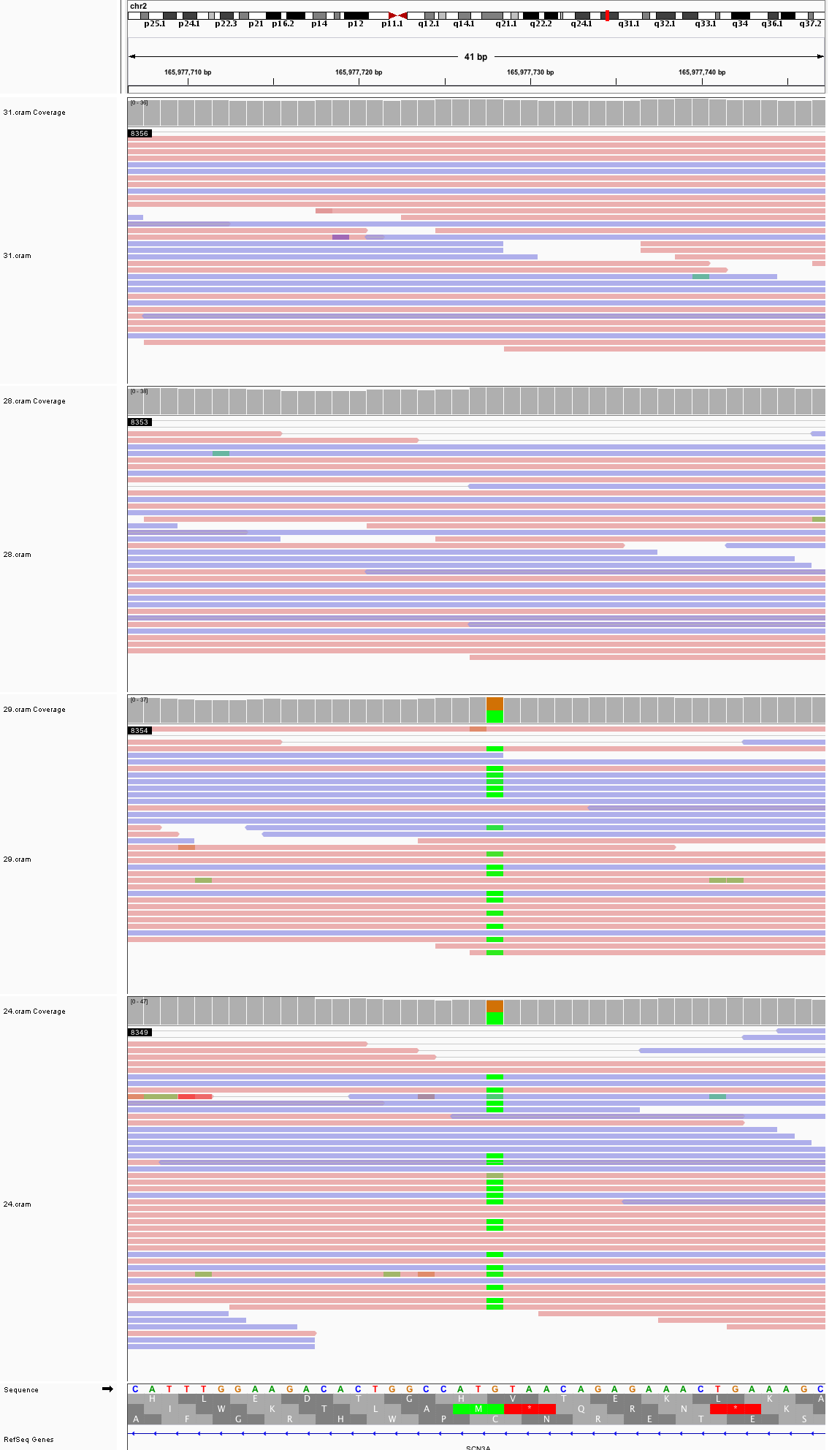

Supplement: Supplementary file 4. — All tracks below contain alignments from the third-generation children that share a DNM at the site. Reads with mapping quality <20 are filtered out, as they were not considered by our variant calling pipeline, and mismatched bases are shaded by quality score (more transparent = lower base quality). [file elife-46922-supp4.zip › supp_file_4/chr2_165,977,707_165,977,747.png]

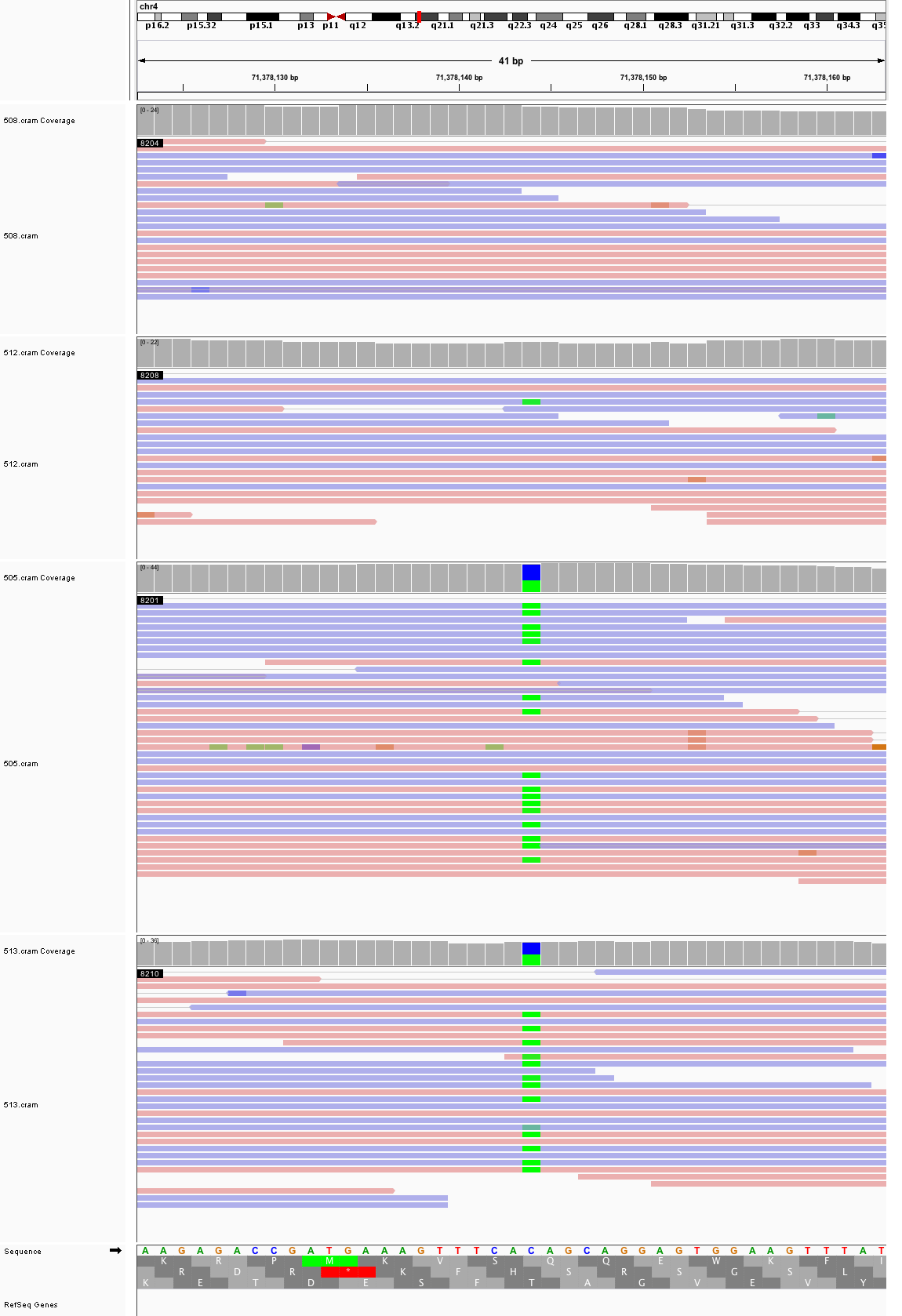

Supplement: Supplementary file 4. — All tracks below contain alignments from the third-generation children that share a DNM at the site. Reads with mapping quality <20 are filtered out, as they were not considered by our variant calling pipeline, and mismatched bases are shaded by quality score (more transparent = lower base quality). [file elife-46922-supp4.zip › supp_file_4/chr4_71,378,123_71,378,163.png]

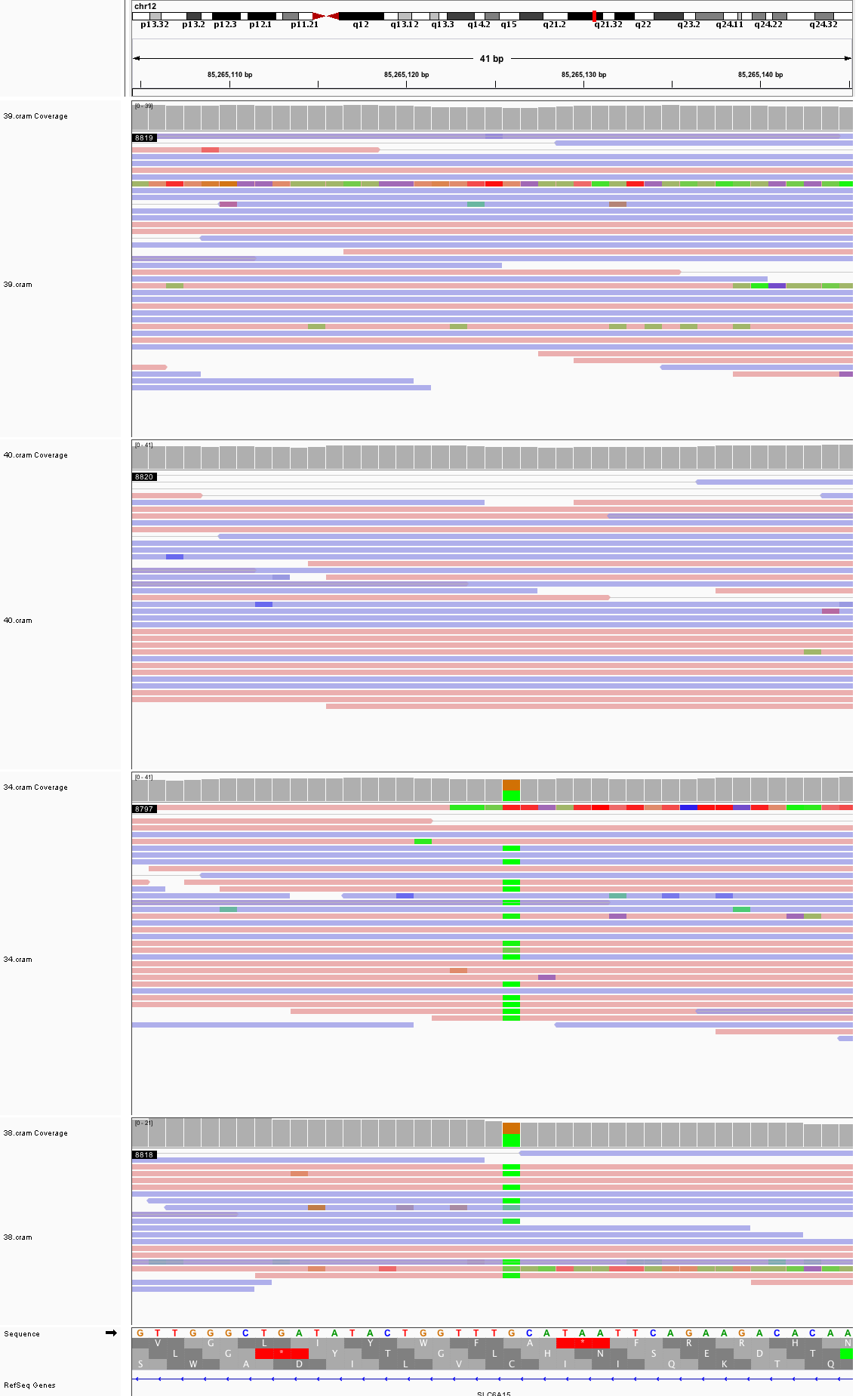

Supplement: Supplementary file 4. — All tracks below contain alignments from the third-generation children that share a DNM at the site. Reads with mapping quality <20 are filtered out, as they were not considered by our variant calling pipeline, and mismatched bases are shaded by quality score (more transparent = lower base quality). [file elife-46922-supp4.zip › supp_file_4/chr12_85,265,105_85,265,145.png]

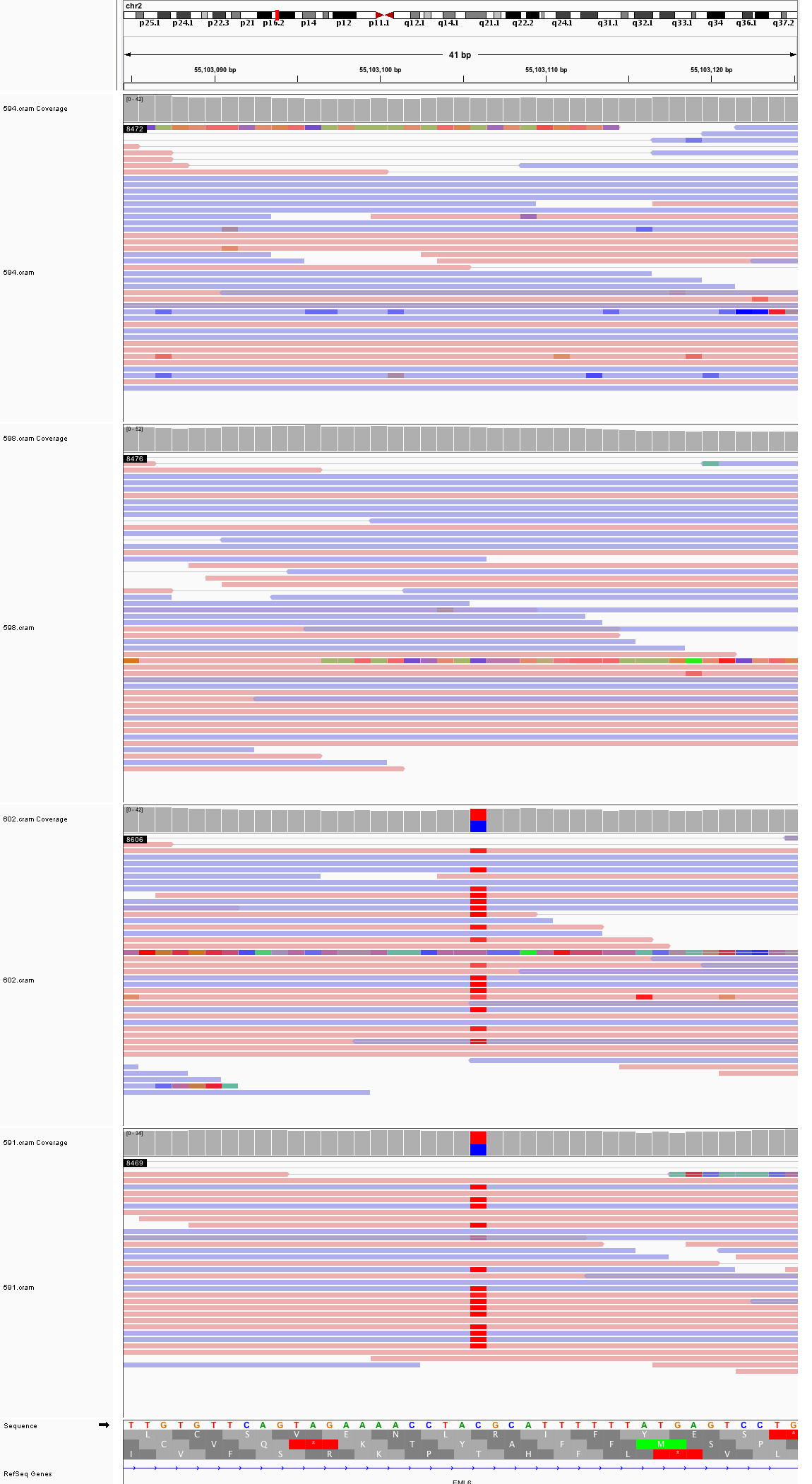

Supplement: Supplementary file 4. — All tracks below contain alignments from the third-generation children that share a DNM at the site. Reads with mapping quality <20 are filtered out, as they were not considered by our variant calling pipeline, and mismatched bases are shaded by quality score (more transparent = lower base quality). [file elife-46922-supp4.zip › supp_file_4/chr2_55,103,085_55,103,125.png]

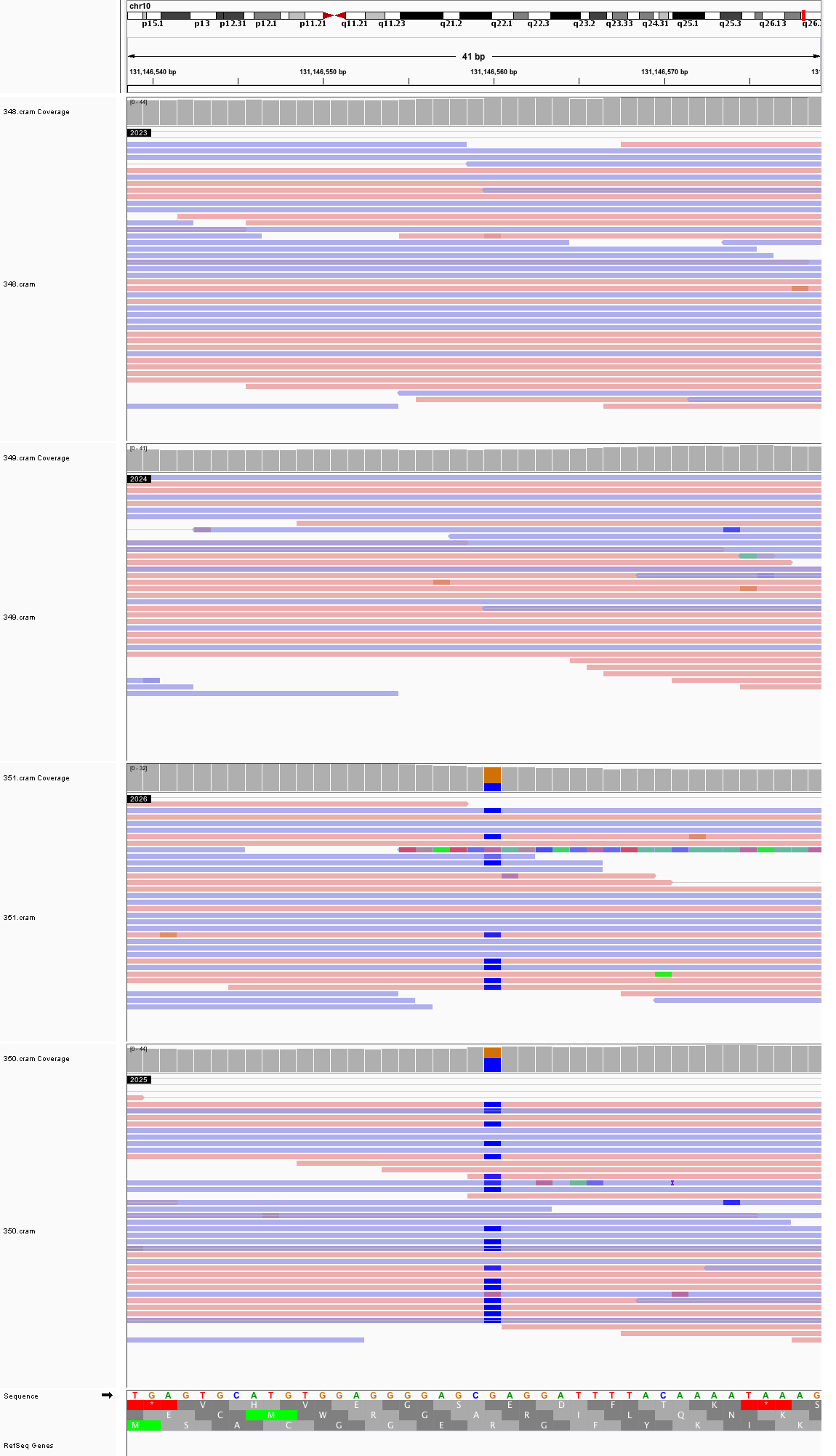

Supplement: Supplementary file 4. — All tracks below contain alignments from the third-generation children that share a DNM at the site. Reads with mapping quality <20 are filtered out, as they were not considered by our variant calling pipeline, and mismatched bases are shaded by quality score (more transparent = lower base quality). [file elife-46922-supp4.zip › supp_file_4/chr10_131,146,539_131,146,579.png]

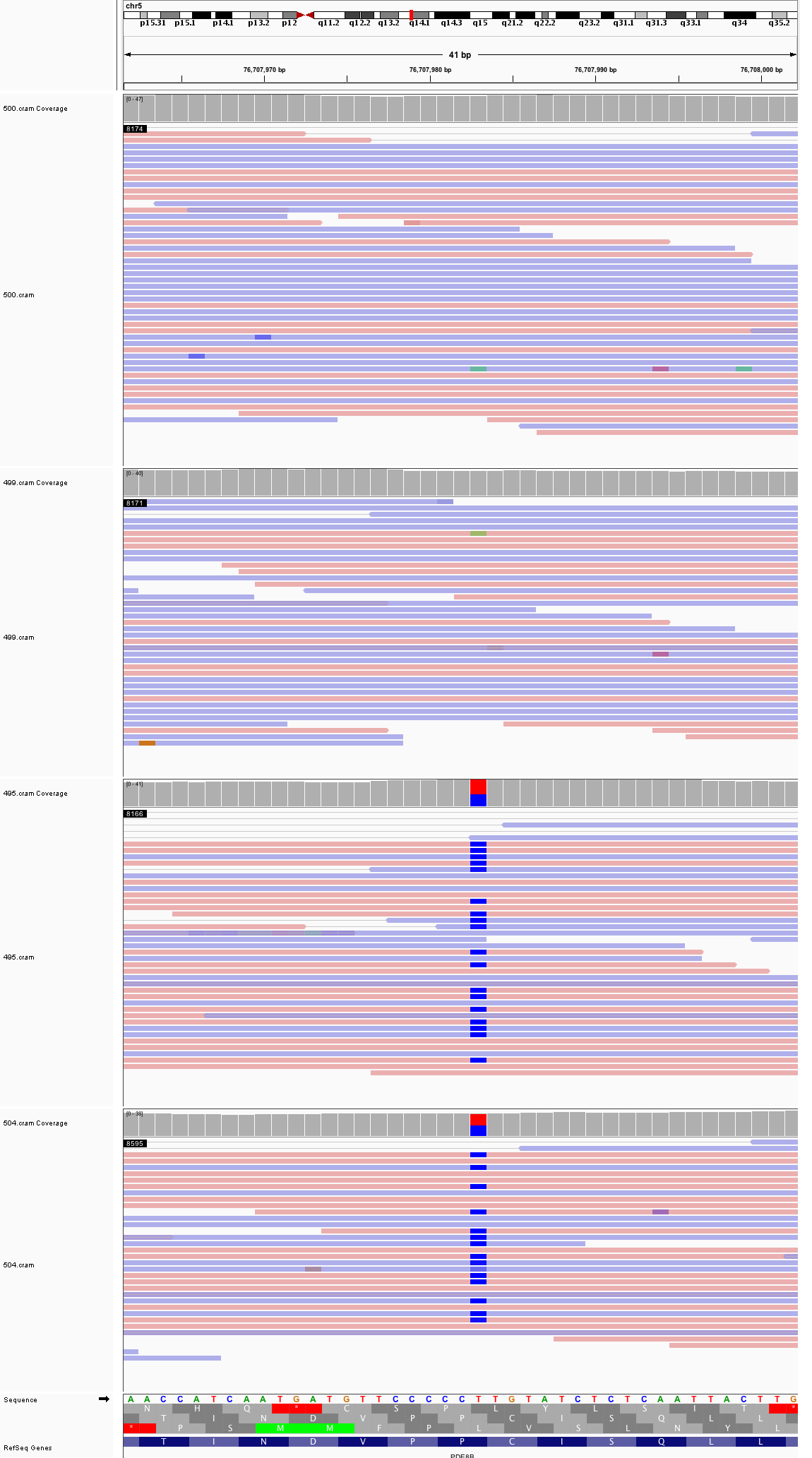

Supplement: Supplementary file 4. — All tracks below contain alignments from the third-generation children that share a DNM at the site. Reads with mapping quality <20 are filtered out, as they were not considered by our variant calling pipeline, and mismatched bases are shaded by quality score (more transparent = lower base quality). [file elife-46922-supp4.zip › supp_file_4/chr5_76,707,962_76,708,002.png]

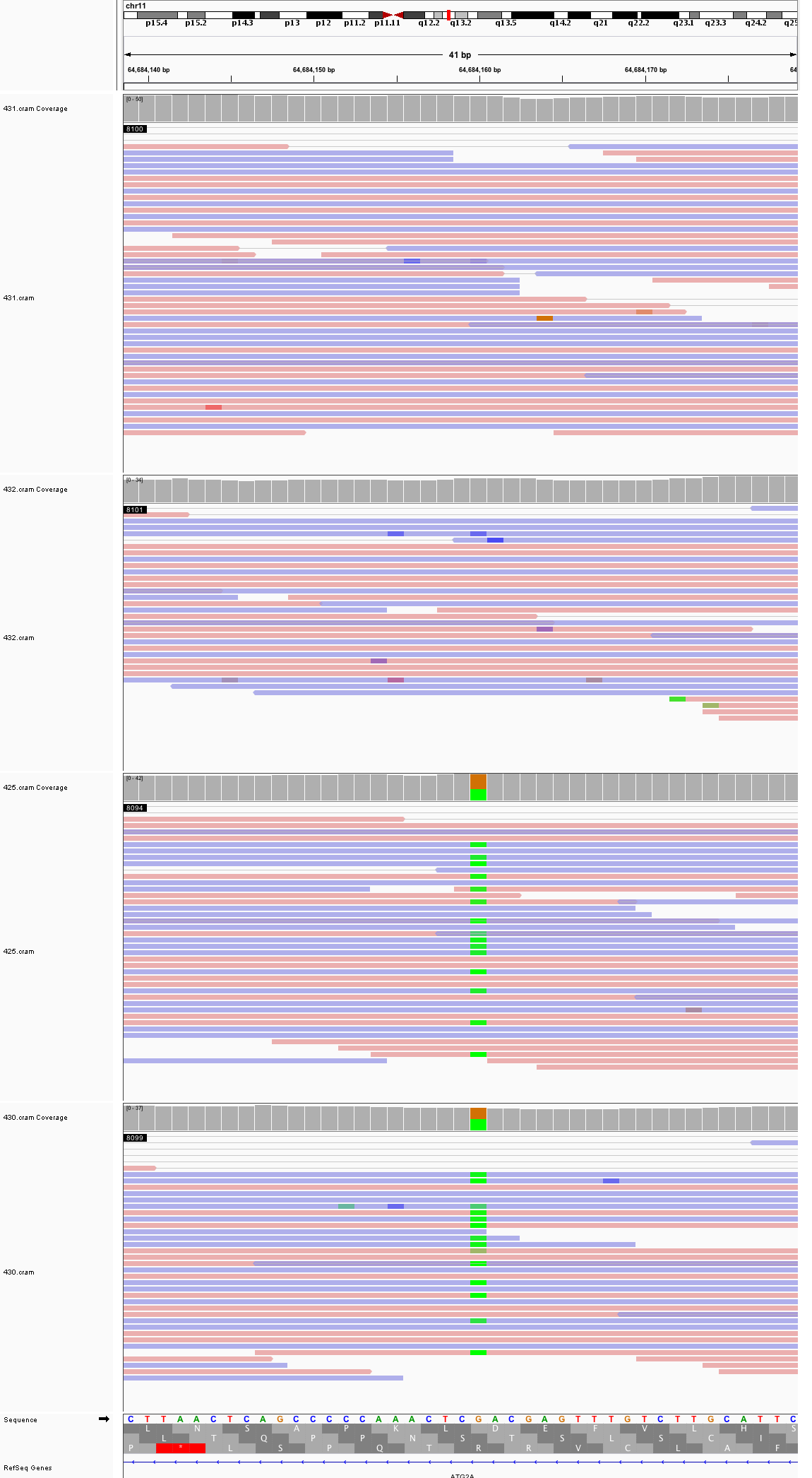

Supplement: Supplementary file 4. — All tracks below contain alignments from the third-generation children that share a DNM at the site. Reads with mapping quality <20 are filtered out, as they were not considered by our variant calling pipeline, and mismatched bases are shaded by quality score (more transparent = lower base quality). [file elife-46922-supp4.zip › supp_file_4/chr11_64,684,139_64,684,179.png]

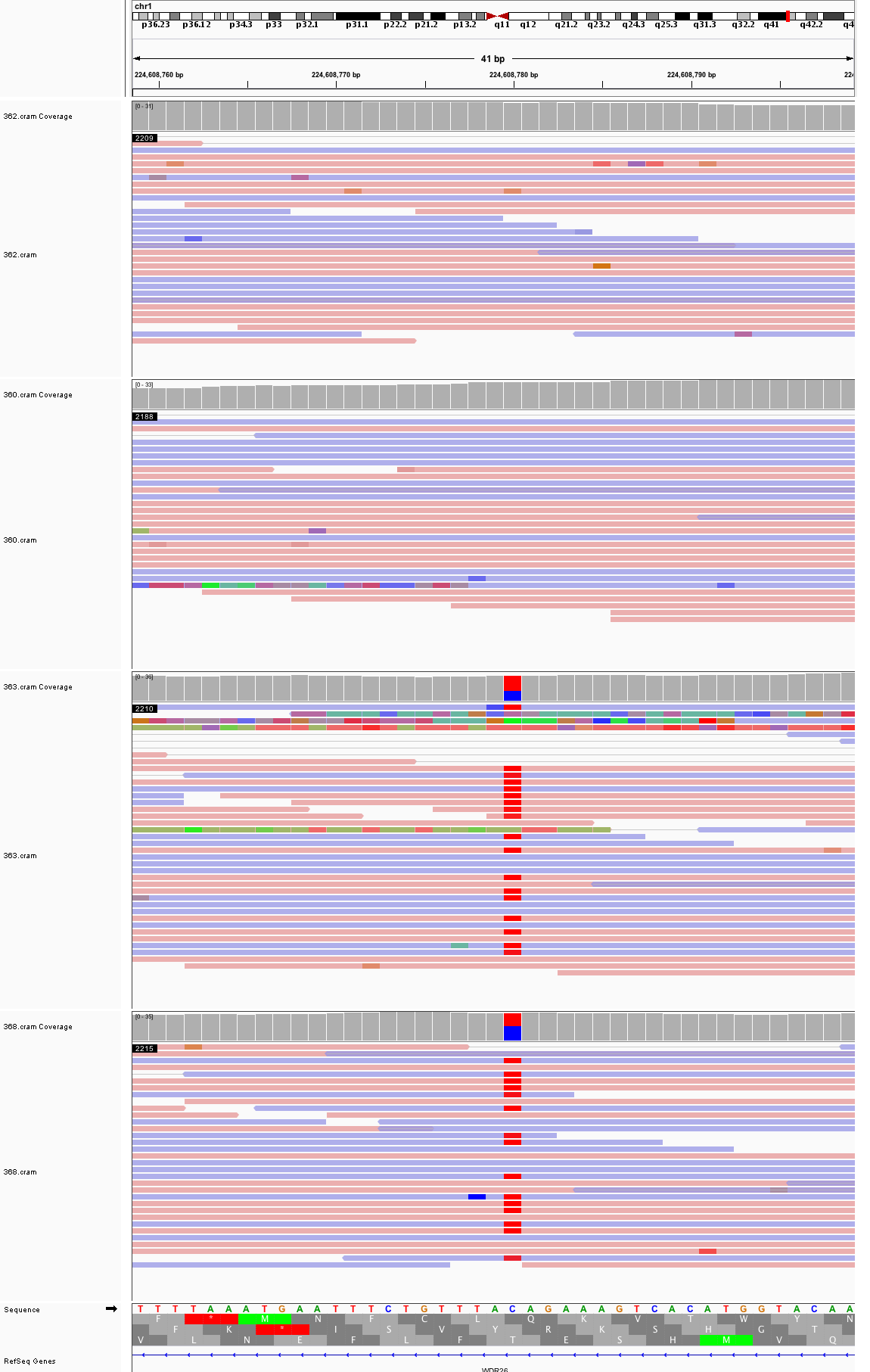

Supplement: Supplementary file 4. — All tracks below contain alignments from the third-generation children that share a DNM at the site. Reads with mapping quality <20 are filtered out, as they were not considered by our variant calling pipeline, and mismatched bases are shaded by quality score (more transparent = lower base quality). [file elife-46922-supp4.zip › supp_file_4/chr1_224,608,759_224,608,799.png]

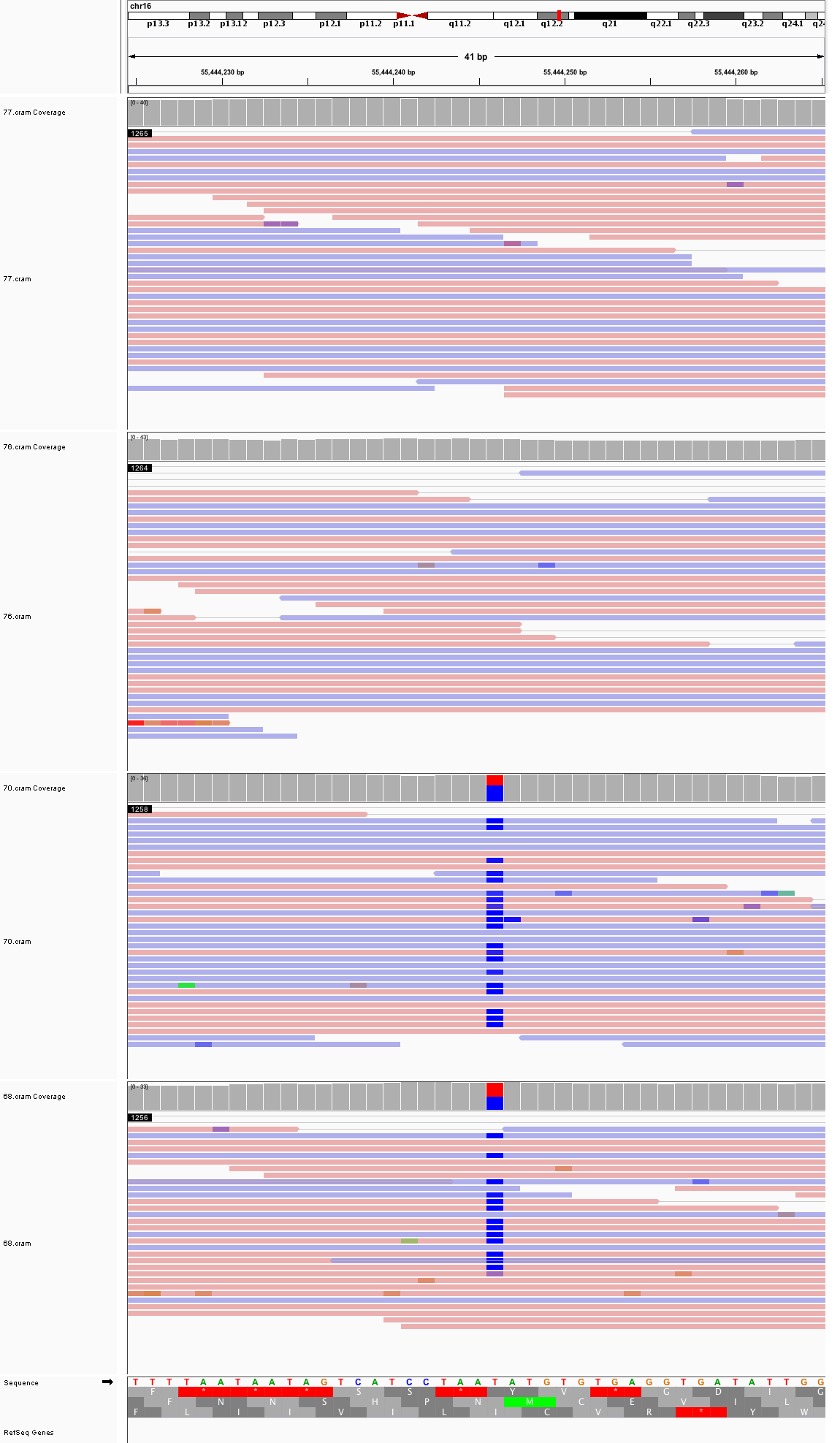

Supplement: Supplementary file 4. — All tracks below contain alignments from the third-generation children that share a DNM at the site. Reads with mapping quality <20 are filtered out, as they were not considered by our variant calling pipeline, and mismatched bases are shaded by quality score (more transparent = lower base quality). [file elife-46922-supp4.zip › supp_file_4/chr16_55,444,225_55,444,265.png]

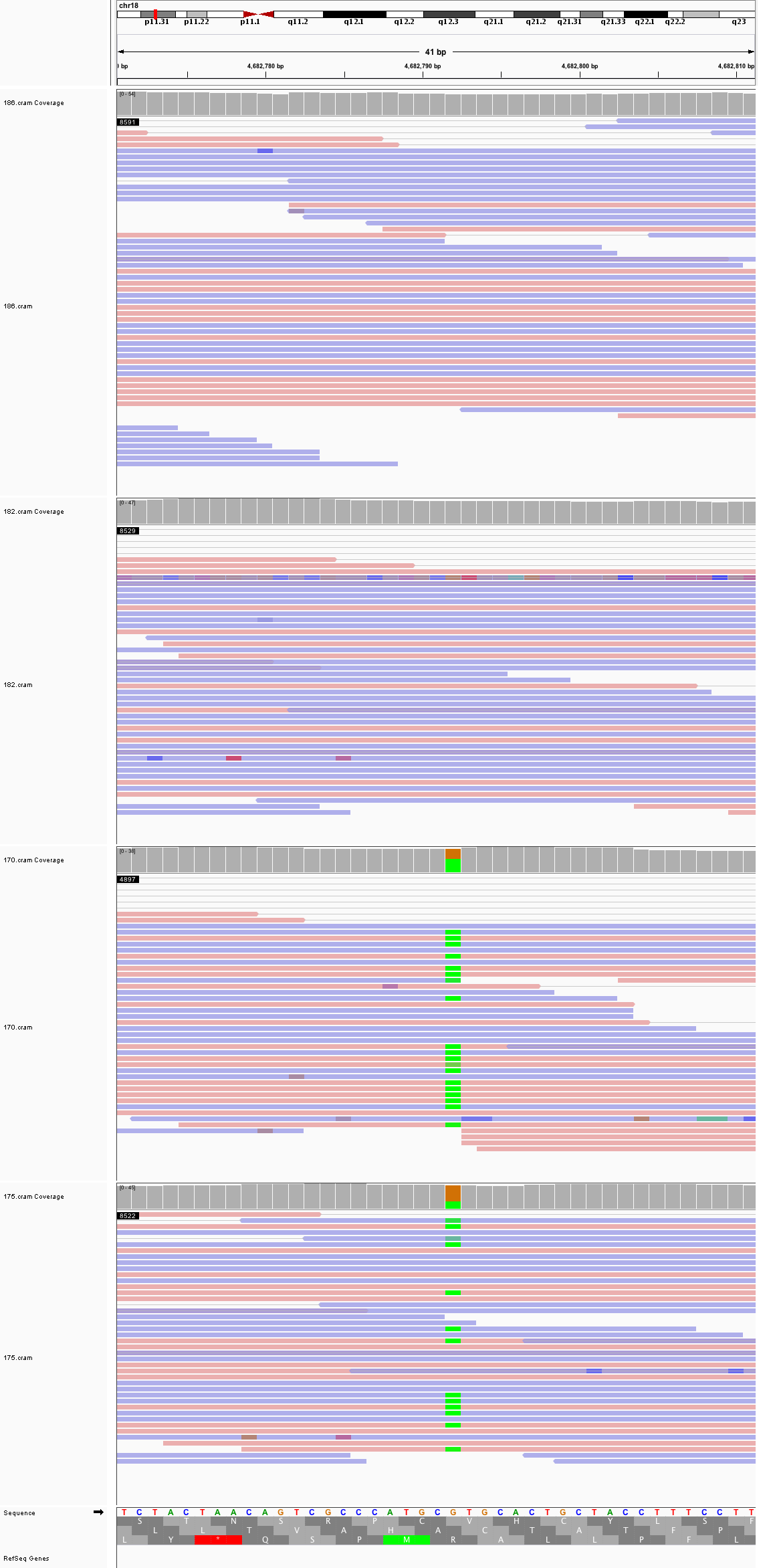

Supplement: Supplementary file 4. — All tracks below contain alignments from the third-generation children that share a DNM at the site. Reads with mapping quality <20 are filtered out, as they were not considered by our variant calling pipeline, and mismatched bases are shaded by quality score (more transparent = lower base quality). [file elife-46922-supp4.zip › supp_file_4/chr18_4,682,771_4,682,811.png]

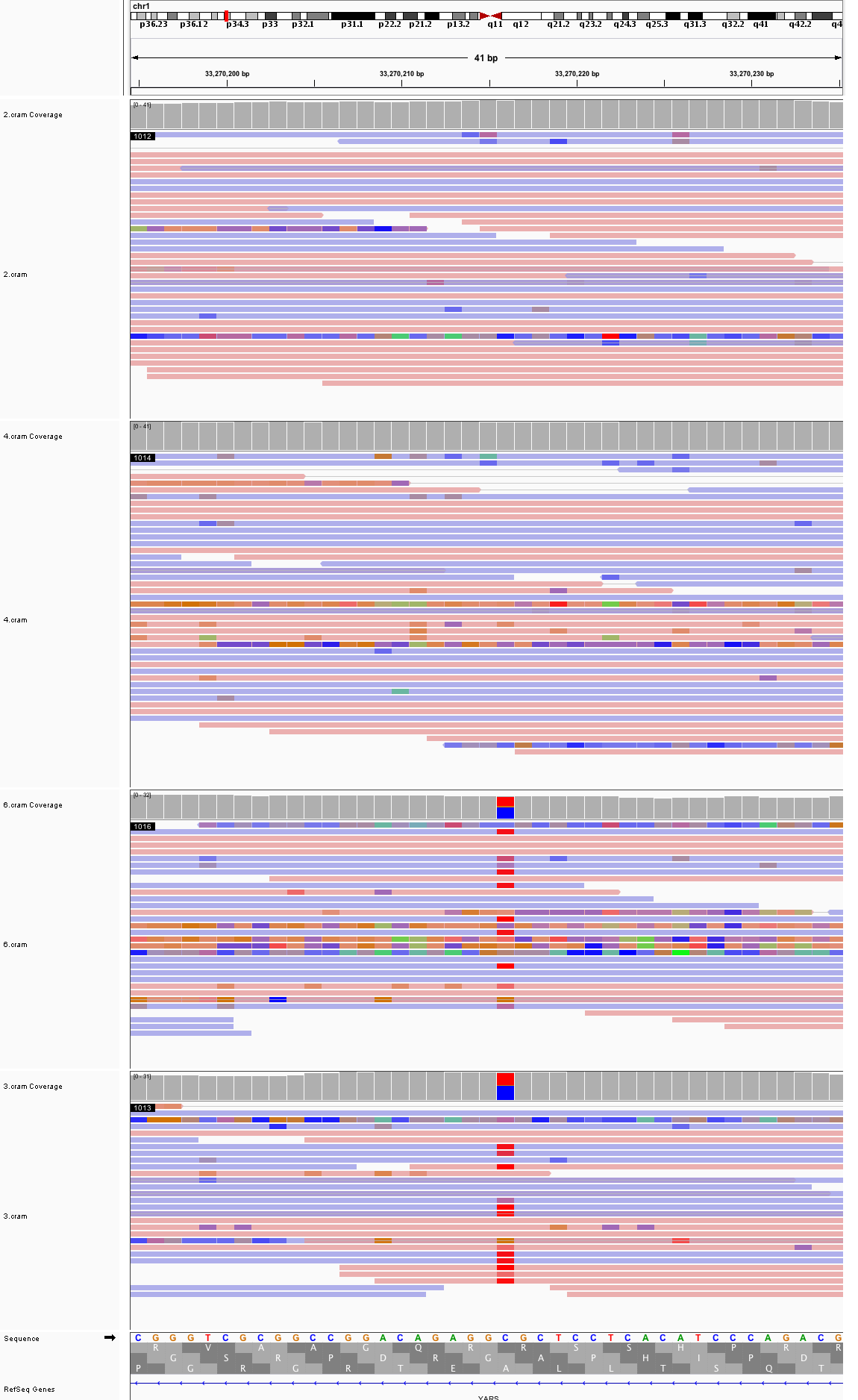

Supplement: Supplementary file 4. — All tracks below contain alignments from the third-generation children that share a DNM at the site. Reads with mapping quality <20 are filtered out, as they were not considered by our variant calling pipeline, and mismatched bases are shaded by quality score (more transparent = lower base quality). [file elife-46922-supp4.zip › supp_file_4/chr1_33,270,195_33,270,235.png]

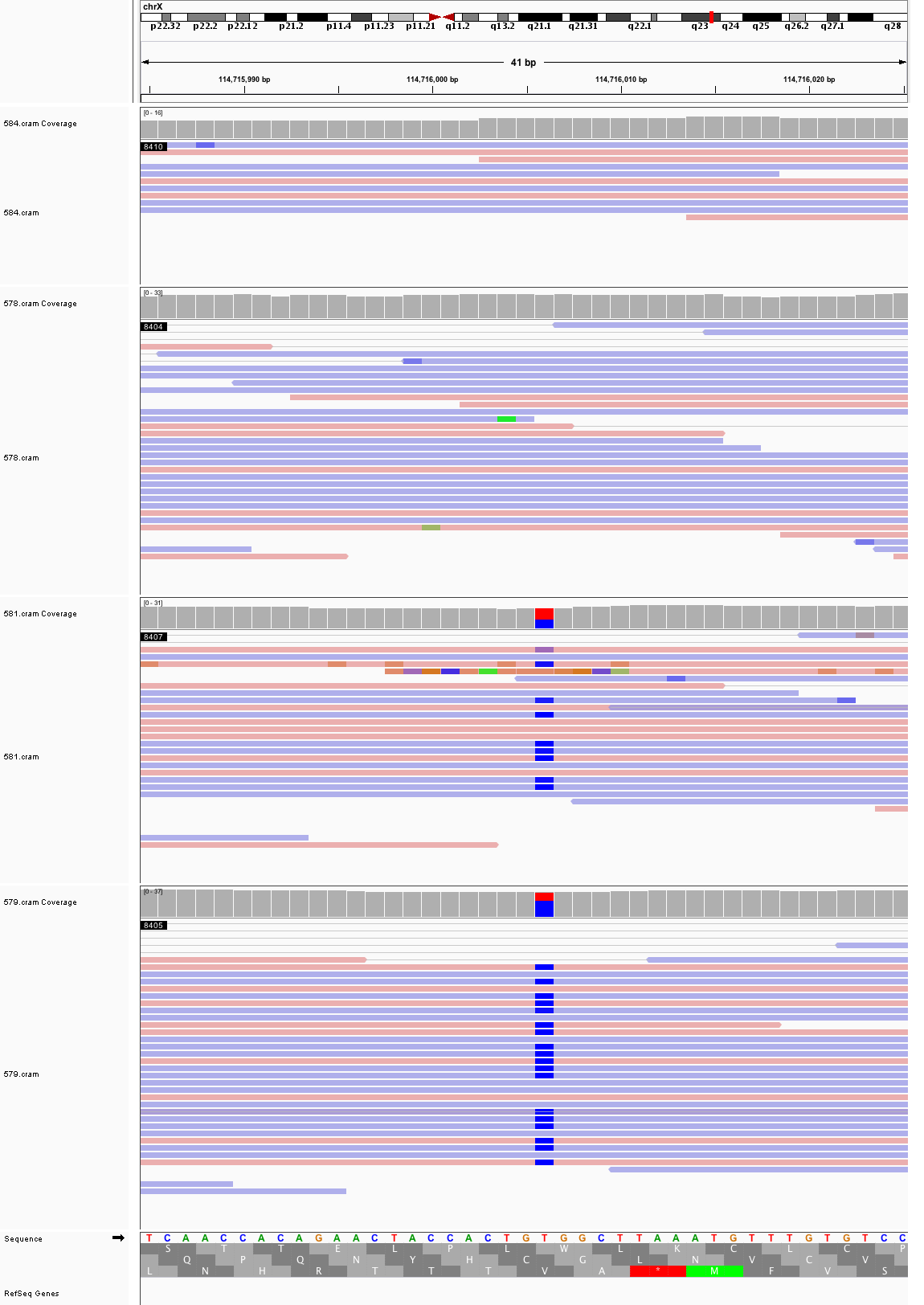

Supplement: Supplementary file 4. — All tracks below contain alignments from the third-generation children that share a DNM at the site. Reads with mapping quality <20 are filtered out, as they were not considered by our variant calling pipeline, and mismatched bases are shaded by quality score (more transparent = lower base quality). [file elife-46922-supp4.zip › supp_file_4/chrX_114,715,985_114,716,025.png]
