# Supplementary material for: Large, three-generation human families reveal post-zygotic mosaicism and variability in germline mutation accumulation
Source: eLife. 2019 Sep 24;8:e46922. doi: 10.7554/eLife.46922 (PMC6759356; doi:10.7554/eLife.46922)
Supplement: Transparent reporting form [file elife-46922-transrepform.pdf]

eLIFE
1st Floor
24 Hills Road
Cambridge CB2 1JP, UK
P 01223 855340
W elifesciences.org
T @elife

eLife's transparent reporting form

We encourage authors to provide detailed information *within their submission* to facilitate the interpretation and replication of experiments. Authors can upload supporting documentation to indicate the use of appropriate reporting guidelines for health-related research (see EQUATOR Network), life science research (see the BioSharing Information Resource), or the ARRIVE guidelines for reporting work involving animal research. Where applicable, authors should refer to any relevant reporting standards documents in this form.

If you have any questions, please consult our Journal Policies and/or contact us: editorial@elifesciences.org.

Sample-size estimation

- You should state whether an appropriate sample size was computed when the study was being designed
- You should state the statistical method of sample size computation and any required assumptions
- If no explicit power analysis was used, you should describe how you decided what sample (replicate) size (number) to use

Please outline where this information can be found within the submission (e.g., sections or figure legends), or explain why this information doesn't apply to your submission:

No statistical methods were used to predetermine sample sizes in this study. Genome sequencing data was collected for a total of 603 CEPH/Utah individuals. As described in the Materials and Methods section, we excluded sequencing data from 10 of these samples due to high levels of heterozygosity, indicative of possible contamination prior to sequencing.

Replicates

- You should report how often each experiment was performed
- You should include a definition of biological versus technical replication
- The data obtained should be provided and sufficient information should be provided to indicate the number of independent biological and/or technical replicates
- If you encountered any outliers, you should describe how these were handled
- Criteria for exclusion/inclusion of data should be clearly stated
- High-throughput sequence data should be uploaded before submission, with a private link for reviewers provided (these are available from both GEO and ArrayExpress)

Please outline where this information can be found within the submission (e.g., sections or figure legends), or explain why this information doesn't apply to your submission:

We did not perform any biological or technical replicates in this analysis. As described above (and in the Materials and Methods section), we excluded sequencing data from 10 of the CEPH/Utah samples due to high levels of heterozygosity. All de novo mutation data is provided at the following URL: https://github.com/quinlan-lab/ceph-dnm-manuscript. Aligned BAM files, as well as a joint-called VCF file, will be made available on the SRA/dbGaP under controlled access prior to publication.

Statistical reporting

- Statistical analysis methods should be described and justified
- Raw data should be presented in figures whenever informative to do so (typically when N per group is less than 10)
- For each experiment, you should identify the statistical tests used, exact values of N, definitions of center, methods of multiple test correction, and dispersion and precision measures (e.g., mean, median, SD, SEM, confidence intervals; and, for the major substantive results, a measure of effect size (e.g., Pearson's r, Cohen's d)
- Report exact p-values wherever possible alongside the summary statistics and 95% confidence intervals. These should be reported for all key questions and not only when the p-value is less than 0.05.

Please outline where this information can be found within the submission (e.g., sections or figure legends), or explain why this information doesn't apply to your submission:

Statistical methods used to identify differences in mutation spectra, in addition to unadjusted p-values and sample sizes, are described in figure legends (Figures 2, 4, 5, and Figure 2—figure supplement 2). Methods used to fit generalized linear models and identify differences in rates of germline mutation accumulation (Figure 3) are described in the Results text, and in greater detail in the Materials and Methods section. Methods used to examine the relationship between parental age at conception and the numbers of germline (Figure 2), post-PGCS mutations (Figure 4), and gonosomal mutations (Figure 5), are provided in the figure legends and Materials and Methods section. Additionally, all code used to generate figures and perform statistical analyses is available in a set of annotated, executable Jupyter notebooks.

(For large datasets, or papers with a very large number of statistical tests, you may upload a single table file with tests, Ns, etc., with reference to sections in the manuscript.)

Group allocation

- Indicate how samples were allocated into experimental groups (in the case of clinical studies, please specify allocation to treatment method); if randomization was used, please also state if restricted randomization was applied
- Indicate if masking was used during group allocation, data collection and/or data analysis

Please outline where this information can be found within the submission (e.g., sections or figure legends), or explain why this information doesn't apply to your submission:

Samples were not allocated into experimental/control groups in this analysis. As described previously, 10 samples were removed from downstream analysis following quality control on the aligned sequencing data.

Additional data files ("source data")

- We encourage you to upload relevant additional data files, such as numerical data that are represented as a graph in a figure, or as a summary table
- Where provided, these should be in the most useful format, and they can be uploaded as "Source data" files linked to a main figure or table
- Include model definition files including the full list of parameters used
- Include code used for data analysis (e.g., R, MatLab)
- Avoid stating that data files are "available upon request"

Please indicate the figures or tables for which source data files have been provided:

All of the de novo mutation data, as well as interactive code to reproduce figures/statistical analyses, are available in tab-separated files and reproducible Jupyter notebooks, respectively, at the following URL: https://github.com/quinlan-lab/ceph-dnm-manuscript. Therefore, we have not explicitly included source data files in the manuscript.
